# ALDH1L2 induces resistance to chemotherapy in small cell lung cancer by inhibiting ferroptosis

**DOI:** 10.1016/j.redox.2026.104098

**Published:** 2026-02-23

**Authors:** Yueming Zhang, Ruibin Yi, Xinyi Zhou, Qiong Lyu, Huiying Liu, Yaru Zhu, Peng Luo, Weitao Shen, Jian Zhang

**Affiliations:** a Department of Oncology, Zhujiang Hospital, Southern Medical University, Guangzhou, Guangdong, 510280, China; b Department of Pathology, School of Basic Medical Science, Southern Medical University, Guangzhou, Guangdong, 510515, China; c Department of Critical Care Medicine, Zhujiang Hospital, Southern Medical University, Guangzhou, Guangdong, 510280, China

**Keywords:** Small cell lung cancer, Chemoresistance, ALDH1L2, Ferroptosis, PRDX3

## Abstract

Small cell lung cancer (SCLC) is known for its rapid growth and early metastasis, and SCLC patients are highly susceptible to chemoresistance. Studies have shown that the combination of ferroptosis induction and TRX pathway inhibition can significantly inhibit SCLC tumor growth, but the molecular mechanisms underlying ferroptosis in SCLC are poorly understood. In this study, we explored the regulatory role of the ALDH1L2-related metabolic pathway in SCLC chemoresistance by machine learning. We found that ALDH1L2 expression is a poor prognostic factor for SCLC patients and that high ALDH1L2 expression can negatively regulate the level of cellular lipid peroxidation and inhibit ferroptosis, thereby promoting SCLC chemoresistance. Mechanistically, ALDH1L2 interacts with the TRX2-PRDX3 antioxidant network to reduce the levels of hyperoxidized PRDX3 and oxidized PRDX3 dimers in the plasma membrane under cisplatin-induced stress and decrease cellular susceptibility to ferroptosis, thus promoting SCLC chemoresistance. In addition, we found that thiostrepton, a PRDX3 inhibitor, can synergize with chemotherapy to suppress tumor growth in SCLC, suggesting that thiostrepton might be a promising new tool for overcoming SCLC chemoresistance.

## Highlights

• ALDH1L2 is a poor prognostic factor and promotes SCLC chemoresistance.
• ALDH1L2 can reduce the level of cellular lipid peroxidation and inhibit ferroptosis, thus contributing to SCLC chemoresistance.
• ALDH1L2 can interact with the TRX2-PRDX3 antioxidant network and reduce the levels of hyperoxidized PRDX3 and oxidized PRDX3 dimers in the plasma membrane under cisplatin-induced stress, decreasing susceptibility to ferroptosis and promoting SCLC chemoresistance.
• The combination of chemotherapy and thiostrepton, a PRDX3 inhibitor, exerts synergistic therapeutic effects against SCLC in vivo.

## Graphical abstract

**Figure:**
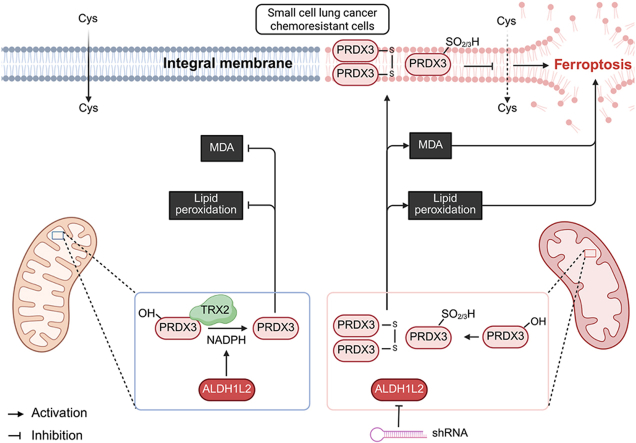

## 1 Introduction

Lung cancer is one of the malignant tumors with the highest morbidity and mortality worldwide [1,2]. Small cell lung cancer (SCLC) accounts for only 15-20% of all lung cancer cases and is among the deadliest malignant tumors because of its rapid growth, invasiveness, early metastasis, and extremely poor prognosis [1,3,4]. Only a small percentage of SCLC patients can receive curative pulmonary resection with adjuvant platinum-etoposide chemotherapy [1,3,4]. Therefore, for the vast majority of SCLC patients, treatment still relies mainly on chemotherapy, radiotherapy and immunotherapy [1,3,4]. Chemotherapy is the cornerstone of SCLC treatment, and patients are highly sensitive to initial chemotherapy [3,4]. However, the response to chemotherapy is short-lived, and the rapid development of chemoresistance leads to disease recurrence [[3], [4], [5]]. The 5-year survival rate of SCLC patients is less than 7% [3]. Therefore, elucidating the mechanisms involved in chemoresistance and exploring new therapeutic targets can provide new possibilities for the treatment of SCLC patients.

The induction of ferroptosis has been considered in an attempt to overcome the resistance of many tumors to current therapies. Lipocalin 2 (LCN2)-neutralizing antibody and phosphoseryl-tRNA kinase (PSTK) inhibitor increases the drug sensitivity of hepatocellular carcinoma cells to sorafenib through a ferroptosis-dependent mechanism [6,7]. Another study suggests that sorafenib fails to induce ferroptosis in multiple cancer cell lines, indicating that combining it with ferroptosis inducers may yield enhanced antitumor efficacy by triggering ferroptosis in tumor cells [8]. The concept of ferroptosis, a form of programmed cell death that is iron-dependent and driven by lipid peroxidation, was first formally proposed by Dixon et al. and published in *Cell* in 2012 [9]. Ferroptosis is characterized by intracellular iron overload and lipid peroxidation [9]. The morphology of mitochondria undergoes the most pronounced changes during ferroptosis, including mitochondrial shrinkage, increased mitochondrial membrane density, a reduction in mitochondrial cristae, and outer mitochondrial membrane rupture [[9], [10], [11]].

SCLC is a highly aggressive type of lung cancer, and its tumor heterogeneity poses a great challenge to treatment. Recent studies have shown that extrinsic apoptosis and necroptosis pathways are inactivated before treatment, and ferroptosis has become a targetable cell death pathway in SCLC [12]. RSL3, ML210, and erastin can induce ferroptosis in SCLC cells [12]. Although the remodeling of lipid metabolism that occurs in neuroendocrine (NE) and non-NE SCLC cells results in different sensitivities of cells to ferroptosis induction, the combination of ferroptosis induction and TRX pathway inhibition overcomes the challenge posed by intratumor heterogeneity to kill SCLC cells effectively [12]. However, the specific mechanism of ferroptosis in the chemoresistance of SCLC has not been completely elucidated, and studies are expected to provide new directions for overcoming chemoresistance and identifying new therapeutic targets in SCLC.

In this study, we performed a bioinformatics screen and revealed that the activity of the aldehyde dehydrogenase 1 family member l2 (ALDH1L2)-regulated metabolic pathway is positively correlated with cisplatin resistance in SCLC and that the expression of ALDH1L2 is correlated with poor prognosis. We then experimentally determined the significantly high expression of ALDH1L2 in chemoresistant SCLC cell lines. Furthermore, transmission electron microscopy revealed that chemoresistant SCLC cells exhibited morphological changes of mitochondria after ALDH1L2 knockdown.

ALDH1L2, also known as mitochondrial 10-formyltetrahydrofolate dehydrogenase, is a mitochondrial enzyme whose primary function is to convert 10-formyltetrahydrofolate (10-formyl-THF) to tetrahydrofolate (THF) and carbon dioxide (CO_2_) while generating reduced nicotinamide adenine dinucleotide phosphate (NADPH) [[13], [14], [15]]. ALDH1L2 is involved mainly in one-carbon metabolism and the buffering of cellular oxidative stress, which in turn affects mitochondrial function and tumor growth [14,16]. Current research on ALDH1L2 is limited, and no study has yet elucidated the specific mechanism underlying the relationship between ALDH1L2 and ferroptosis. ALDH1L2 affects radiotherapy resistance and 5-fluorouracil resistance in colorectal cancer [17,18], but no study has reported whether ALDH1L2 is involved in the regulation of chemoresistance in SCLC. On the basis of the results of our bioinformatics analysis, we aimed to further explore the mechanism through which ALDH1L2 regulates SCLC chemoresistance through ferroptosis.

We further used molecular docking to identify the potential interaction between ALDH1L2 protein and TRX2-PRDX3 pathway. Thioredoxin-2 (TRX2) can catalyze the dissociation of peroxiredoxin 3 (PRDX3) dimer into monomer, which requires the participation of thioredoxin reductase (TRXR2) and NADPH [19,20]. TRXR2 is an NADPH-dependent enzyme that transfers electrons from NADPH to TRX, reducing oxidized TRX (TRX-S_2_) to the reduced TRX (TRX-SH_2_) [19,20]. The reduced TRX then reacts with the disulfide bond in the PRDX3 dimer through the sulfhydryl (-SH) group at its active site, thereby restoring the catalytic activity of PRDX3 [19,20].

In this study, we uncovered a novel dynamic regulation pattern of ferroptosis in the development of chemoresistance in SCLC. Mechanistically, ALDH1L2 interacts with the TRX2-PRDX3 antioxidant network, reducing the levels of hyperoxidized PRDX3 and oxidized PRDX3 dimers in the plasma membrane, which in turn decreases susceptibility to ferroptosis. Our findings illuminate the complexity and targetability of ferroptosis regulatory network in chemoresistant tumor cells, potentially offering insights to guide the development of precision therapy to overcome chemoresistance in SCLC.

## 2 Materials and methods

### 2.1 Data sources

The transcriptomic data of 104 SCLC cell lines and their half maximal inhibitory concentration (IC50) for cisplatin were downloaded from the Genomics of Drug Sensitivity in Cancer (GDSC) database [21]. A total of 98 metabolic pathways were screened from the H, C2, and C5 gene sets of the MSigDB database (http://software.broadinstitute.org/gsea/msigdb/index.jsp) [22], which are summarized in Table S1. The SCLC clinical cohorts included two public cohorts and one local cohort. The George cohort was published by Julie George et al. in *Nature* in 2015 [23], and the TU-SCLC cohort was published by Qian Liu et al. in *Cell* in 2024 [24]. The local cohort consisted of 58 SCLC patients followed at Zhujiang Hospital of Southern Medical University. Informed consent was obtained from all patients before the collection of specimens. Ethics committee approval for the study was obtained from the Ethics Committee of Zhujiang Hospital of Southern Medical University.

### 2.2 Construction of machine learning models

In accordance with the standards of GDSC1, we defined SCLC cell lines with a cisplatin IC50 of less than 3 μM as relatively sensitive strains (n = 58) and others as relatively resistant strains (n = 46). Before modeling, 104 SCLC cell lines were randomly grouped (seed number = 35678), with 70% used as the training set and the remaining 30% used as the testing set. The model was first trained to predict the cisplatin IC50 values of SCLC cell lines using the gene transcriptome data of each metabolic pathway via the extreme gradient boosting (XGBoost) algorithm, and the meaningful gene features identified by filtering were entered into the least absolute shrinkage and selection operator (Lasso) regression to construct binary classification models to predict the cisplatin sensitivity (relatively sensitive/relatively resistant) of SCLC cell lines (seed number = 35678). Model performance was assessed by the area under the curve (AUC) obtained from the receiver operating characteristic (ROC) curves and the concordance index (C-index) of the clinical cohorts. The importance of each gene feature in the model was obtained by feature importance analysis. All the above analyses were performed using RStudio 2024.04.2 software.

### 2.3 Survival analysis, meta-analysis and correlation analysis

In the survival analysis, the survival time of SCLC patients is presented in months, and the outcome event was death. Survival analysis was performed on the SCLC clinical cohorts using the survival and survminer packages in R, and the optimal cutoff values were determined using the surv_cutpoint function. A meta-analysis of the survival analysis results of SCLC clinical cohorts was performed using the meta package in R to comprehensively evaluate the impact of ALDH1L2 on the prognosis of patients. Correlation analysis was used to assess the correlation between model scores and cisplatin IC50 in SCLC cell lines.

### 2.4 Cell line

The human-derived SCLC cell lines H69, H69AR and H446 were obtained from the American Type Culture Collection (ATCC). The H69AR cell line is a multidrug-resistant cell line. The cisplatin-resistant cell line H446DDP was induced by culturing H446 cells with increasing concentrations of cisplatin in our laboratory, as described previously [25]. The mouse-derived RP cell line was obtained from the Rb1^flox/flox^, Trp53^flox/flox^ (RP) spontaneous SCLC mouse model. All cells were cultured in RPMI-1640 medium (KeyGEN, China) supplemented with 10% (v/v) fetal bovine serum (Procell, China) and maintained at 37 °C in a 5% CO_2_ incubator.

### 2.5 Transcriptome sequencing

After washed with PBS buffer, cells were collected in the RNA later solution. High-throughput RNA sequencing was performed by Haplox Biotechnology (Shenzhen, China).

### 2.6 Differential gene expression analysis

H69 and H446 cells served as the control groups for the analysis. After low-count filtering and TMM normalization, the raw gene expression count data were subjected to differential gene expression analysis using the edgeR package. The results were visualized using the ggplot2 package. All the above analyses were performed using RStudio 2024.04.2 software.

### 2.7 Immunofluorescence

The cells were fixed with 4% paraformaldehyde, permeabilized with 0.3% Triton-X, and blocked with 10% normal goat serum. Cells were treated with primary antibodies overnight for immunofluorescence staining, followed by 1 h of labeling with secondary antibodies. Finally, DAPI staining was applied to the cell nuclei, and a confocal laser microscope (Nikon, Japan) was used to capture images.

### 2.8 RNA extraction and RT-qPCR analyses

Total RNA was extracted from cells with TRIzol (ECOTOP SCIENTIFIC, China), followed by sequential purification with chloroform, isopropanol, and 70% ethanol. An ultraviolet spectrophotometer was used to measure the RNA concentration after the total RNA was dissolved in RNase-free water. RNA samples were reverse transcribed into cDNA using an AG reverse transcription kit (Accurate Biology, China), and quantitative analysis of RNA expression was performed with a SYBR Green qPCR kit (Accurate Biology, China). The primer sequences employed in this study are listed in Table S2.

### 2.9 Immunoblotting

Total protein was extracted using RIPA lysis buffer supplemented with 1% protease and phosphatase inhibitors. After the protein concentration was measured by the BCA method, protein loading buffer was added, and the samples were boiled for 10 min. During electrophoresis, SDS-PAGE (12% gels) was used to separate proteins of varying molecular weights, which were then transferred to PVDF membranes. The PVDF membranes were subsequently blocked with 5% skim milk, incubated with primary antibodies at 4 °C overnight, and then incubated with the secondary antibody for 1.5 h. The protein bands were subsequently detected using an ECL Chemiluminescence Kit, and the proteins were imaged using a chemiluminescence detection system (UVITEC, UK) and stored.

### 2.10 Cell transfection

The cells were transiently transfected with plasmid DNA using a GenJet Plus in vitro DNA transfection kit (SignaGen Laboratories), and the medium used for transfection was exchanged for fresh medium 12-18 h post transfection. The cells were transfected with siRNA using a PepMute siRNA Transfection Kit (SignaGen Laboratories), and the medium used for transfection was exchanged for fresh medium 6 h post transfection. For lentiviral transduction experiments, lentiviral vectors containing shALDH1L2 and overexpressing ALDH1L2 sequences (Shanghai Jikai Gene, China) were used to inoculate target cells. The medium used for transduction was exchanged for fresh medium 16 h post transfection. The stable cell lines were screened with 2 μg/mL puromycin. The sequences of the siRNAs, shRNAs and plasmids used in this study are summarized in Tables S3–S5.

### 2.11 Cytotoxicity assay

A CCK-8 assay was used to evaluate the cytotoxic effects of drugs on SCLC cells. Cells to be assayed were seeded in 96-well plates at a density of 10^4^ cells per well. The negative control was the group of cells without drug treatment. The other cells were treated with a gradient of drug concentrations for 24 h. The culture medium was subsequently replaced with 10% CCK-8 (GLPBIO, Shanghai, China) solution in culture medium. After incubation at 37 °C for one to 4 h, the absorbance at 450 nm was measured with a microplate reader. The data were adjusted by subtracting the background absorbance and then normalized against the no-treatment control. The IC50 values of the drugs were calculated by SPSS 27 software.

### 2.12 Immunohistochemistry

Fresh tumor tissues were fixed in 4% paraformaldehyde, embedded in paraffin, and cut into 5 μm slices. After the samples were incubated with primary and secondary antibodies, immunohistochemical staining was assessed under a microscope (Leica DM2500, Germany).

### 2.13 Transmission electron microscopy assay

Cells were fixed in 2.5% glutaraldehyde solution, followed by embedding, sectioning, and staining using uranium acetate and lead citrate. Finally, the cells were observed under a transmission electron microscope (HT7800, HITACHI).

### 2.14 Lipid peroxidation assay

To test lipid peroxidation, an Image-iT Lipid Peroxidation Kit from Life Technologies (Invitrogen) was used. Cells were pretreated with cisplatin (5 μg/mL for chemosensitive cell lines and 10 μg/mL for chemoresistant cell lines) in complete medium supplemented with 10% serum for 24 h prior to the assay. Finally, the samples were detected under the FITC and PE channels of a CytoFLEX flow cytometer (Beckman, USA).

### 2.15 Malondialdehyde detection

The malondialdehyde content was measured using a Lipid Peroxidation MDA Assay Kit (Beyotime, Shanghai, China). Cells were pretreated with cisplatin (5 μg/mL for chemosensitive cell lines and 10 μg/mL for chemoresistant cell lines) in complete medium supplemented with 10% serum for 24 h prior to the assay. The concentration of proteins was assessed using the BCA assay. The MDA content of the samples was calculated and expressed as nmol/mg protein.

### 2.16 NADPH quantification and NADPH/NADP^+^ ratio determination

Prior to the assay, cells were either left untreated or exposed to 10 μg/mL cisplatin for 24 h or 20 μM erastin for 72 h. Total NADP^+^/NADPH pools and NADPH content were quantified using an enhanced NADP^+^/NADPH Assay Kit (Beyotime, Shanghai, China) according to the manufacturer's instructions. After protein concentration was quantified by BCA assay, NADPH content and the NADPH/NADP^+^ ratio were expressed per milligram of protein.

### 2.17 Isolation of mitochondria

Prior to the assay, cells were either left untreated or exposed to 10 μg/mL cisplatin for 24 h or 20 μM erastin for 72 h. Mitochondria were isolated from cells using a Mitochondria Isolation Kit (Thermo Scientific, 89874) according to the manufacturer's instructions.

### 2.18 Molecular docking

The PDB file containing the predicted 3D structure of the ALDH1L2 protein was downloaded from the AlphaFold Protein Structure Database, and the PDB files of the 3D structures of PRDX3 (PDB ID: 5UCX) and TRX2 (PDB ID: 1UVZ) proteins were downloaded from the Protein Data Bank (PDB) database. HADDOCK 2.4 was used for protein-protein docking to generate different binding modes by rotation and translation of each molecule. The optimal conformations for each docking process were chosen on the basis of the lowest binding energies, which indicates the predicted stability of binding modes. The docking results were visualized using PyMOL 2.5.5.

### 2.19 Co-immunoprecipitation assay

Co-immunoprecipitation assays were performed using a Pierce Crosslink Magnetic IP/Co-IP kit (Thermo Scientific, 88805). Bait proteins and their binding partners were obtained from total cell extracts and subjected to immunoblotting.

### 2.20 Reaction of PRDX3 with hydroperoxides

The purified PRDX3 protein was obtained from MedChemExpress (HY–P71147, MCE, USA). The oxidation reaction was carried out at room temperature for 1 min in a mixture containing 0.5 μg/μL PRDX3 purified protein and 1 mM H_2_O_2_, quenched with 100 mM N-ethylmaleimide, and analyzed by SDS-PAGE.

### 2.21 Protein isolation

Subcellular fractionation was performed using a Minute™ Protein Isolation and Cell Fractionation Kit (total: SD-001/SN-002; plasma membrane: SM-005, Invent Biotechnologies, USA) according to the manufacturer's instructions, yielding total, cytosolic, plasma membrane, and organelle (including mitochondrial) protein extracts that were analyzed by SDS-PAGE.

### 2.22 Construction of orthotopic SCLC model

The orthotopic SCLC model was constructed using female C57BL/6 mice and RP cell line. For orthotopic injection, each mouse was injected with 10^6^ RP cells in the left lung under isoflurane anesthesia. Tumor growth was tracked using bioluminescence imaging with PerkinElmer in vivo imaging system (IVIS). After the tumors were successfully generated, the mice were randomly divided into two groups: a chemotherapy group and a combination group. The treatment regimen for the mice in the chemotherapy group consisted of cisplatin and etoposide. Cisplatin was administered at a dosage of 2 mg/kg once every 8 days by intraperitoneal injection. Etoposide was administered at a dosage of 4 mg/kg once every 2 days by intraperitoneal injection. The mice in the combination group were treated with the PRDX3 inhibitor thiostrepton combined with chemotherapy. Cisplatin was administered at a dosage of 2 mg/kg once every 8 days by intraperitoneal injection. Etoposide was administered at a dosage of 4 mg/kg once every 2 days by intraperitoneal injection. Thiostrepton was administered at a dosage of 17 mg/kg three times a week by intraperitoneal injection.

### 2.23 Construction of subcutaneous xenograft tumor model

The subcutaneous xenograft tumor model was constructed using female BALB/c nude mice and human-derived SCLC cell lines. To construct the xenograft tumor model, each mouse was injected subcutaneously with 5 × 10^6^ target cells. When the tumors reached approximately 100 mm^3^, the nude mice were randomly grouped according to the experimental arrangement. Cisplatin was administered at a dosage of 2 mg/kg once every 8 days by intraperitoneal injection. Etoposide was administered at a dosage of 4 mg/kg once every 2 days by intraperitoneal injection. Thiostrepton was administered at a dosage of 17 mg/kg three times a week by intraperitoneal injection. The long and short diameters of the tumors were regularly measured using a vernier caliper, and the tumor volume was calculated by the following formula: tumor volume = (long diameter × short diameter^2^)/2.

### 2.24 Statistical methods

All experiments in this study were repeated independently three times. GraphPad Prism 9 software was used to perform the statistical analyses and generate graphical representations of the raw data. An independent samples *t*-test was employed to compare the means between two groups, while ANOVA was used to compare the means of more than two groups. The data are expressed as mean ± standard deviation. In this study, p values are indicated by ∗ for p < 0.05; ∗∗ for p < 0.01; ∗∗∗ for p < 0.001; ∗∗∗∗ for p < 0.0001; and ns for not significant.

## 3 Results

### 3.1 Machine learning reveals that the activity of water-soluble vitamins and cofactors metabolism pathway is positively correlated with cisplatin resistance in SCLC

**Fig. 1:**
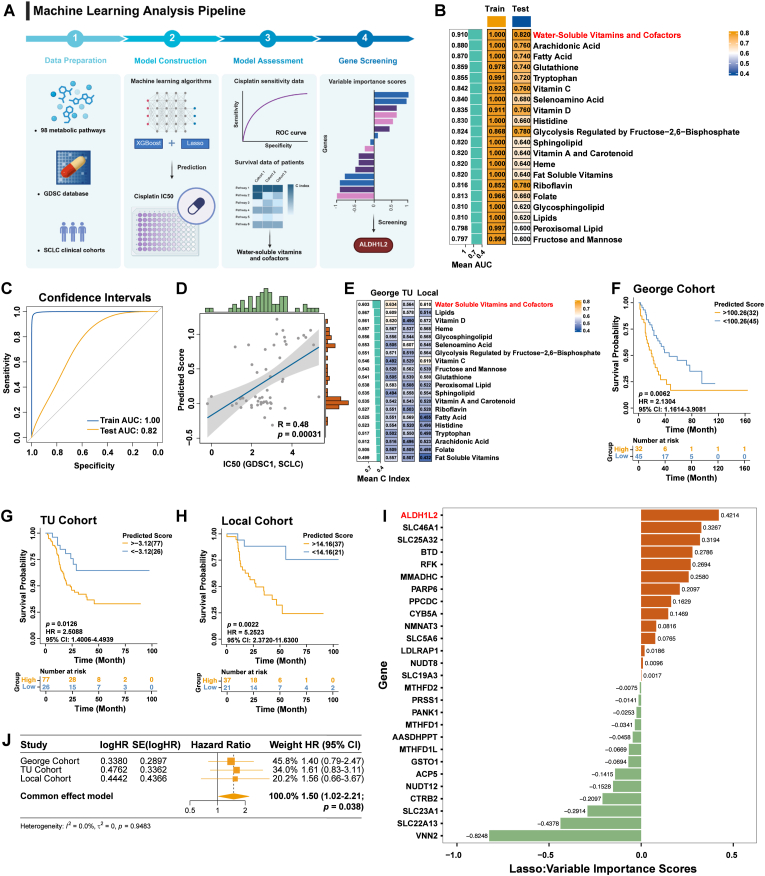
Water-soluble vitamins and cofactors metabolic pathway positively regulates SCLC chemoresistance. (A) Machine learning workflow. (B) Heatmap shows the AUC values of the top 20 metabolic models in the training set and testing set, and the left bar shows the arithmetic average AUC value of each metabolic model in the training set and testing set. (C) ROC curves of the water-soluble vitamins and cofactors metabolic model in the training set and testing set. The blue curve represents the ROC curve of the training set (AUC = 1.00), and the yellow curve represents the ROC curve of the testing set (AUC = 0.82). (D) Spearman correlation analysis of the scores of the water-soluble vitamins and cofactors metabolism model and the IC50 values for cisplatin in SCLC cell lines. (E) C-index of the 20 metabolic models with the highest arithmetic mean AUC values. The main body of the heatmap shows the C-index of the George cohort, TU-SCLC cohort, and local cohort, and the bar on the left side shows the arithmetic mean of the C-index of each metabolic model in the SCLC clinical cohorts. (F–H) Kaplan-Meier survival curves for overall survival in the George cohort (F), TU-SCLC cohort (G), and local cohort (H). The yellow curves represent SCLC patients with higher water-soluble vitamins and cofactors metabolism model scores, and the blue curves represent SCLC patients with lower water-soluble vitamins and cofactors metabolism model scores. (I) Feature importance analysis of the water-soluble vitamins and cofactors metabolism model. The red bars represent positive feature importance scores, and the green bars represent negative feature importance scores. (J) Forest plot demonstrating the result of the meta-analysis of the prognostic value of ALDH1L2 in clinical cohorts. AUC, area under the curve; ROC, receiver operating characteristic; IC50, half maximal inhibitory concentration; C-index, concordance index; HR, hazard ratio; CI, confidence interval.

The present study focused initially on metabolic reprogramming in chemoresistant SCLC cells, for which we identified 98 metabolism-related signaling pathways from the MSigDB database and summarized them in Table S1. The XGBoost algorithm in combination with lasso regression was applied to construct prediction models of metabolic pathway genes to predict cisplatin resistance in SCLC cell lines (Fig. 1A). Among them, the water-soluble vitamins and cofactors metabolism model performed best in predicting cisplatin resistance in SCLC cell lines (AUC of 1 for the training set and AUC of 0.82 for the testing set; Fig. 1B-C; complete results in Fig. S1A). Correlation analysis revealed that the scores of the water-soluble vitamins and cofactors metabolism model were positively correlated with the IC50 values of cisplatin in SCLC cell lines (R = 0.48, p = 0.00031; Fig. 1D). When the prediction models with the top 20 mean AUC values were applied to the SCLC clinical cohorts, the water-soluble vitamins and cofactors metabolism model also achieved the highest C-index in predicting the clinical outcomes of SCLC patients (mean C-index = 0.603; Fig. 1E). Survival analyses of the George cohort (HR = 2.1304, p = 0.0062; Fig. 1F), TU-SCLC cohort (HR = 2.5088, p = 0.0126; Fig. 1G), and local cohort (HR = 5.2523, p = 0.0022; Fig. 1H) revealed that the higher the model score, the worse the prognosis for patient survival.

The water-soluble vitamins and cofactors metabolism pathway consists of 127 genes (Table S1), and the final prediction model contained 27 genes. Feature importance analysis revealed that ALDH1L2 exhibited the highest positive contribution to the resistance of SCLC cell lines to cisplatin (feature weight = 0.4214; Fig. 1I). We subsequently determined the hazard ratio (HR) and 95% confidence interval (CI) of ALDH1L2 expression for overall survival in clinical cohorts by survival analysis (Fig. S1B–S1D), and further meta-analysis revealed that high expression of ALDH1L2 was a poor predictor of prognosis for SCLC patients (HR = 1.5, p = 0.038; Fig. 1J). The higher the ALDH1L2 expression, the worse the prognosis of SCLC patients. Overall, these results indicated that ALDH1L2 expression was positively correlated with the chemoresistance of SCLC.

### 3.2 ALDH1L2 promotes chemoresistance in SCLC

**Fig. 2:**
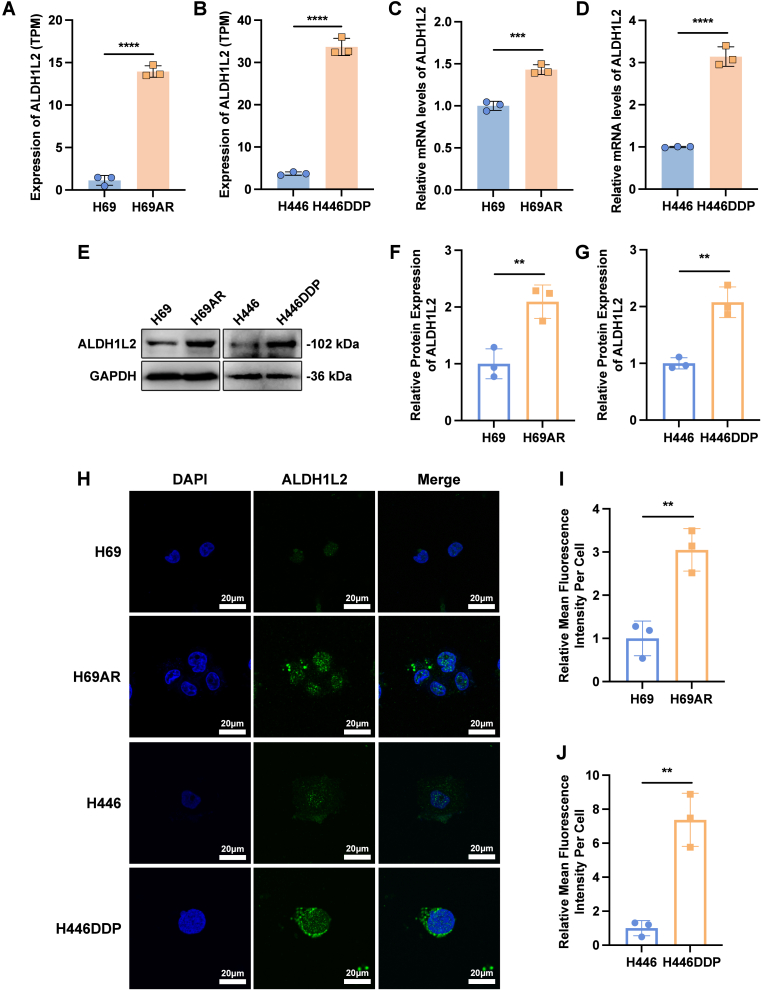
ALDH1L2 is highly expressed in chemoresistant SCLC cells. (A) Bar plot demonstrating the TPM expression values of ALDH1L2 in H69 and H69AR cells. (B) Bar plot demonstrating the TPM expression values of ALDH1L2 in H446 and H446DDP cells. (C) Verification of the mRNA expression of ALDH1L2 in H69 and H69AR cells by RT-qPCR. (D) Verification of the mRNA expression of ALDH1L2 in H446 and H446DDP cells by RT-qPCR. (E-G) Verification of the protein expression of ALDH1L2 in SCLC cell lines by immunoblotting. Bar plots show the results of the semiquantitative analysis of the gray values of the protein bands by ImageJ software. (H-J) Immunofluorescence staining to detect the basal expression of ALDH1L2 in SCLC cell lines. The cell nuclei are labeled with blue fluorescence, and the ALDH1L2 protein is labeled with green fluorescence. Scale bar: 20 μm. Bar plots show the results of the semiquantitative analysis of the average fluorescence intensity by ImageJ software. The data are expressed as mean ± standard deviation (n = 3). ns, not significant; ∗, p < 0.05; ∗∗, p < 0.01; ∗∗∗, p < 0.001; ∗∗∗∗, p < 0.0001.

To clarify the correlation between ALDH1L2 and SCLC chemoresistance, we first performed whole transcriptome sequencing of chemosensitive and chemoresistant SCLC cell lines. The results of differential expression analysis revealed that ALDH1L2 was highly expressed in both chemoresistant H69AR and H446DDP SCLC cells (Fig. 2A-B, Fig. S2A–S2B). The results of quantitative real-time PCR (Fig. 2C-D) and western blotting (Fig. 2E-G) further confirmed that ALDH1L2 expression was significantly increased in chemoresistant SCLC cell lines. Immunofluorescence experiments revealed the same results (Fig. 2H-J), suggesting that ALDH1L2 might be involved in the regulatory process of chemoresistance in SCLC.

**Fig. 3:**
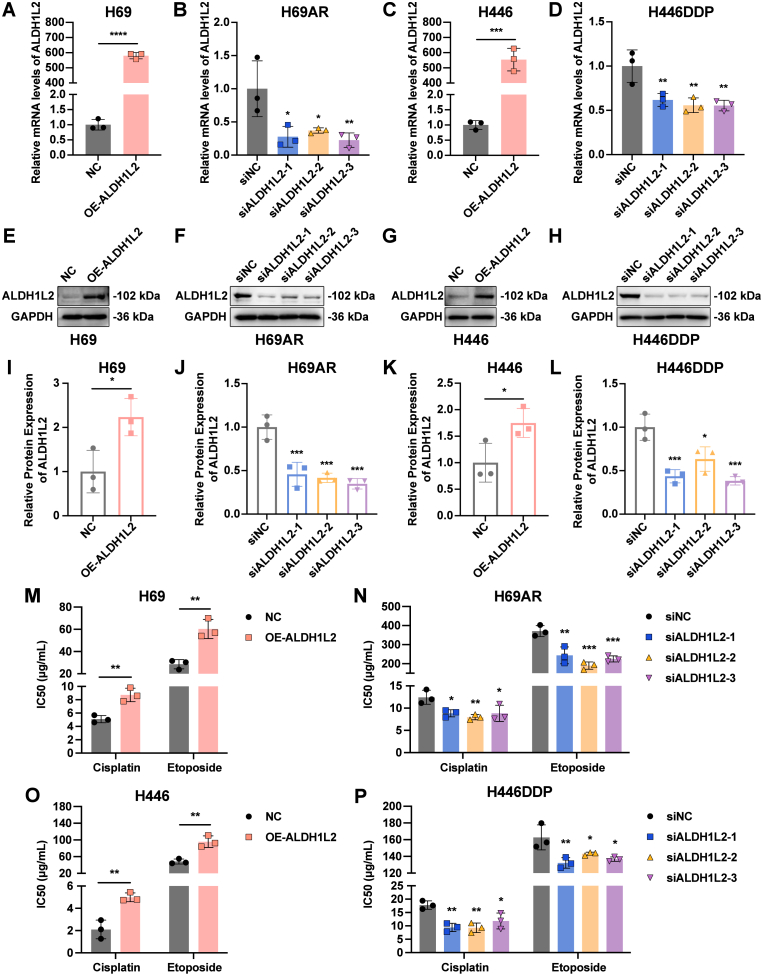
ALDH1L2 positively regulates SCLC chemoresistance in vitro. (A-D) RT-qPCR was used to verify the overexpression and knockdown efficiency of ALDH1L2 in SCLC cells at the mRNA level. (E-L) Immunoblotting was performed to validate the overexpression and knockdown efficiency of ALDH1L2 in SCLC cells at the protein level. Bar plots show the results of the semiquantitative analysis of the gray values of the protein bands by ImageJ software. (M) The results of the CCK-8 assay demonstrated that the IC50 values of cisplatin and etoposide increased in H69 cells after ALDH1L2 was overexpressed. (N) The results of the CCK-8 assay demonstrated that the IC50 values of cisplatin and etoposide decreased in H69AR cells after ALDH1L2 knockdown. (O) The results of the CCK-8 assay demonstrated that the IC50 values of cisplatin and etoposide increased in H446 cells after ALDH1L2 was overexpressed. (P) The results of the CCK-8 assay demonstrated that the IC50 values of cisplatin and etoposide decreased in H446DDP cells after ALDH1L2 knockdown. The data are expressed as mean ± standard deviation (n = 3). ns, not significant; ∗, p < 0.05; ∗∗, p < 0.01; ∗∗∗, p < 0.001; ∗∗∗∗, p < 0.0001.

To further explore the regulatory role of ALDH1L2 in SCLC chemoresistance, we overexpressed ALDH1L2 in chemosensitive cells and knocked down ALDH1L2 in chemoresistant cells for subsequent studies (Fig. 3A-L). We found that overexpression of ALDH1L2 significantly increased the IC50 values of cisplatin and etoposide in H69 and H446 cells (Fig. 3M and O). Similarly, ALDH1L2 knockdown significantly decreased the IC50 values of cisplatin and etoposide in H69AR and H446DDP cells (Fig. 3N and P). These results suggest that ALDH1L2 promotes the resistance of SCLC cells to chemotherapeutic agents.

**Fig. 4:**
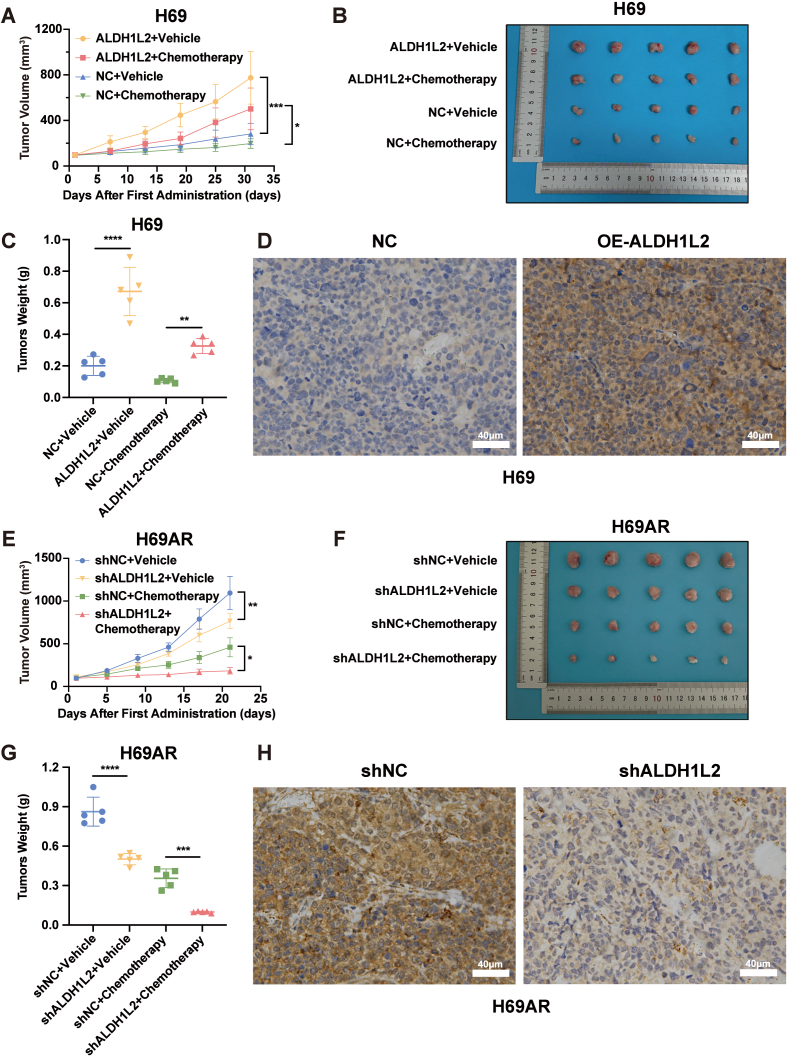
ALDH1L2 positively regulates SCLC chemoresistance in vivo. (A) Growth curves of xenograft tumors derived from ALDH1L2-overexpressing H69 cells and control cells (n = 5). The tumor volume was calculated as follows: (longest diameter) × (shortest diameter)^2^/2. (B) Xenografted tumors derived from ALDH1L2-overexpressing H69 cells and control cells were removed and photographed after the mice were euthanized. (C) Weights of xenograft tumors derived from ALDH1L2-overexpressing H69 cells and control cells are shown. (D) ALDH1L2 expression in tumors derived from ALDH1L2-overexpressing H69 cells and control cells was observed through immunohistochemistry. Scale bar: 40 μm. (E) Growth curves of xenograft tumors derived from ALDH1L2-knockdown H69AR cells and control cells (n = 5). The tumor volume was calculated as follows: (longest diameter) × (shortest diameter)^2^/2. (F) Xenografted tumors derived from ALDH1L2-knockdown H69AR cells and control cells were removed and photographed after the mice were euthanized. (G) Weights of xenograft tumors derived from ALDH1L2-knockdown H69AR cells and control cells are shown. (H) ALDH1L2 expression in tumors derived from ALDH1L2-knockdown H69AR cells and control cells was observed through immunohistochemistry. Scale bar: 40 μm. The data are expressed as mean ± standard deviation. ns, not significant; ∗, p < 0.05; ∗∗, p < 0.01; ∗∗∗, p < 0.001; ∗∗∗∗, p < 0.0001.

To further validate the effect of ALDH1L2 on SCLC chemoresistance in vivo, we established the subcutaneous xenograft tumor model in nude mice using H69 cells stably overexpressing ALDH1L2 and H69AR cells in which ALDH1L2 was stably knocked down. After ALDH1L2 was overexpressed, compared with control tumors, H69 cell-derived tumors displayed significantly accelerated growth and were significantly less sensitive to treatment with cisplatin combined with etoposide (Fig. 4A-C). Similarly, knocking down the expression of ALDH1L2 in H69AR cells suppressed tumor growth and sensitized tumors to chemotherapy in mice (Fig. 4E-G). Finally, the expression of ALDH1L2 in the xenograft tumors was verified by immunohistochemistry (Fig. 4D and H).

The results of the in vitro and in vivo experiments above indicated that ALDH1L2 plays a positive regulatory role in SCLC chemoresistance.

### 3.3 ALDH1L2 promotes SCLC chemoresistance by inhibiting ferroptosis

**Fig. 5:**
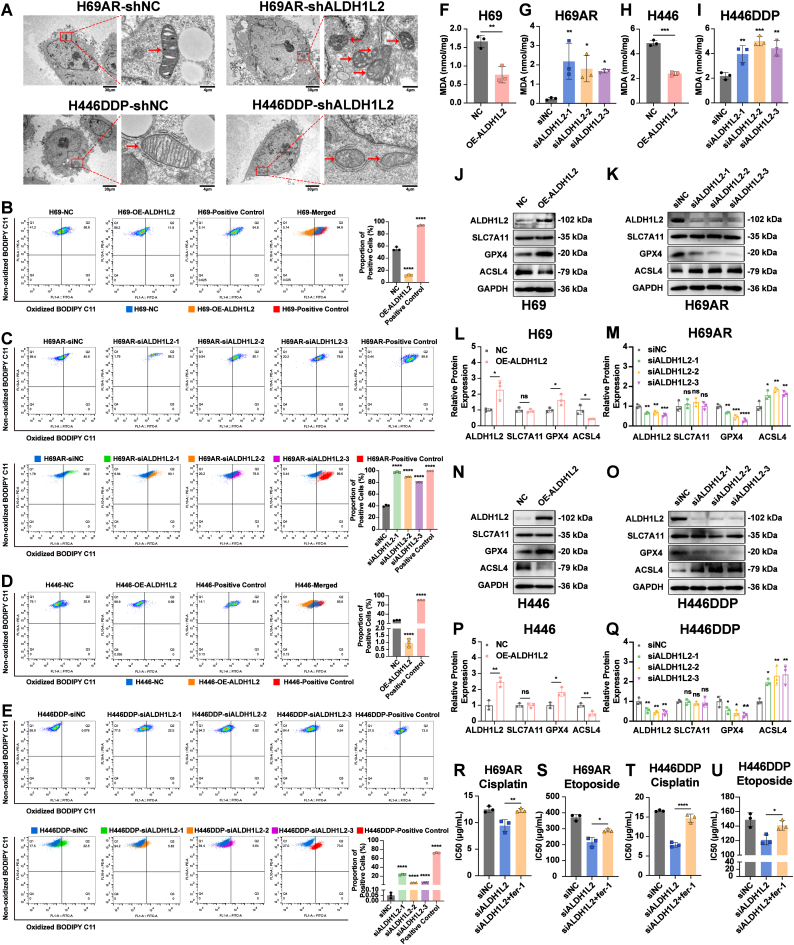
ALDH1L2 inhibits ferroptosis in SCLC cells. (A) After ALDH1L2 knockdown, morphological changes in mitochondria (red arrows) were detected by transmission electron microscopy in H69AR and H446DDP cells. Low-magnification view, scale bar: 30 μm; high-magnification view, scale bar: 4 μm. (B-E) The effects of overexpression and knockdown of ALDH1L2 on the extent of lipid peroxidation in SCLC cells in the presence of cisplatin were detected by flow cytometry. The bar plots show the proportion of FITC/PE-double-positive cells in the upper right quadrant for each group. (F–I) Effect of overexpression and knockdown of ALDH1L2 on the MDA content of SCLC cells in the presence of cisplatin. (J-Q) Changes in ferroptosis marker expression after overexpression or knockdown of ALDH1L2 in SCLC cells detected by immunoblotting. Bar plots show the results of the semiquantitative analysis of the gray values of the protein bands by ImageJ software. (R–U) CCK-8 assay to detect the effect of Fer-1, a ferroptosis inhibitor, on the chemotherapeutic sensitivity of SCLC cells after interference with ALDH1L2 expression. The data are expressed as mean ± standard deviation (n = 3). ns, not significant; ∗, p < 0.05; ∗∗, p < 0.01; ∗∗∗, p < 0.001; ∗∗∗∗, p < 0.0001.

To further explore the specific pathways through which ALDH1L2 promotes chemoresistance in SCLC, we first examined the morphological changes in chemoresistant SCLC cells after knocking down ALDH1L2 expression by transmission electron microscopy. The results revealed that control cells had intact mitochondria and normal mitochondrial cristae, whereas the chemoresistant cells in which ALDH1L2 was knocked down had shrunken mitochondria, a reduction in or disappearance of mitochondrial cristae, and outer mitochondrial membrane rupture (Fig. 5A), indicating that ALDH1L2 knockdown led to an induction of cell death and morphological changes of mitochondria.

Considering mitochondrial alterations induced by ferroptosis, we subsequently assessed the effects of ALDH1L2 on cellular lipid peroxidation levels and the content of malondialdehyde (MDA), which are important indicators of ferroptosis [26,27]. Flow cytometry was used to measure the levels of lipid peroxidation, and the results revealed that in the positive control group (red in merged images), the cell population undergoing lipid peroxidation was present in the upper right quadrant after cumene hydroperoxide treatment (Fig. 5B-E). After ALDH1L2 was overexpressed in H69 and H446 cells, the bulk of the cell populations were in the upper left quadrant, and the proportion of FITC/PE-double-positive cells decreased in the presence of cisplatin, indicating that the degree of lipid peroxidation was reduced in chemosensitive cells after overexpression of ALDH1L2 compared with that in control cells (Fig. 5B and D). Similarly, the proportions of both H69AR and H446DDP cells in the upper right quadrant increased after ALDH1L2 was downregulated in the presence of cisplatin, suggesting that the degree of lipid peroxidation in chemoresistant cells significantly increased after ALDH1L2 knockdown (Fig. 5C and E). MDA is an important byproducts of lipid peroxidation. We found that the MDA content in H69 and H446 cells overexpressing ALDH1L2 was significantly lower than that in the control group under cisplatin treatment (Fig. 5F and H). In contrast, after ALDH1L2 was knocked down, MDA levels significantly increased in H69AR and H446DDP cells (Fig. 5G and I). These results indicated that ALDH1L2 inhibited lipid peroxidation reactions in SCLC cells.

Solute carrier family 7 member 11 (SLC7A11), glutathione peroxidase 4 (GPX4), and long-chain acyl-coenzyme A synthase 4 (ACSL4) are landmark proteins of ferroptosis, and their expression may be closely connected with ferroptosis [10,[27], [28], [29], [30], [31]]. Decreased expression of SLC7A11 and GPX4, as well as increased expression of ACSL4, may threaten redox imbalance and lead to ferroptosis [10,[27], [28], [29], [30], [31]]. Immunoblotting experiments revealed no significant change in the expression of SLC7A11, whereas the expression of GPX4 increased and the expression of ACSL4 decreased after ALDH1L2 was overexpressed in H69 (Fig. 5J and L) and H446 cells (Fig. 5N and P). In contrast, after the expression of ALDH1L2 was knocked down in H69AR (Fig. 5K and M) and H446DDP cells (Fig. 5O and Q), the expression of SLC7A11 did not significantly change, but the expression of GPX4 decreased and the expression of ACSL4 increased, suggesting that the SCLC cells were under increased oxidative stress, which contributes to the occurrence of ferroptosis.

To further confirm the critical role of ferroptosis in the regulation of SCLC chemoresistance by ALDH1L2, we performed rescue experiments of cytotoxicity induced by ferrostatin-1 (Fer-1), a synthetic selective ferroptosis inhibitor that inhibits lipid peroxidation and prevents damage to membrane lipids [11,32]. In chemoresistant H69AR and H446DDP cells, Fer-1 treatment (2 μM) reversed the effect of ALDH1L2 downregulation on chemoresistance to cisplatin and etoposide (Fig. 5R-U). These results suggest that ALDH1L2 promotes SCLC chemoresistance by regulating ferroptosis.

### 3.4 Enzymatic activity of ALDH1L2 operates to suppress ferroptosis and foster chemoresistance in SCLC

**Fig. 6:**
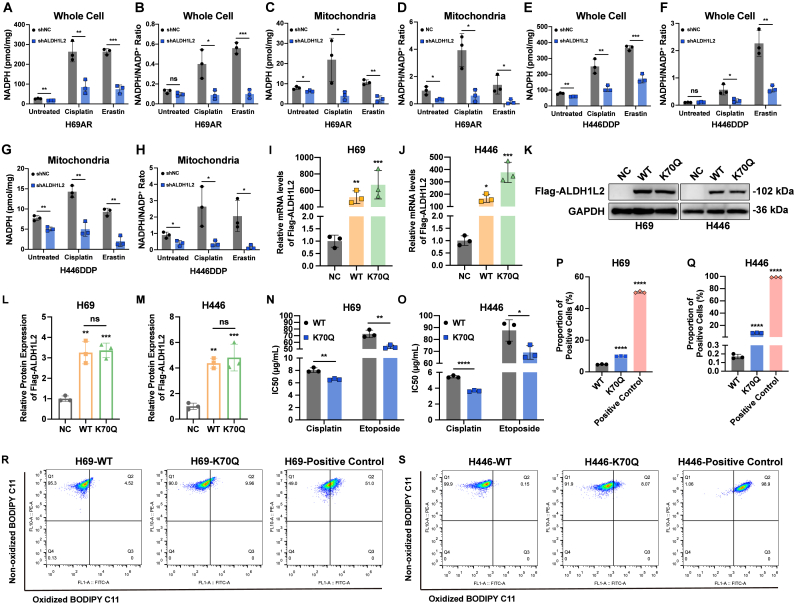
Enzymatic activity of ALDH1L2 participates in ferroptosis suppression and chemoresistance facilitation within SCLC cells. (A-B) Effect of ALDH1L2 knockdown on whole-cell NADPH level (A) and NADPH/NADP^+^ ratio (B) in H69AR cells. (C-D) Effect of ALDH1L2 knockdown on mitochondrial NADPH level (C) and NADPH/NADP^+^ ratio (D) in H69AR cells. (E-F) Effect of ALDH1L2 knockdown on whole-cell NADPH level (E) and NADPH/NADP^+^ ratio (F) in H446DDP cells. (G-H) Effect of ALDH1L2 knockdown on mitochondrial NADPH level (G) and NADPH/NADP^+^ ratio (H) in H446DDP cells. (I-J) RT-qPCR was used to verify the overexpression efficiency of Flag-ALDH1L2 (WT and K70Q) in H69 (I) and H446 (J) cells at the mRNA level. (K-M) Immunoblotting was performed to validate the overexpression efficiency of Flag-ALDH1L2 (WT and K70Q) in H69 and H446 cells at the protein level. Bar plots show the results of the semiquantitative analysis of the gray values of the protein bands by ImageJ software. (N–O) CCK-8 assay showed the IC50 difference between ALDH1L2-WT and ALDH1L2-K70Q cells under cisplatin and etoposide. (P–S) The difference in the proportion of lipid-peroxidized cells between ALDH1L2-WT and ALDH1L2-K70Q populations in the presence of cisplatin were detected by flow cytometry. The bar plots show the proportion of FITC/PE-double-positive cells in the upper right quadrant for each group. The data are expressed as mean ± standard deviation (n = 3). ns, not significant; ∗, p < 0.05; ∗∗, p < 0.01; ∗∗∗, p < 0.001; ∗∗∗∗, p < 0.0001.

As the mitochondrial 10-formyltetrahydrofolate dehydrogenase, ALDH1L2 converts 10-formyl-THF to THF and CO_2_, with concomitant reduction of NADP^+^ to NADPH [[13], [14], [15]]. We therefore first ascertained the impact of ALDH1L2 on NADPH homeostasis. Under basal conditions, ALDH1L2 knockdown produced only a modest reduction in whole-cell NADPH levels in chemoresistant SCLC cells (Fig. 6A-B, Fig. 6E-F). In contrast, when cells were treated with cisplatin or erastin, ALDH1L2 knockdown precipitated a pronounced decline in both whole-cell NADPH levels and the NADPH/NADP^+^ ratios (Fig. 6A-B, Fig. 6E-F). To further interrogate ALDH1L2-mediated control of NADPH homeostasis at the subcellular level, we isolated mitochondria for NADPH quantification and NADPH/NADP^+^ ratio determination. ALDH1L2 knockdown precipitated a marked drop in mitochondrial NADPH levels and the NADPH/NADP^+^ ratio; this decline was further amplified by cisplatin or erastin exposure, indicating that ALDH1L2 buffers drug-induced oxidative stress by producing mitochondrial reducing equivalents (Fig. 6C-D, Fig. 6G-H).

After confirming ALDH1L2's role in maintaining cellular NADPH homeostasis, we next examined whether its enzymatic activity modulates SCLC biology. Guided by published studies, we generated ALDH1L2-K70Q (catalytically impaired) and wild-type (WT) plasmids [17], both of which were stably overexpressed in chemosensitive SCLC cells (Fig. 6I-M). Immunoblotting confirmed equivalent exogenous ALDH1L2 abundance in ALDH1L2-WT and ALDH1L2-K70Q cells (Fig. 6K-M).

We next evaluated whether attenuated ALDH1L2 activity alters SCLC chemosensitivity. Compared with ALDH1L2-WT cells, ALDH1L2-K70Q mutants exhibited a significant reduction in IC50 values for both cisplatin and etoposide (Fig. 6N-O). Consistently, cisplatin-treated ALDH1L2-K70Q cells displayed a significantly higher proportion of lipid-peroxidized cells than their WT counterparts (Fig. 6P-S). These data indicate that enzymatic activity of ALDH1L2 contributes to its suppression of ferroptosis and the consequent promotion of chemoresistance in SCLC.

### 3.5 ALDH1L2 promoted SCLC chemoresistance by reducing the level of hyperoxidized PRDX3 and oxidized PRDX3 dimer on the plasma membrane

ALDH1L2 is localized mainly in mitochondria, where it plays its catalytic role and produces NADPH [[13], [14], [15]]. Approximately 90% of reactive oxygen species (ROS) in eukaryotic cells are generated in mitochondria and are cleared mainly by PRDX3 [[33], [34], [35], [36]]. Reduced PRDX3 can be transformed into oxidized PRDX3 homodimers [37]. The reduction of inactive PRDX3 dimers relies on the participation of TRX2 and NADPH [20,38]. After being reduced by NADPH, TRX2 restores PRDX3 activity by reacting with the disulfide bond in the PRDX3 dimer through the sulfhydryl (-SH) group at its active site [19,20,38]. Therefore, we hypothesized that ALDH1L2 might interact with the TRX2-PRDX3 signaling axis. We simulated this interaction by molecular docking analysis.

**Fig. 7:**
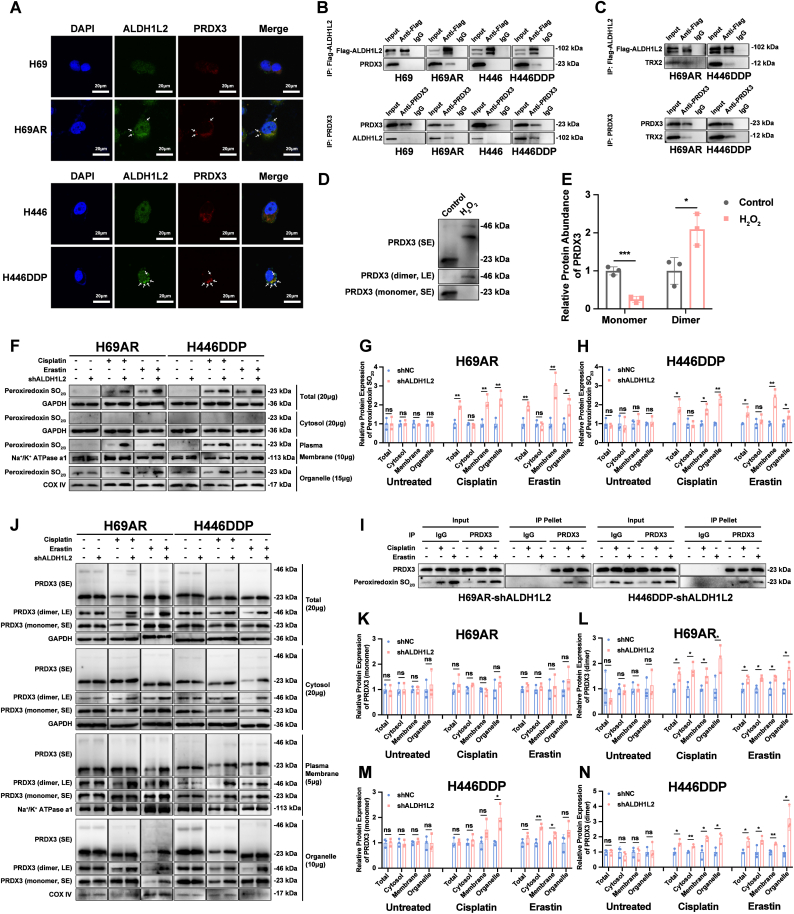
ALDH1L2 promotes SCLC chemoresistance by negatively regulating the hyperoxidized PRDX3 and PRDX3 dimer content in the plasma membrane. (A) Immunofluorescence detection of the colocalization of ALDH1L2 and PRDX3 in SCLC cells. The cell nuclei are labeled with blue fluorescence, the ALDH1L2 protein is labeled with green fluorescence, and the PRDX3 protein is labeled with red fluorescence. Scale bar: 20 μm. (B) Coimmunoprecipitation assays revealed the interaction of the ALDH1L2 protein with the PRDX3 protein in chemoresistant SCLC cells. (C) Coimmunoprecipitation assays revealed the interaction of the TRX2 protein with the ALDH1L2 protein and the PRDX3 protein in chemoresistant SCLC cells. (D-E) The reaction of PRDX3 with hydroperoxides was analyzed by immunoblotting. Bar plots show the results of the semiquantitative analysis of the gray values of the protein bands by ImageJ software. (F–H) Analysis of the effect of ALDH1L2 knockdown on the content of hyperoxidized PRDXs protein in each fraction of H69AR and H446DDP cells. The results under no treatment, cisplatin treatment (10 μg/mL for 24 h) and erastin treatment (20 μM for 72 h) are shown in the graph. Bar plots show the results of the semiquantitative analysis of the gray values of the protein bands by ImageJ software. (I) Coimmunoprecipitation assays revealed that hyperoxidized PRDXs protein could be immunoprecipitated by anti-PRDX3 antibody. (J-N) Analysis of the effect of ALDH1L2 knockdown on the content of oxidized PRDX3 dimers in each fraction of H69AR and H446DDP cells. The results under no treatment, cisplatin treatment (10 μg/mL for 24 h) and erastin treatment (20 μM for 72 h) are shown in the graph. LE, long exposure; SE, short exposure. Bar plots show the results of the semiquantitative analysis of the gray values of the protein bands by ImageJ software. The data are expressed as mean ± standard deviation (n = 3). ns, not significant; ∗, p < 0.05; ∗∗, p < 0.01; ∗∗∗, p < 0.001; ∗∗∗∗, p < 0.0001.

The protein structure of ALDH1L2 predicted by AlphaFold 3 was used for molecular docking analysis (Fig. S3A). The binding energies of the ALDH1L2 protein with the PRDX3 protein and TRX2 protein were all less than −10 kcal/mol (Fig. S3B–S3C), which indicated a strong connection. We found that the binding energy of the ALDH1L2-PRDX3 complex was lower than that of the ALDH1L2-TRX2 complex, suggesting that the binding of ALDH1L2 to PRDX3 was more stable (Fig. S3B–S3C). Therefore, we performed immunofluorescence colocalization experiments. We observed extensive colocalization of ALDH1L2 and PRDX3 in H69AR and H446DDP cells, whereas no evident colocalization of both proteins was observed in H69 and H446 cells (Fig. 7A). To confirm this interaction, we carried out coimmunoprecipitation experiments, and the results further confirmed the interaction of ALDH1L2 and PRDX3 in H69AR and H446DDP cells but not in H69 and H446 cells (Fig. 7B). In addition, we confirmed that both ALDH1L2 and PRDX3 interact with TRX2 in H69AR and H446DDP cells (Fig. 7C).

Recent studies have shown that hyperoxidized PRDX3 is a hallmark of ferroptosis [39]. PRDX3 utilizes its own Cys residue to reduce lipid peroxides in mitochondria, and oxidative reactions result in the formation of disulfide-linked PRDX3 homodimers [40,41]. Using purified PRDX3 protein, we recapitulated this transition: after 1 min of H_2_O_2_ exposure, PRDX3 shifted to the dimeric species with concomitant loss of the reduced monomers (Fig. 7D-E). In the presence of excess lipid peroxides, PRDX3 undergoes hyperoxidation and is translocated from the mitochondria to the plasma membrane, where it inhibits cystine uptake and thereby induces ferroptosis [39].

Therefore, we hypothesized that the binding of ALDH1L2 and PRDX3 may influence the formation of hyperoxidized PRDX3 and oxidized PRDX3 dimers, which further affect the occurrence of ferroptosis and SCLC chemoresistance. To verify this hypothesis, we first visualized the subcellular distribution of PRDX3 by high-resolution immunofluorescence. As shown in Fig. S3D–S3E, PRDX3 clearly co-localized with Na^+^/K^+^ ATPase a1 at the cell periphery in ALDH1L2-knockdown chemoresistant cells after treatment with either cisplatin or erastin (Fig. S3D–S3E).

Next, we performed subcellular fractionation of chemoresistant SCLC cells, and total cellular proteins were separated into cytosolic proteins, plasma membrane proteins, and organelle proteins by differential and density centrifugation. Proper separation of subcellular fractions was tested by the immunoblotting of marker proteins characteristic of the respective fractions (Fig. S3F–S3G).

To interrogate the oxidative status of PRDX3, we performed immunoblotting to detect hyperoxidized PRDX3 and PRDX3 homodimers with an anti-Peroxiredoxin-SO_2_/SO_3_ antibody and an anti-PRDX3 antibody, respectively. Anti-Peroxiredoxin-SO_2_/SO_3_ antibody can specifically recognizes a peptide containing Cys-SO_2/3_H present in PRDXs [39]. Erastin, a ferroptosis inducer, was used as the positive control. Previous studies have demonstrated that erastin treatment elicits the production of hyperoxidized PRDX3 [39]. Our data demonstrated that hyperoxidized PRDXs protein markedly increased in ALDH1L2-knockdown chemoresistant cells upon either cisplatin or erastin challenge. This elevation was detected in both the plasma membrane and organelle fractions, whereas no appreciable signal was observed in the cytosolic fraction (Fig. 7F-H). Co-immunoprecipitation verified that these hyperoxidized PRDXs were indeed hyperoxidized PRDX3, because they could be successfully immunoprecipitated by the anti-PRDX3 antibody (Fig. 7I). Finally, under the same conditions, the content of PRDX3 dimers was significantly greater in the plasma membrane and organelle fractions of ALDH1L2-knockdown cells than that in control cells (Fig. 7J–N), corroborating that oxidized PRDX3 produced in mitochondria are transferred to the plasma membrane, creating a favorable situation for ferroptosis.

**Fig. 8:**
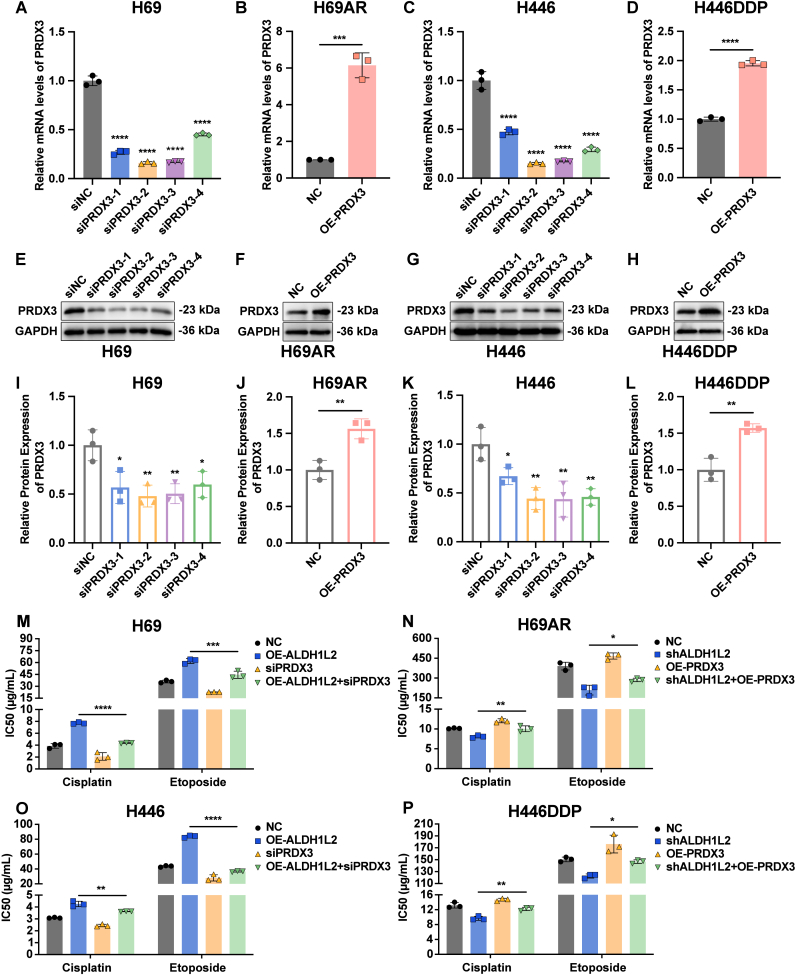
PRDX3 contributes to the ALDH1L2-mediated chemoresistance program in SCLC. (A-D) RT-qPCR was used to verify the knockdown and overexpression efficiency of PRDX3 in SCLC cells at the mRNA level. (E-L) Immunoblotting was performed to validate the knockdown and overexpression efficiency of PRDX3 in SCLC cells at the protein level. Bar plots show the results of the semiquantitative analysis of the gray values of the protein bands by ImageJ software. (M − P) CCK-8 assay to determine the role of PRDX3 in the regulation of chemoresistance elicited by ALDH1L2 in SCLC cells. IC50, half maximal inhibitory concentration. The data are expressed as mean ± standard deviation (n = 3). ns, not significant; ∗, p < 0.05; ∗∗, p < 0.01; ∗∗∗, p < 0.001; ∗∗∗∗, p < 0.0001.

To further confirm the critical role of PRDX3 in the effect of ALDH1L2 on SCLC chemoresistance, we simultaneously interfered with the expression of ALDH1L2 and PRDX3 and tested the sensitivity of SCLC cells to cisplatin and etoposide. First, we detected the efficiency of PRDX3 overexpression and knockdown, and siPRDX3-2, whose knockdown efficiency was the highest, was selected for subsequent rescue experiments (Fig. 8A-L). Consistent with the previous results, ALDH1L2 overexpression increased the tolerance to cisplatin and etoposide in H69 and H446 cells, whereas PRDX3 knockdown partially restored the sensitivity of H69 and H446 cells to cisplatin and etoposide (Fig. 8M and O). Similarly, ALDH1L2 knockdown sensitized H69AR and H446DDP cells to cisplatin and etoposide, whereas PRDX3 overexpression partially restored the resistance of H69AR and H446DDP cells to cisplatin and etoposide (Fig. 8N and P). These results suggested that the ability of ALDH1L2 to promote SCLC chemoresistance was, at least in part, dependent on PRDX3.

In summary, ALDH1L2 could negatively regulate the levels of hyperoxidized PRDX3 and PRDX3 dimers in the plasma membrane, which in turn promoted SCLC chemoresistance.

### 3.6 The PRDX3 inhibitor thiostrepton helps overcome SCLC chemoresistance

The above data revealed that ALDH1L2 negatively regulates the formation of hyperoxidized PRDX3 and PRDX3 dimers to promote SCLC chemoresistance. Therefore, we investigated the potential of the PRDX3 inhibitor thiostrepton in overcoming SCLC chemoresistance. Thiostrepton has been reported to form covalent bonds with catalytic cysteine residues of PRDX3, leading to the inactivation of PRDX3, which increases ROS generation in mitochondria and leads to tumor cell death [20,42,43].

**Fig. 9:**
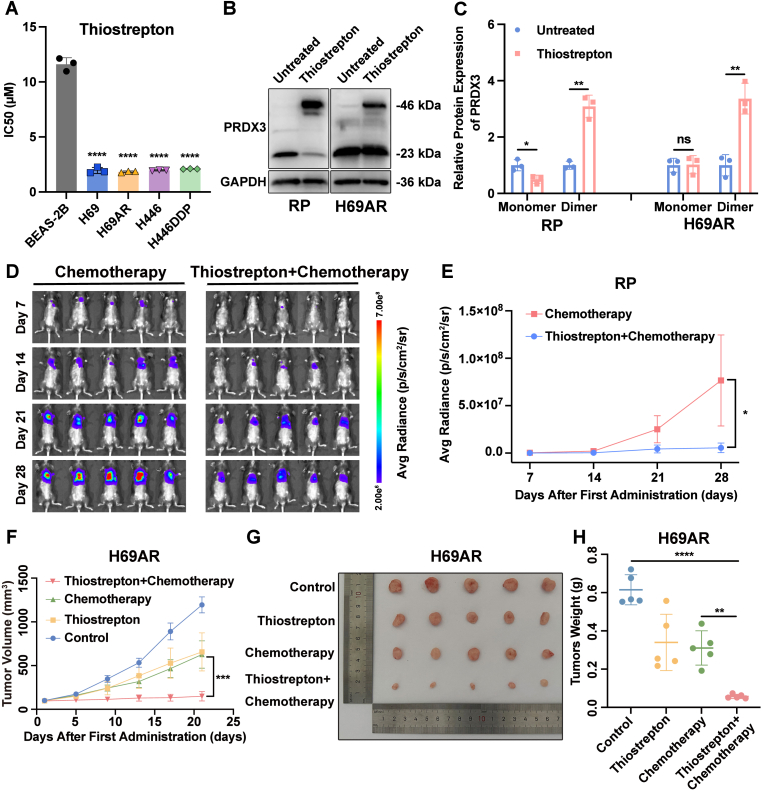
The PRDX3 inhibitor thiostrepton synergizes with chemotherapy to suppress tumor growth in SCLC. (A) CCK-8 assay to determine the IC50 values of thiostrepton in human normal bronchial epithelial BEAS-2B cells and SCLC cells (n = 3). (B–C) Verification of the inhibitory effect of thiostrepton on PRDX3 in SCLC cells by immunoblotting. Bar plots show the results of the semiquantitative analysis of the gray values of the protein bands by ImageJ software (n = 3). (D) The growth of tumors in the orthotopic SCLC model was recorded with the live animal imaging system (n = 5). (E) Line chart demonstrating the quantitative bioluminescence intensities for each group. (F) Growth curves of xenografted tumors derived from H69AR cells under different treatments are shown (n = 5). The tumor volume was calculated as follows: (longest diameter) × (shortest diameter)^2^/2. (G) Xenografted tumors derived from H69AR cells under different treatments were removed and photographed after the mice were euthanized. (H) Weights of xenografted tumors derived from H69AR cells under different treatments are shown. IC50, half maximal inhibitory concentration. The data are expressed as mean ± standard deviation. ns, not significant; ∗, p < 0.05; ∗∗, p < 0.01; ∗∗∗, p < 0.001; ∗∗∗∗, p < 0.0001.

Given the considerable therapeutic value of thiostrepton for tumor treatment, we evaluated the IC50 of thiostrepton in human normal bronchial epithelial BEAS-2B cells and SCLC cells to validate its tumor specificity and safety. The data revealed that the IC50 of thiostrepton for BEAS-2B cells was 11.61 μM, which was much greater than that for SCLC cell lines (Fig. 9A). Thiostrepton was more cytotoxic to SCLC cells than to normal bronchial epithelial cells, making it an interesting candidate for new therapeutics for SCLC.

Next, we applied thiostrepton to mouse-derived RP cells and human-derived H69AR cells. The results revealed that inactive PRDX3 dimers were significantly increased in RP and H69AR cells after thiostrepton treatment (Fig. 9B-C). In addition, the intensity of the monomeric band of PRDX3 was significantly reduced in RP cells in the presence of thiostrepton (Fig. 9B-C). These results suggest that thiostrepton inhibits the activity of PRDX3 in SCLC cells.

We next constructed the orthotopic SCLC model in C57BL/6 mice using the RP cell line. According to live imaging, compared with that in the chemotherapy group, tumor growth in the group of combination of thiostrepton and chemotherapy was significantly inhibited (Fig. 9D-E), suggesting that the application of thiostrepton enhanced the cytotoxic effect of chemotherapeutic agents on SCLC cells.

We further constructed the subcutaneous xenograft tumor model in nude mice using human-derived H69AR cells. The results showed that the combination therapy of chemotherapy and thiostrepton achieved optimal tumor suppression. Compared with that in the thiostrepton group and the chemotherapy group, the rate of tumor growth was significantly decreased in the combination-treated group (Fig. 9F-H).

These results suggested that thiostrepton, a PRDX3 inhibitor, significantly enhanced the cytotoxic effects of cisplatin and etoposide on SCLC cells.

## 4 Discussion

Small cell lung cancer is a subtype of lung cancer known for its high aggressiveness [3,4,44]. The tumor grows rapidly and is prone to early metastasis, demonstrating complex and variable biological features [3,4,44]. Chemotherapy with etoposide and platinum remains a standard approach for the first-line treatment of SCLC patients [[3], [4], [5],^44^]. However, despite the high initial remission rate, SCLC patients often experience rapid progression of chemoresistance, leading to tumor recurrence and poor prognosis [[3], [4], [5],^44^]. Therefore, how to overcome SCLC chemoresistance has become an urgent problem for clinicians and researchers.

The heterogeneity of SCLC results in complex chemoresistance mechanisms, including differential gene expression, cell cycle regulation, metabolic reprogramming, epigenetic regulation, and the tumor microenvironment [3,44]. Metabolic reprogramming is among the important characteristics of tumors, and targeting key metabolic pathways can effectively inhibit tumor growth and provide new ideas for overcoming therapeutic resistance. Statins targeting the mevalonate-geranylgeranyl diphosphate pathway can significantly inhibit the growth of chemoresistant SCLC tumors [45]. In addition, the de novo purine biosynthesis pathway is significantly activated in SCLC cells with low ASCL1 expression, and inhibition of inosine monophosphate dehydrogenase expression suppresses the growth and proliferation of SCLC cells with low ASCL1 expression [46]. Therefore, in this study, we first focused on changes in metabolic pathways between chemosensitive and chemoresistant SCLC cells. Through bioinformatics analysis, we confirmed the key role of water-soluble vitamins and cofactors metabolism pathway in promoting SCLC chemoresistance and further revealed that ALDH1L2 could positively regulate SCLC chemoresistance.

ALDH1L2 is highly expressed in a variety of tumor cells [17,47,48]. Several studies have shown that targeting ALDH1L2 can alter the responsiveness of tumors to therapy. In colorectal cancer, the acetylation of ALDH1L2 inhibits its enzymatic activity and reduces the production of NADPH and GSH, which increases the sensitivity of tumor cells to 5-fluorouracil [17]. Another study revealed that colorectal cancer patients with low ALDH1L2 expression are resistant to radiotherapy [18]. These findings suggest that ALDH1L2 plays different roles in different biological situations. In this study, we verified the positive regulatory role of ALDH1L2 in SCLC chemoresistance through in vitro and in vivo experiments.

Mitochondria are the main site where ALDH1L2 exerts its catalytic effect, and these organelles undergo the most significant morphological changes during ferroptosis [[9], [10], [11],[13], [14], [15]]. The main source of cellular ROS is the oxidative phosphorylation process in mitochondria, and excessive ROS can lead to oxidative stress and oxidative damage to lipids, proteins, and DNA [27]. Therefore, a complete antioxidant system is established in the mitochondria to buffer the ROS generated by the electron transport chain, and this antioxidant defense line is composed of the glutathione system, thioredoxin system, peroxiredoxin family, superoxide dismutase and nonenzymatic antioxidant systems [[49], [50], [51], [52], [53], [54]]. The antioxidant functions of the glutathione system, thioredoxin system, and peroxiredoxin family are highly dependent on NADPH, an important source of intracellular reducing equivalents and one of the key products of the ALDH1L2-catalyzed reaction [38,55]. Therefore, abnormal expression or defective function of ALDH1L2 affects the redox balance of mitochondria. In the present study, transmission electron microscopy revealed that the mitochondria of chemoresistant SCLC cells after ALDH1L2 knockdown exhibited morphological changes. Under cisplatin treatment, ALDH1L2 knockdown resulted in increased lipid peroxidation and MDA content, downregulation of GPX4 expression, and upregulation of ACSL4 expression in chemoresistant SCLC cells. Fer-1, a ferroptosis inhibitor, could rescue the effect of ALDH1L2 knockdown on chemoresistance in SCLC cells. Thus, in our study, the specific cellular sublocalization and function of ALDH1L2 linked SCLC chemoresistance and ferroptosis.

Recent studies have shown that hyperoxidized PRDX3 can be used as a marker of ferroptosis [39]. PRDX3 is a mitochondria-specific peroxidase that scavenges up to 90% of H_2_O_2_ and lipid peroxides in mitochondria [35,36]. The antioxidant effect of PRDX3 results in the formation of the inactive form of PRDX3 as a homodimer [37]. Hyperoxidized PRDX3 can translocate from the mitochondria to the cell membrane and inhibit cystine uptake, thereby inducing ferroptosis [39]. The reduction of the oxidized PRDX3 dimer relies mainly on TRX2 and NADPH to reengage in the catalytic cycle in mitochondria [20,38]. In the present study, we confirmed the interaction between ALDH1L2 and PRDX3 in chemoresistant SCLC cells. In addition, we verified that both ALDH1L2 and PRDX3 interact with TRX2. Notably, ALDH1L2 knockdown in chemoresistant SCLC cells resulted in an increase in the levels of hyperoxidized PRDX3 and PRDX3 dimers in the presence of cisplatin, and this difference was significant in the plasma membrane fractions. The results of the rescue experiments suggested that PRDX3 is a key molecule through which ALDH1L2 regulates SCLC chemoresistance.

The combination of immunotherapy with chemotherapy has been applied as first-line therapy for extensive-stage small cell lung cancer, yet durable responses remain exceptional [1,3,4]. Non-NE SCLC cells exhibit high expression of ACSL4 and LPCAT3, along with high levels of ether lipids containing polyunsaturated fatty acids (PUFAs), resulting in high sensitivity to ferroptosis [12]. NE SCLC cells are dependent on the TRX pathway and are more sensitive to TRX pathway inhibitor [12]. The combination of ferroptosis inducer and TRX pathway inhibitor could effectively overcome the heterogeneity of SCLC cell subtypes and significantly inhibit tumor growth [12]. This study fully demonstrates the feasibility and potential value of targeting ferroptosis in combination therapy for SCLC. In this study, combining the PRDX3 inhibitor thiostrepton with chemotherapy significantly inhibited the growth of SCLC tumors. Thiostrepton, a polypeptide antibiotic produced by the Actinomyces genus, initially attracted attention because of its antimicrobial activity [56]. In recent years, thiostrepton has demonstrated antitumor activity in a variety of tumors [[57], [58], [59], [60], [61]]. Thiostrepton sensitizes tumor cells to chemotherapy and exhibits synergistic effects when combined with cisplatin and paclitaxel in platinum-resistant ovarian cancer [59]. Additionally, thiostrepton treatment markedly increased intratumoral CD3^+^ T cell infiltration and demonstrated synergistic antitumor efficacy when combined with anti-4-1BB antibody in immune-resistant lung tumors [62]. Thiostrepton also significantly suppressed regulatory T cell differentiation in MC38 tumor model [63]. These findings indicate that thiostrepton reprograms the tumor microenvironment toward an immunostimulatory state, thereby facilitating its integration with both cytotoxic chemotherapy and immune checkpoint blockade. Concurrently, the combination permits dose-reduction of cisplatin, mitigating platinum-associated toxicities without compromising therapeutic efficacy [64]. Building on our finding that ALDH1L2 maintains mitochondrial NADPH to restrain PRDX3 hyperoxidation and ferroptosis, prospectively quantifying ALDH1L2 and PRDX3 protein levels can stratify patients for thiostrepton-chemotherapy combinations, aligning therapy with tumour redox biology and advancing precision medicine in SCLC.

Compared with other PRDX isoforms, thiostrepton displays a marked selectivity for PRDX3 and preferentially reacts with this protein in cells [43]. Thiostrepton inhibits the activity of PRDX3 by covalently binding to it [20,42,43]. Notably, the oxidized PRDX3 dimeric structure is more responsive to thiostrepton [43]. However, thiostrepton additionally exerts pleiotropic effects, including FOXM1 interaction [65], proteasome inhibition [66], and ribosome binding [67]. The development of small-molecule inhibitors that selectively target PRDX3 represents a crucial direction for future research [68].

Thiostrepton has been the subject of a clinical trial in the United Kingdom aimed at evaluating its efficacy in patients with malignant pleural effusion due to advanced/metastatic solid tumors, including mesothelioma (ClinicalTrials. gov ID: NCT05278975). To date, no peer-reviewed study has reported the human intravenous pharmacokinetics, pharmacodynamics, dose-exposure relationship, dose-limiting toxicity, or bioavailability of thiostrepton; the only available data come from murine studies, which have not revealed significant alterations in blood biochemistry or histological changes in vital organs [62]. Multiple studies have shown that thiostrepton selectively impairs tumor cell survival without affecting normal tissue cells, rendering it a promising antineoplastic agent [62,[69], [70], [71]]. Despite its promising antitumor activity, the clinical translation of thiostrepton is hindered by poor aqueous solubility and drug delivery [72], which may account for the current absence of active clinical trials employing intravenous thiostrepton regimens. The development of nanomedicine and hemisynthetic analogues that exhibit improved chemical stability, solubility, and tumor selectivity relative to the parent compound is poised to unlock the clinical potential of thiostrepton in oncology [68,[72], [73], [74], [75]].

## 5 Conclusions

Through bioinformatics analysis, we found that ALDH1L2-associated water-soluble vitamins and cofactors metabolic pathway was positively correlated with cisplatin resistance in SCLC. High expression of ALDH1L2 was a poor prognostic factor for SCLC patients. We subsequently verified that ALDH1L2 promoted SCLC chemoresistance via in vitro and in vivo experiments. Mechanistically, ALDH1L2 negatively regulates cellular lipid peroxidation levels and inhibits ferroptosis. In chemoresistant SCLC cells, ALDH1L2 interacts with the TRX2-PRDX3 antioxidant network to reduce the content of hyperoxidized PRDX3 and oxidized PRDX3 dimers in the plasma membrane in the presence of cisplatin, protecting cells from ferroptosis and promoting chemoresistance. In addition, we demonstrated that thiostrepton, a PRDX3 inhibitor, enhanced the killing effect of chemotherapeutic agents on SCLC cells, providing a new combination strategy for overcoming SCLC chemoresistance.

## Ethics statement

All animal experiments were conducted with the approval of the Ethics Committee for Animal Experimentation of Zhujiang Hospital of Southern Medical University.

## CRediT authorship contribution statement

**Yueming Zhang:** Conceptualization, Data curation, Formal analysis, Investigation, Methodology, Validation, Visualization, Writing – original draft. **Ruibin Yi:** Formal analysis, Investigation. **Xinyi Zhou:** Investigation. **Qiong Lyu:** Investigation. **Huiying Liu:** Investigation. **Yaru Zhu:** Investigation. **Peng Luo:** Conceptualization, Methodology, Writing – review & editing. **Weitao Shen:** Conceptualization, Funding acquisition, Methodology, Writing – review & editing. **Jian Zhang:** Conceptualization, Funding acquisition, Supervision, Writing – review & editing.

## Declaration of competing interest

The authors declare that they have no known competing financial interests or personal relationships that could have appeared to influence the work reported in this paper.

## Data Availability

Data will be made available on request.

## Appendix A Supplementary data

Fig. S1 Water-soluble vitamins and cofactors metabolic pathway positively regulates SCLC chemoresistance, corresponding to Fig. 1. (A) Heatmap showing the AUC values of 98 metabolic models in the training set and testing set, and the left bar shows the arithmetic average AUC value of each metabolic model in the training set and testing set. (B–D) Kaplan-Meier survival curves for overall survival in the George cohort (B), TU-SCLC cohort (C), and local cohort (D). The yellow curves represent SCLC patients with higher ALDH1L2 expression, and the blue curves represent SCLC patients with lower ALDH1L2 expression. Fig. S1

Fig. S2 ALDH1L2 is highly expressed in chemoresistant SCLC cells, corresponding to Fig. 2 (A-B) Volcano plots showing the genes that are differentially expressed between chemosensitive and chemoresistant SCLC cells, with the blue dots representing genes highly expressed in chemosensitive cells (p < 0.05, log_2_FC < −2) and the orange dots representing genes highly expressed in chemoresistant cells (p < 0.05, log_2_FC > 2). FDR, false discovery rate; FC, fold change. Fig. S2

Fig. S3 ALDH1L2 promotes SCLC chemoresistance by negatively regulating the hyperoxidized PRDX3 and PRDX3 dimer content in the plasma membrane, corresponding to Fig. 7. (A) The 3D structure prediction of the ALDH1L2 protein was obtained from the AlphaFold database. (B–C) Molecular docking was used to simulate the interaction of the ALDH1L2 protein with the PRDX3 protein (B) and the TRX2 protein (C). The blue color represents the ALDH1L2 protein, the green color represents the PRDX3 protein, and the gray color represents the TRX2 protein. (D-E) Immunofluorescence detection of the colocalization of PRDX3 and Na^+^/K^+^ ATPase a1 in H69AR (D) and H446DDP (E) cells. The cell nuclei are labeled with blue fluorescence, the Na^+^/K^+^ ATPase a1 protein is labeled with green fluorescence, and the PRDX3 protein is labeled with red fluorescence. Scale bar: 20 μm. (F-G) Total cell lysates of H69AR (F) and H446DDP (G) cells were separated into to cytosolic, plasma membrane and organelle fractions (containing mitochondria) using the protein isolation and cell fractionation kit (Invent Biotechnologies, catalog number: SM-005). Immunoblotting was used to verify successful cell fractionation. GAPDH was used as an internal reference protein for the total and cytosolic fractions, and Na^+^/K^+^ ATPase a1 was used as an internal reference protein for the membrane fraction. PRDX3 is a mitochondrial protein; therefore, COX IV was used as an internal reference protein for the organelle fraction. Fig. S3
