# Supplementary figures and images for: ALDH1L2 induces resistance to chemotherapy in small cell lung cancer by inhibiting ferroptosis
Source: Redox Biol. 2026 Feb 23;91:104098. doi: 10.1016/j.redox.2026.104098 (PMC12966749; doi:10.1016/j.redox.2026.104098)

A

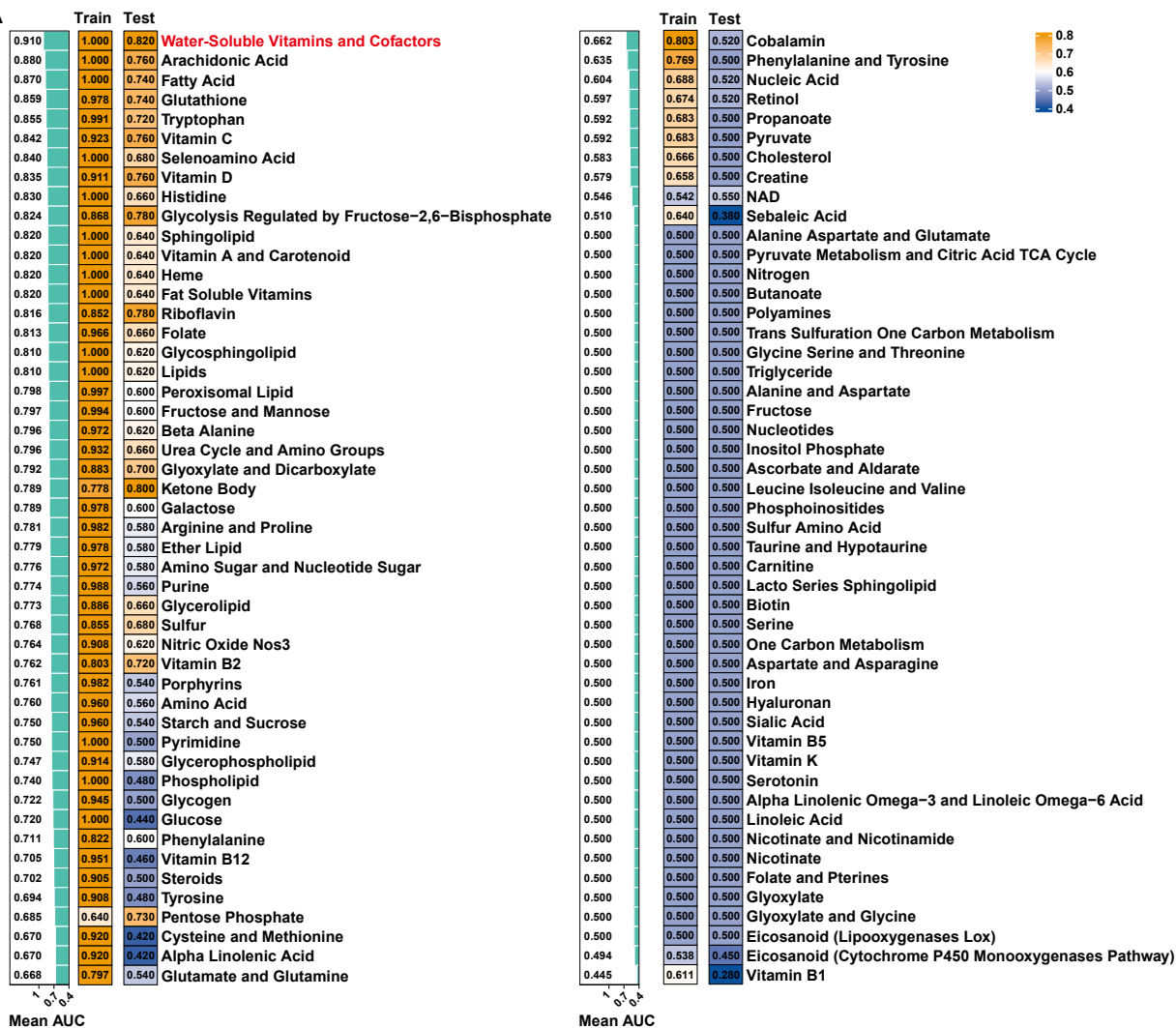

Mean AUC

Mean AUC

B

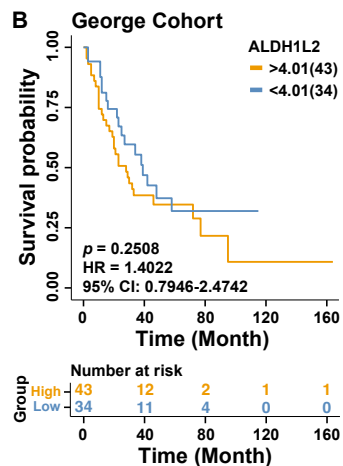

C

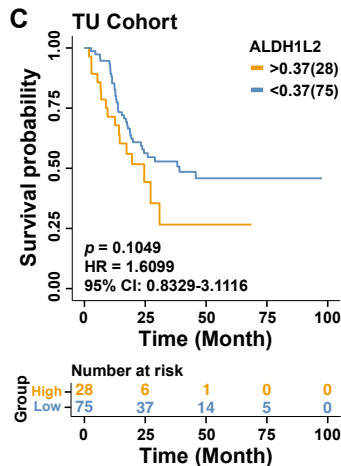

D

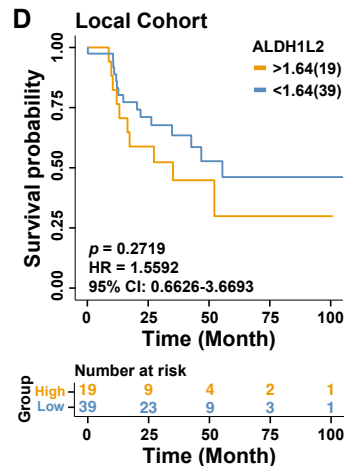

Supplement: Fig. S1 — Water-soluble vitamins and cofactors metabolic pathway positively regulates SCLC chemoresistance, corresponding to Fig. 1. (A) Heatmap showing the AUC values of 98 metabolic models in the training set and testing set, and the left bar shows the arithmetic average AUC value of each metabolic model in the training set and testing set. (B–D) Kaplan-Meier survival curves for overall survival in the George cohort (B), TU-SCLC cohort (C), and local cohort (D). The yellow curves represent SCLC patients with higher ALDH1L2 expression, and the blue curves represent SCLC patients with lower ALDH1L2 expression. [file mmc1.pdf]

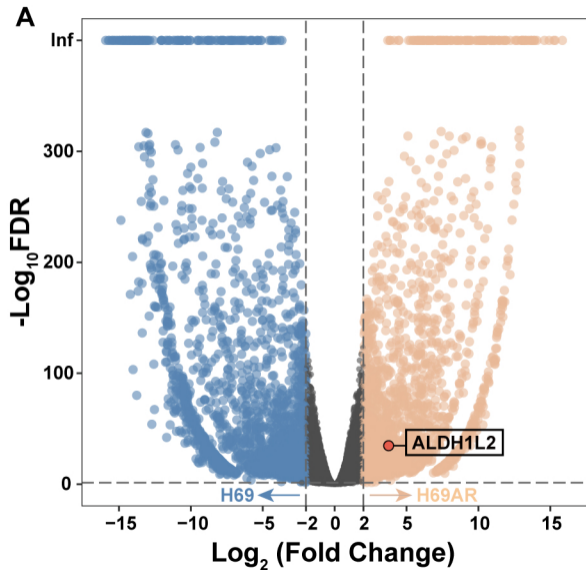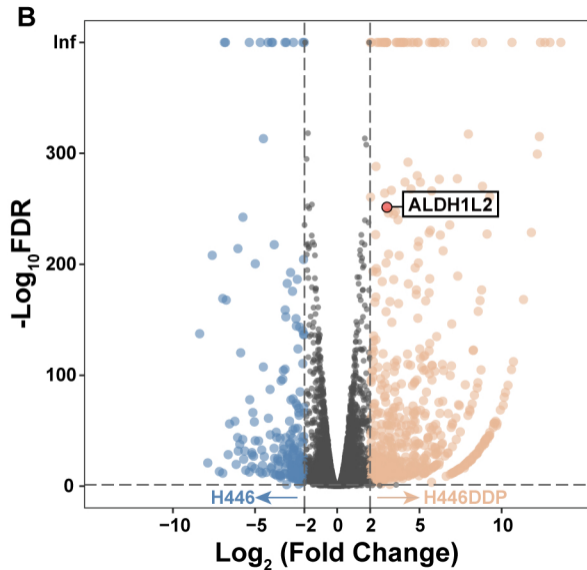

Supplement: Fig. S2 — ALDH1L2 is highly expressed in chemoresistant SCLC cells, corresponding to Fig. 2 (A-B) Volcano plots showing the genes that are differentially expressed between chemosensitive and chemoresistant SCLC cells, with the blue dots representing genes highly expressed in chemosensitive cells (p < 0.05, log2FC < −2) and the orange dots representing genes highly expressed in chemoresistant cells (p < 0.05, log2FC > 2). FDR, false discovery rate; FC, fold change. [file mmc2.pdf]
