# Supplementary material for: ALDH1L2 induces resistance to chemotherapy in small cell lung cancer by inhibiting ferroptosis
Source: Redox Biol. 2026 Feb 23;91:104098. doi: 10.1016/j.redox.2026.104098 (PMC12966749; doi:10.1016/j.redox.2026.104098)

Raw Data

Fig. 2E First Repetition

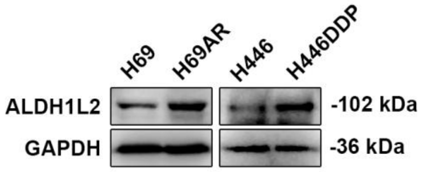

H69 vs. H69AR

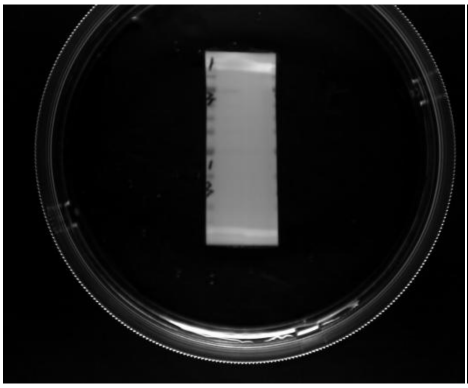
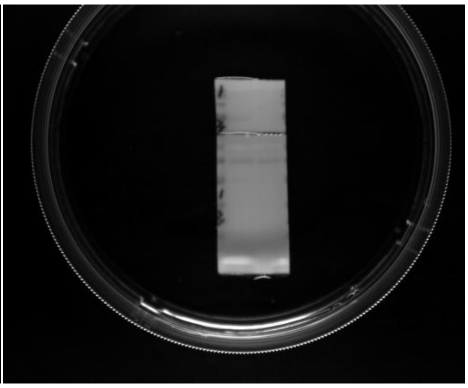

ALDH1L2

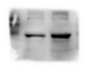
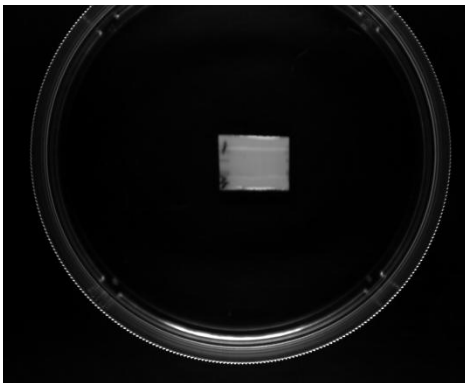

GAPDH

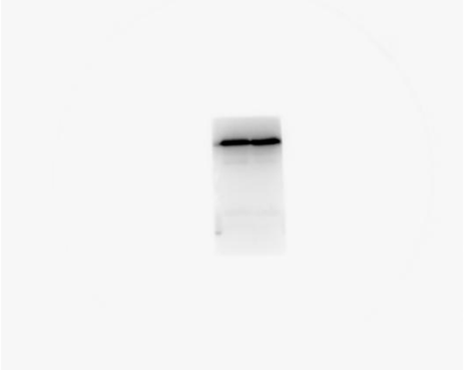
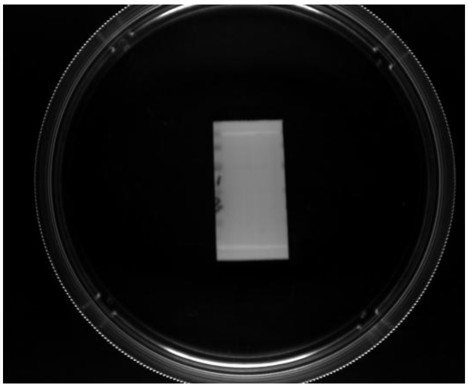

H446 vs. H446DDP

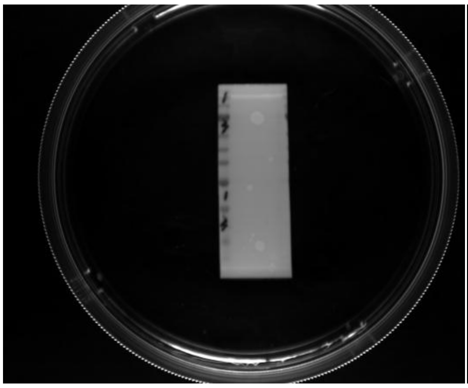

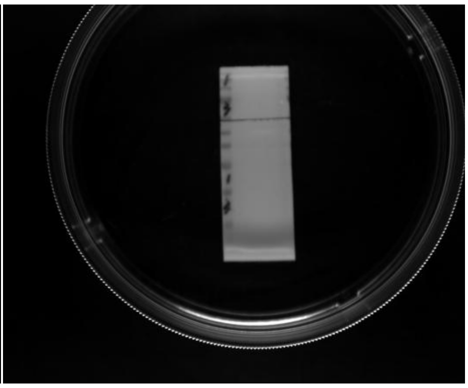

ALDH1L2

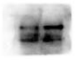

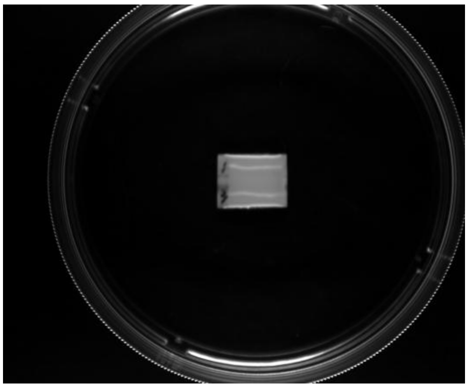

GAPDH

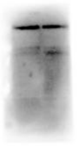

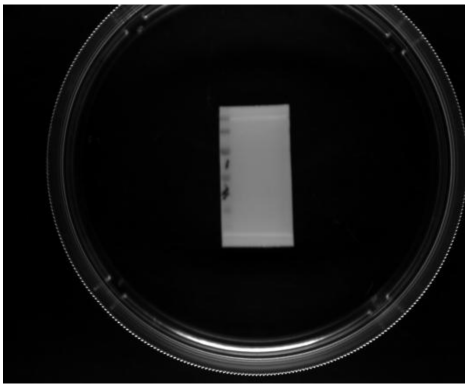

Fig. 2E Second Repetition

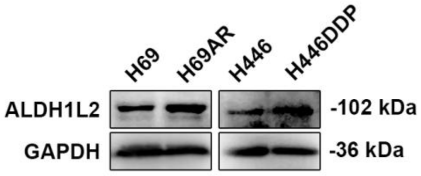

H69 vs. H69AR

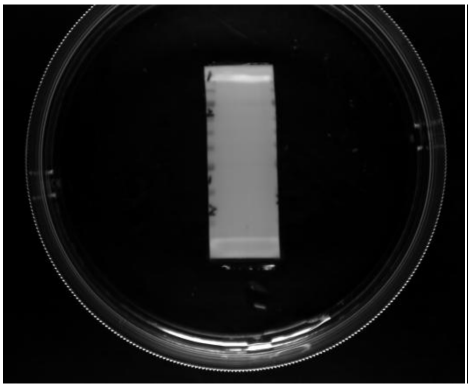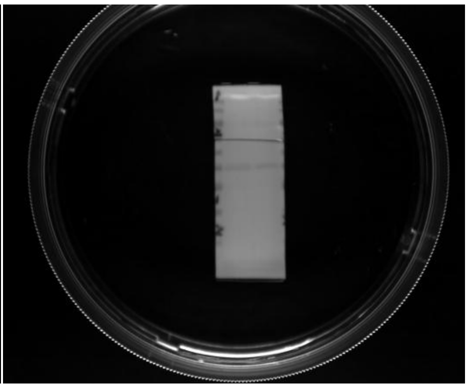

ALDH1L2

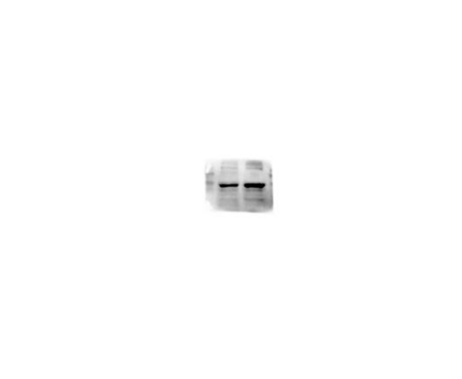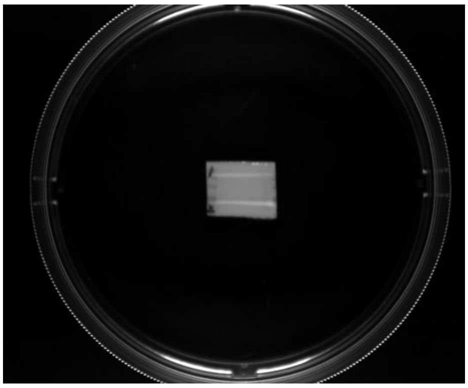

GAPDH

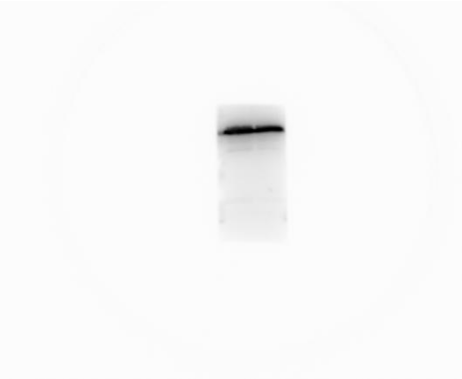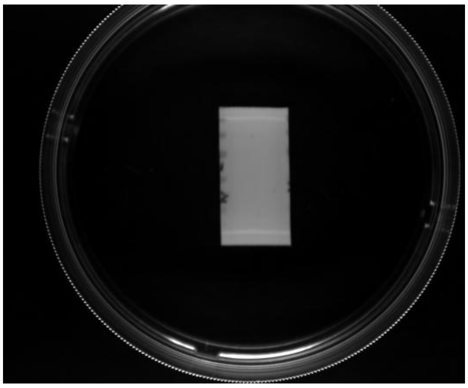

H446 vs. H446DDP

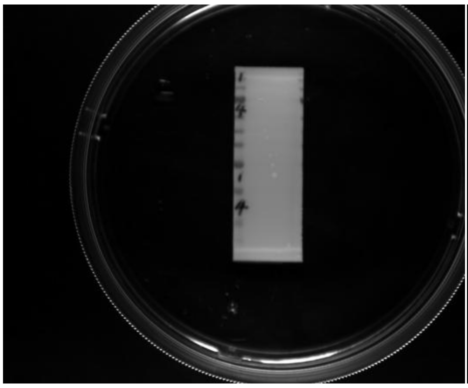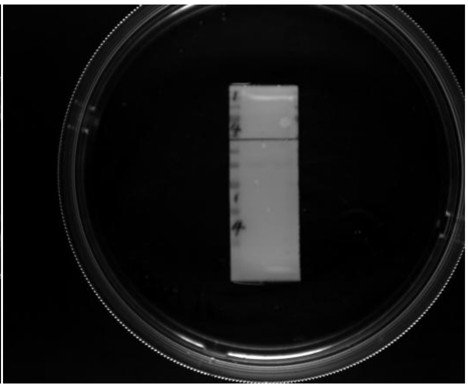

ALDH1L2

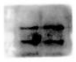

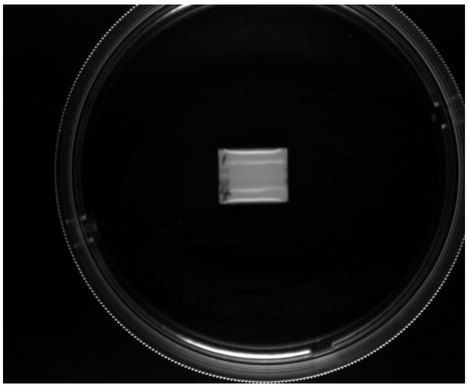

GAPDH

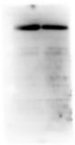

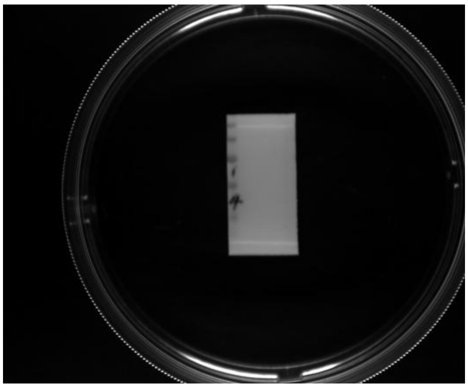

Fig. 2E Third Repetition

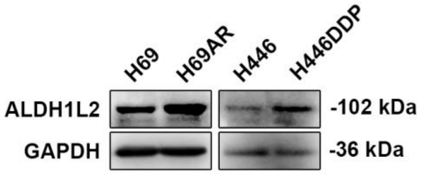

H69 vs. H69AR

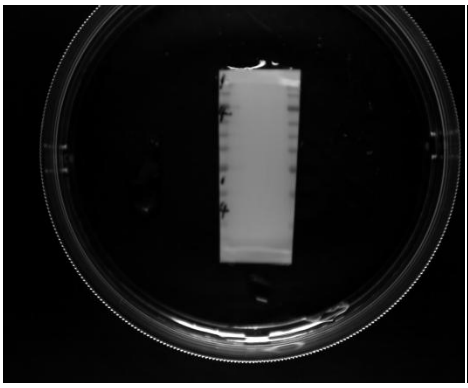

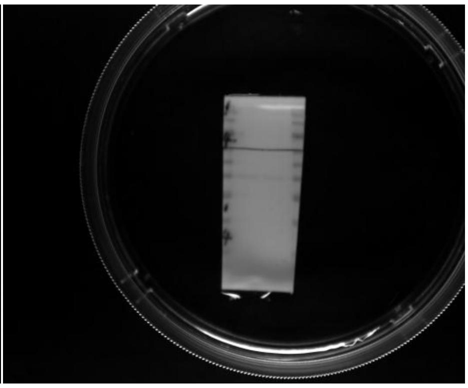

ALDH1L2

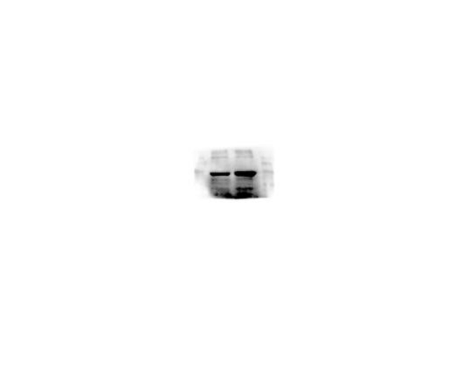

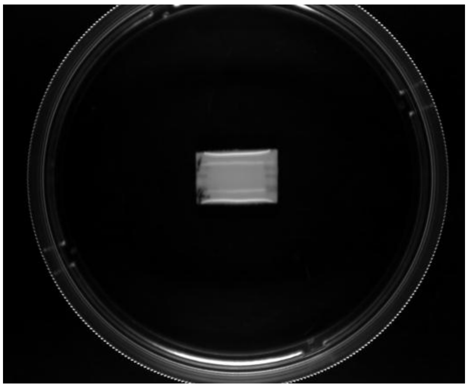

GAPDH

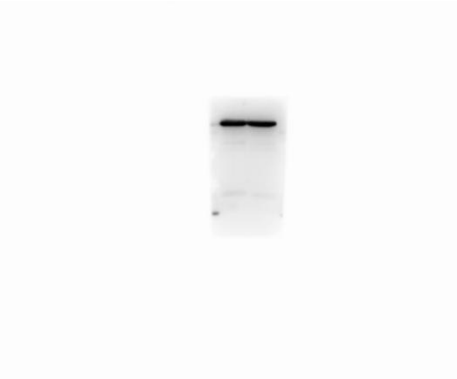

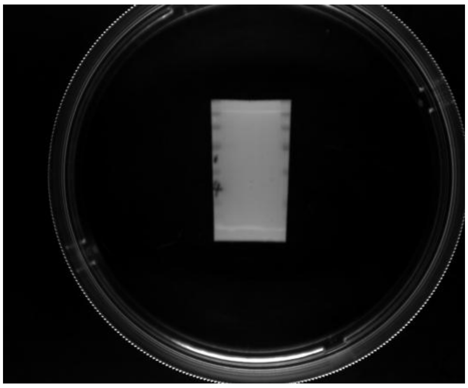

H446 vs. H446DDP

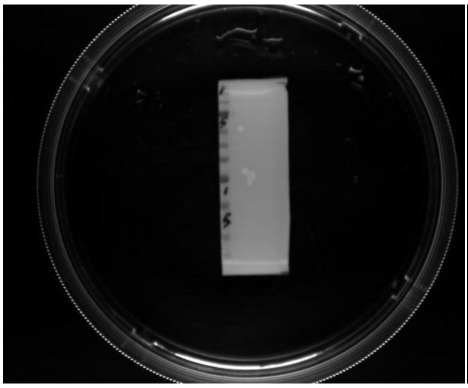

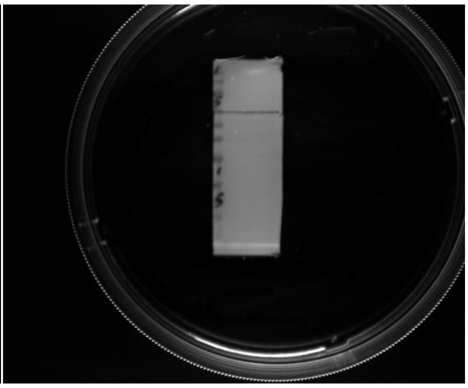

ALDH1L2

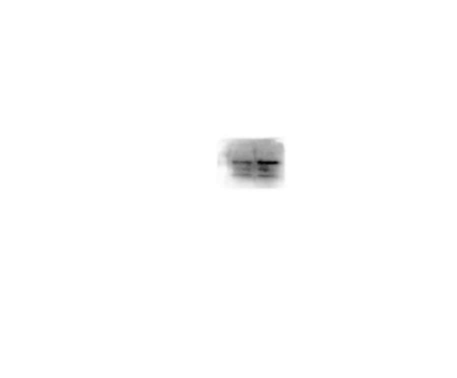

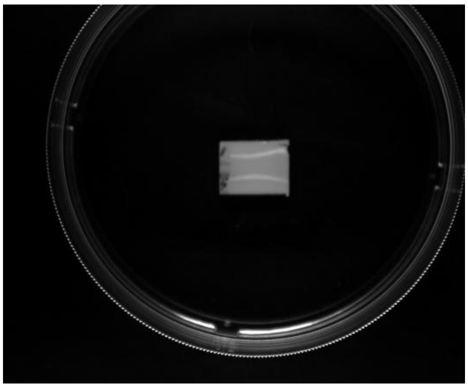

GAPDH

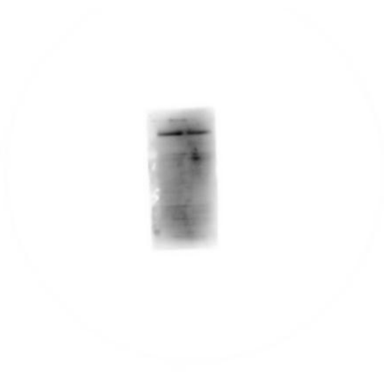

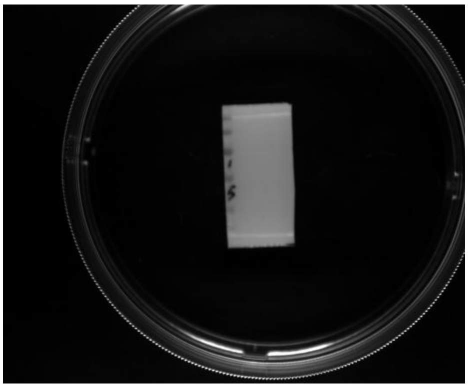

Fig. 2H First Repetition

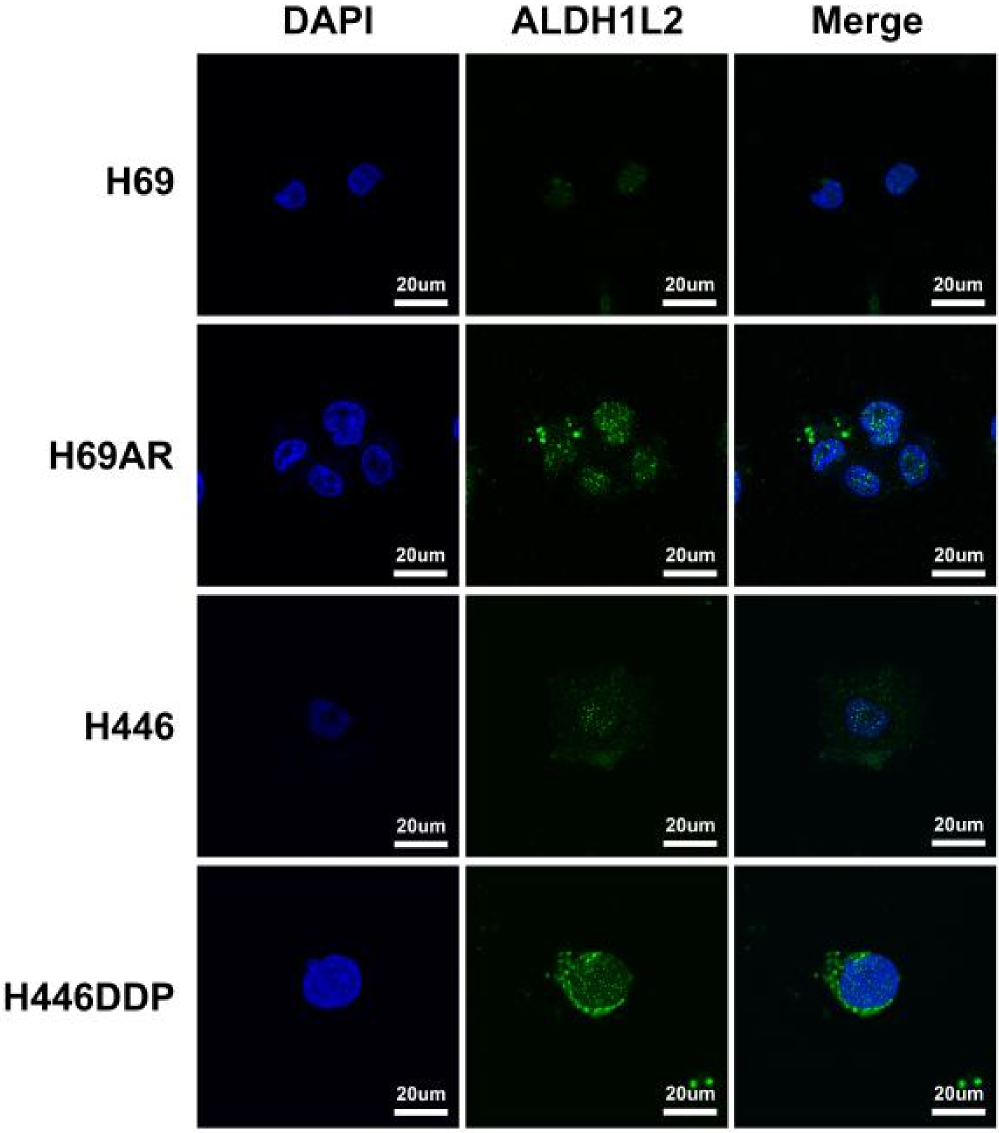

Fig. 2H Second Repetition

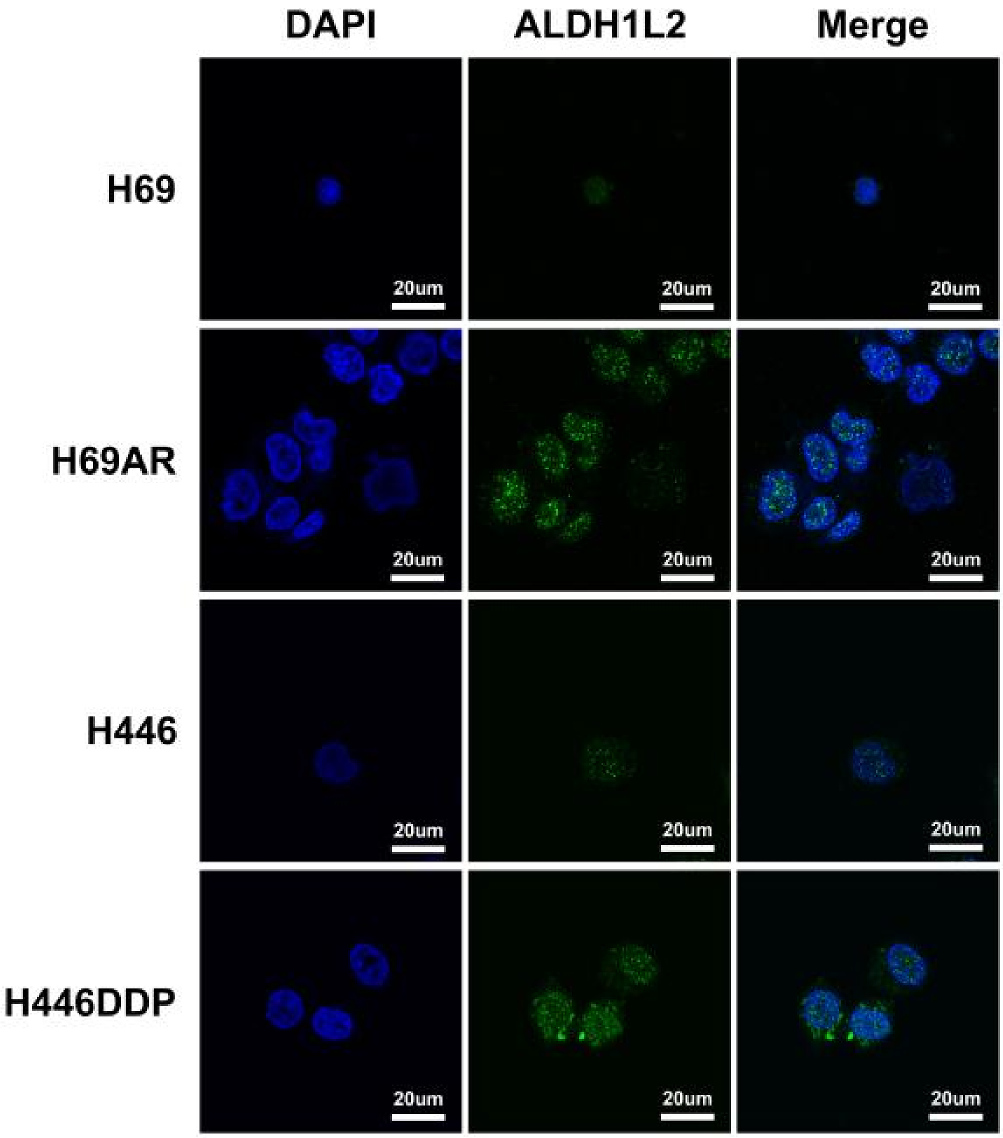

Fig. 2H Third Repetition

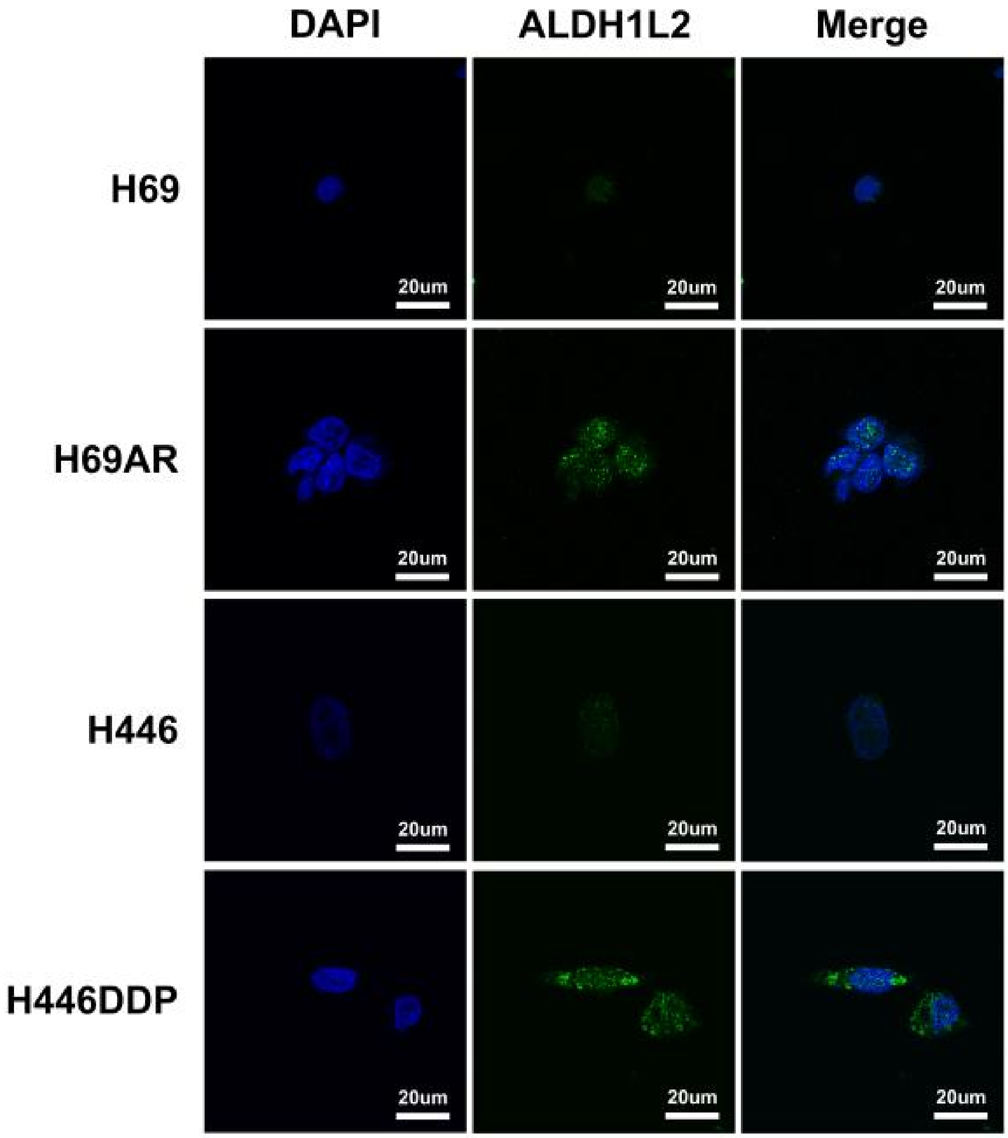

Fig. 3E First Repetition

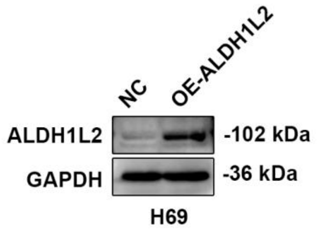

H69

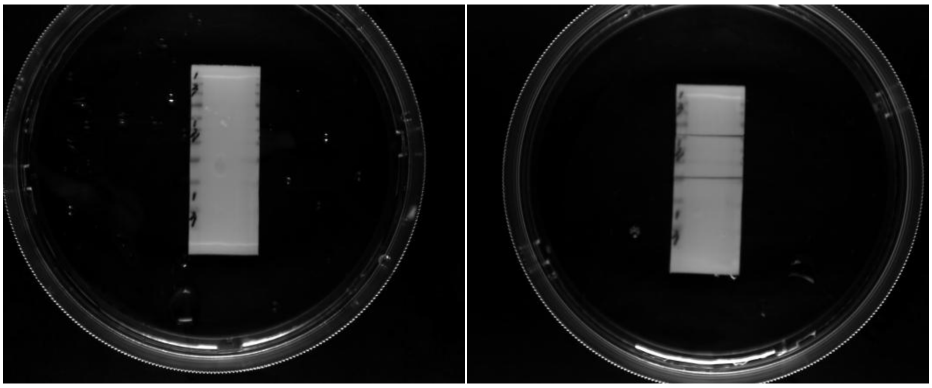

ALDH1L2

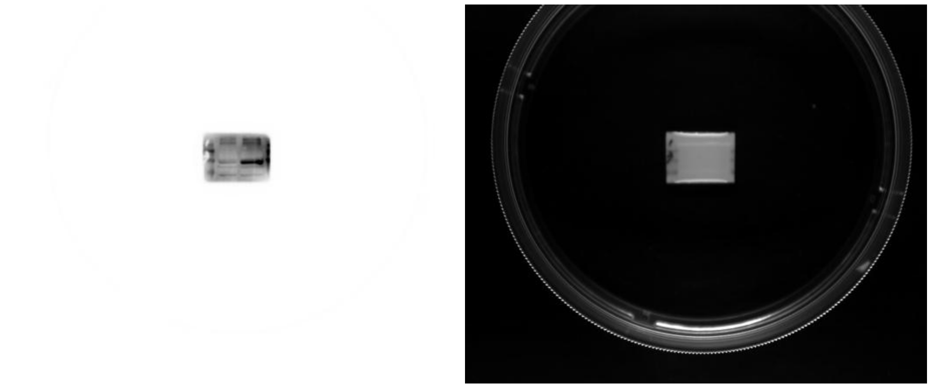

GAPDH

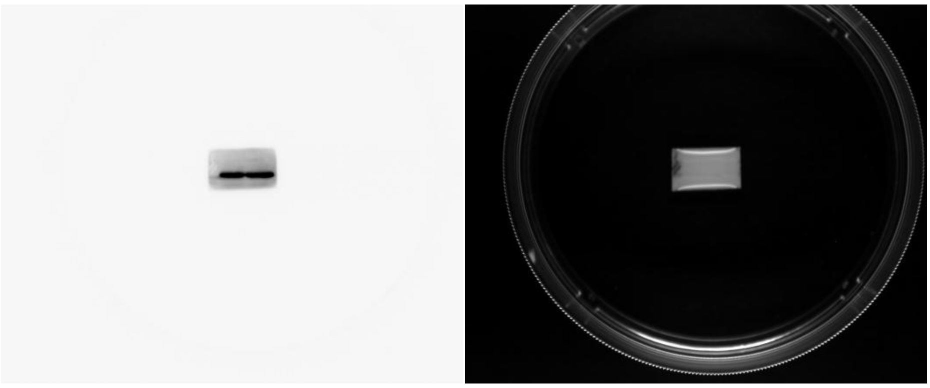

Fig. 3E Second Repetition

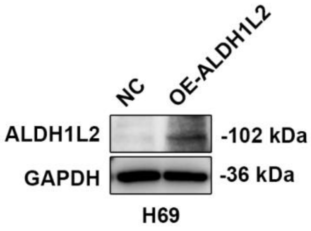

H69

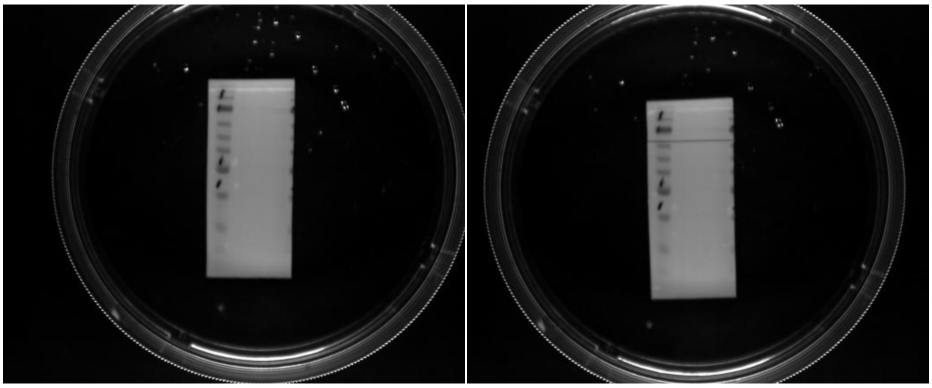

ALDH1L2

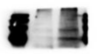

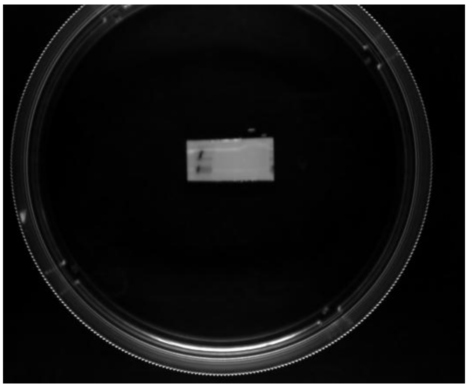

GAPDH

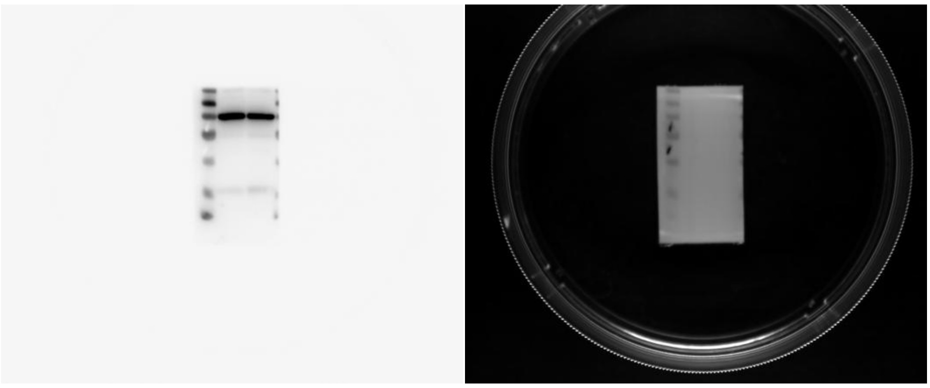

Fig. 3E Third Repetition

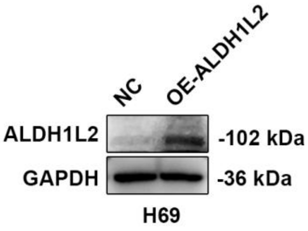

H69

ALDH1L2

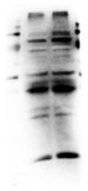

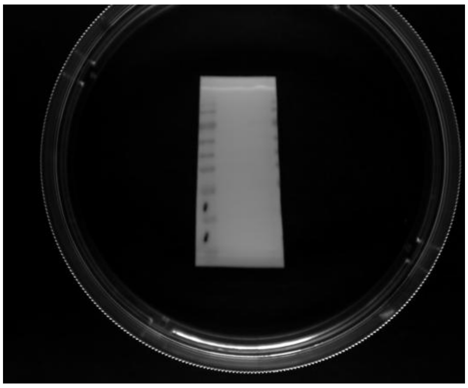

GAPDH

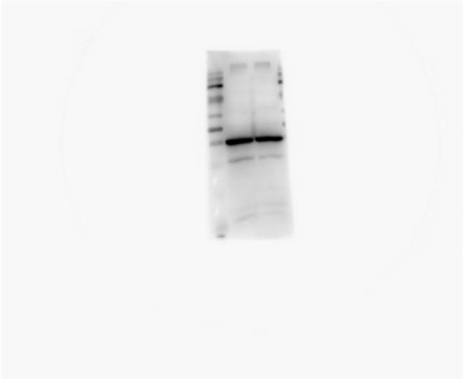

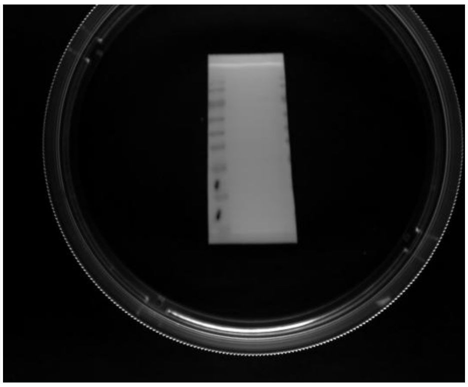

Fig. 3F First Repetition

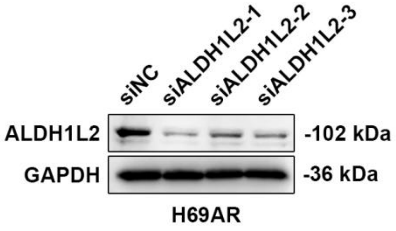

H69AR

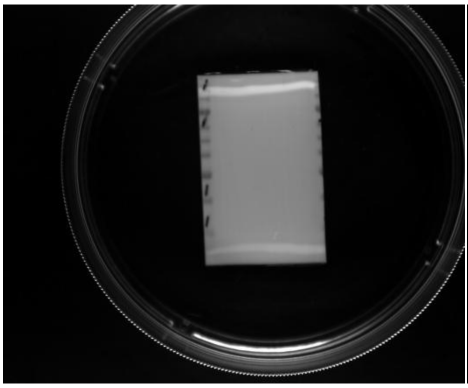

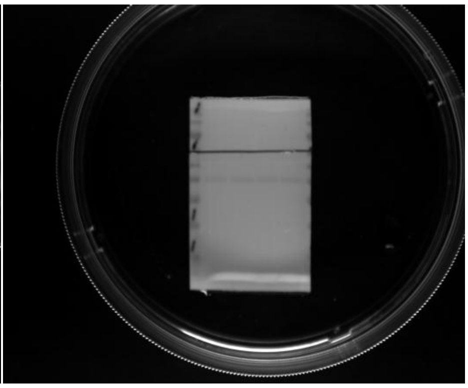

ALDH1L2

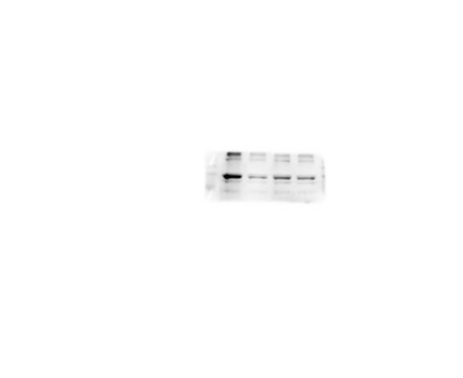

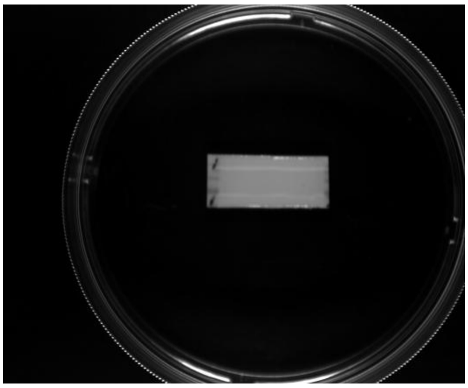

GAPDH

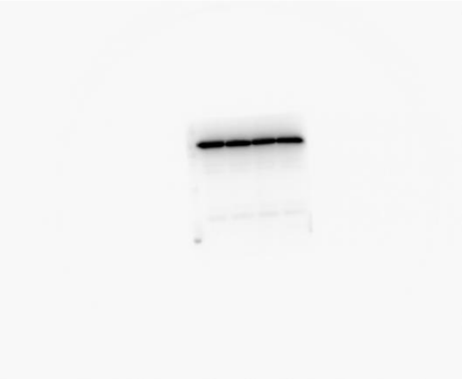

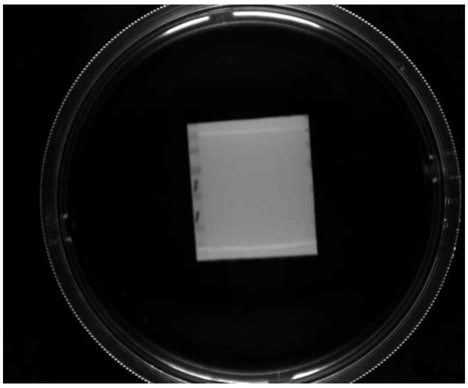

Fig. 3F Second Repetition

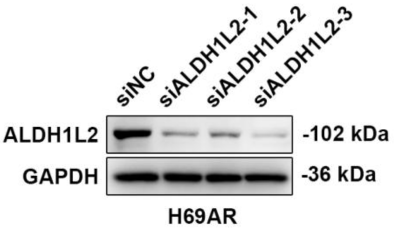

H69AR

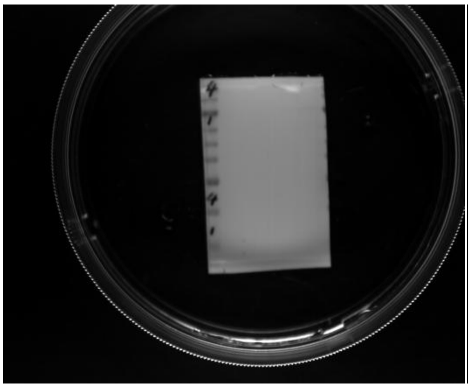

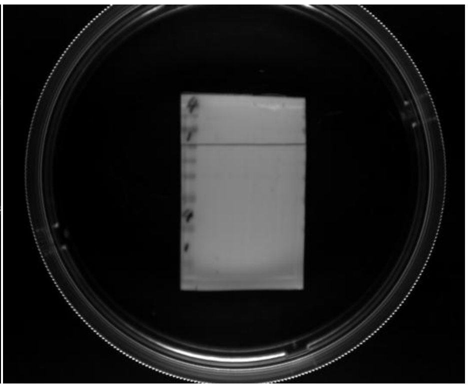

ALDH1L2

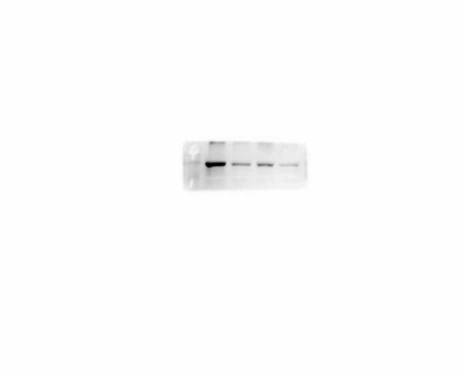

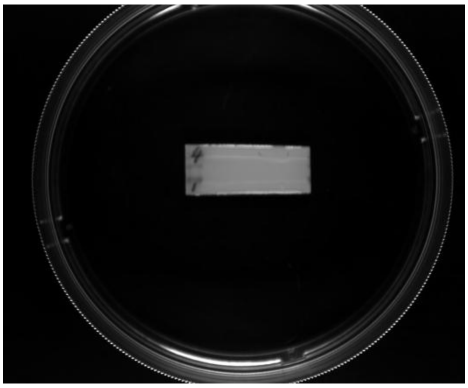

GAPDH

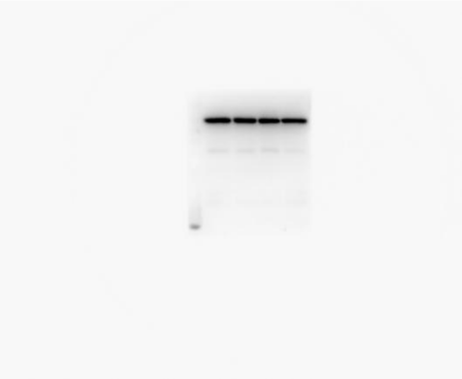

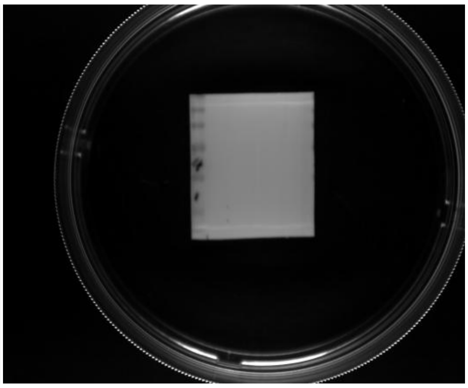

Fig. 3F Third Repetition

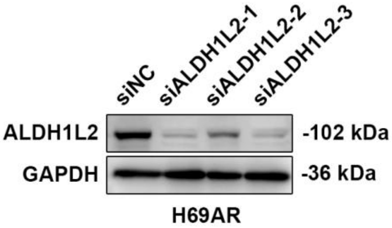

H69AR

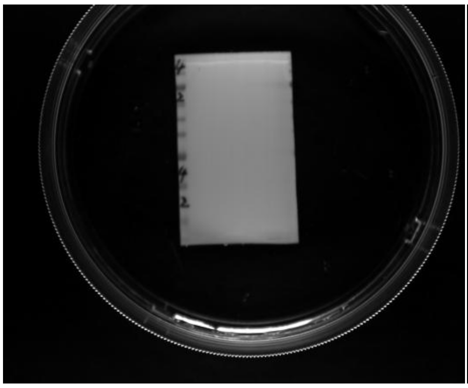

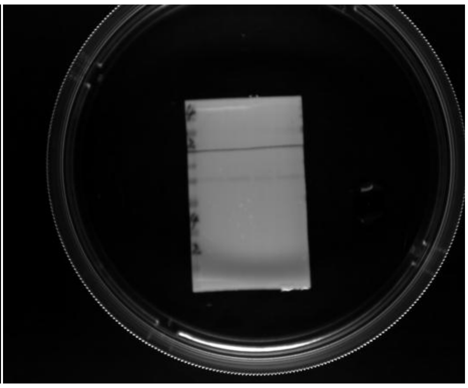

ALDH1L2

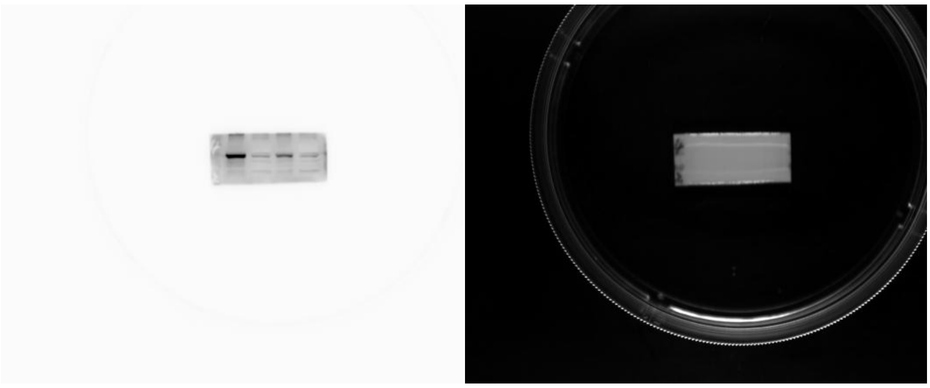

GAPDH

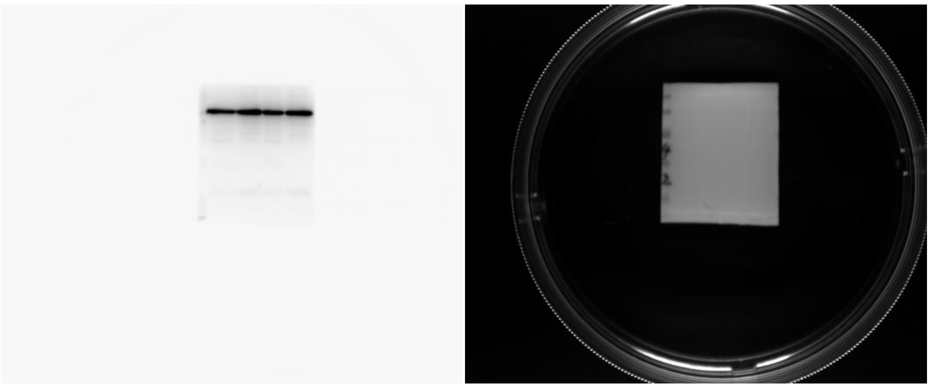

Fig. 3G First Repetition

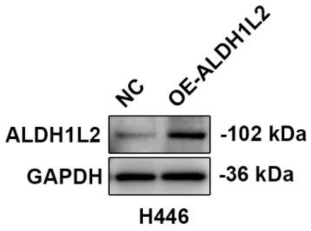

H446

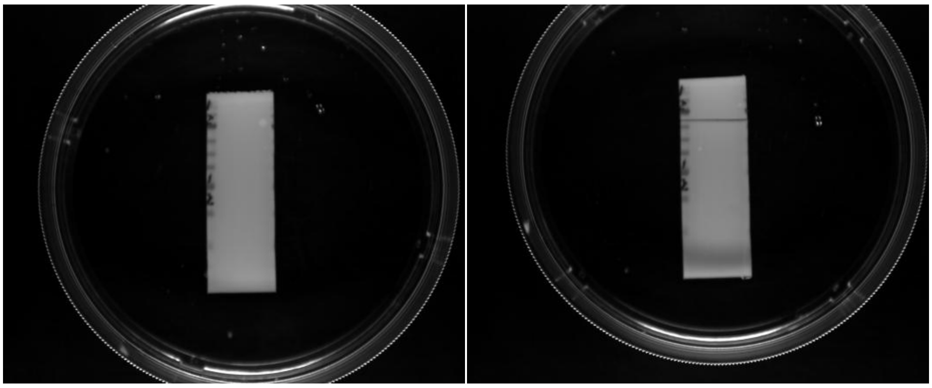

ALDH1L2

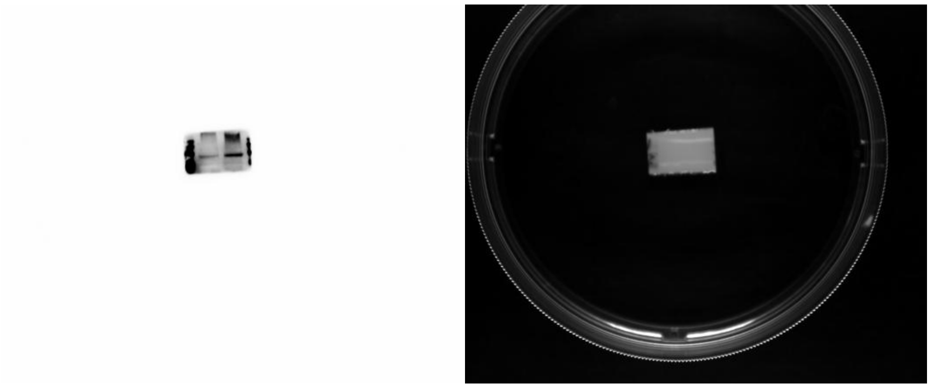

GAPDH

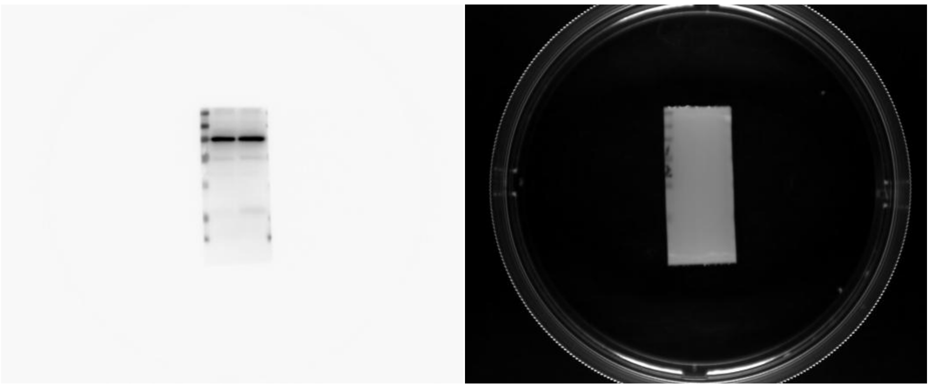

Fig. 3G Second Repetition

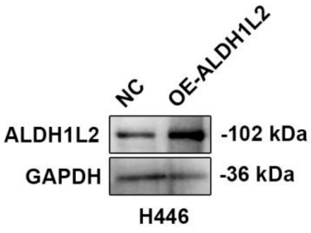

H446

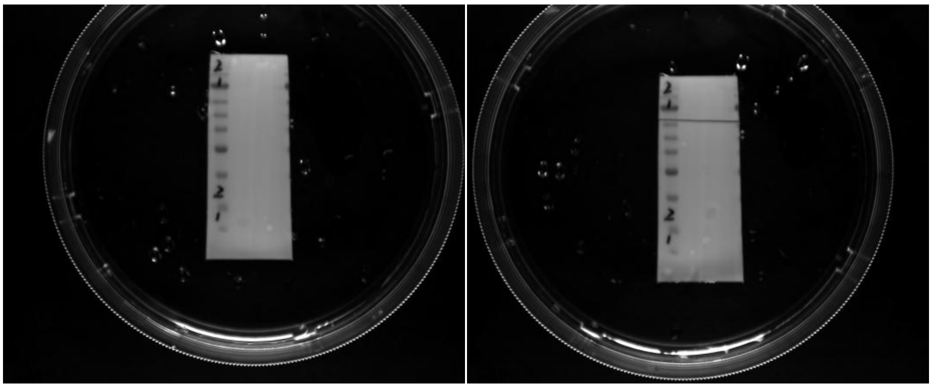

ALDH1L2

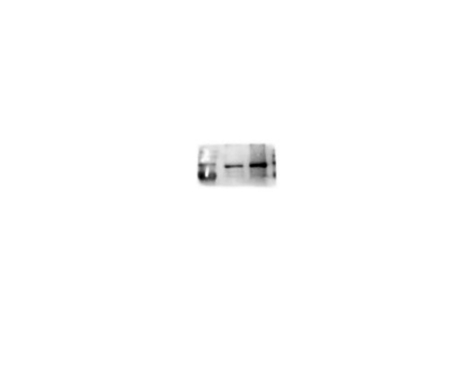
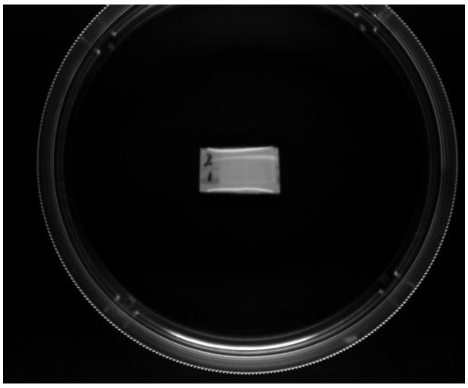

GAPDH

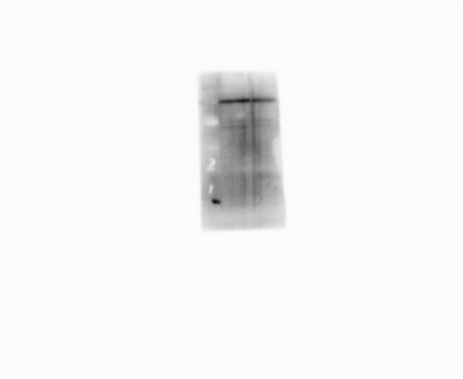
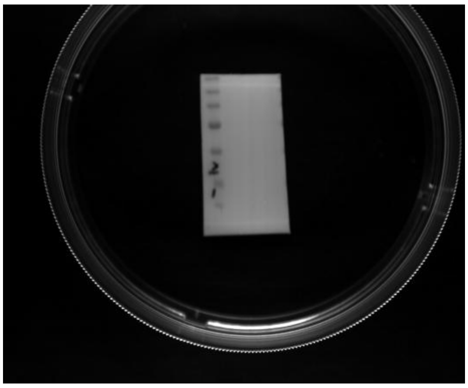

Fig. 3G Third Repetition

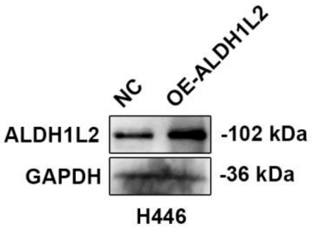

H446

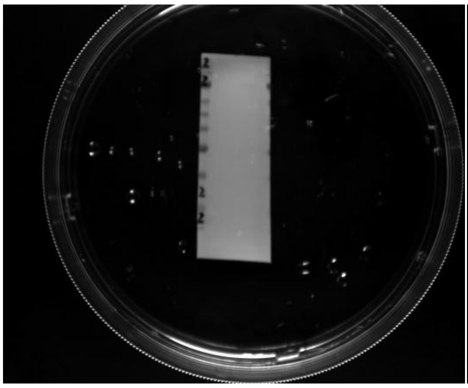
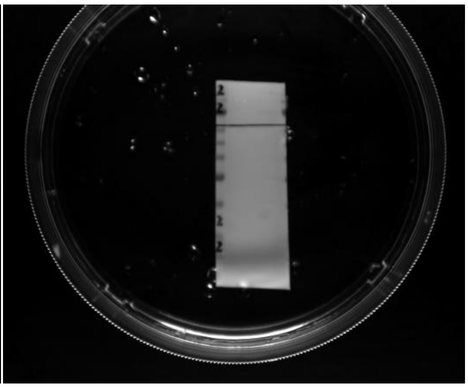

ALDH1L2

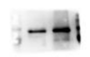

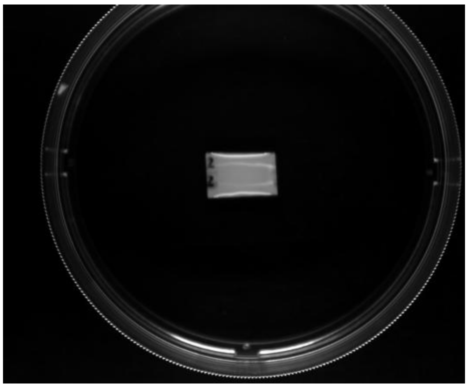

GAPDH

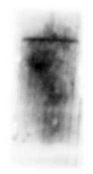

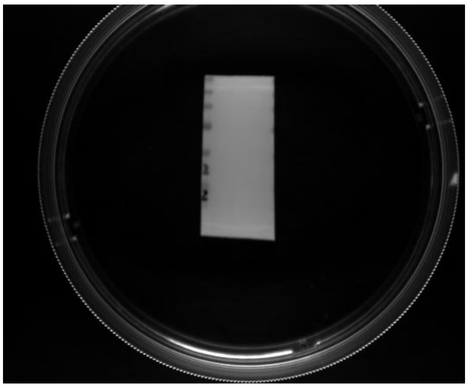

Fig. 3H First Repetition

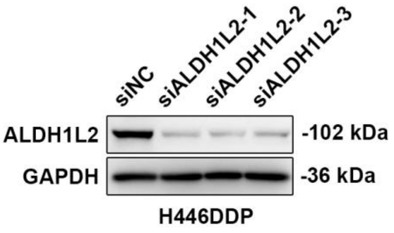

H446DDP

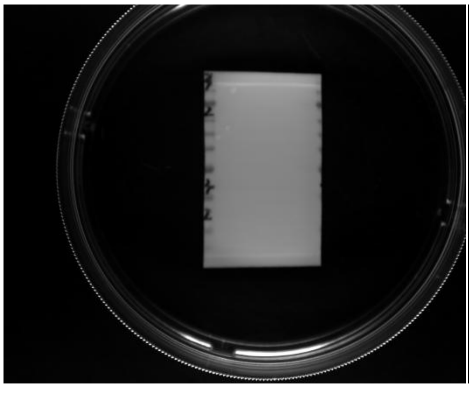

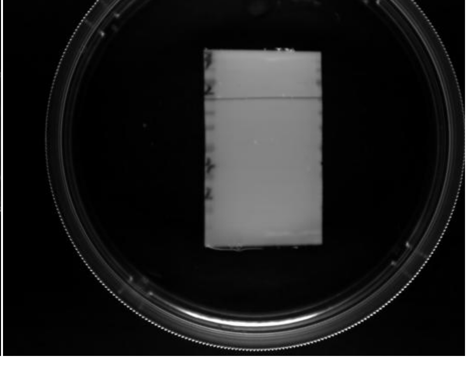

ALDH1L2

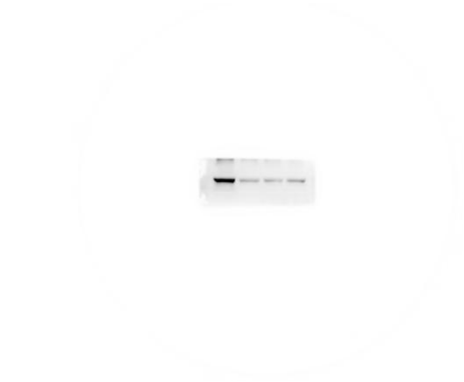

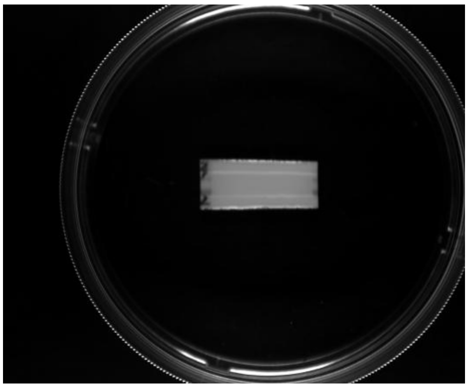

GAPDH

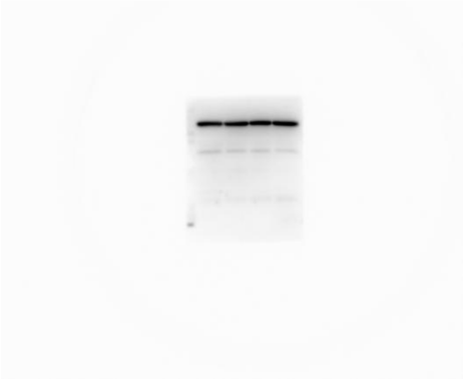

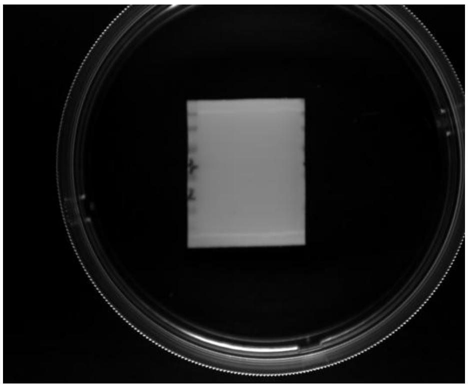

Fig. 3H Second Repetition

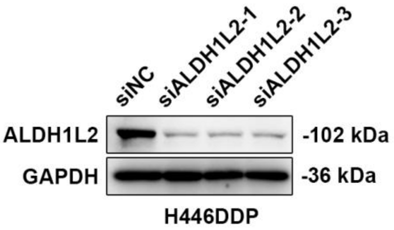

H446DDP

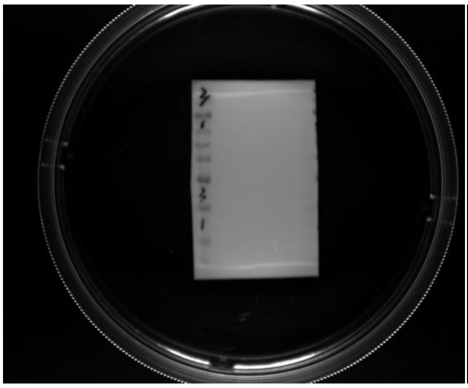

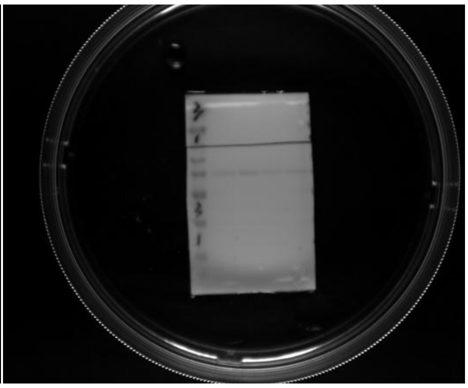

ALDH1L2

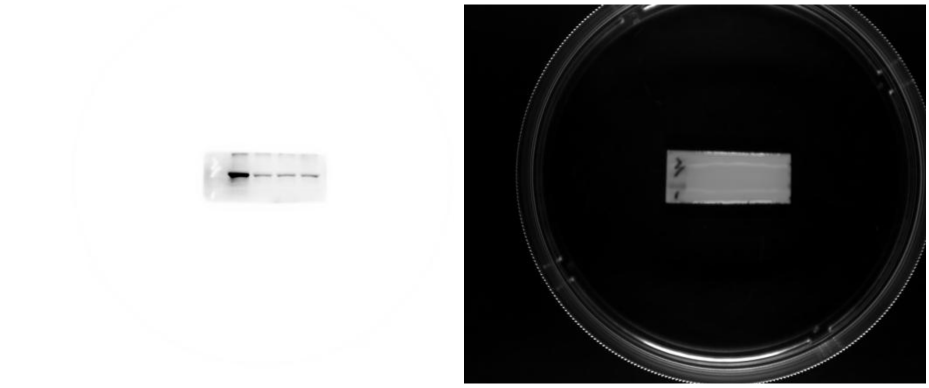

GAPDH

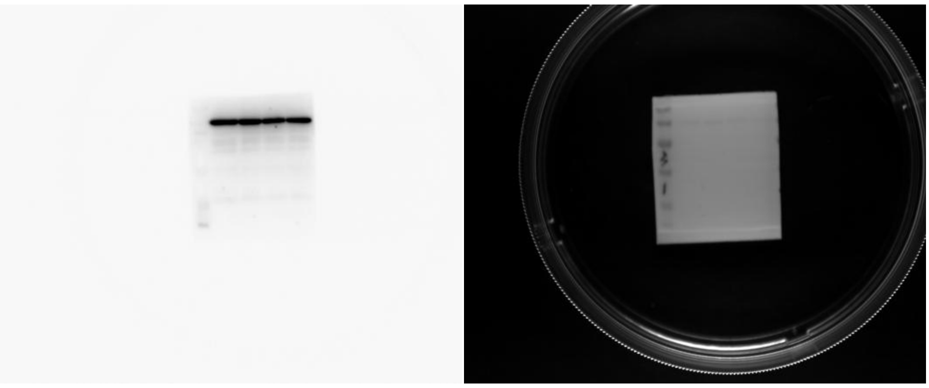

Fig. 3H Third Repetition

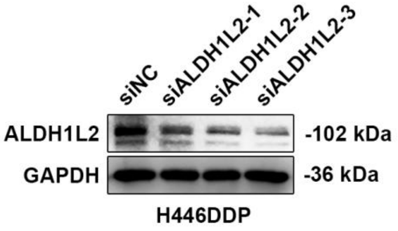

H446DDP

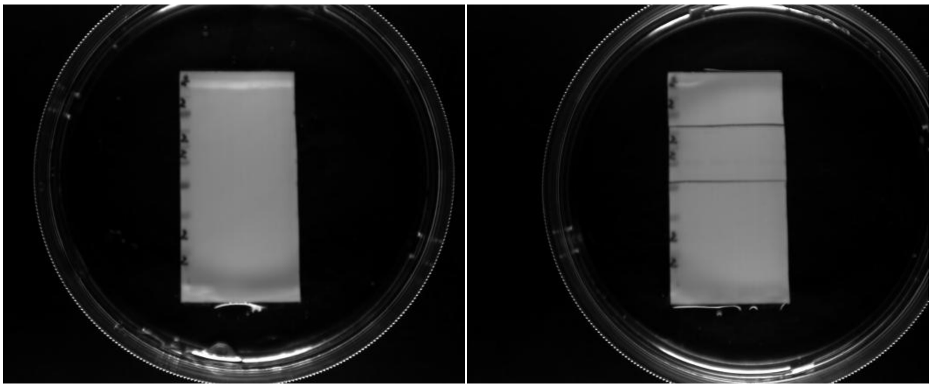

ALDH1L2

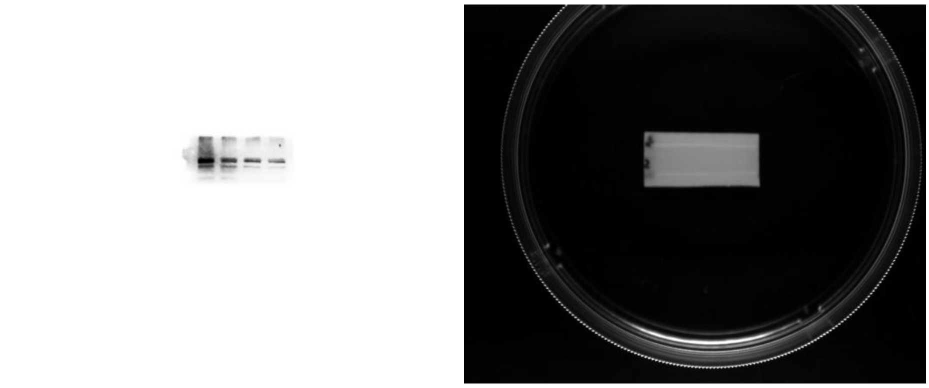

GAPDH

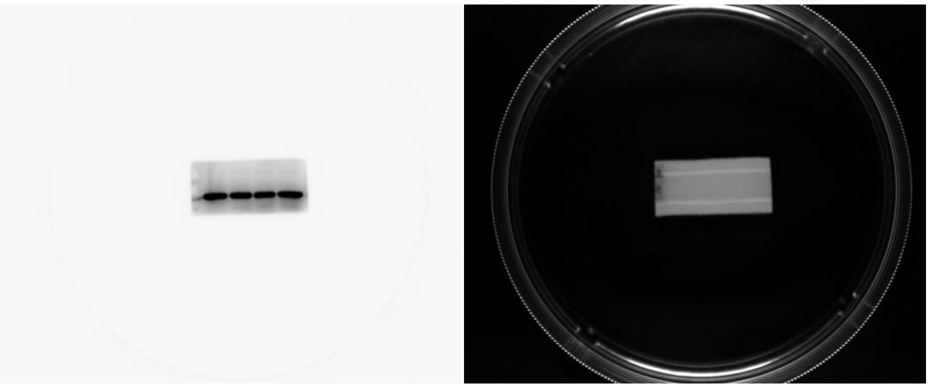

Fig. 5B First Repetition

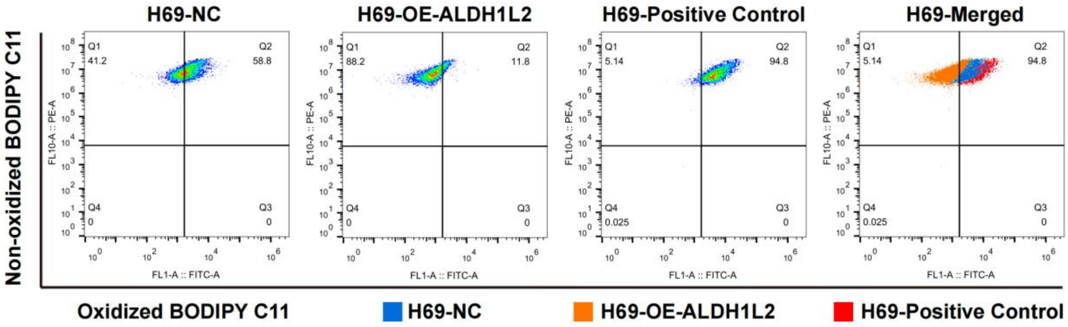

Fig. 5B Second Repetition

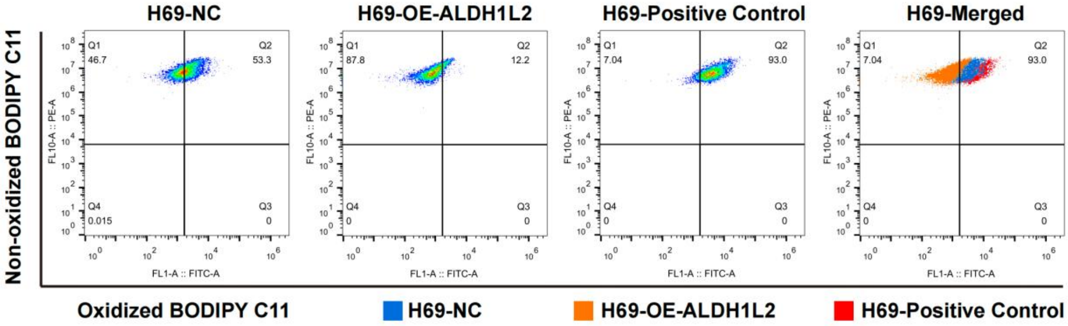

Fig. 5B Third Repetition

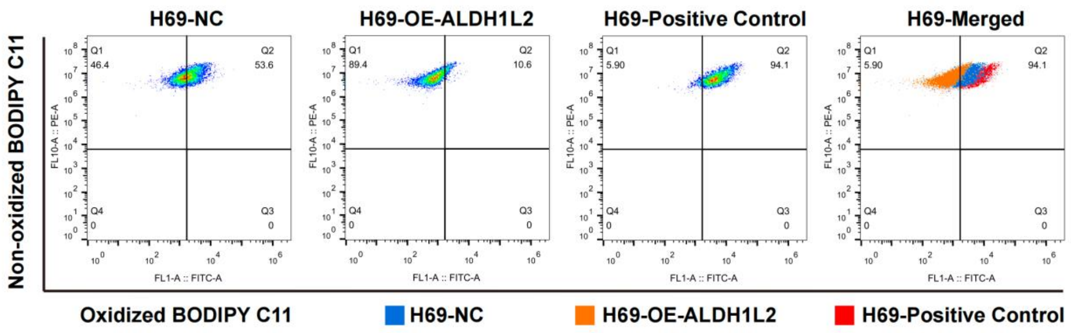

Fig. 5C First Repetition

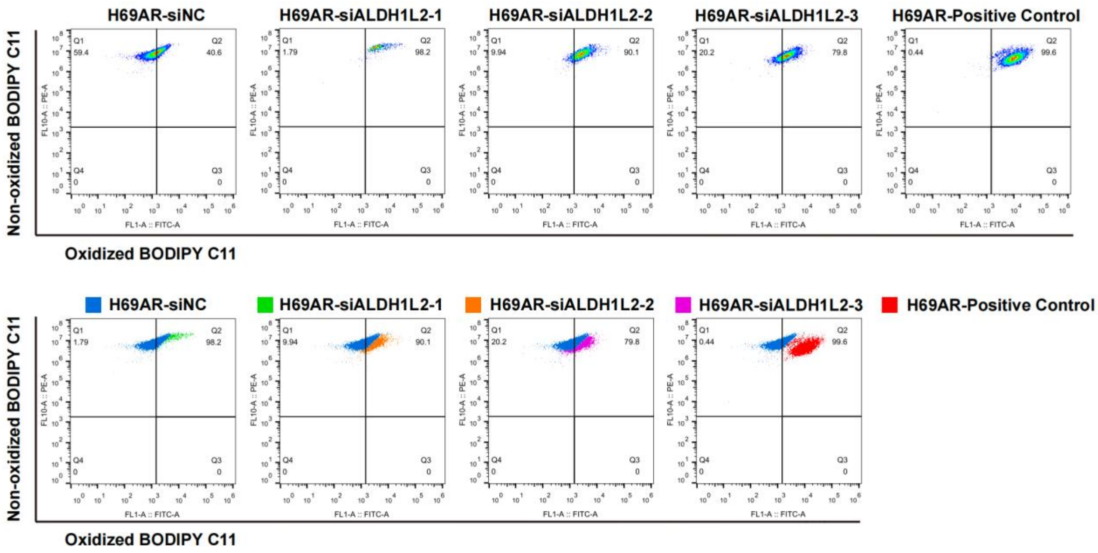

Fig. 5C Second Repetition

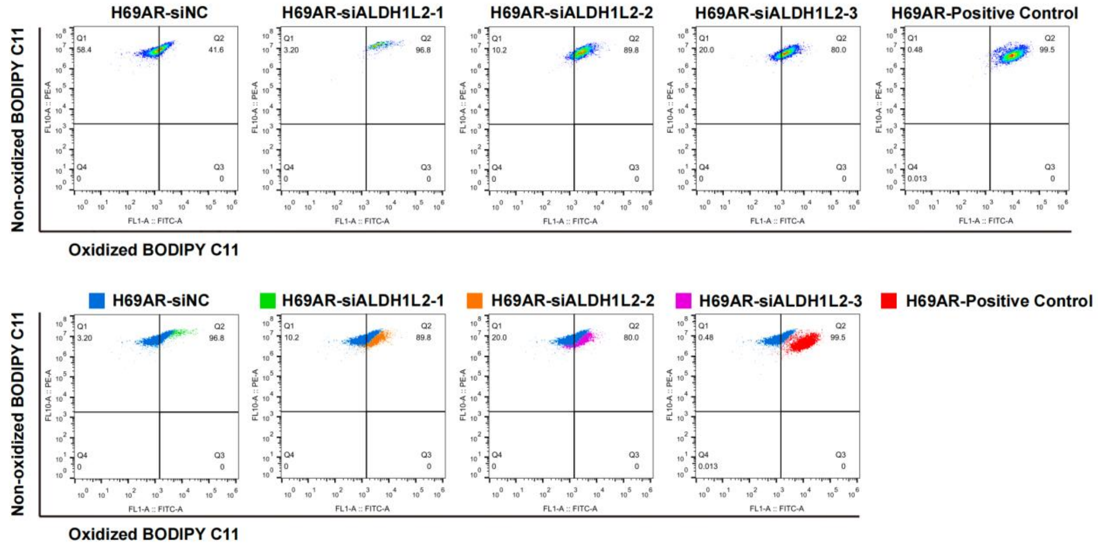

Fig. 5C Third Repetition

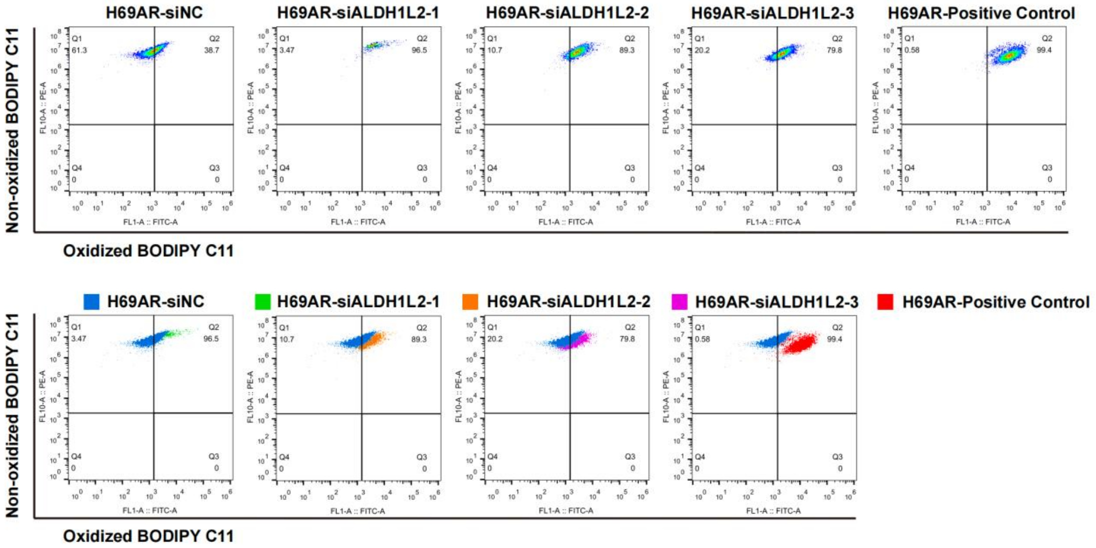

Fig. 5D First Repetition

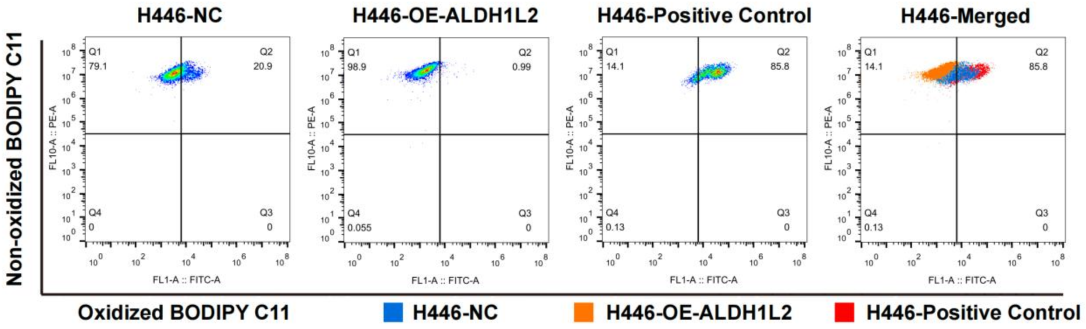

Fig. 5D Second Repetition

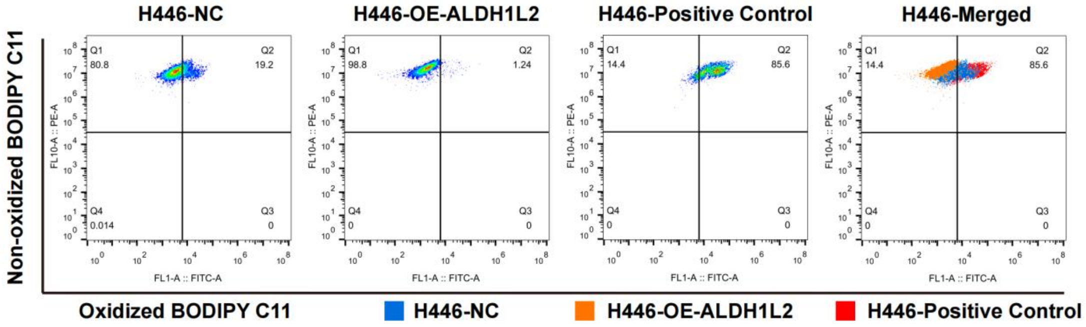

Fig. 5D Third Repetition

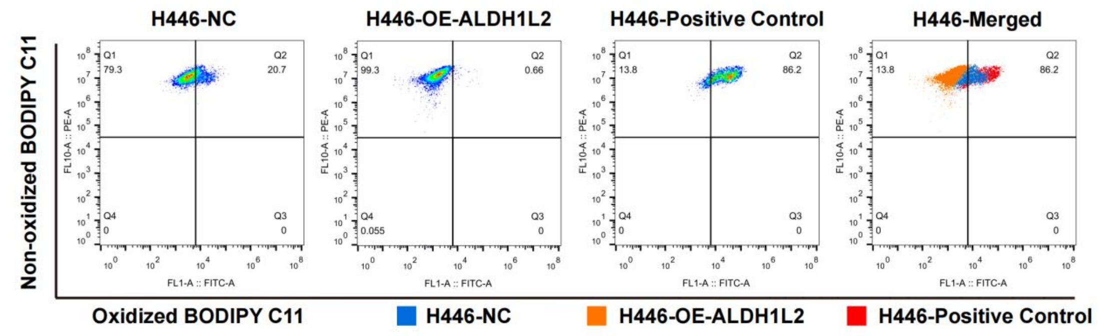

Fig. 5E First Repetition

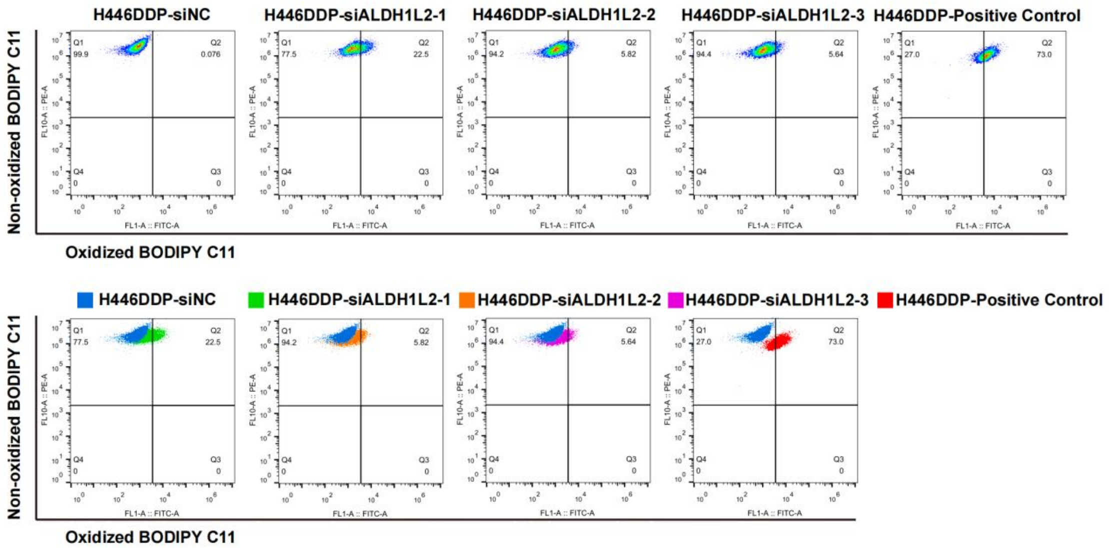

Fig. 5E Second Repetition

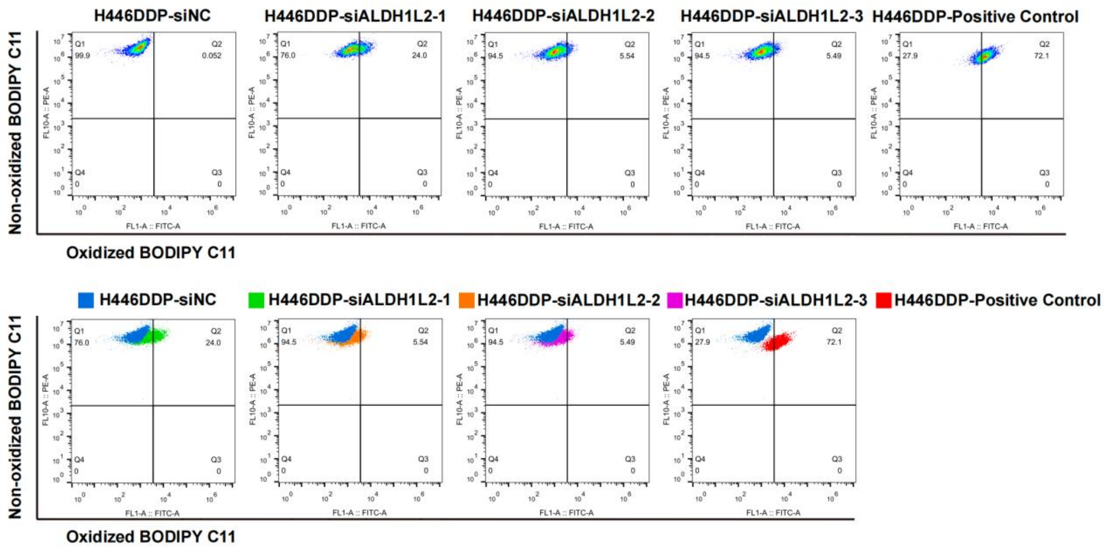

Fig. 5E Third Repetition

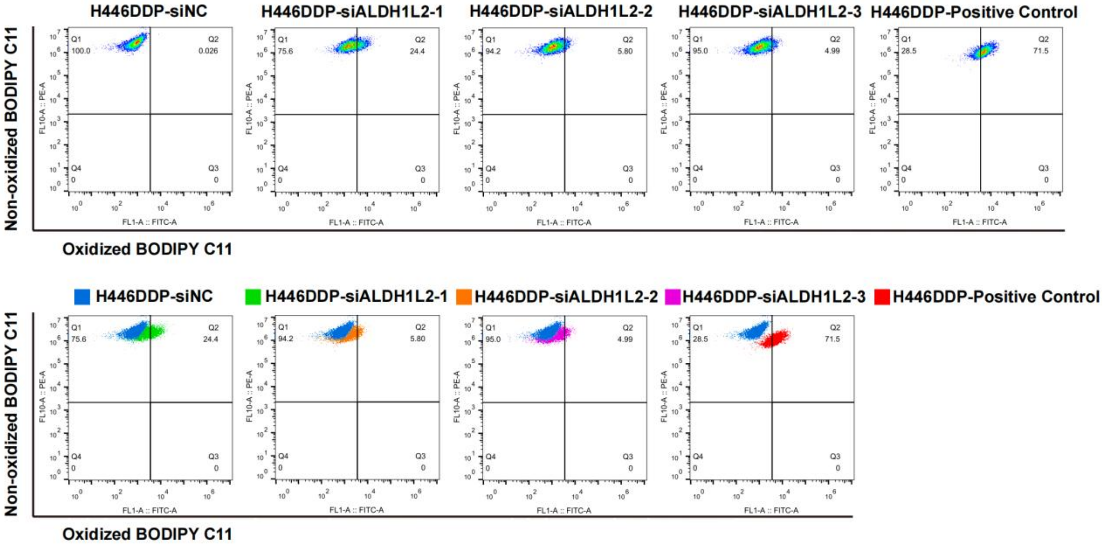

Fig. 5J First Repetition

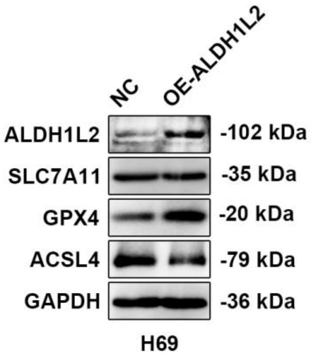

H69

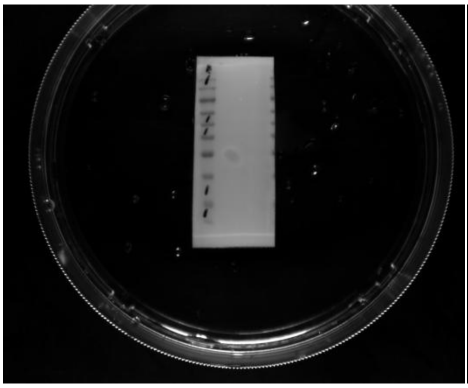

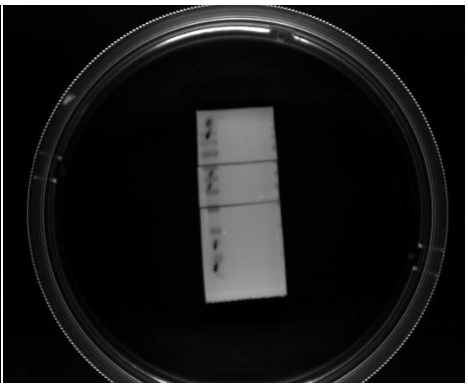

ALDH1L2

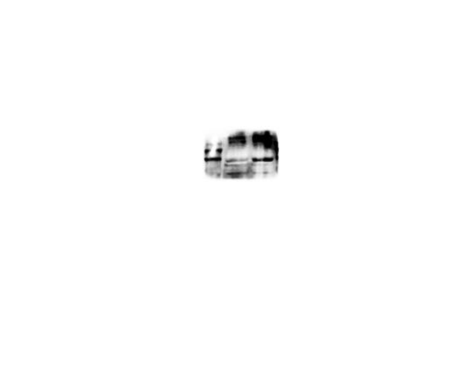

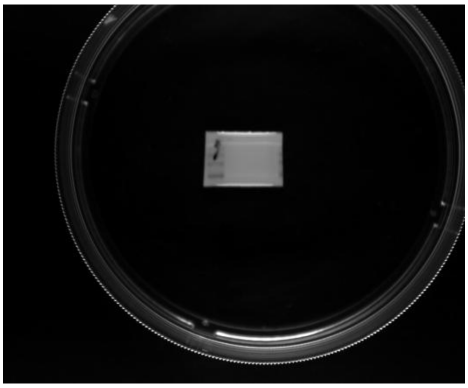

SLC7A11

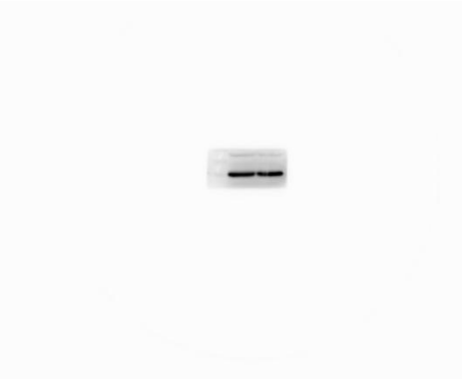

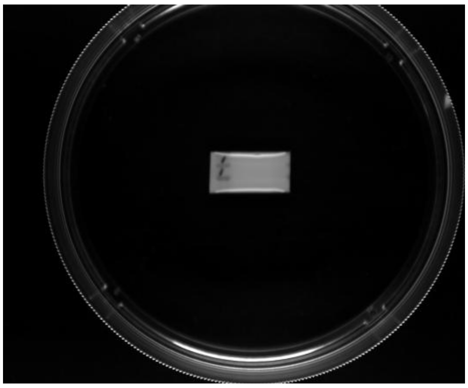

GPX4

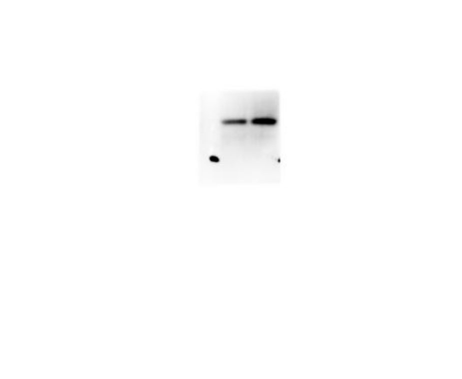

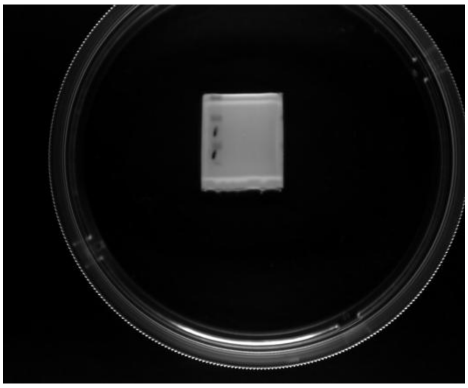

ACSL4

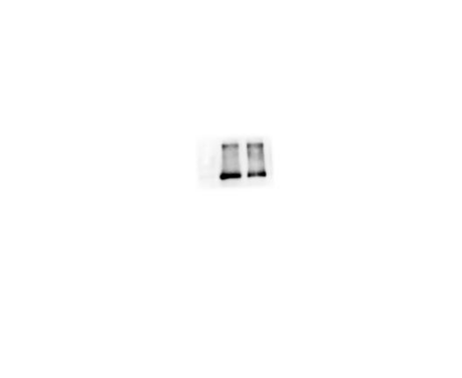

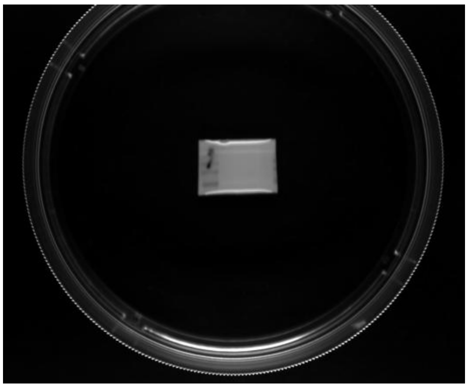

GAPDH

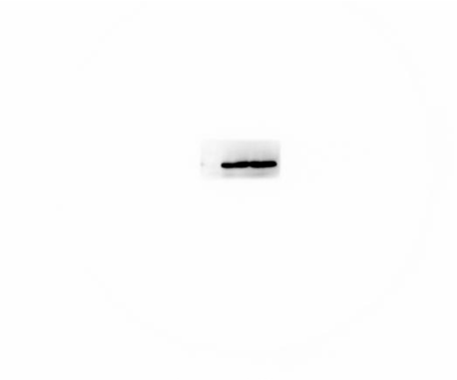

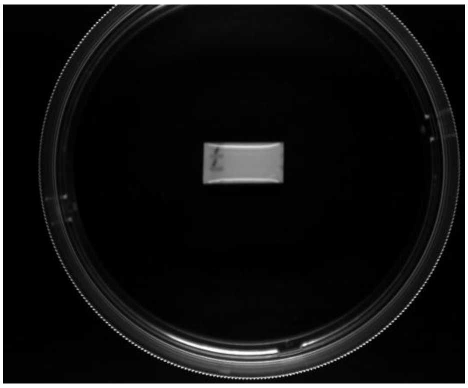

Fig. 5J Second Repetition

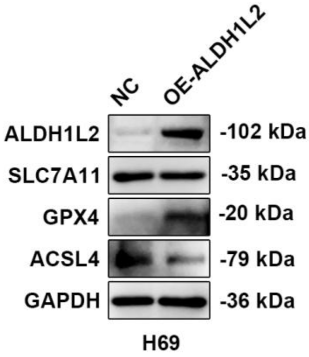

H69

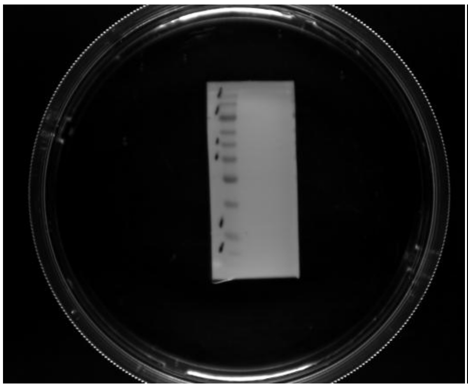

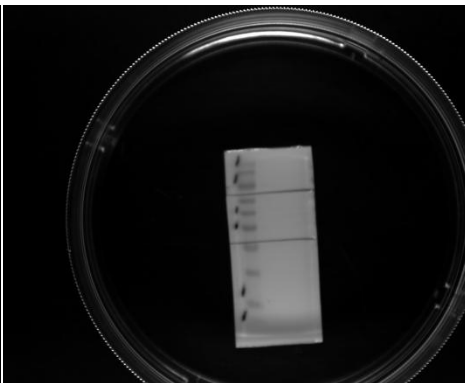

ALDH1L2

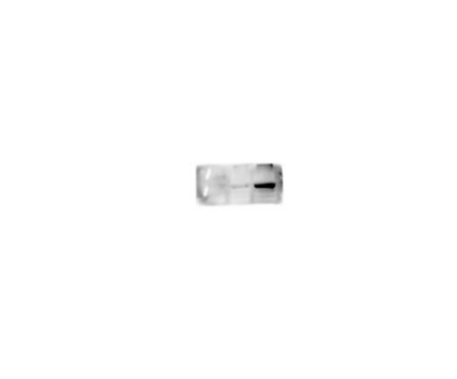

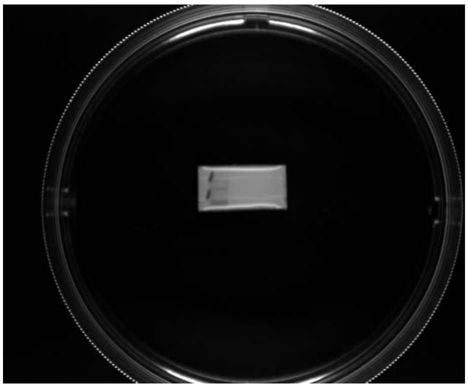

SLC7A11

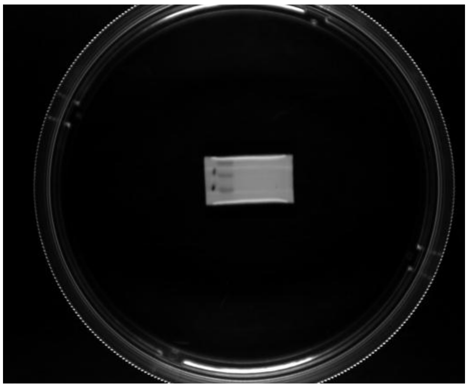

GPX4

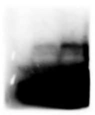
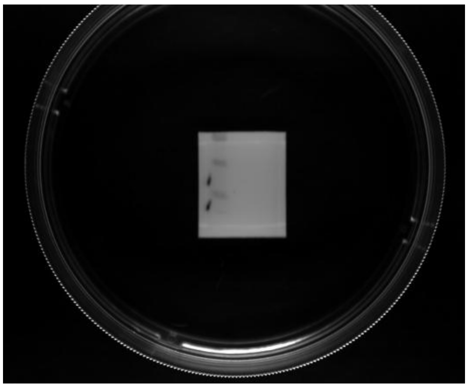

ACSL4

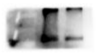
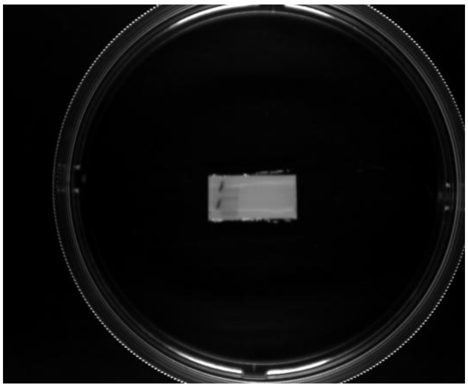

GAPDH

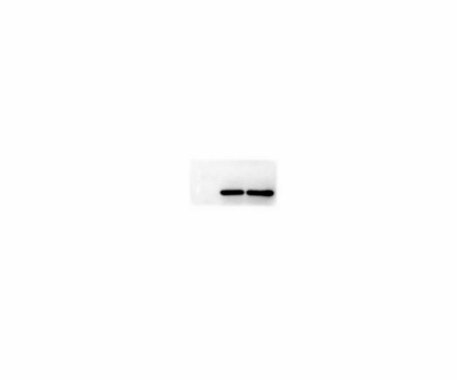
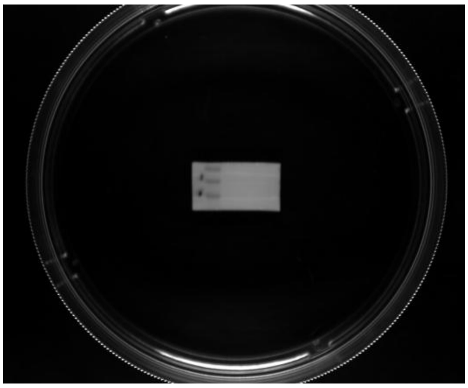

Fig. 5J Third Repetition

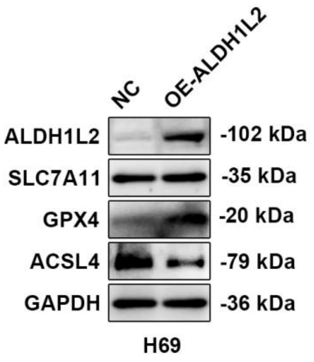

H69

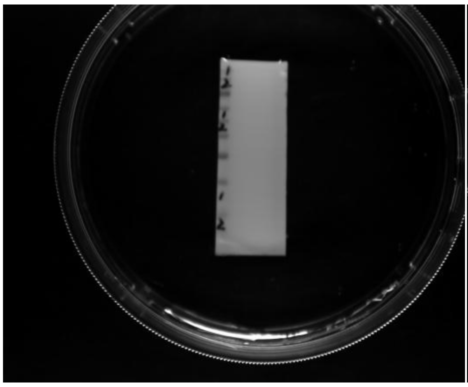

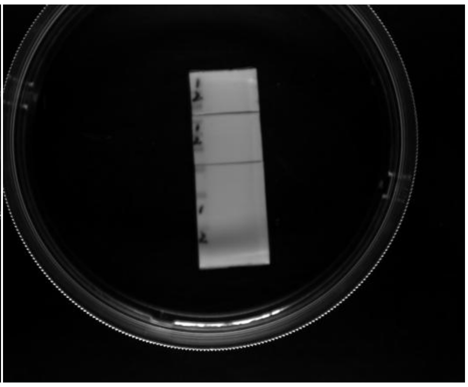

ALDH1L2

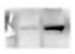

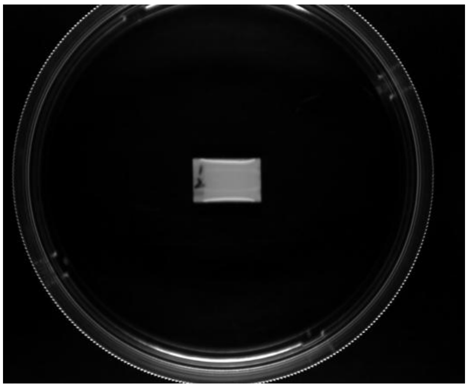

SLC7A11

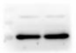

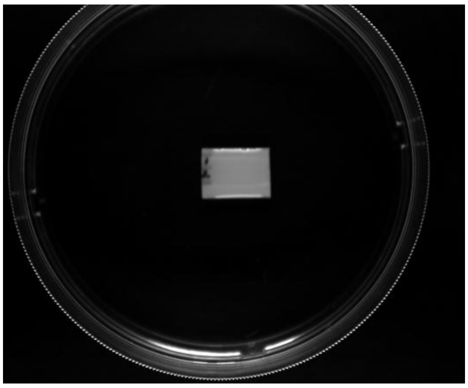

GPX4

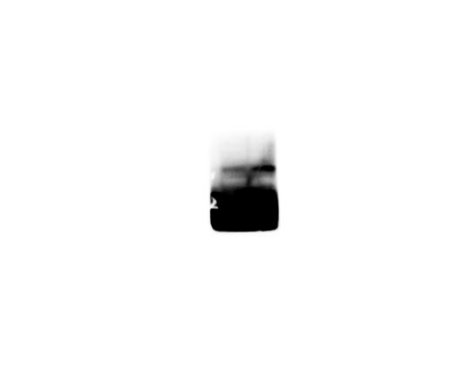

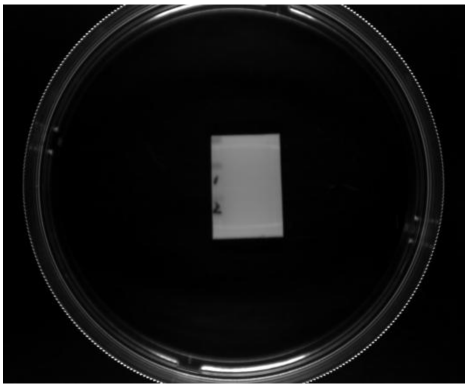

ACSL4

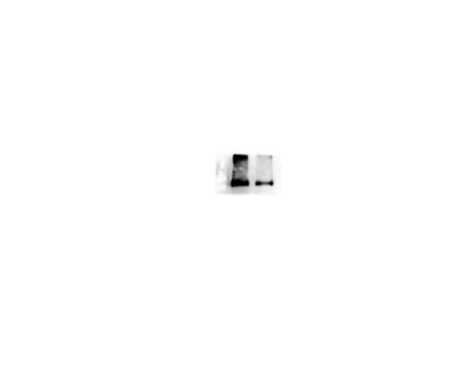

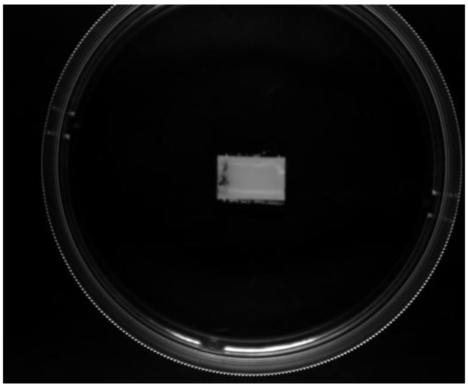

GAPDH

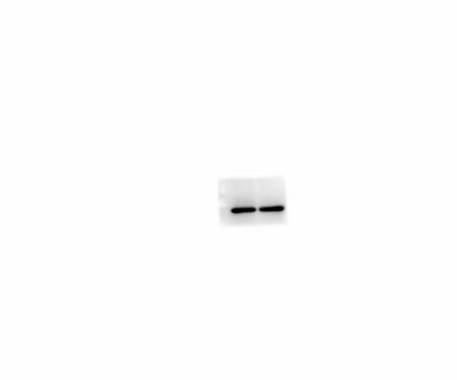

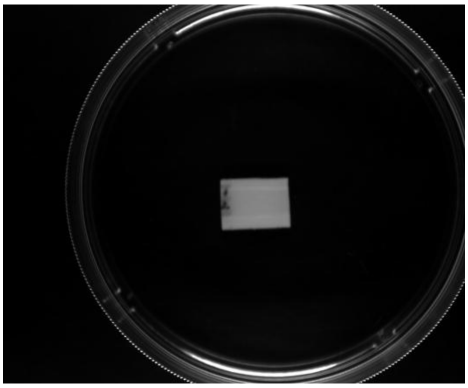

Fig. 5K First Repetition

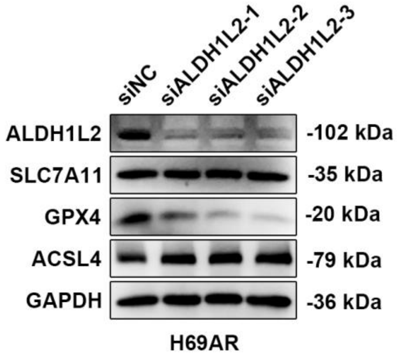

H69AR

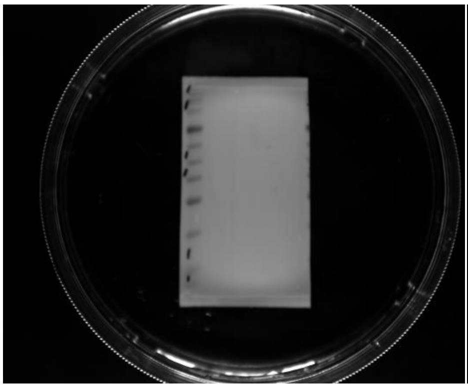

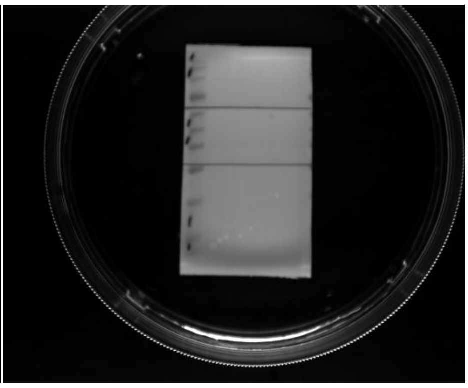

ALDH1L2

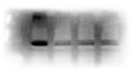

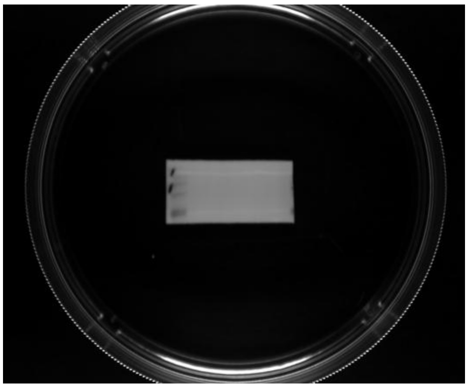

SLC7A11

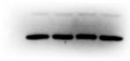

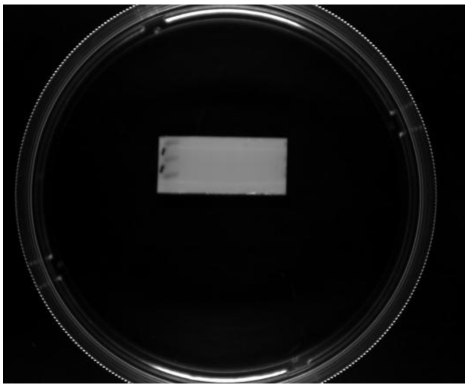

GPX4

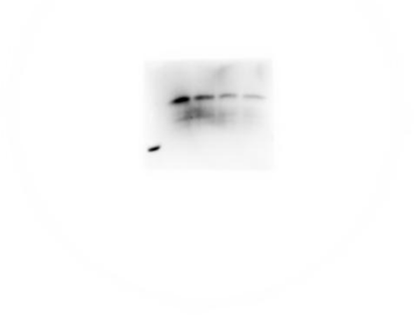

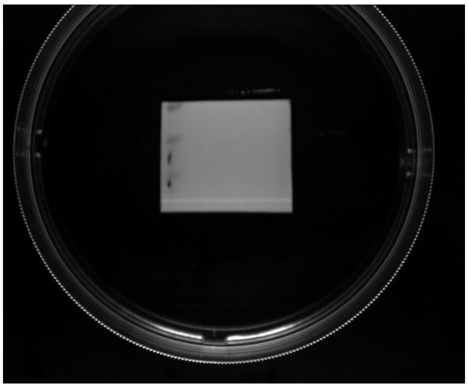

ACSL4

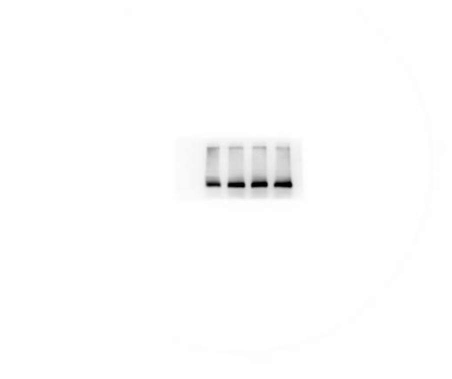

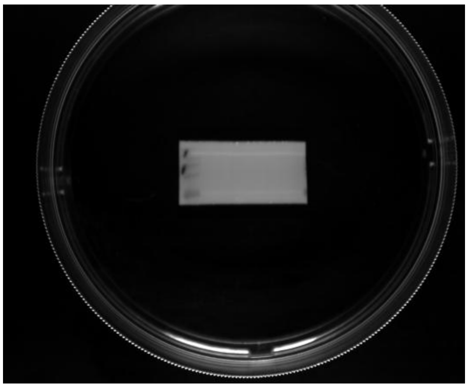

GAPDH

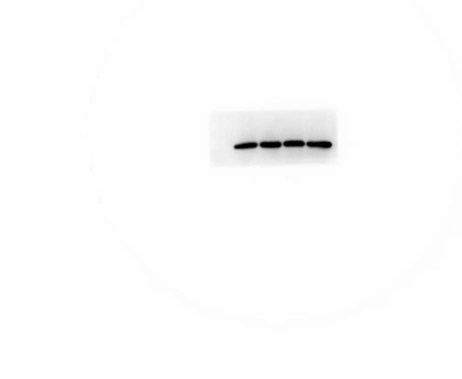

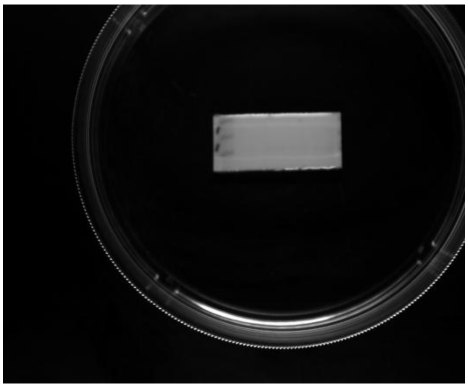

Fig. 5K Second Repetition

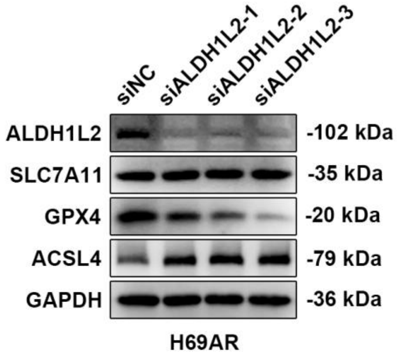

H69AR

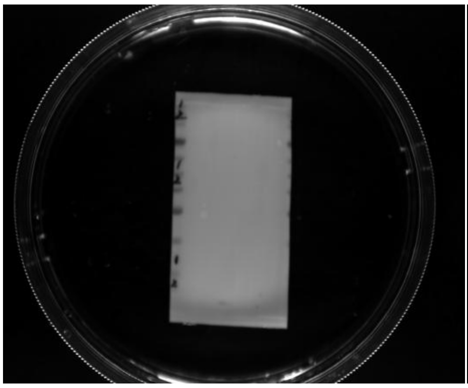

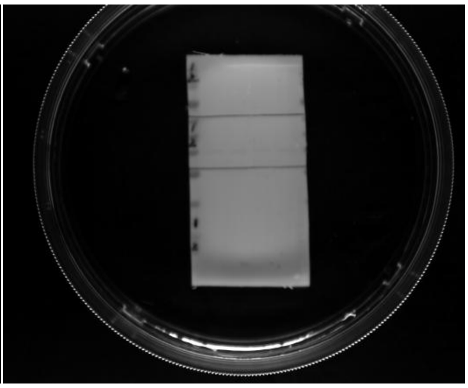

ALDH1L2

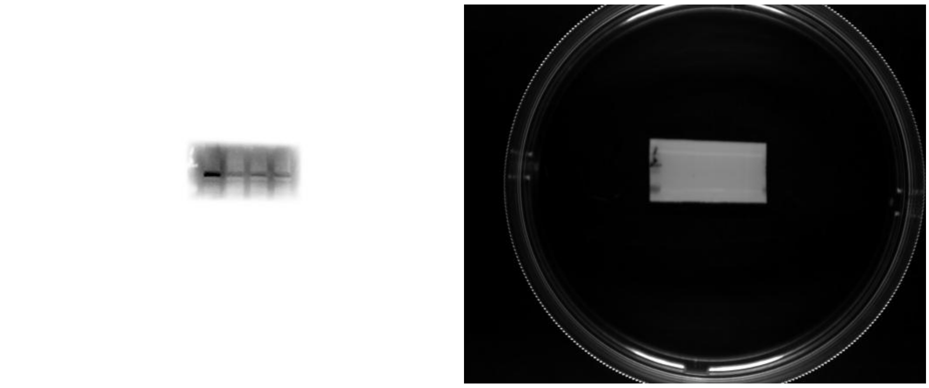

SLC7A11

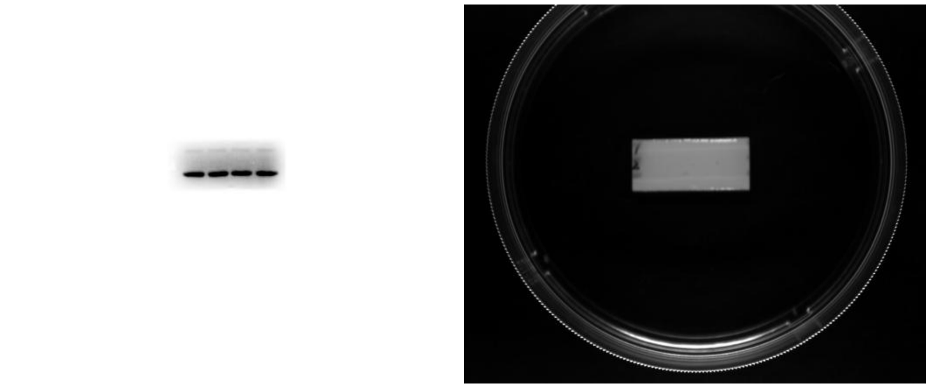

GPX4

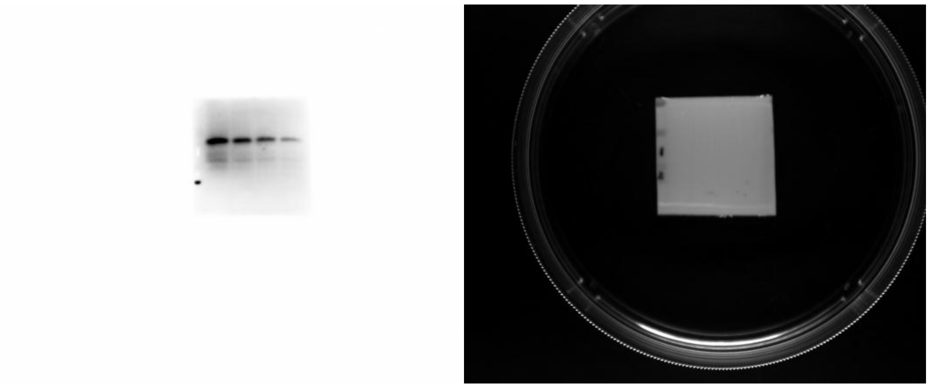

ACSL4

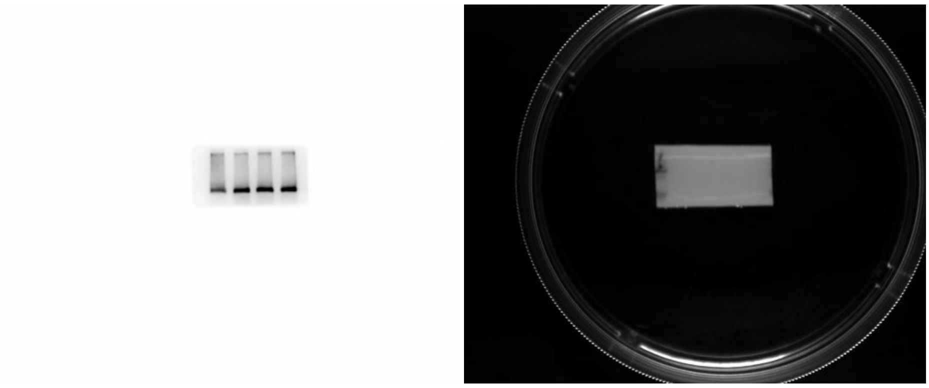

GAPDH

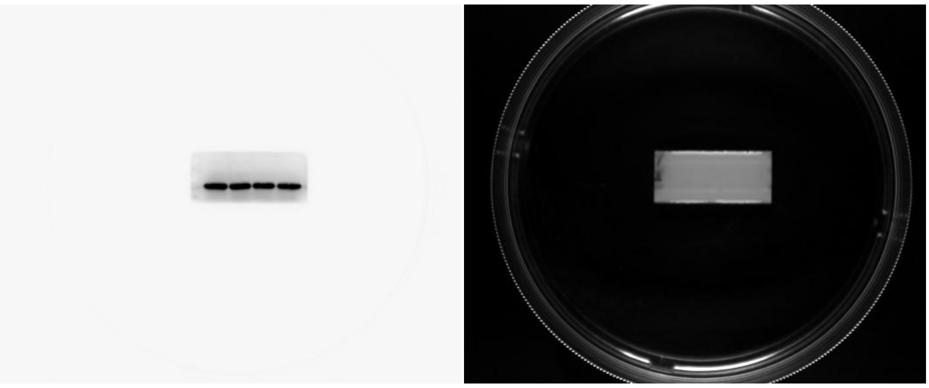

Fig. 5K Third Repetition

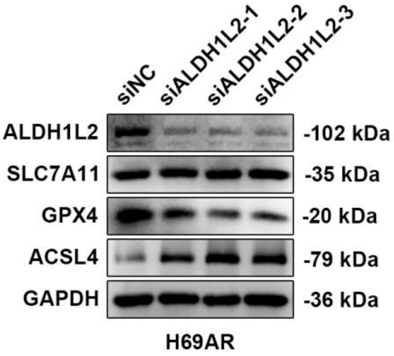

H69AR

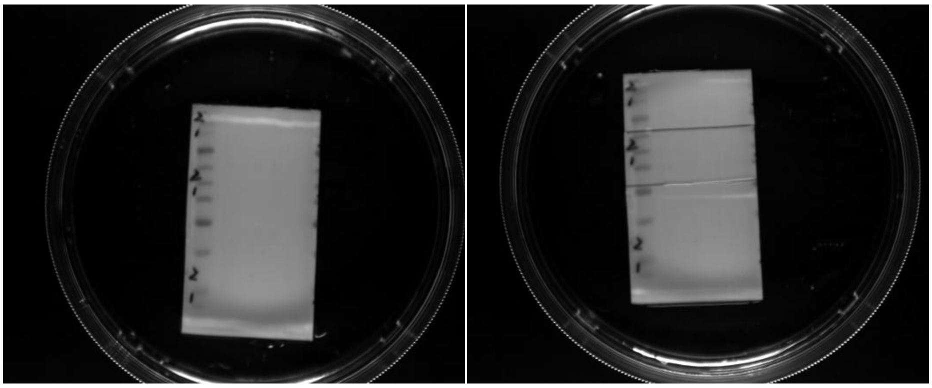

ALDH1L2

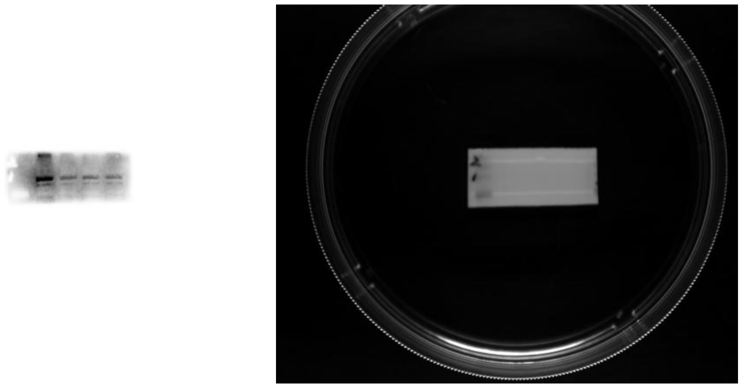

SLC7A11

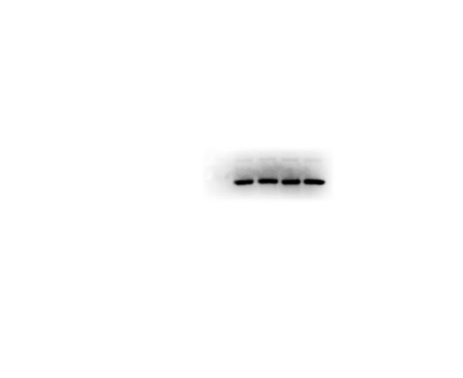
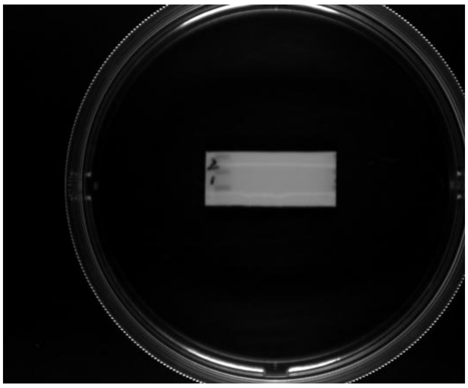

GPX4

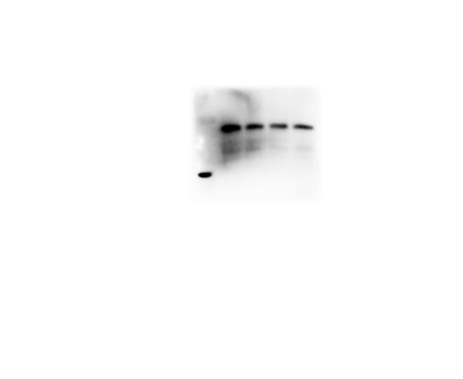
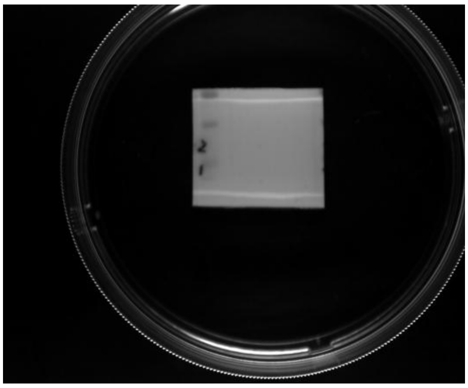

ACSL4

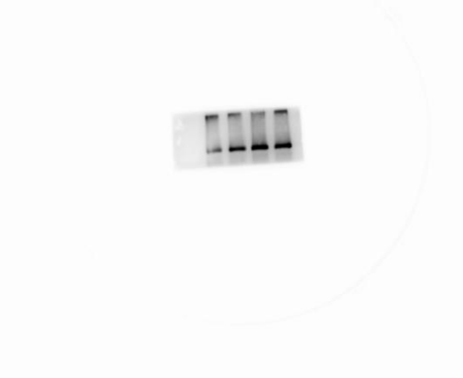
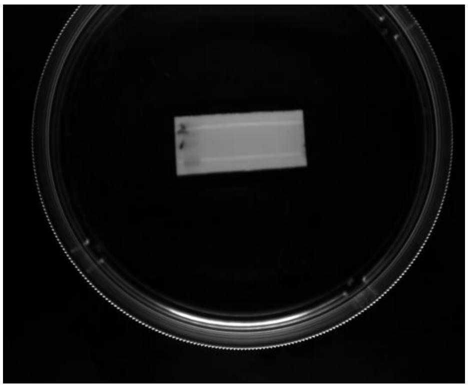

GAPDH

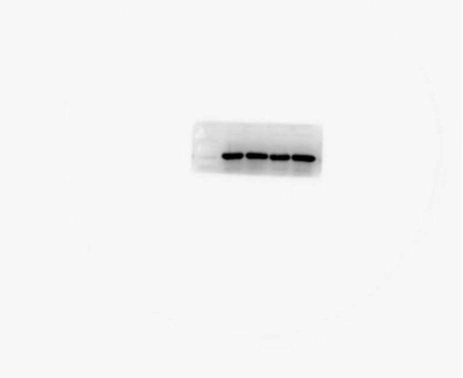
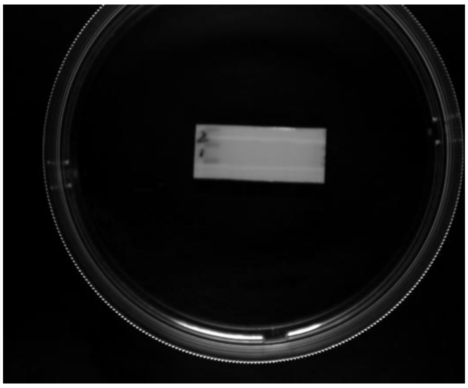

Fig. 5N First Repetition

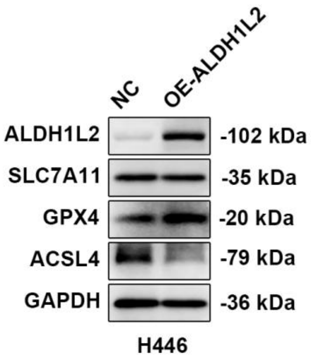

H446

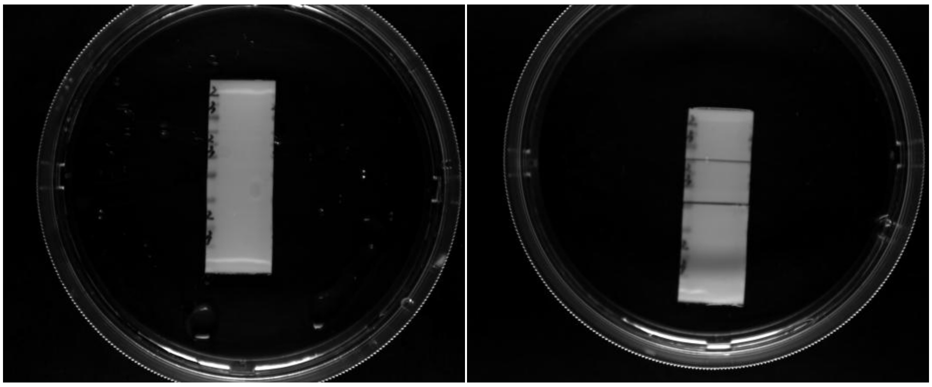

ALDH1L2

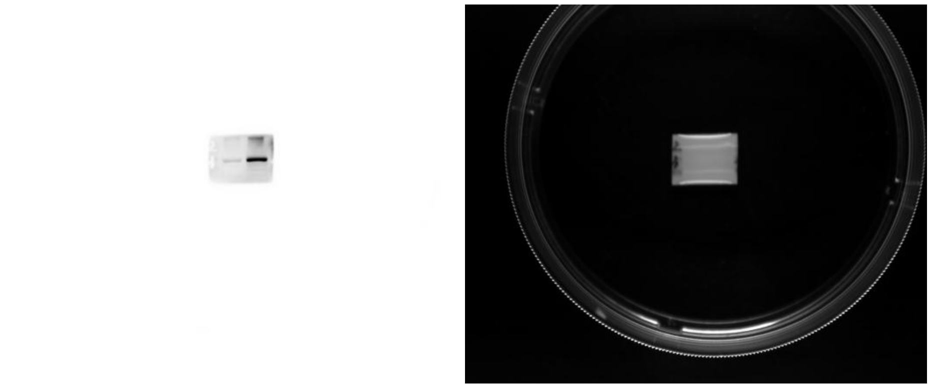

SLC7A11

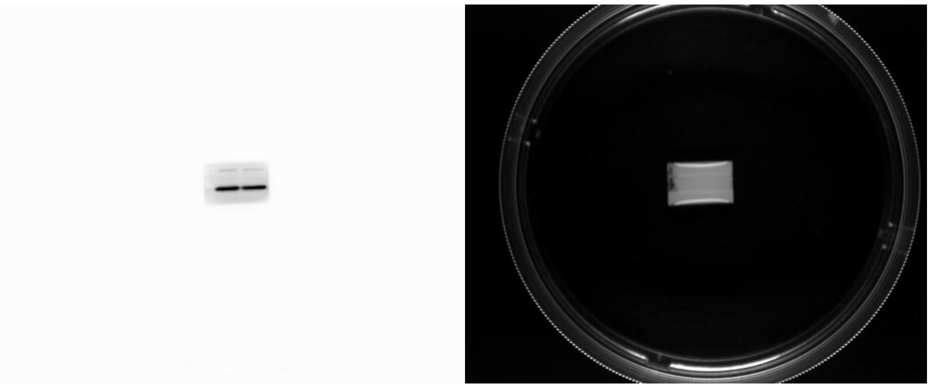

GPX4

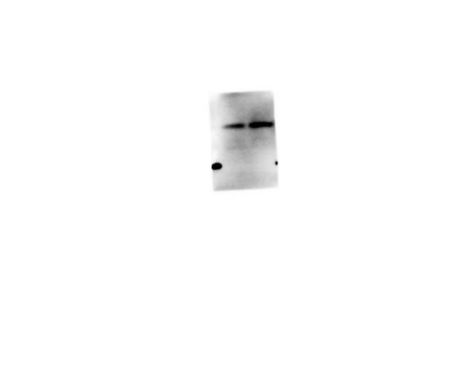

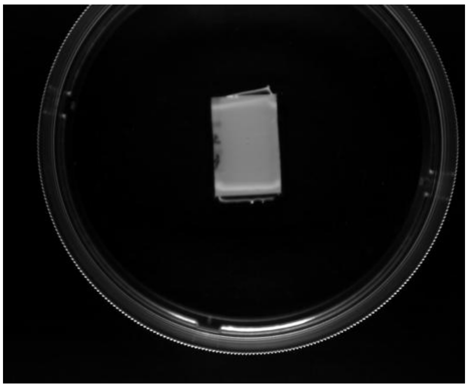

ACSL4

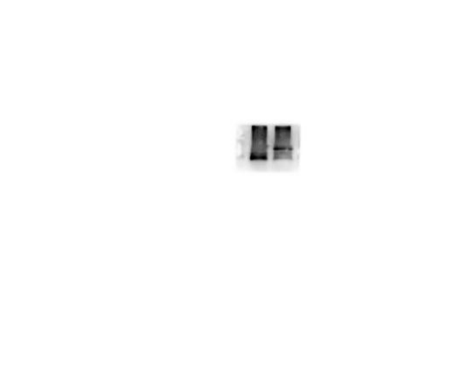

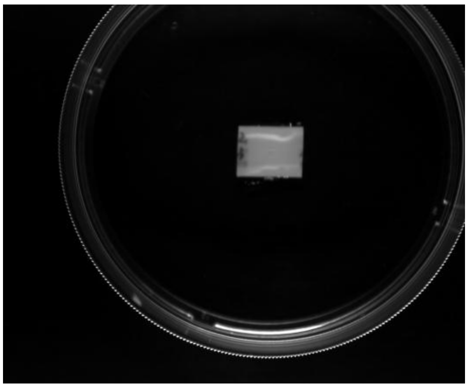

GAPDH

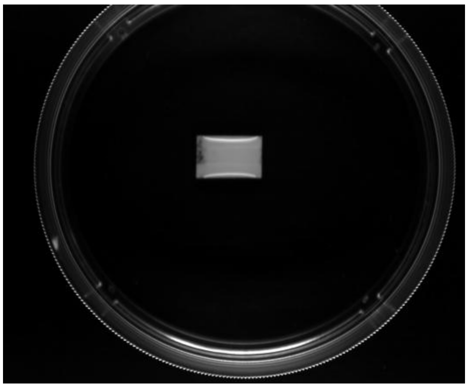

Fig. 5N Second Repetition

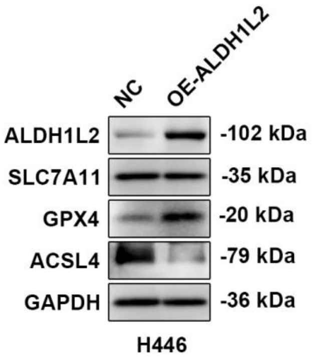

H446

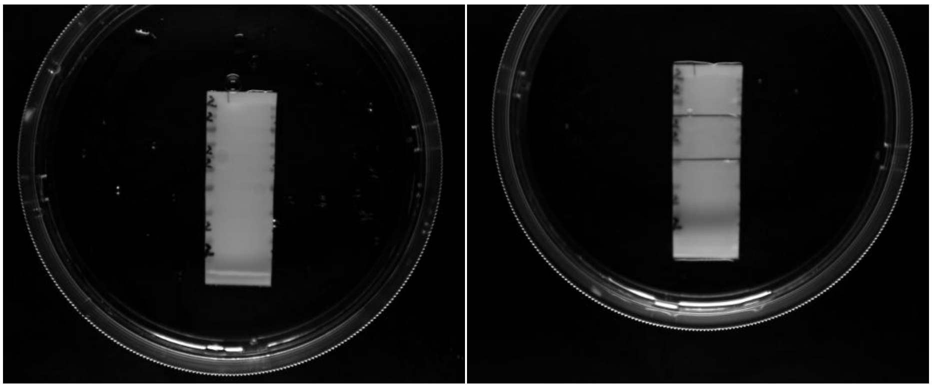

ALDH1L2

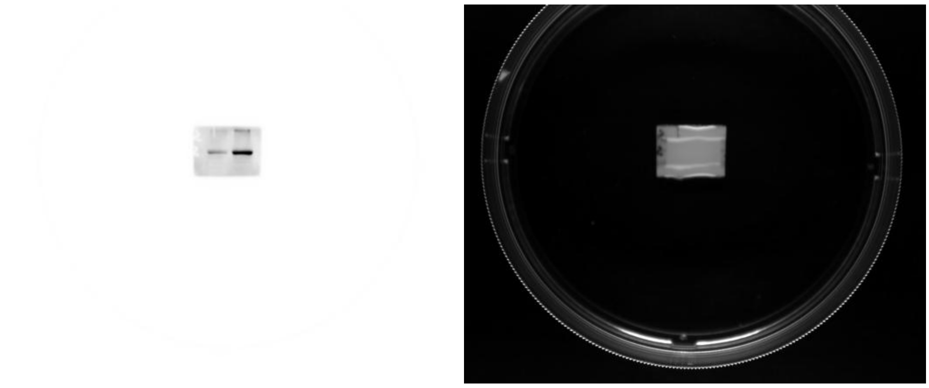

SLC7A11

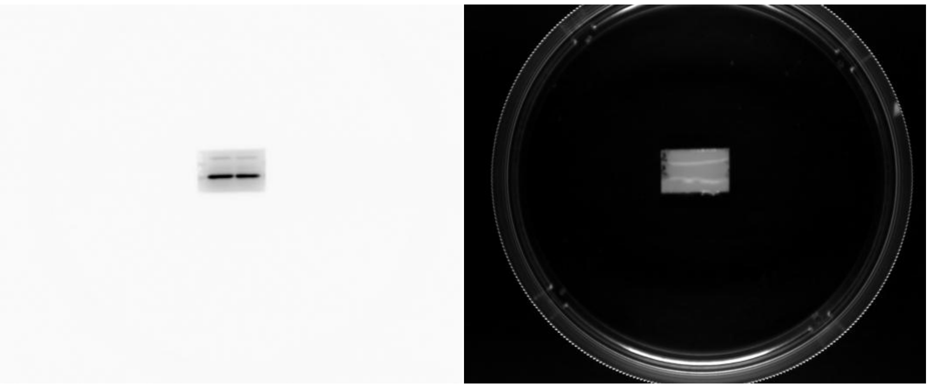

GPX4

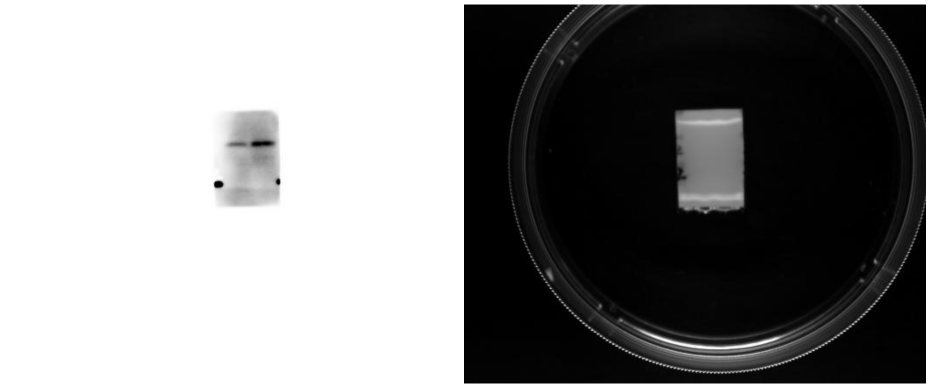

ACSL4

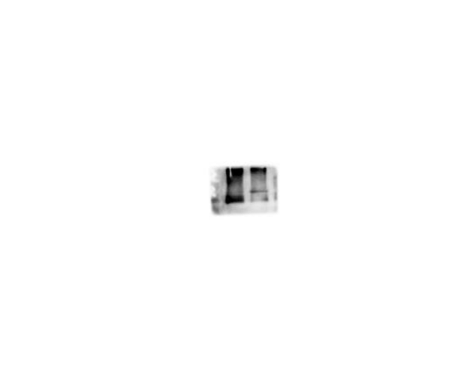

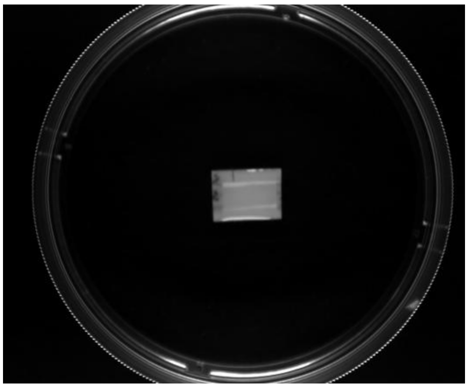

GAPDH

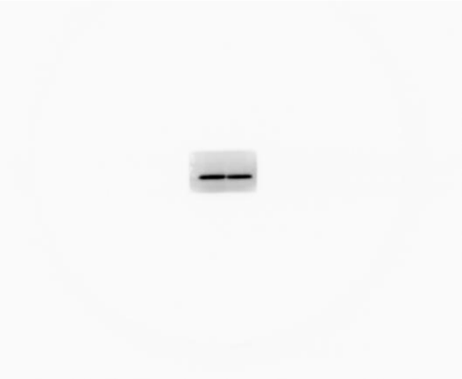

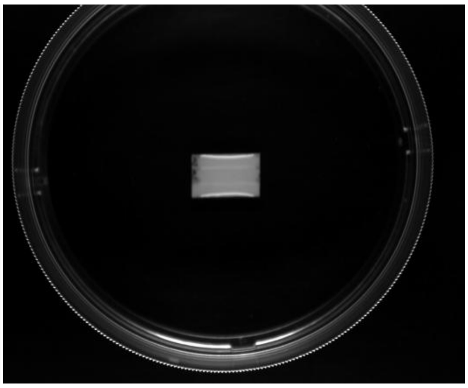

Fig. 5N Third Repetition

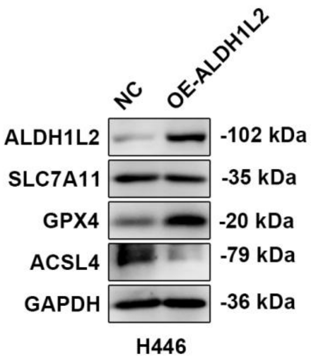

H446

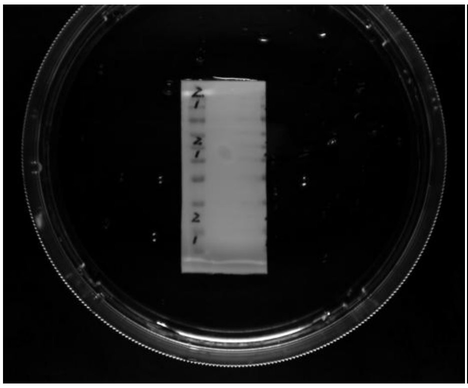

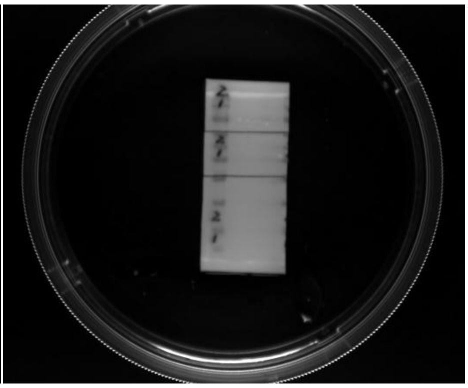

ALDH1L2

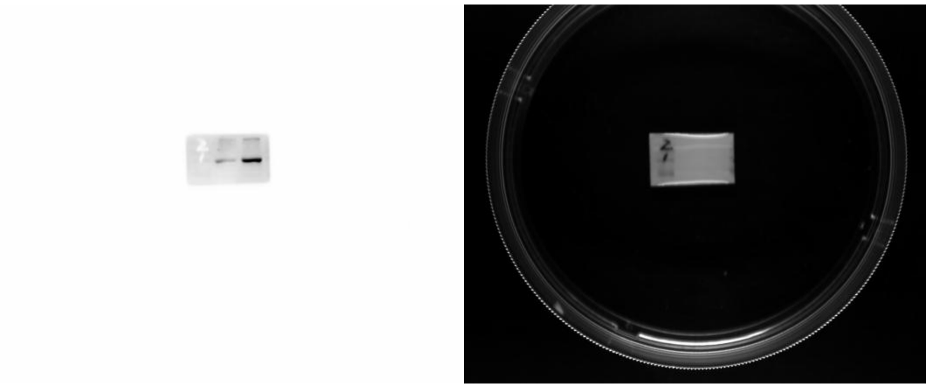

SLC7A11

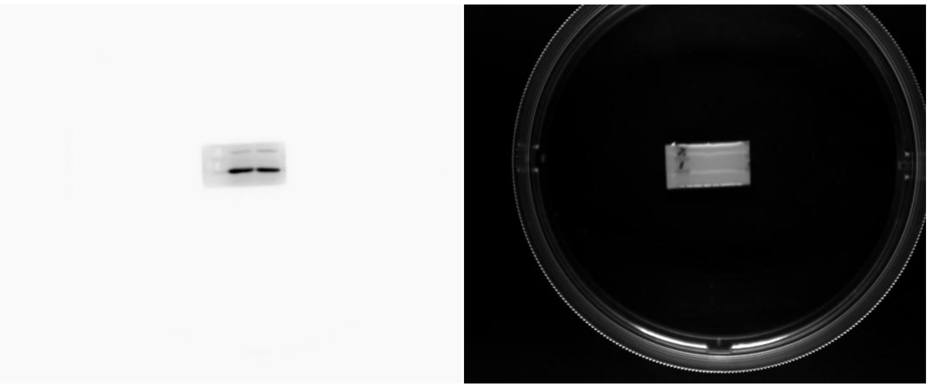

GPX4

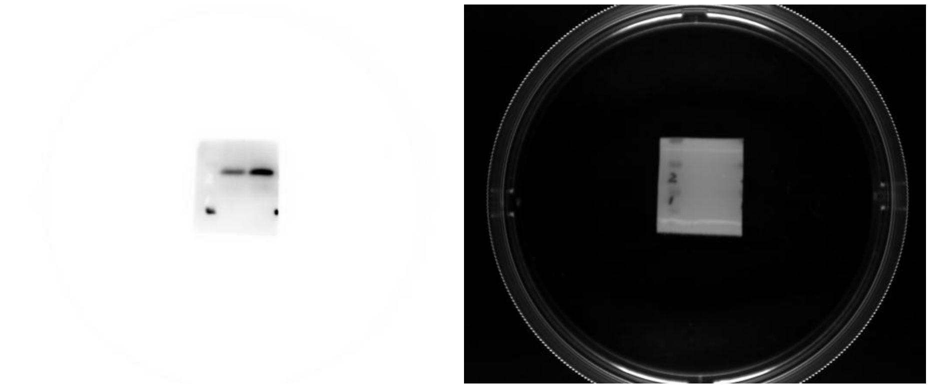

ACSL4

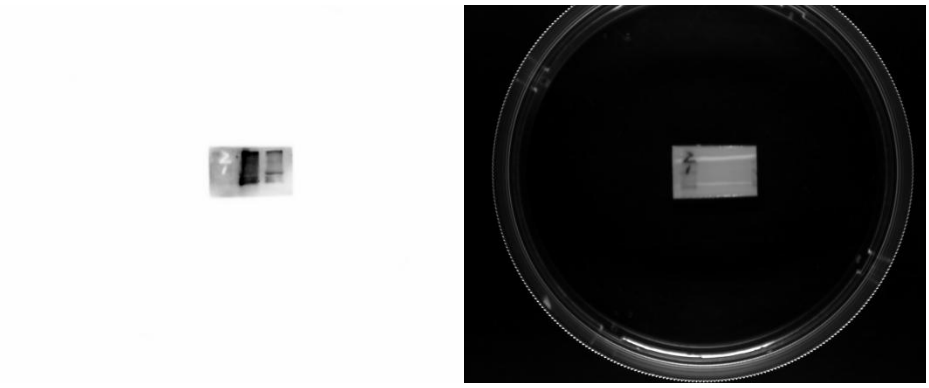

GAPDH

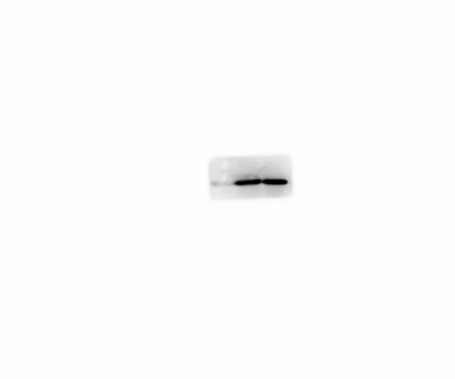

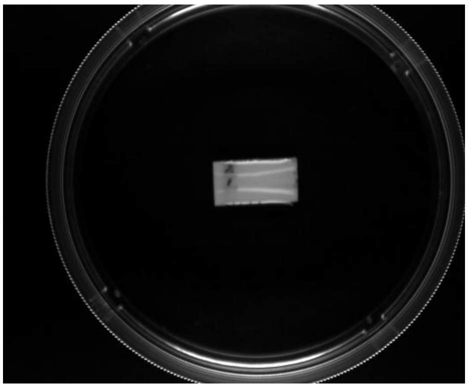

Fig. 5O First Repetition

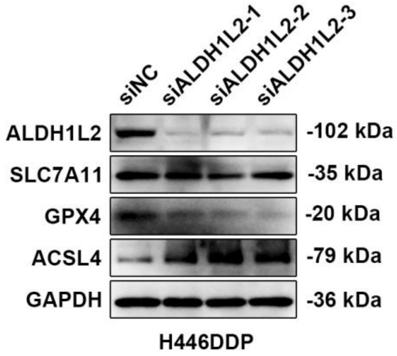

H446DDP

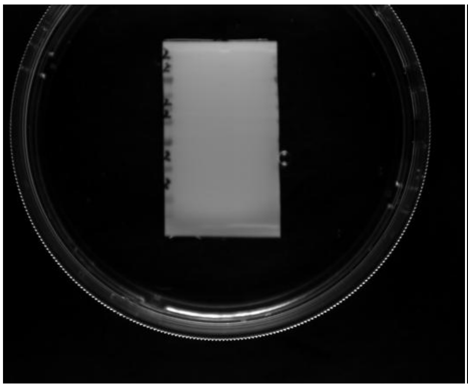

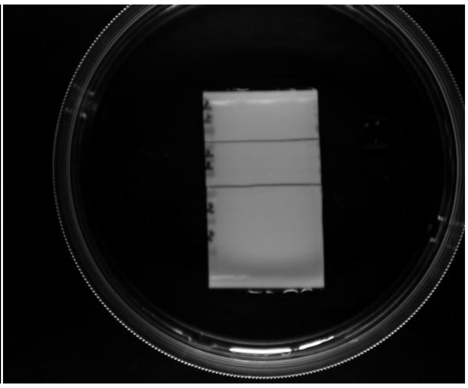

ALDH1L2

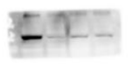

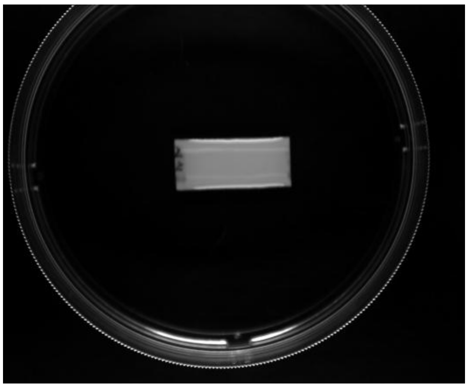

SLC7A11

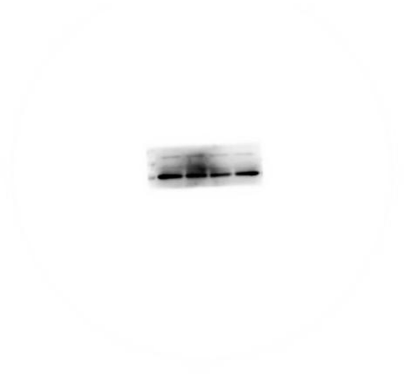

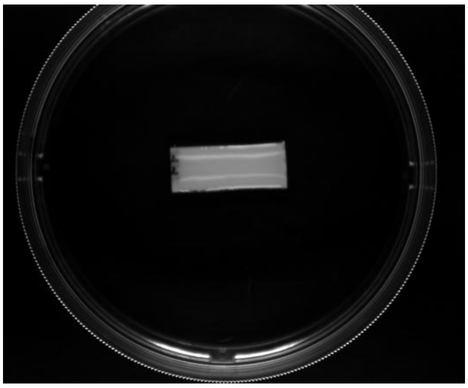

GPX4

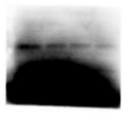

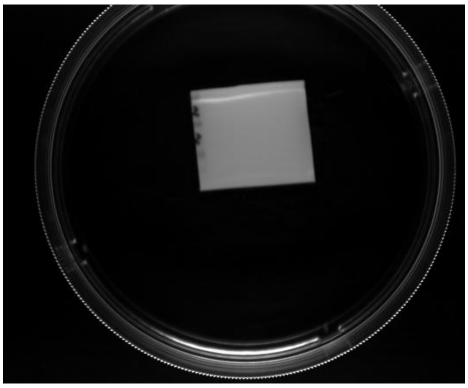

ACSL4

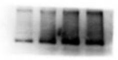

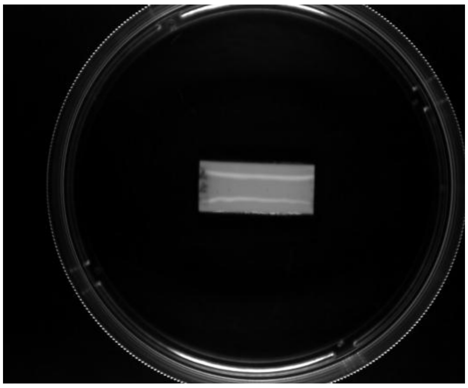

GAPDH

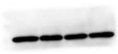

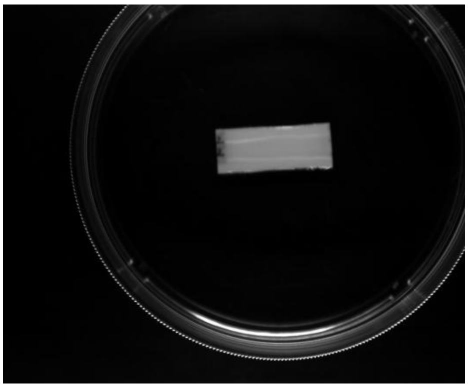

Fig. 5O Second Repetition

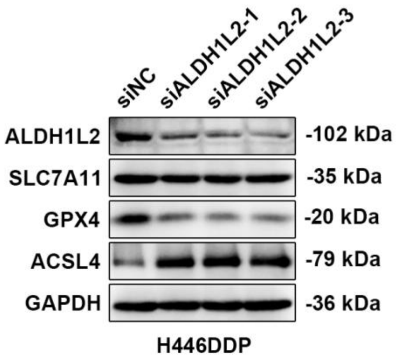

H446DDP

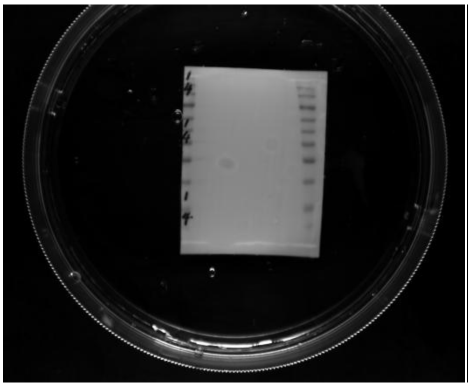

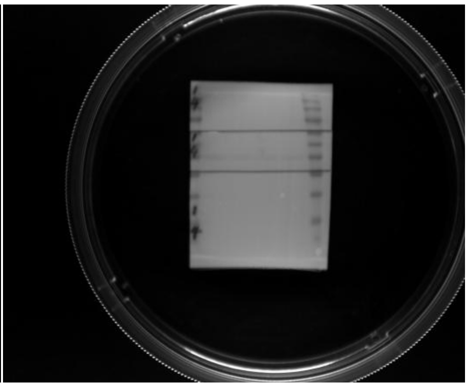

ALDH1L2

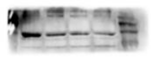

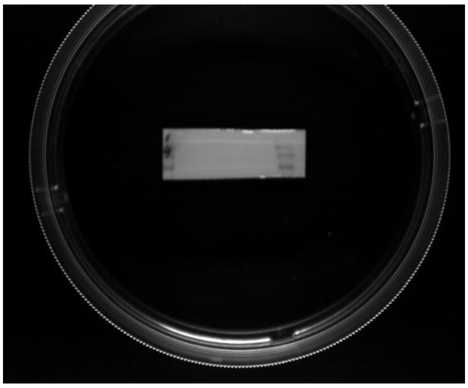

SLC7A11

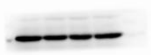

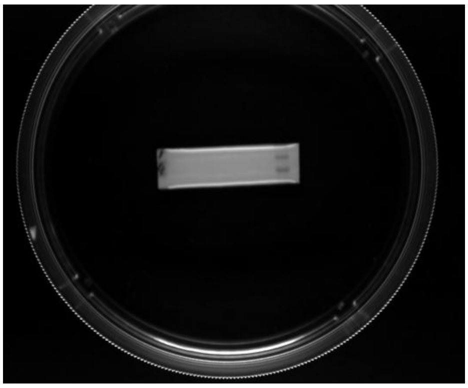

GPX4

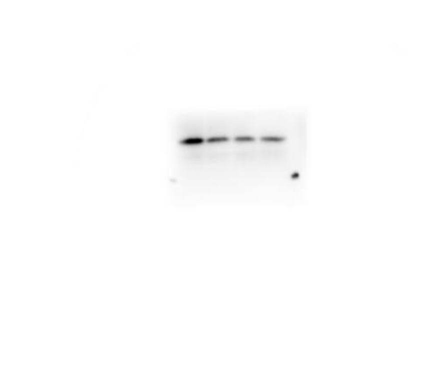

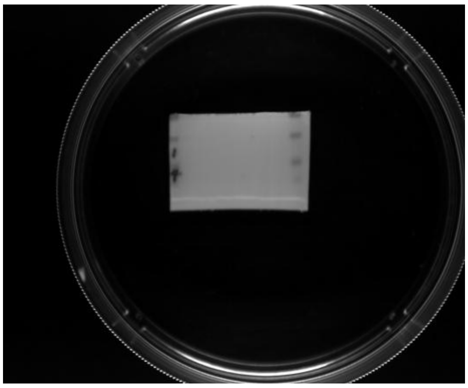

ACSL4

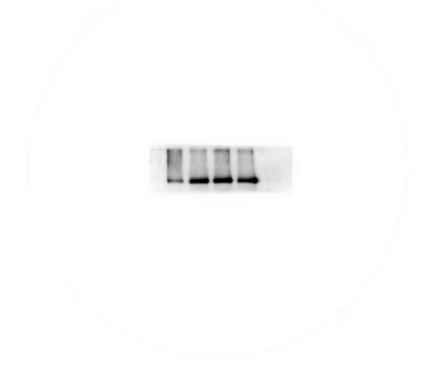

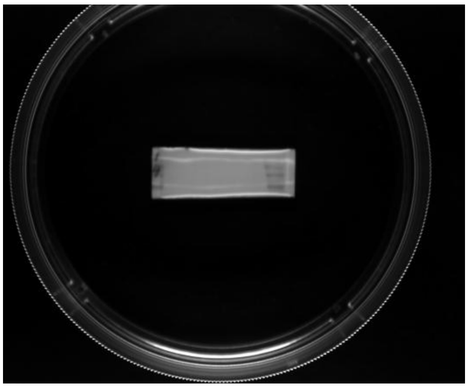

GAPDH

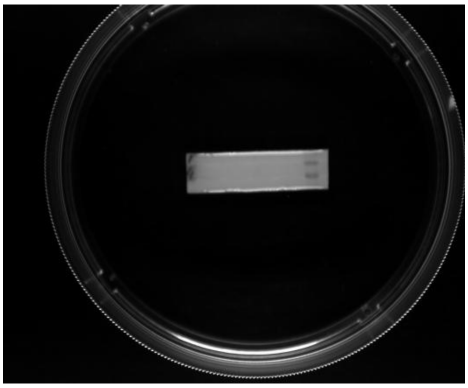

Fig. 5O Third Repetition

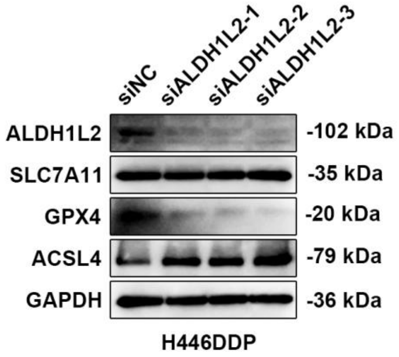

H446DDP

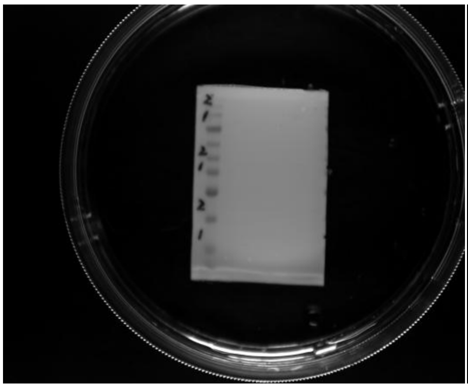

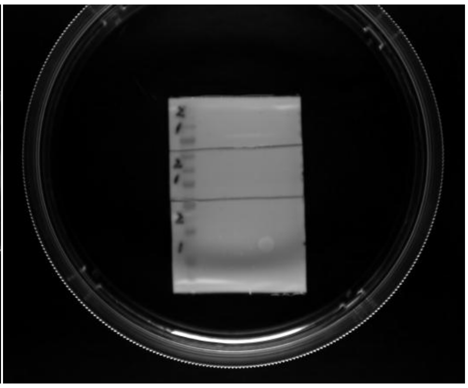

ALDH1L2

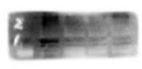

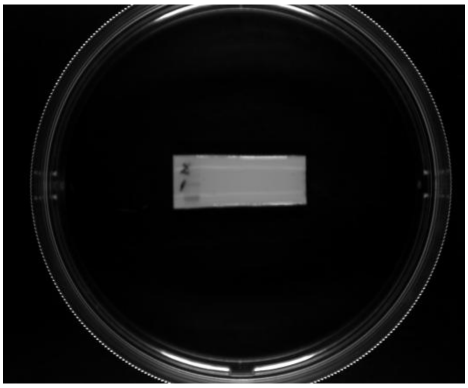

SLC7A11

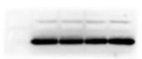

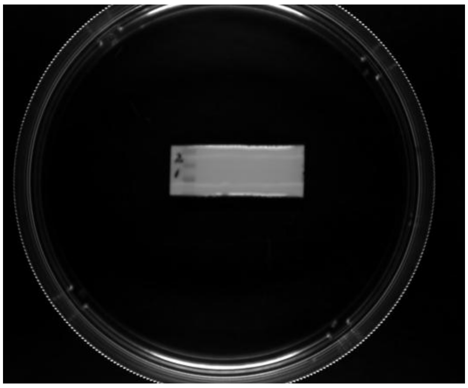

GPX4

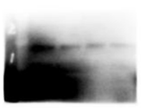

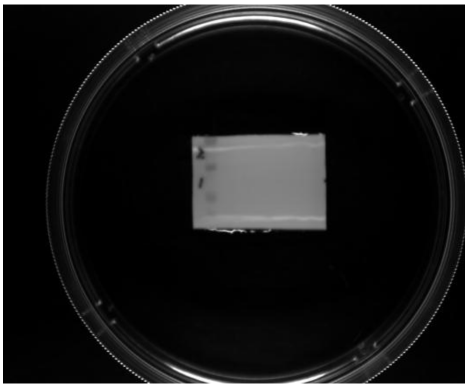

ACSL4

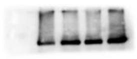

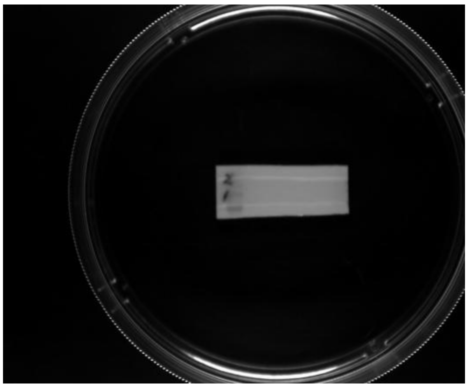

GAPDH

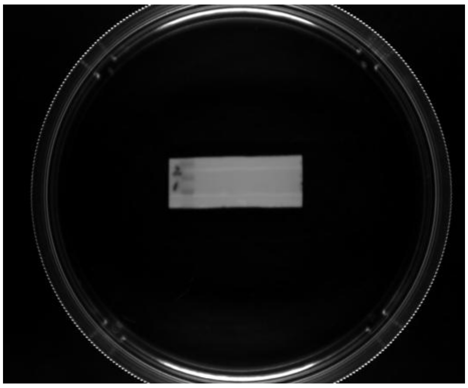

Fig. 6K First Repetition

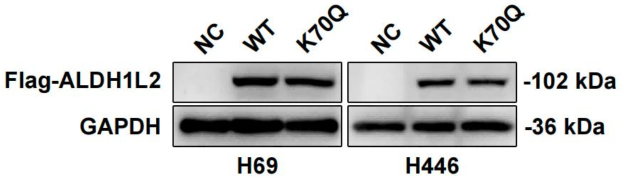

H69

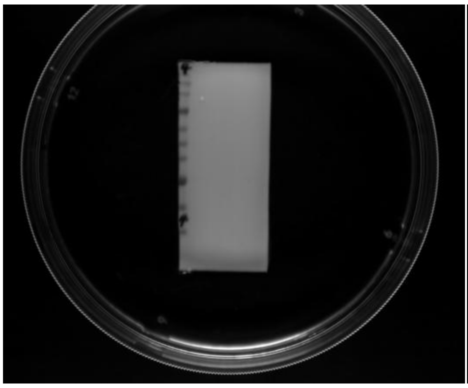

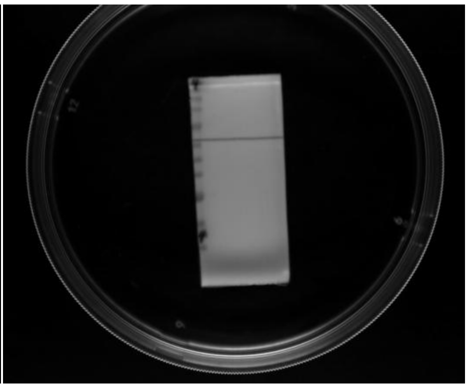

Flag-ALDH1L2

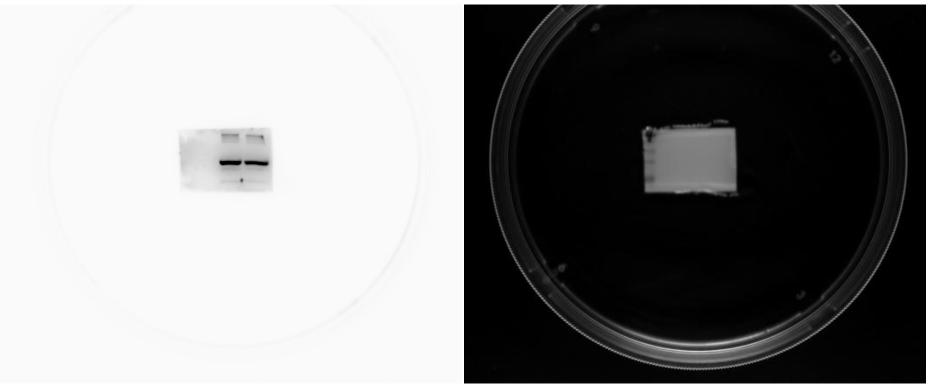

GAPDH

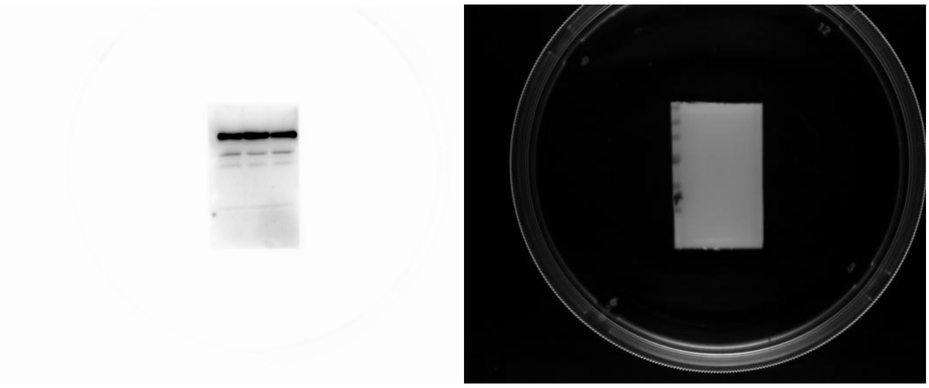

H446

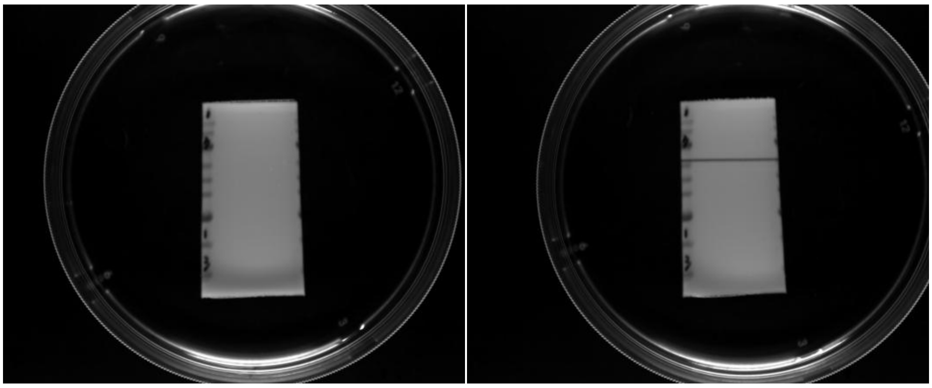

Flag-ALDH1L2

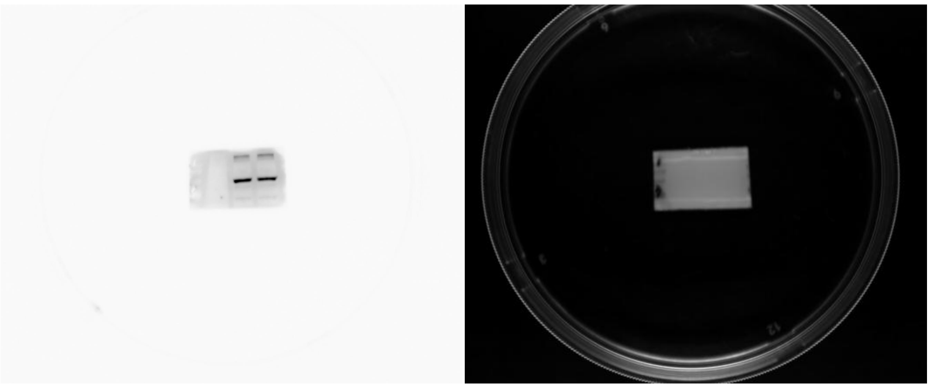

GAPDH

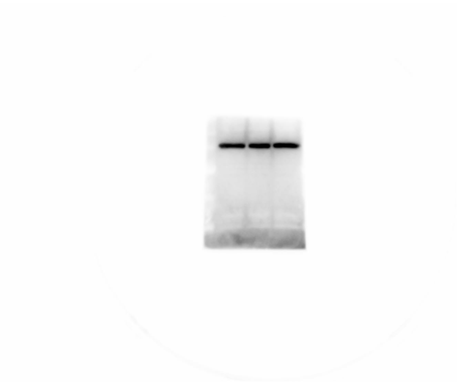

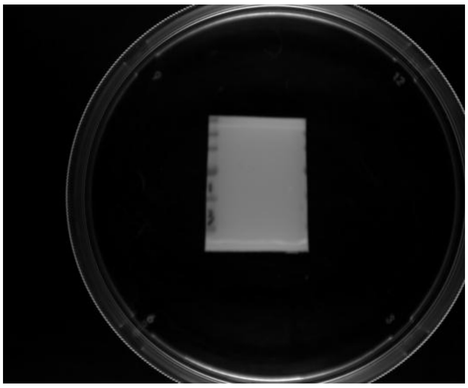

Fig. 6K Second Repetition

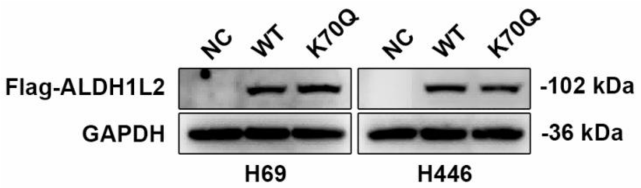

H69

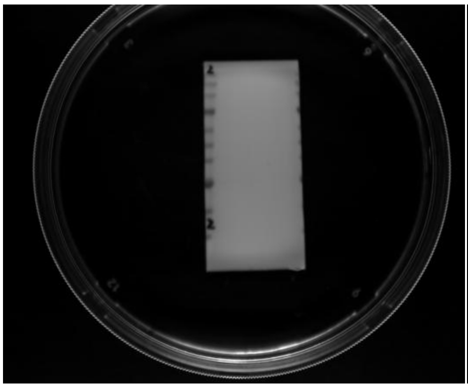

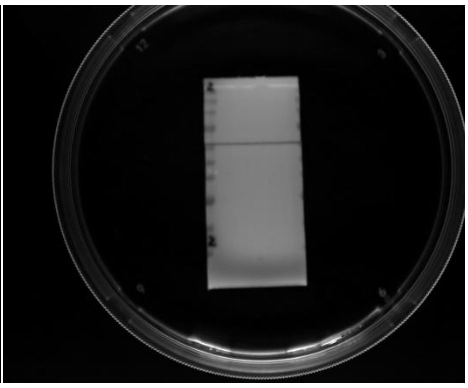

Flag-ALDH1L2

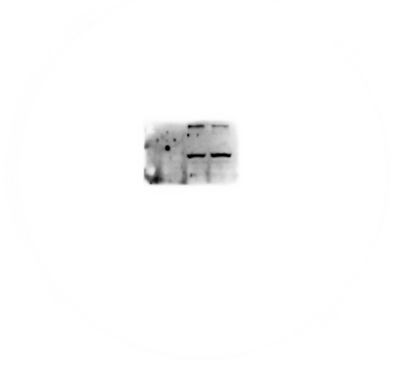

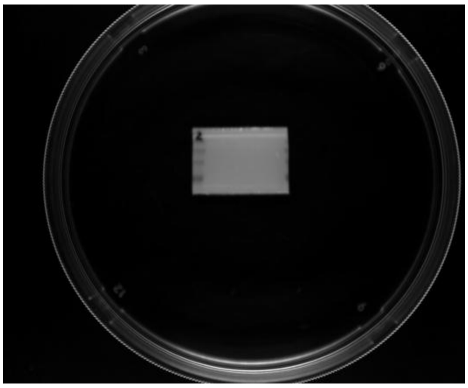

GAPDH

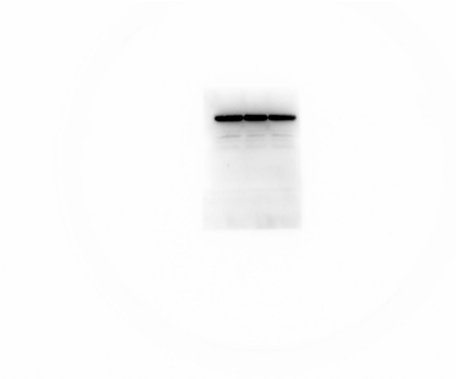
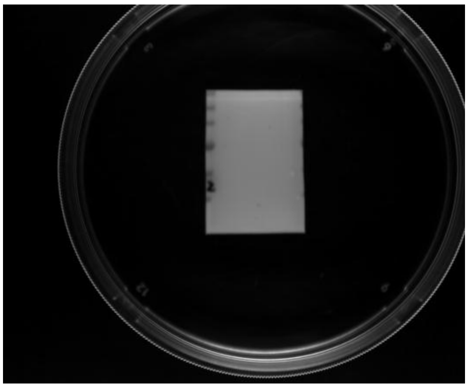

H446

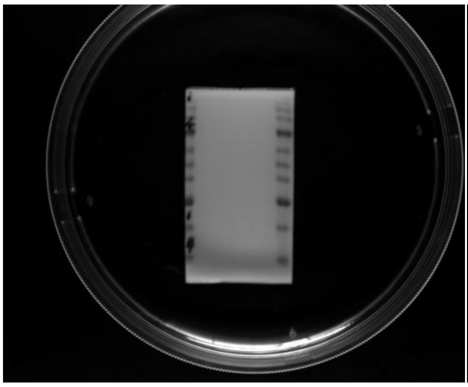
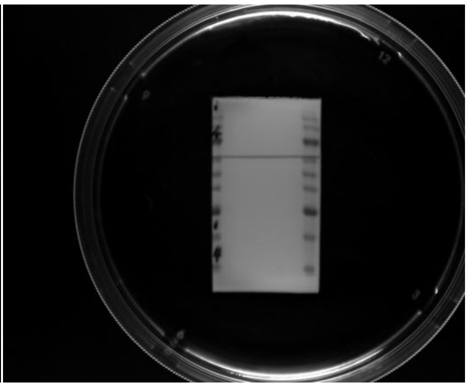

Flag-ALDH1L2

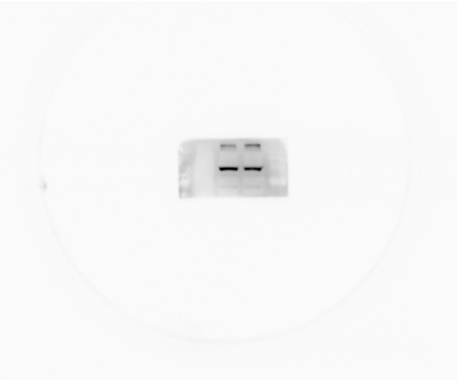
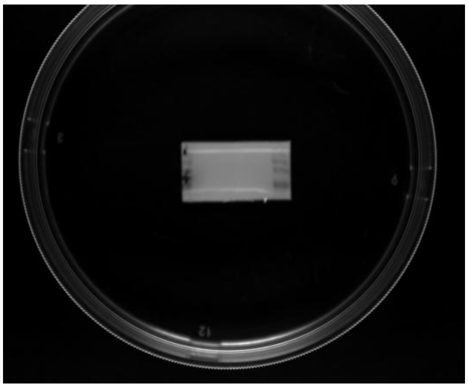

GAPDH

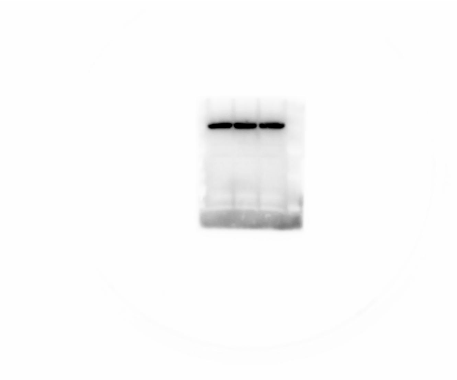
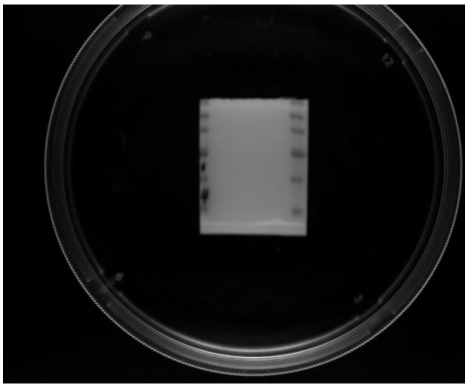

Fig. 6K Third Repetition

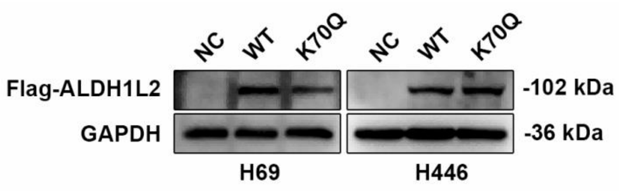

H69

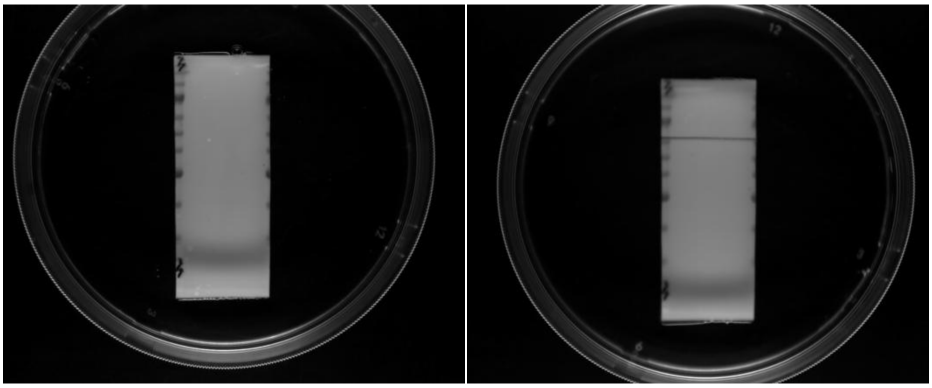

Flag-ALDH1L2

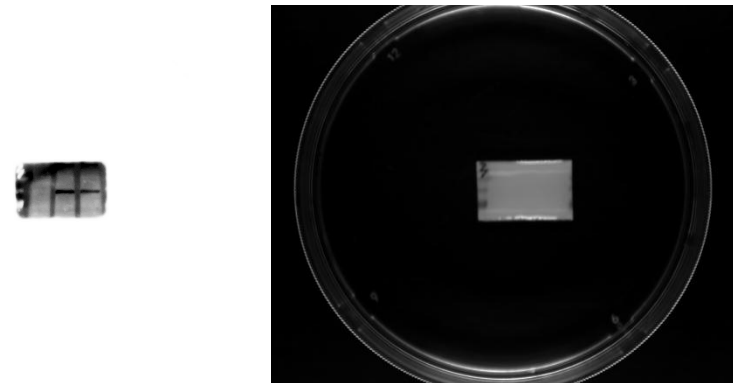

GAPDH

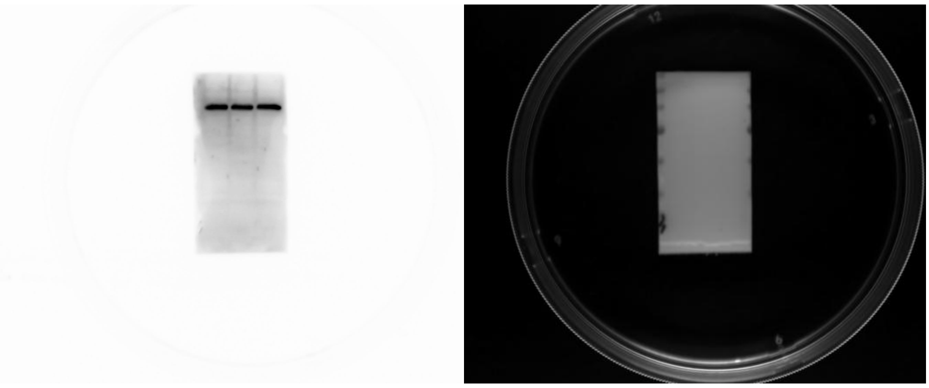

H446

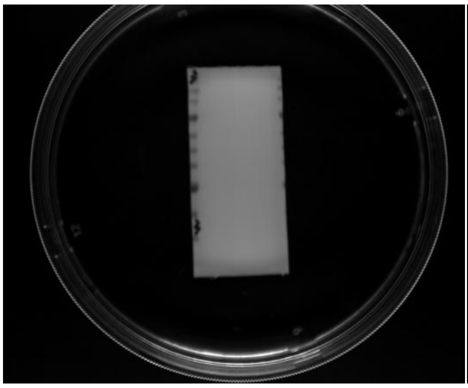

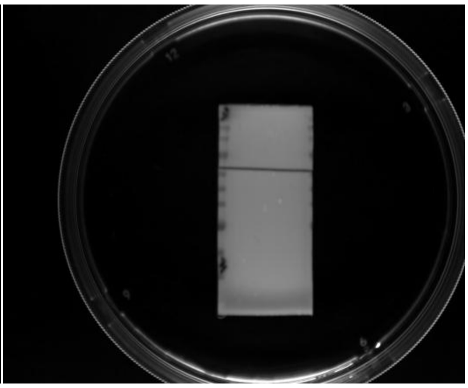

Flag-ALDH1L2

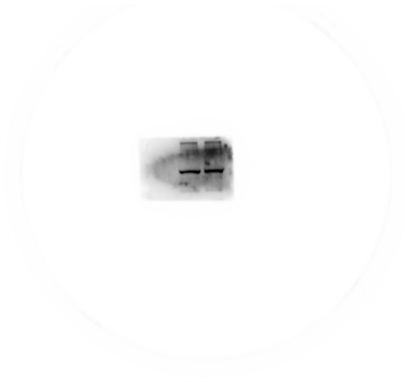

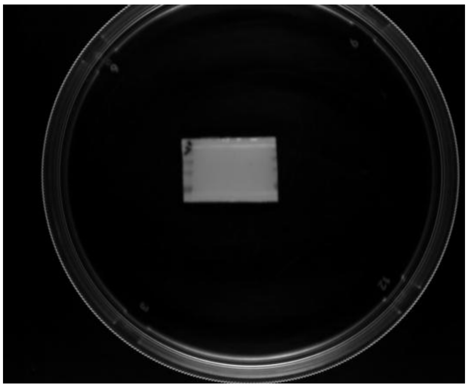

GAPDH

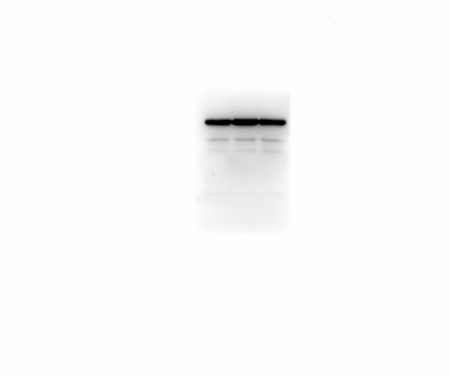

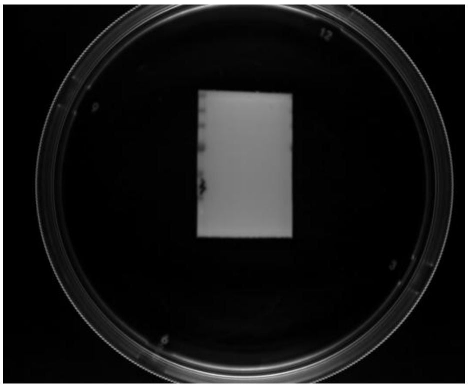

Fig. 6R First Repetition

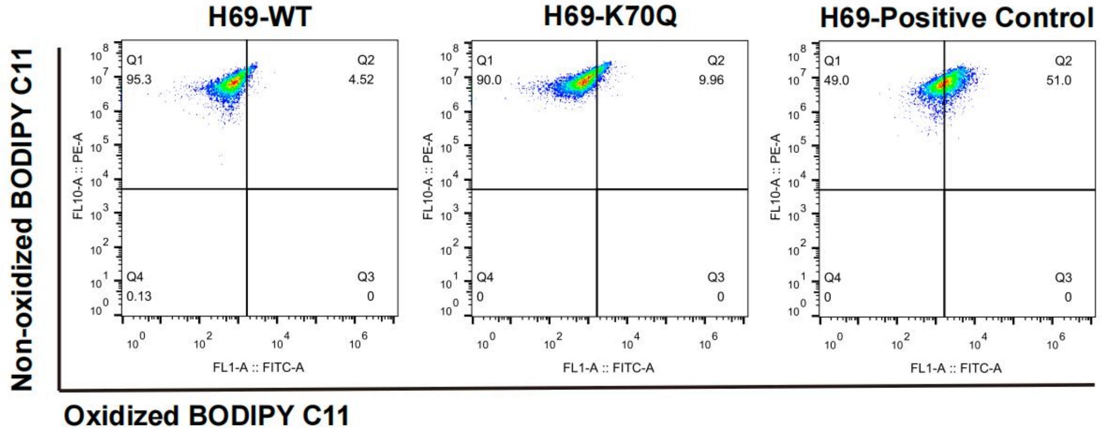

Fig. 6R Second Repetition

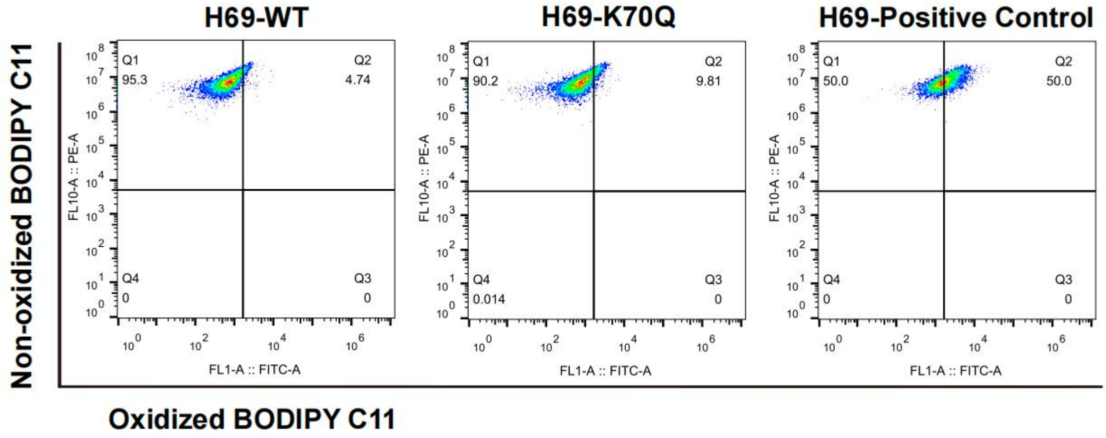

Fig. 6R Third Repetition

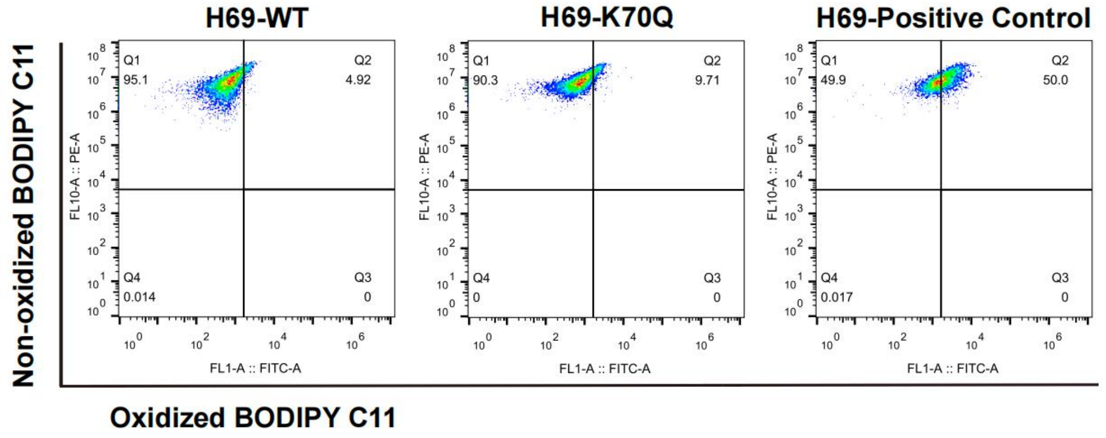

Fig. 6S First Repetition

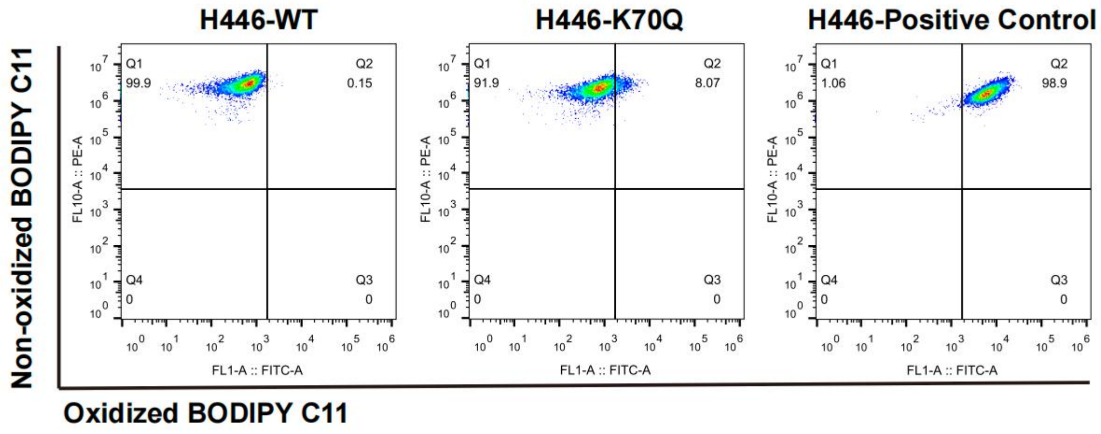

Fig. 6S Second Repetition

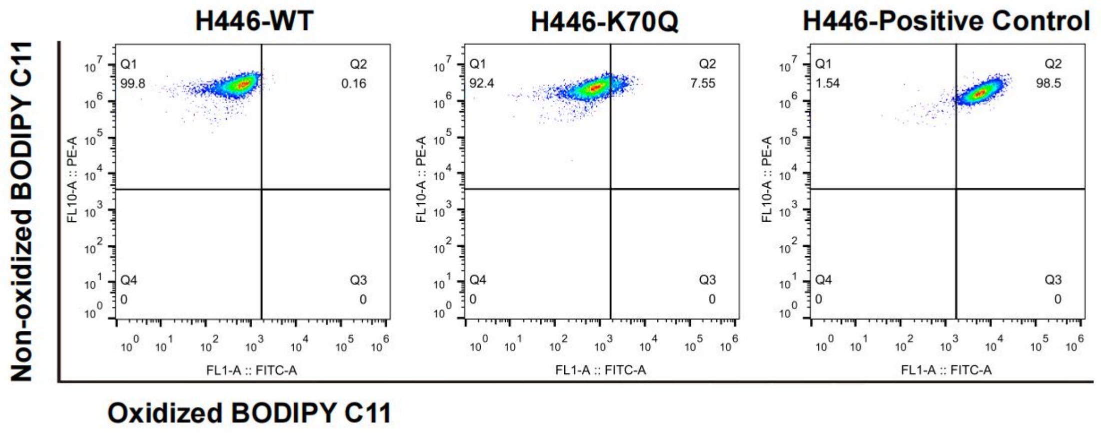

Fig. 6S Third Repetition

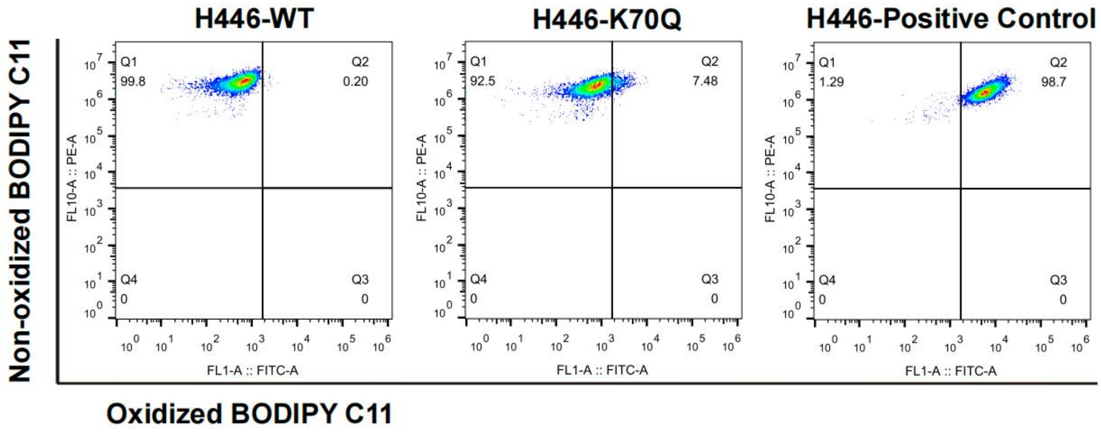

Fig. 7A First Repetition

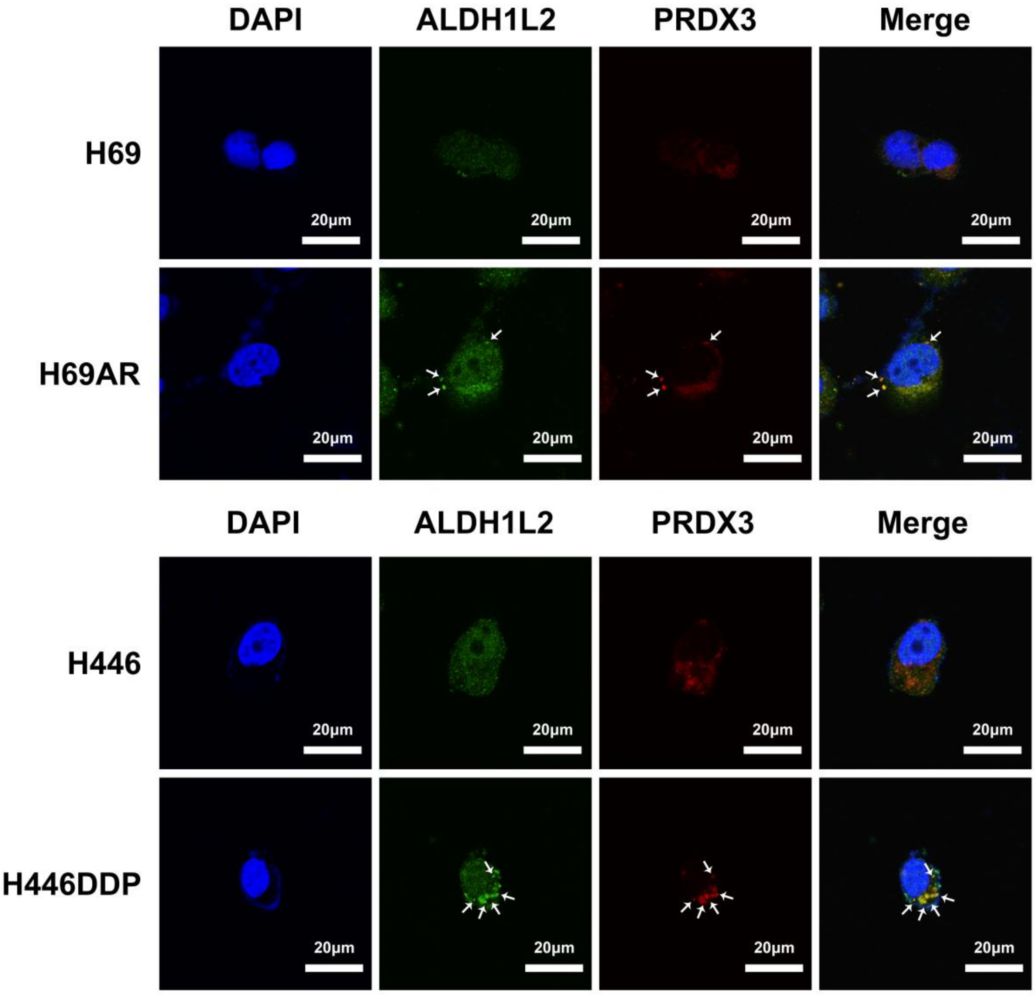

Fig. 7A Second Repetition

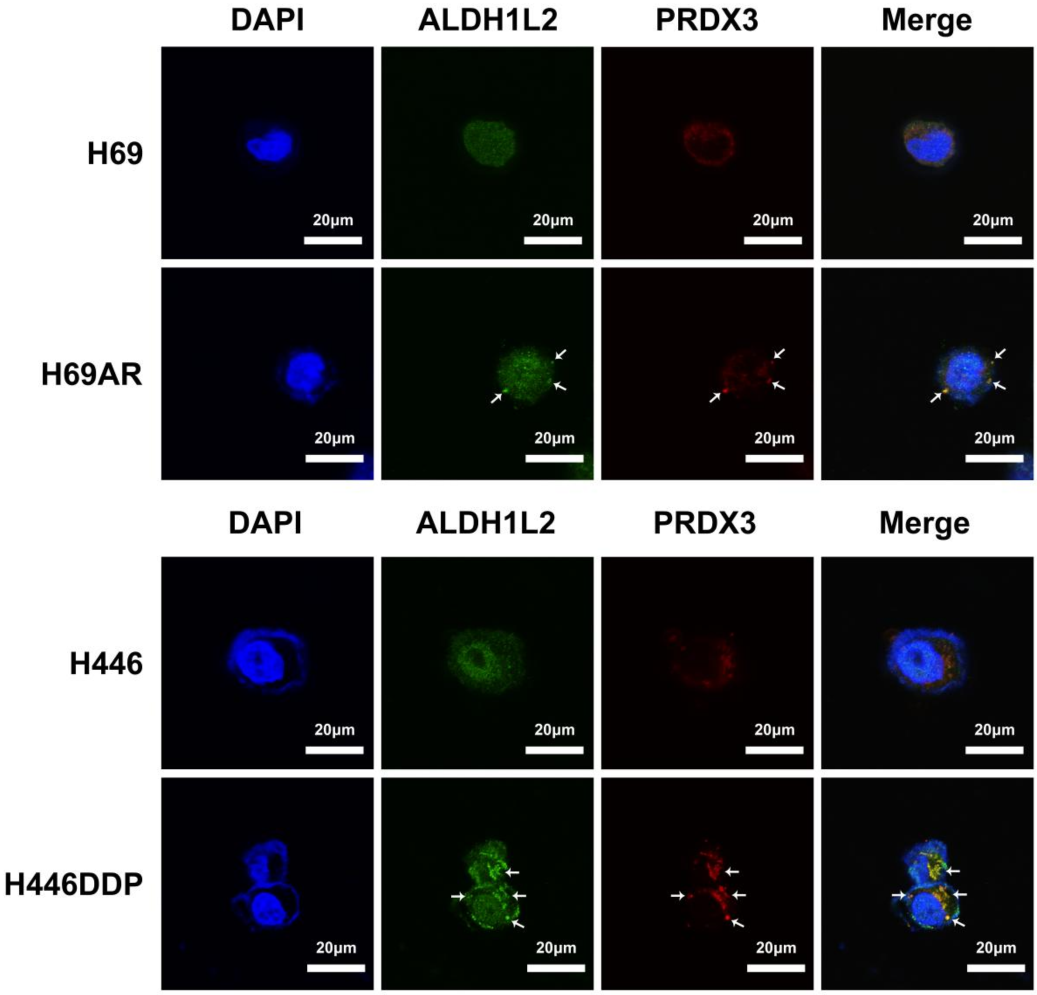

Fig. 7A Third Repetition

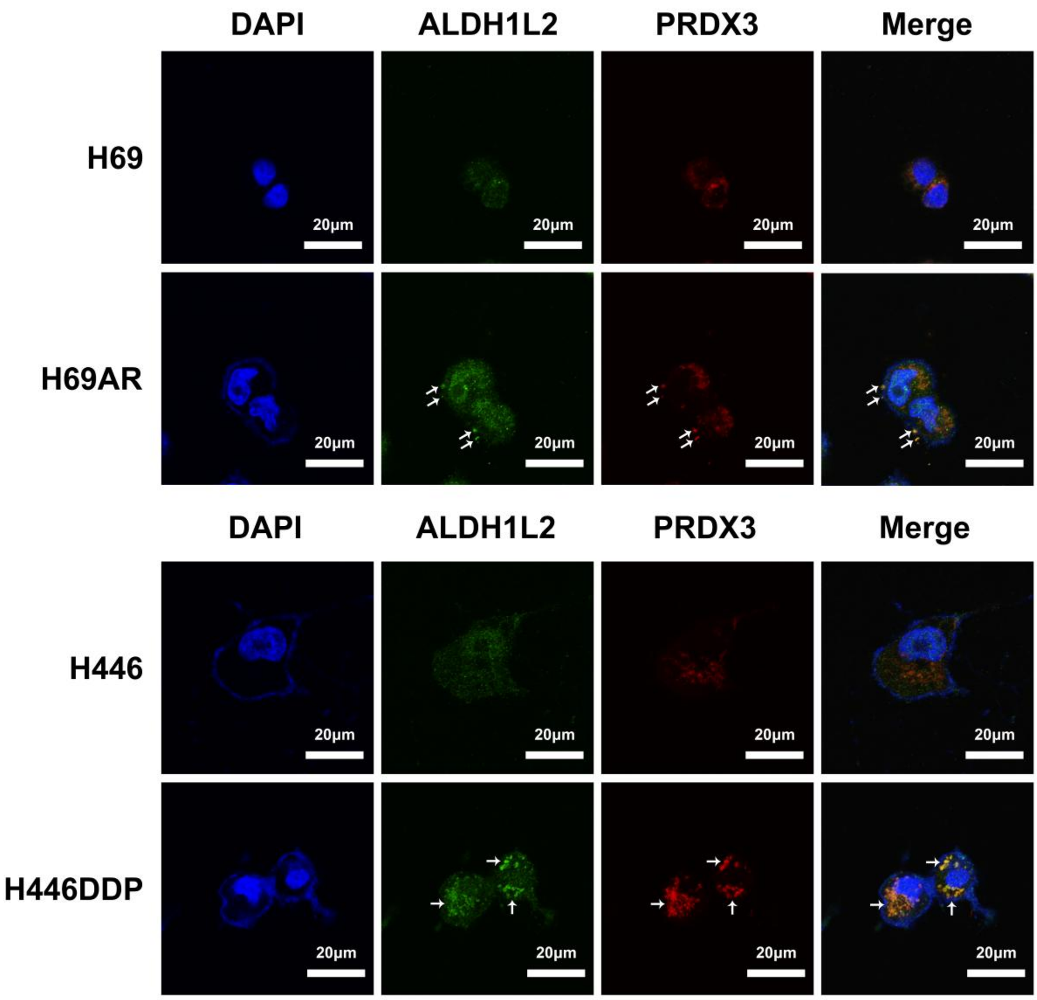

Fig. 7B First Repetition

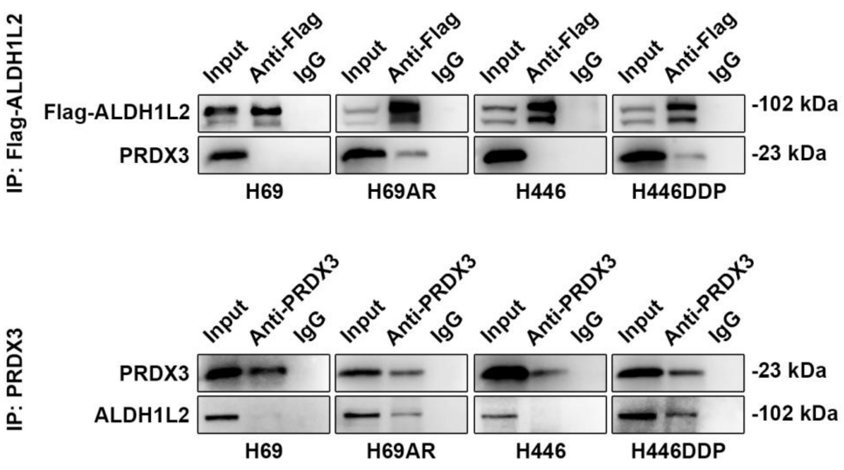

H69

Flag-ALDH1L2 (IP: Flag-ALDH1L2)

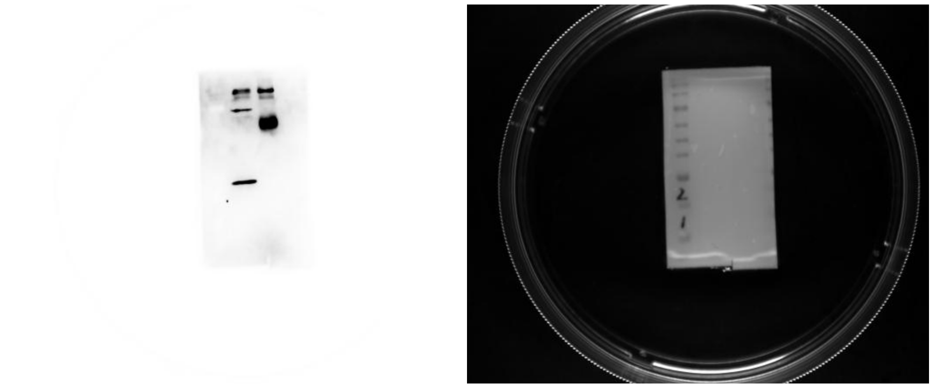

PRDX3 (IP: Flag-ALDH1L2)

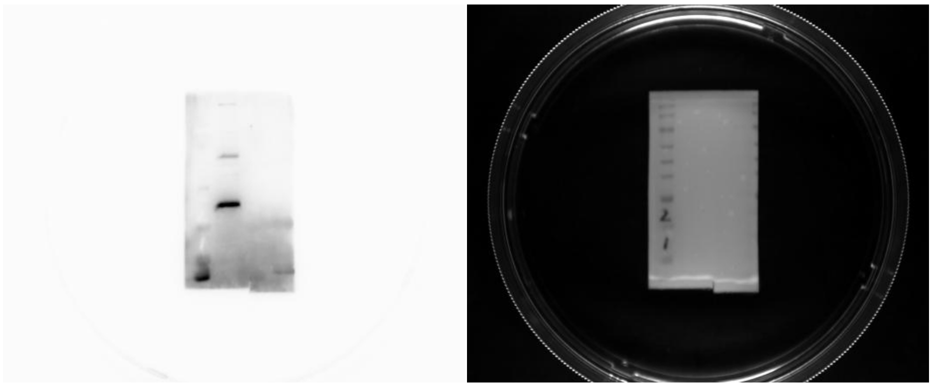

PRDX3 (IP: PRDX3)

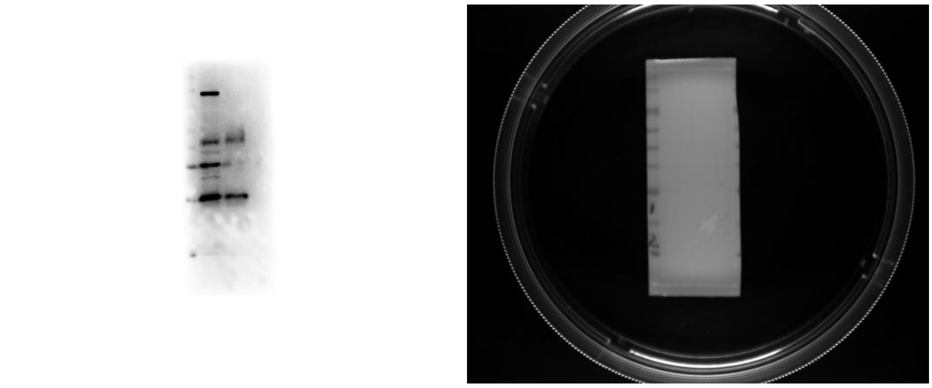

ALDH1L2 (IP: PRDX3)

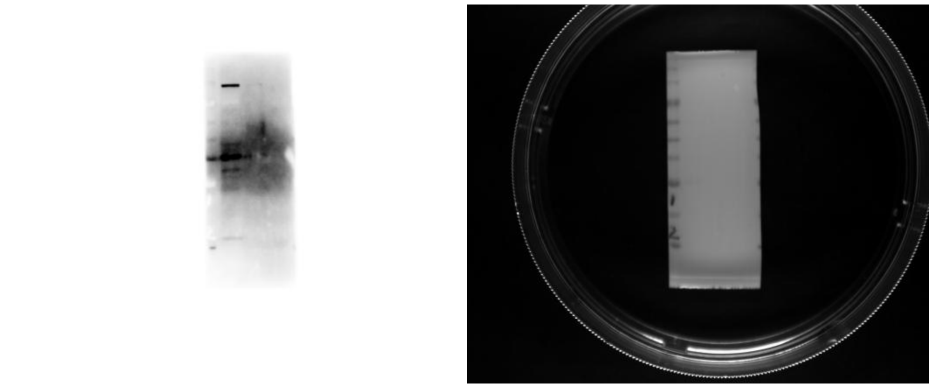

H69AR

Flag-ALDH1L2 (IP: Flag-ALDH1L2)

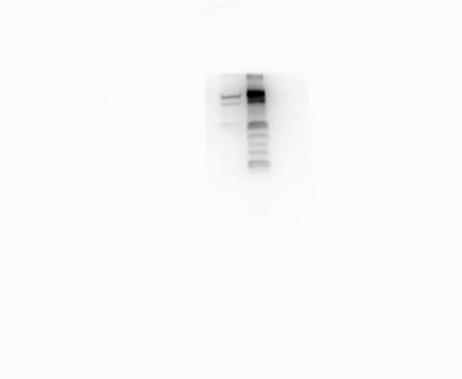
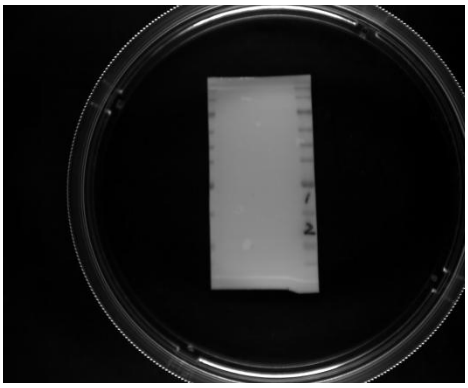

PRDX3 (IP: Flag-ALDH1L2)

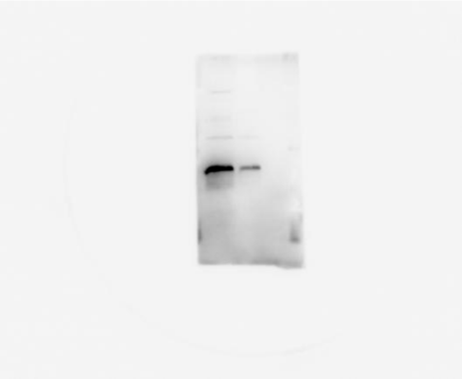
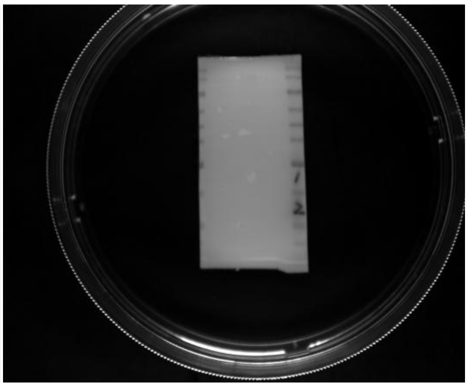

PRDX3 (IP: PRDX3)

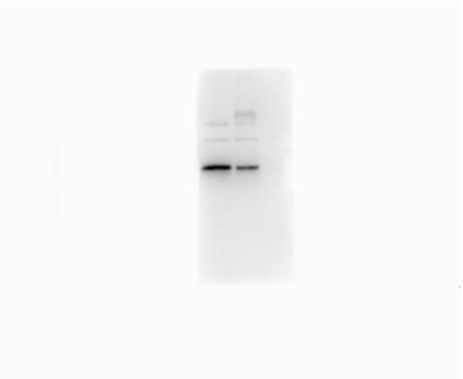
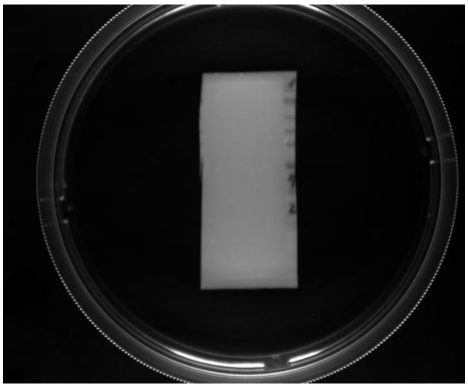

ALDH1L2 (IP: PRDX3)

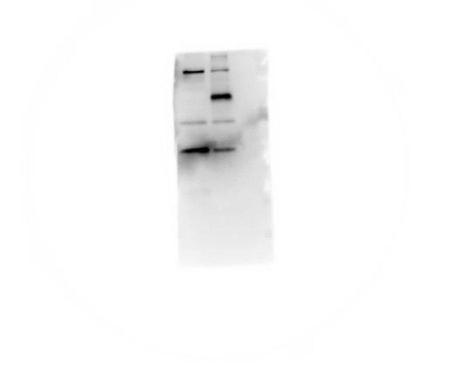
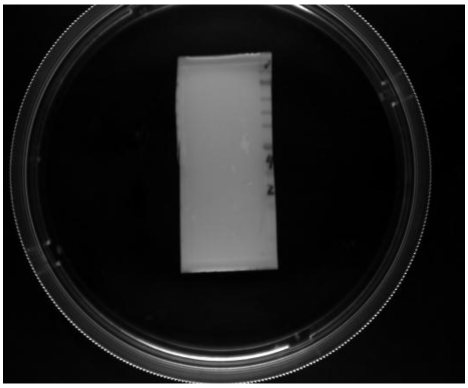

H446

Flag-ALDH1L2 (IP: Flag-ALDH1L2)

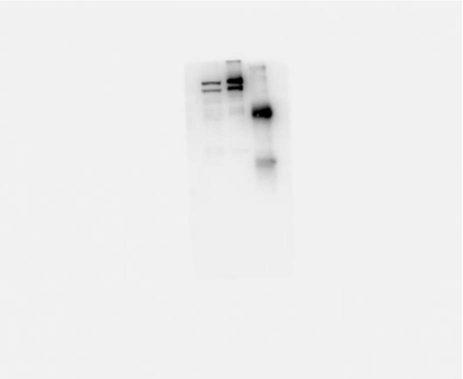

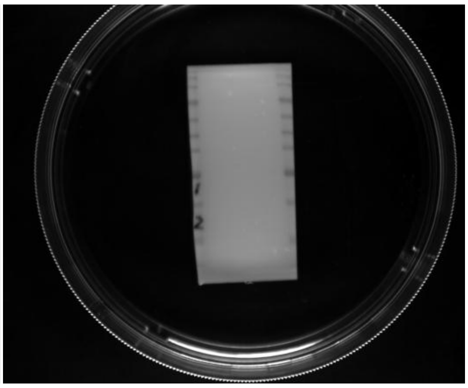

PRDX3 (IP: Flag-ALDH1L2)

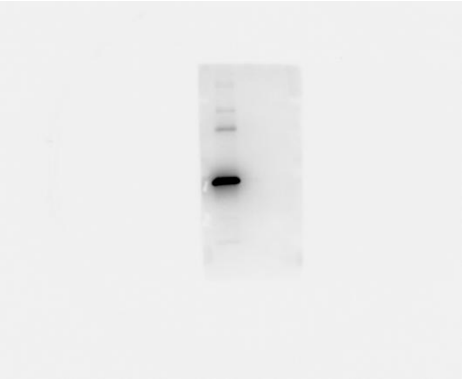

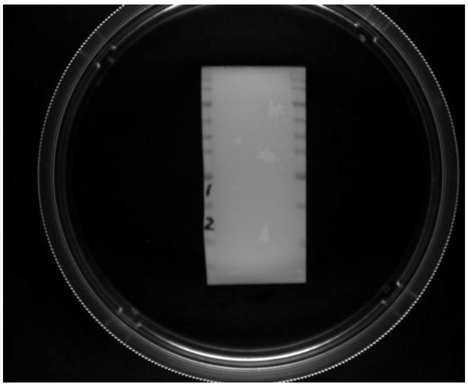

PRDX3 (IP: PRDX3)

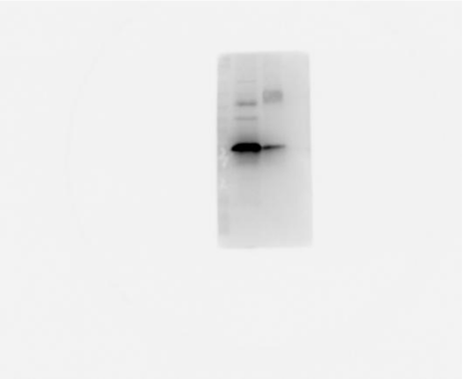

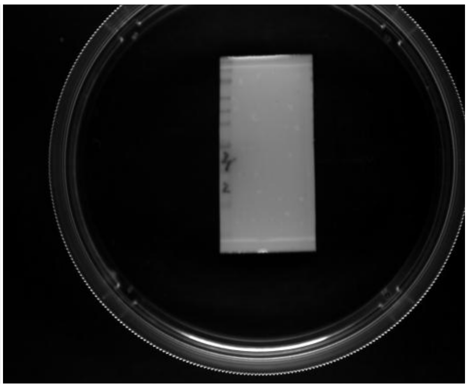

ALDH1L2 (IP: PRDX3)

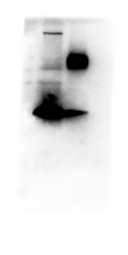

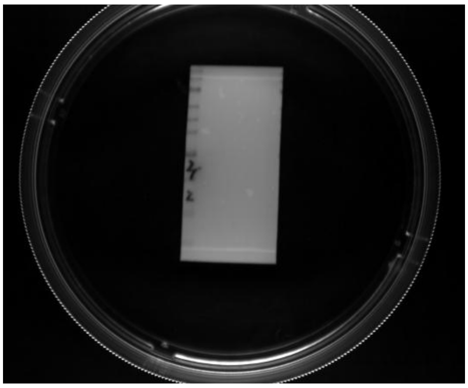

H446DDP

Flag-ALDH1L2 (IP: Flag-ALDH1L2)

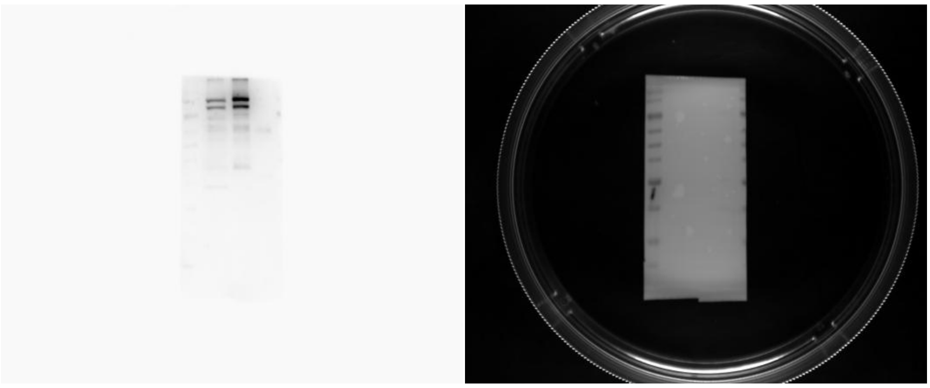

PRDX3 (IP: Flag-ALDH1L2)

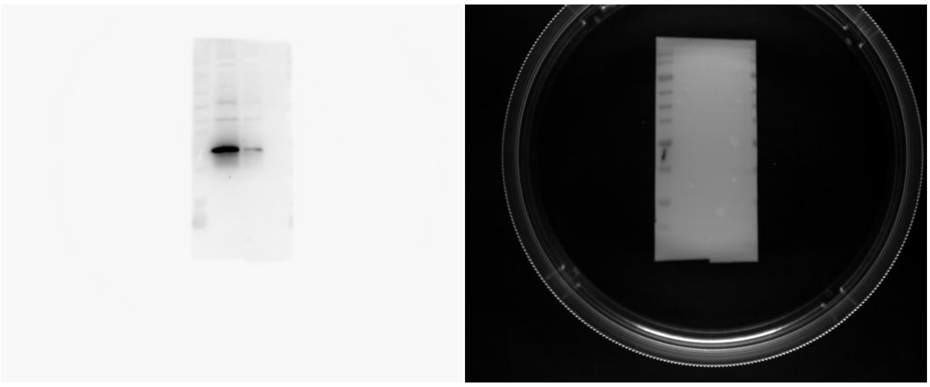

PRDX3 (IP: PRDX3)

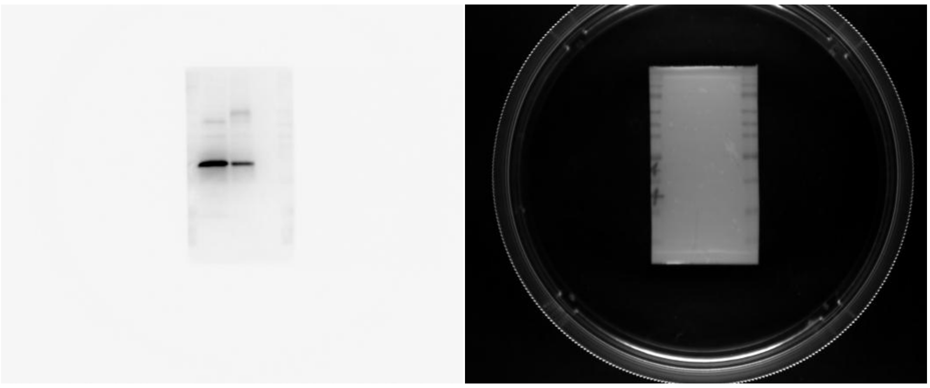

ALDH1L2 (IP: PRDX3)

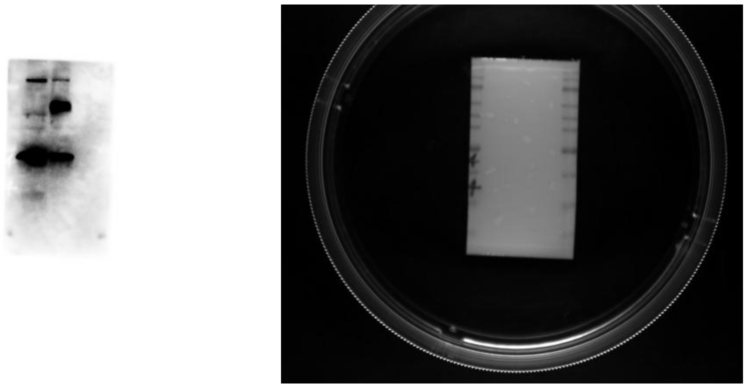

Fig. 7B Second Repetition

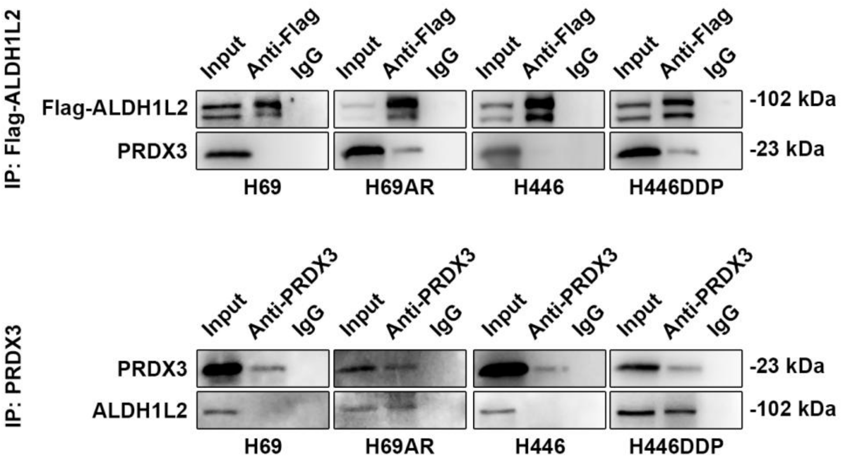

H69

Flag-ALDH1L2 (IP: Flag-ALDH1L2)

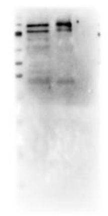

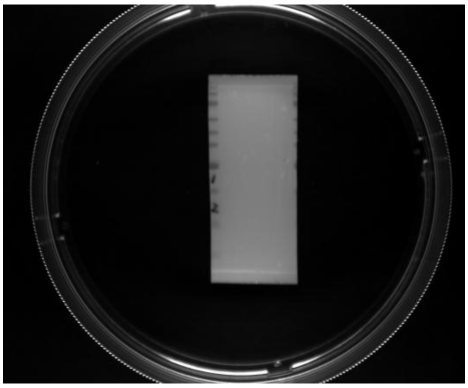

PRDX3 (IP: Flag-ALDH1L2)

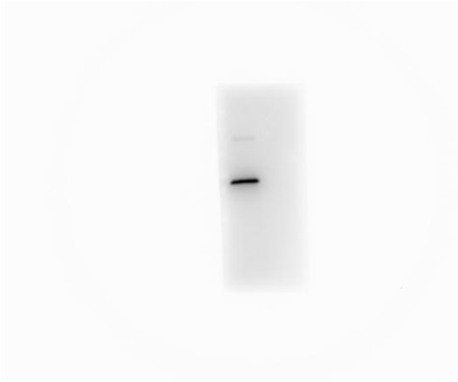

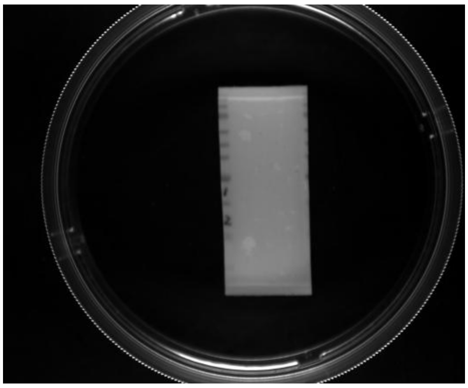

PRDX3 (IP: PRDX3)

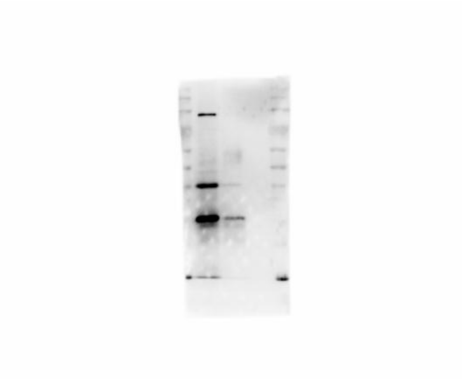

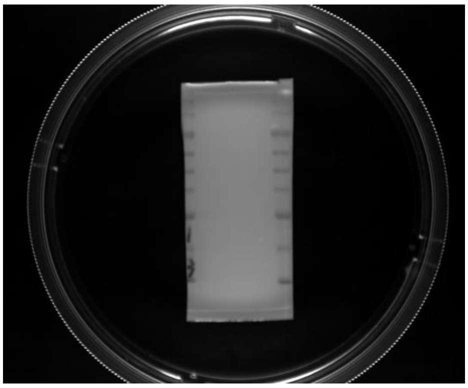

ALDH1L2 (IP: PRDX3)

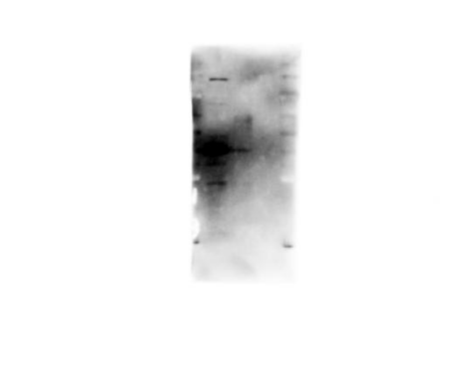

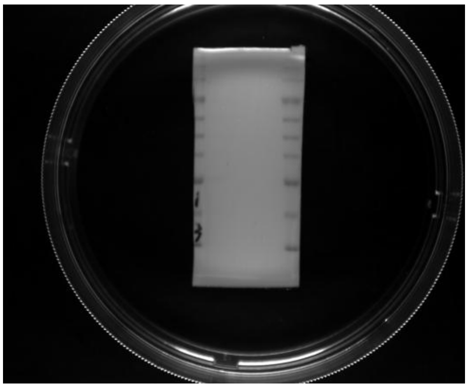

H69AR

Flag-ALDH1L2 (IP: Flag-ALDH1L2)

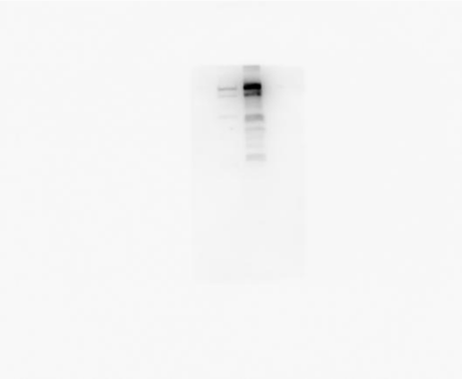

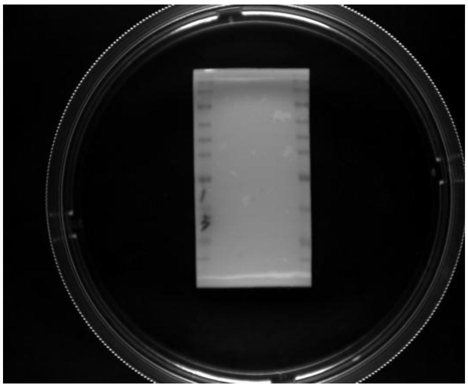

PRDX3 (IP: Flag-ALDH1L2)

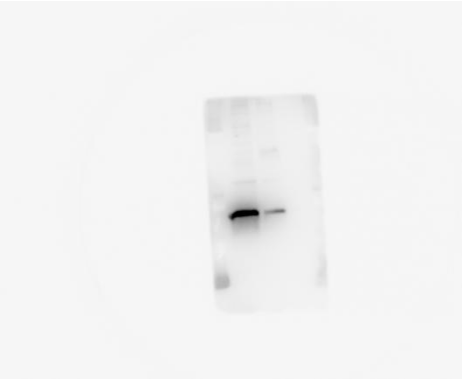

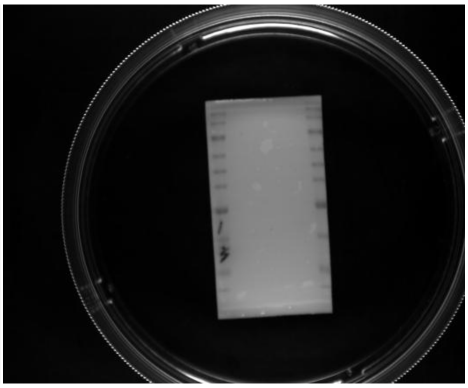

PRDX3 (IP: PRDX3)

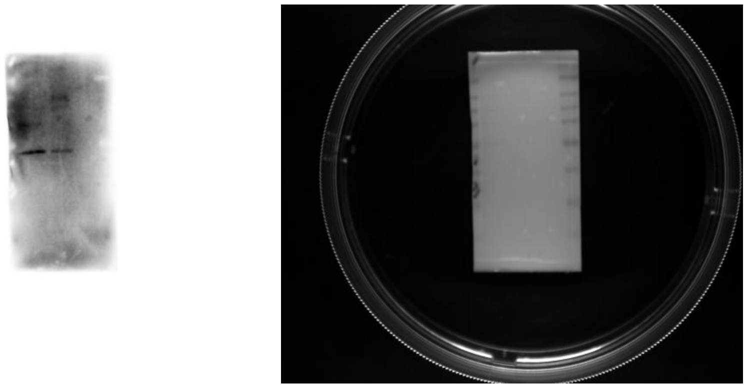

ALDH1L2 (IP: PRDX3)

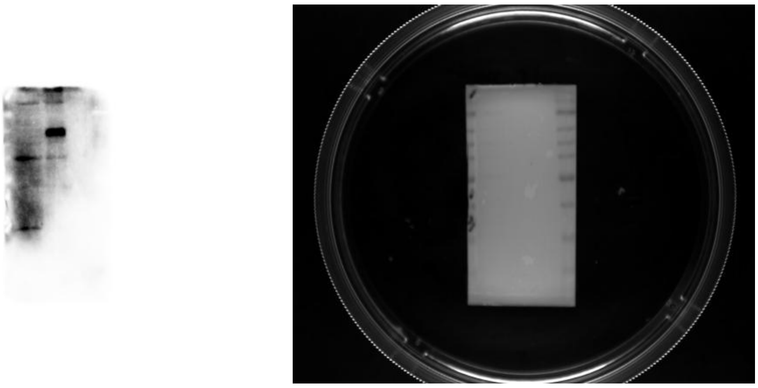

H446

Flag-ALDH1L2 (IP: Flag-ALDH1L2)

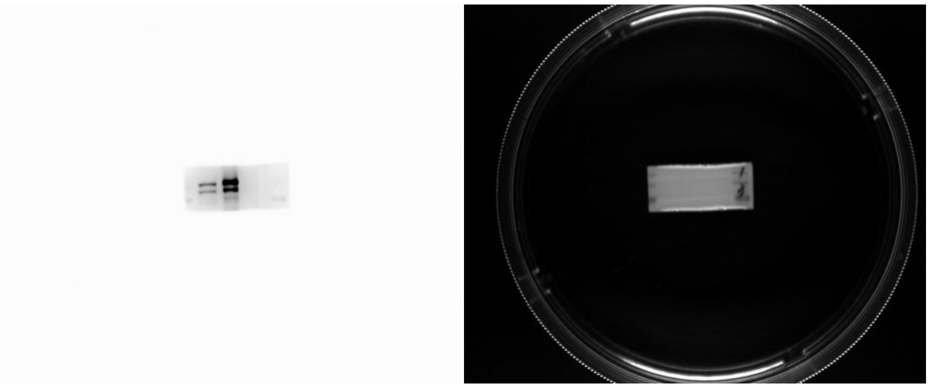

PRDX3 (IP: Flag-ALDH1L2)

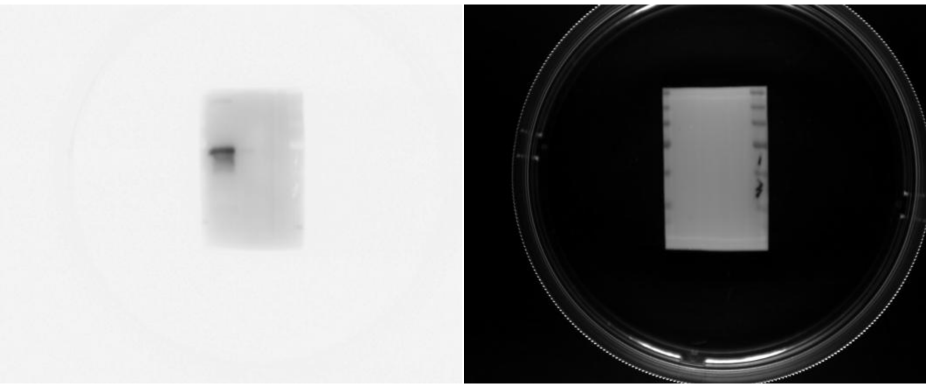

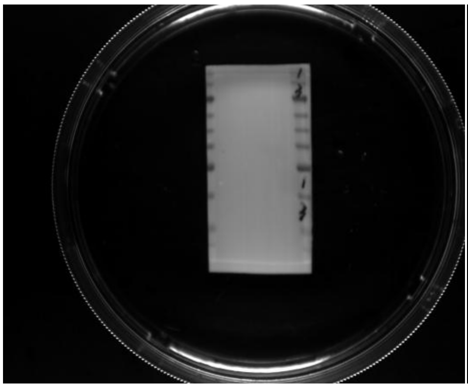
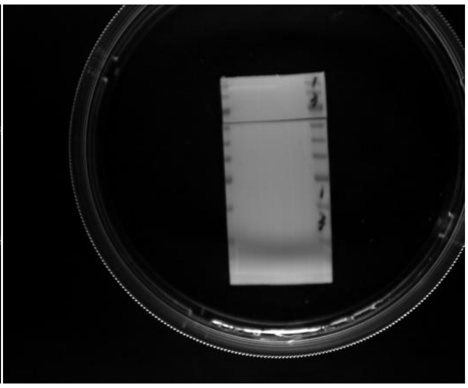

PRDX3 (IP: PRDX3)

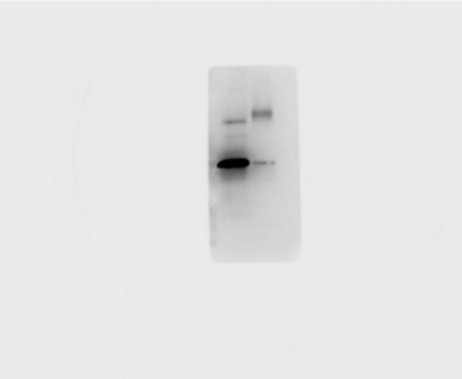
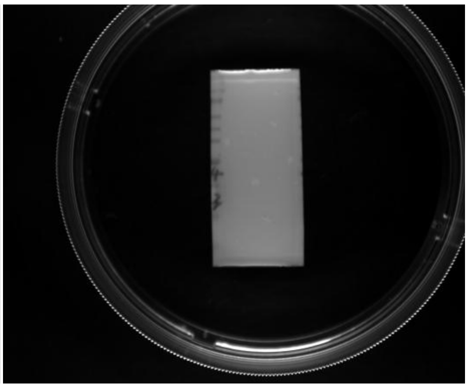

ALDH1L2 (IP: PRDX3)

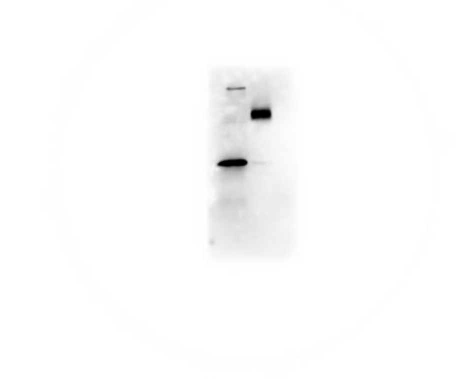
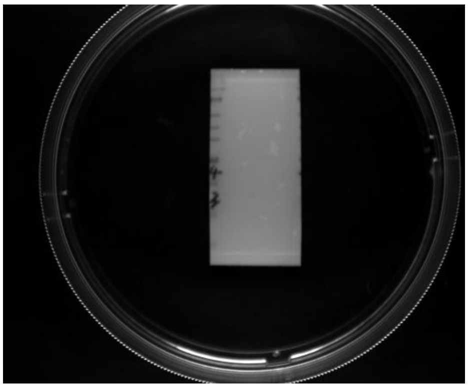

H446DDP

Flag-ALDH1L2 (IP: Flag-ALDH1L2)

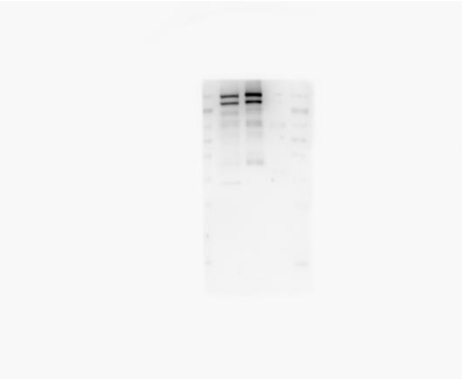
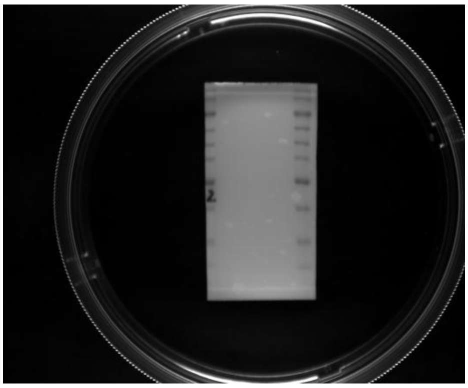

PRDX3 (IP: Flag-ALDH1L2)

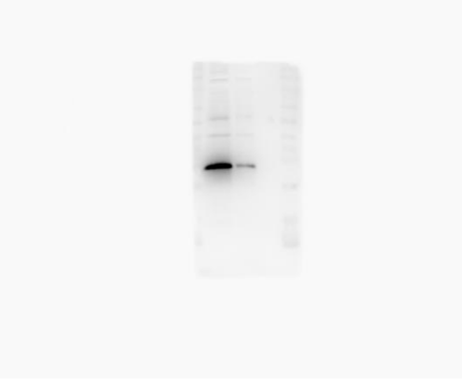

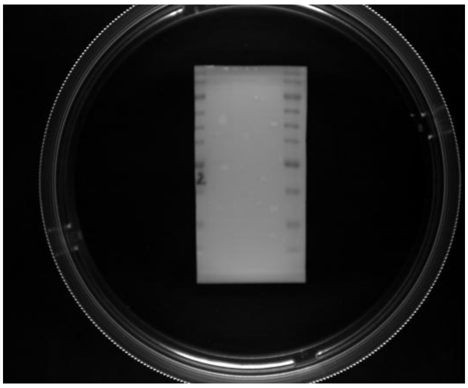

PRDX3 (IP: PRDX3)

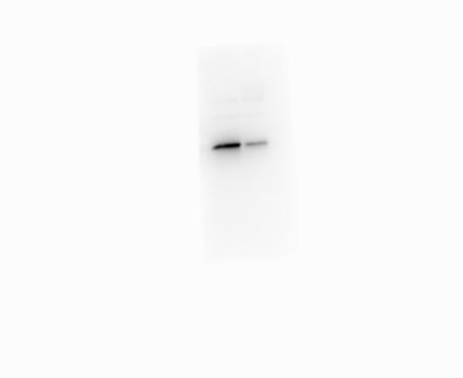

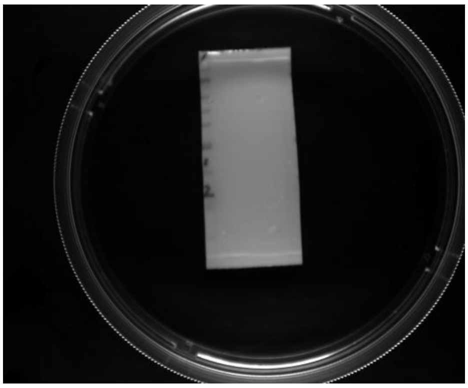

ALDH1L2 (IP: PRDX3)

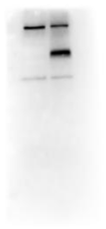

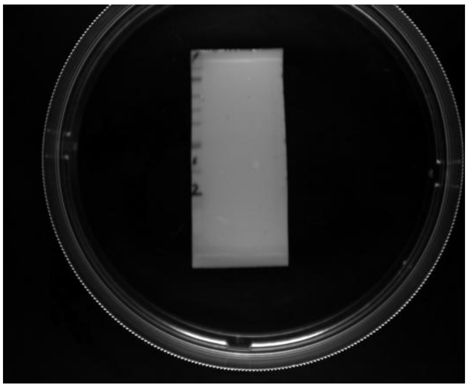

Fig. 7B Third Repetition

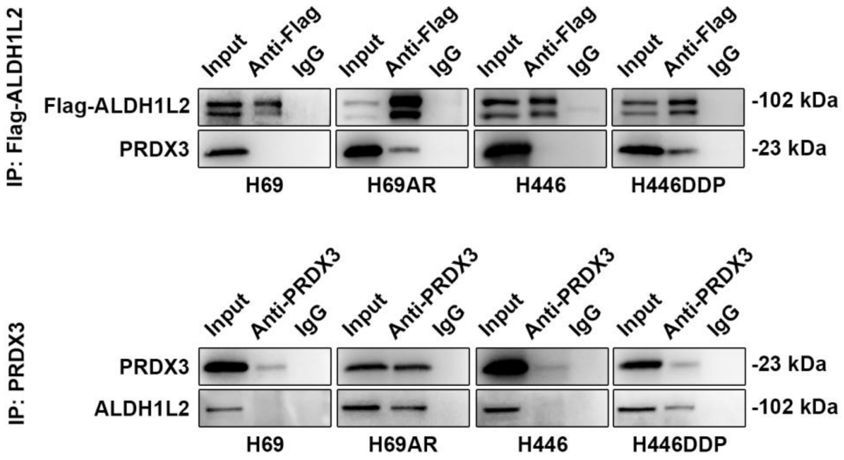

H69

Flag-ALDH1L2 (IP: Flag-ALDH1L2)

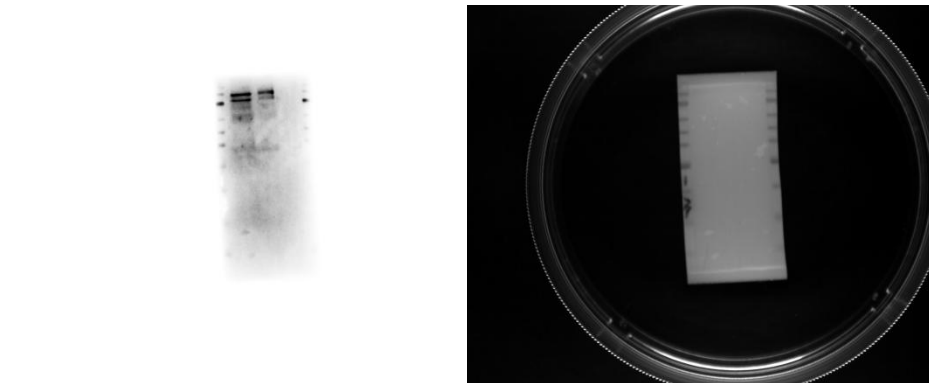

PRDX3 (IP: Flag-ALDH1L2)

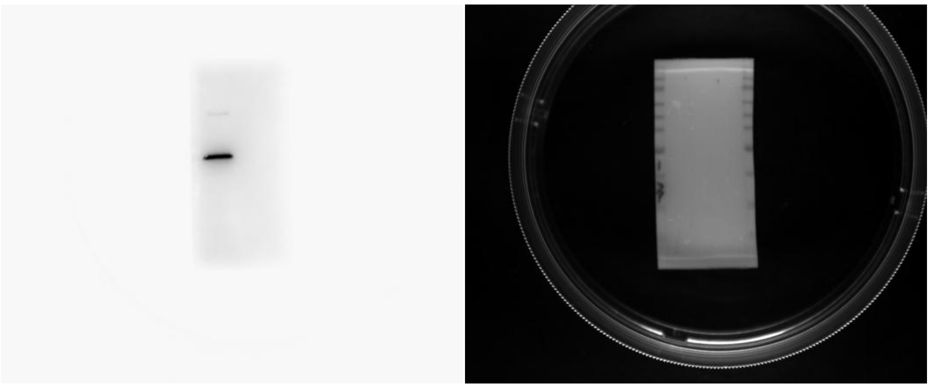

PRDX3 (IP: PRDX3)

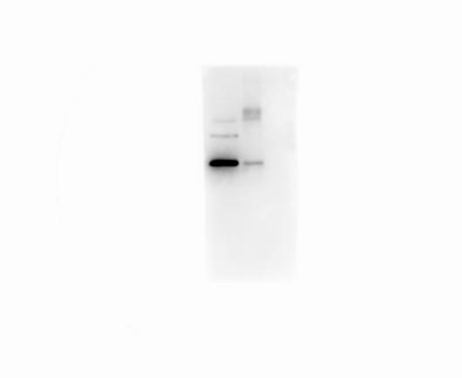
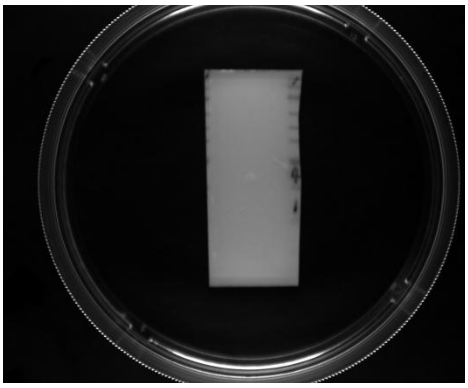

ALDH1L2 (IP: PRDX3)

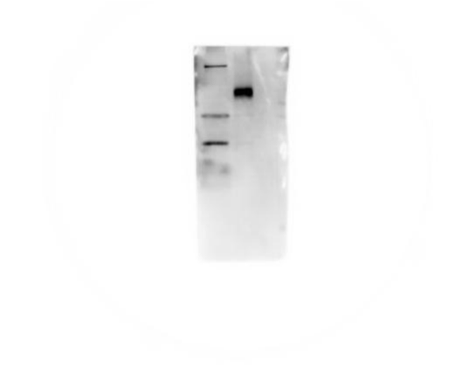
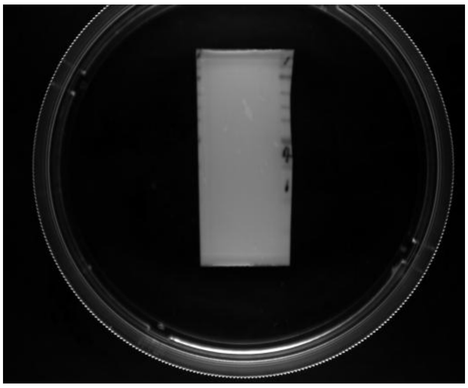

H69AR

Flag-ALDH1L2 (IP: Flag-ALDH1L2)

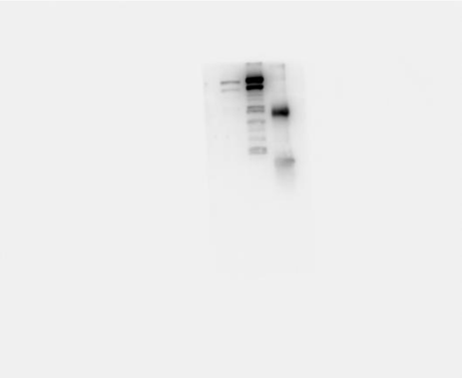
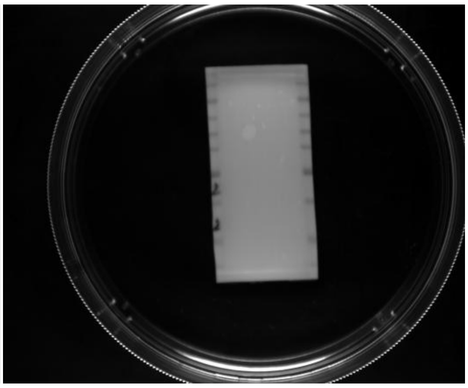

PRDX3 (IP: Flag-ALDH1L2)

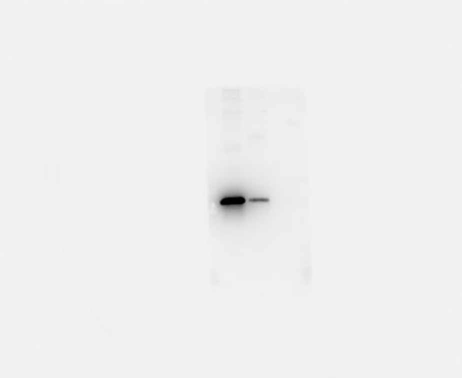
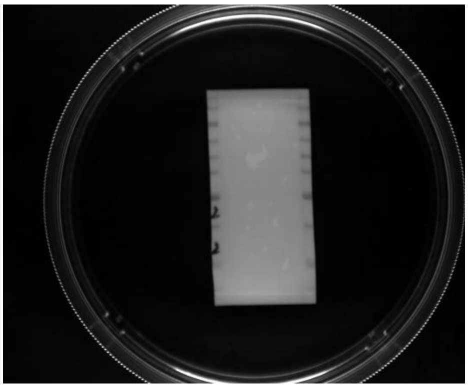

PRDX3 (IP: PRDX3)

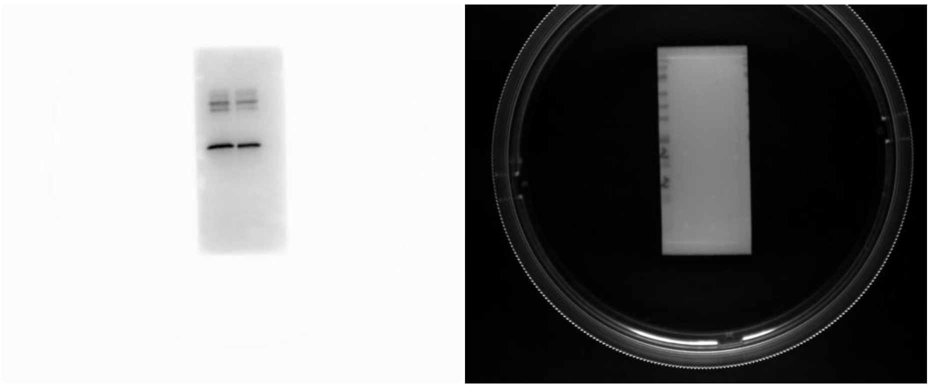

ALDH1L2 (IP: PRDX3)

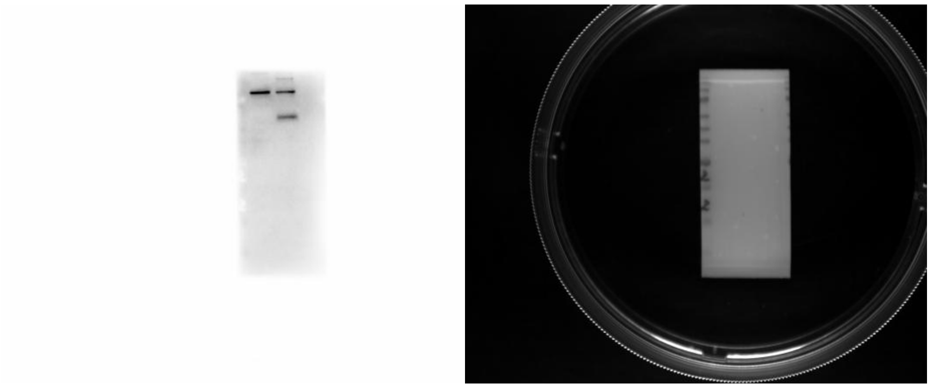

H446

Flag-ALDH1L2 (IP: Flag-ALDH1L2)

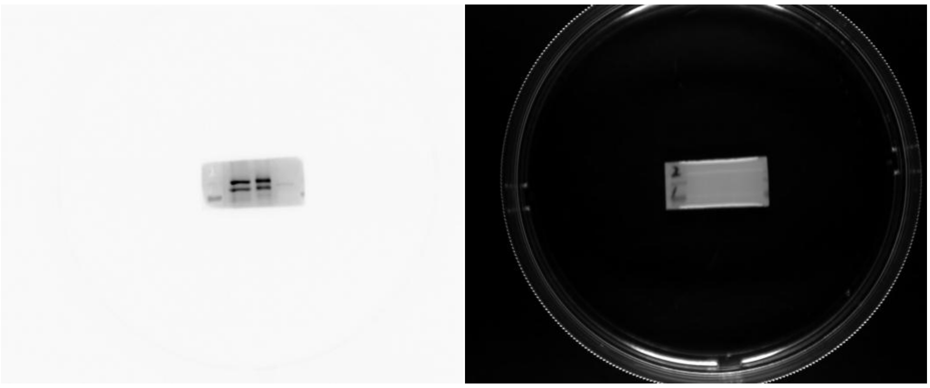

PRDX3 (IP: Flag-ALDH1L2)

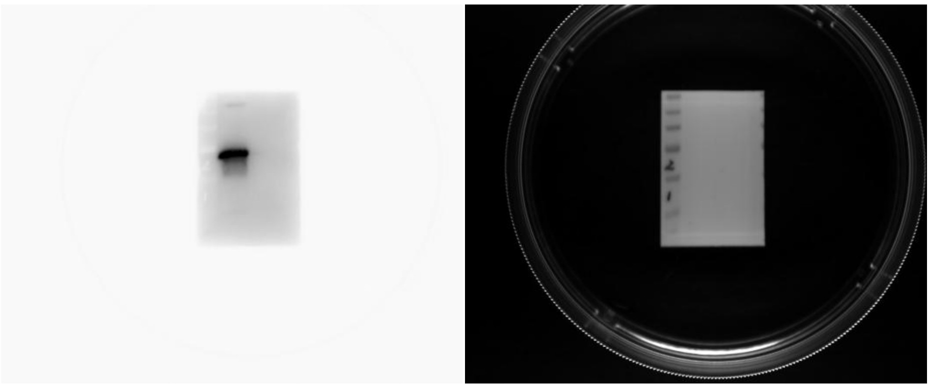

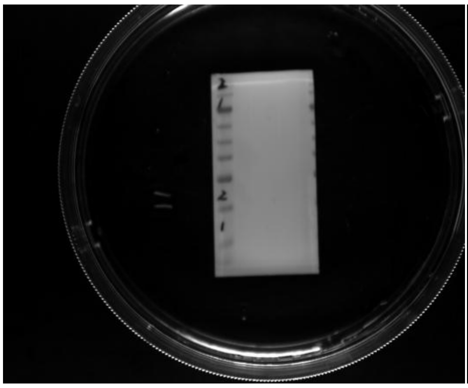
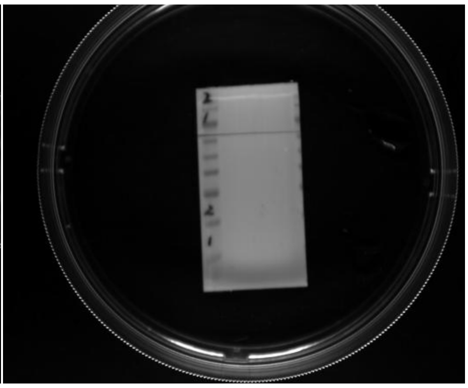

PRDX3 (IP: PRDX3)

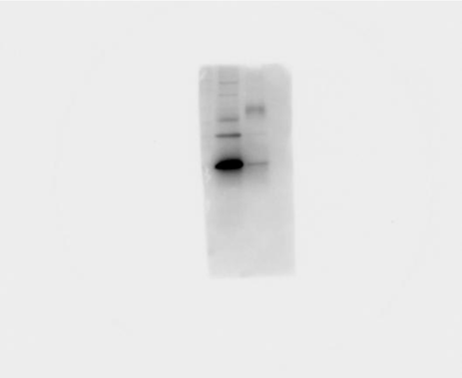
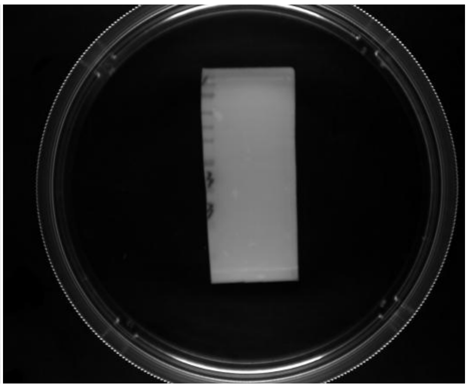

ALDH1L2 (IP: PRDX3)

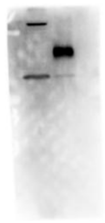
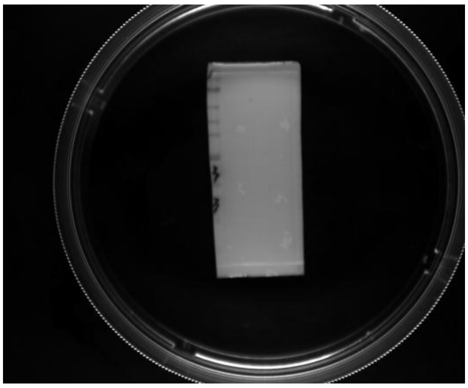

H446DDP

Flag-ALDH1L2 (IP: Flag-ALDH1L2)

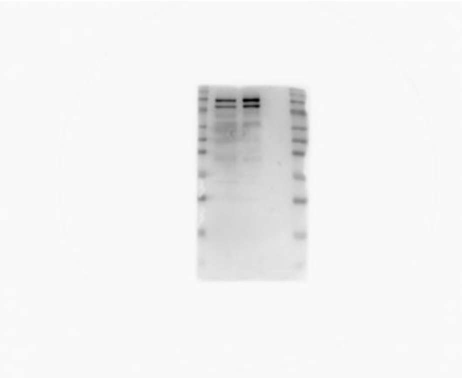
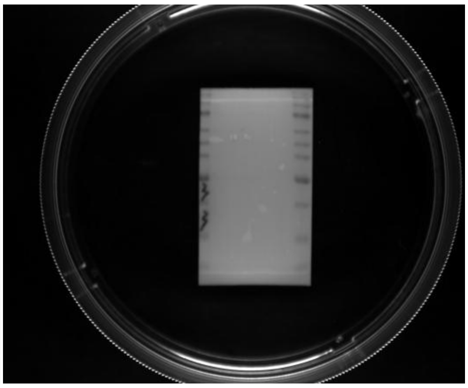

PRDX3 (IP: Flag-ALDH1L2)

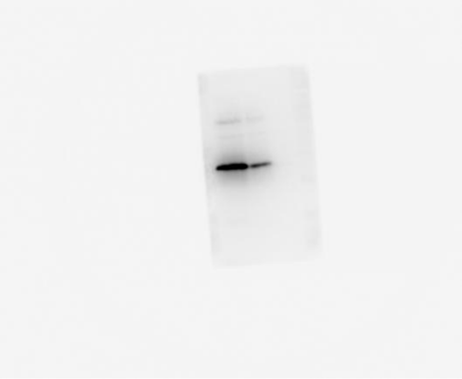

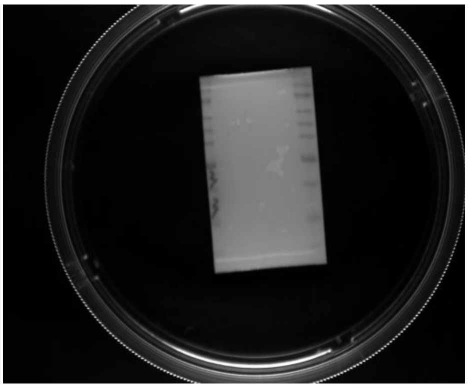

PRDX3 (IP: PRDX3)

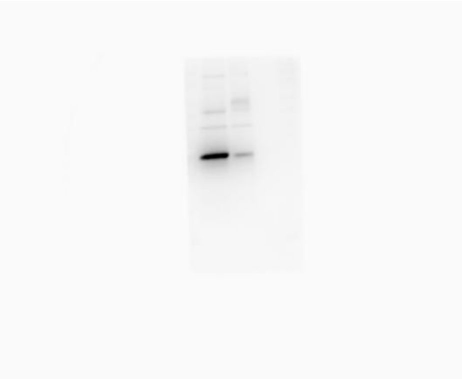

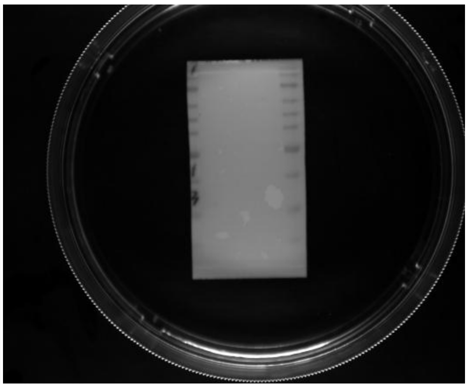

ALDH1L2 (IP: PRDX3)

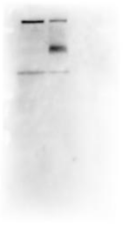

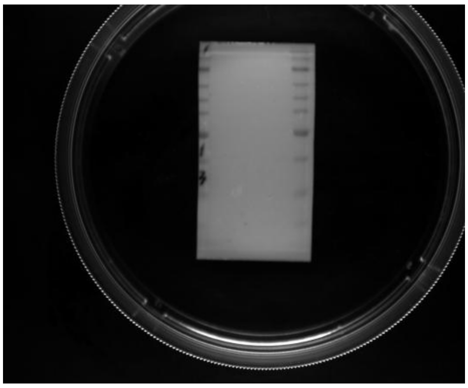

Fig. 7C First Repetition

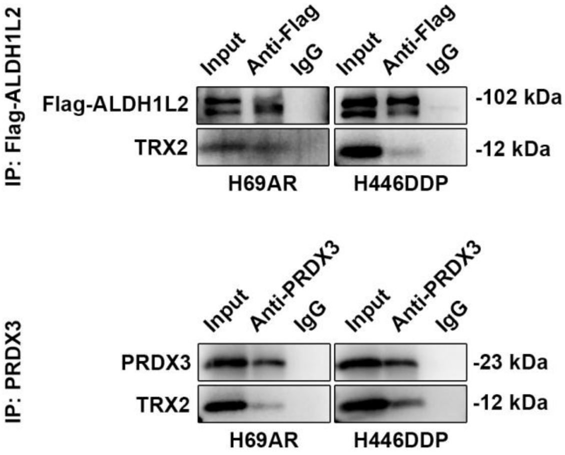

H69AR

Flag-ALDH1L2 (IP: Flag-ALDH1L2)

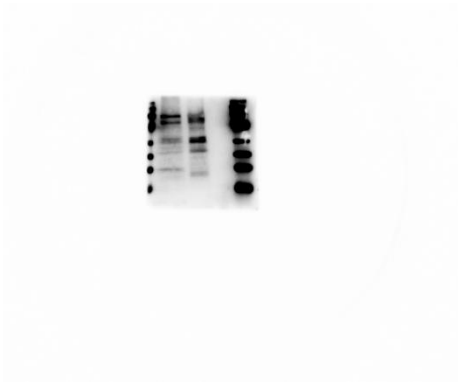

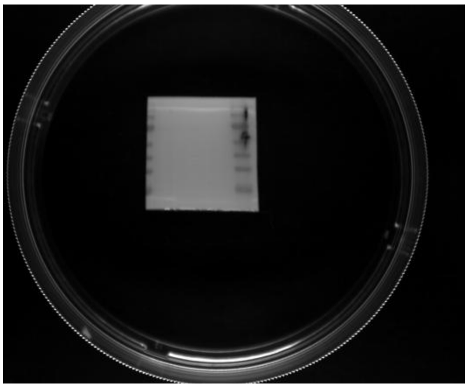

TRX2 (IP: Flag-ALDH1L2)

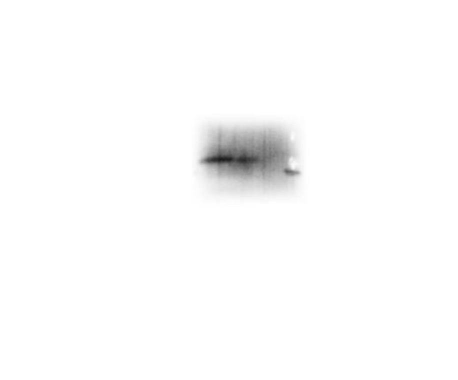

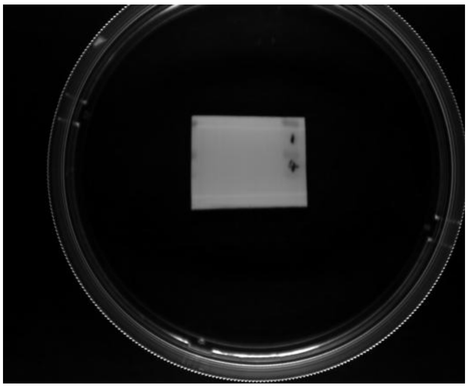

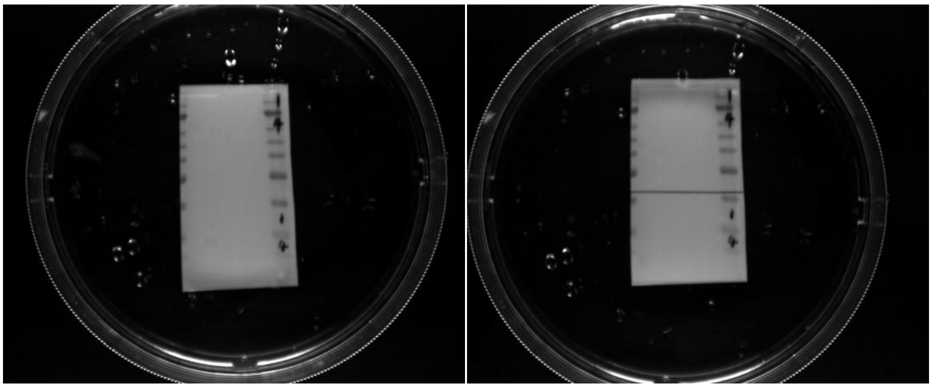

PRDX3 (IP: PRDX3)

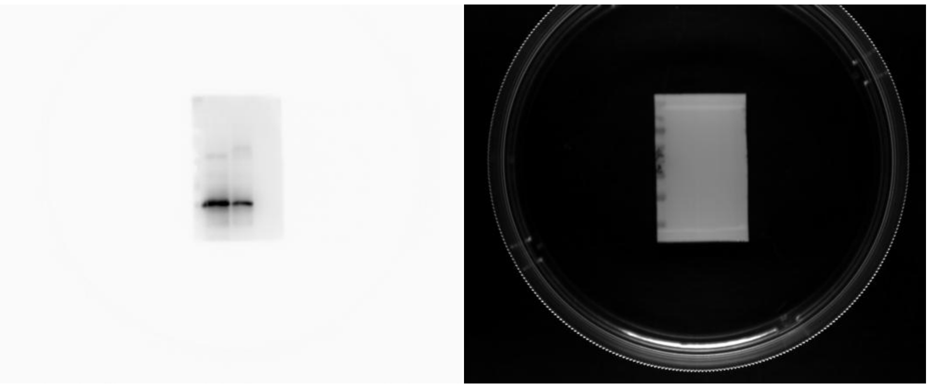

TRX2 (IP: PRDX3)

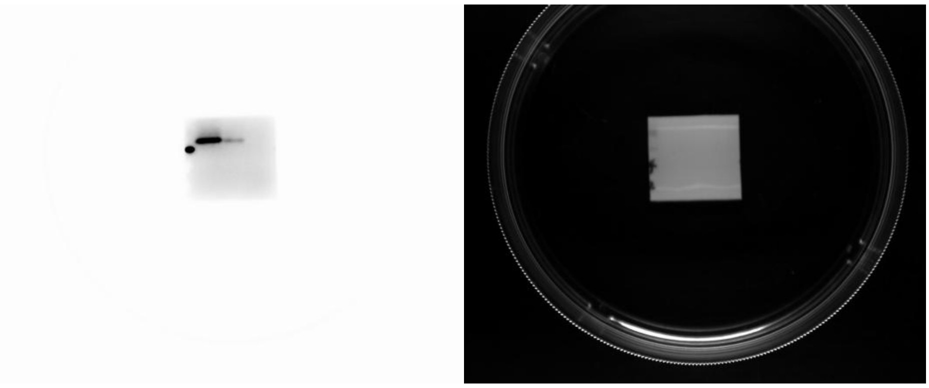

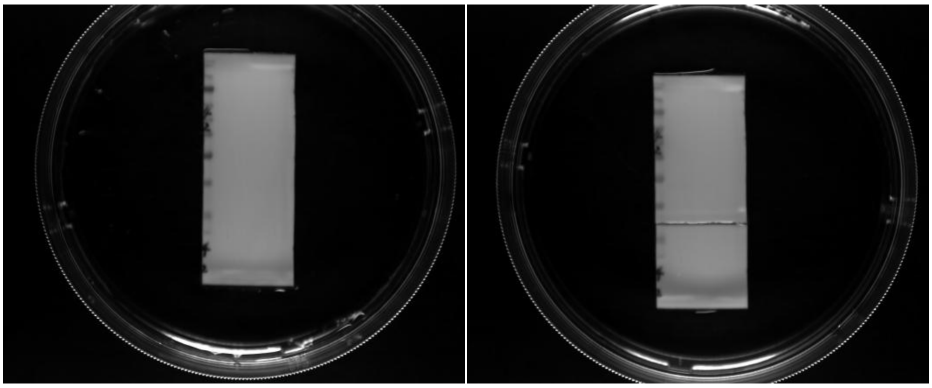

H446DDP

Flag-ALDH1L2 (IP: Flag-ALDH1L2)

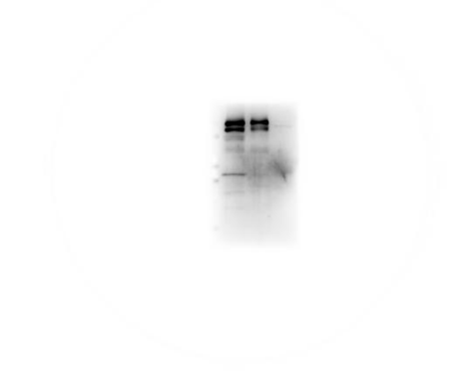
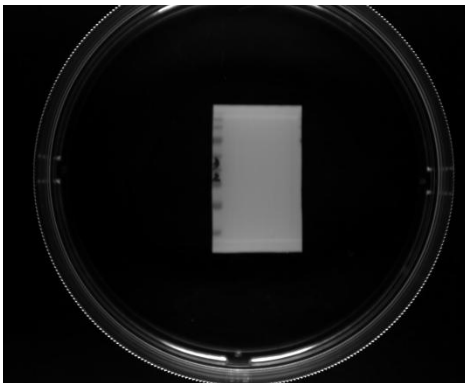

TRX2 (IP: Flag-ALDH1L2)

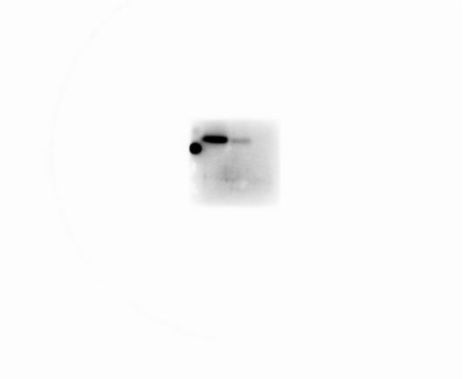
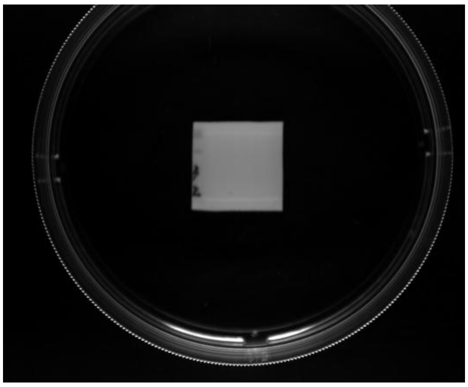

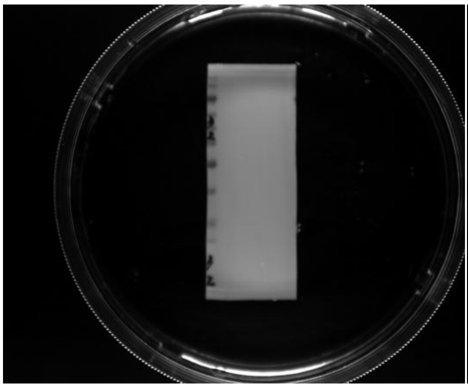
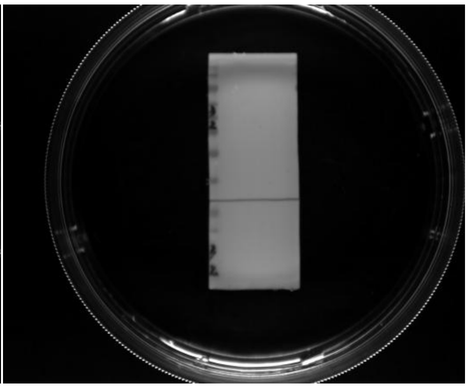

PRDX3 (IP: PRDX3)

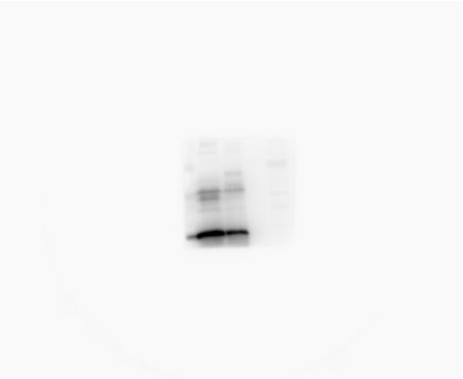
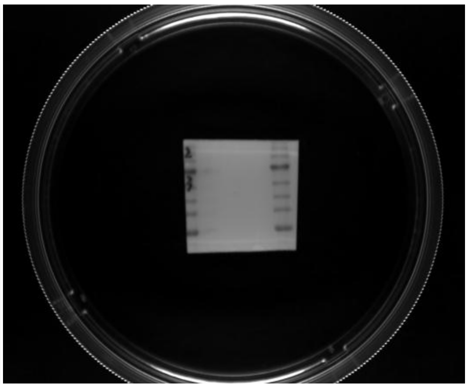

TRX2 (IP: PRDX3)

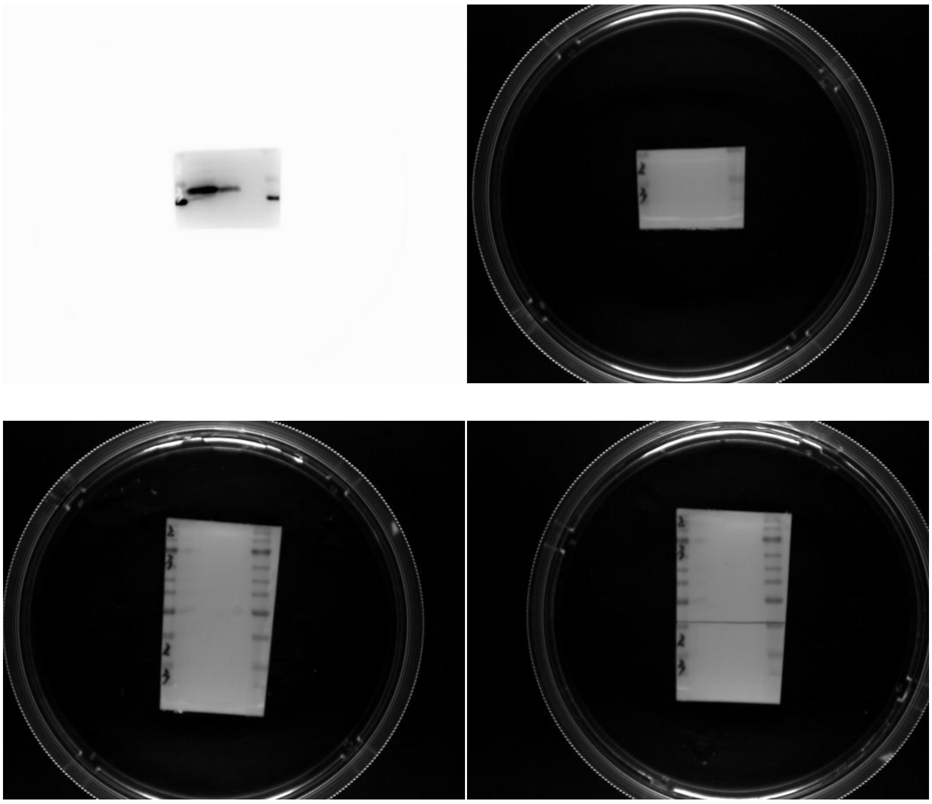

Fig. 7C Second Repetition

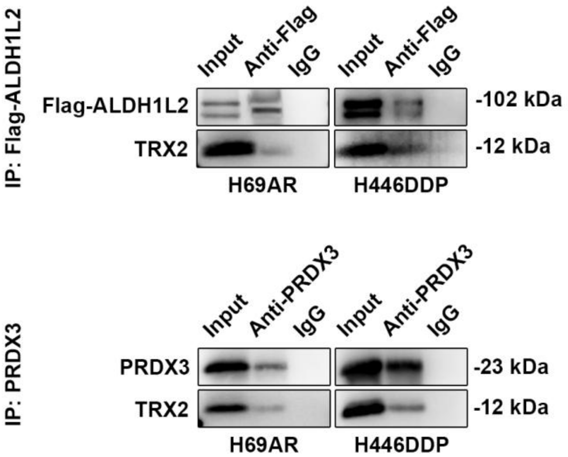

H69AR

Flag-ALDH1L2 (IP: Flag-ALDH1L2)

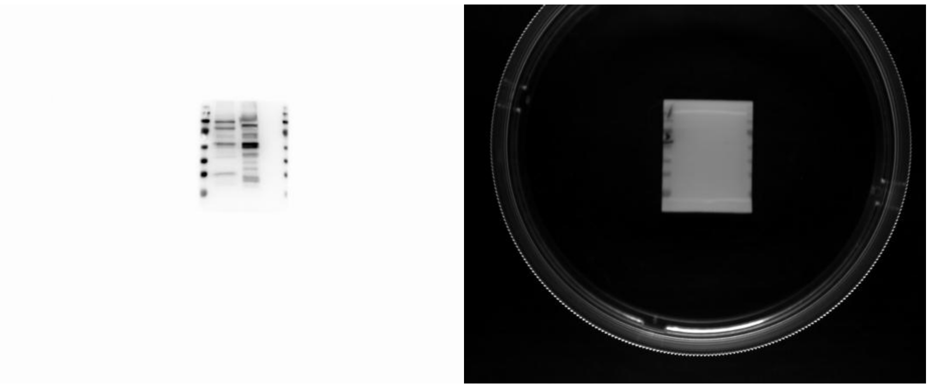

TRX2 (IP: Flag-ALDH1L2)

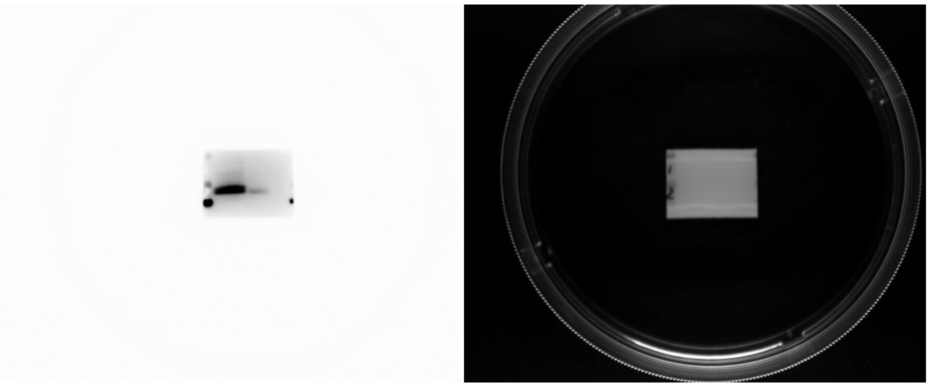

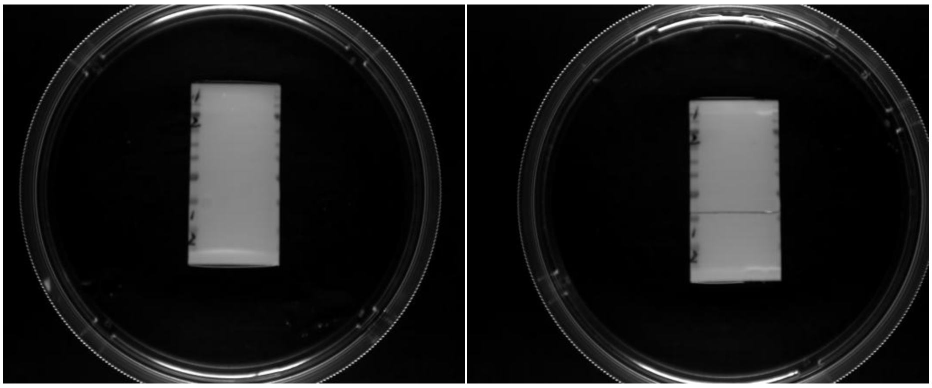

PRDX3 (IP: PRDX3)

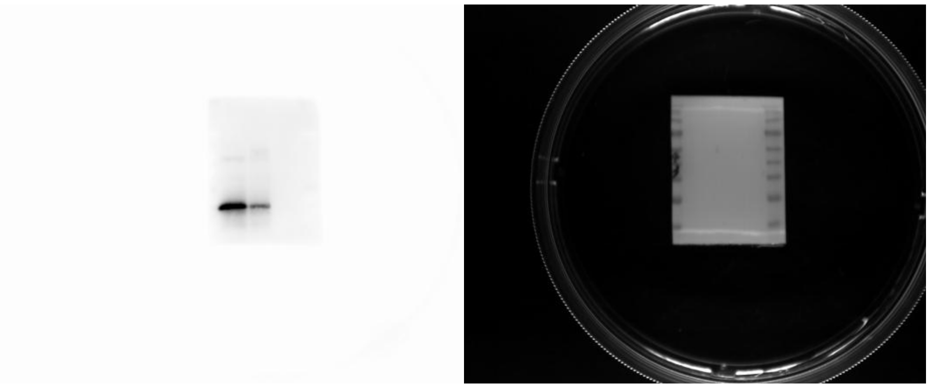

TRX2 (IP: PRDX3)

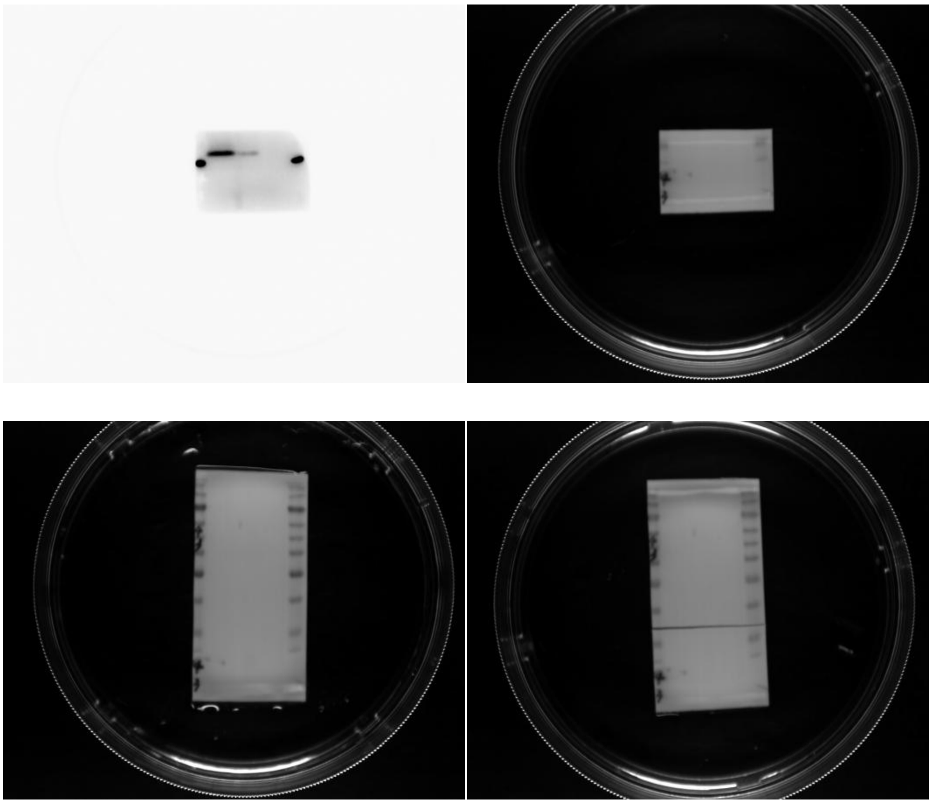

H446DDP

Flag-ALDH1L2 (IP: Flag-ALDH1L2)

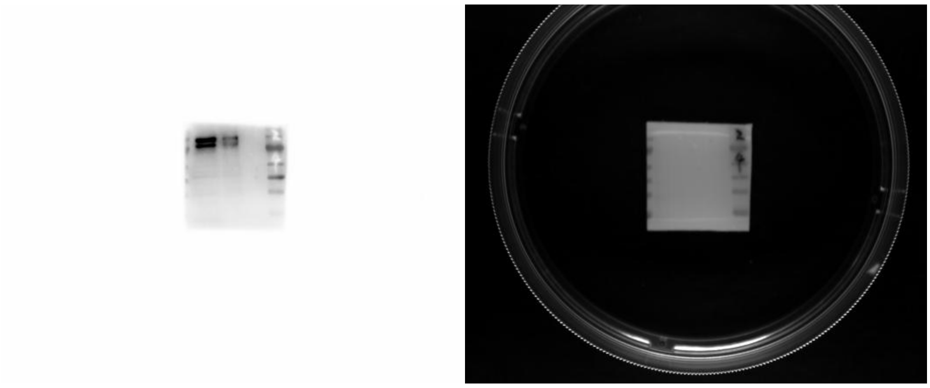

TRX2 (IP: Flag-ALDH1L2)

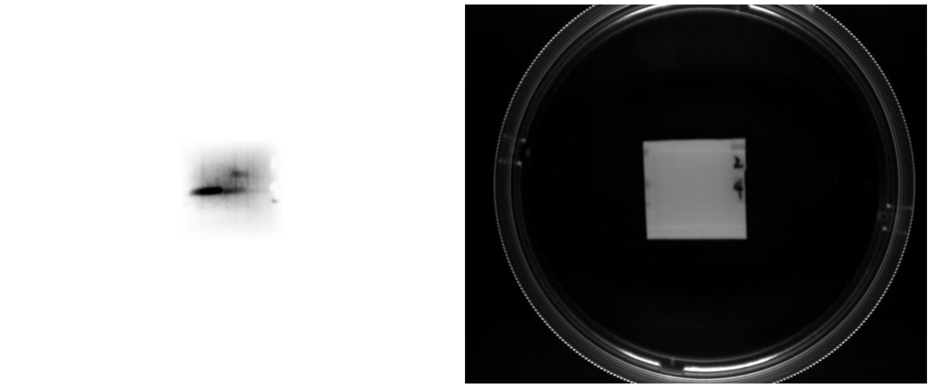

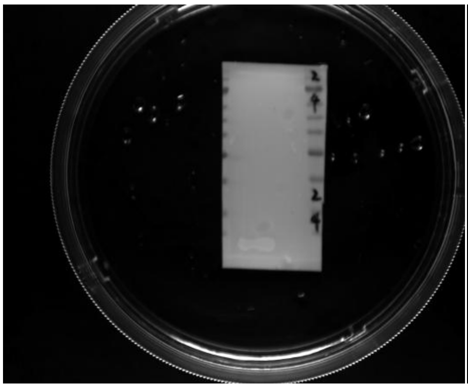
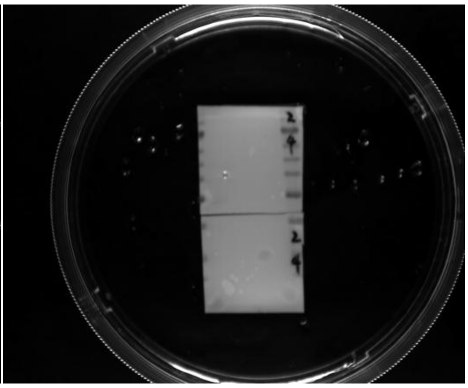

PRDX3 (IP: PRDX3)

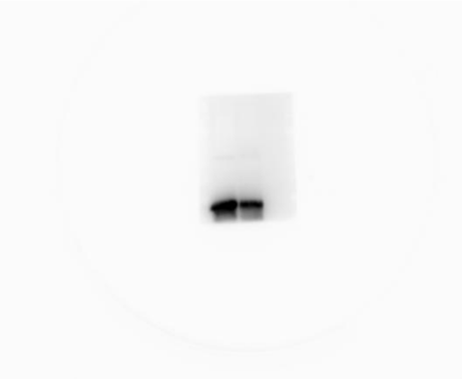
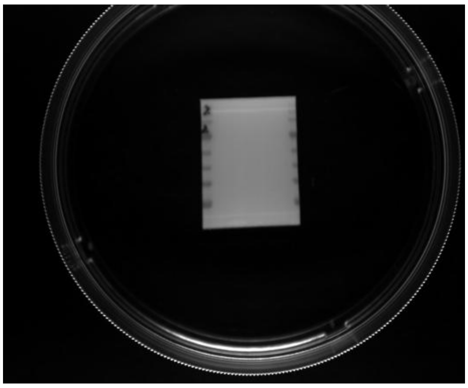

TRX2 (IP: PRDX3)

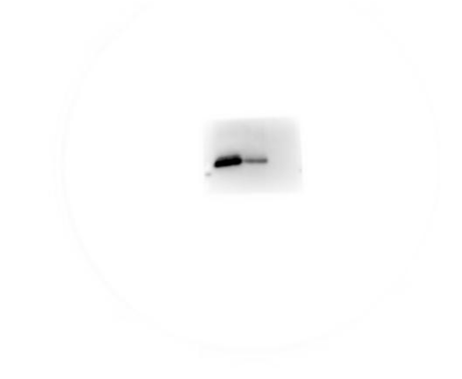
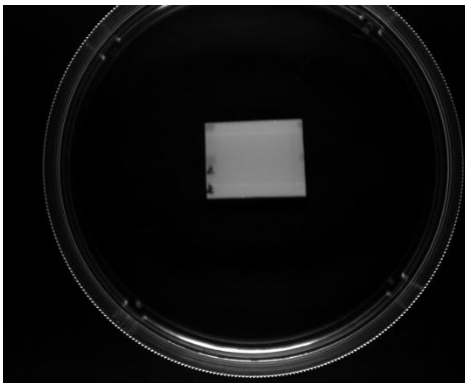

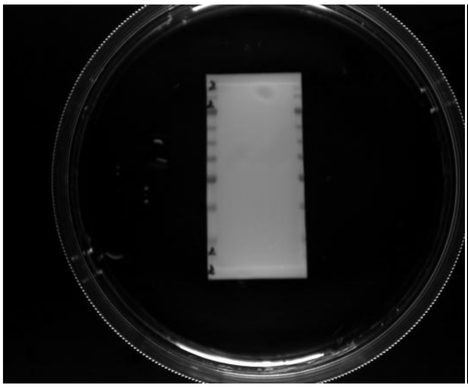
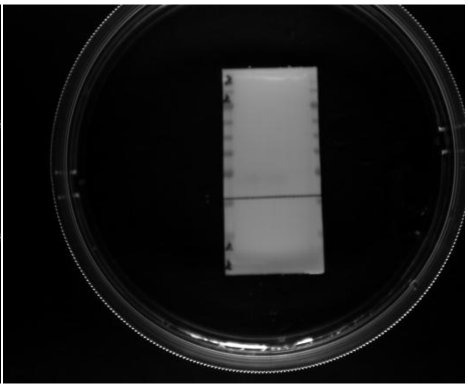

Fig. 7C Third Repetition

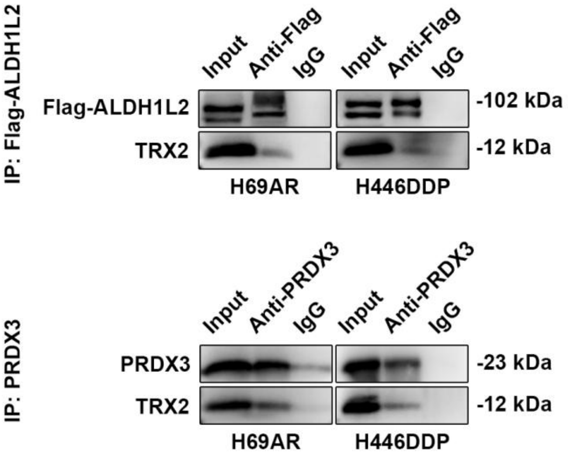

H69AR

Flag-ALDH1L2 (IP: Flag-ALDH1L2)

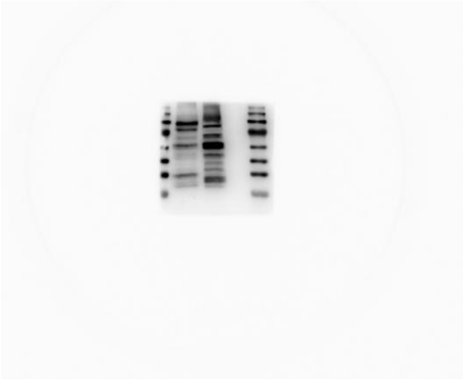
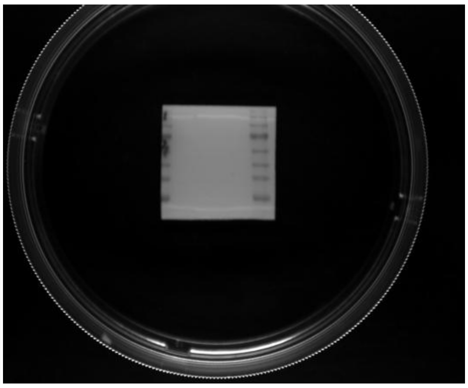

TRX2 (IP: Flag-ALDH1L2)

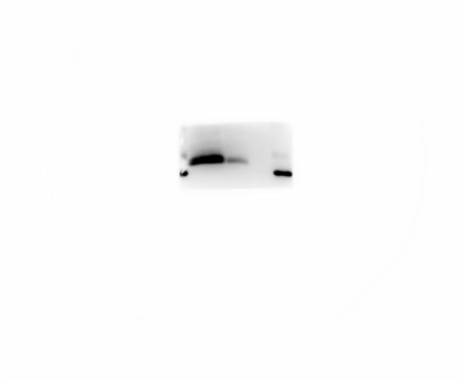
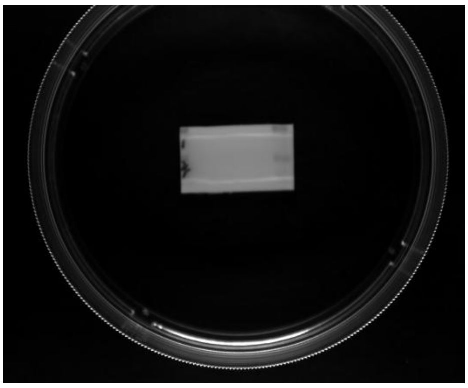

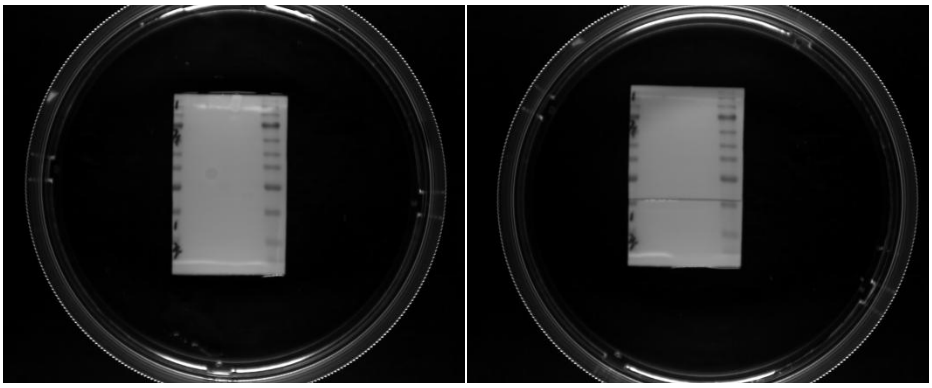

PRDX3 (IP: PRDX3)

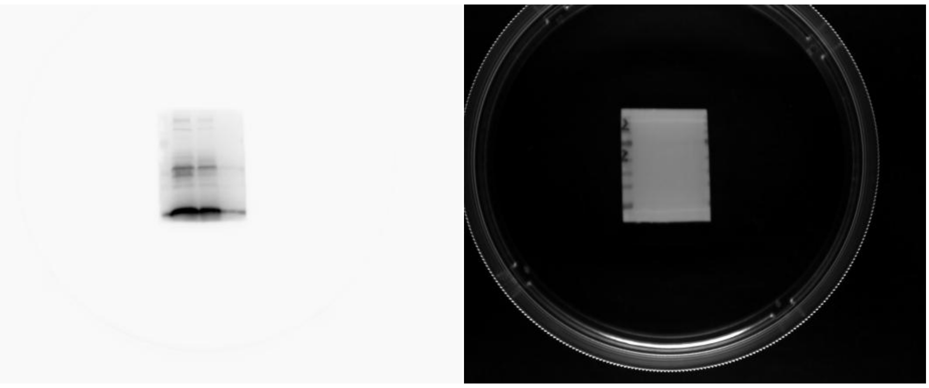

TRX2 (IP: PRDX3)

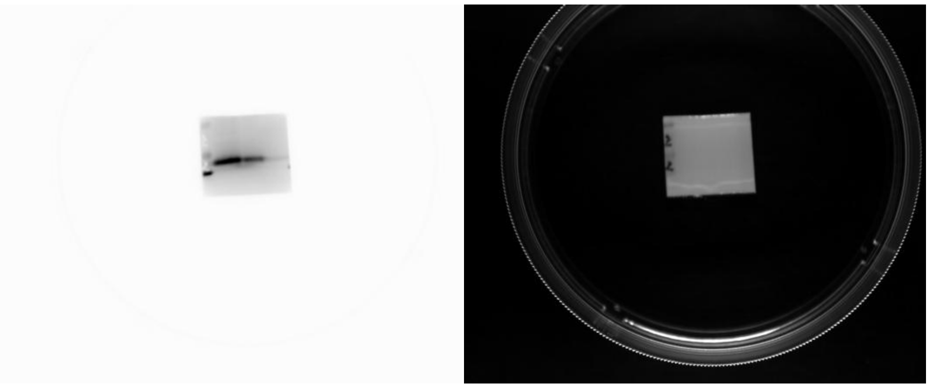

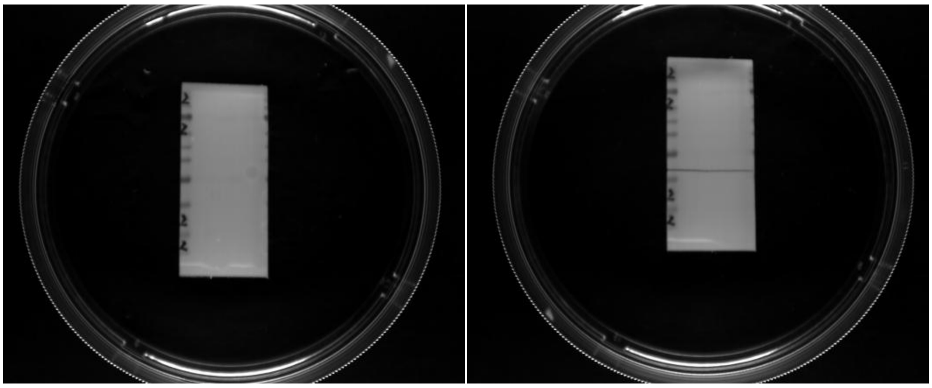

H446DDP

Flag-ALDH1L2 (IP: Flag-ALDH1L2)

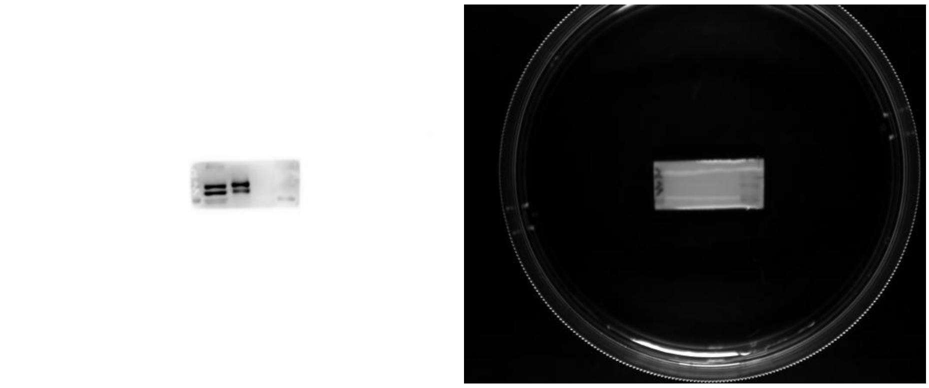

TRX2 (IP: Flag-ALDH1L2)

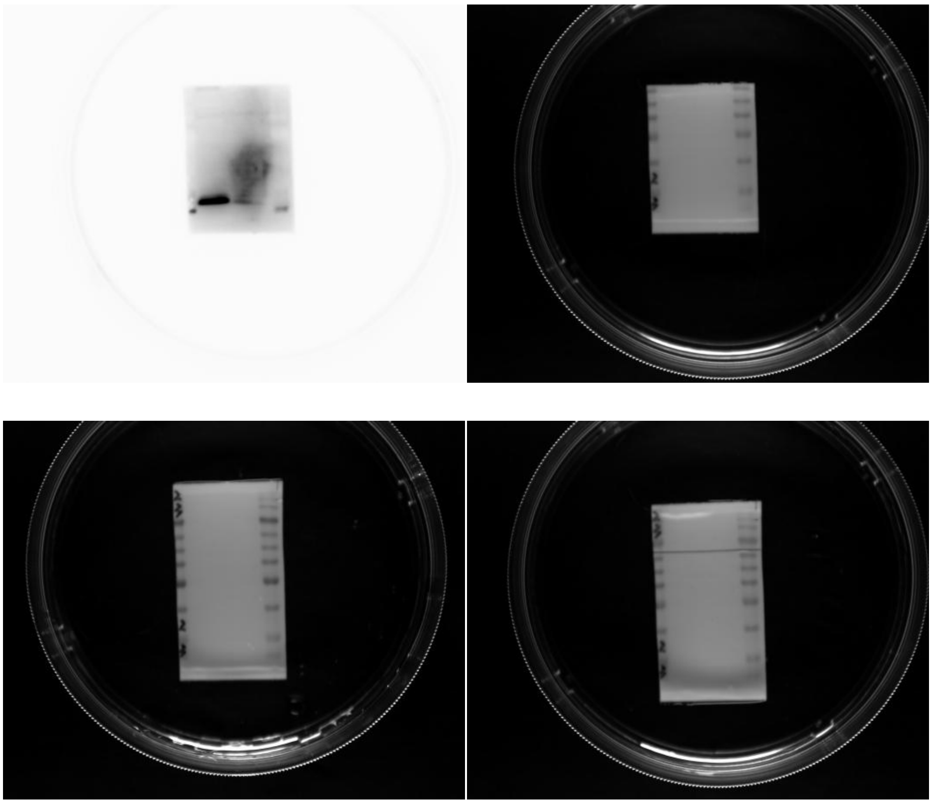

PRDX3 (IP: PRDX3)

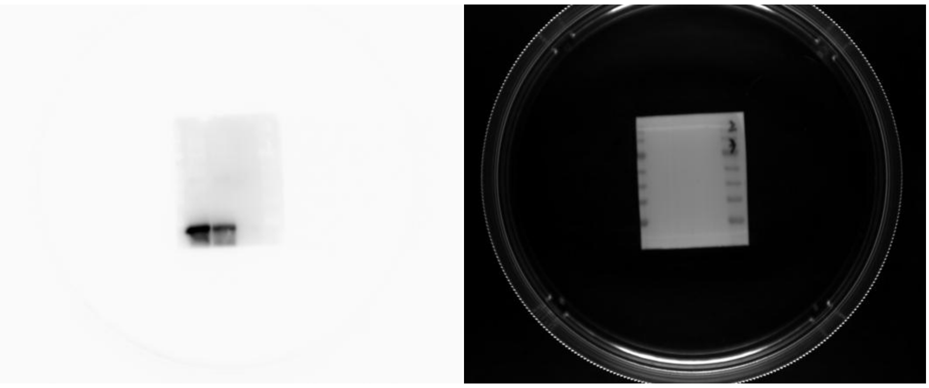

TRX2 (IP: PRDX3)

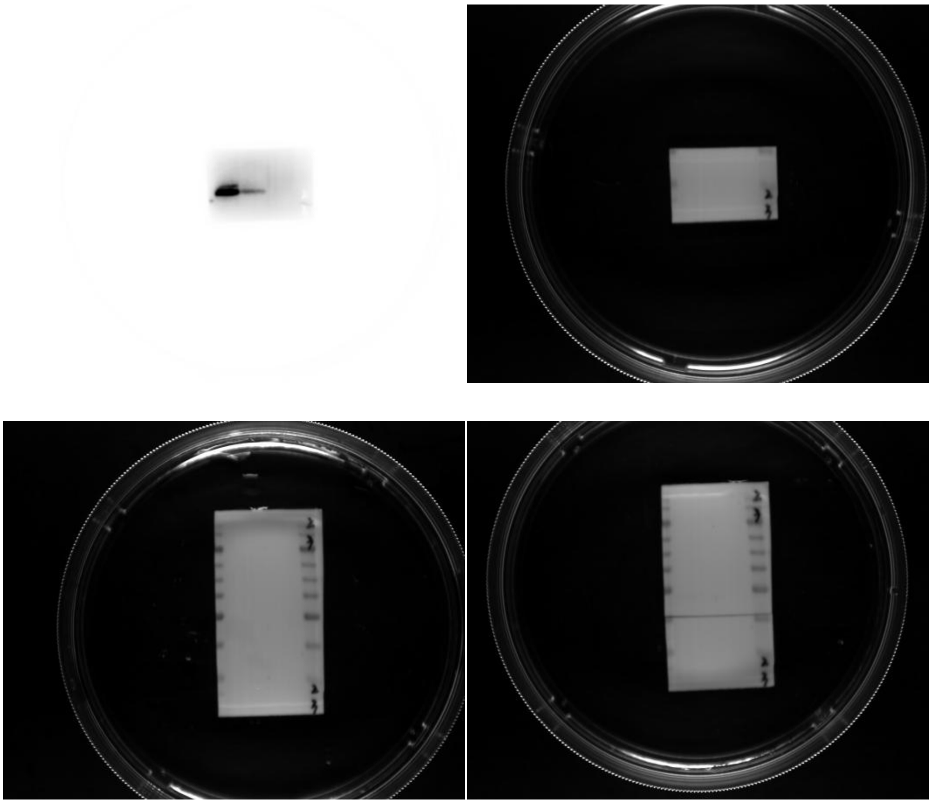

Fig. 7D First Repetition

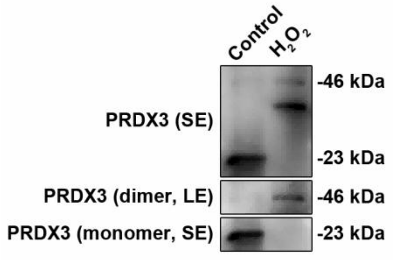

Control vs. H_2O_2

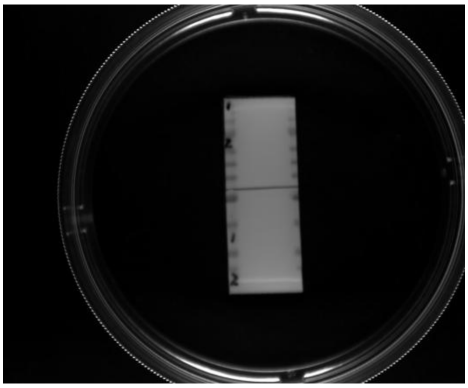

PRDX3 (SE)

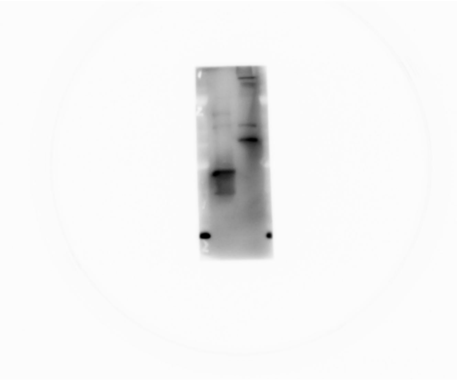

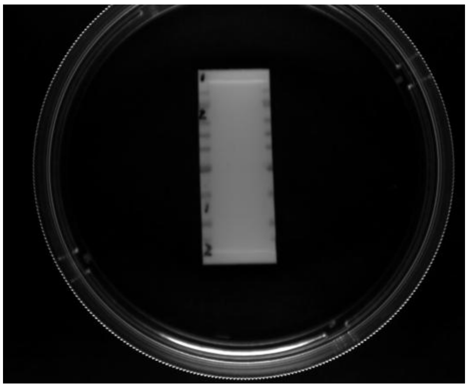

PRDX3 (dimer, LE)

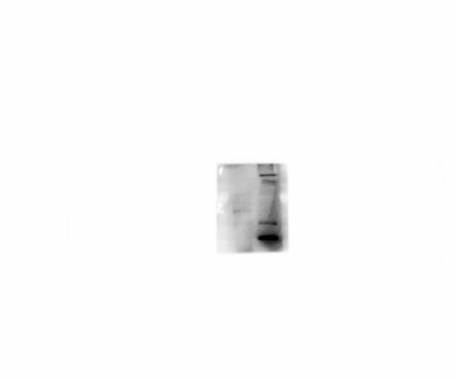

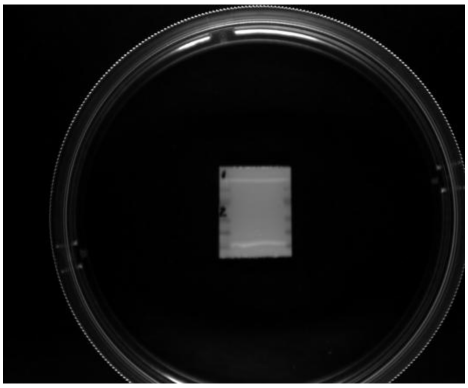

PRDX3 (monomer, SE)

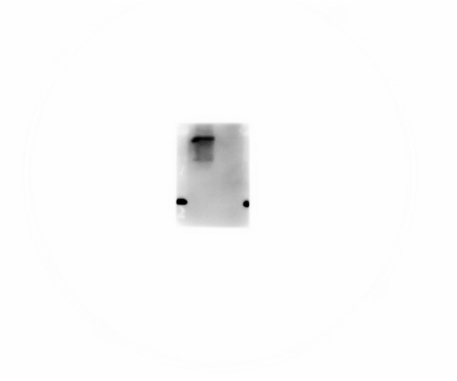

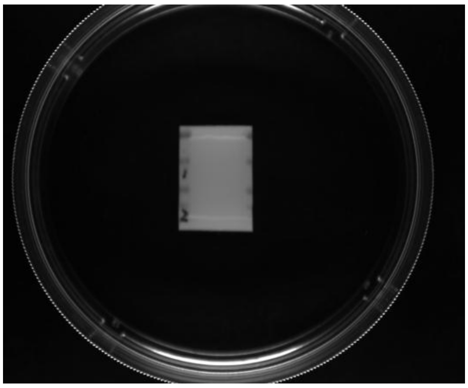

Fig. 7D Second Repetition

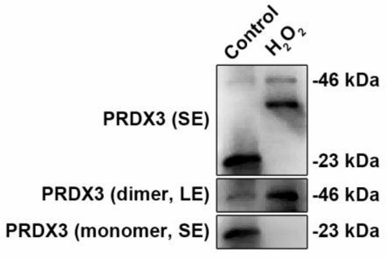

Control vs. H_2O_2

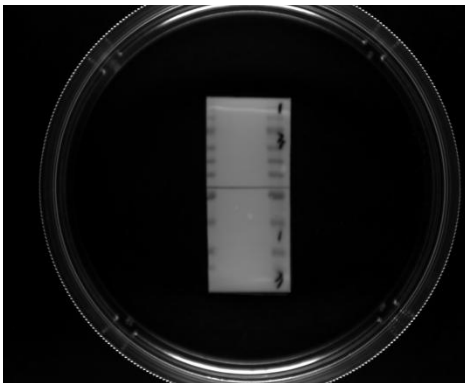

PRDX3 (SE)

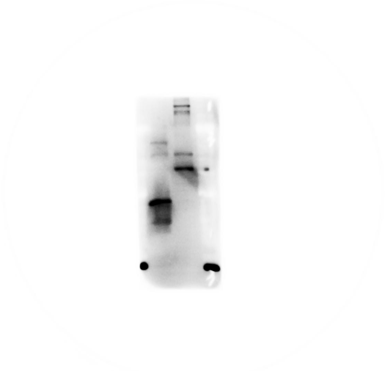

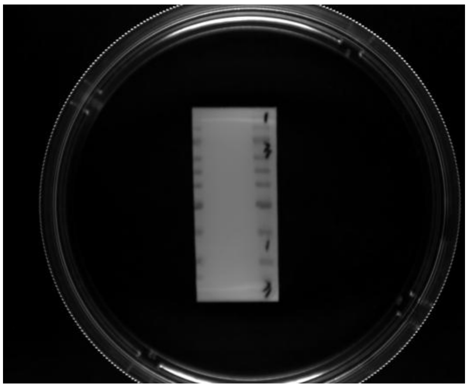

PRDX3 (dimer, LE)

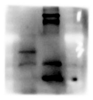

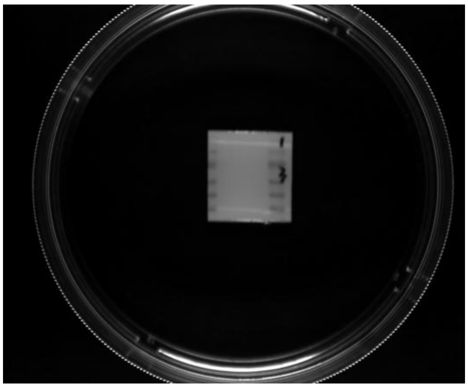

PRDX3 (monomer, SE)

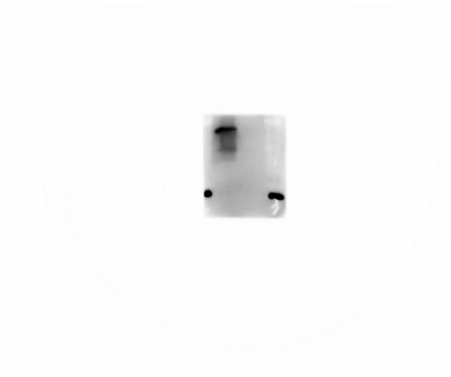

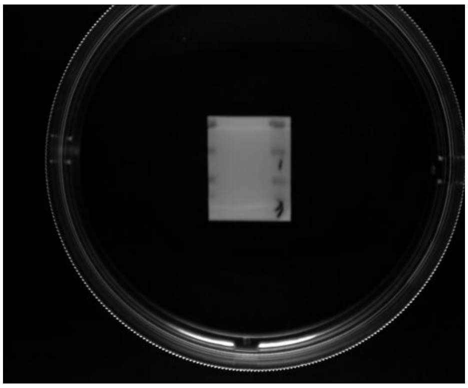

Fig. 7D Third Repetition

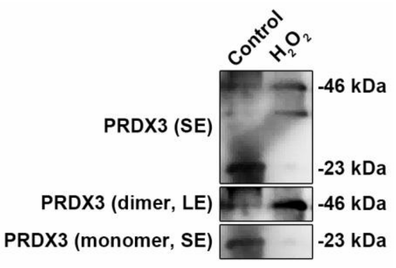

Control vs. H_2O_2

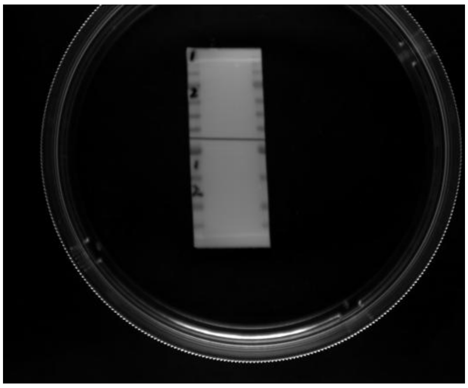

PRDX3 (SE)

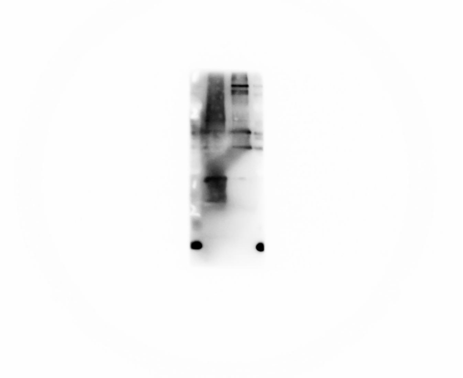

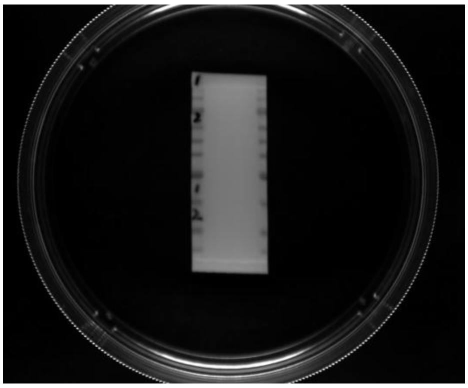

PRDX3 (dimer, LE)

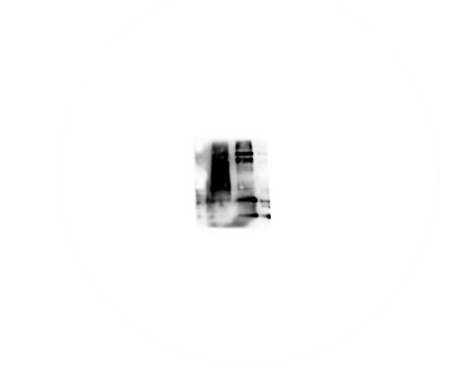

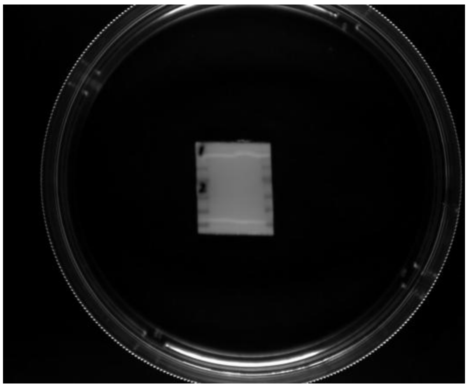

PRDX3 (monomer, SE)

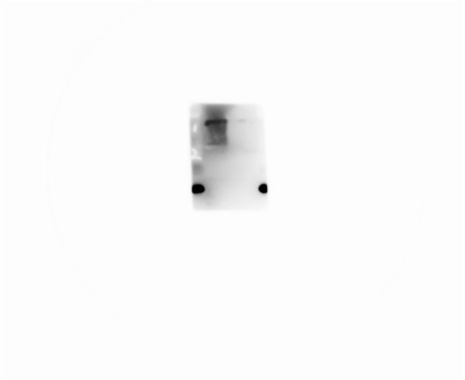

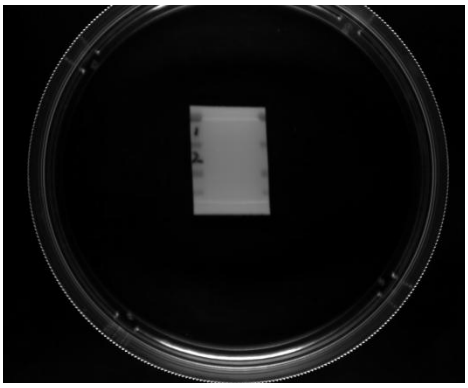

Fig. 7F First Repetition

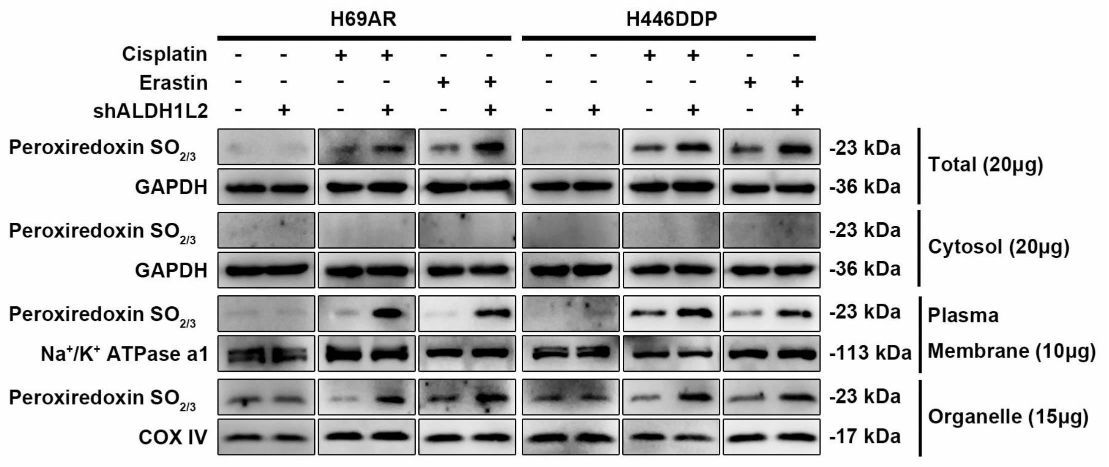

H69AR

Peroxiredoxin $SO_{2/3}$ (untreated, total)

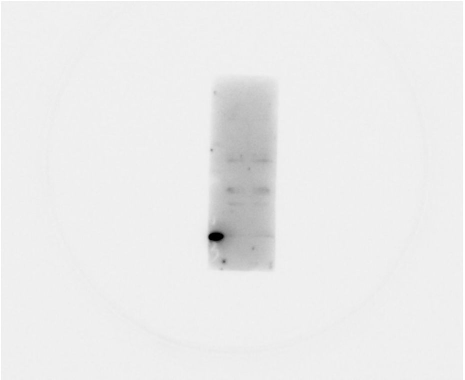

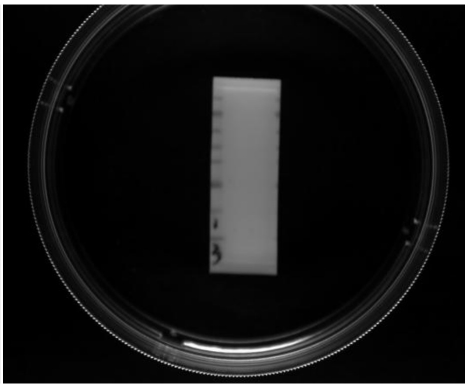

GAPDH (untreated, total)

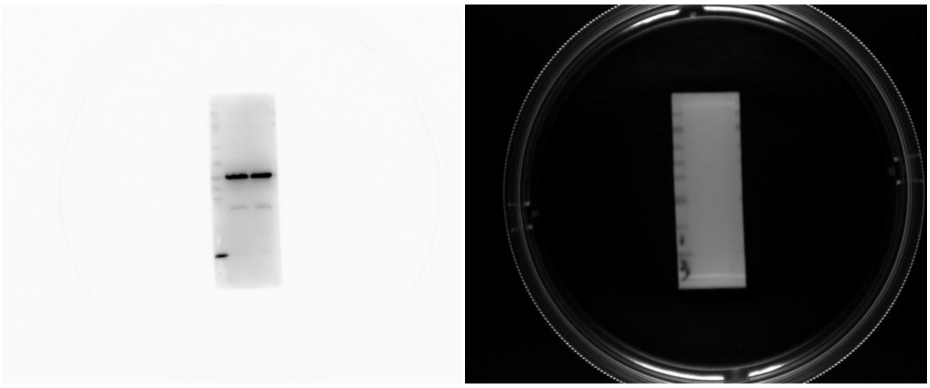

Peroxiredoxin $SO_{2/3}$ (untreated, cytosol)

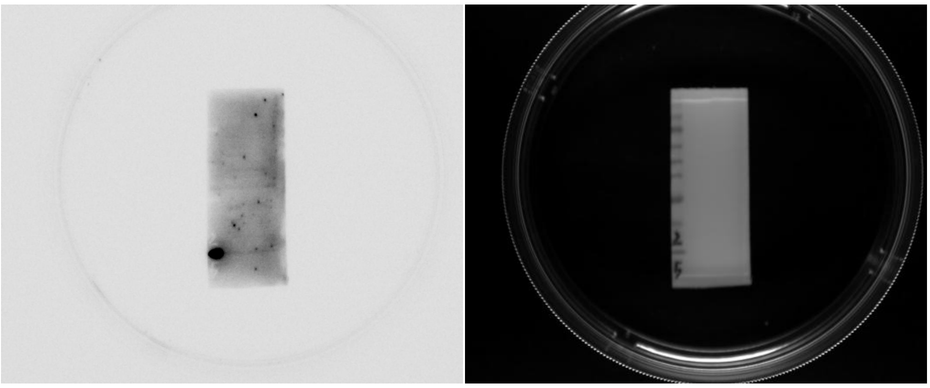

GAPDH (untreated, cytosol)

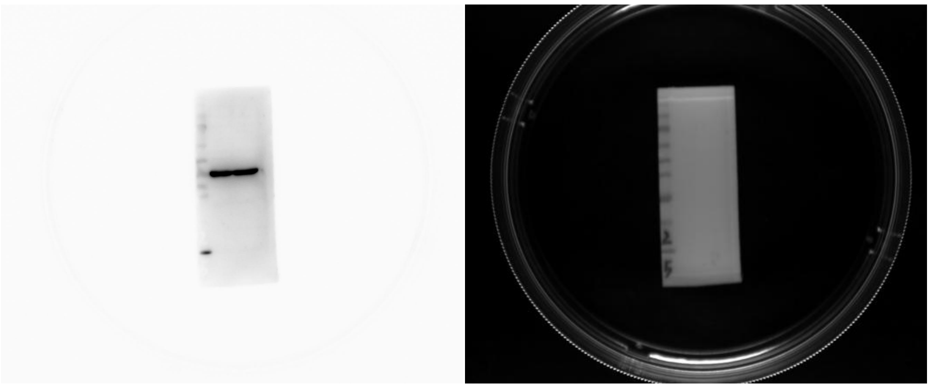

Peroxiredoxin $SO_{2/3}$ (untreated, plasma membrane)

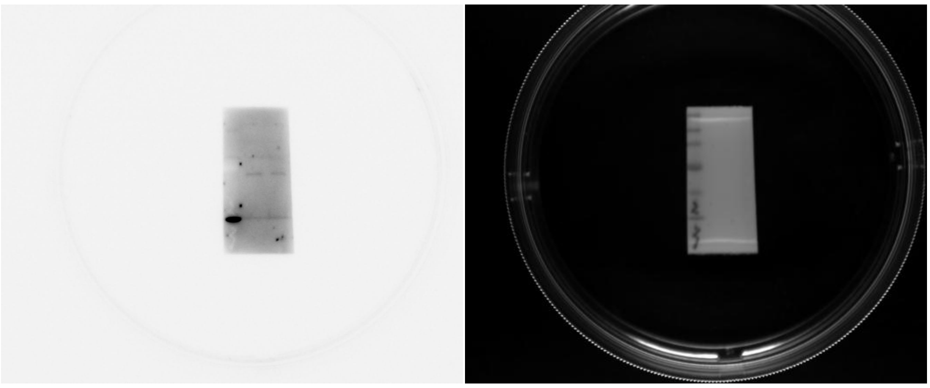

Na$^+$/K$^+$ ATPase a1 (untreated, plasma membrane)

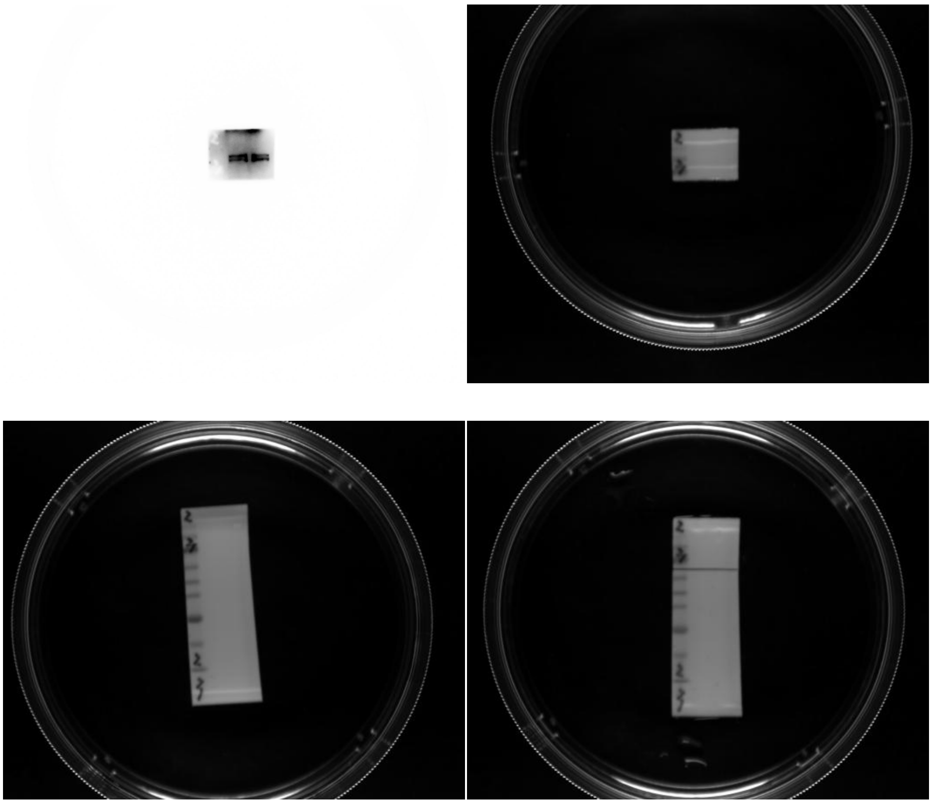

Peroxiredoxin $SO_{2/3}$ (untreated, organelle)

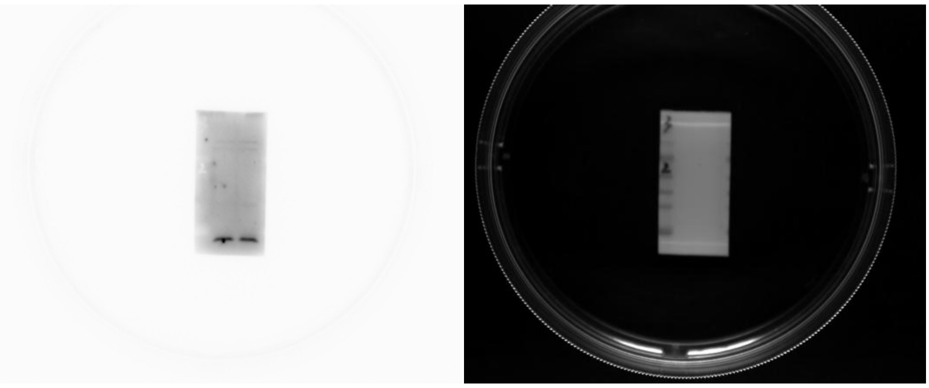

COX IV (untreated, organelle)

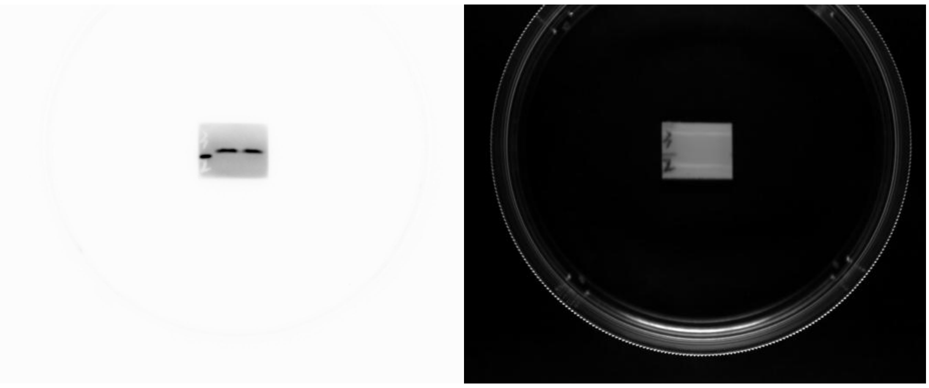

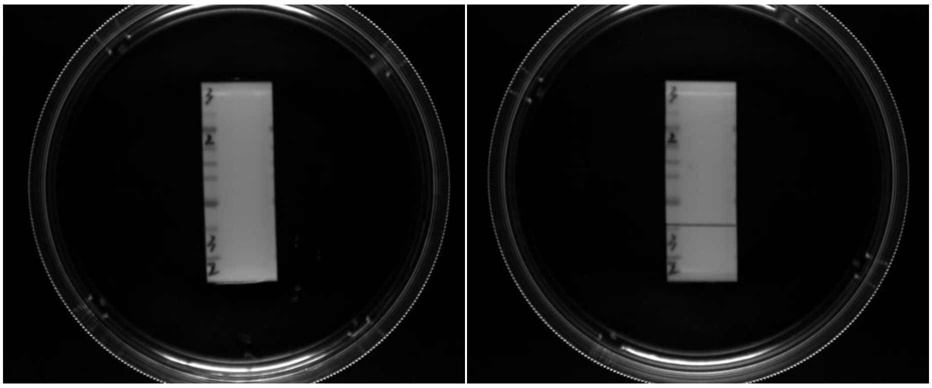

Peroxiredoxin $SO_{2/3}$ (cisplatin, total)

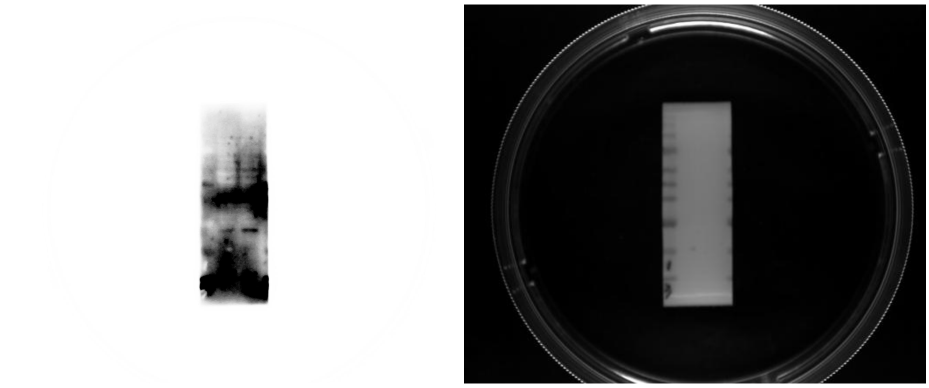

GAPDH (cisplatin, total)

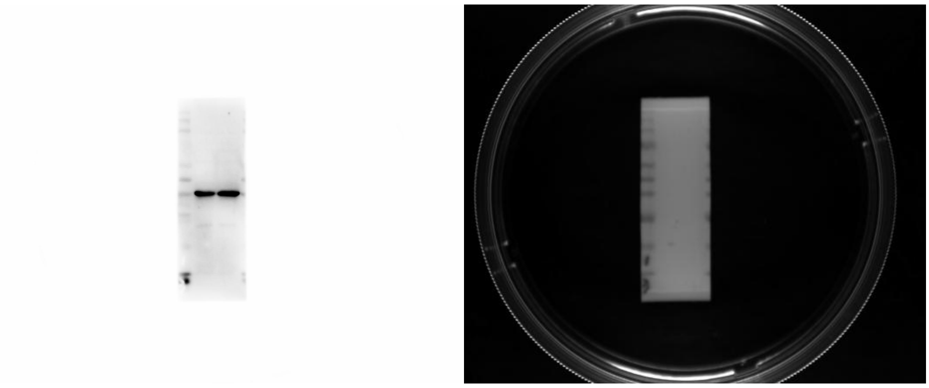

Peroxiredoxin $SO_{2/3}$ (cisplatin, cytosol)

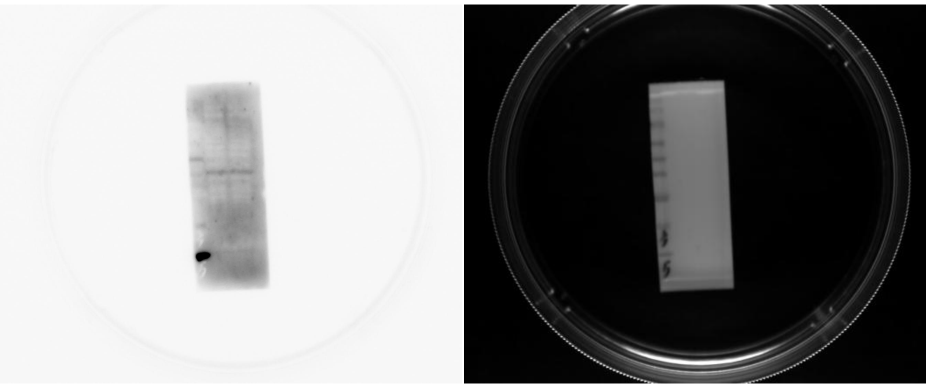

GAPDH (cisplatin, cytosol)

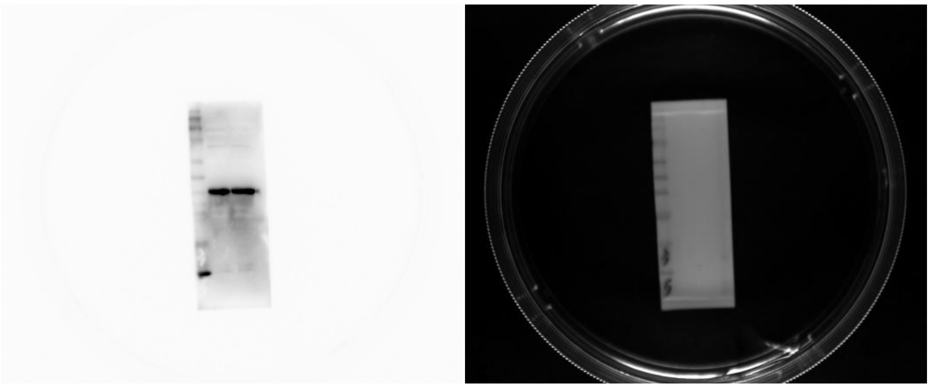

Peroxiredoxin $SO_{2/3}$ (cisplatin, plasma membrane)

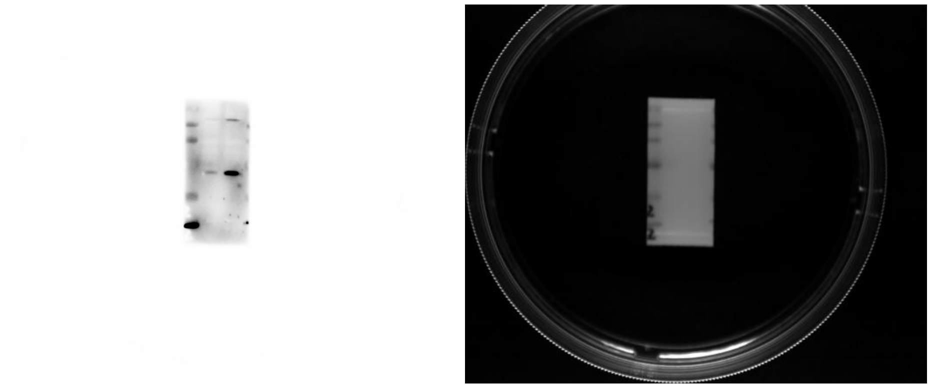

Na^{+}/K^{+} ATPase a1 (cisplatin, plasma membrane)

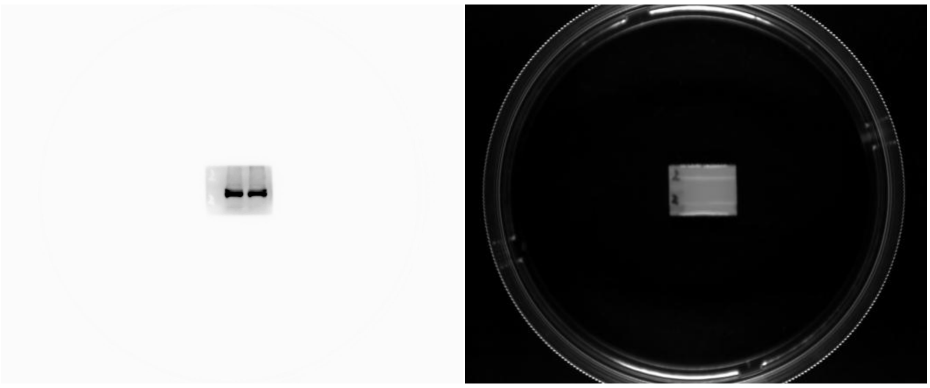

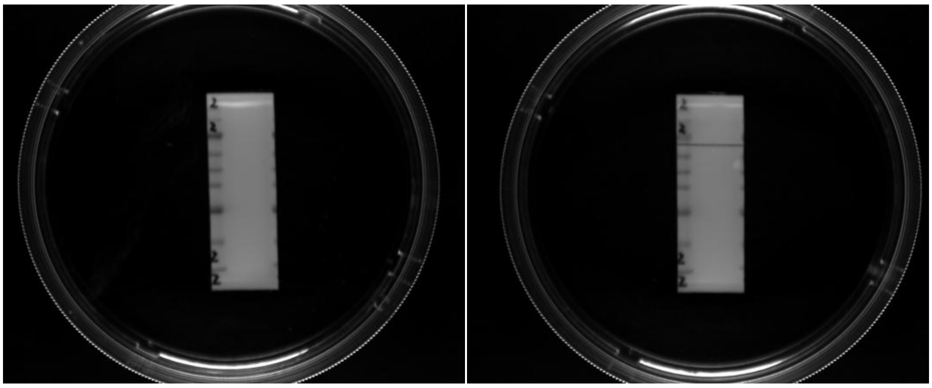

Peroxiredoxin $SO_{2/3}$ (cisplatin, organelle)

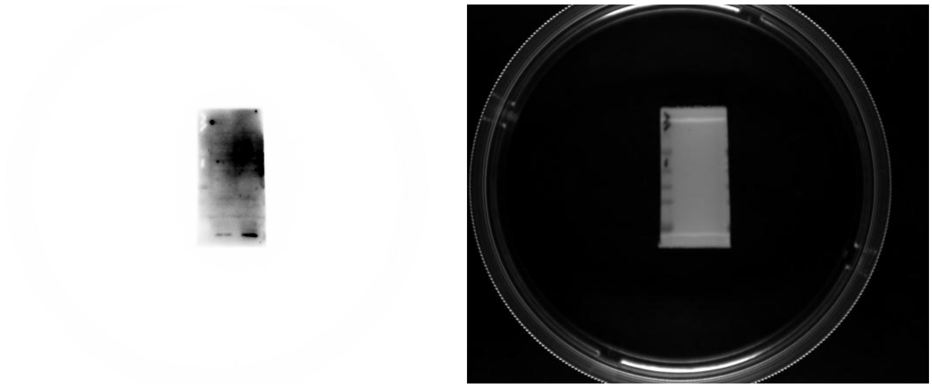

COX IV (cisplatin, organelle)

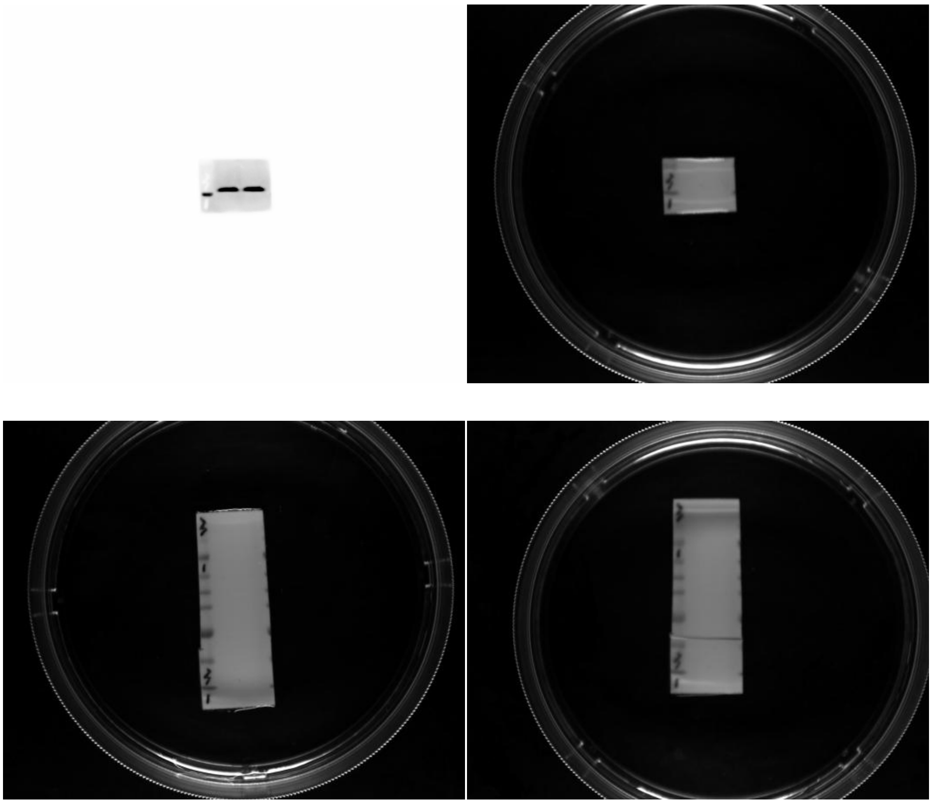

Peroxiredoxin $SO_{2/3}$ (erastin, total)

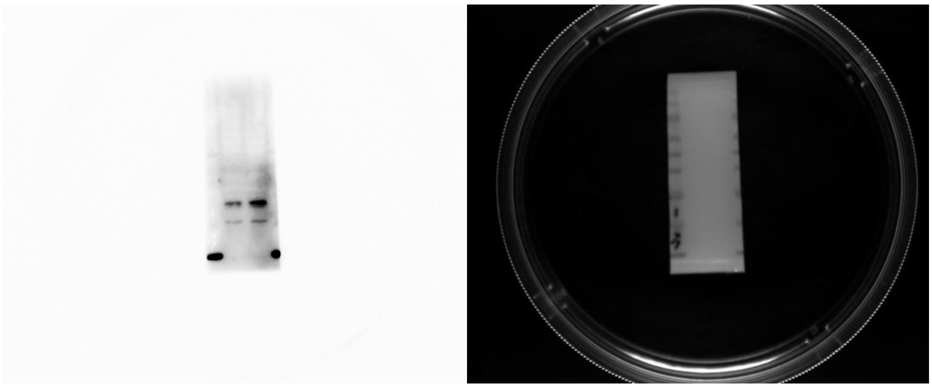

GAPDH (erastin, total)

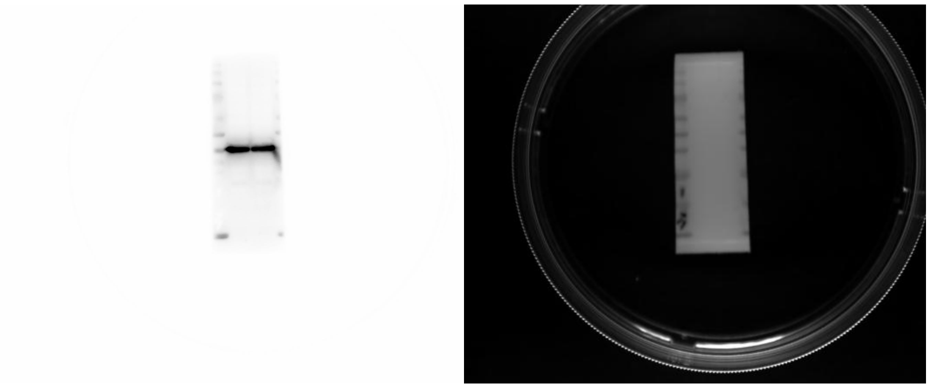

Peroxiredoxin $SO_{2/3}$ (erastin, cytosol)

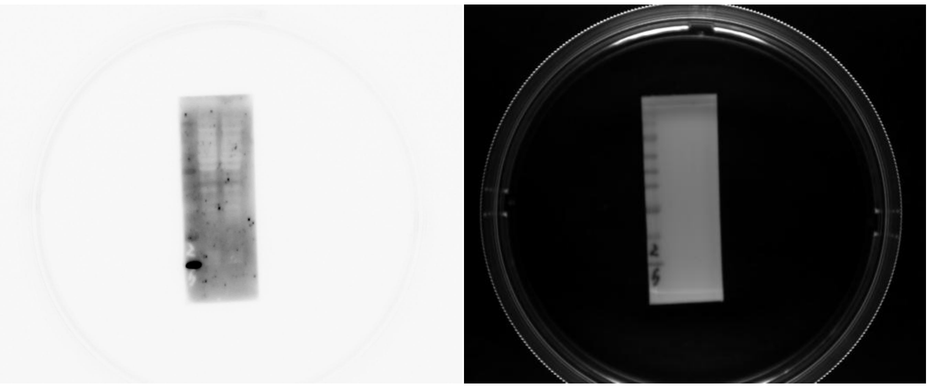

GAPDH (erastin, cytosol)

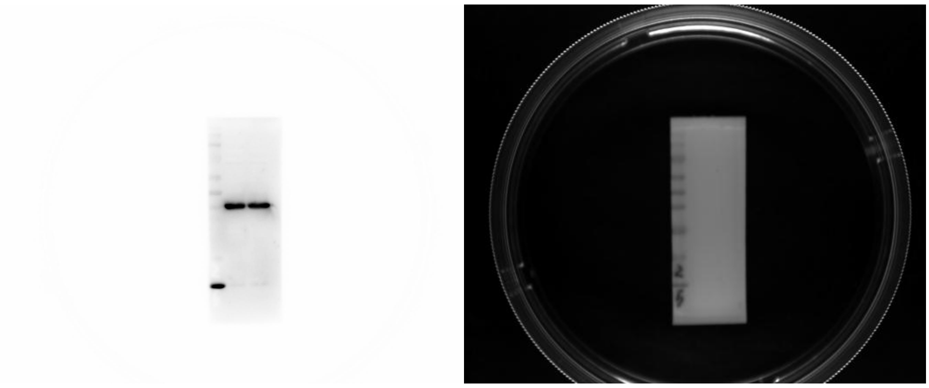

Peroxiredoxin $SO_{2/3}$ (erastin, plasma membrane)

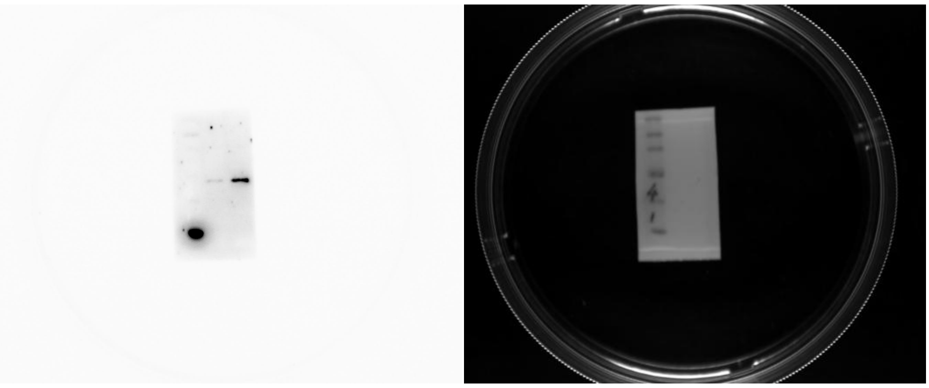

Na^+/K^+ ATPase a1 (erastin, plasma membrane)

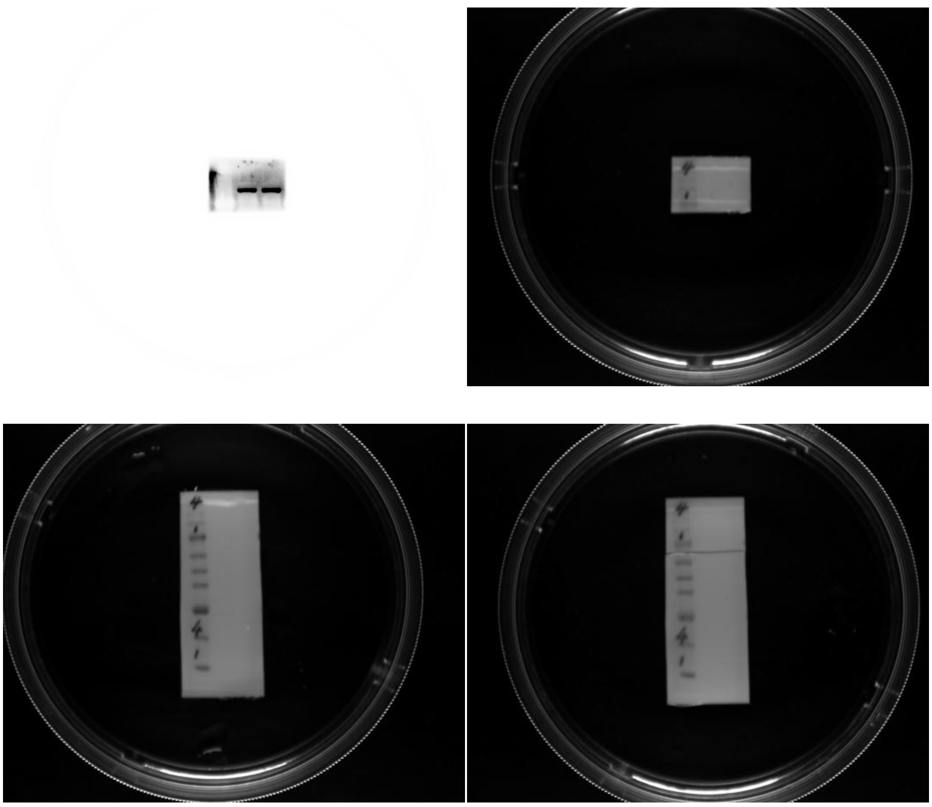

Peroxiredoxin $SO_{2/3}$ (erastin, organelle)

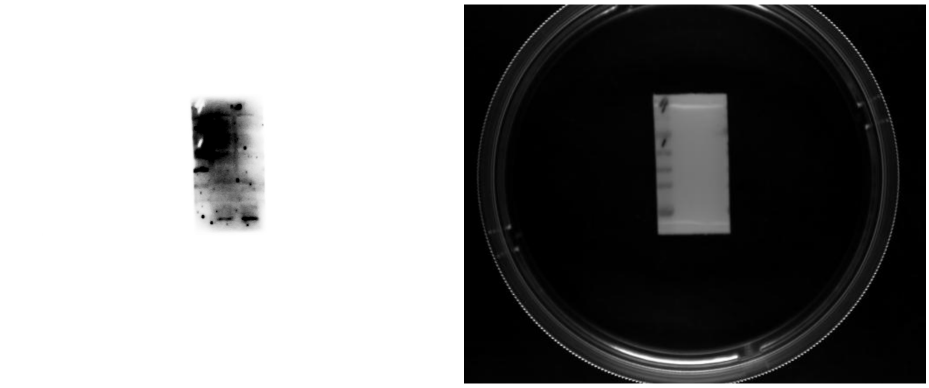

COX IV (erastin, organelle)

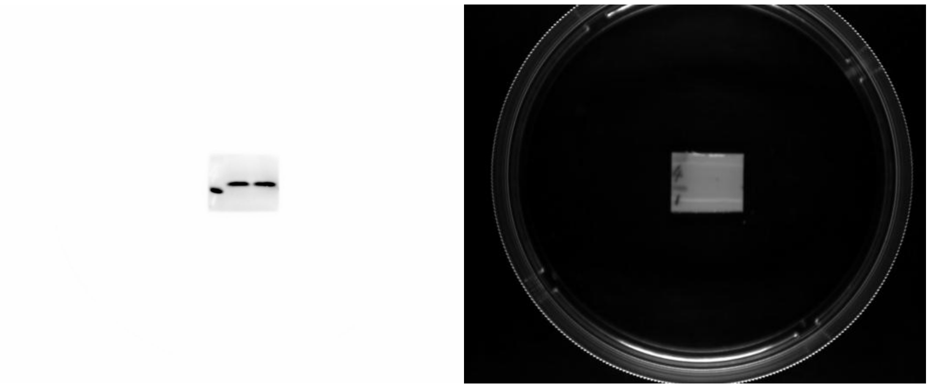

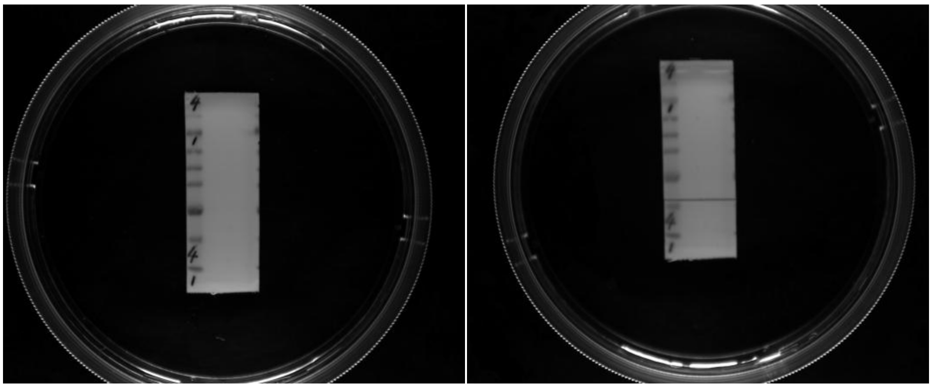

H446DDP

Peroxiredoxin $SO_{2/3}$ (untreated, total)

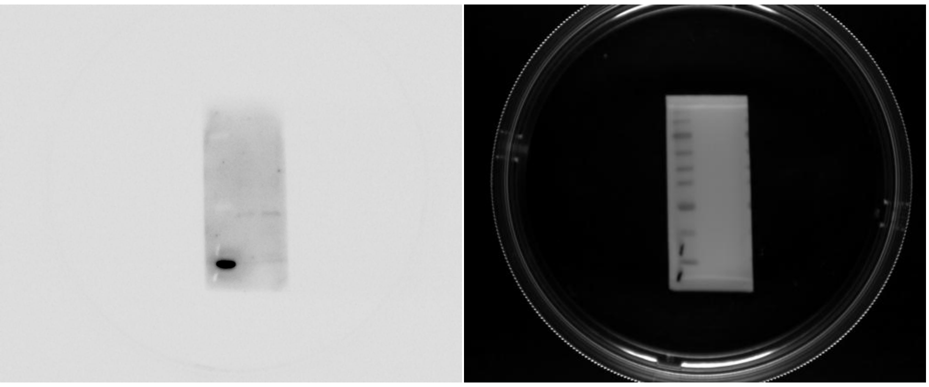

GAPDH (untreated, total)

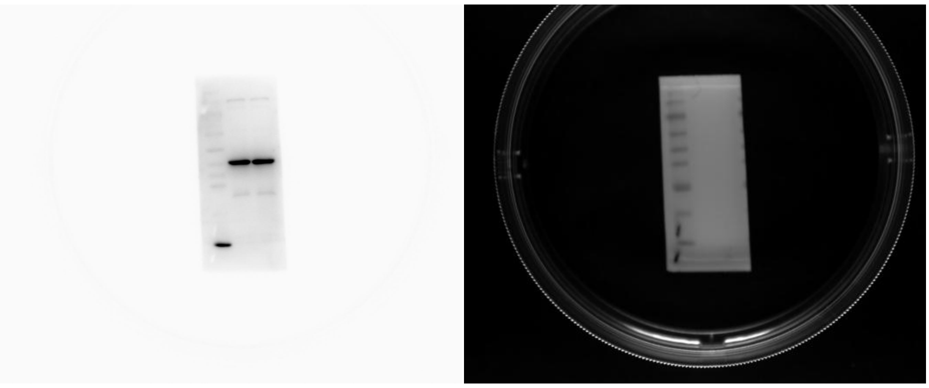

Peroxiredoxin $SO_{2/3}$ (untreated, cytosol)

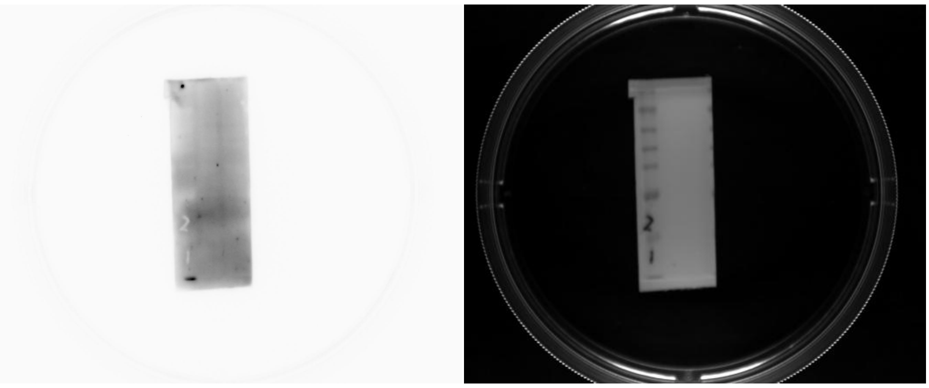

GAPDH (untreated, cytosol)

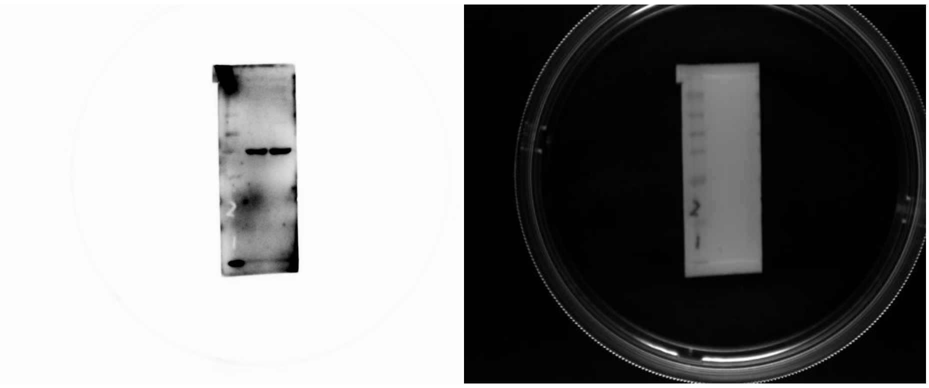

Peroxiredoxin $SO_{2/3}$ (untreated, plasma membrane)

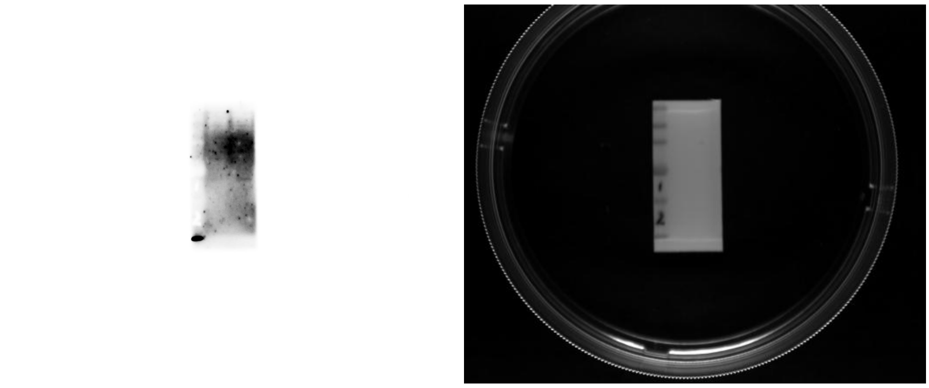

Na^+/K^+ ATPase a1 (untreated, plasma membrane)

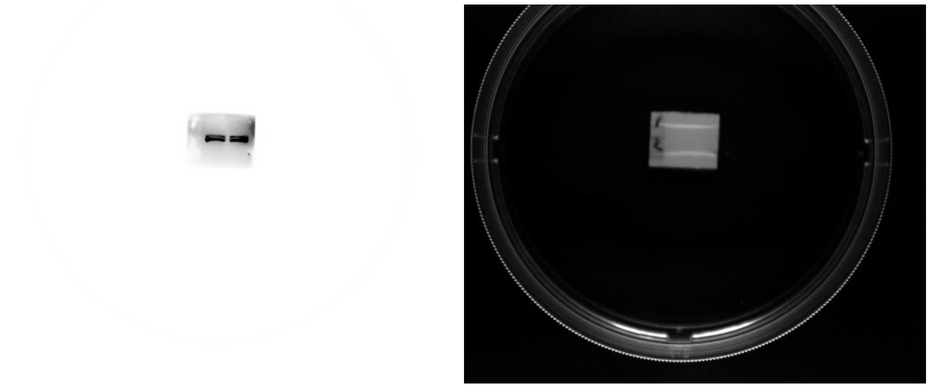

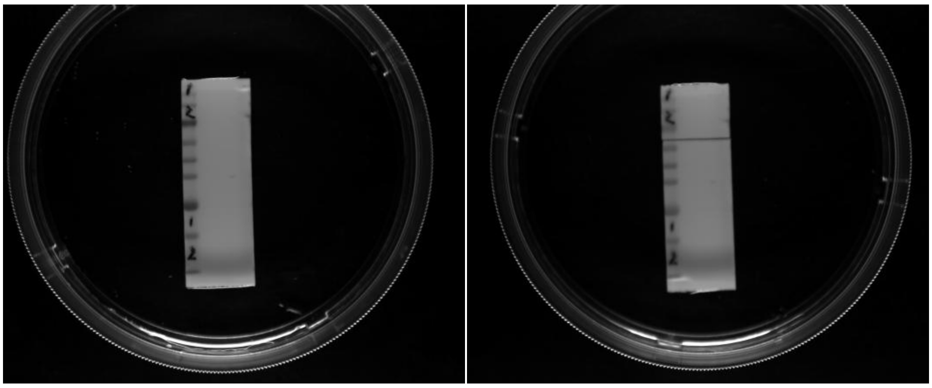

Peroxiredoxin $SO_{2/3}$ (untreated, organelle)

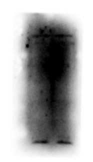
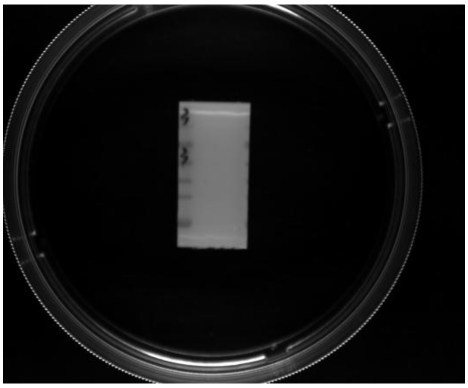

COX IV (untreated, organelle)

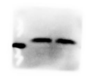
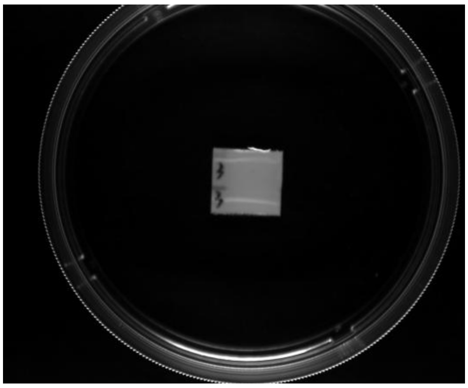

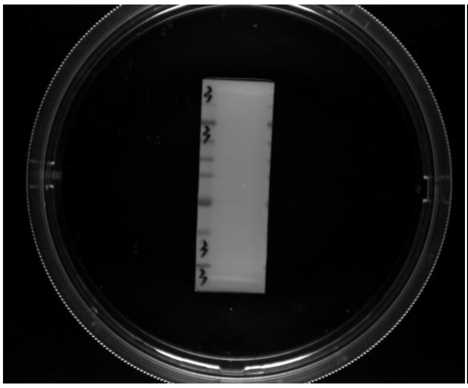
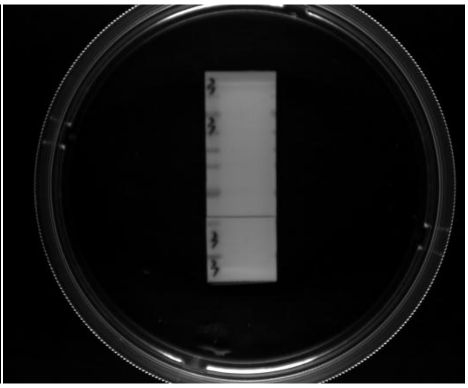

Peroxiredoxin $SO_{2/3}$ (cisplatin, total)

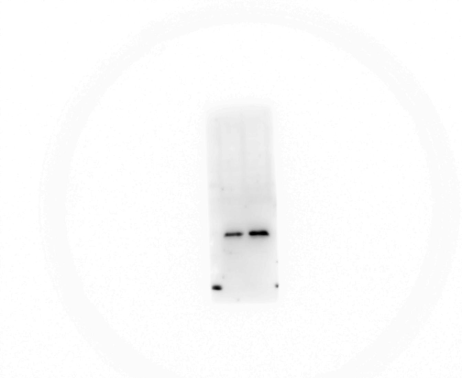
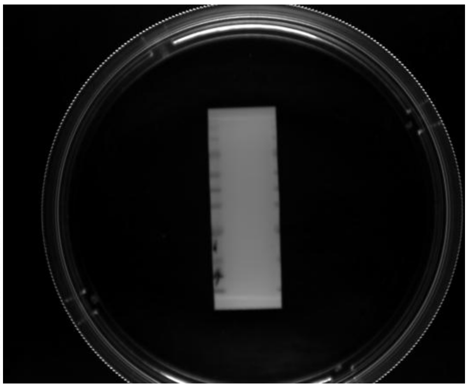

GAPDH (cisplatin, total)

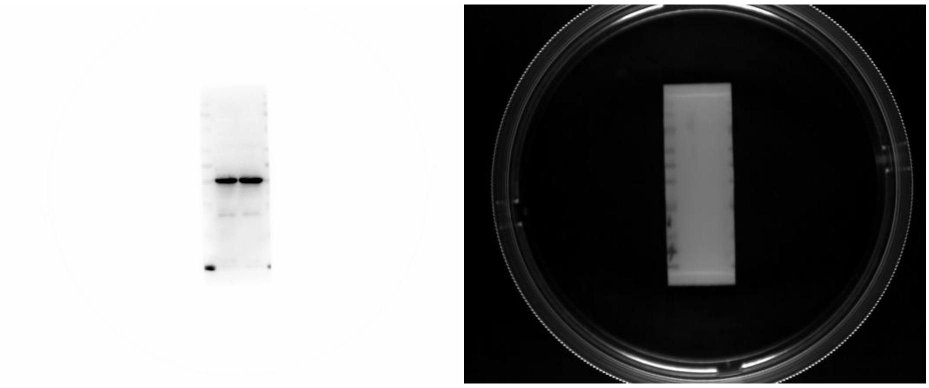

Peroxiredoxin $SO_{2/3}$ (cisplatin, cytosol)

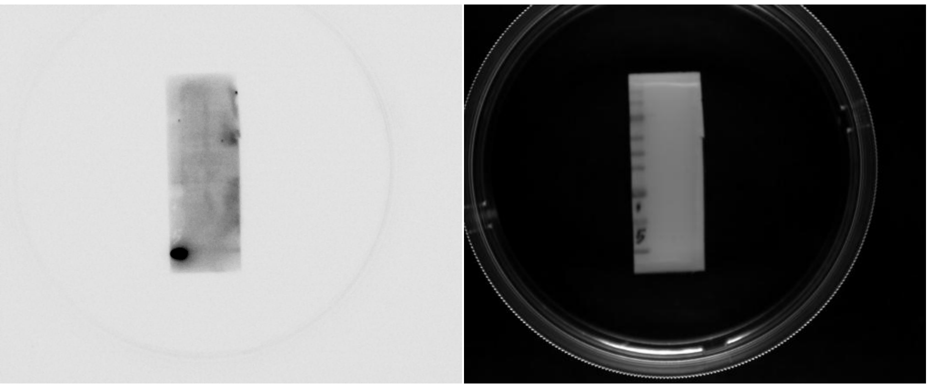

GAPDH (cisplatin, cytosol)

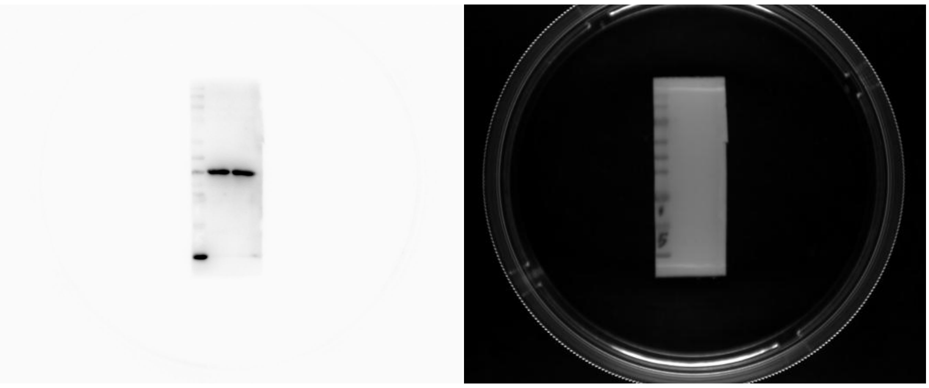

Peroxiredoxin $SO_{2/3}$ (cisplatin, plasma membrane)

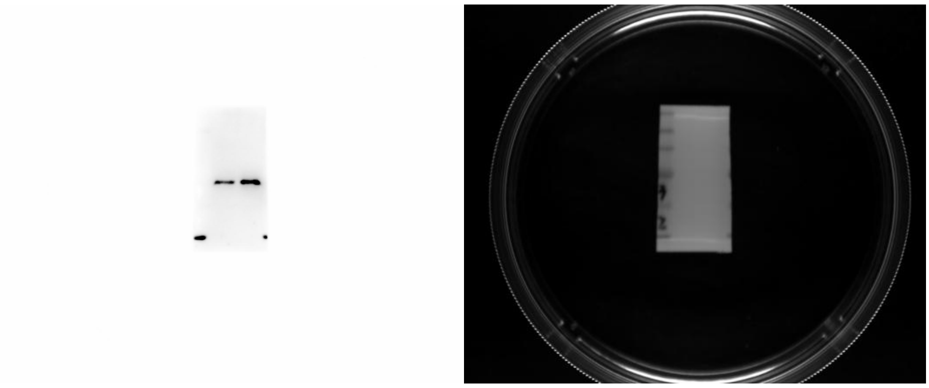

Na^+/K^+ ATPase a1 (cisplatin, plasma membrane)

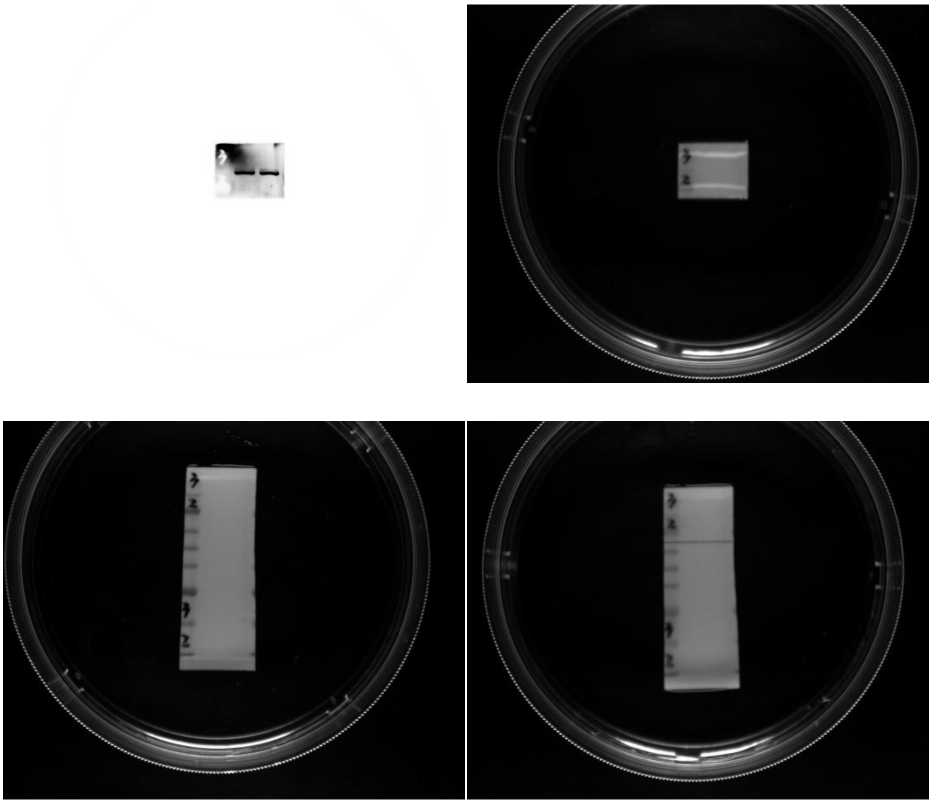

Peroxiredoxin $SO_{2/3}$ (cisplatin, organelle)

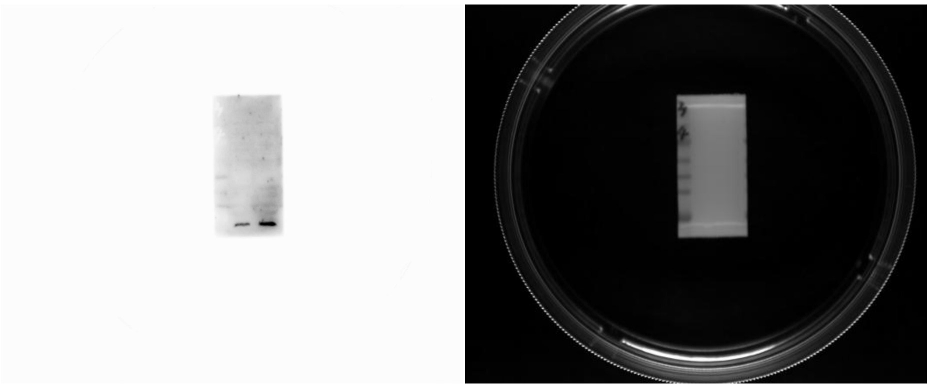

COX IV (cisplatin, organelle)

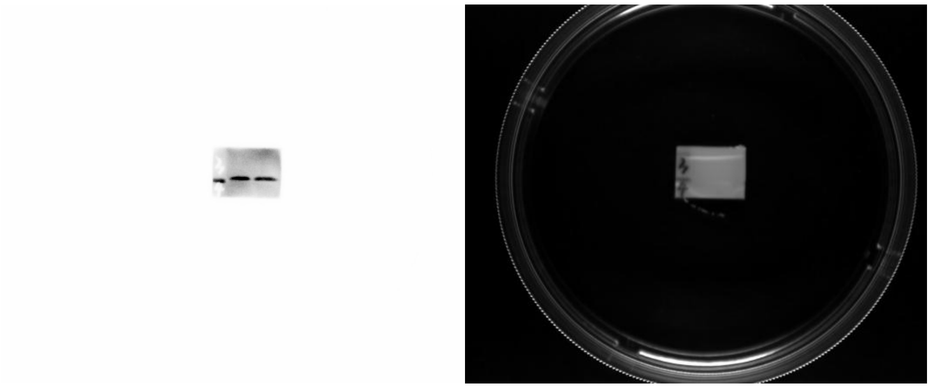

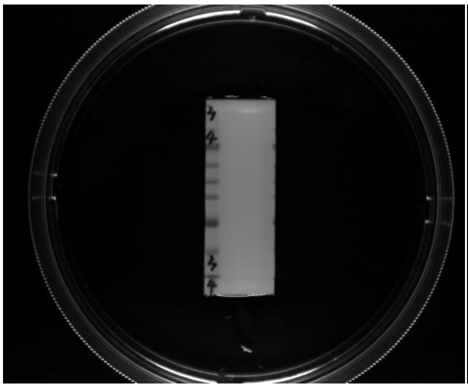
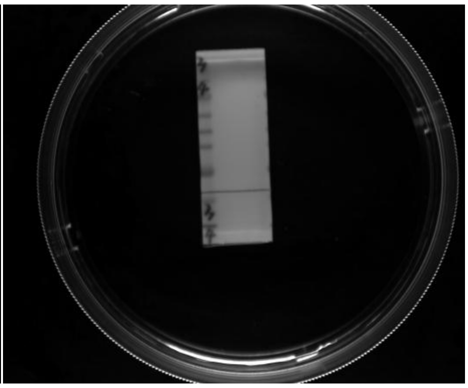

Peroxiredoxin $SO_{2/3}$ (erastin, total)

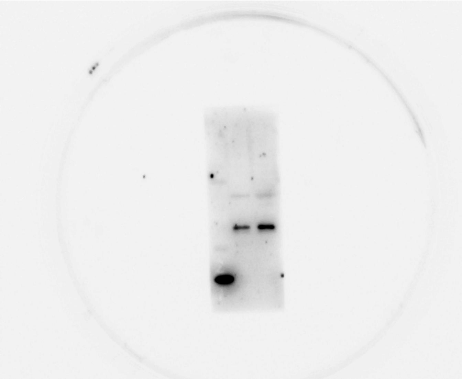
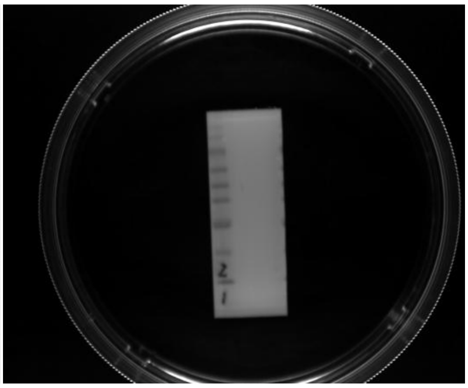

GAPDH (erastin, total)

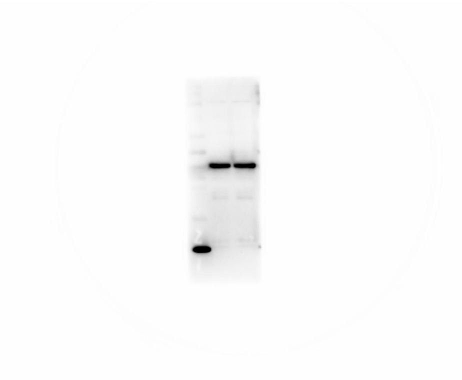
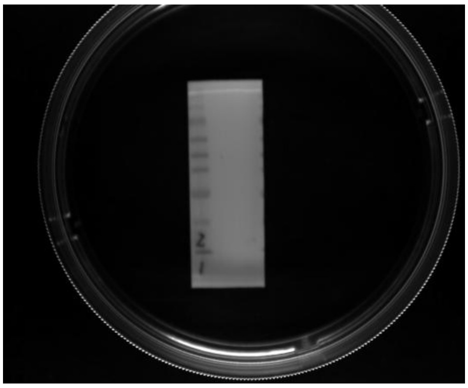

Peroxiredoxin $SO_{2/3}$ (erastin, cytosol)

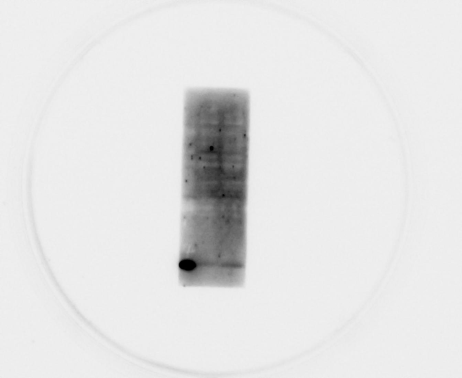
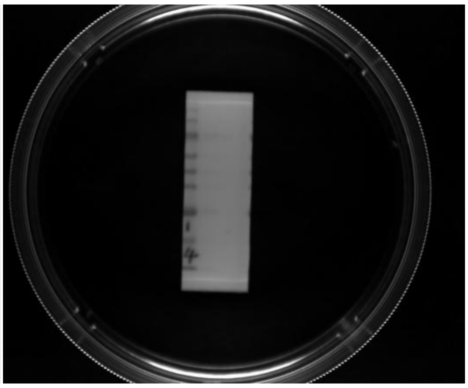

GAPDH (erastin, cytosol)

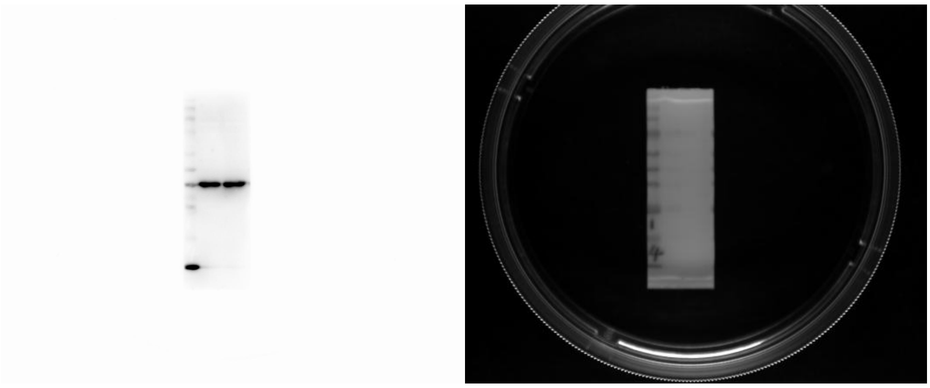

Peroxiredoxin $SO_{2/3}$ (erastin, plasma membrane)

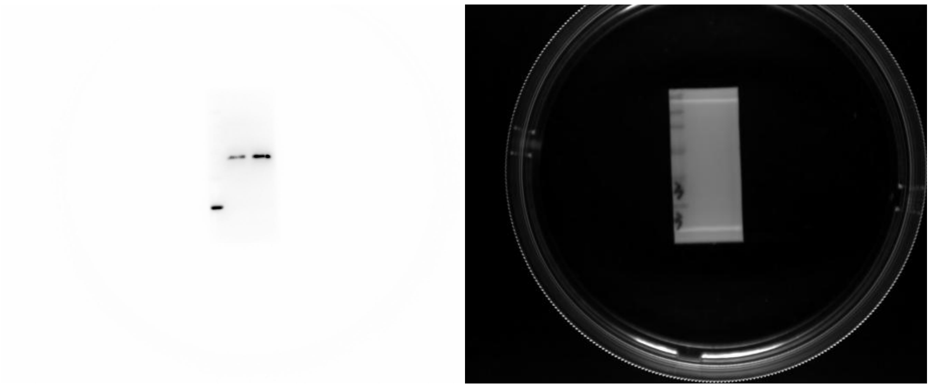

Na^{+}/K^{+} ATPase a1 (erastin, plasma membrane)

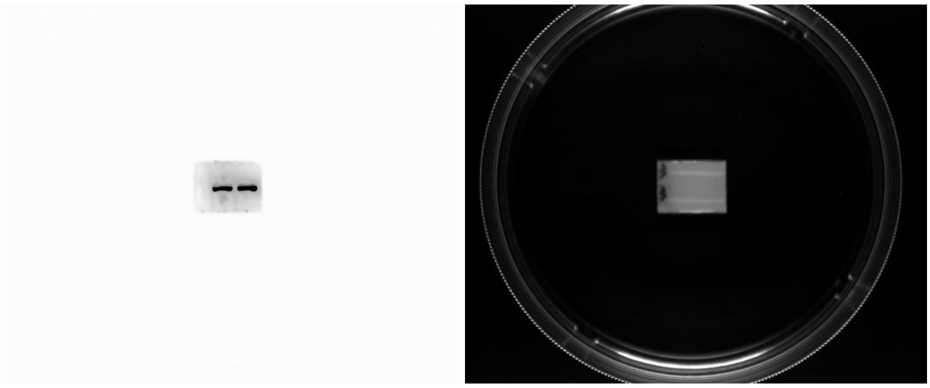

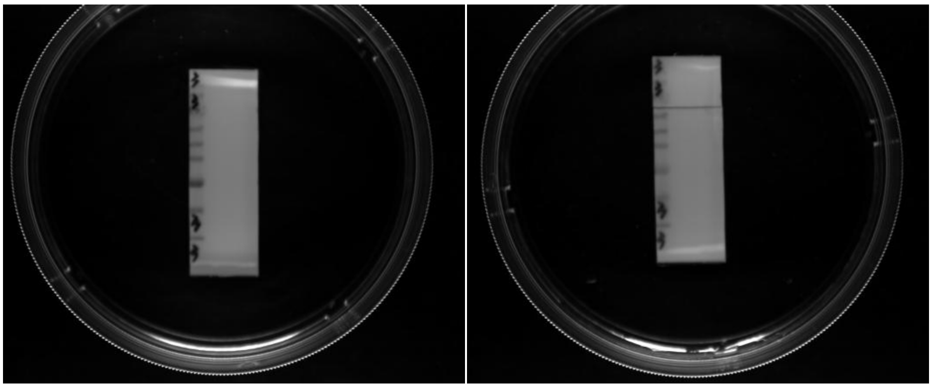

Peroxiredoxin $SO_{2/3}$ (erastin, organelle)

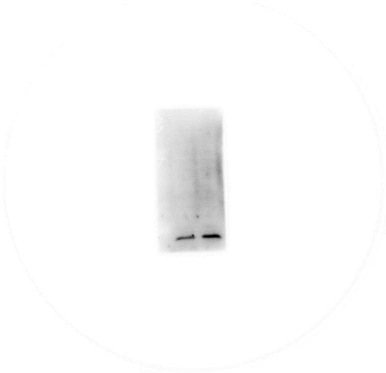

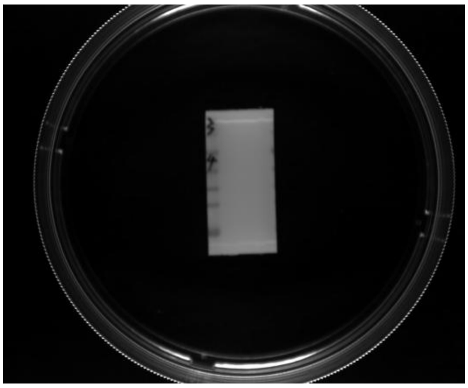

COX IV (erastin, organelle)

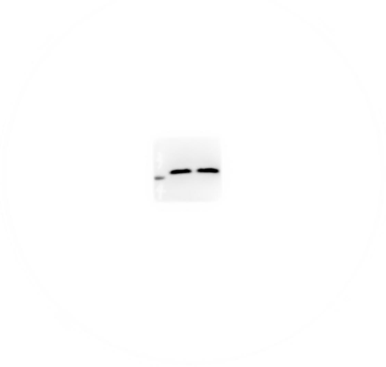

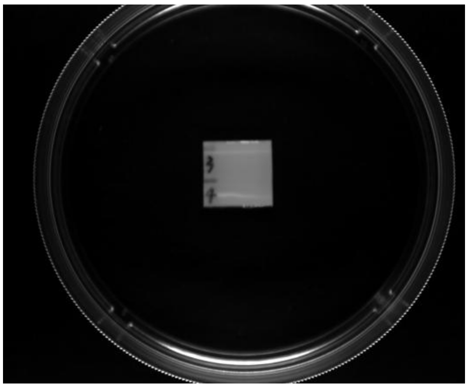

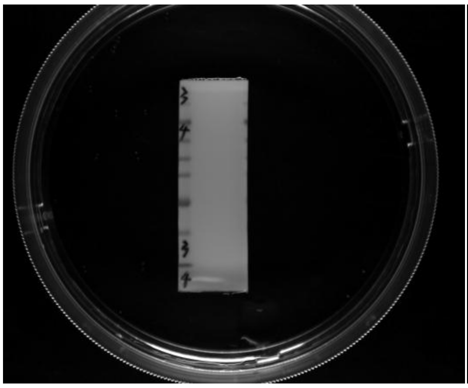

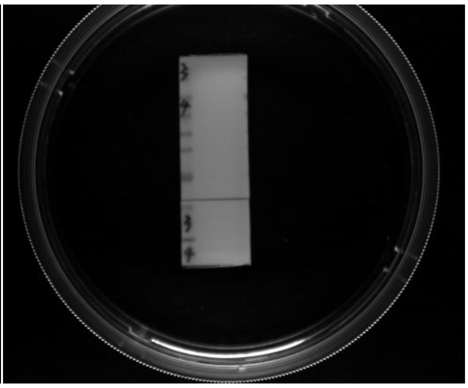

Fig. 7F Second Repetition

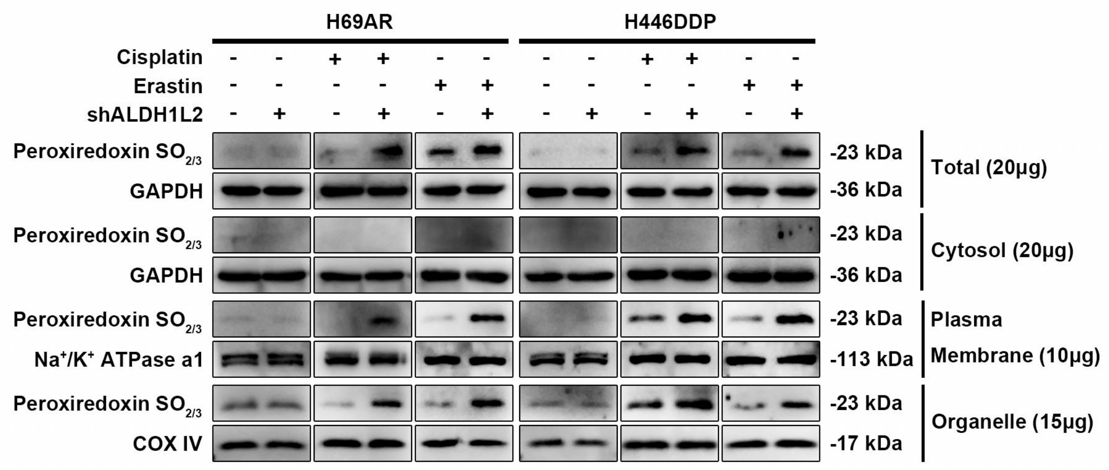

H69AR

Peroxiredoxin $SO_{2/3}$ (untreated, total)

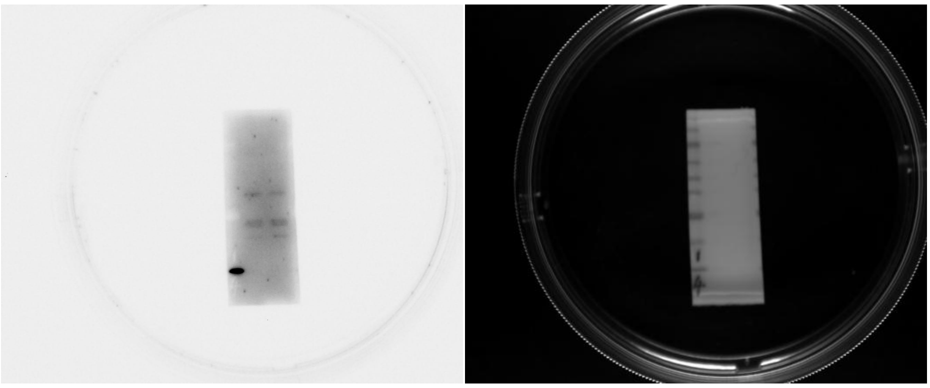

GAPDH (untreated, total)

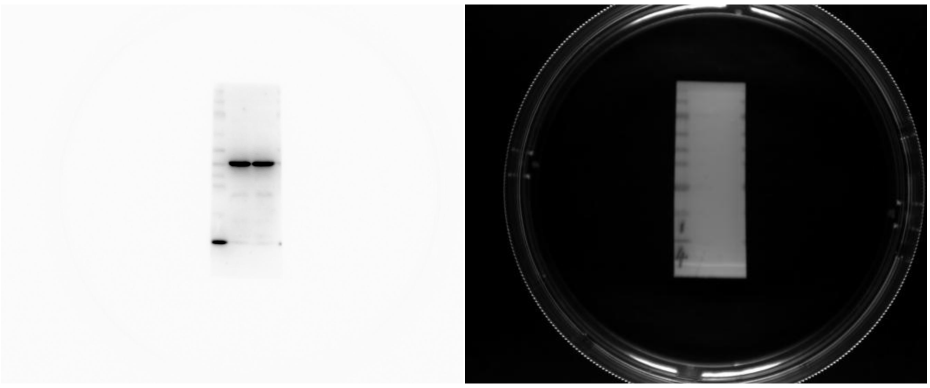

Peroxiredoxin $SO_{2/3}$ (untreated, cytosol)

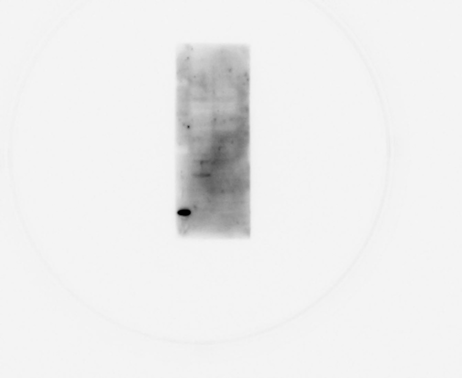
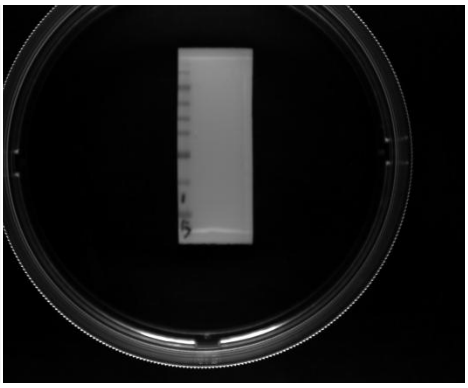

GAPDH (untreated, cytosol)

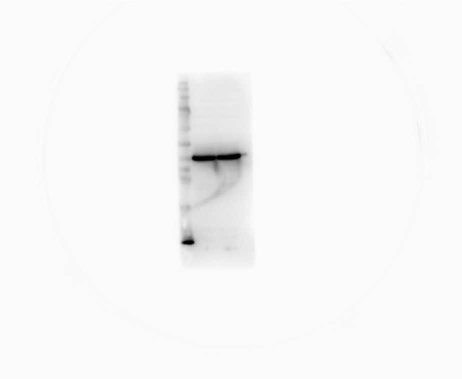
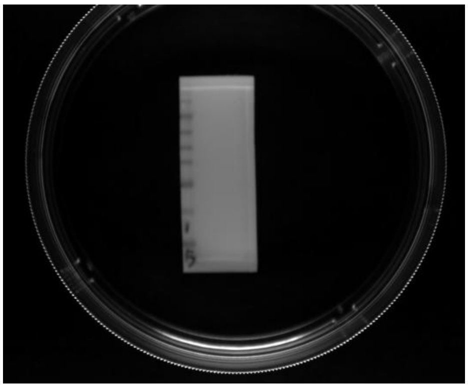

Peroxiredoxin $SO_{2/3}$ (untreated, plasma membrane)

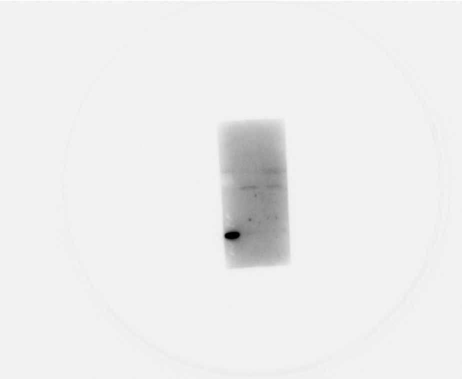
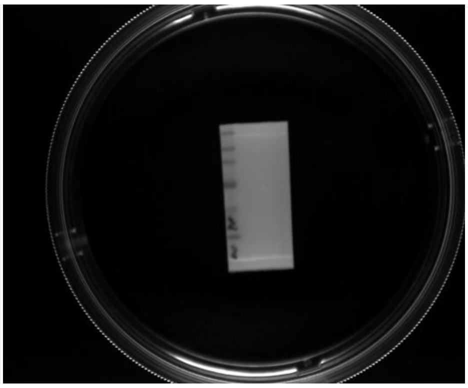

Na^+/K^+ ATPase a1 (untreated, plasma membrane)

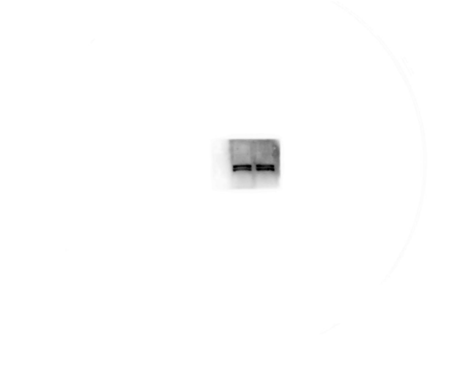
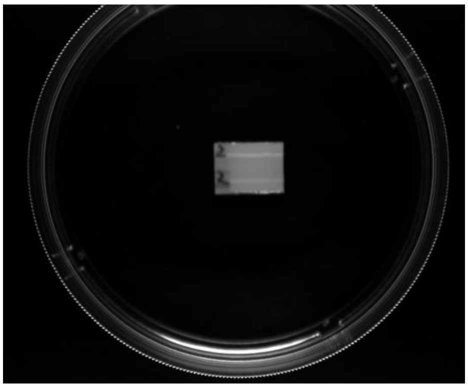

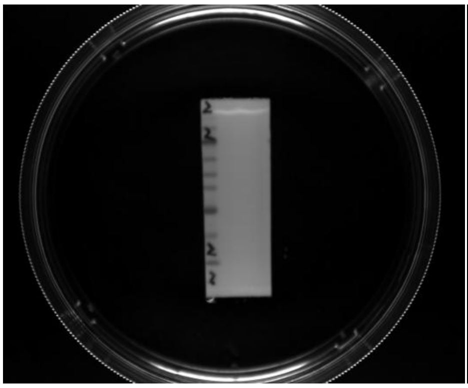
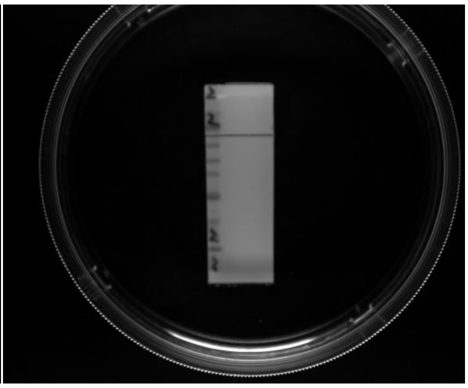

Peroxiredoxin $SO_{2/3}$ (untreated, organelle)

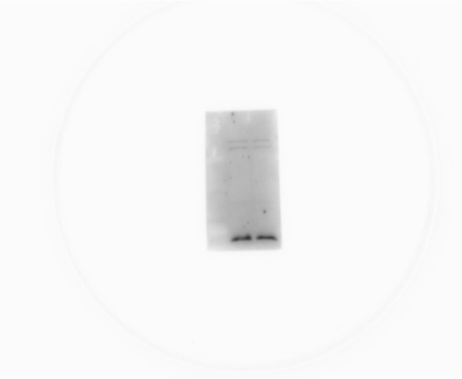
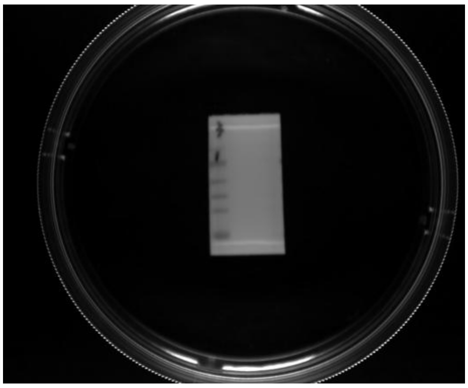

COX IV (untreated, organelle)

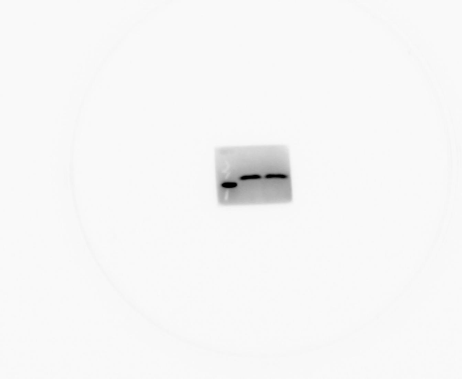
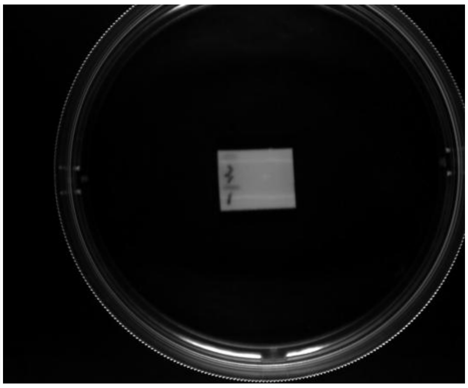

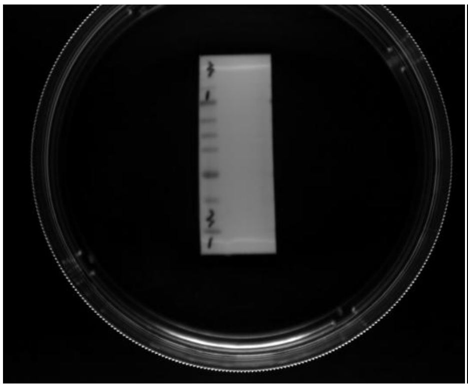
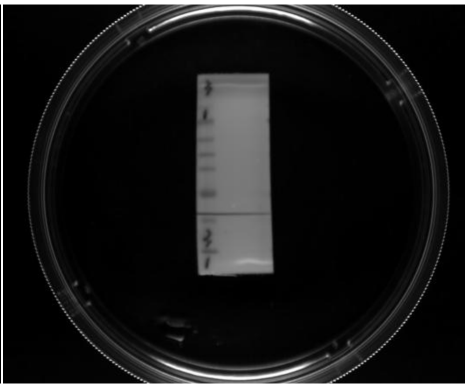

Peroxiredoxin $SO_{2/3}$ (cisplatin, total)

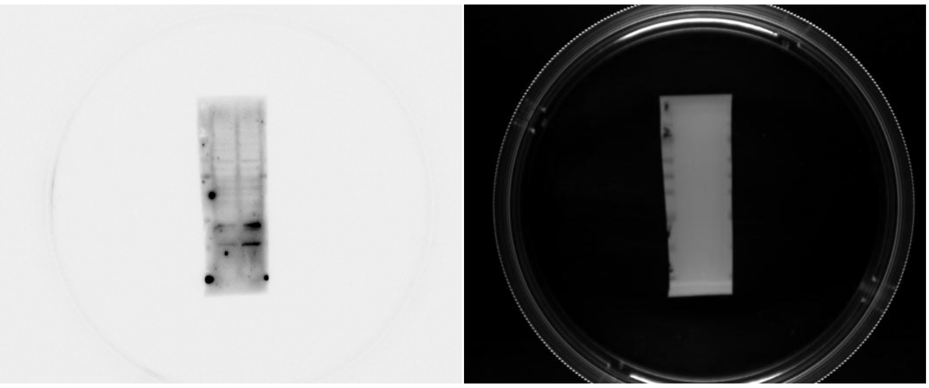

GAPDH (cisplatin, total)

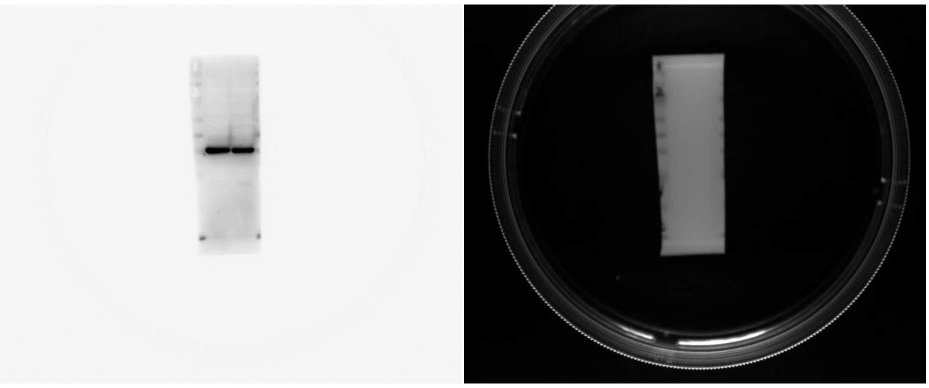

Peroxiredoxin $SO_{2/3}$ (cisplatin, cytosol)

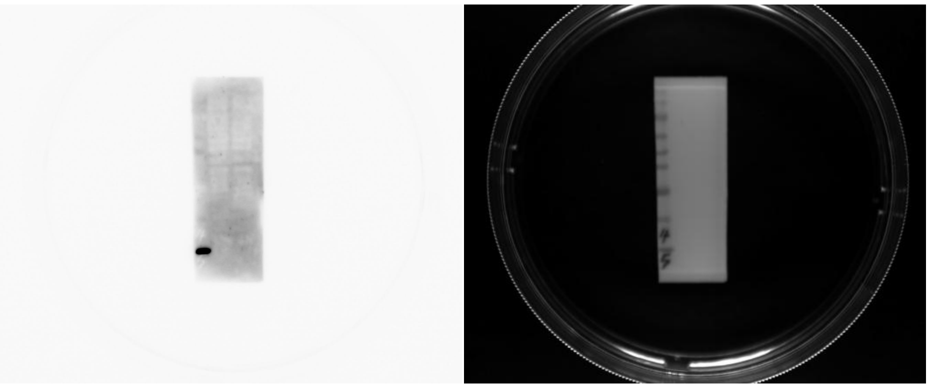

GAPDH (cisplatin, cytosol)

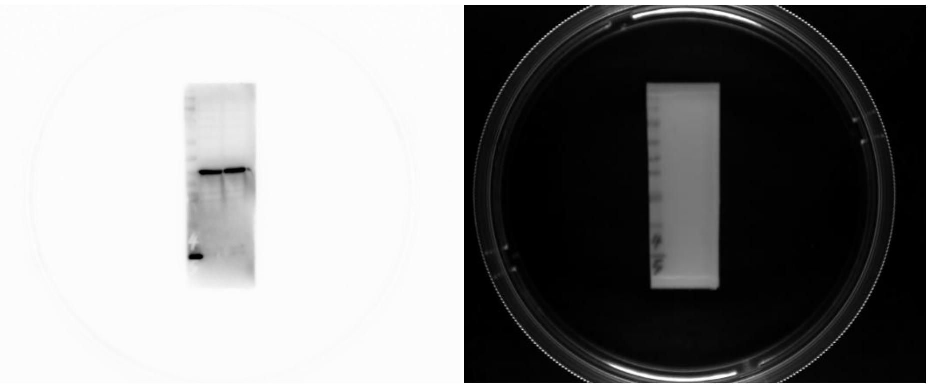

Peroxiredoxin $SO_{2/3}$ (cisplatin, plasma membrane)

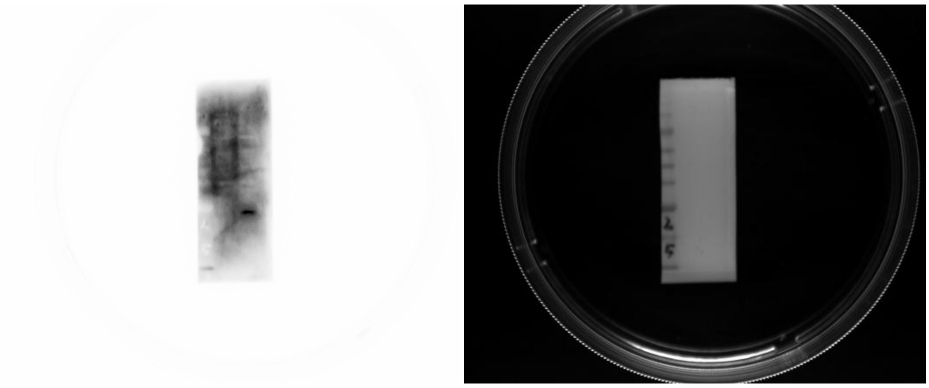

Na^+/K^+ ATPase a1 (cisplatin, plasma membrane)

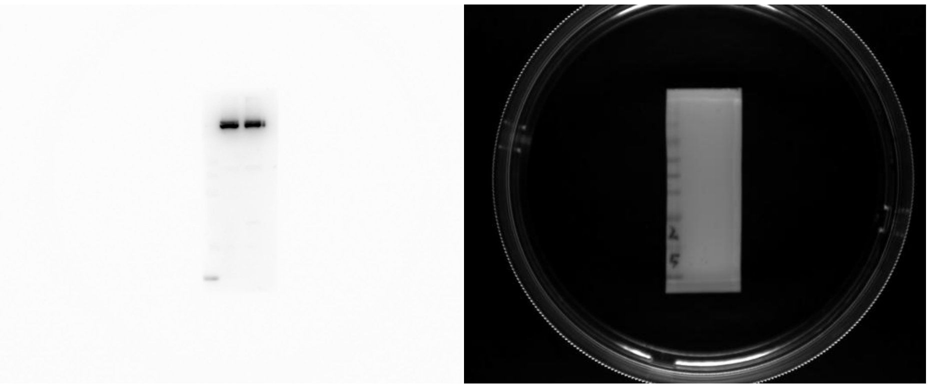

Peroxiredoxin $SO_{2/3}$ (cisplatin, organelle)

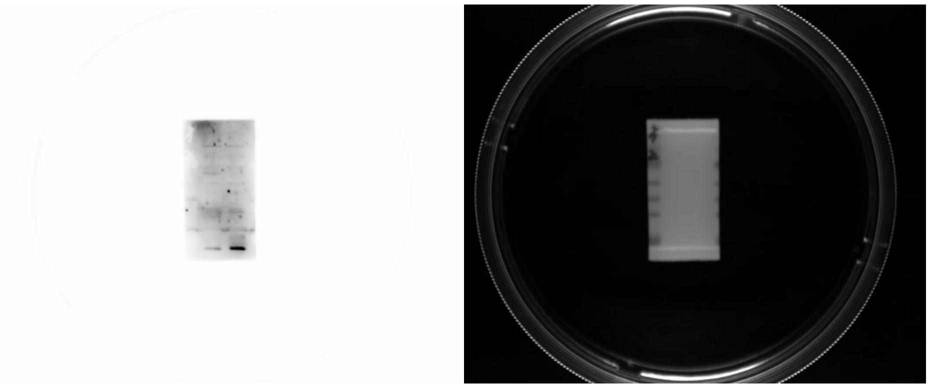

COX IV (cisplatin, organelle)

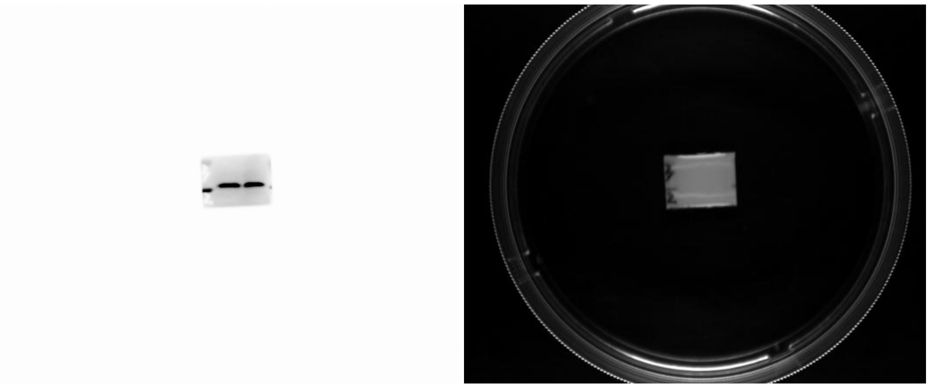

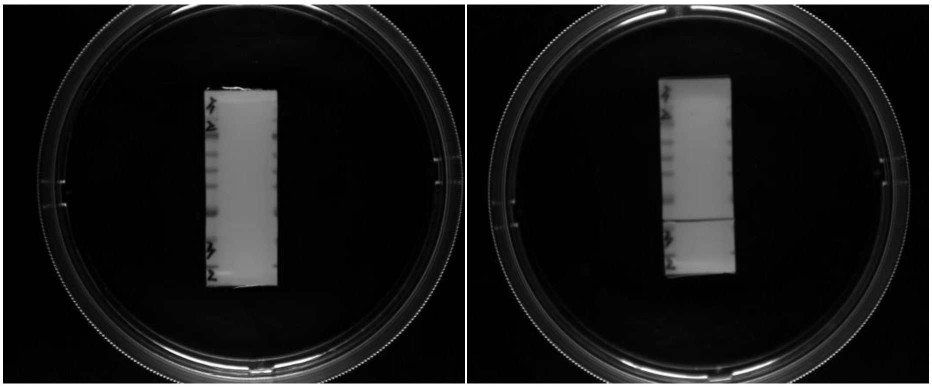

Peroxiredoxin $SO_{2/3}$ (erastin, total)

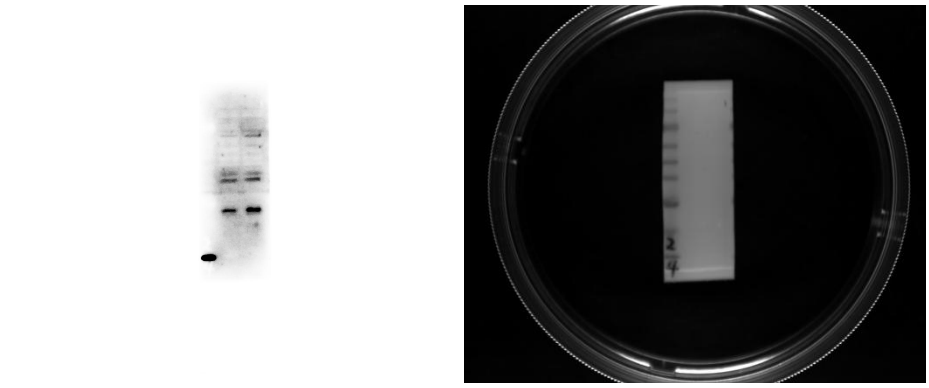

GAPDH (erastin, total)

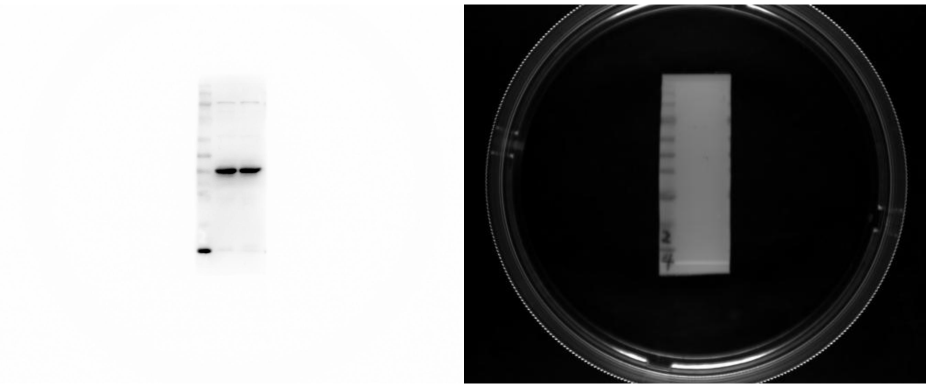

Peroxiredoxin $SO_{2/3}$ (erastin, cytosol)

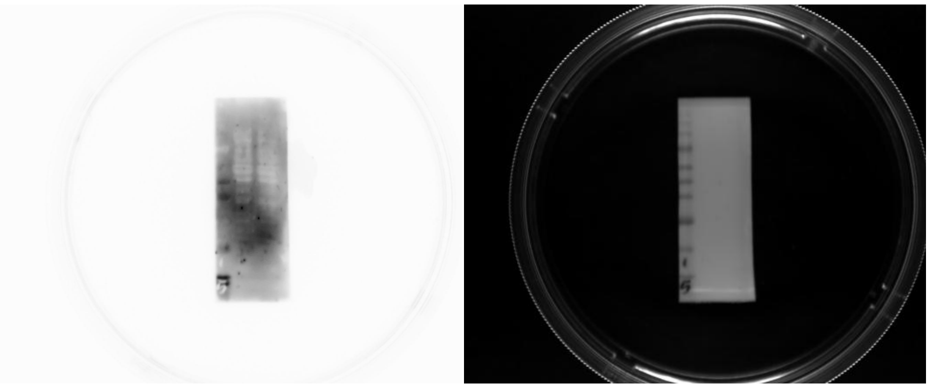

GAPDH (erastin, cytosol)

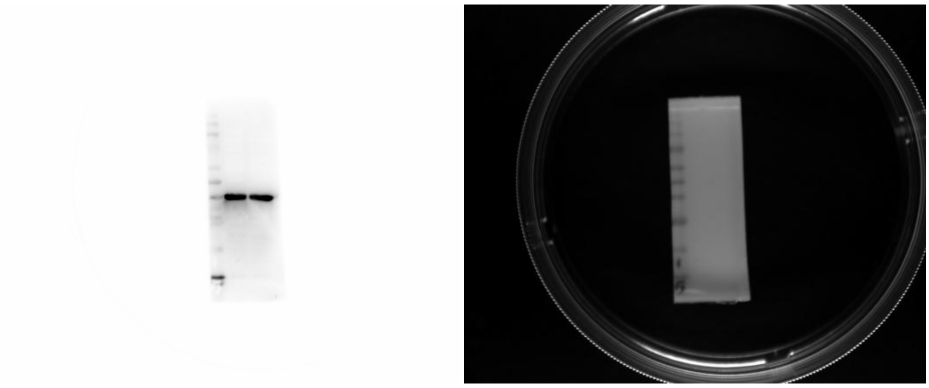

Peroxiredoxin $SO_{2/3}$ (erastin, plasma membrane)

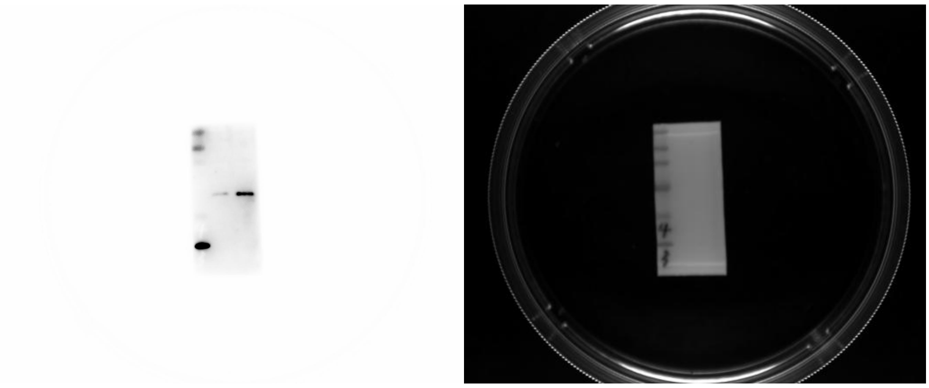

Na^{+}/K^{+} ATPase a1 (erastin, plasma membrane)

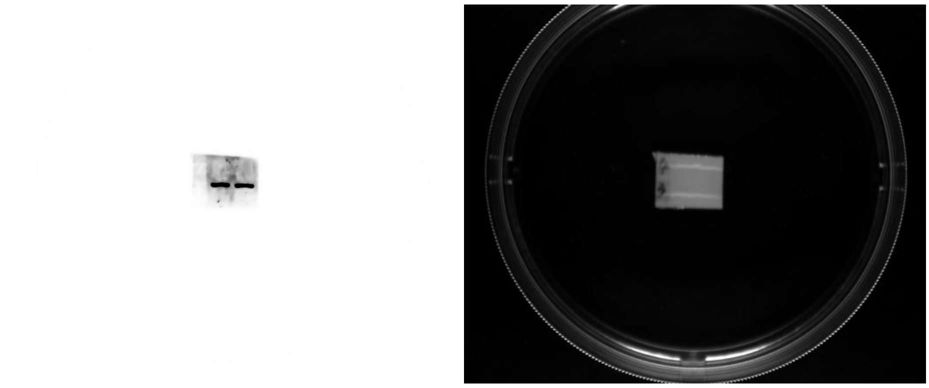

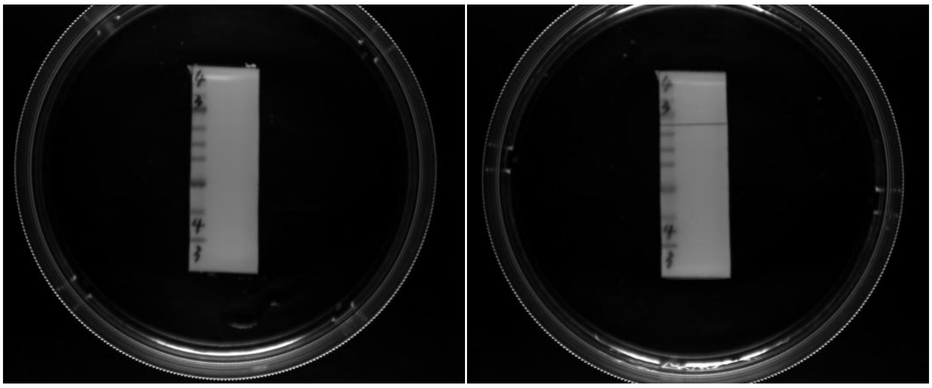

Peroxiredoxin $SO_{2/3}$ (erastin, organelle)

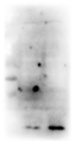

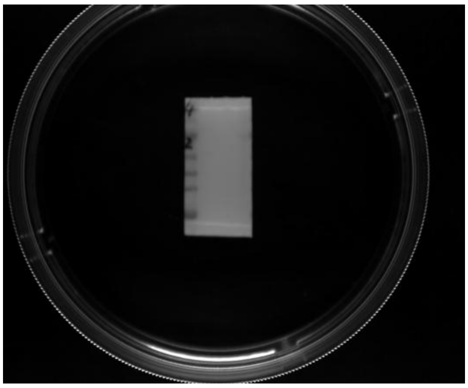

COX IV (erastin, organelle)

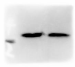

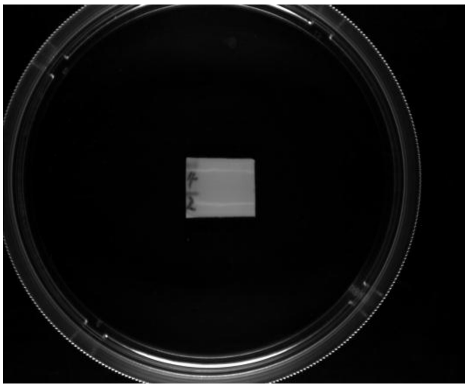

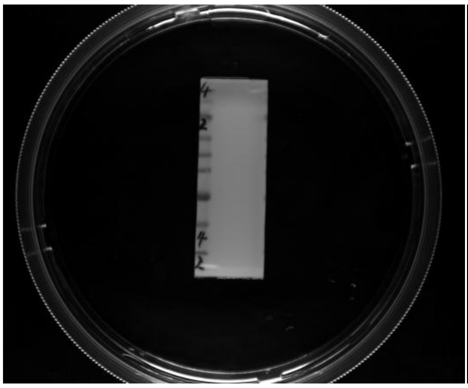

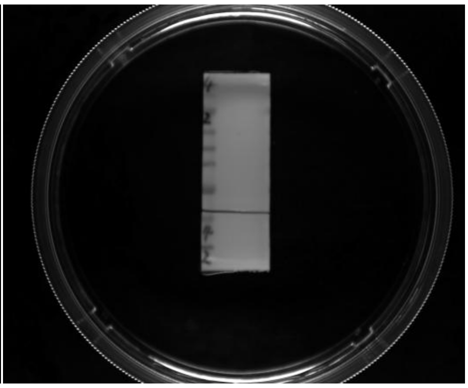

H446DDP

Peroxiredoxin $SO_{2/3}$ (untreated, total)

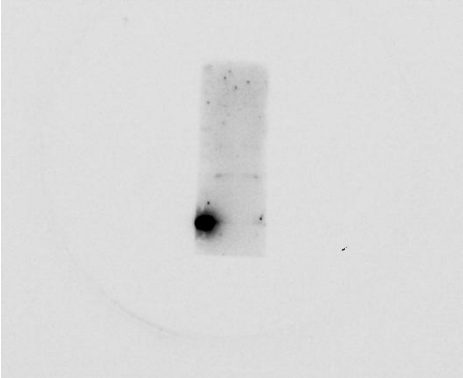

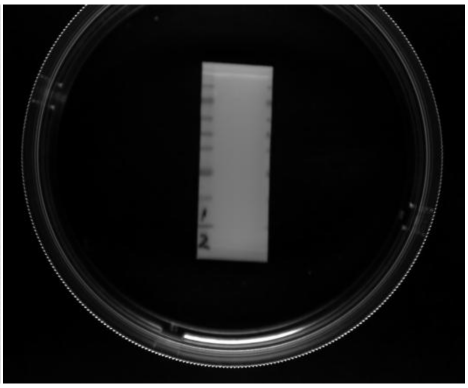

GAPDH (untreated, total)

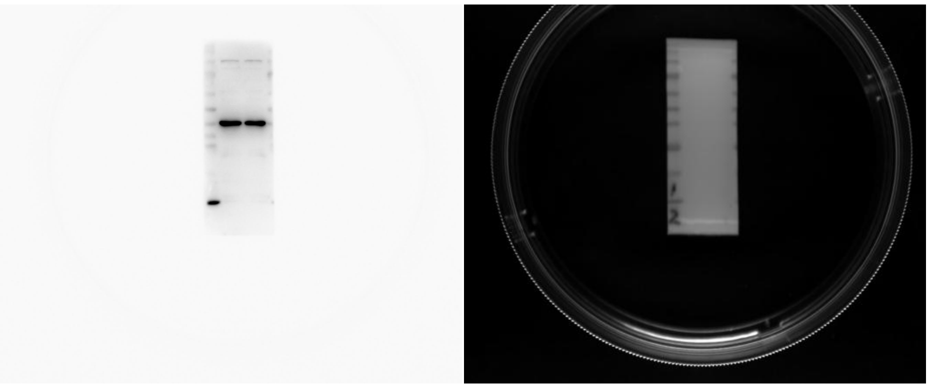

Peroxiredoxin $SO_{2/3}$ (untreated, cytosol)

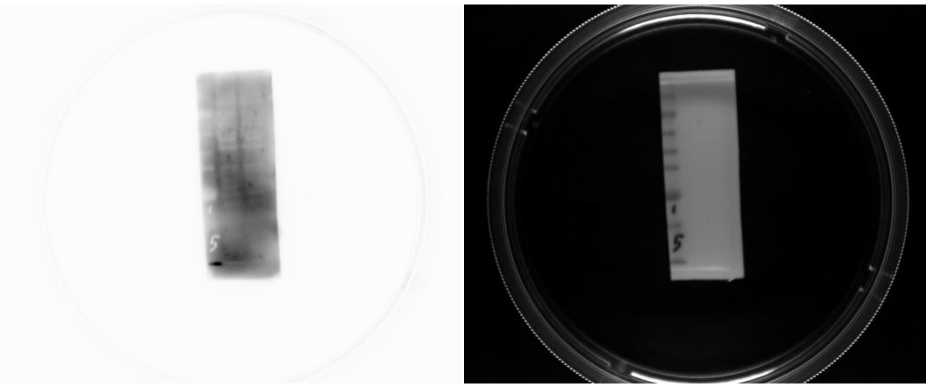

GAPDH (untreated, cytosol)

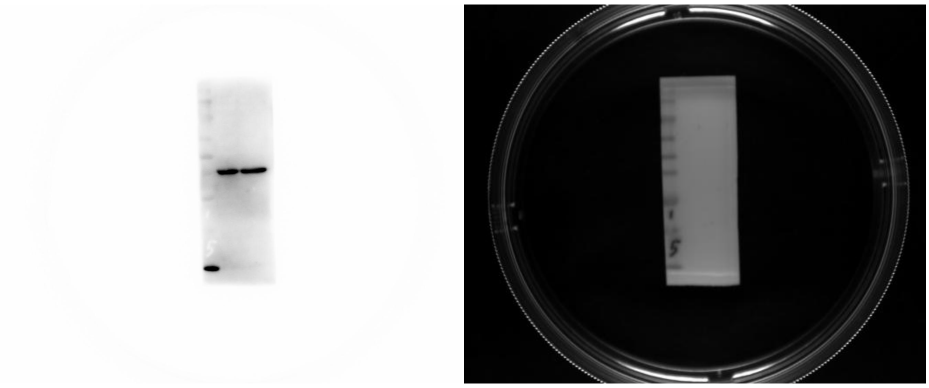

Peroxiredoxin $SO_{2/3}$ (untreated, plasma membrane)

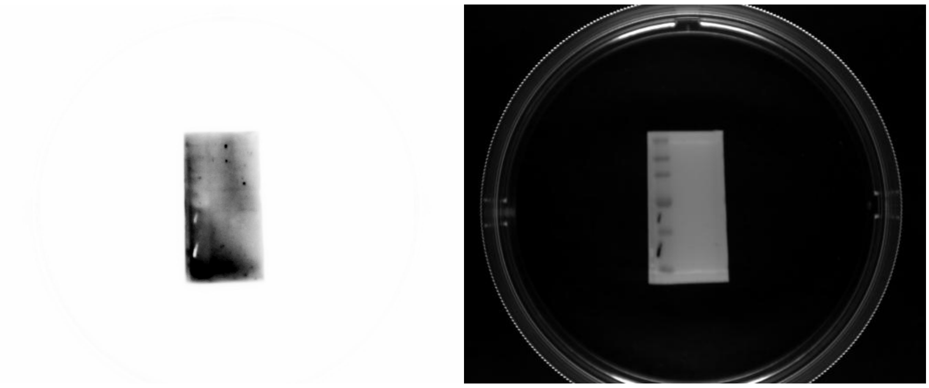

Na^+/K^+ ATPase a1 (untreated, plasma membrane)

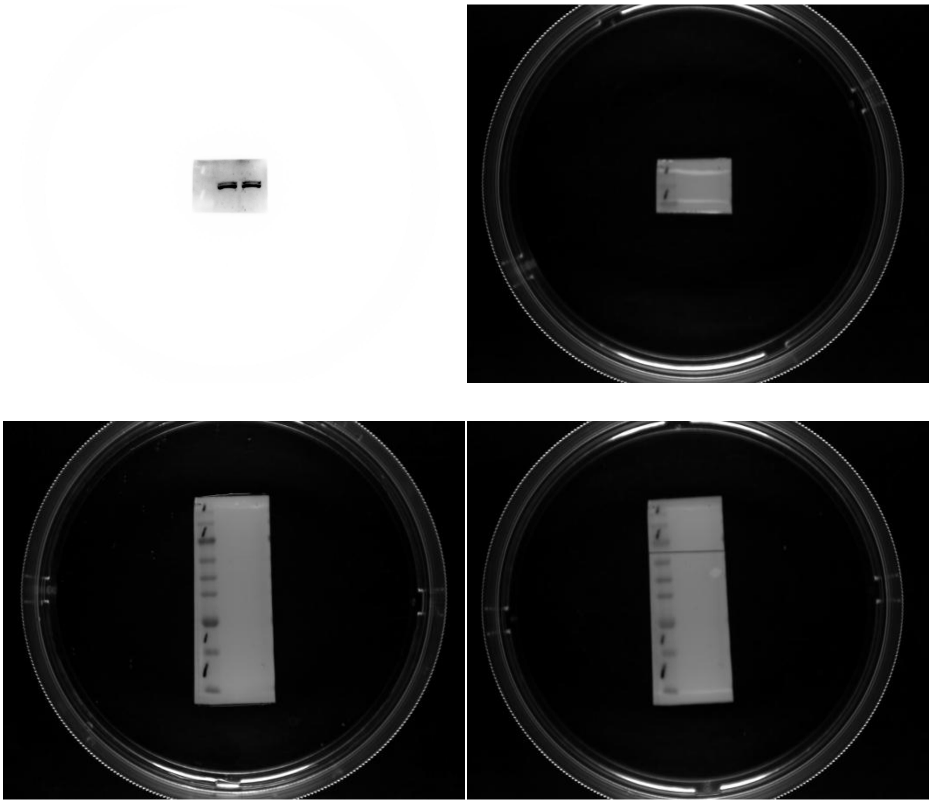

Peroxiredoxin $SO_{2/3}$ (untreated, organelle)

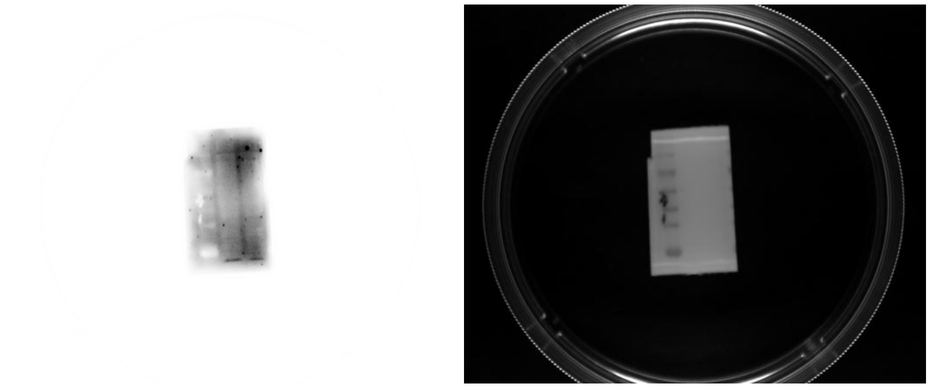

COX IV (untreated, organelle)

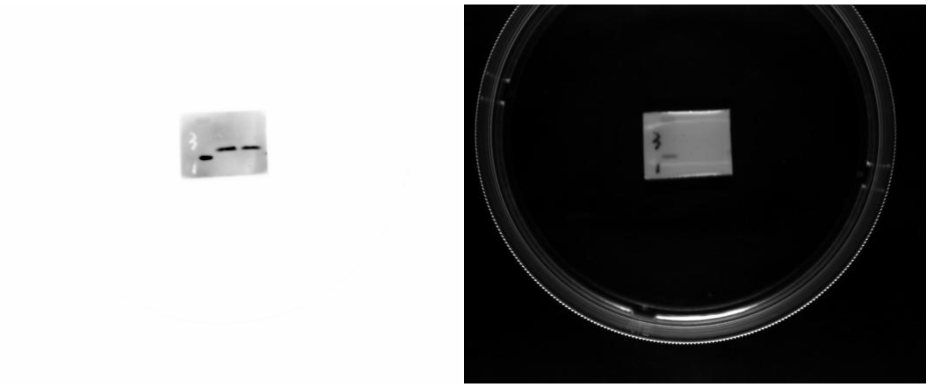

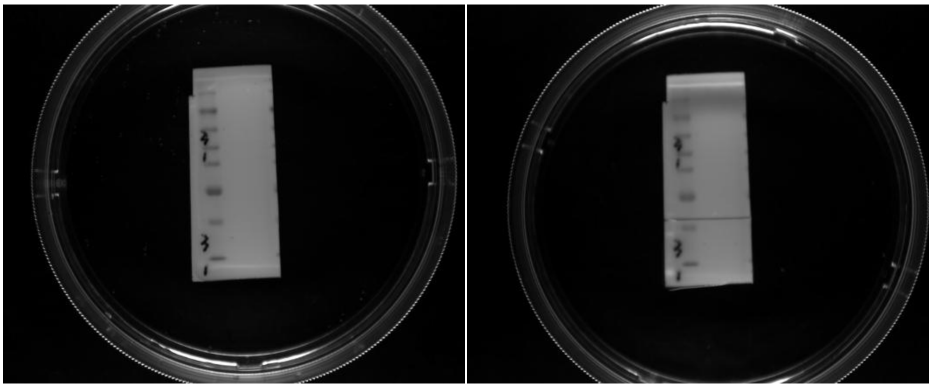

Peroxiredoxin $SO_{2/3}$ (cisplatin, total)

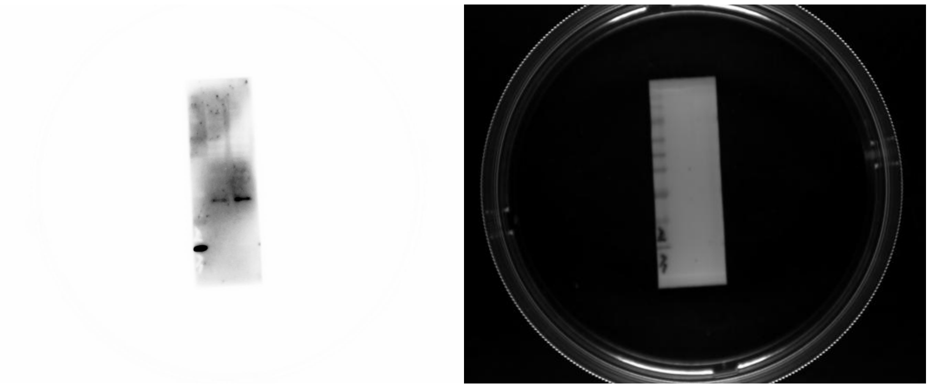

GAPDH (cisplatin, total)

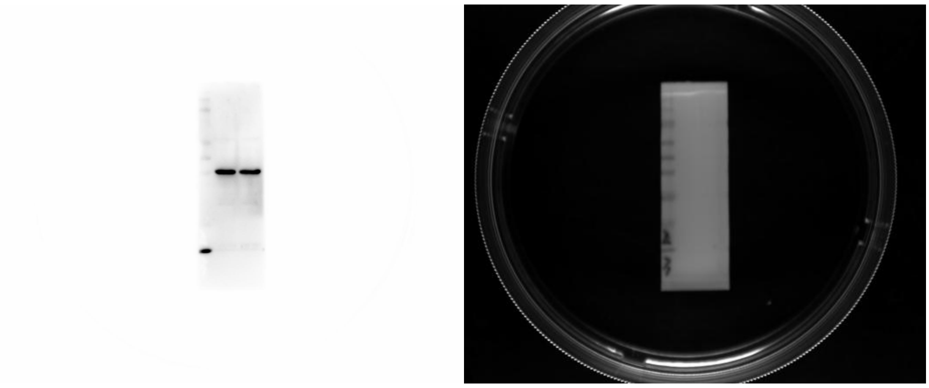

Peroxiredoxin $SO_{2/3}$ (cisplatin, cytosol)

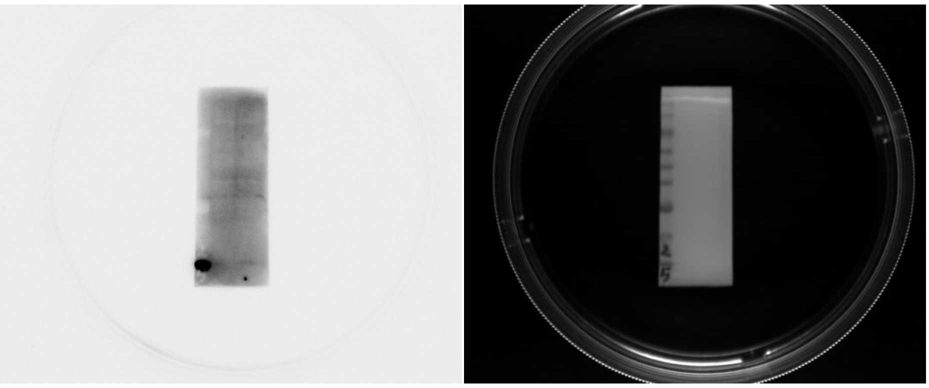

GAPDH (cisplatin, cytosol)

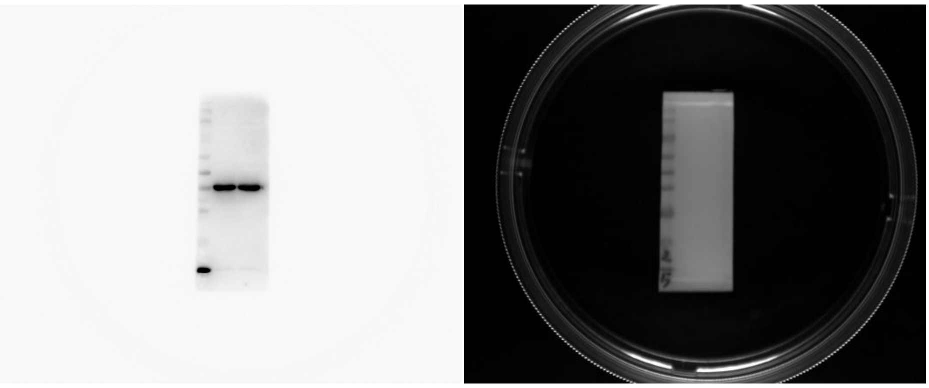

Peroxiredoxin $SO_{2/3}$ (cisplatin, plasma membrane)

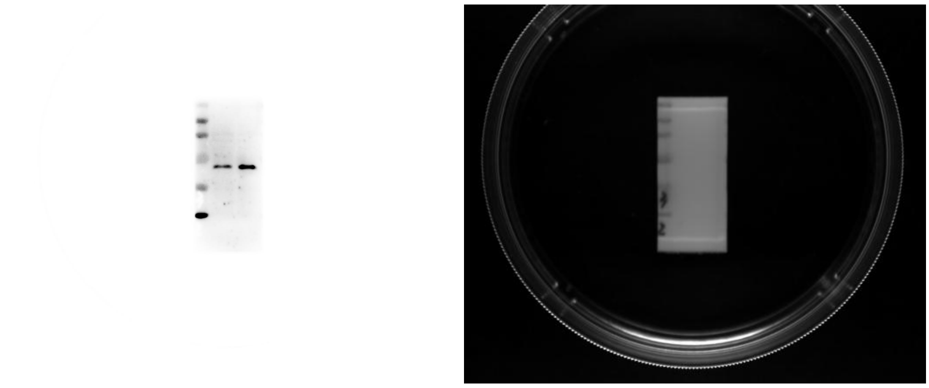

Na^+/K^+ ATPase a1 (cisplatin, plasma membrane)

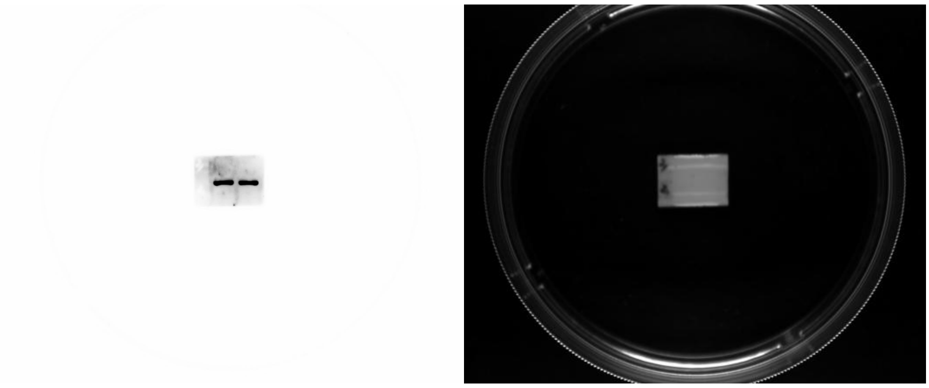

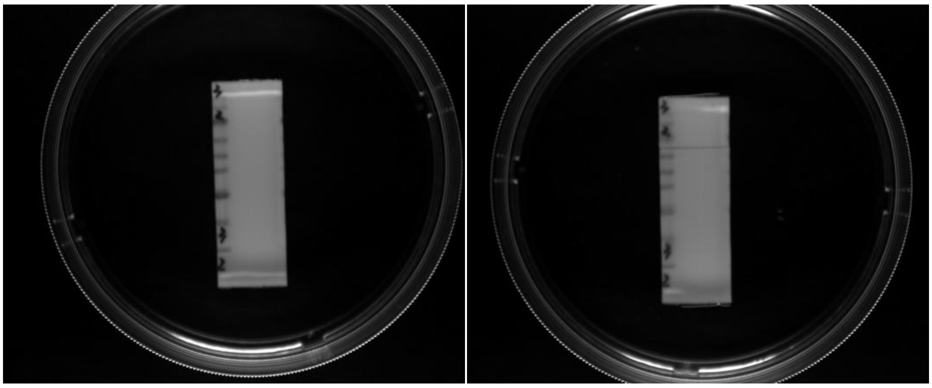

Peroxiredoxin $SO_{2/3}$ (cisplatin, organelle)

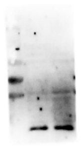

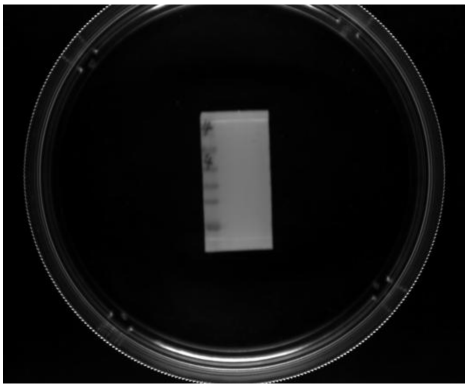

COX IV (cisplatin, organelle)

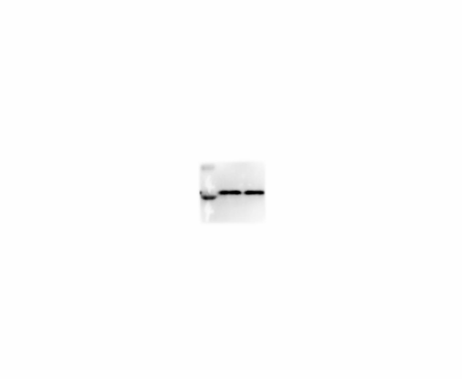

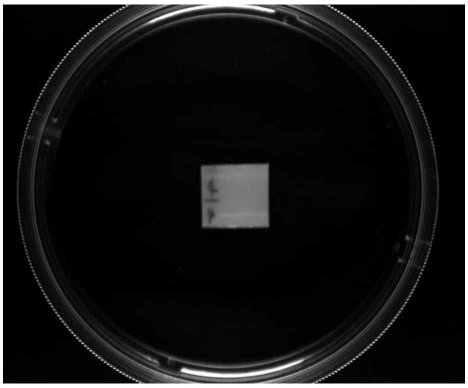

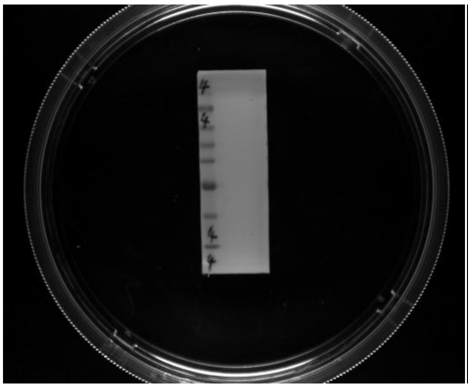

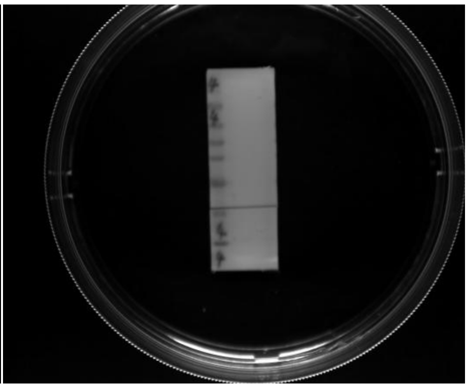

Peroxiredoxin $SO_{2/3}$ (erastin, total)

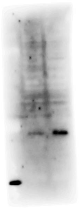

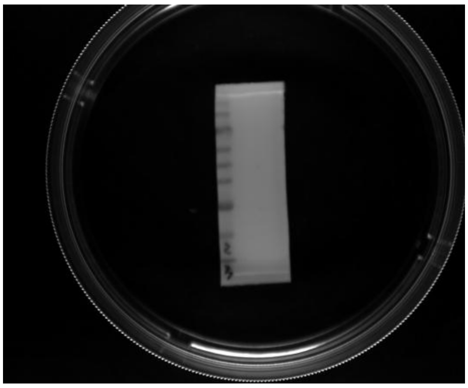

GAPDH (erastin, total)

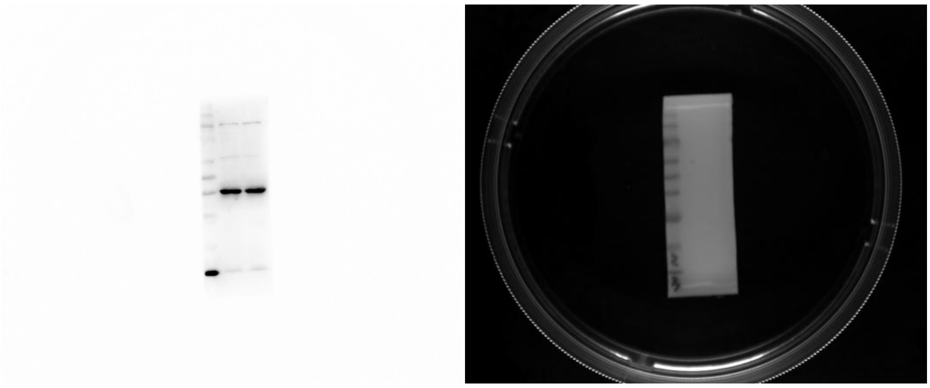

Peroxiredoxin $SO_{2/3}$ (erastin, cytosol)

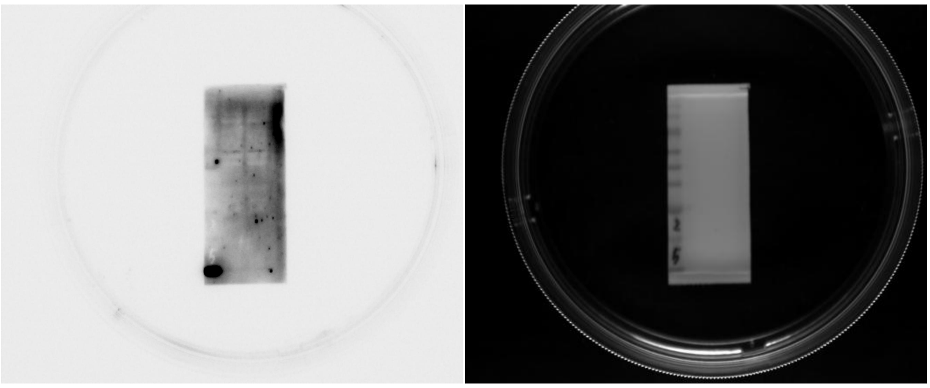

GAPDH (erastin, cytosol)

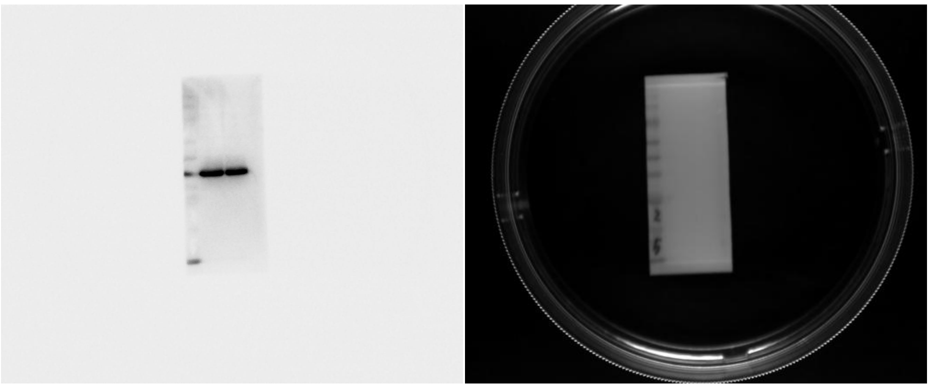

Peroxiredoxin $SO_{2/3}$ (erastin, plasma membrane)

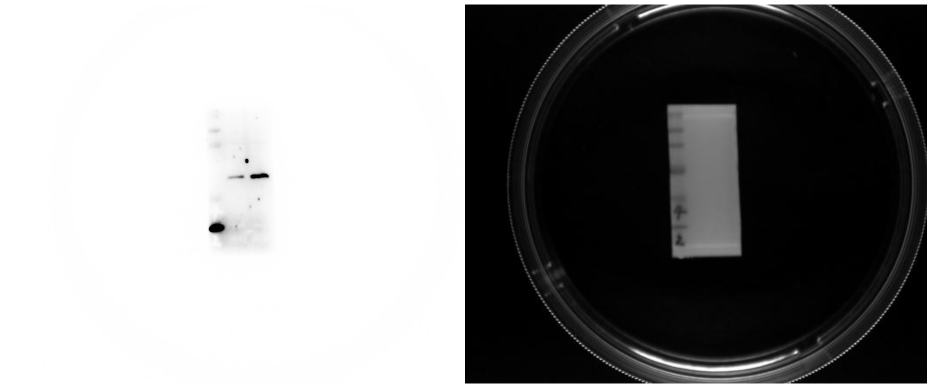

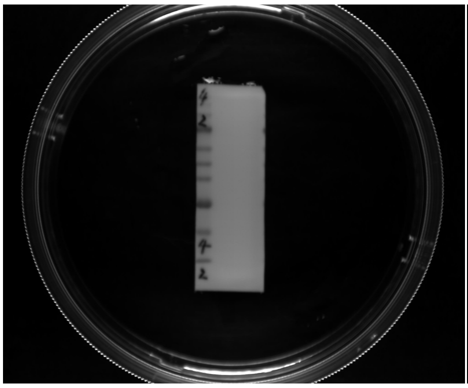

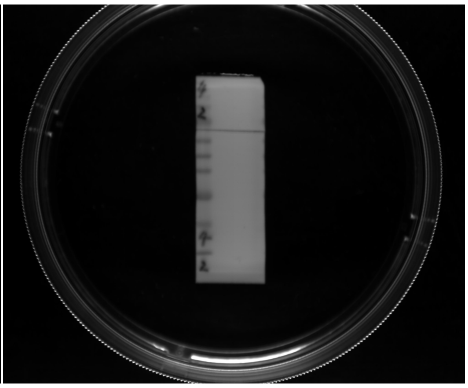

Na$^+$/K$^+$ ATPase a1 (erastin, plasma membrane)

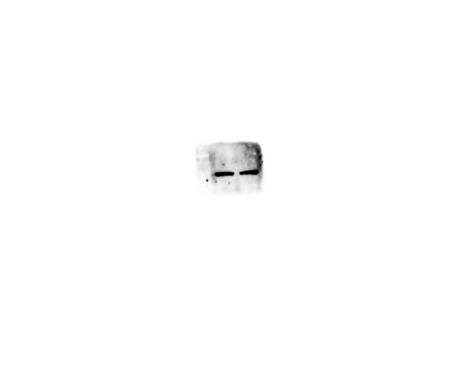

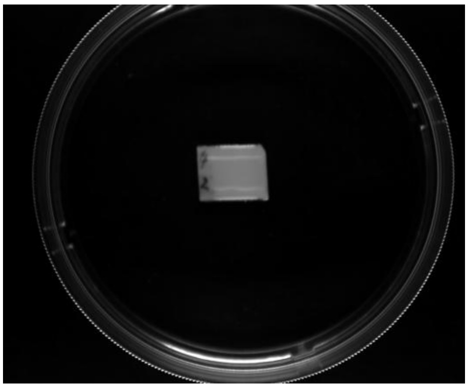

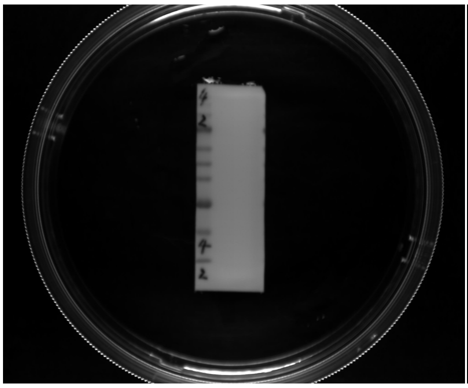

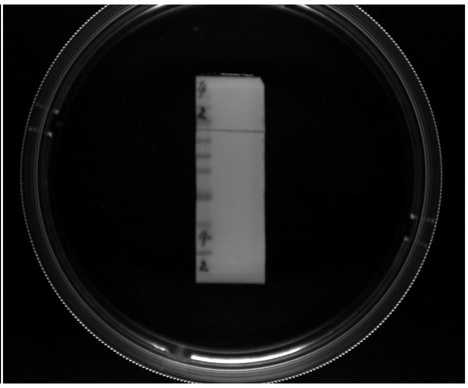

Peroxiredoxin $SO_{2/3}$ (erastin, organelle)

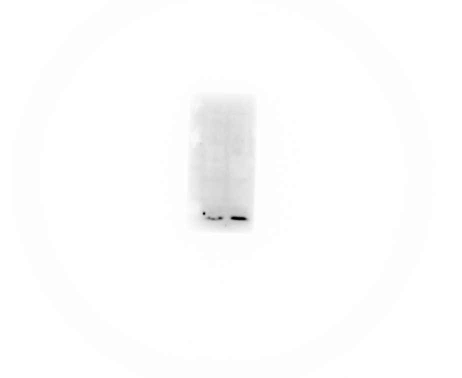

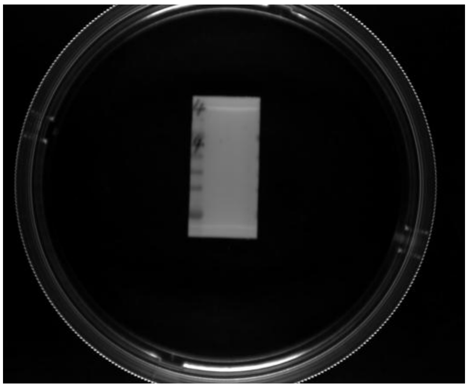

COX IV (erastin, organelle)

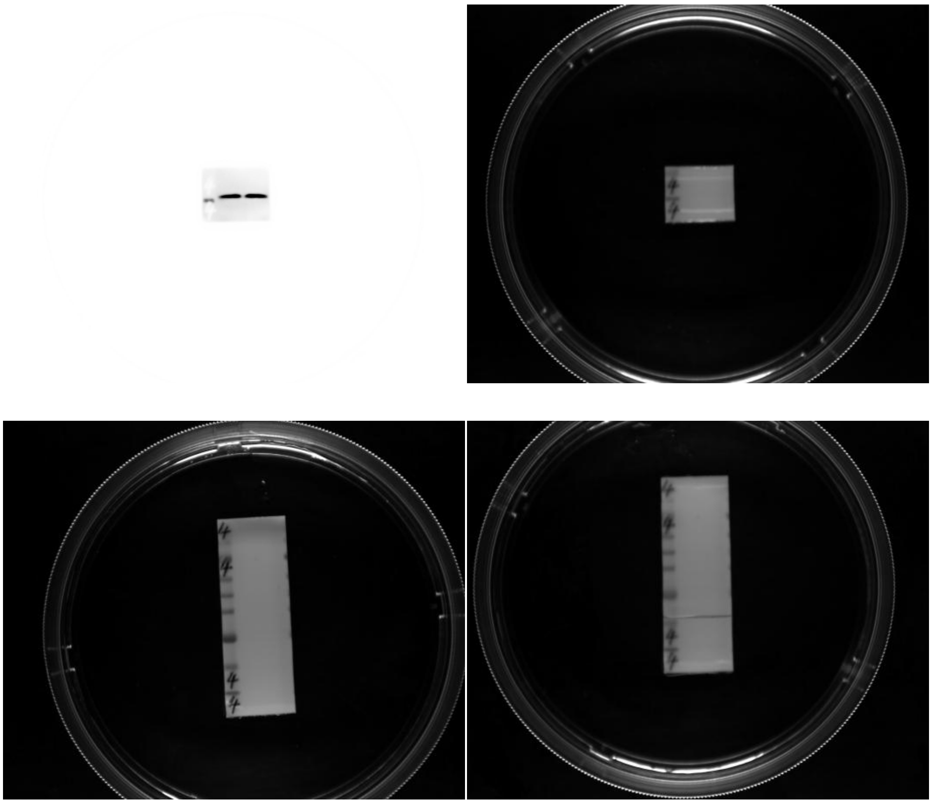

Fig. 7F Third Repetition

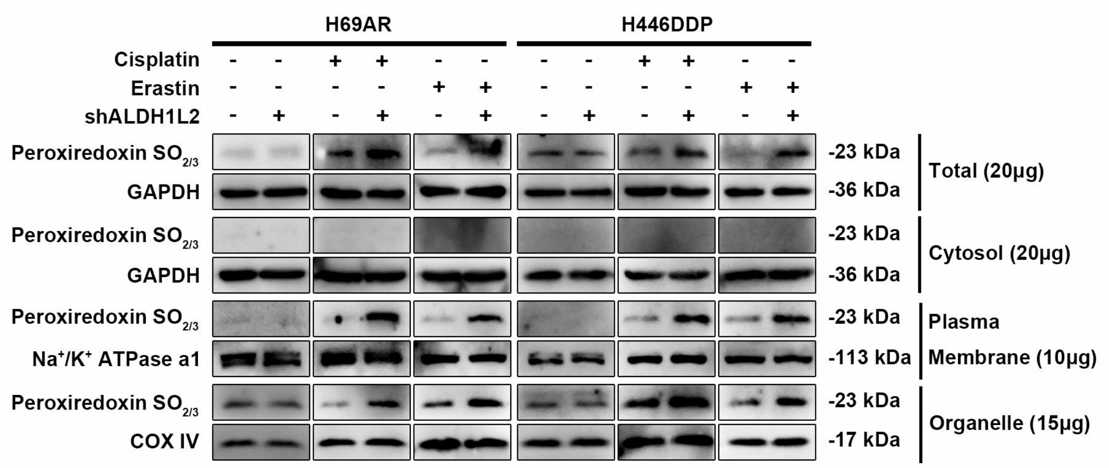

H69AR

Peroxiredoxin $SO_{2/3}$ (untreated, total)

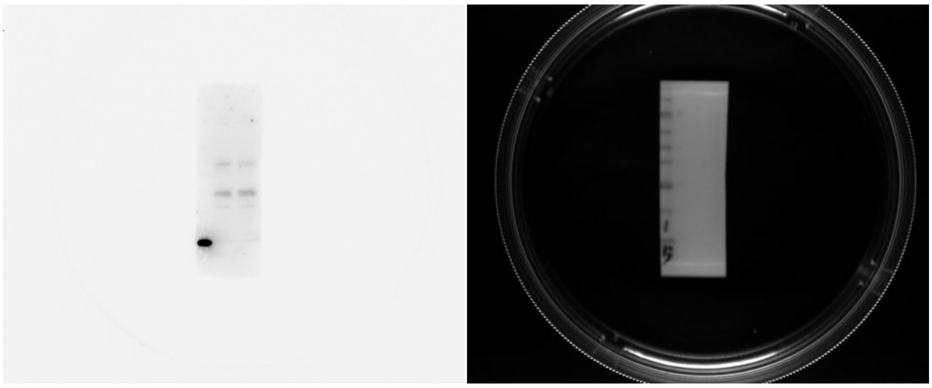

GAPDH (untreated, total)

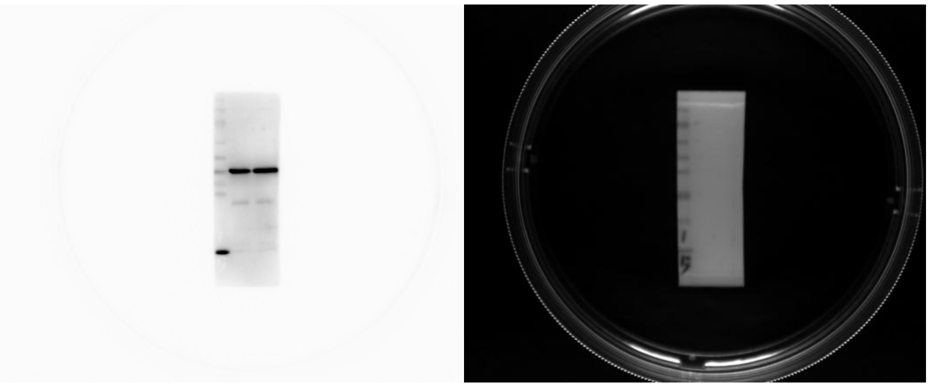

Peroxiredoxin $SO_{2/3}$ (untreated, cytosol)

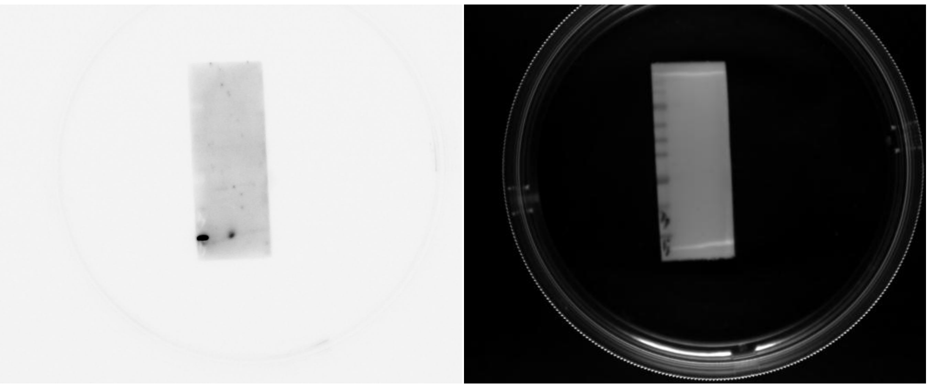

GAPDH (untreated, cytosol)

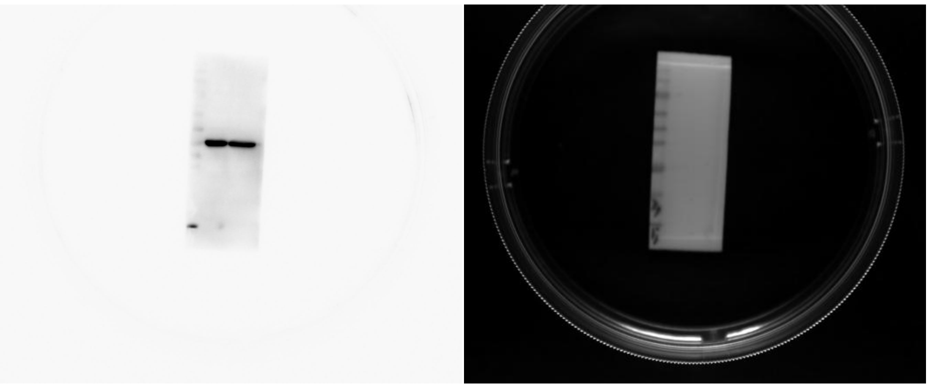

Peroxiredoxin $SO_{2/3}$ (untreated, plasma membrane)

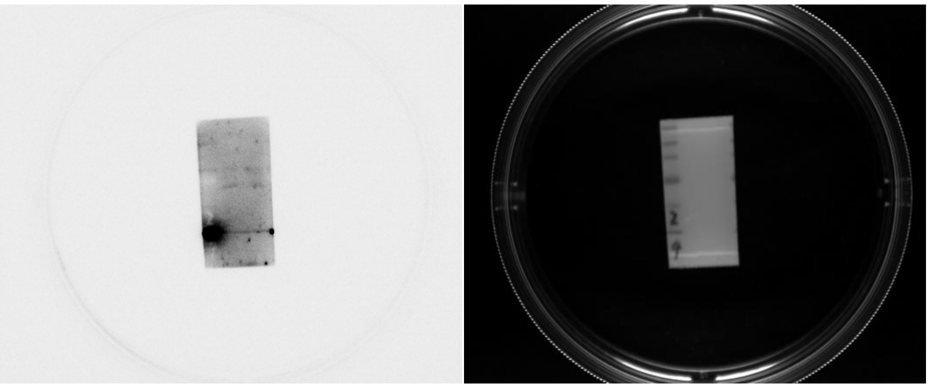

Na^{+}/K^{+} ATPase a1 (untreated, plasma membrane)

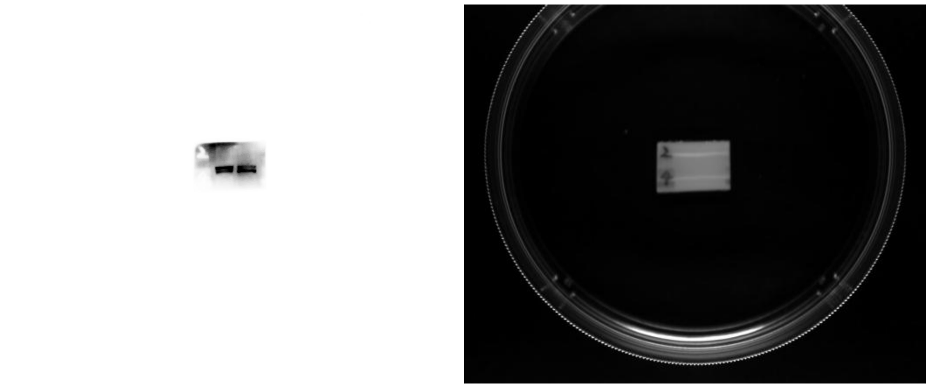

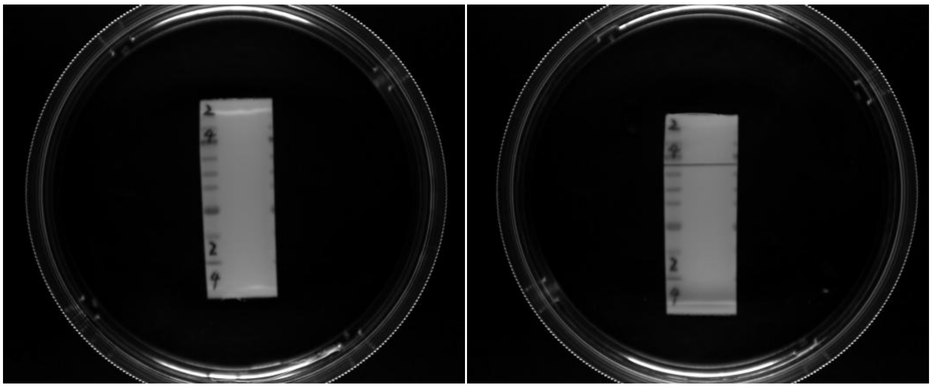

Peroxiredoxin $SO_{2/3}$ (untreated, organelle)

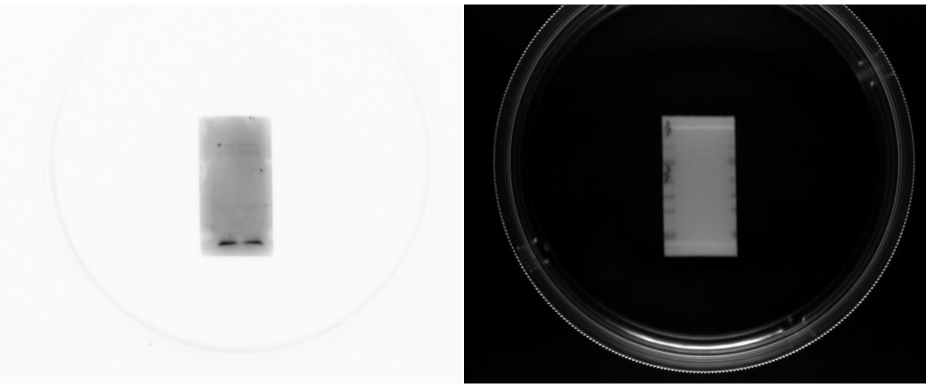

COX IV (untreated, organelle)

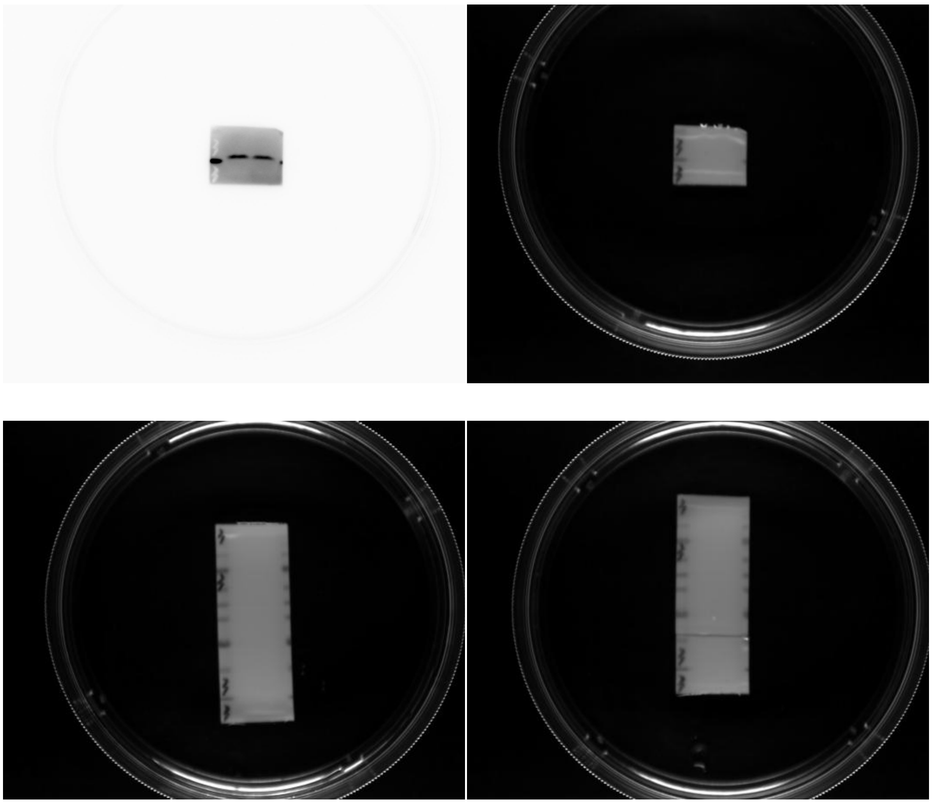

Peroxiredoxin $SO_{2/3}$ (cisplatin, total)

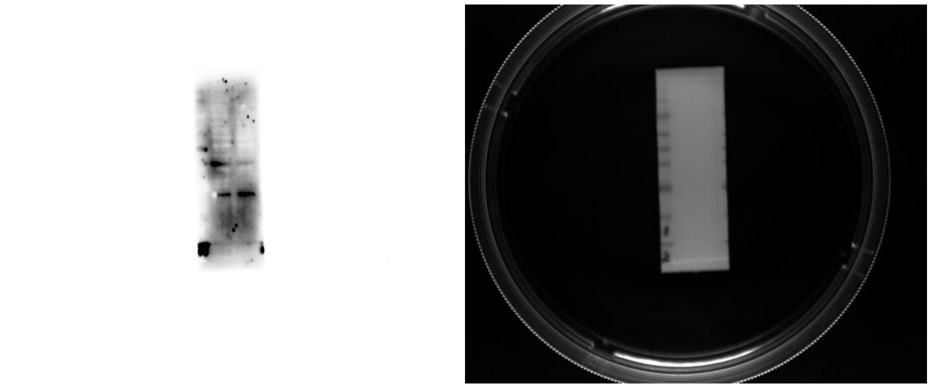

GAPDH (cisplatin, total)

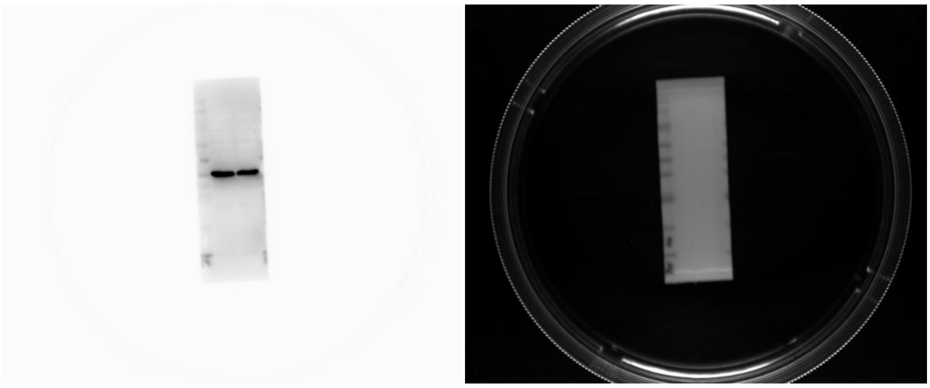

Peroxiredoxin $SO_{2/3}$ (cisplatin, cytosol)

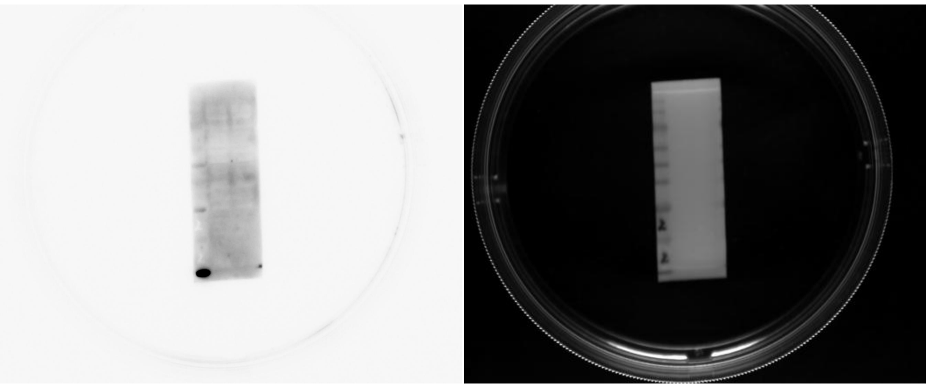

GAPDH (cisplatin, cytosol)

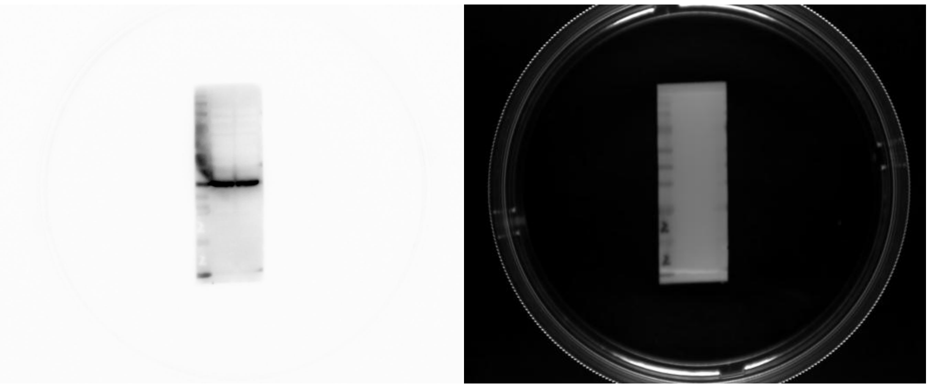

Peroxiredoxin $SO_{2/3}$ (cisplatin, plasma membrane)

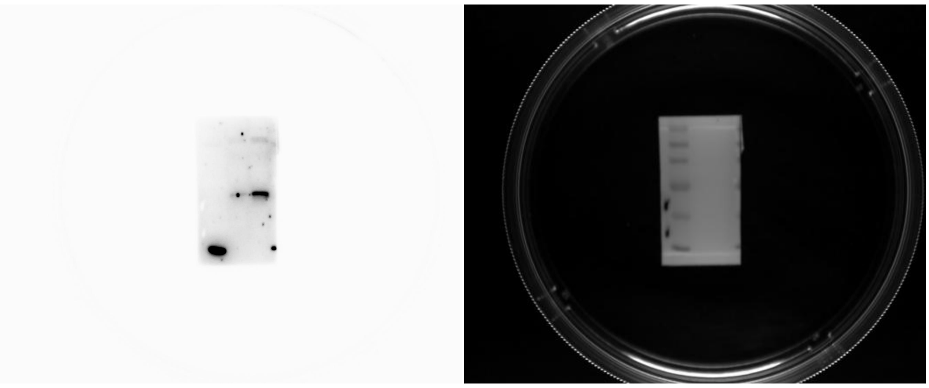

Na^+/K^+ ATPase a1 (cisplatin, plasma membrane)

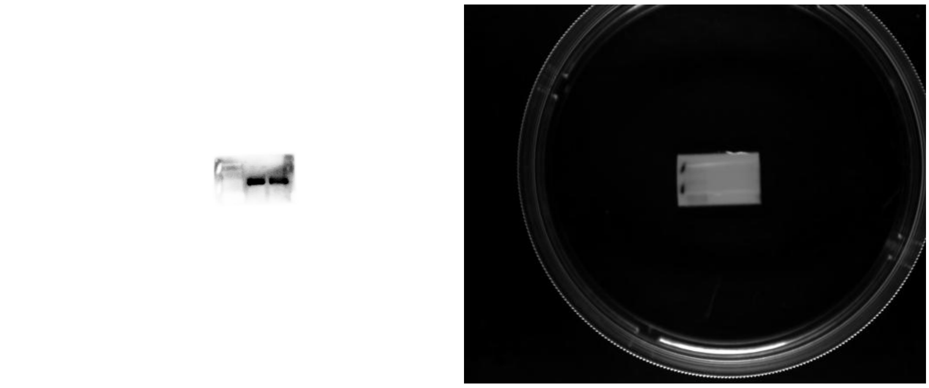

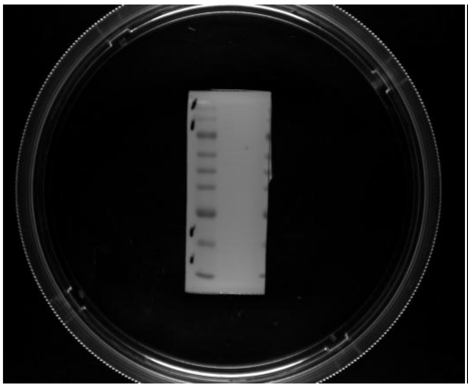
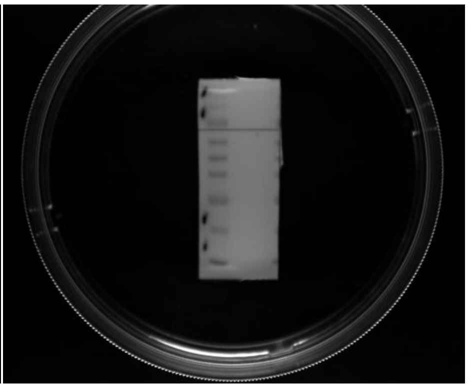

Peroxiredoxin $SO_{2/3}$ (cisplatin, organelle)

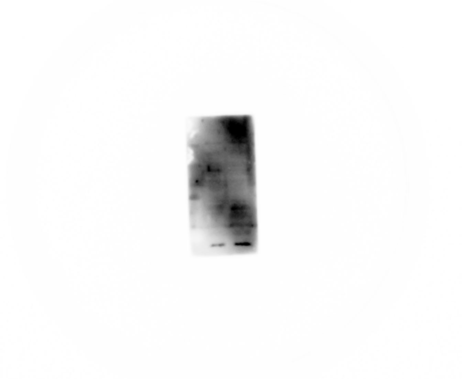
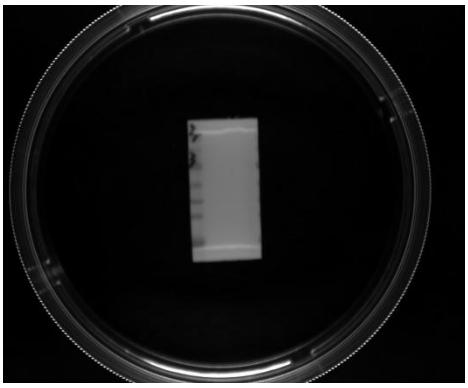

COX IV (cisplatin, organelle)

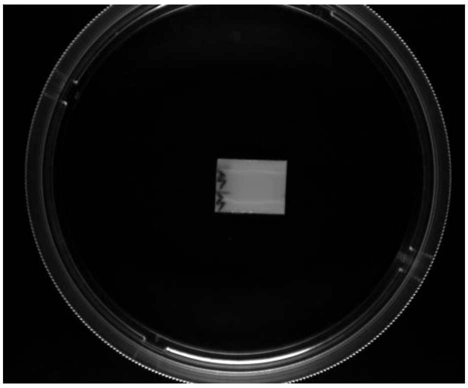

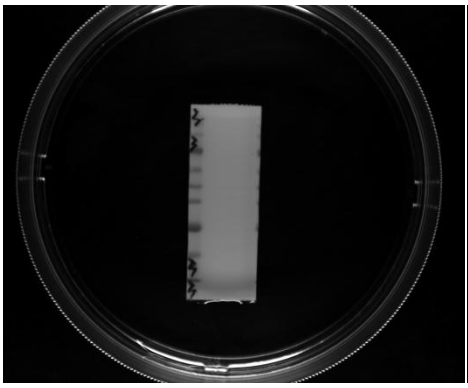
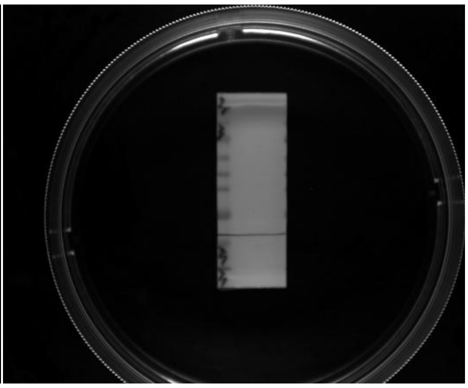

Peroxiredoxin $SO_{2/3}$ (erastin, total)

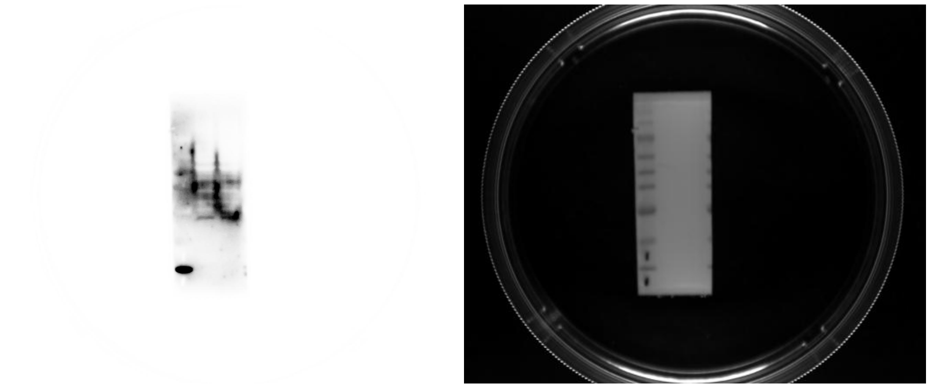

GAPDH (erastin, total)

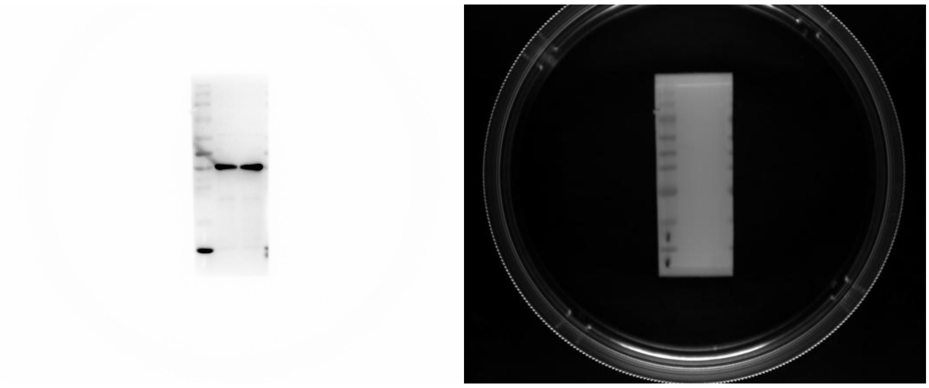

Peroxiredoxin $SO_{2/3}$ (erastin, cytosol)

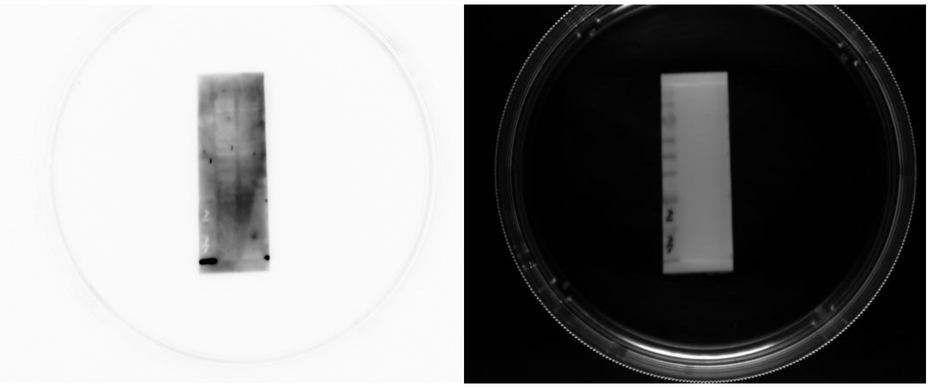

GAPDH (erastin, cytosol)

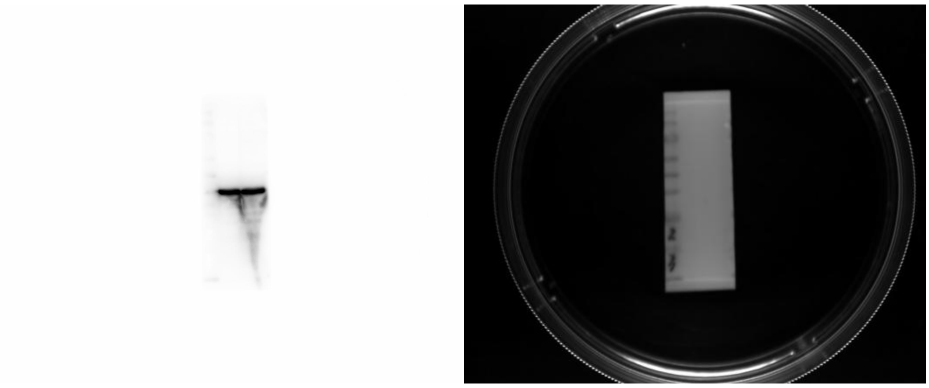

Peroxiredoxin $SO_{2/3}$ (erastin, plasma membrane)

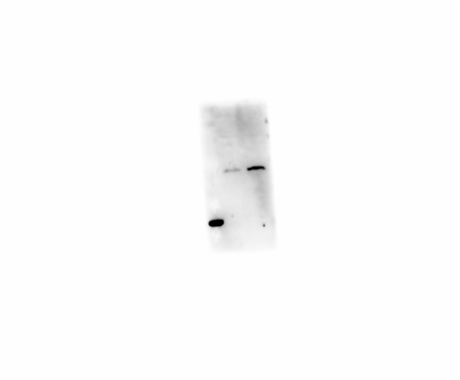

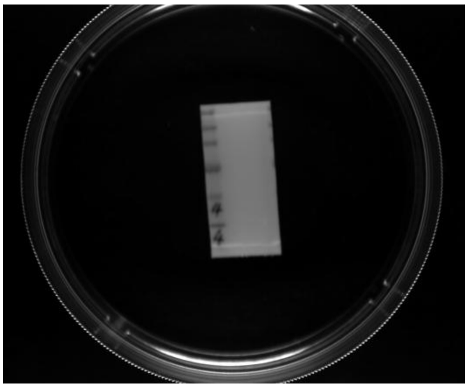

Na^+/K^+ ATPase a1 (erastin, plasma membrane)

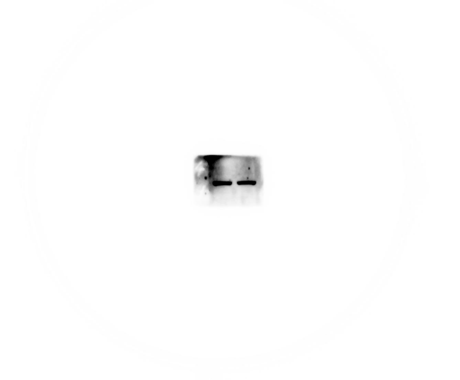

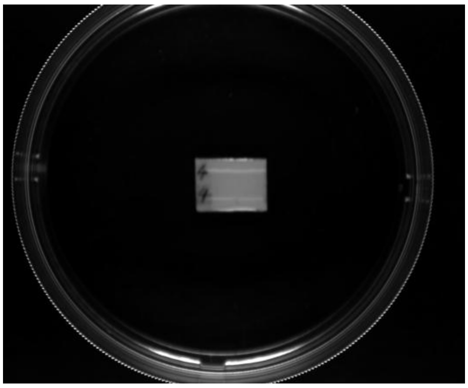

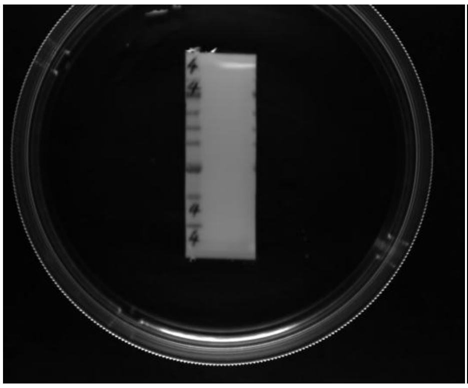

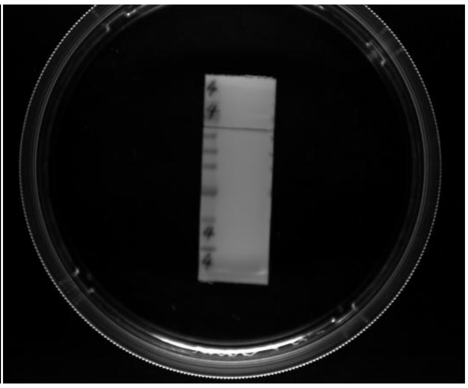

Peroxiredoxin $SO_{2/3}$ (erastin, organelle)

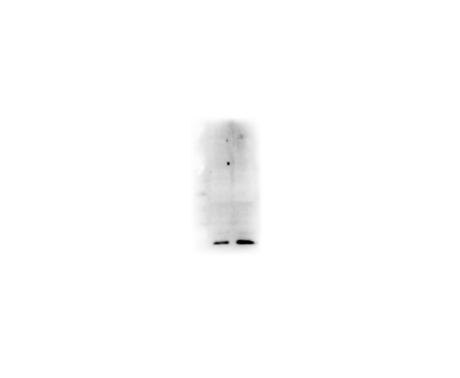

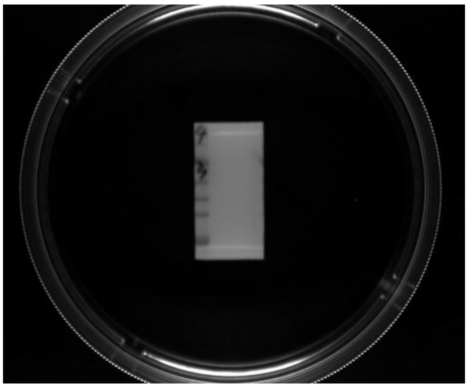

COX IV (erastin, organelle)

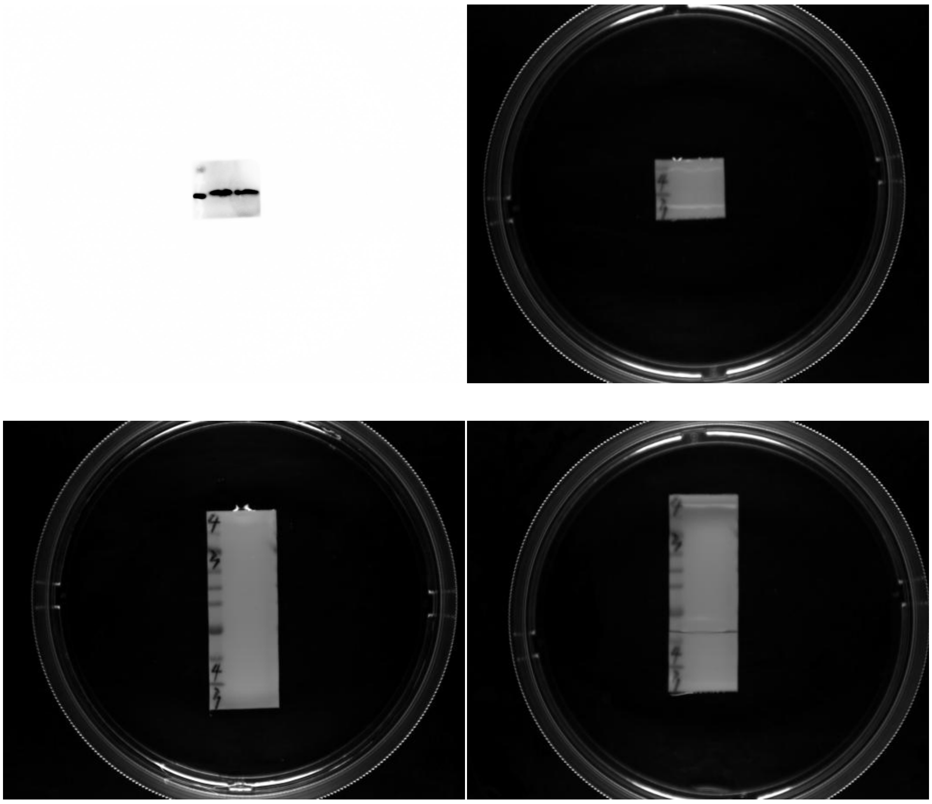

H446DDP

Peroxiredoxin $SO_{2/3}$ (untreated, total)

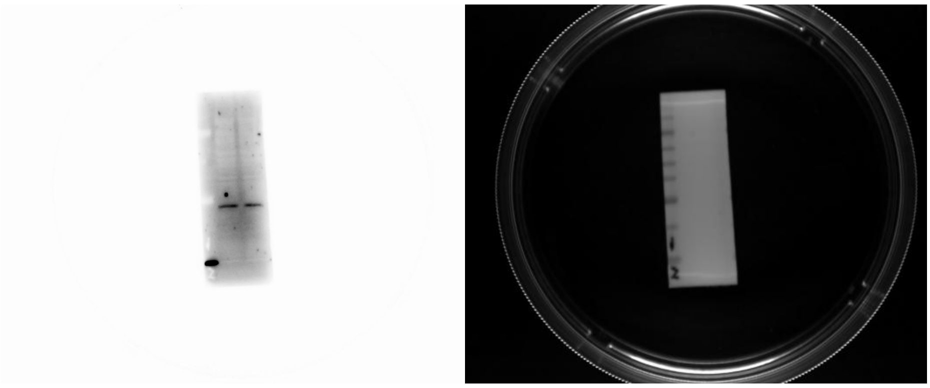

GAPDH (untreated, total)

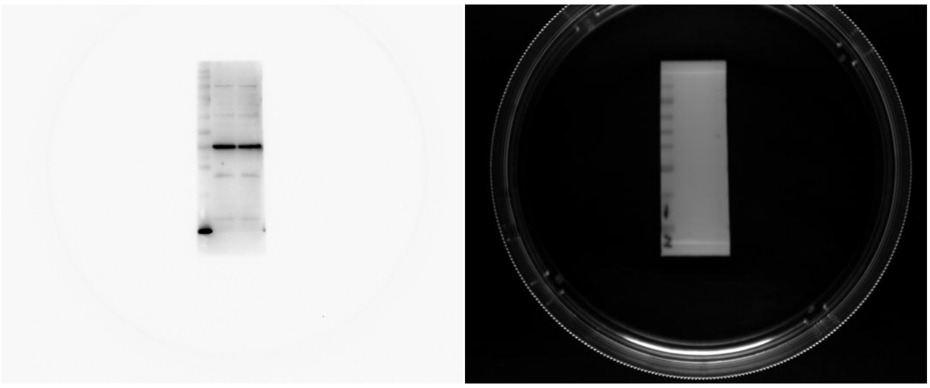

Peroxiredoxin $SO_{2/3}$ (untreated, cytosol)

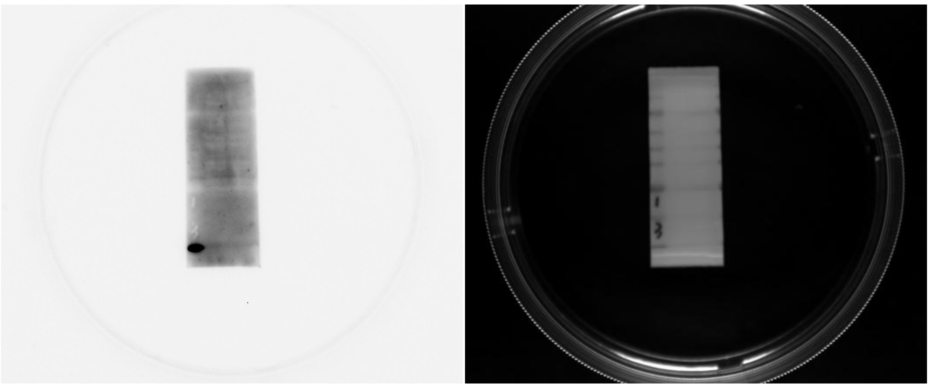

GAPDH (untreated, cytosol)

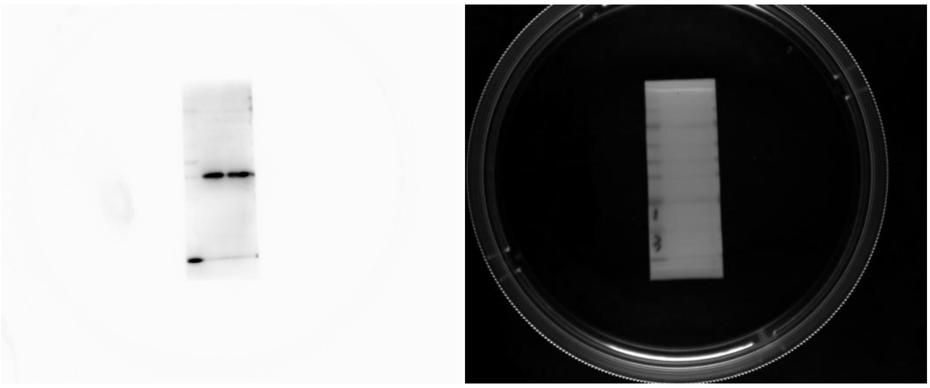

Peroxiredoxin $SO_{2/3}$ (untreated, plasma membrane)

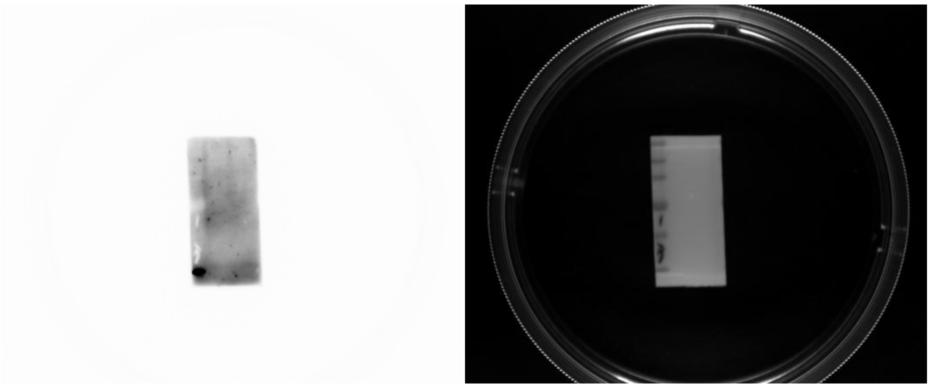

Na^{+}/K^{+} ATPase a1 (untreated, plasma membrane)

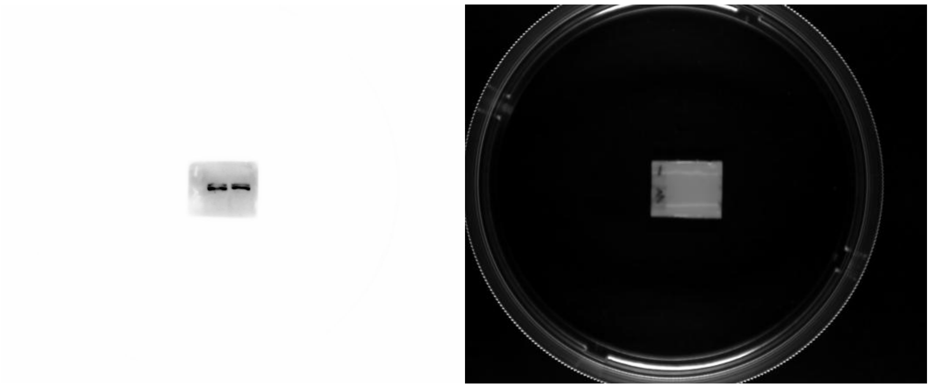

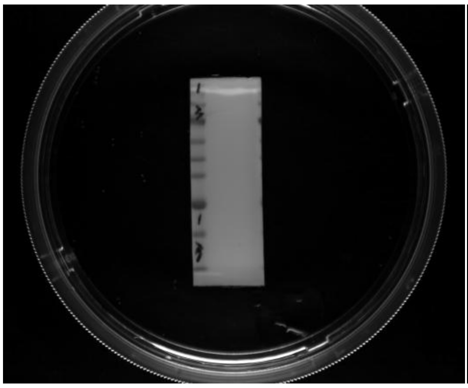

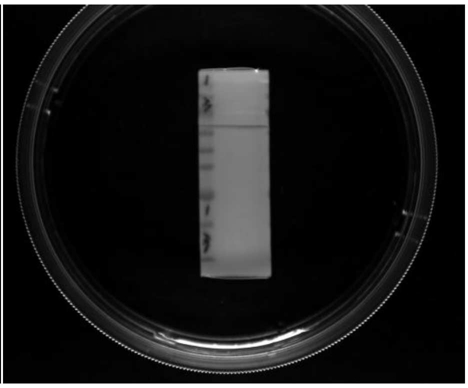

Peroxiredoxin $SO_{2/3}$ (untreated, organelle)

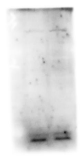

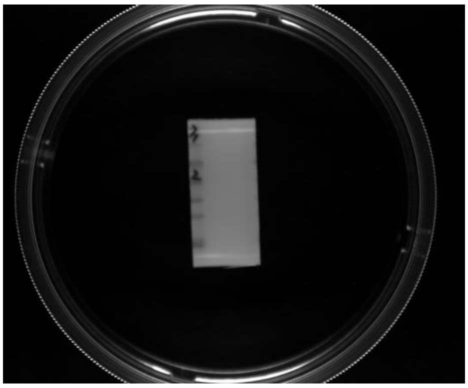

COX IV (untreated, organelle)

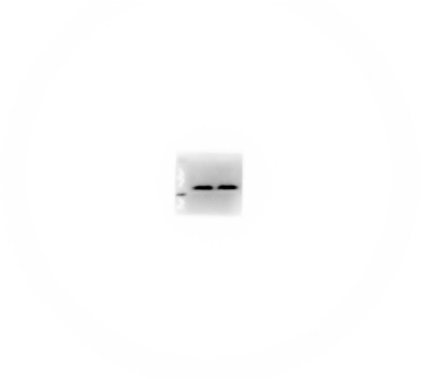

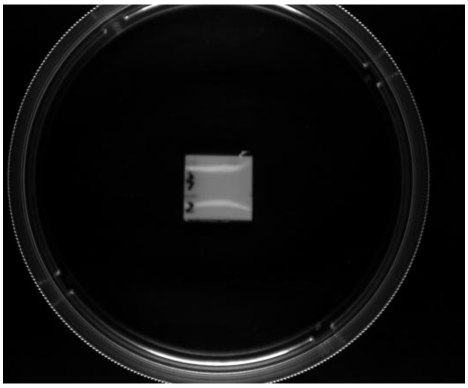

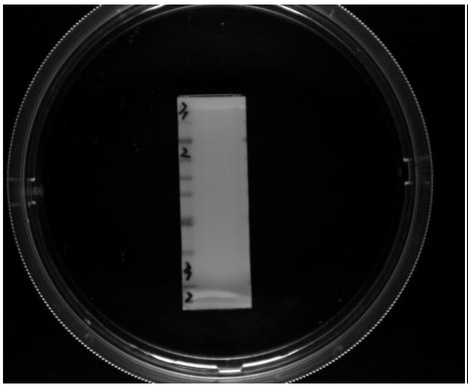

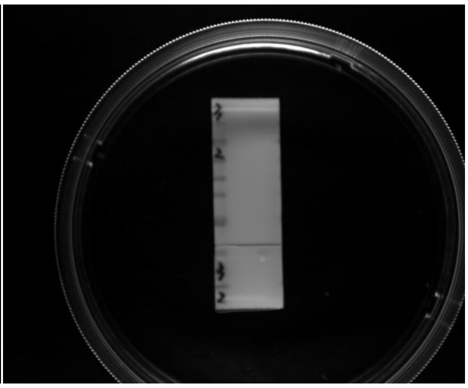

Peroxiredoxin $SO_{2/3}$ (cisplatin, total)

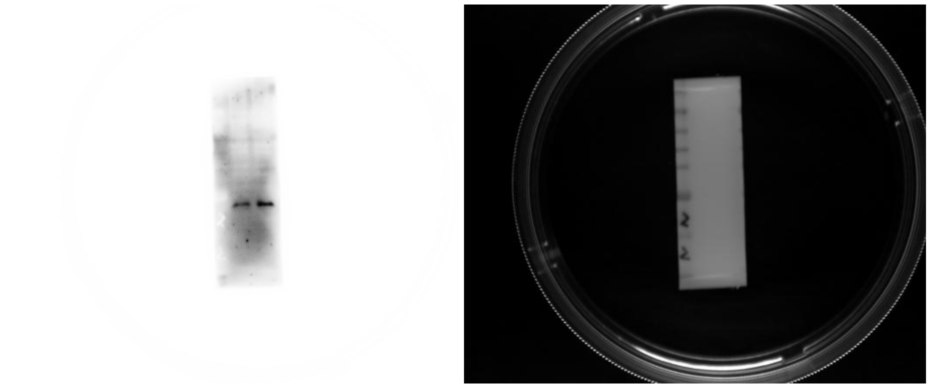

GAPDH (cisplatin, total)

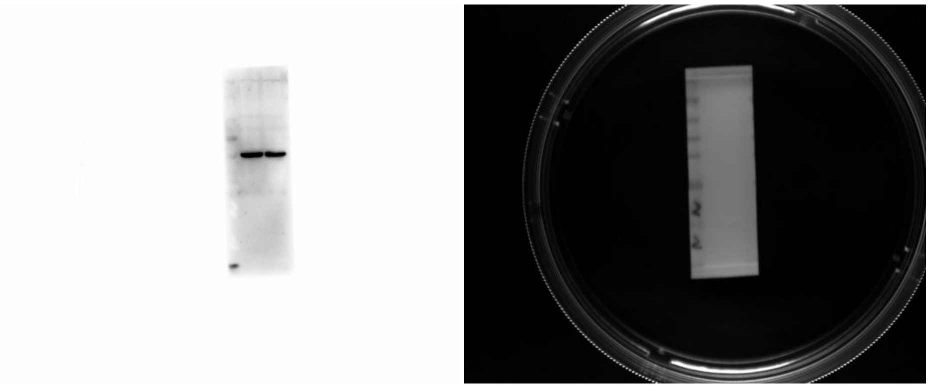

Peroxiredoxin $SO_{2/3}$ (cisplatin, cytosol)

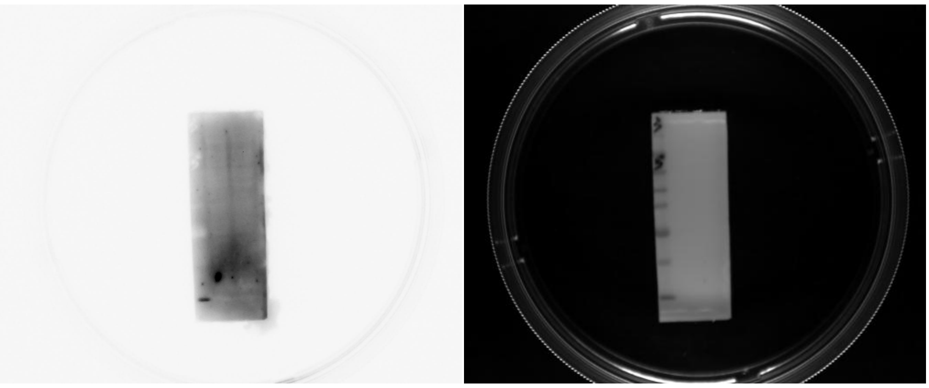

GAPDH (cisplatin, cytosol)

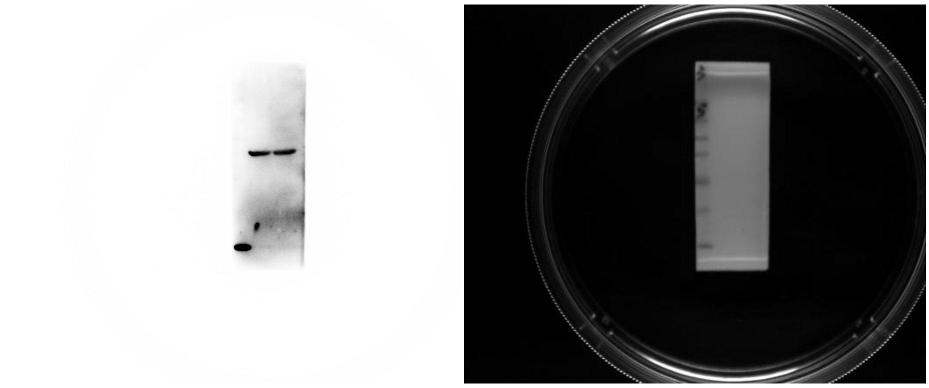

Peroxiredoxin $SO_{2/3}$ (cisplatin, plasma membrane)

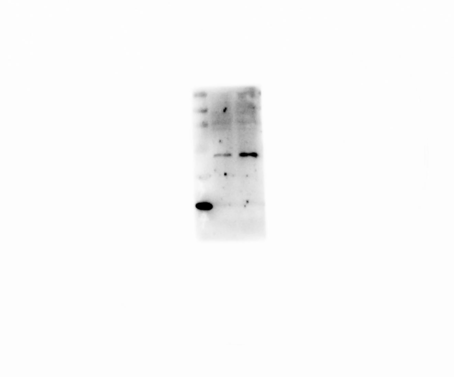
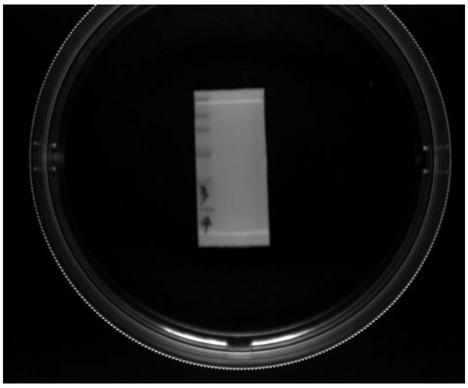

Na^{+}/K^{+} ATPase a1 (cisplatin, plasma membrane)

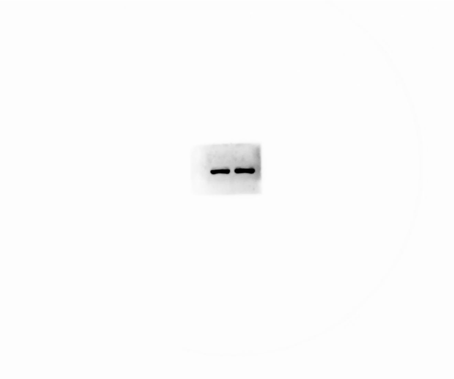
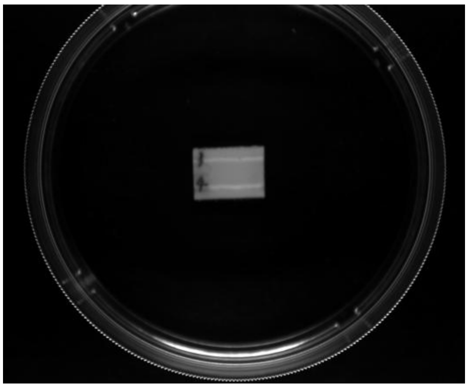

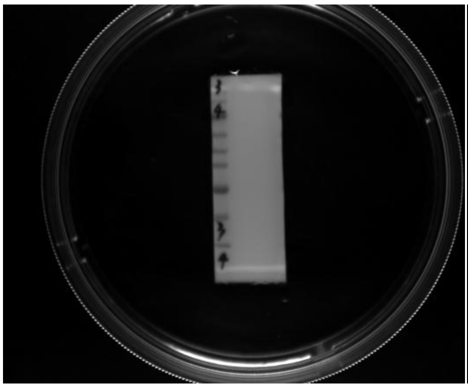
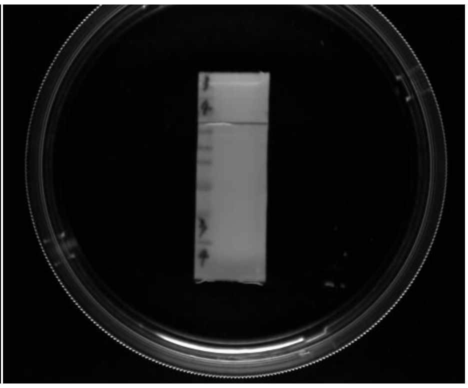

Peroxiredoxin $SO_{2/3}$ (cisplatin, organelle)

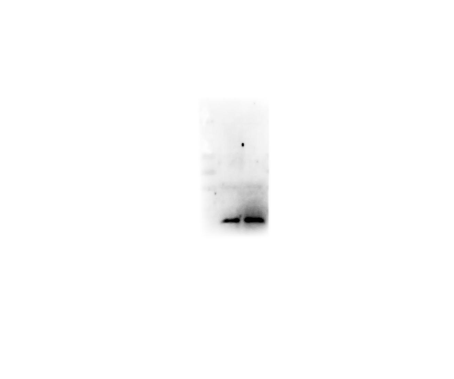
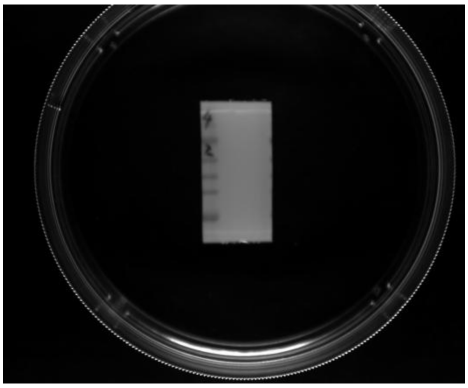

COX IV (cisplatin, organelle)

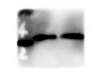

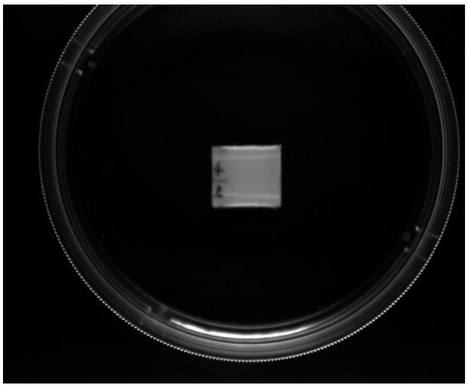

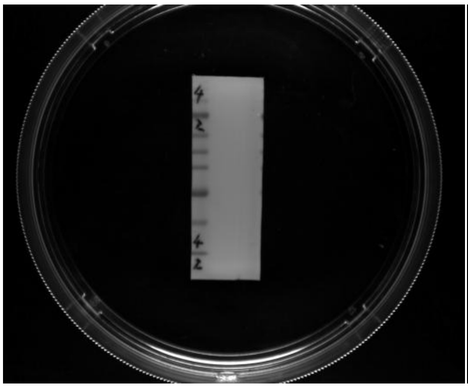

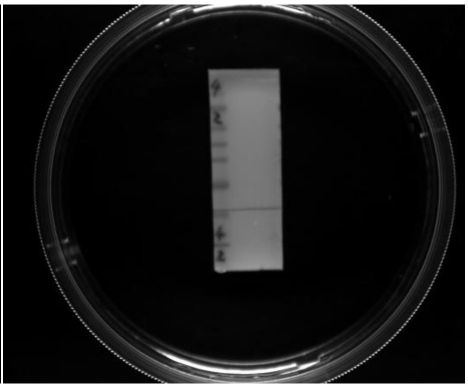

Peroxiredoxin $SO_{2/3}$ (erastin, total)

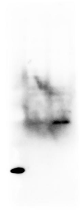

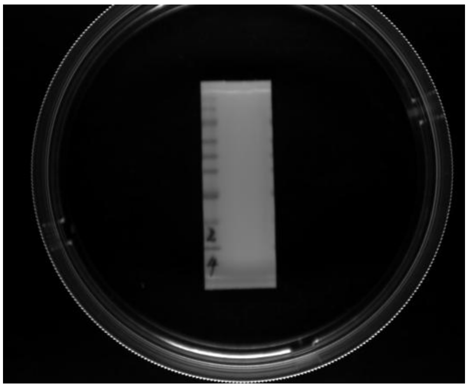

GAPDH (erastin, total)

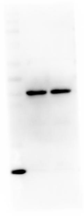

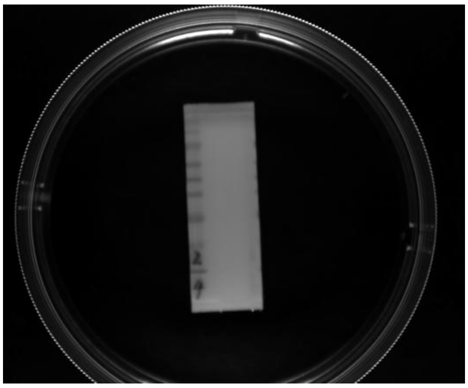

Peroxiredoxin $SO_{2/3}$ (erastin, cytosol)

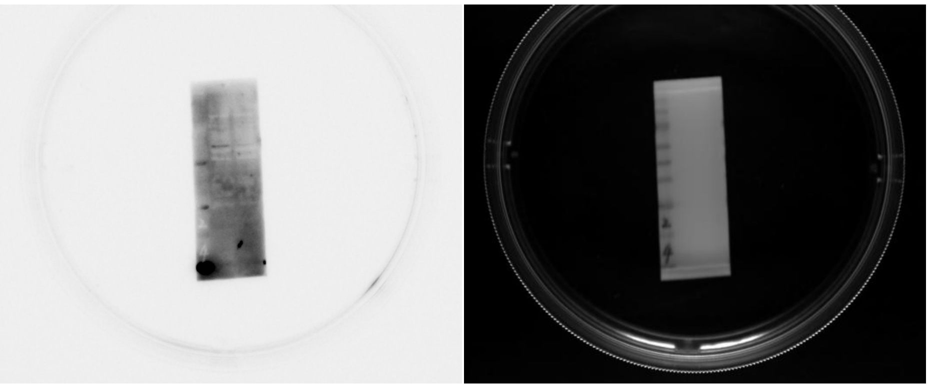

GAPDH (erastin, cytosol)

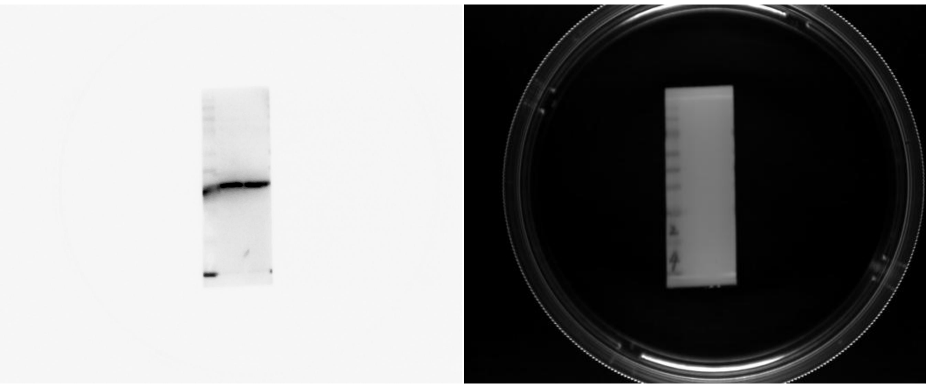

Peroxiredoxin $SO_{2/3}$ (erastin, plasma membrane)

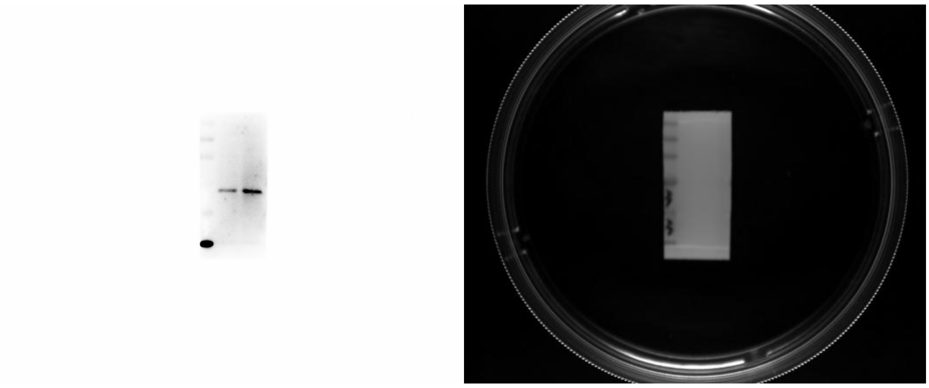

Na^{+}/K^{+} ATPase a1 (erastin, plasma membrane)

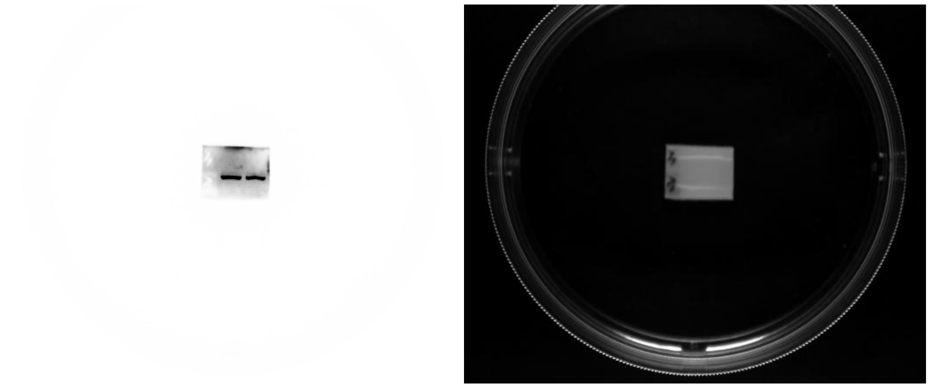

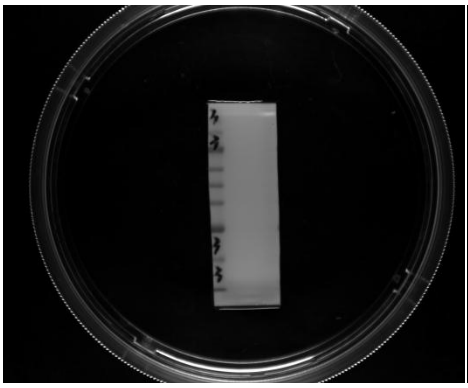

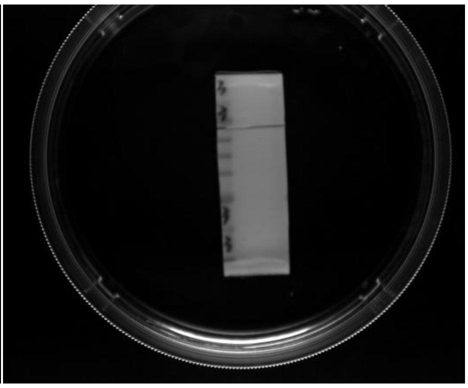

Peroxiredoxin $SO_{2/3}$ (erastin, organelle)

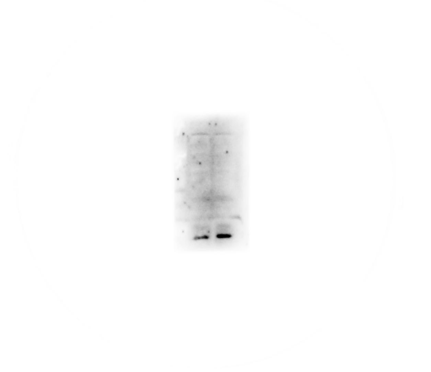

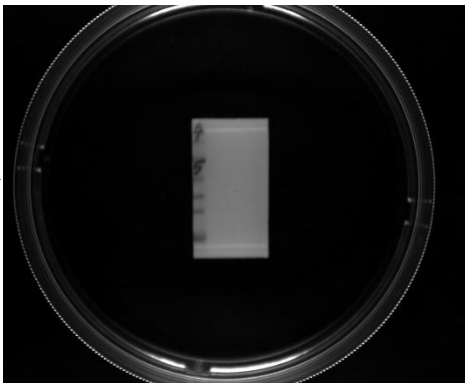

COX IV (erastin, organelle)

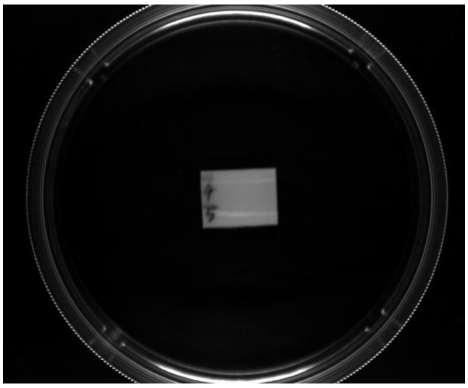

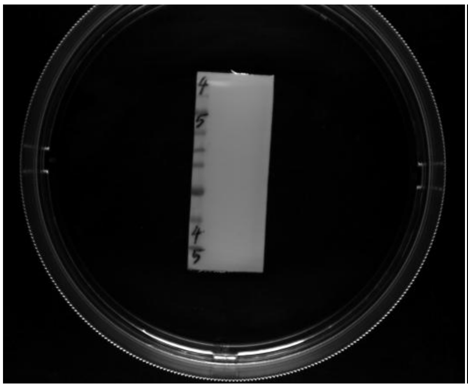

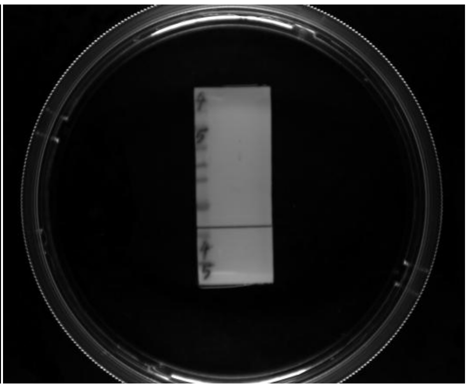

Fig. 7I First Repetition

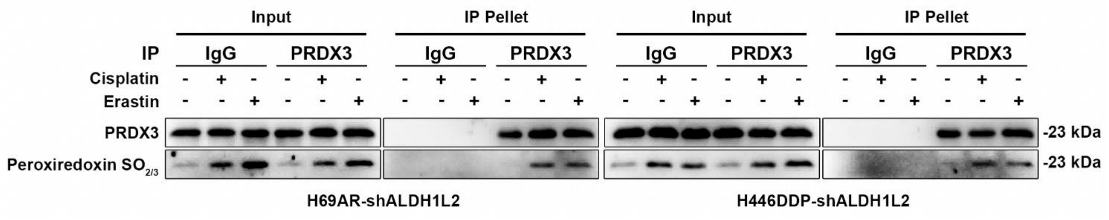

H69AR-shALDH1L2

PRDX3 (Input)

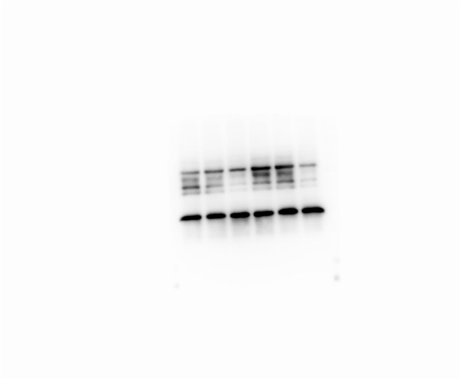
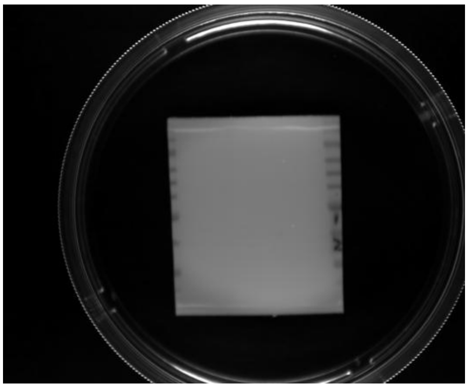

PRDX3 (IP Pellet)

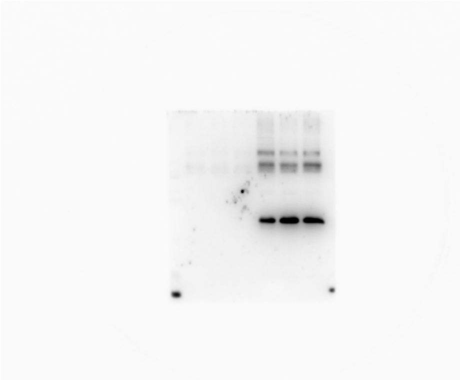
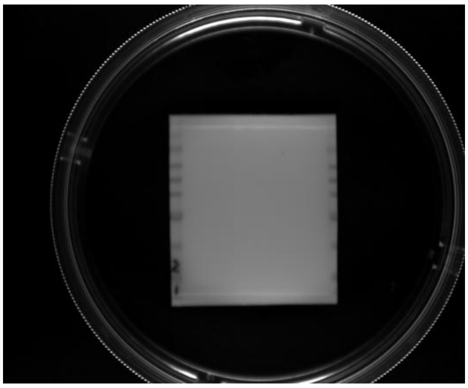

Peroxiredoxin $SO_{2/3}$ (Input)

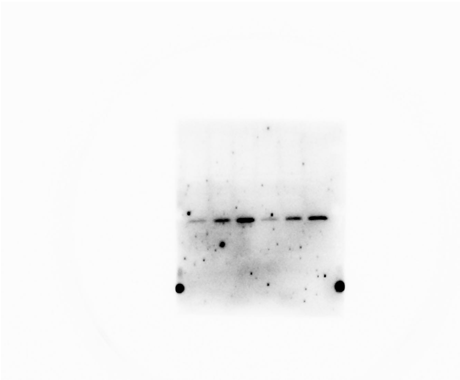
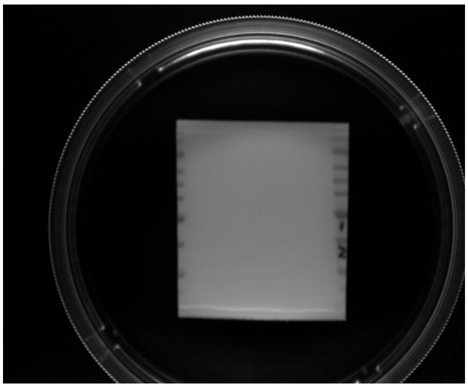

Peroxiredoxin $SO_{2/3}$ (IP Pellet)

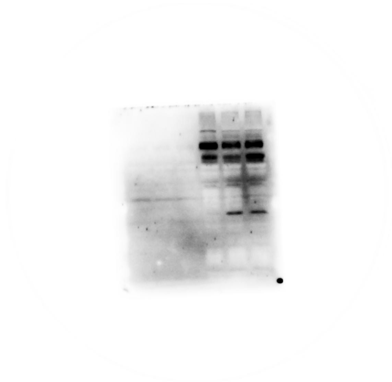

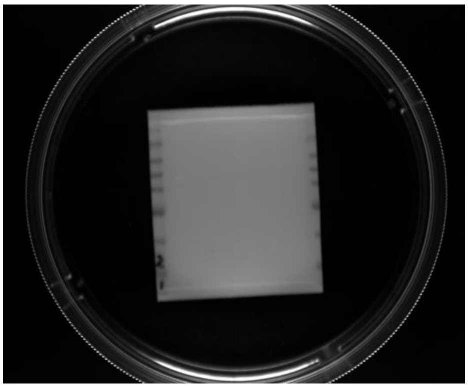

H446DDP-shALDH1L2

PRDX3 (Input)

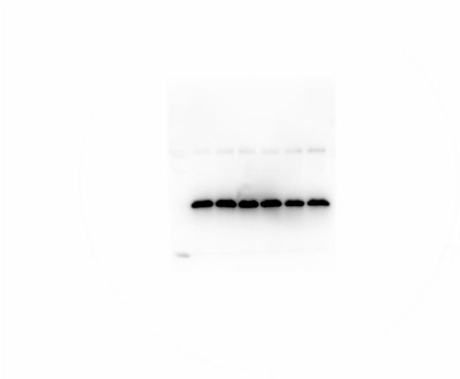

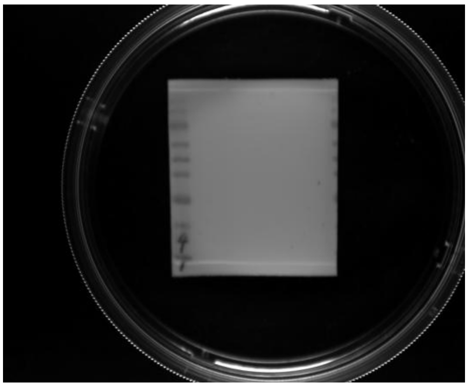

PRDX3 (IP Pellet)

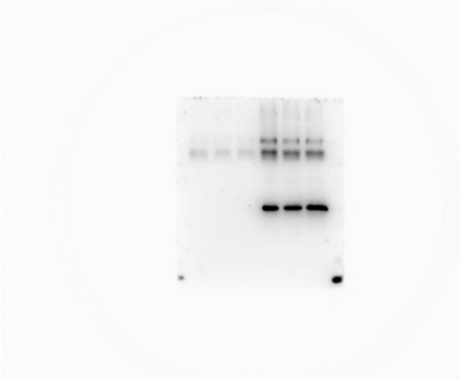

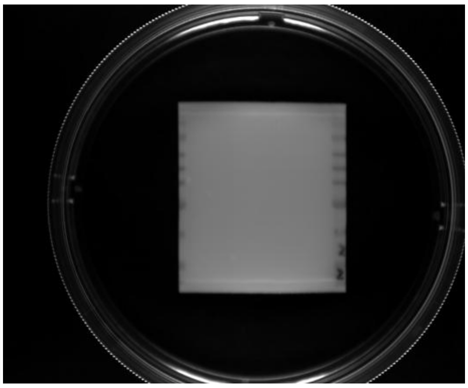

Peroxiredoxin $SO_{2/3}$ (Input)

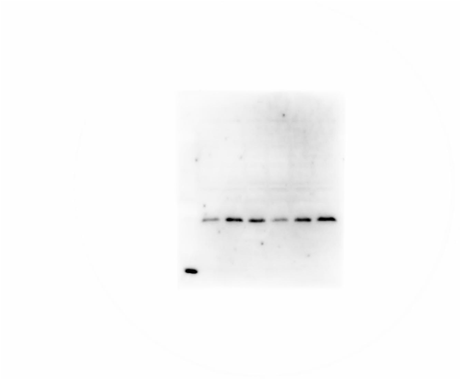

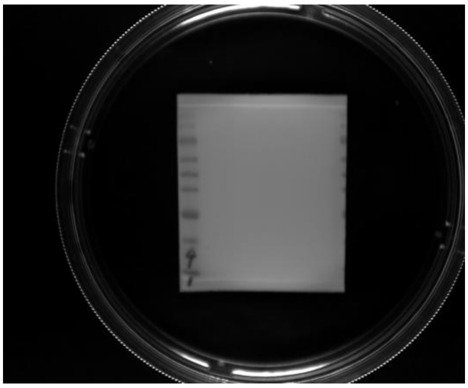

Peroxiredoxin $SO_{2/3}$ (IP Pellet)

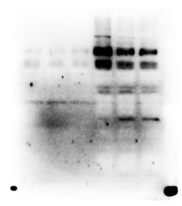

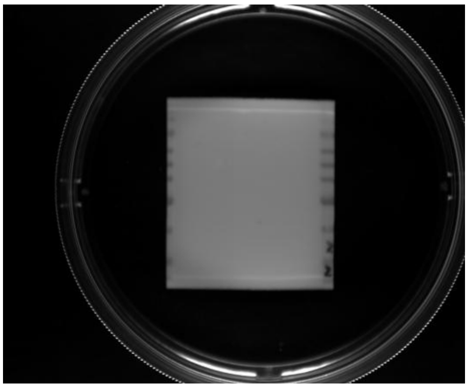

Fig. 7I Second Repetition

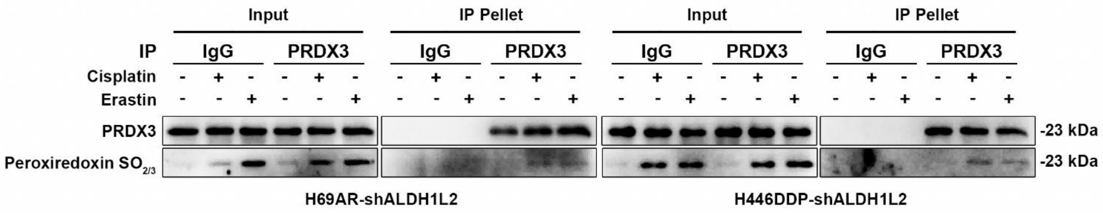

H69AR-shALDH1L2

PRDX3 (Input)

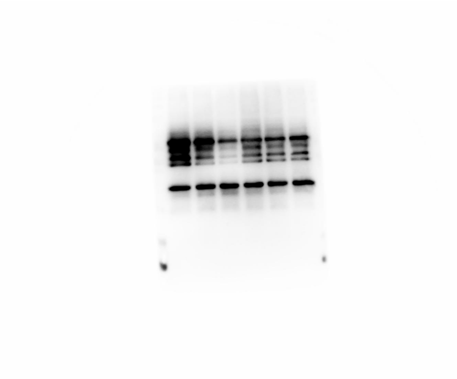

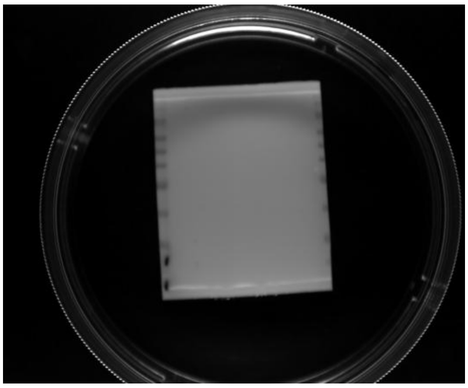

PRDX3 (IP Pellet)

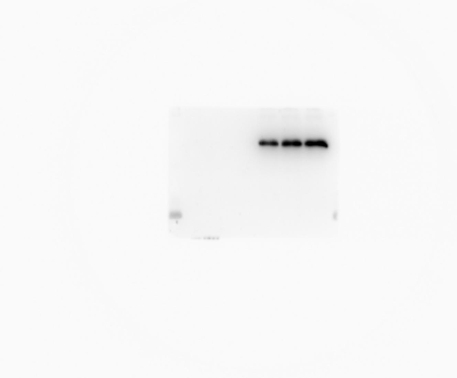

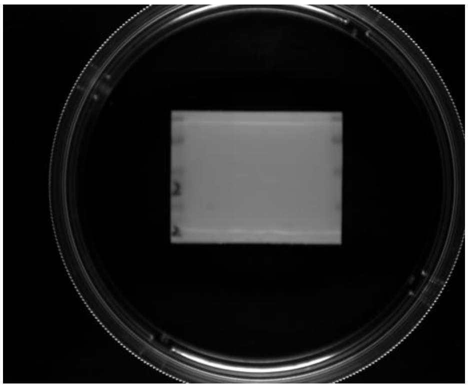

Peroxiredoxin $SO_{2/3}$ (Input)

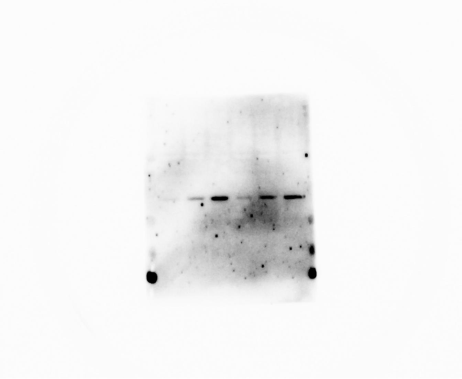

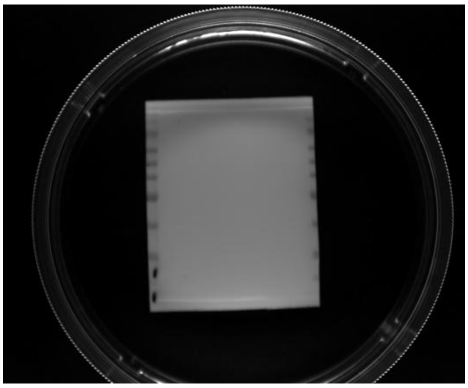

Peroxiredoxin $SO_{2/3}$ (IP Pellet)

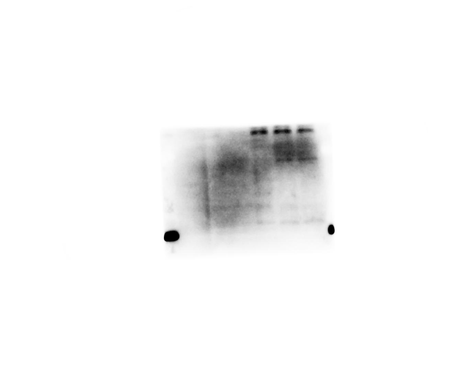

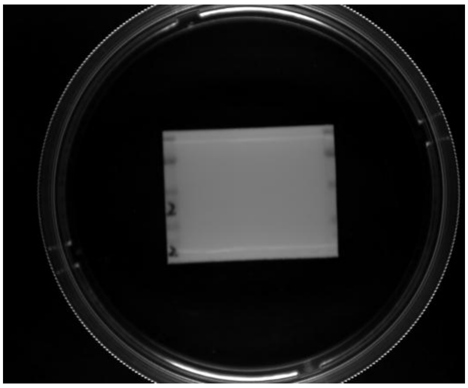

H446DDP-shALDH1L2

PRDX3 (Input)

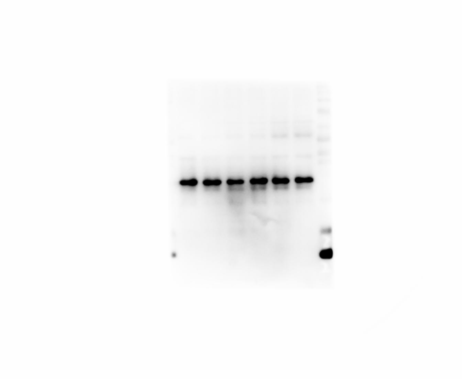

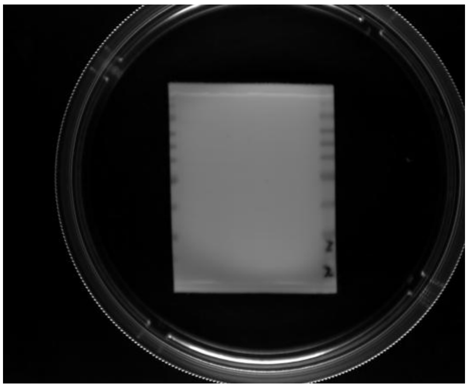

PRDX3 (IP Pellet)

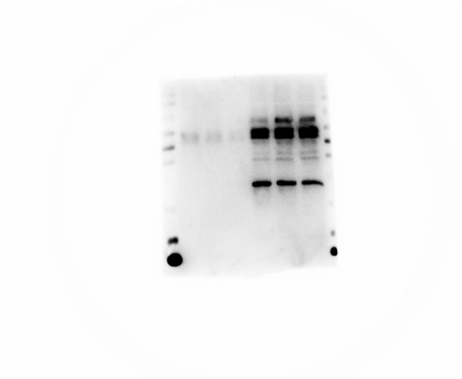

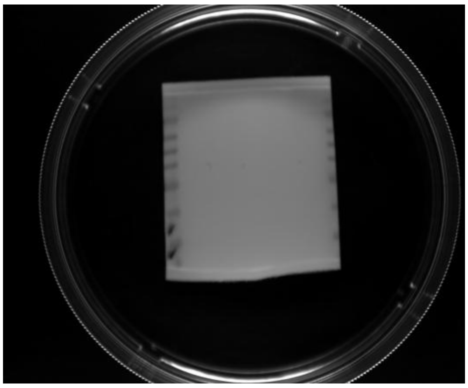

Peroxiredoxin $SO_{2/3}$ (Input)

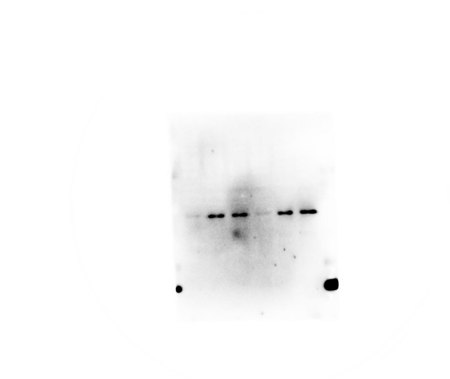

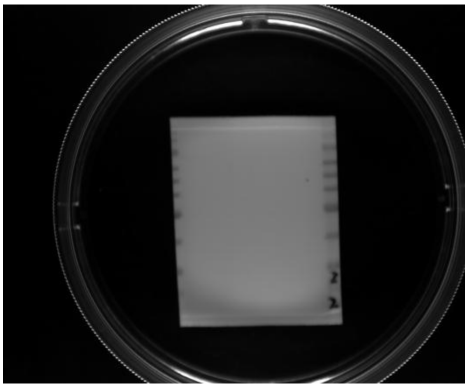

Peroxiredoxin $SO_{2/3}$ (IP Pellet)

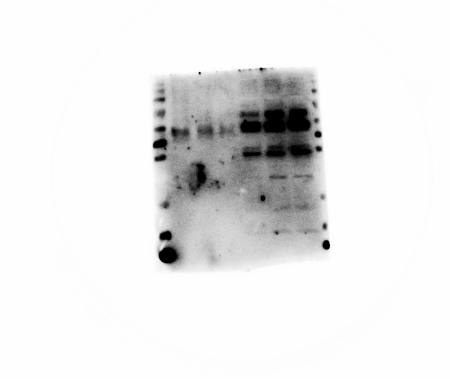

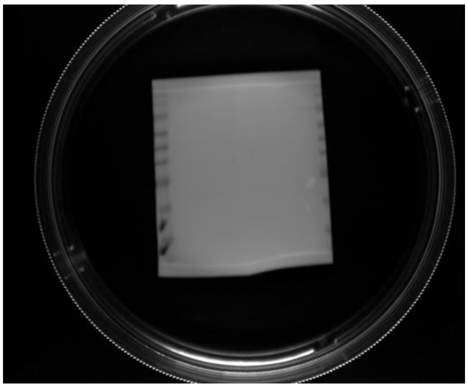

Fig. 7I Third Repetition

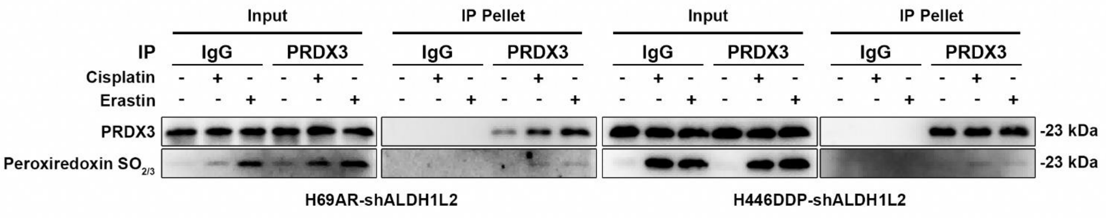

H69AR-shALDH1L2

PRDX3 (Input)

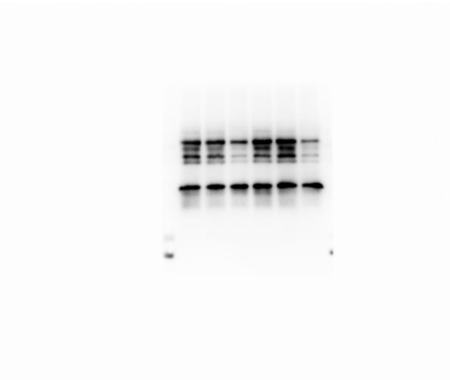

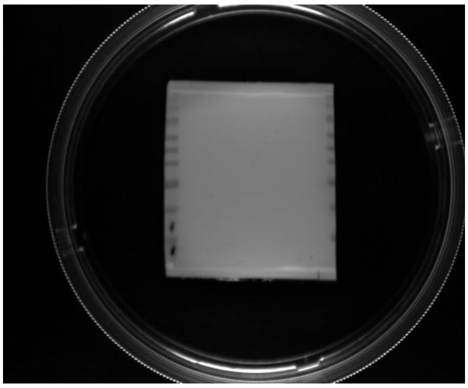

PRDX3 (IP Pellet)

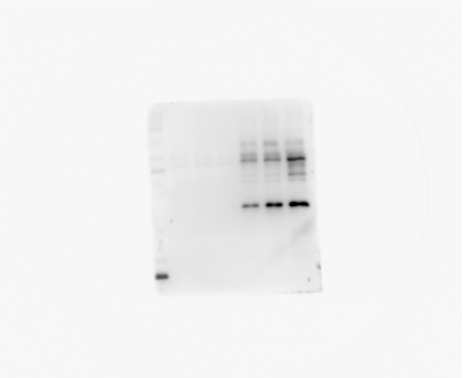
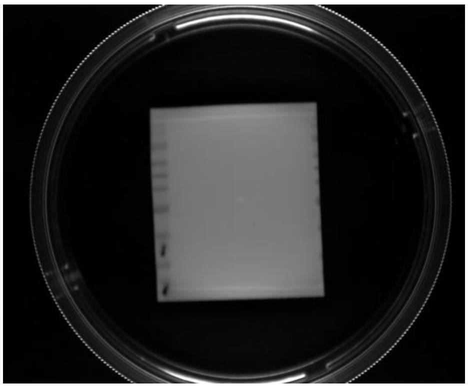

Peroxiredoxin $SO_{2/3}$ (Input)

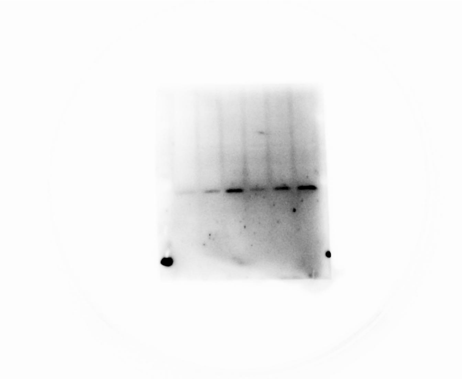
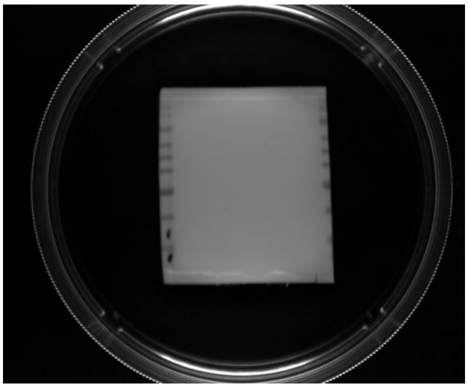

Peroxiredoxin $SO_{2/3}$ (IP Pellet)

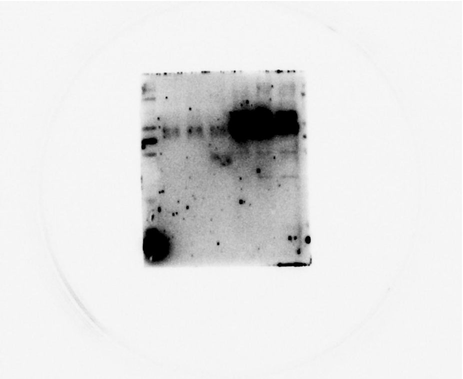
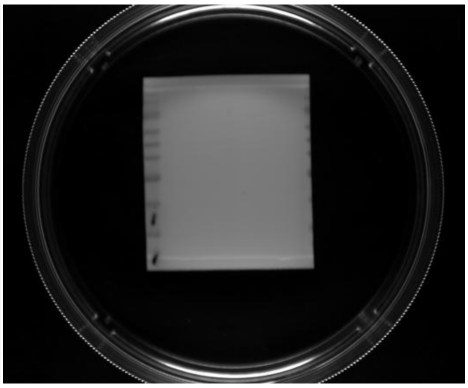

H446DDP-shALDH1L2

PRDX3 (Input)

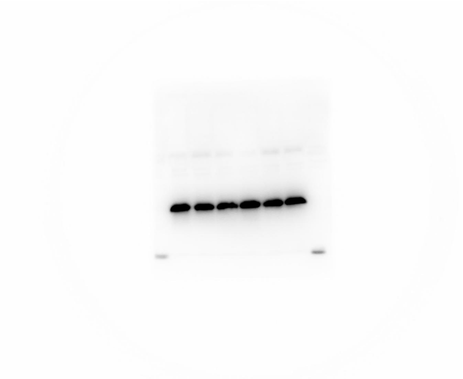
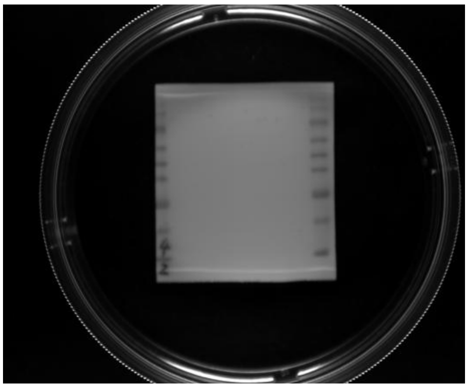

PRDX3 (IP Pellet)

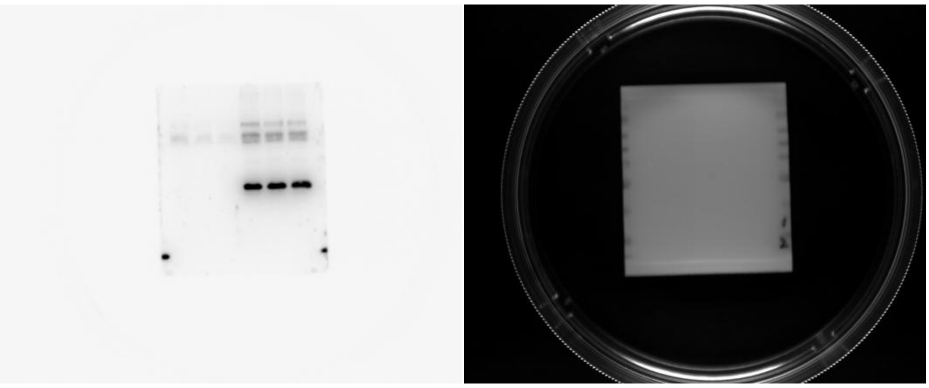

Peroxiredoxin $SO_{2/3}$ (Input)

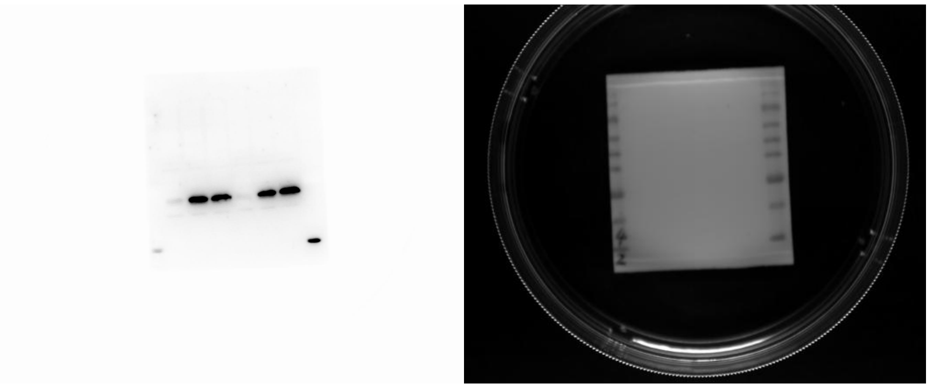

Peroxiredoxin $SO_{2/3}$ (IP Pellet)

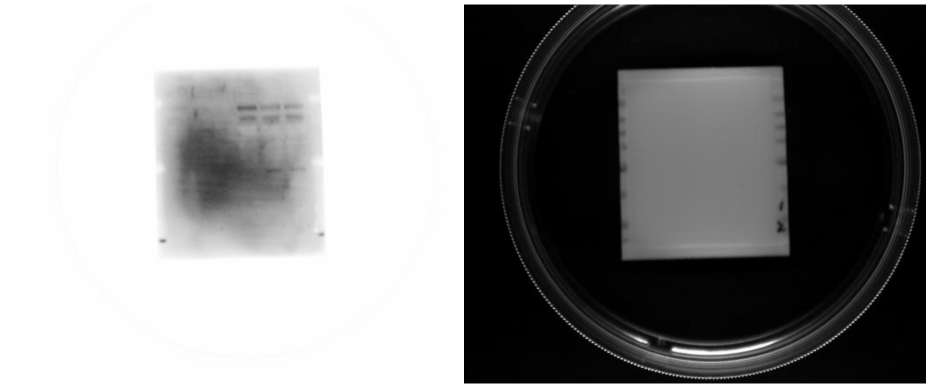

Fig. 7J First Repetition

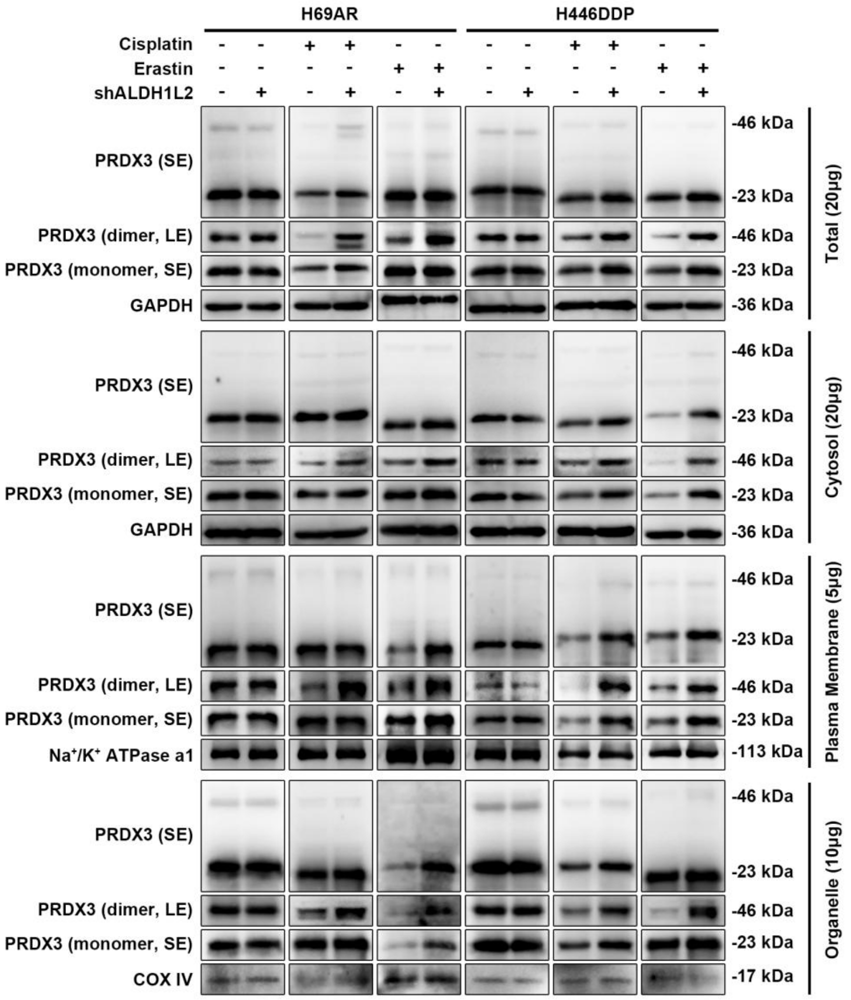

H69AR

PRDX3 (SE, untreated, total)

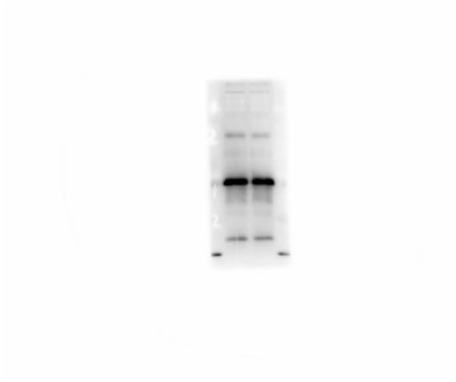

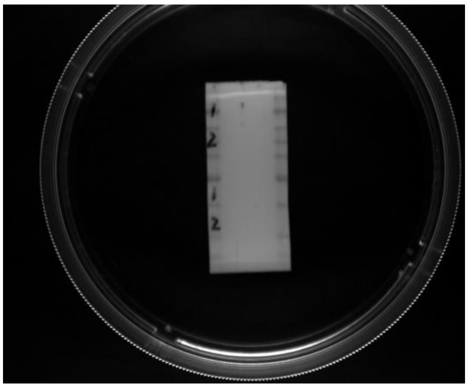

PRDX3 (dimer, LE, untreated, total)

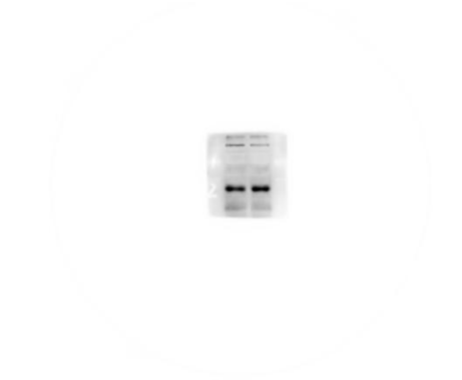

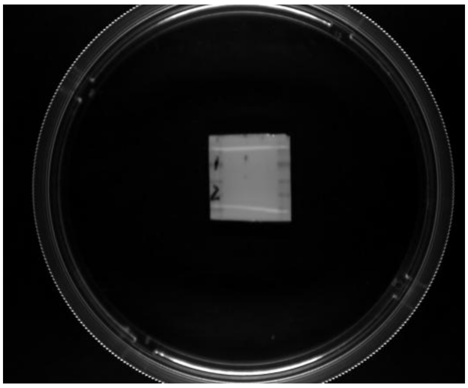

PRDX3 (monomer, SE, untreated, total)

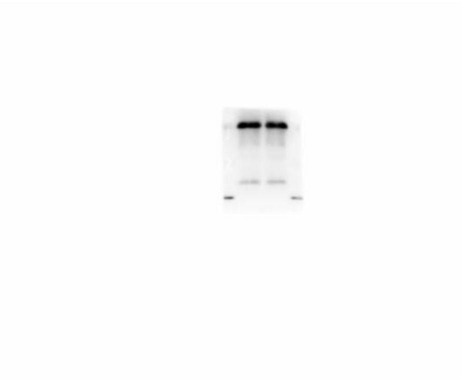

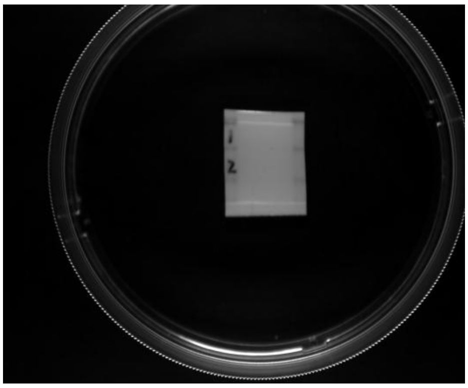

GAPDH (untreated, total)

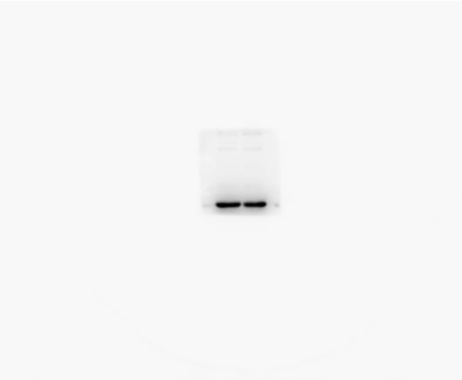

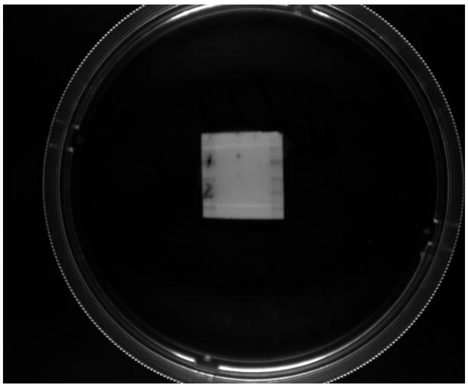

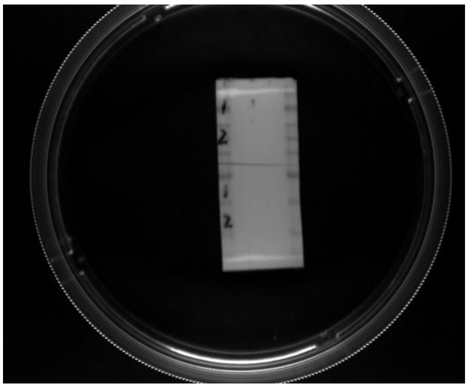

PRDX3 (SE, untreated, cytosol)

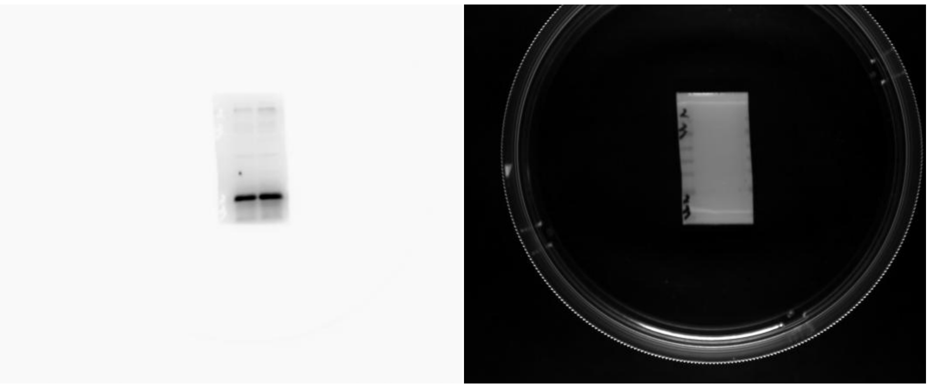

PRDX3 (dimer, LE, untreated, cytosol)

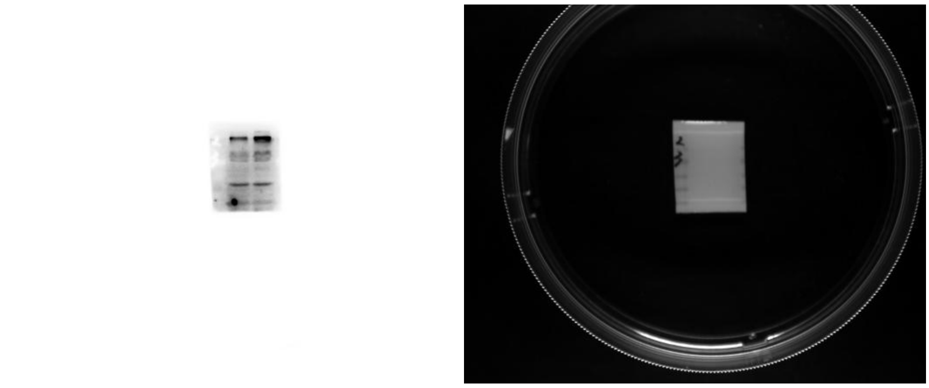

PRDX3 (monomer, SE, untreated, cytosol)

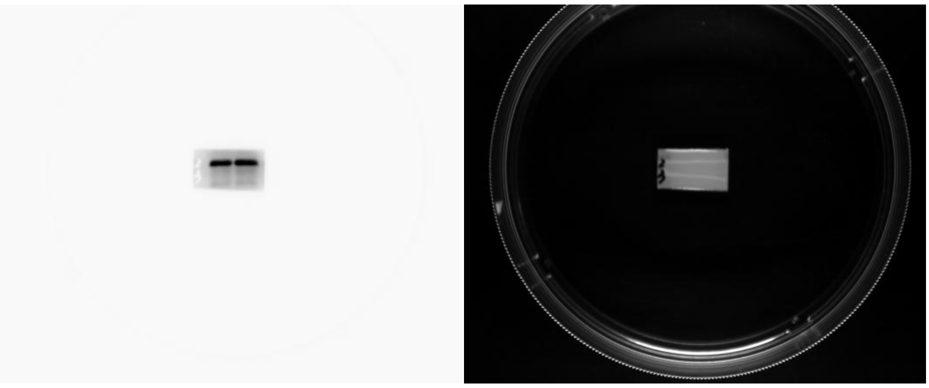

GAPDH (untreated, cytosol)

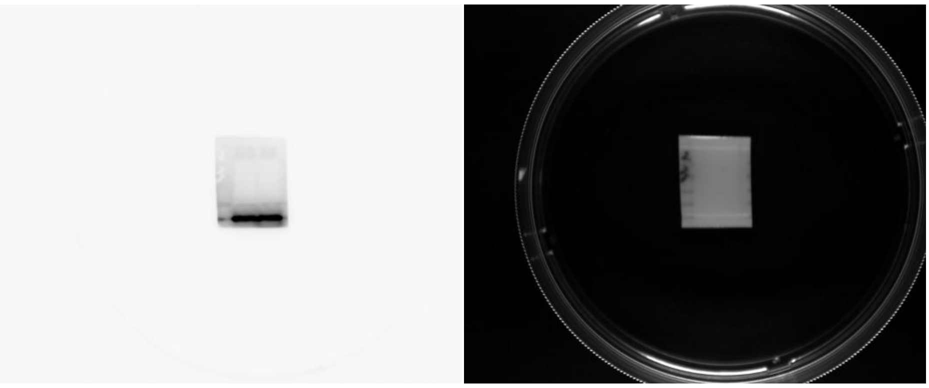

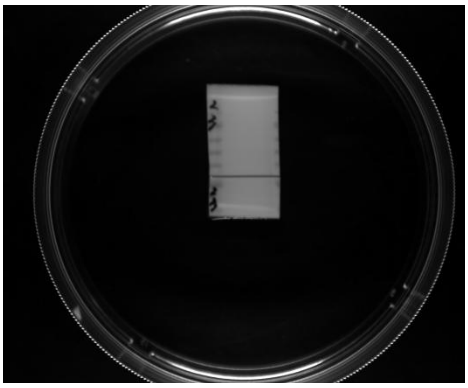

PRDX3 (SE, untreated, plasma membrane)

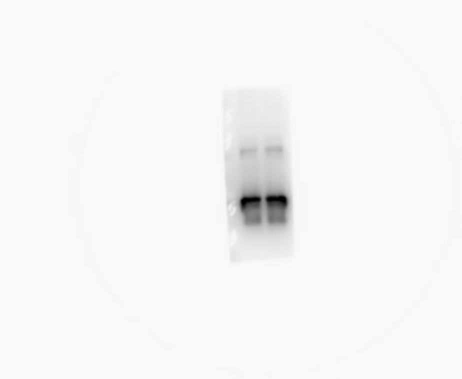

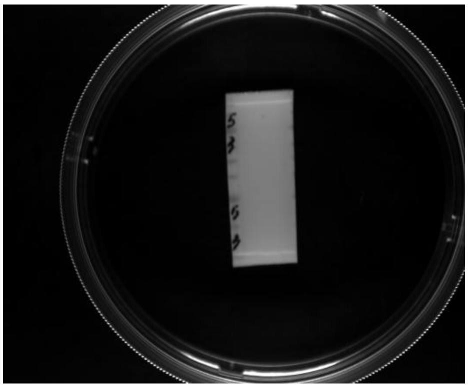

PRDX3 (dimer, LE, untreated, plasma membrane)

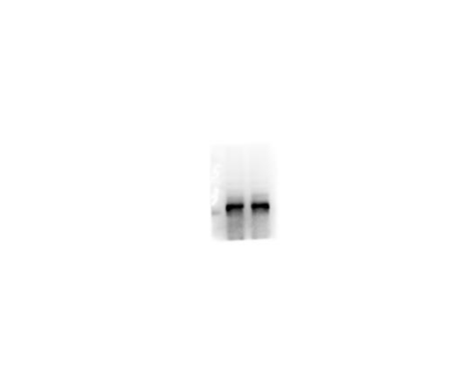

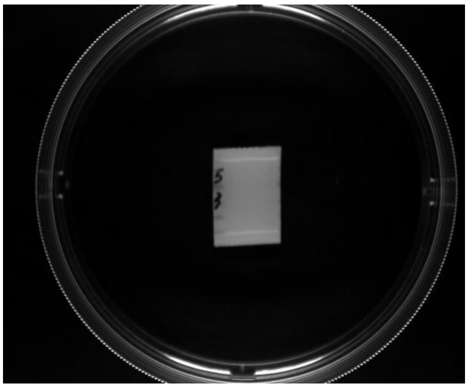

PRDX3 (monomer, SE, untreated, plasma membrane)

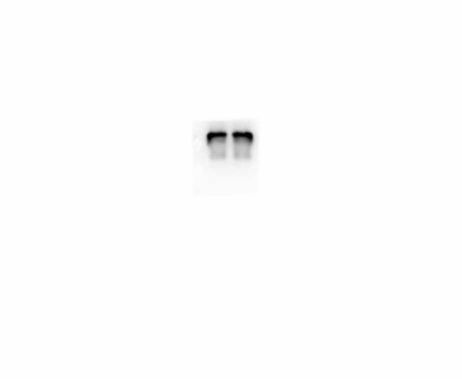

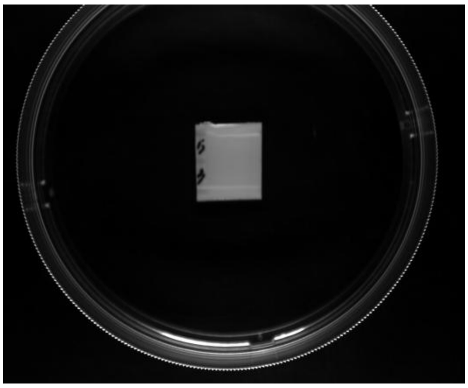

Na^{+}/K^{+} ATPase a1 (untreated, plasma membrane)

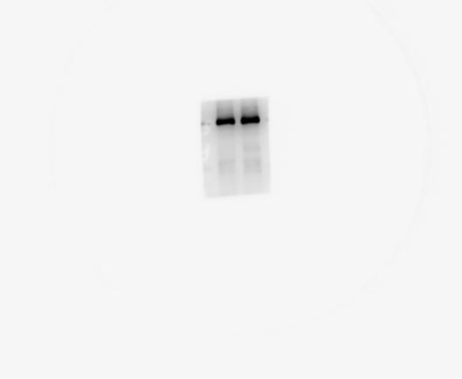

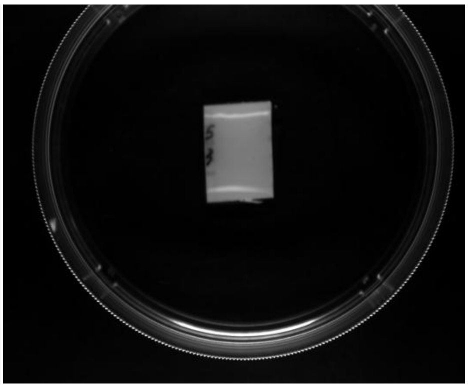

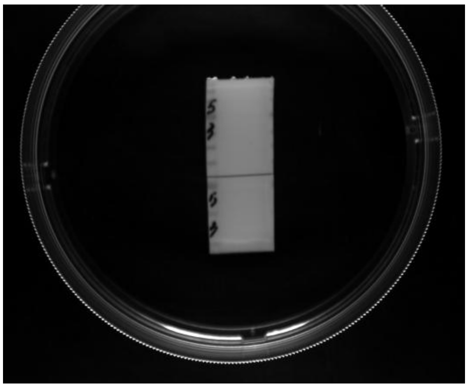

PRDX3 (SE, untreated, organelle)

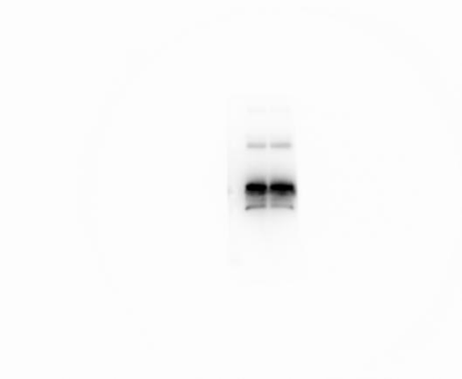

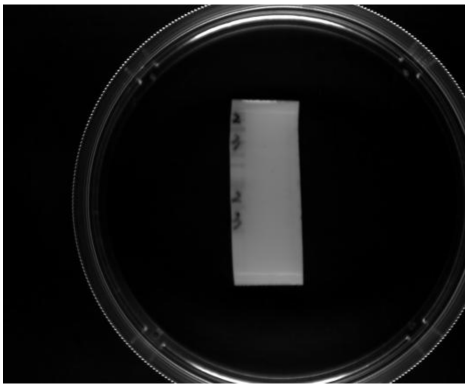

PRDX3 (dimer, LE, untreated, organelle)

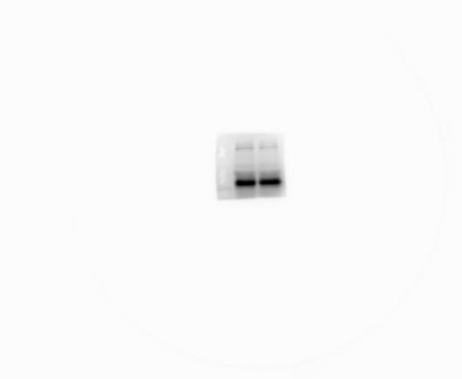

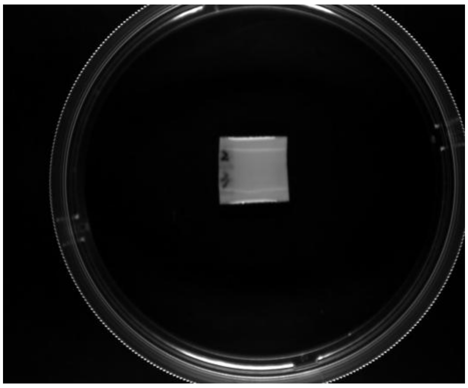

PRDX3 (monomer, SE, untreated, organelle)

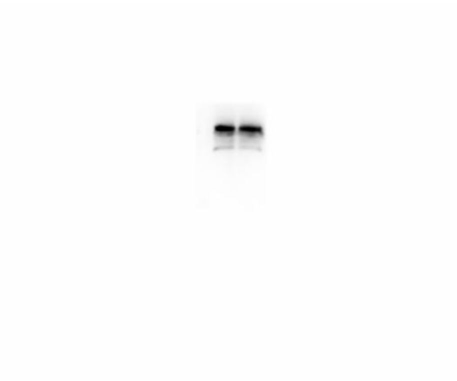

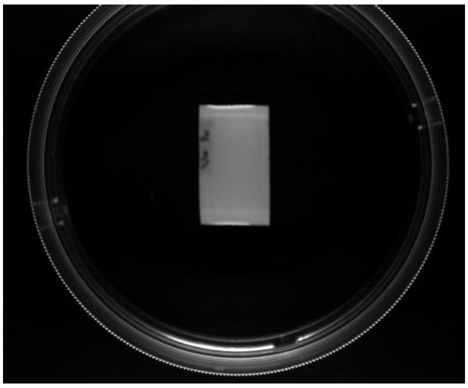

COX IV (untreated, organelle)

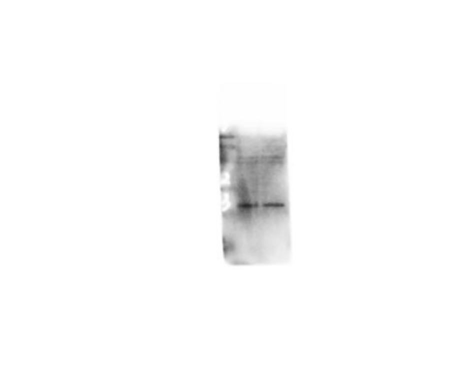

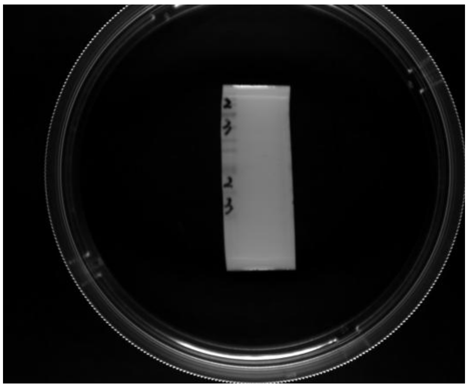

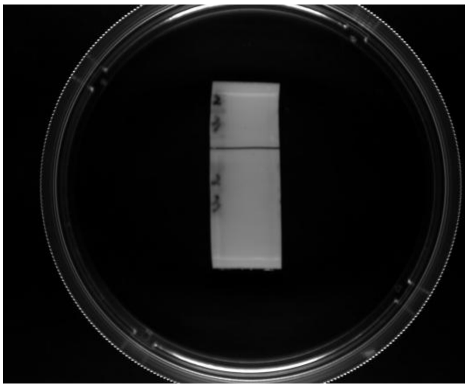

PRDX3 (SE, cisplatin, total)

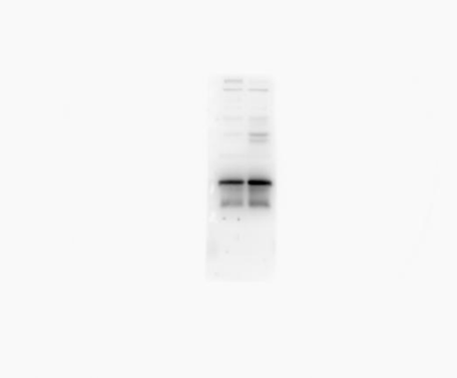

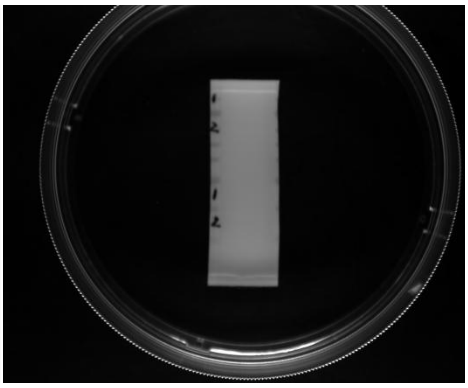

PRDX3 (dimer, LE, cisplatin, total)

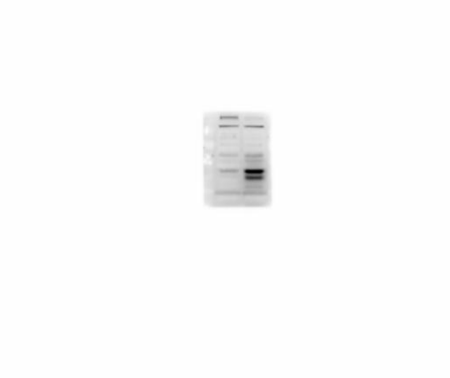

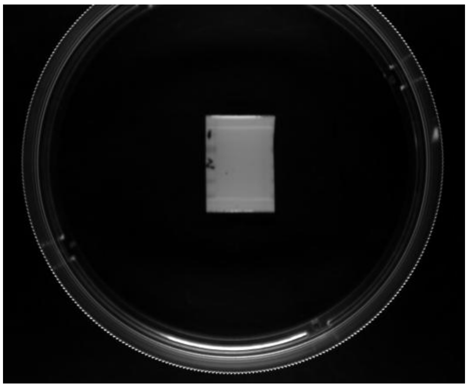

PRDX3 (monomer, SE, cisplatin, total)

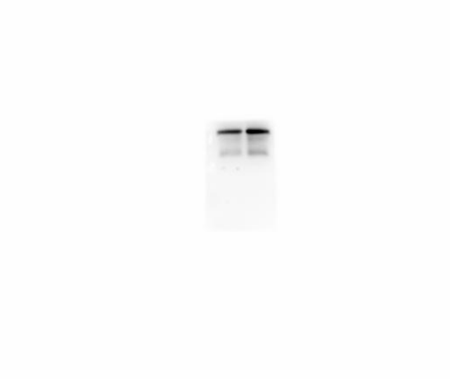

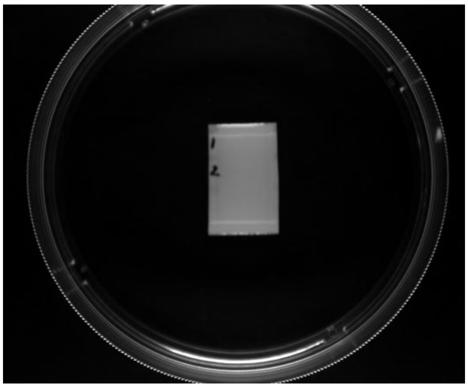

GAPDH (cisplatin, total)

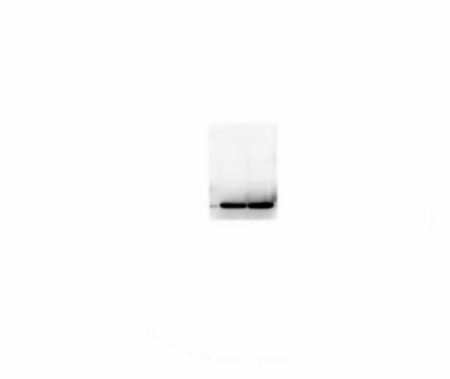

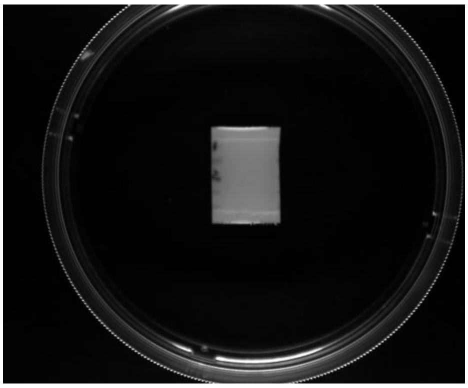

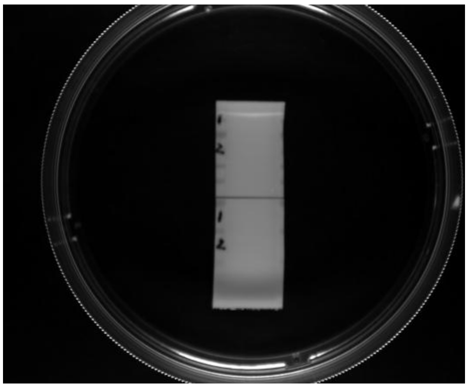

PRDX3 (SE, cisplatin, cytosol)

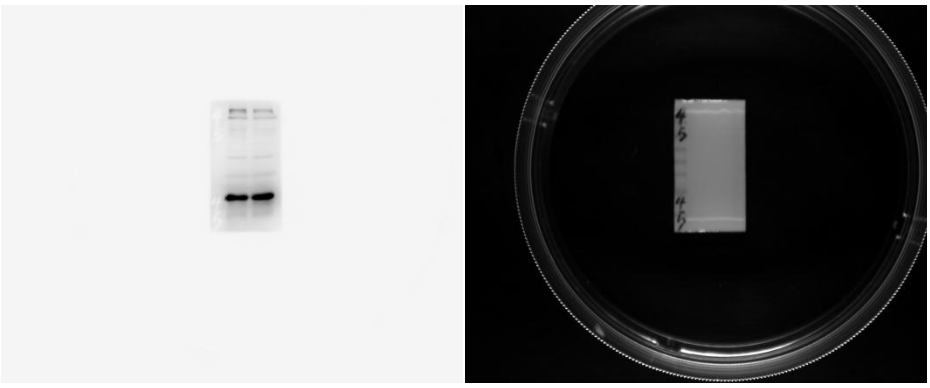

PRDX3 (dimer, LE, cisplatin, cytosol)

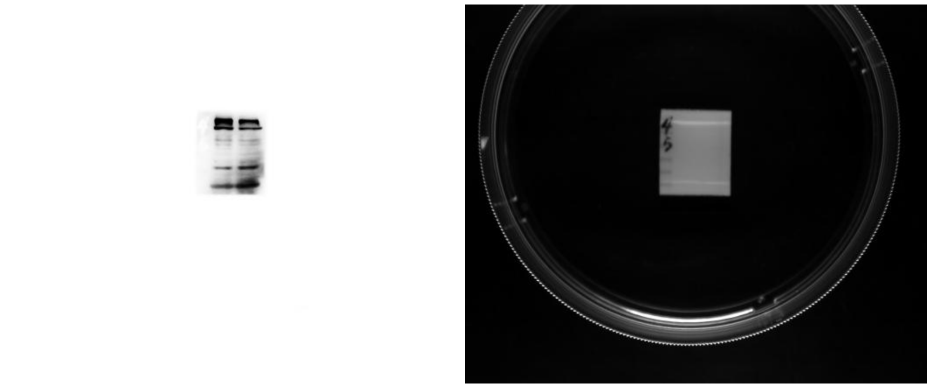

PRDX3 (monomer, SE, cisplatin, cytosol)

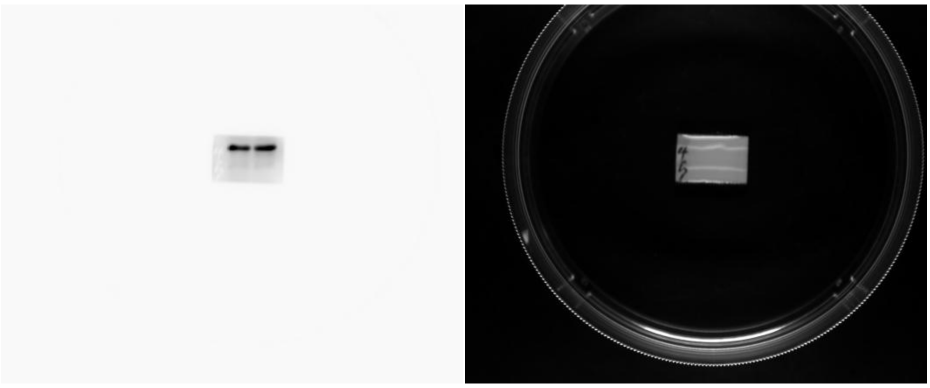

GAPDH (cisplatin, cytosol)

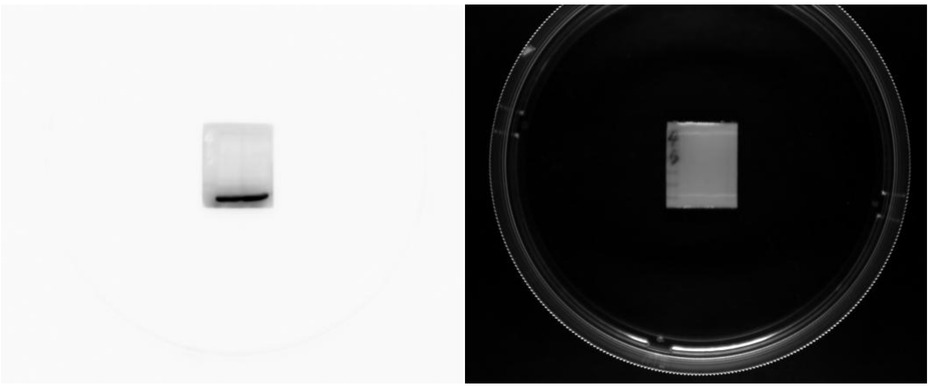

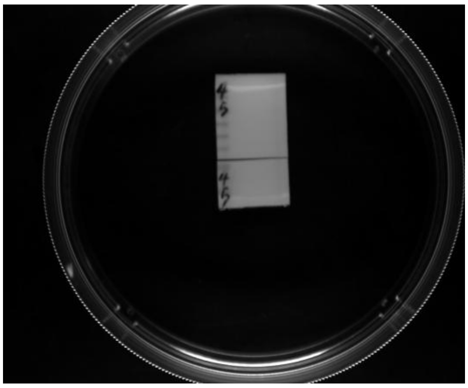

PRDX3 (SE, cisplatin, plasma membrane)

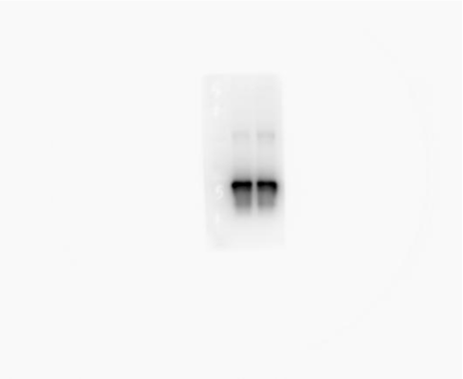 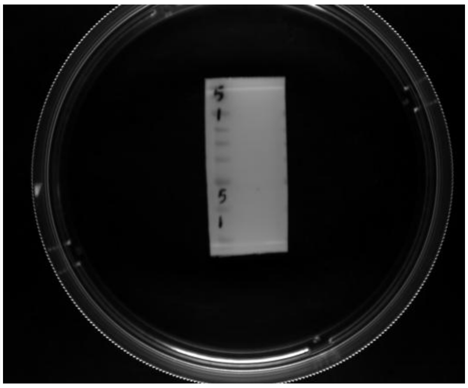

PRDX3 (dimer, LE, cisplatin, plasma membrane)

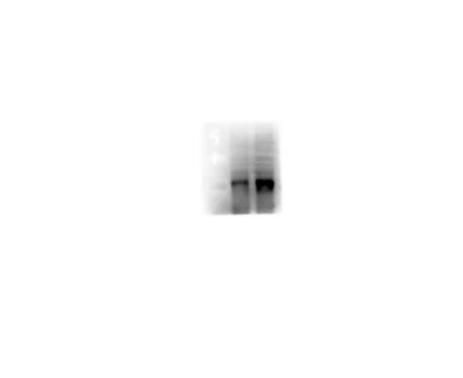 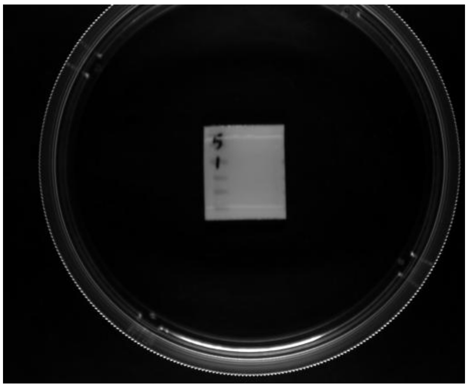

PRDX3 (monomer, SE, cisplatin, plasma membrane)

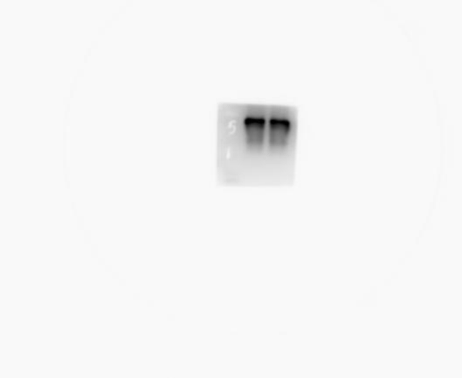 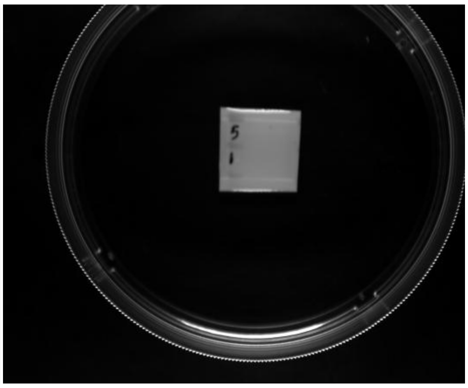

Na$^+$/K$^+$ ATPase a1 (cisplatin, plasma membrane)

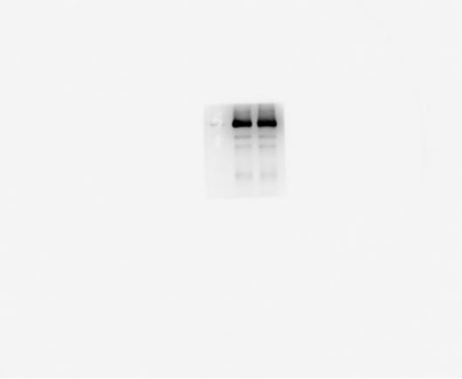

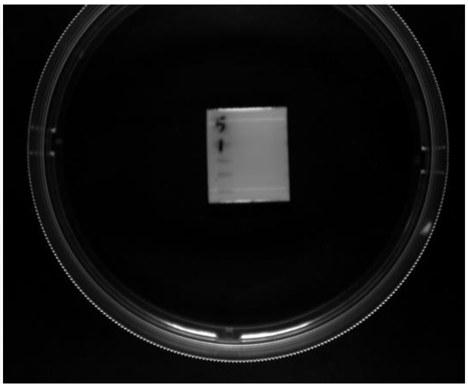

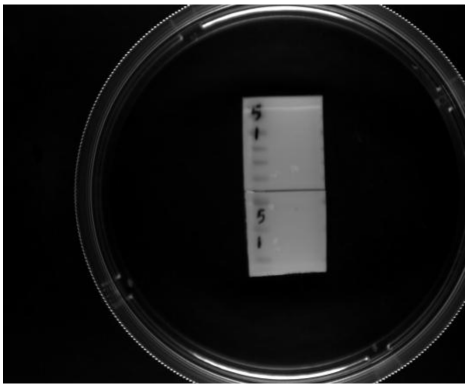

PRDX3 (SE, cisplatin, organelle)

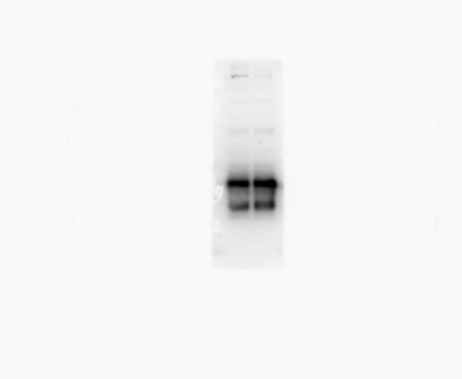

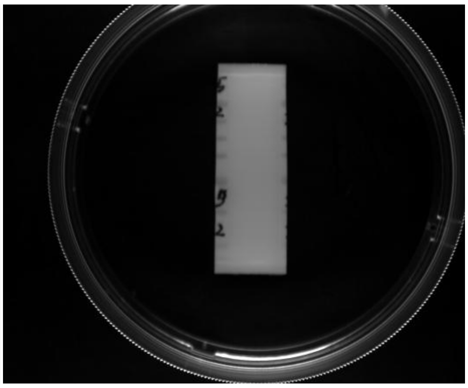

PRDX3 (dimer, LE, cisplatin, organelle)

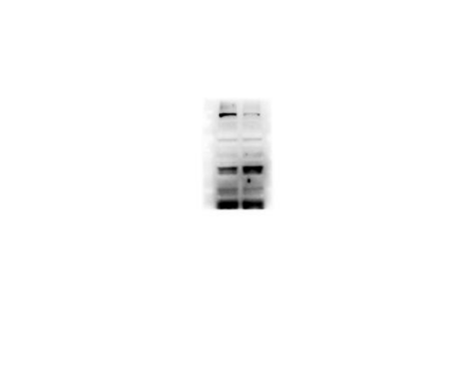

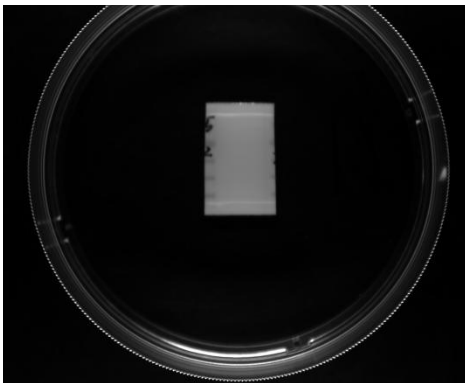

PRDX3 (monomer, SE, cisplatin, organelle)

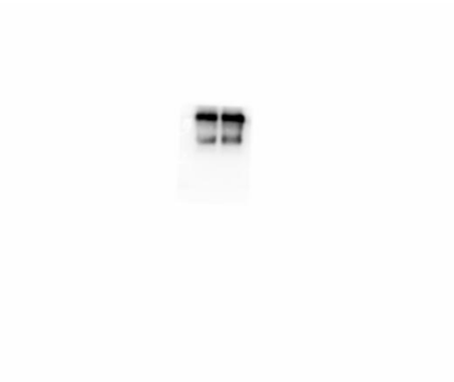

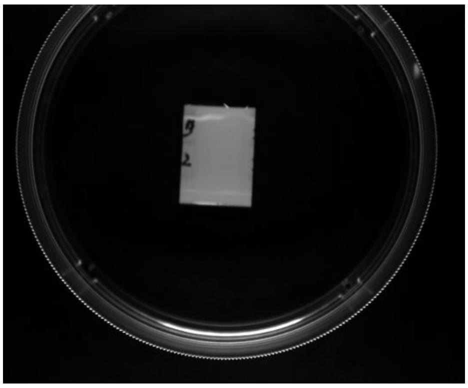

COX IV (cisplatin, organelle)

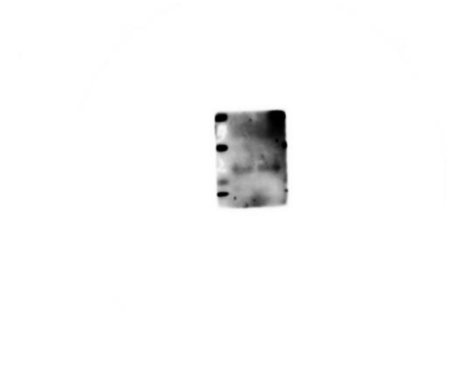

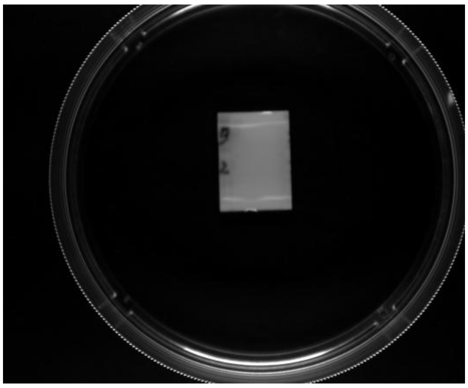

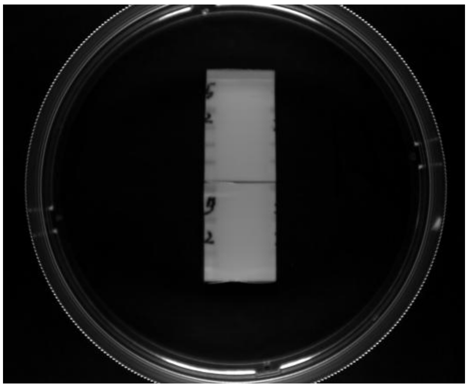

PRDX3 (SE, erastin, total)

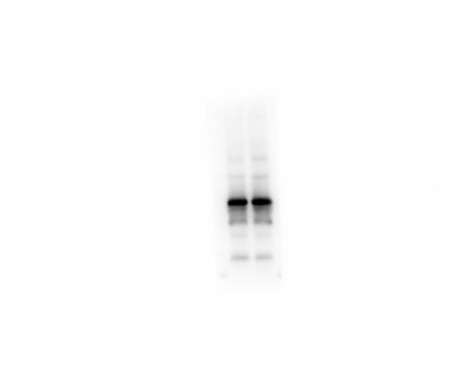

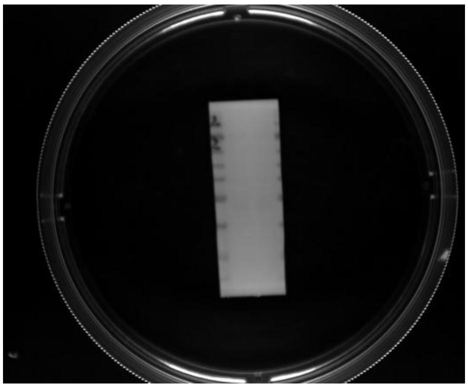

PRDX3 (dimer, LE, erastin, total)

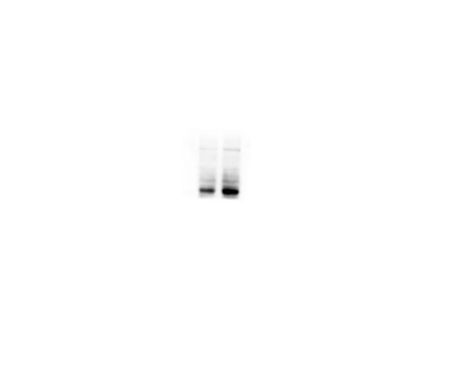

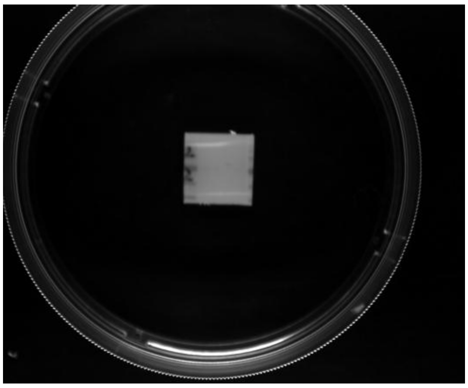

PRDX3 (monomer, SE, erastin, total)

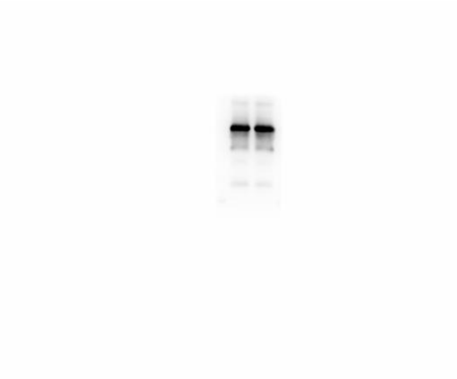

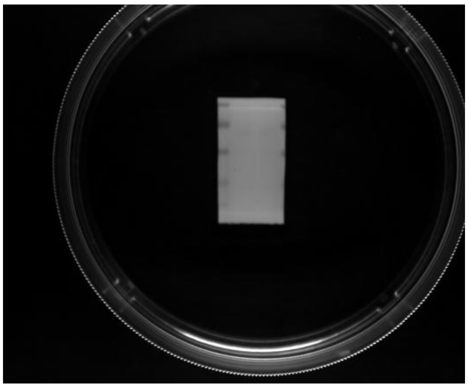

GAPDH (erastin, total)

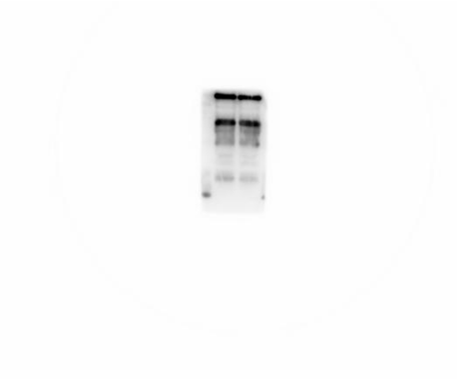

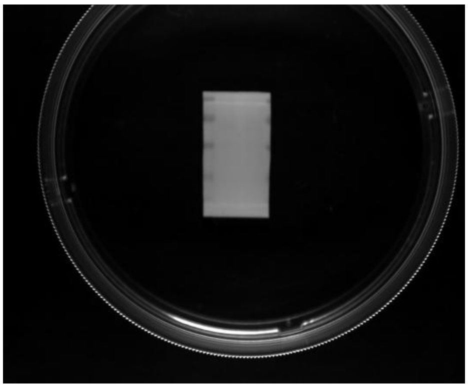

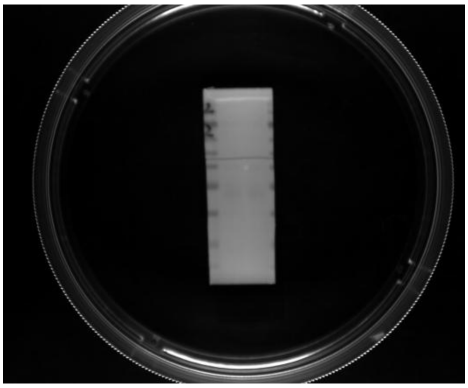

PRDX3 (SE, erastin, cytosol)

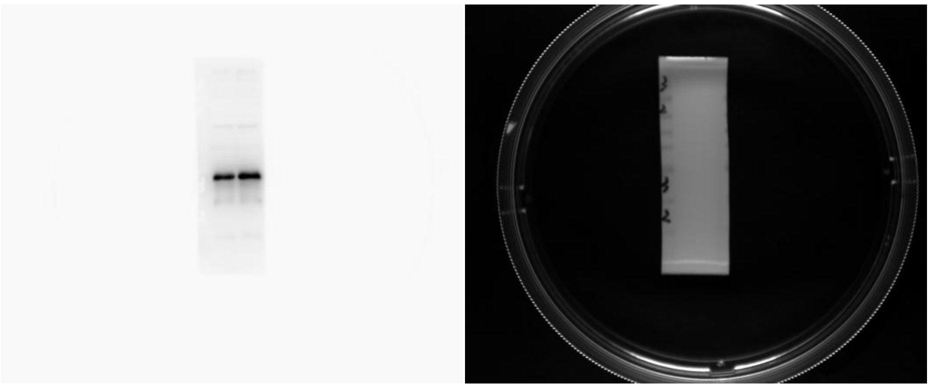

PRDX3 (dimer, LE, erastin, cytosol)

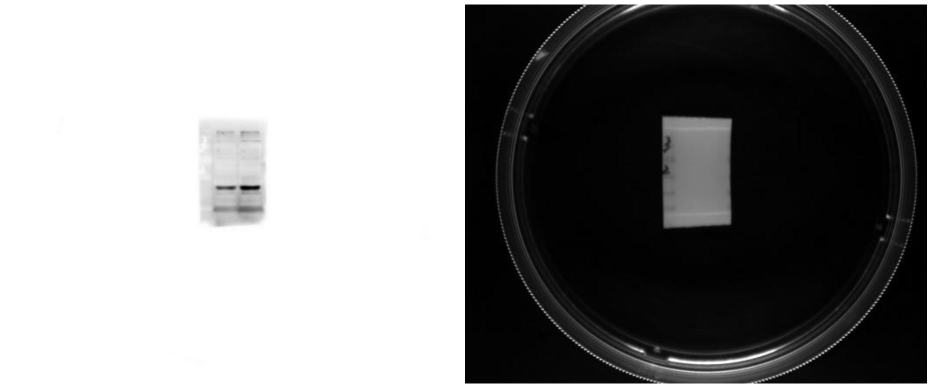

PRDX3 (monomer, SE, erastin, cytosol)

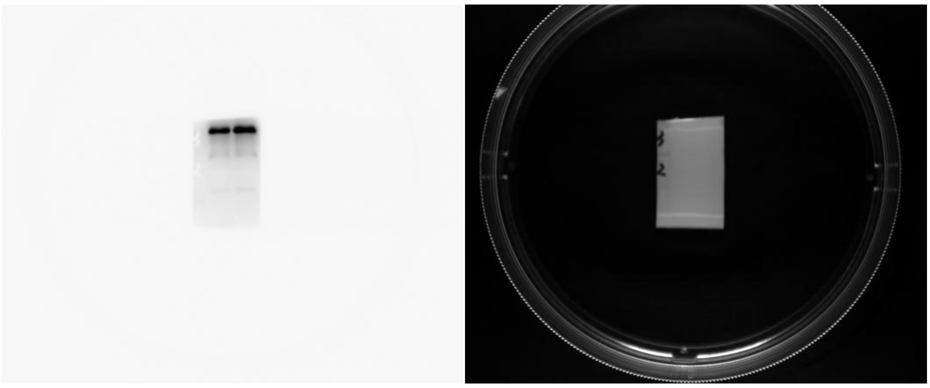

GAPDH (erastin, cytosol)

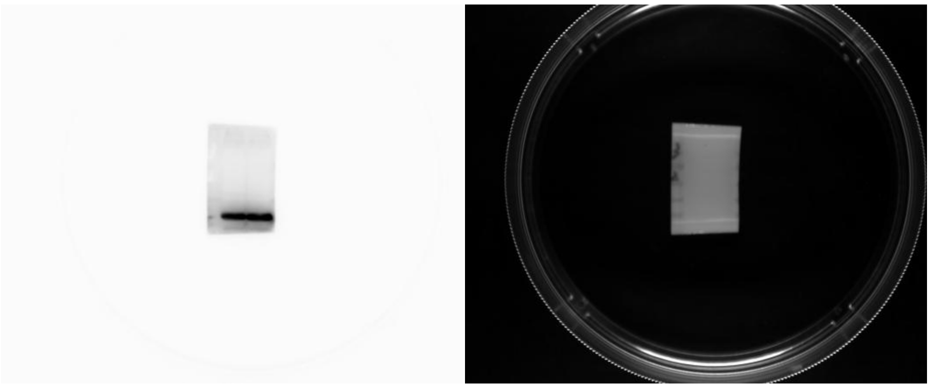

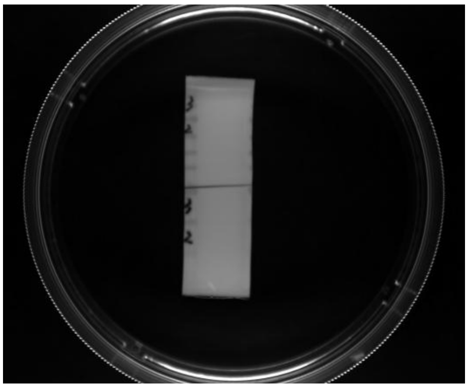

PRDX3 (SE, erastin, plasma membrane)

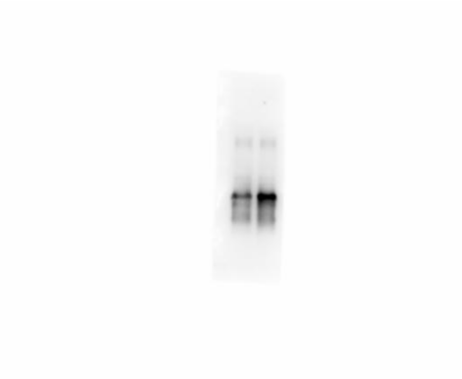

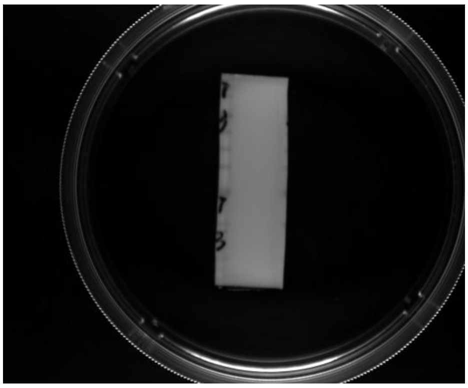

PRDX3 (dimer, LE, erastin, plasma membrane)

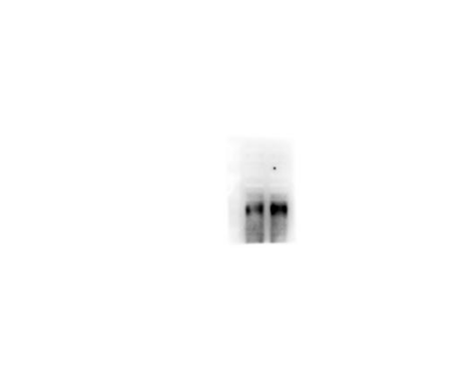

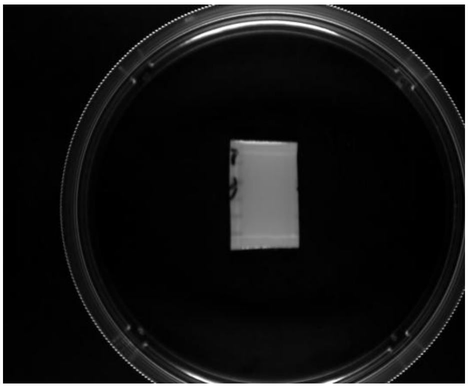

PRDX3 (monomer, SE, erastin, plasma membrane)

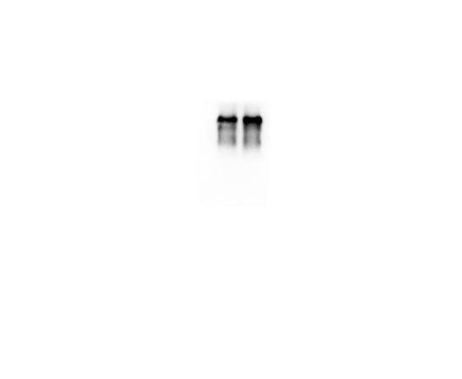

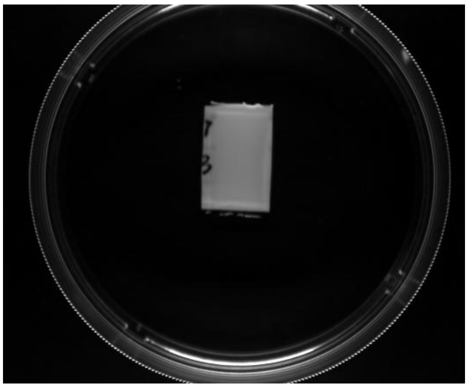

Na^{+}/K^{+} ATPase a1 (erastin, plasma membrane)

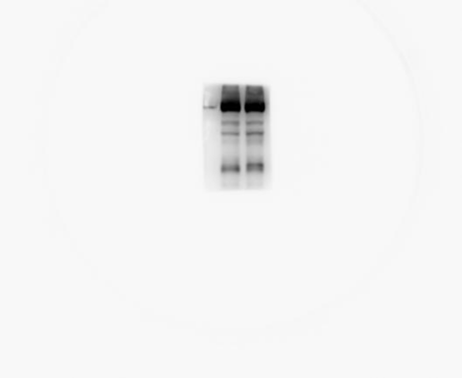
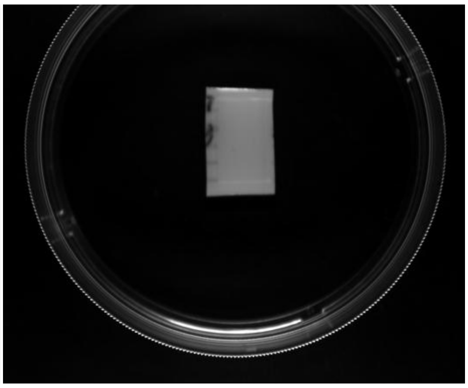

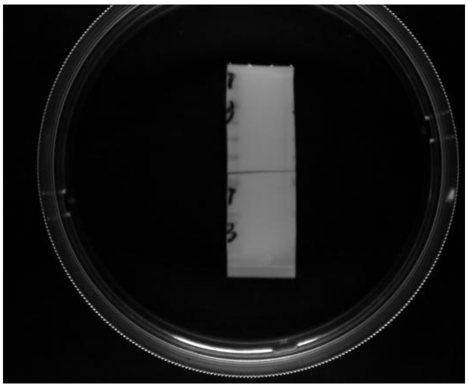

PRDX3 (SE, erastin, organelle)

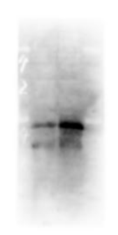
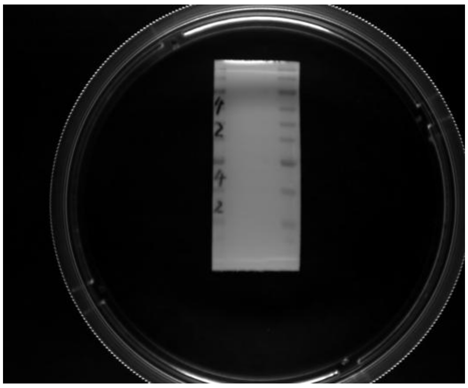

PRDX3 (dimer, LE, erastin, organelle)

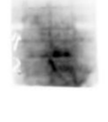
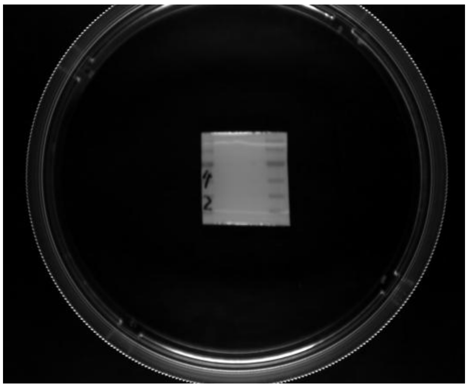

PRDX3 (monomer, SE, erastin, organelle)

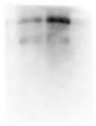

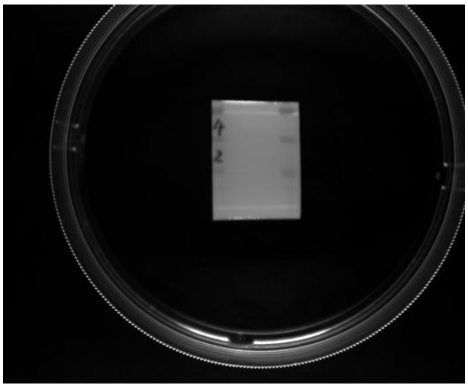

COX IV (erastin, organelle)

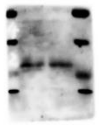

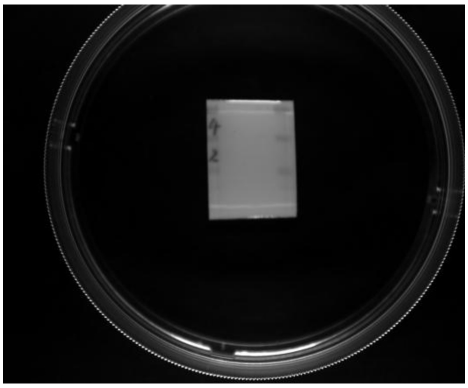

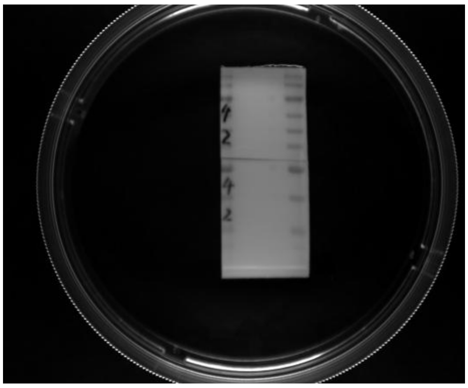

H446DDP

PRDX3 (SE, untreated, total)

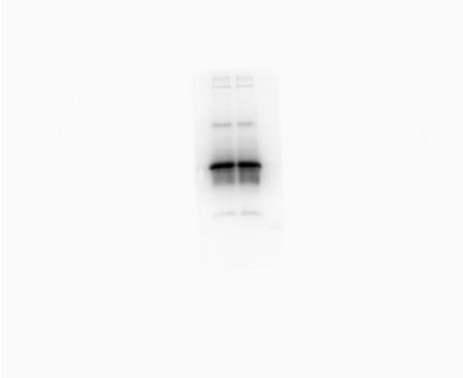

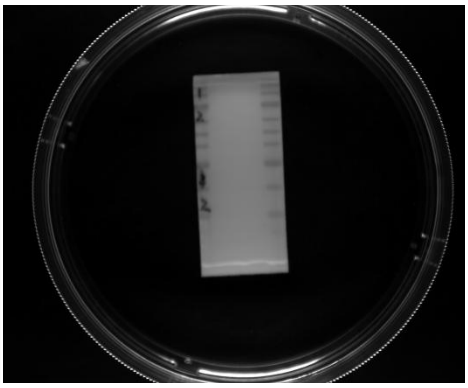

PRDX3 (dimer, LE, untreated, total)

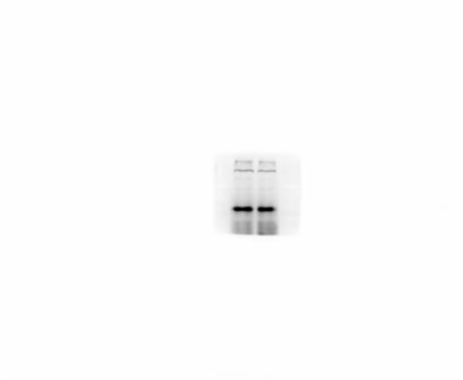

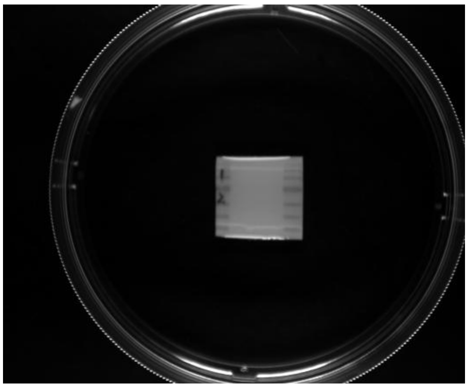

PRDX3 (monomer, SE, untreated, total)

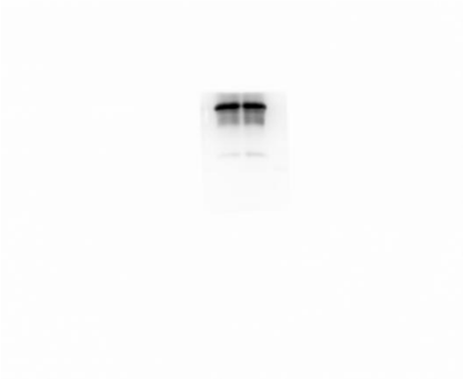

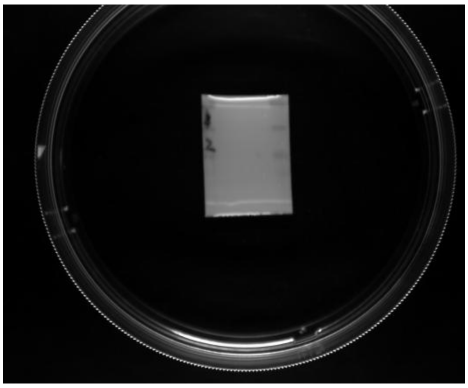

GAPDH (untreated, total)

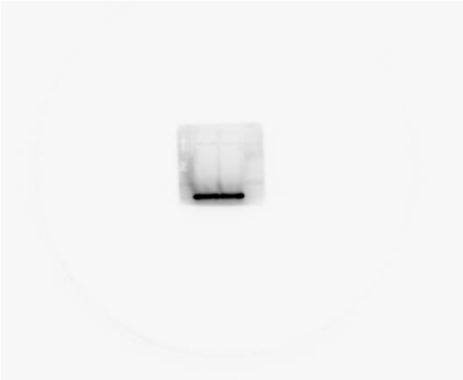

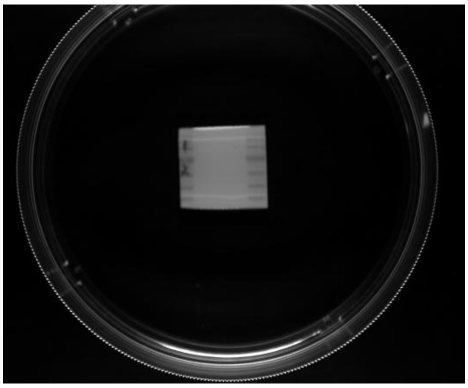

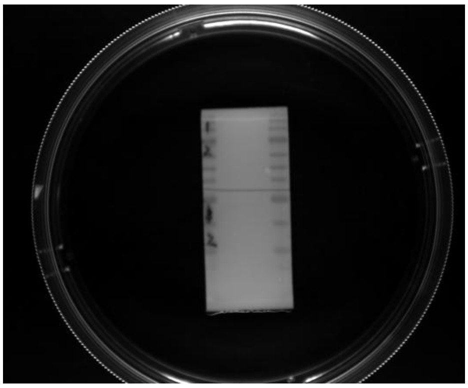

PRDX3 (SE, untreated, cytosol)

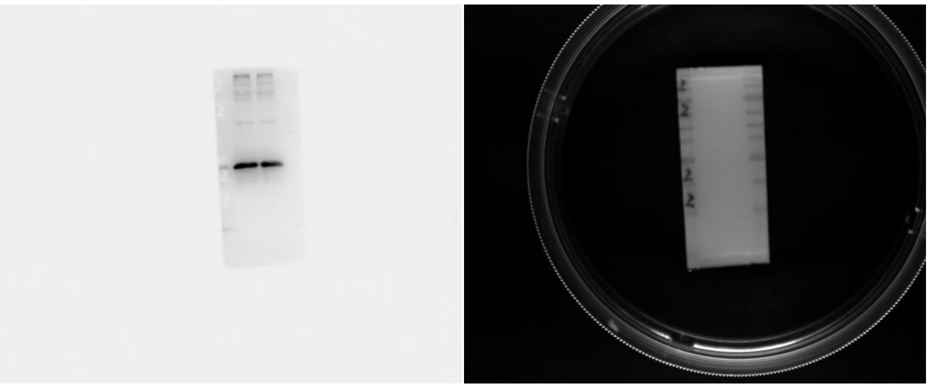

PRDX3 (dimer, LE, untreated, cytosol)

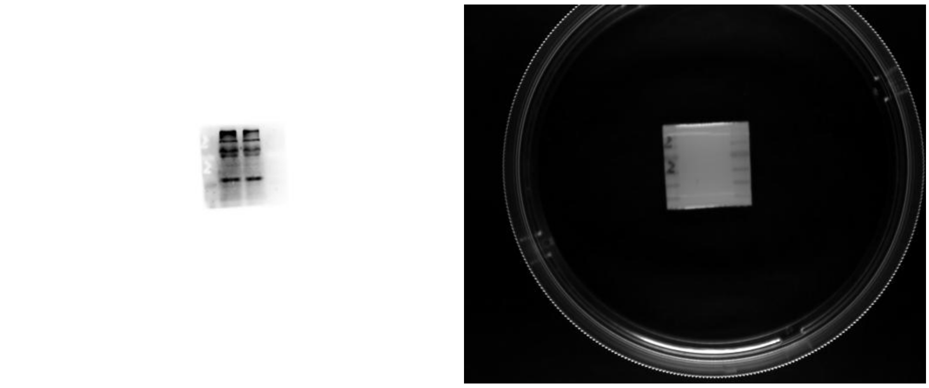

PRDX3 (monomer, SE, untreated, cytosol)

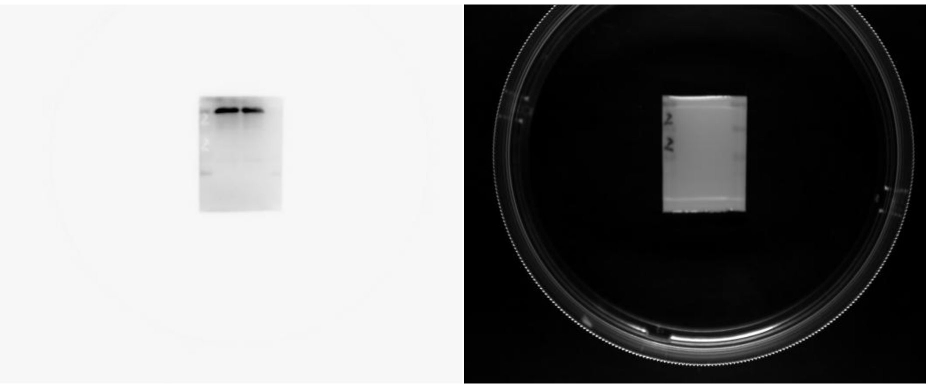

GAPDH (untreated, cytosol)

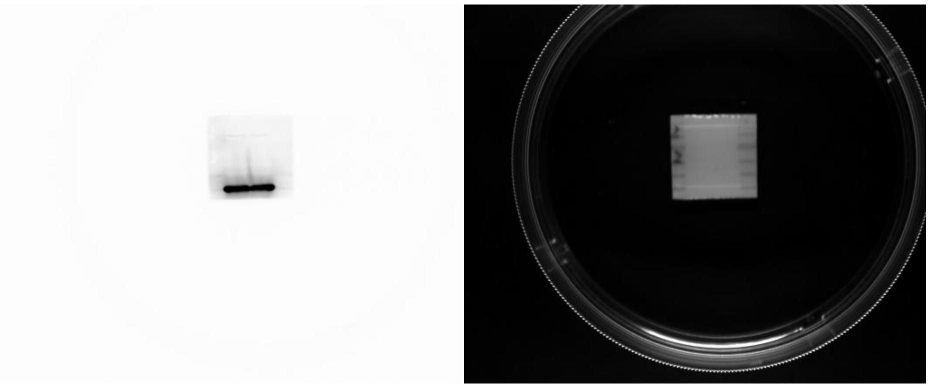

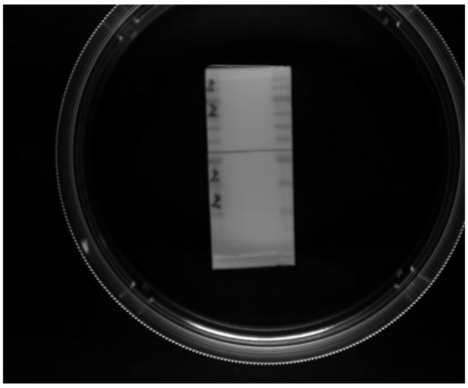

PRDX3 (SE, untreated, plasma membrane)

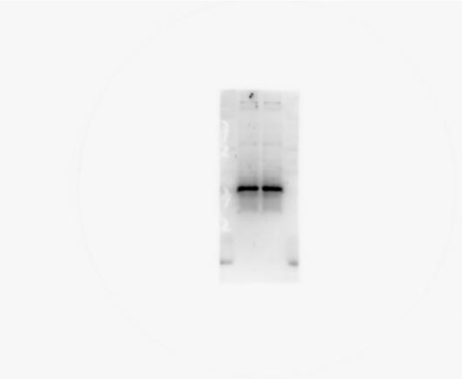

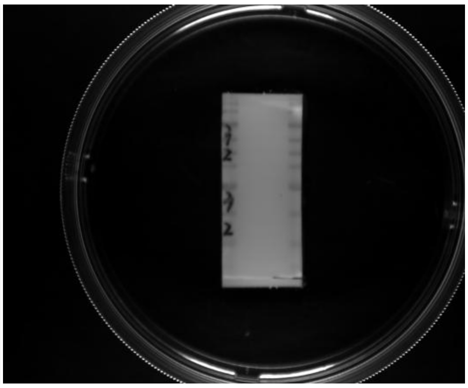

PRDX3 (dimer, LE, untreated, plasma membrane)

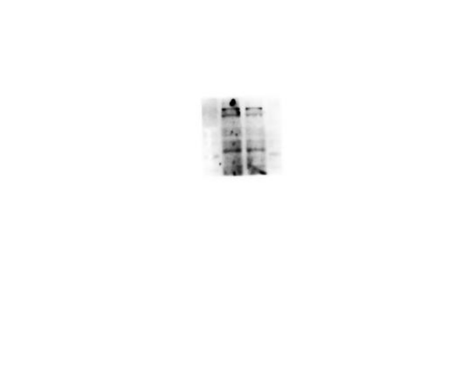

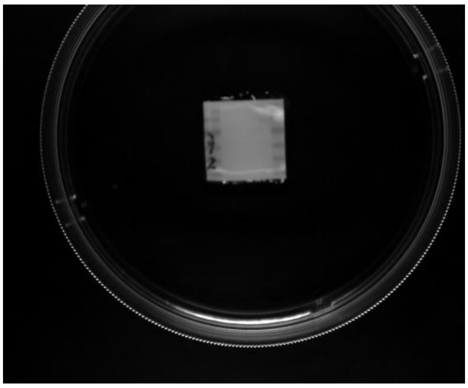

PRDX3 (monomer, SE, untreated, plasma membrane)

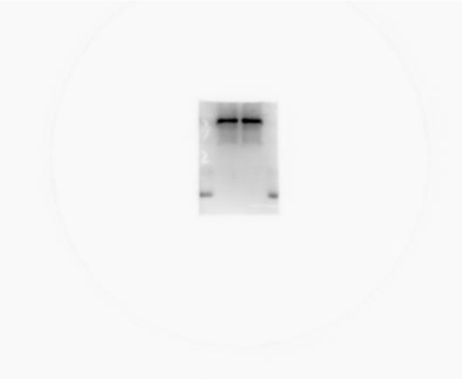

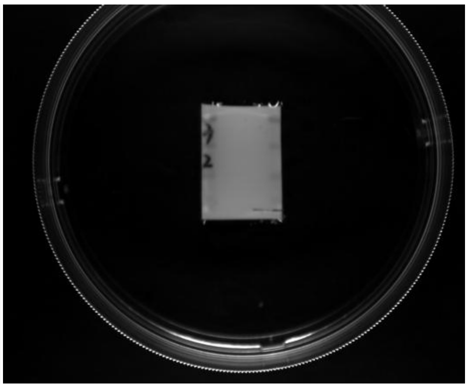

Na^{+}/K^{+} ATPase a1 (untreated, plasma membrane)

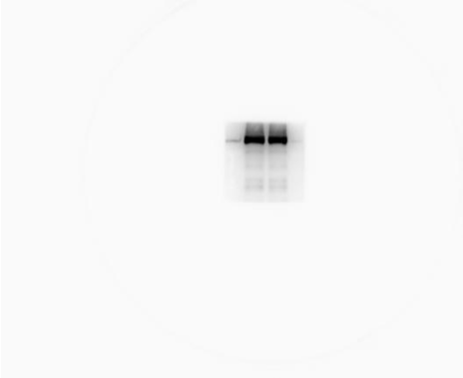
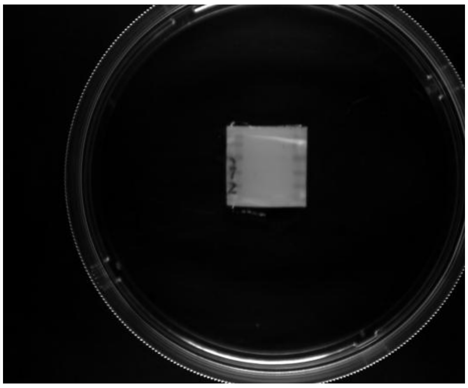

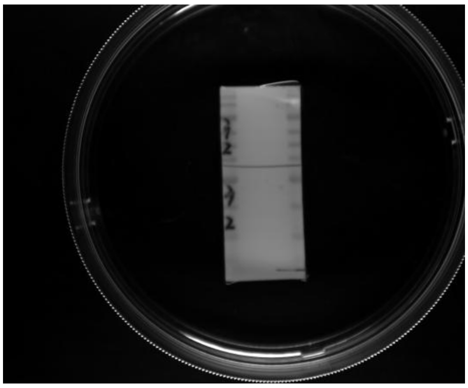

PRDX3 (SE, untreated, organelle)

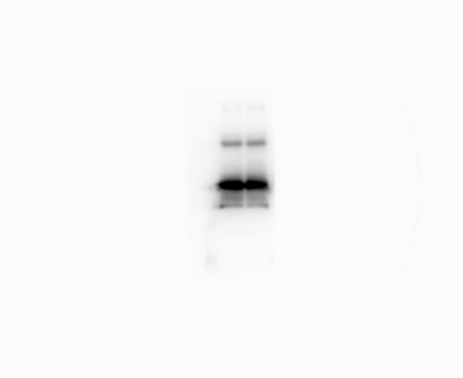
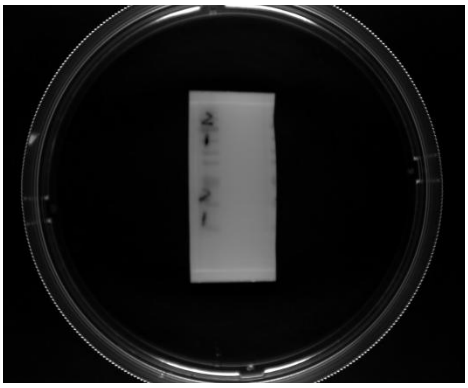

PRDX3 (dimer, LE, untreated, organelle)

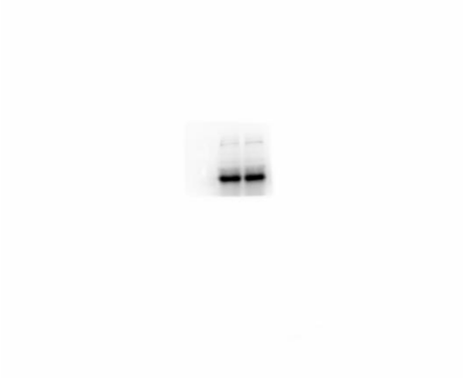
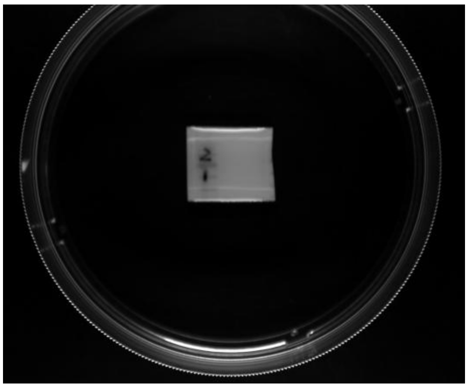

PRDX3 (monomer, SE, untreated, organelle)

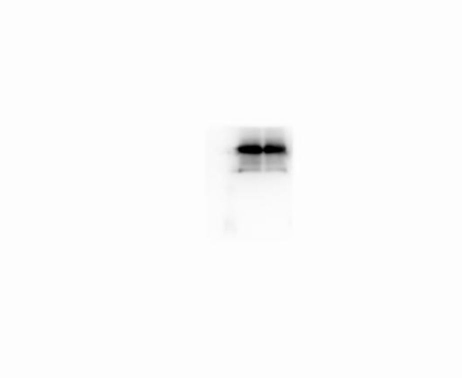

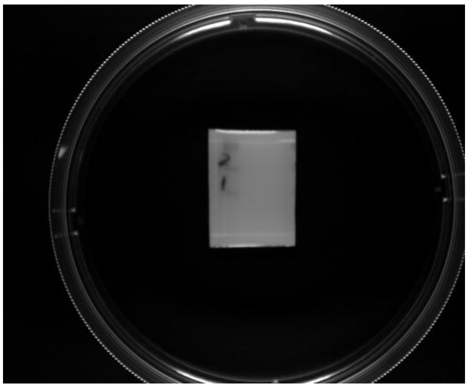

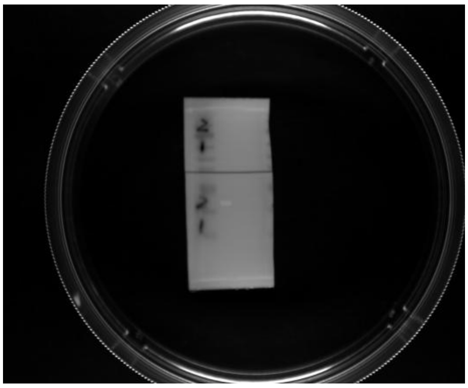

COX IV (untreated, organelle)

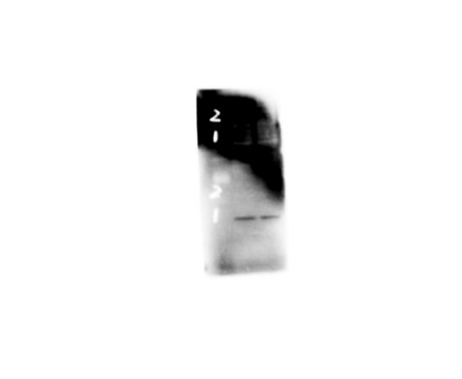

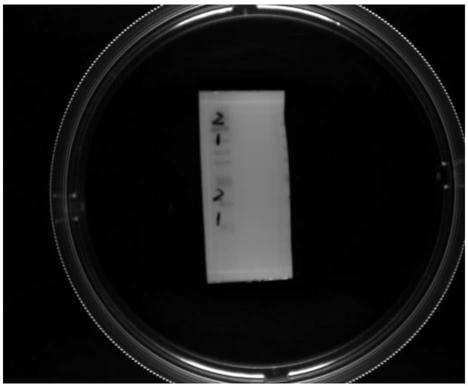

PRDX3 (SE, cisplatin, total)

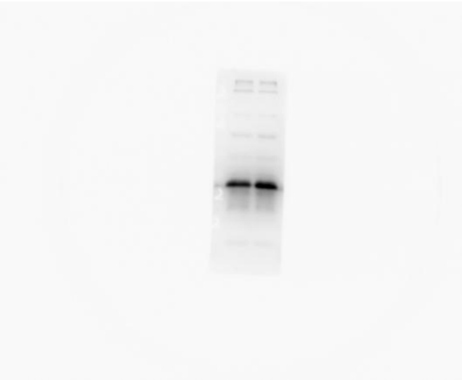

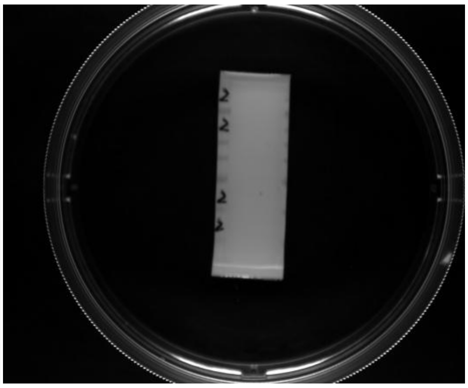

PRDX3 (dimer, LE, cisplatin, total)

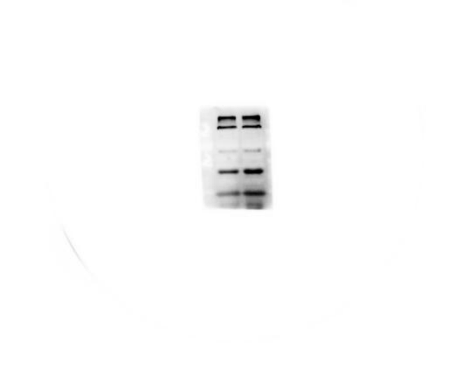

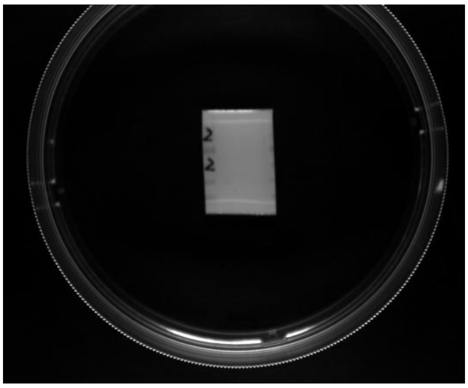

PRDX3 (monomer, SE, cisplatin, total)

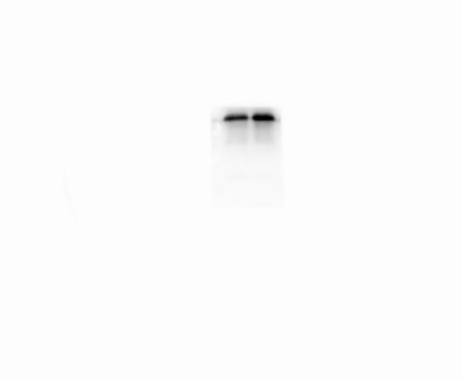

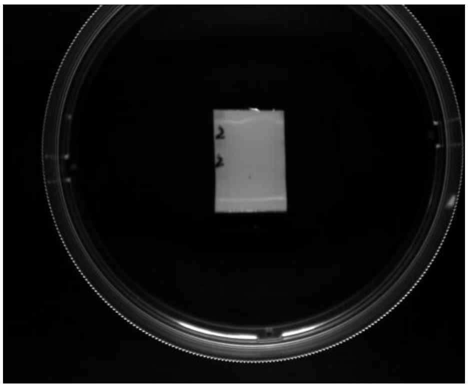

GAPDH (cisplatin, total)

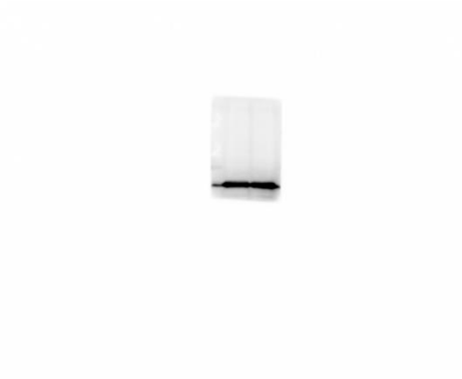

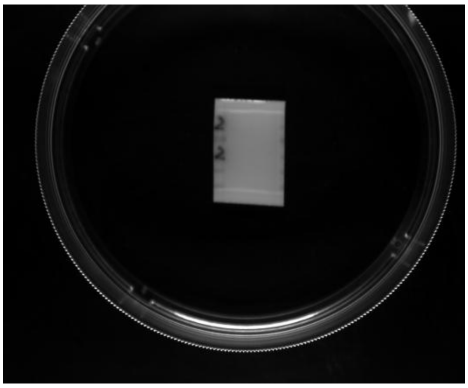

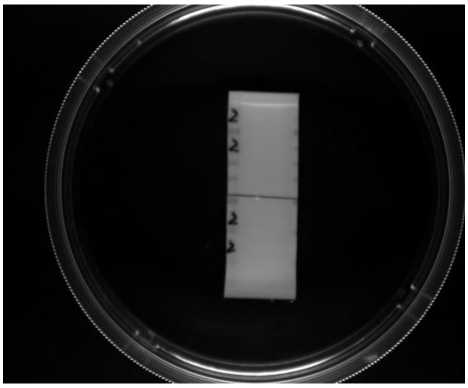

PRDX3 (SE, cisplatin, cytosol)

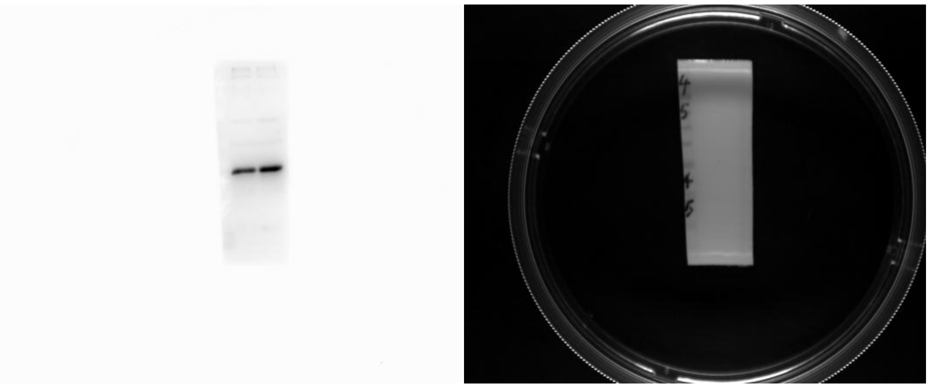

PRDX3 (dimer, LE, cisplatin, cytosol)

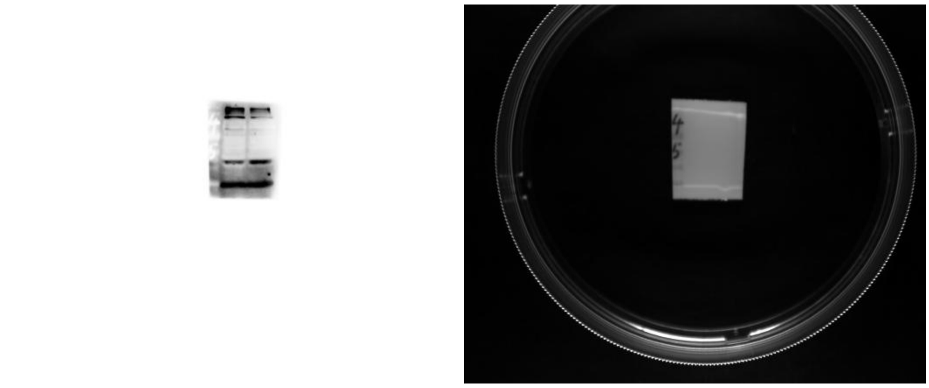

PRDX3 (monomer, SE, cisplatin, cytosol)

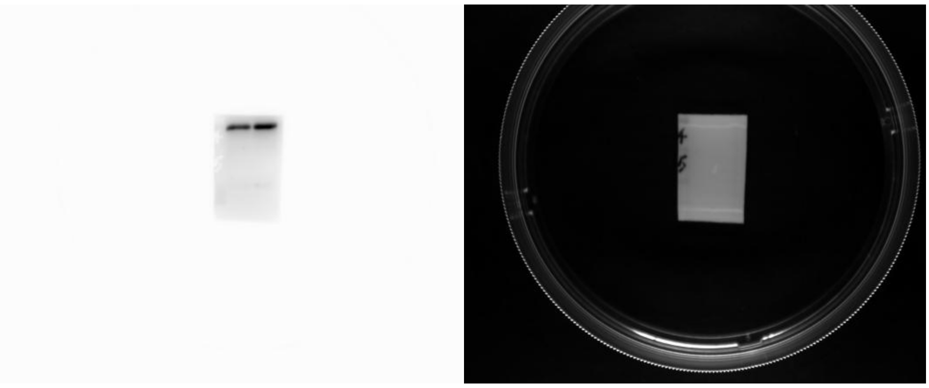

GAPDH (cisplatin, cytosol)

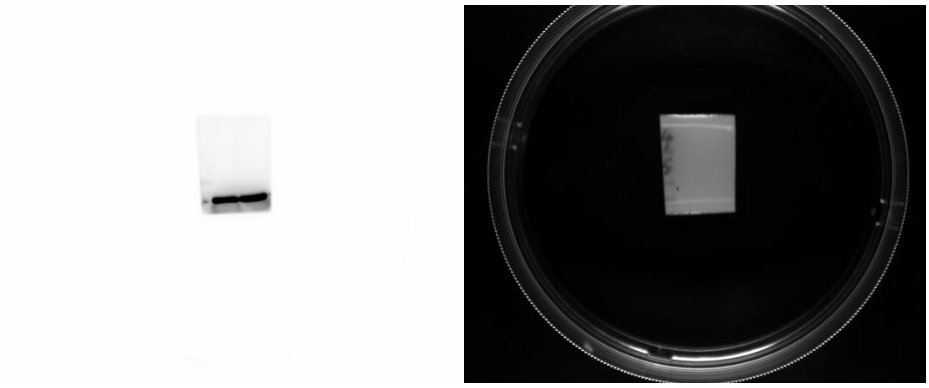

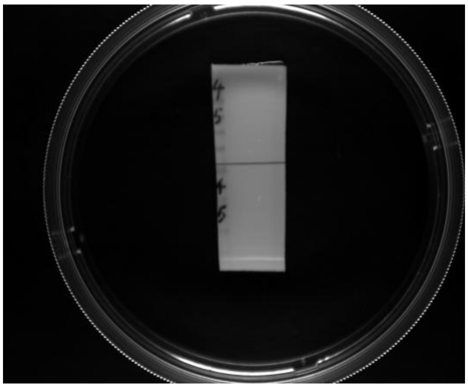

PRDX3 (SE, cisplatin, plasma membrane)

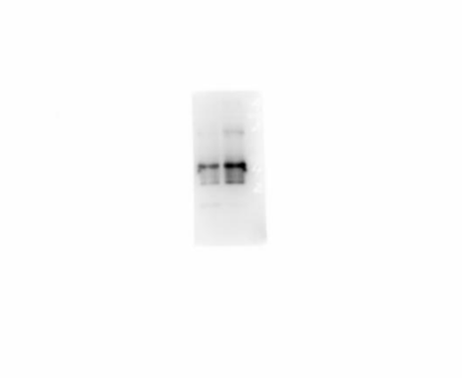

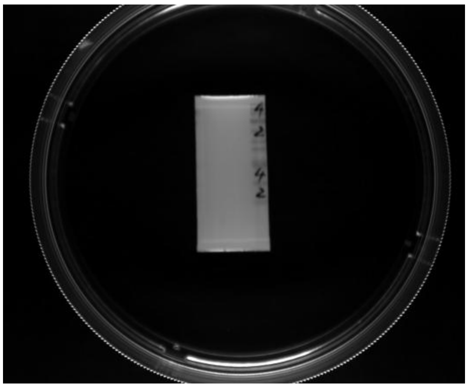

PRDX3 (dimer, LE, cisplatin, plasma membrane)

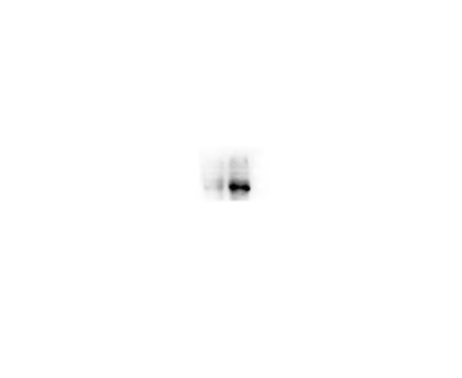

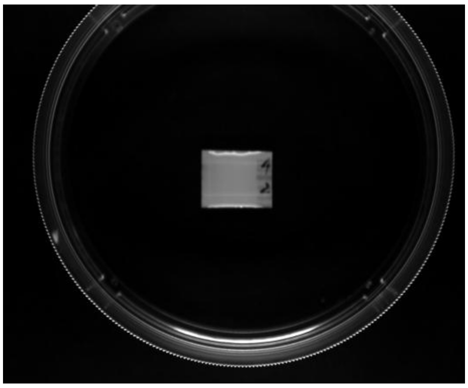

PRDX3 (monomer, SE, cisplatin, plasma membrane)

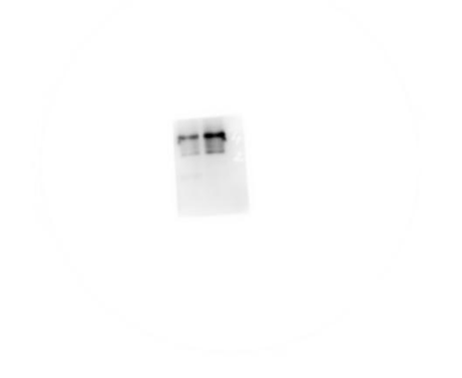

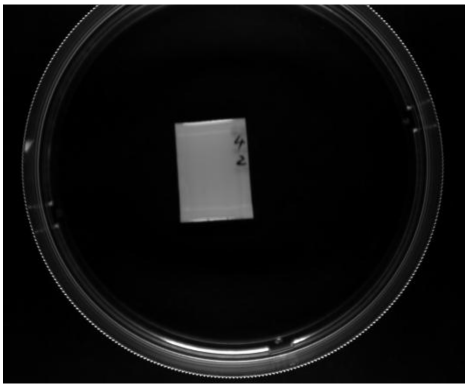

Na^+/K^+ ATPase a1 (cisplatin, plasma membrane)

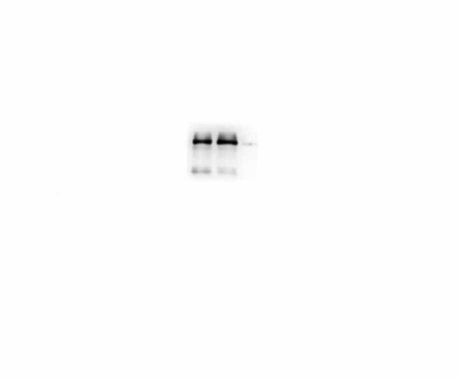
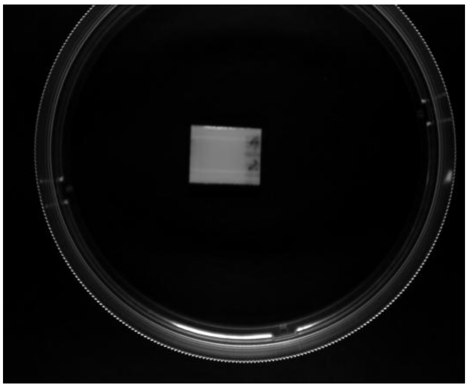
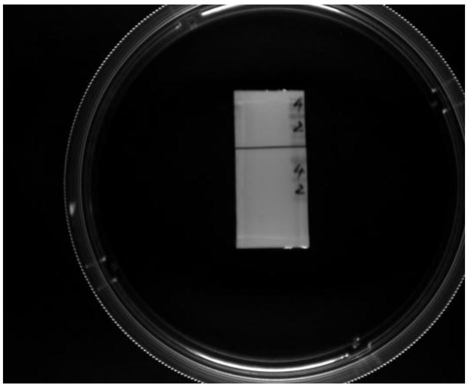

PRDX3 (SE, cisplatin, organelle)

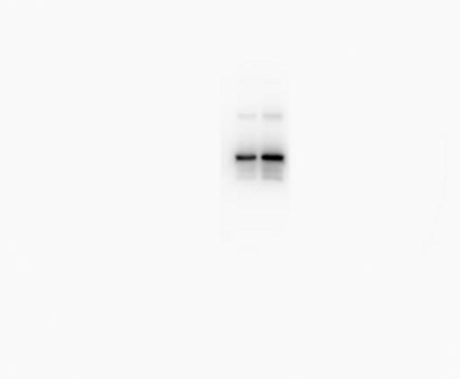
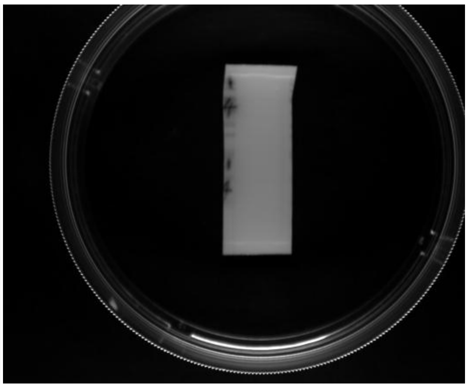

PRDX3 (dimer, LE, cisplatin, organelle)

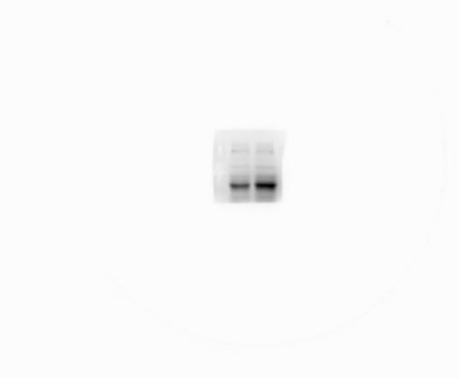
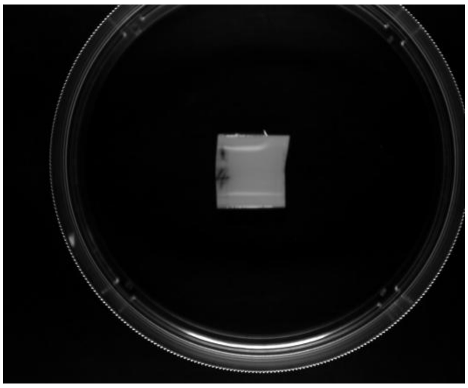

PRDX3 (monomer, SE, cisplatin, organelle)

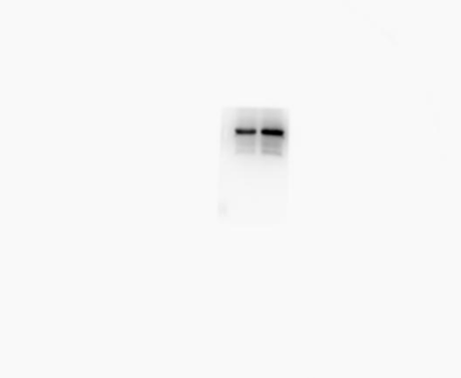

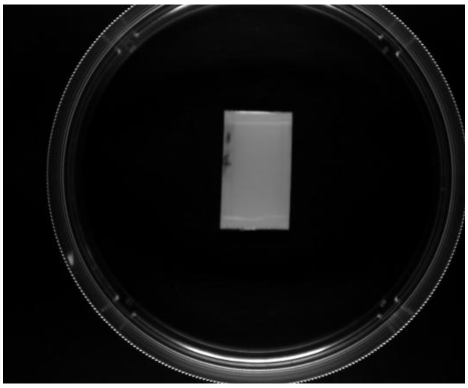

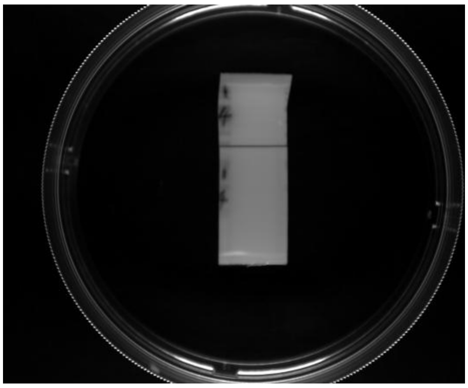

COX IV (cisplatin, organelle)

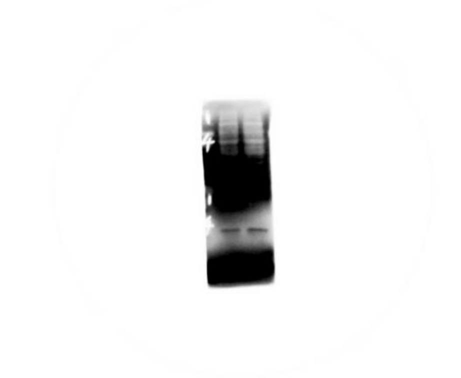

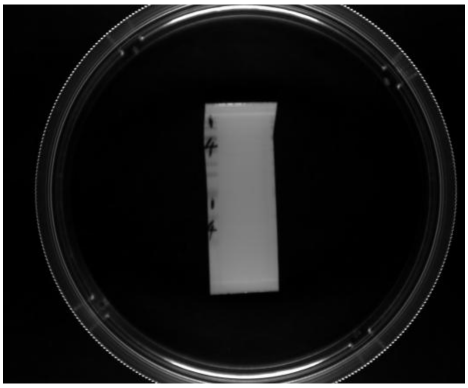

PRDX3 (SE, erastin, total)

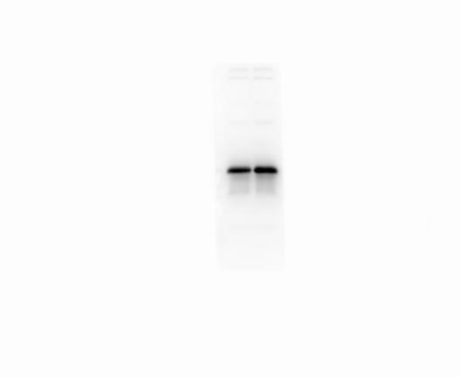

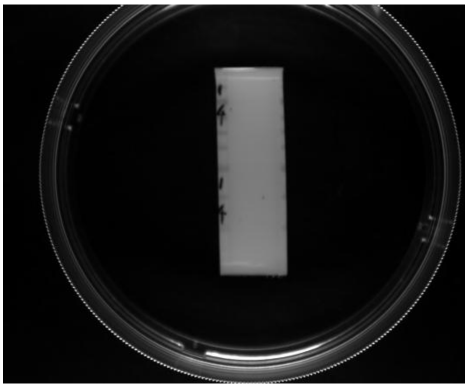

PRDX3 (dimer, LE, erastin, total)

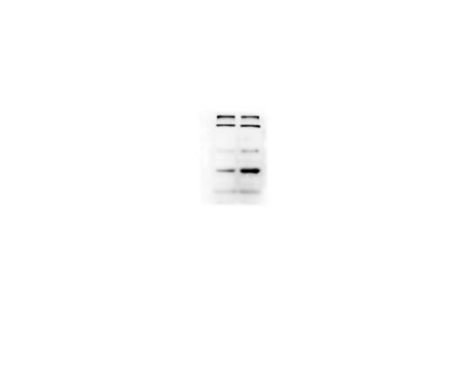

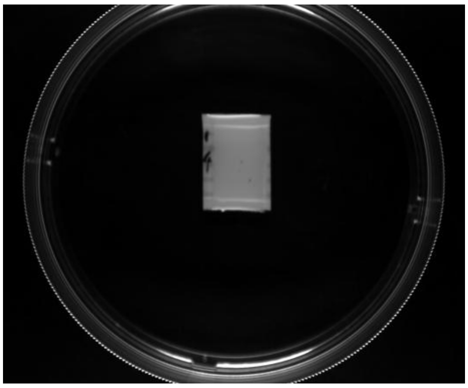

PRDX3 (monomer, SE, erastin, total)

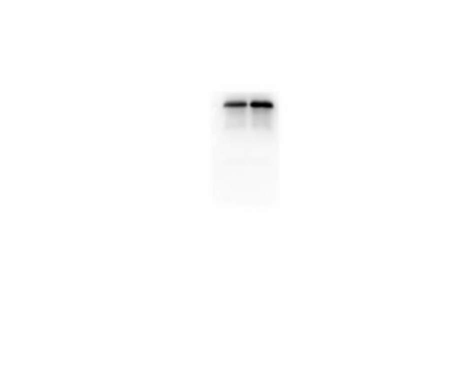

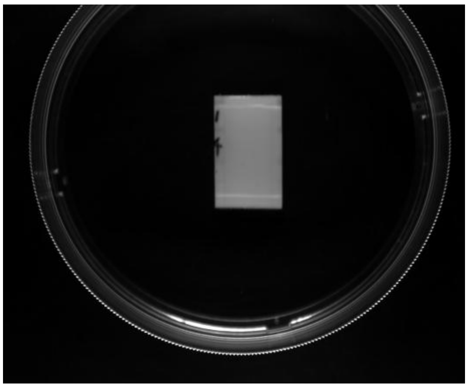

GAPDH (erastin, total)

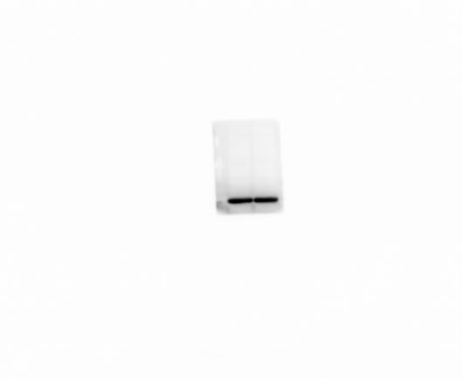

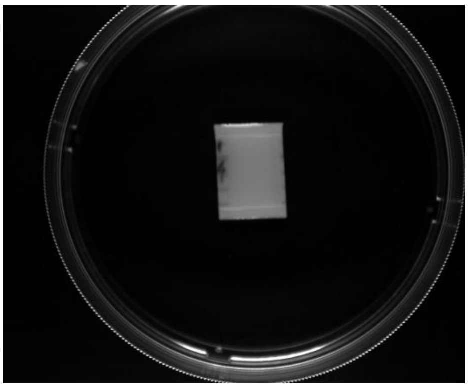

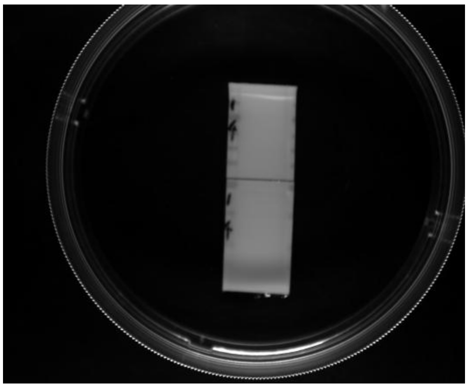

PRDX3 (SE, erastin, cytosol)

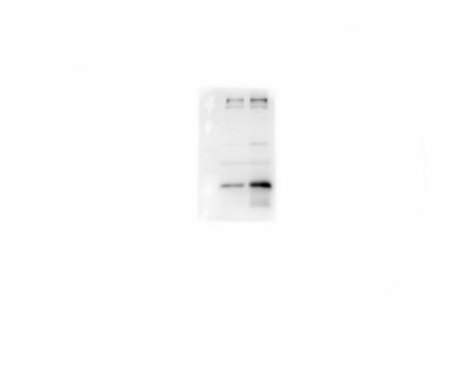

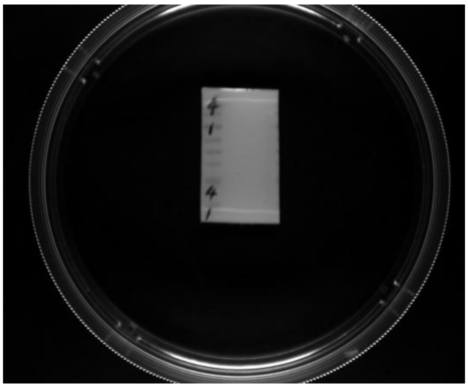

PRDX3 (dimer, LE, erastin, cytosol)

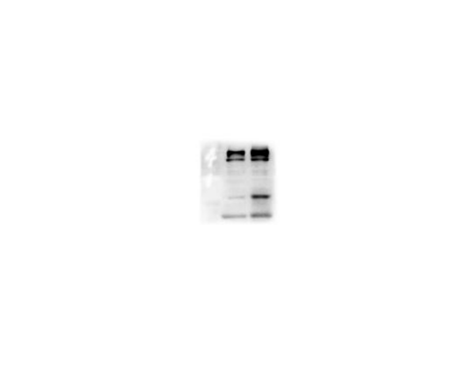

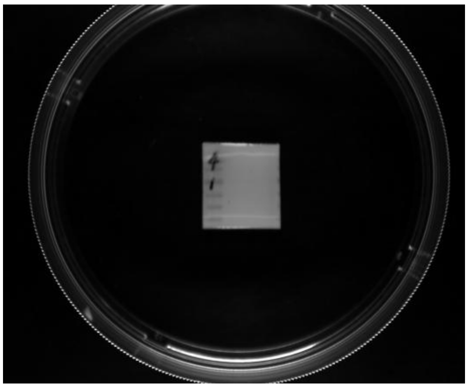

PRDX3 (monomer, SE, erastin, cytosol)

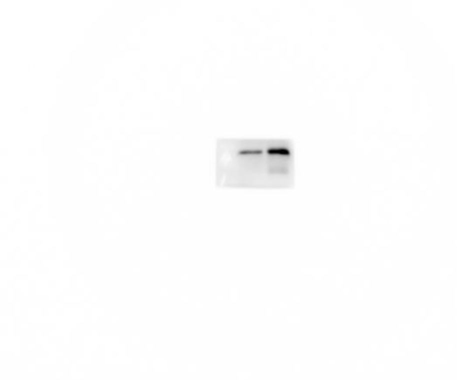

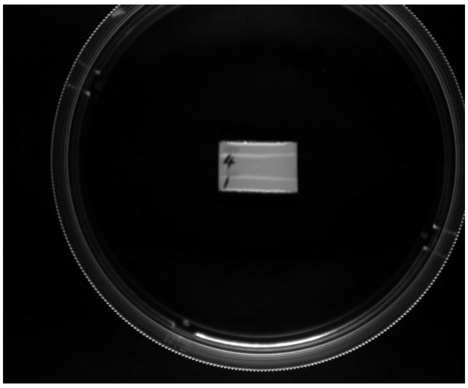

GAPDH (erastin, cytosol)

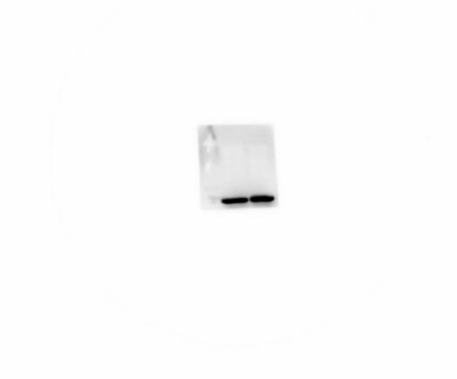

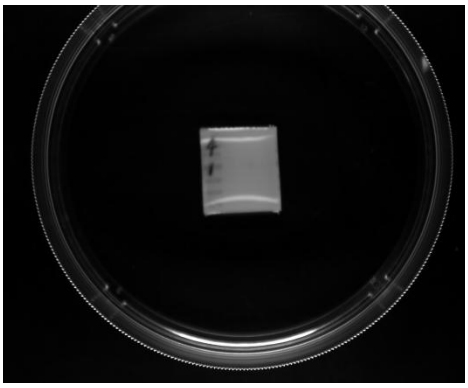

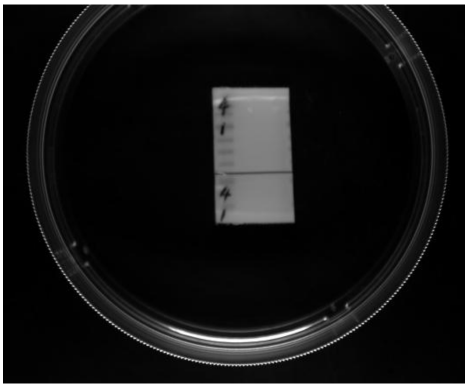

PRDX3 (SE, erastin, plasma membrane)

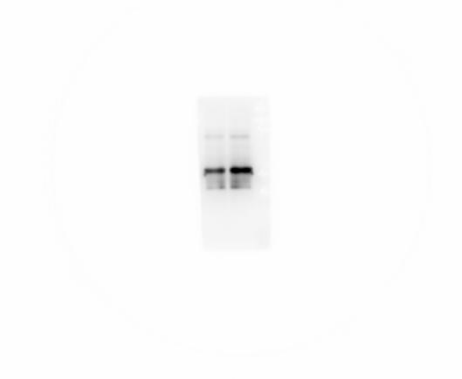
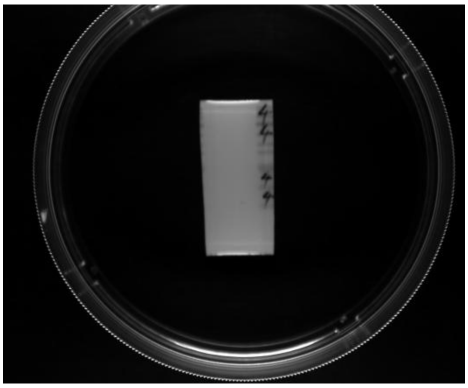

PRDX3 (dimer, LE, erastin, plasma membrane)

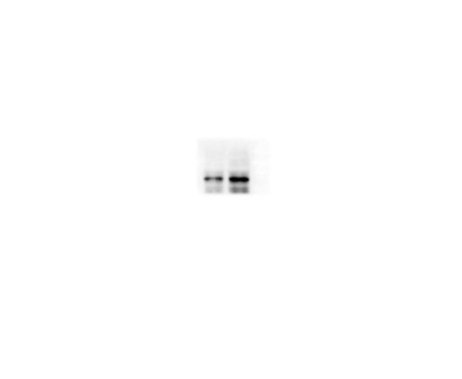
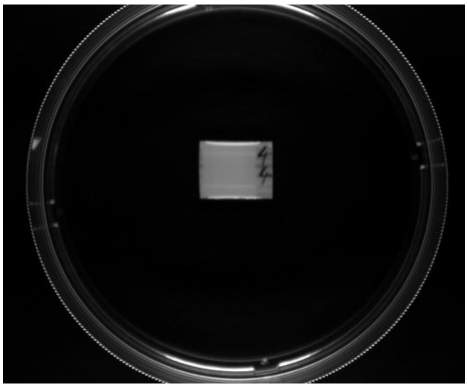

PRDX3 (monomer, SE, erastin, plasma membrane)

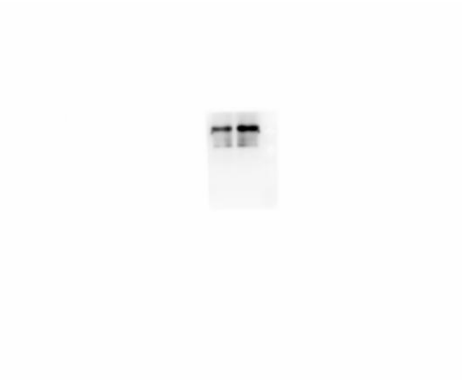
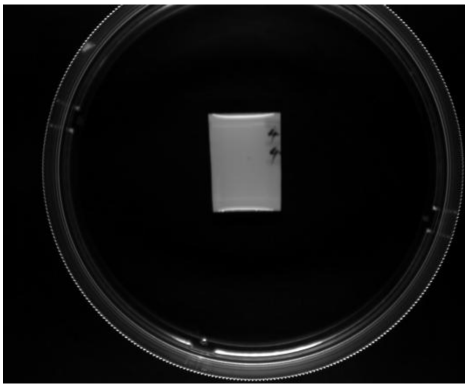

Na$^+$/K$^+$ ATPase a1 (erastin, plasma membrane)

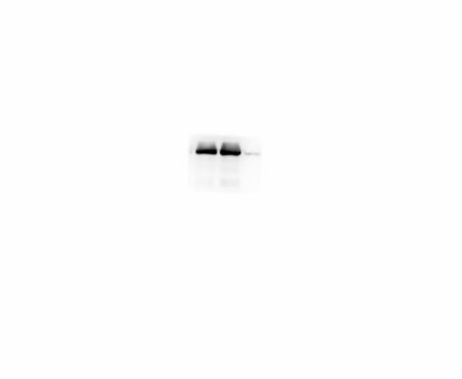

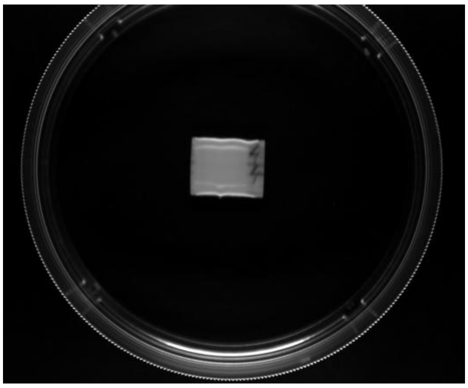

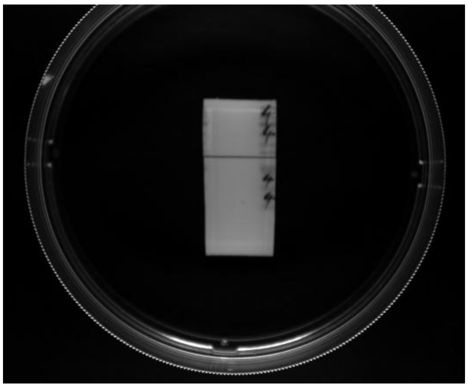

PRDX3 (SE, erastin, organelle)

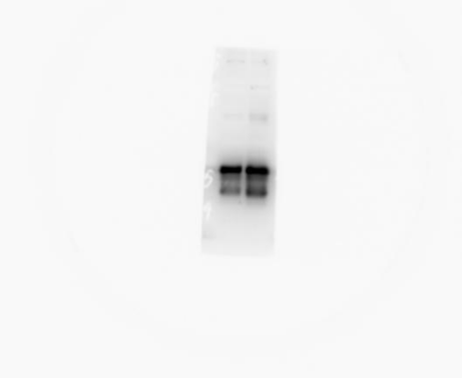

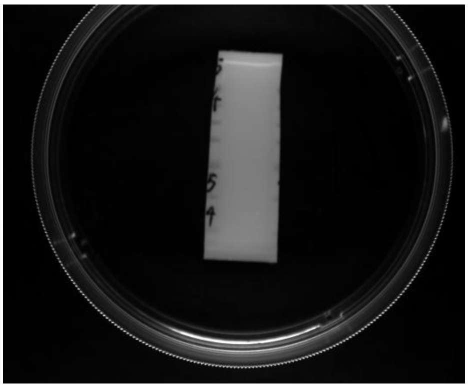

PRDX3 (dimer, LE, erastin, organelle)

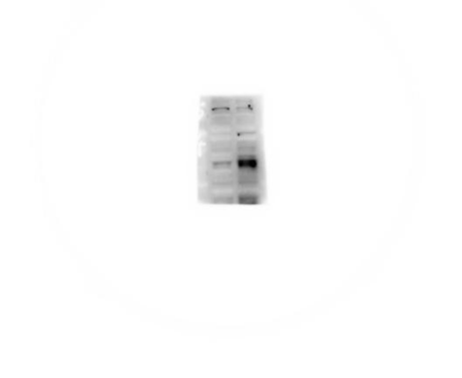

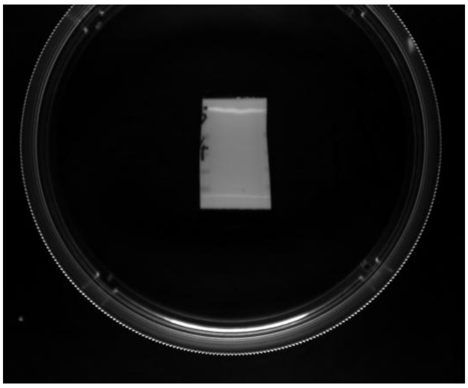

PRDX3 (monomer, SE, erastin, organelle)

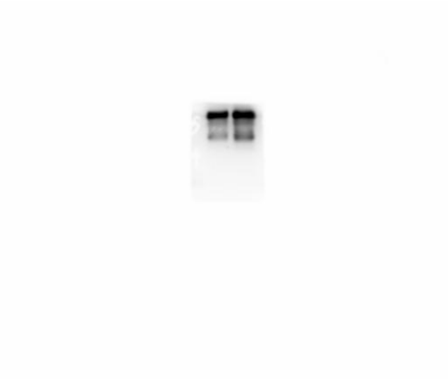

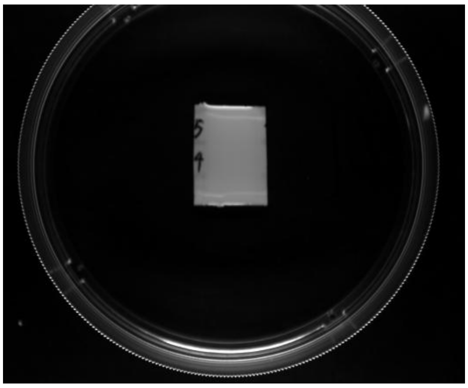

COX IV (erastin, organelle)

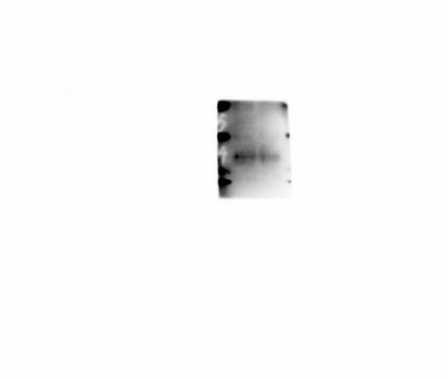

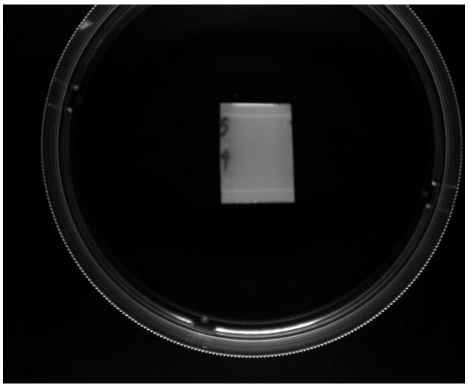

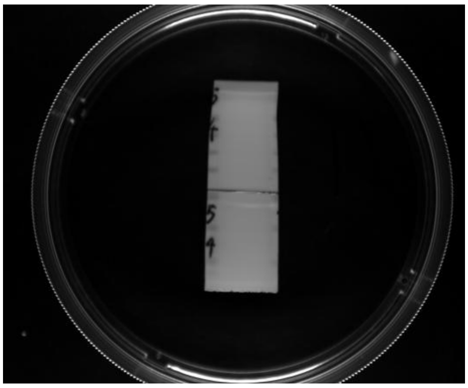

Fig. 7J Second Repetition

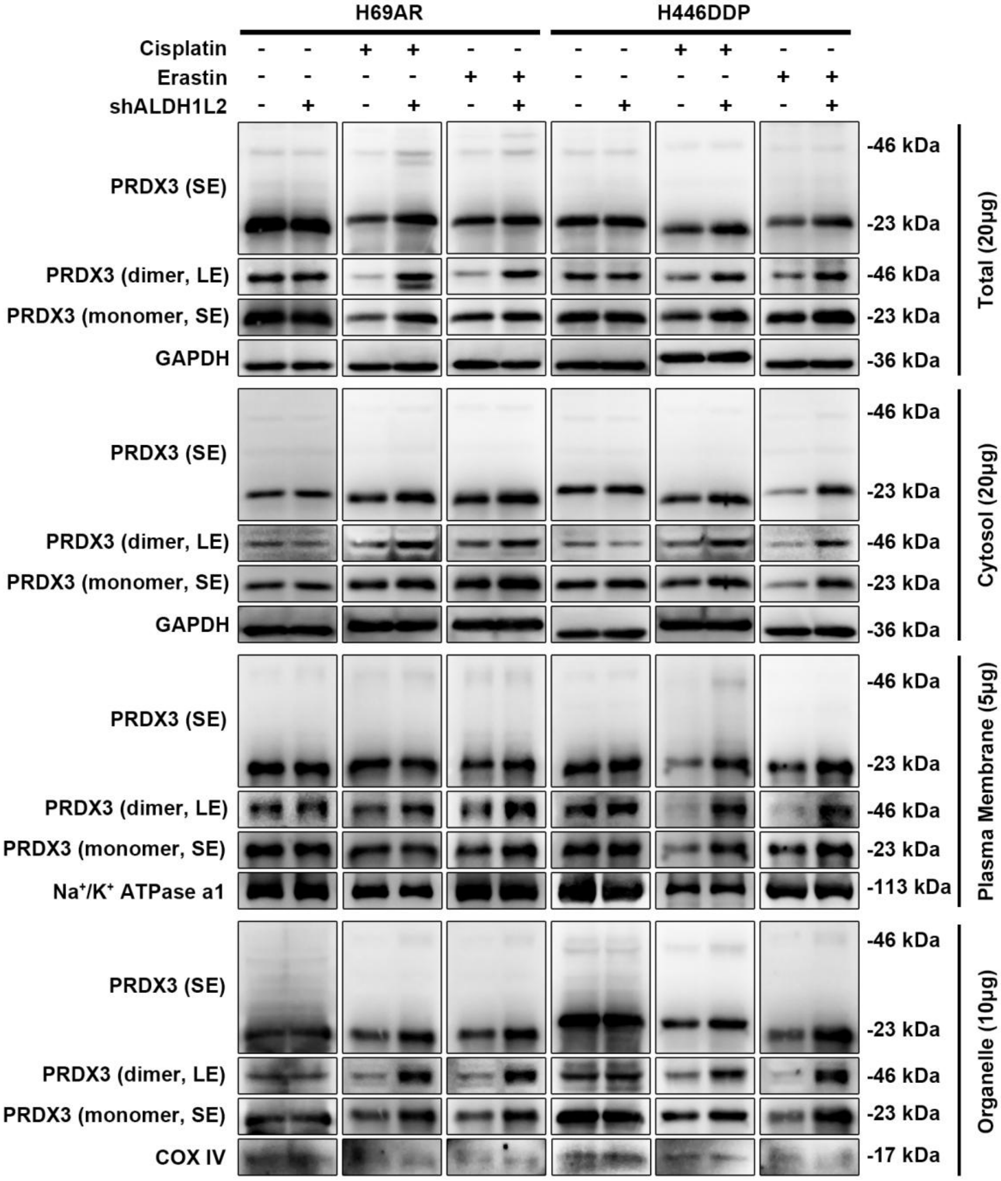

H69AR

PRDX3 (SE, untreated, total)

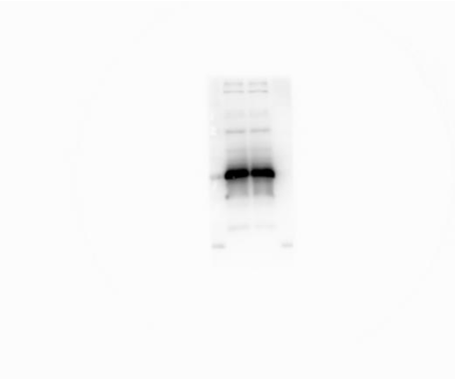

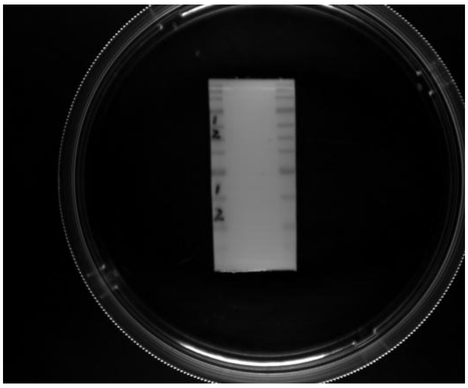

PRDX3 (dimer, LE, untreated, total)

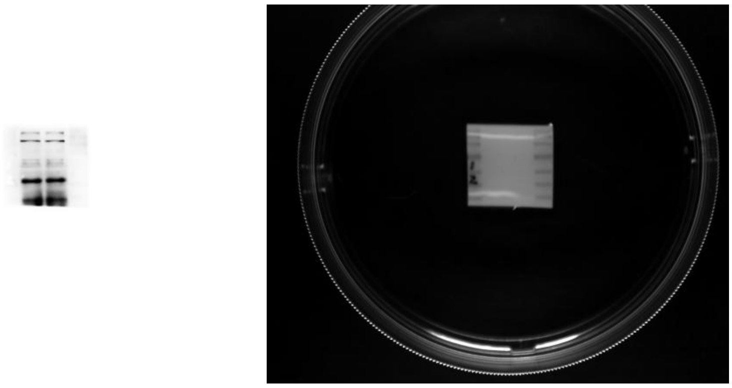

PRDX3 (monomer, SE, untreated, total)

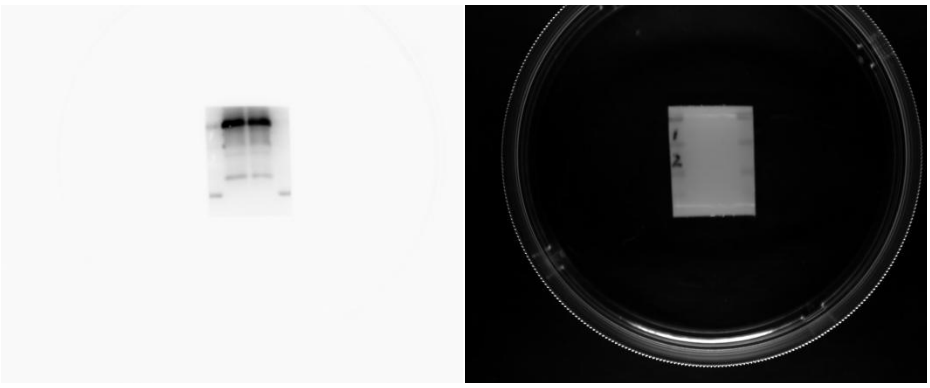

GAPDH (untreated, total)

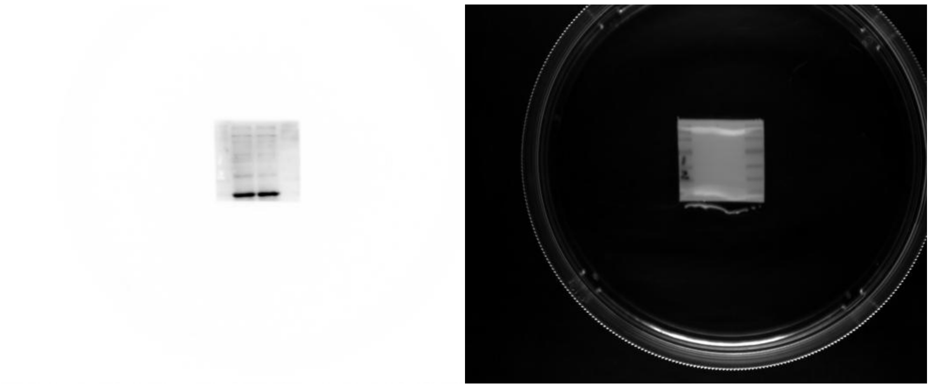

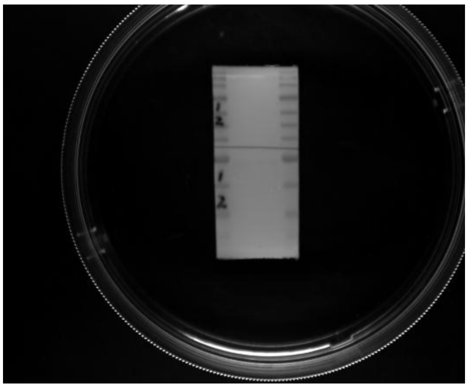

PRDX3 (SE, untreated, cytosol)

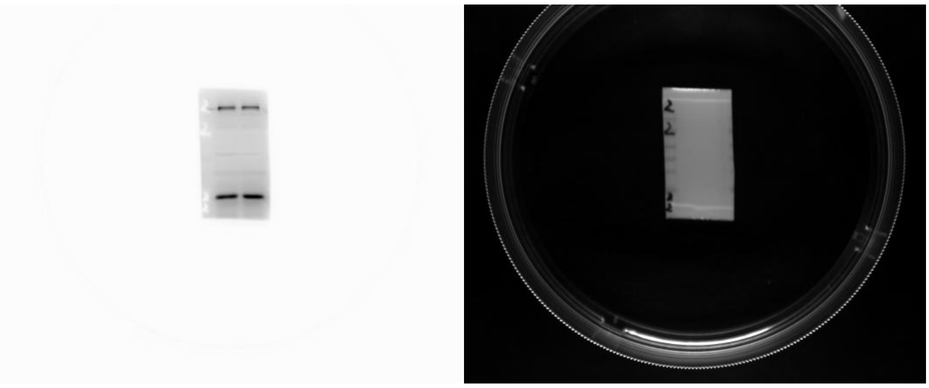

PRDX3 (dimer, LE, untreated, cytosol)

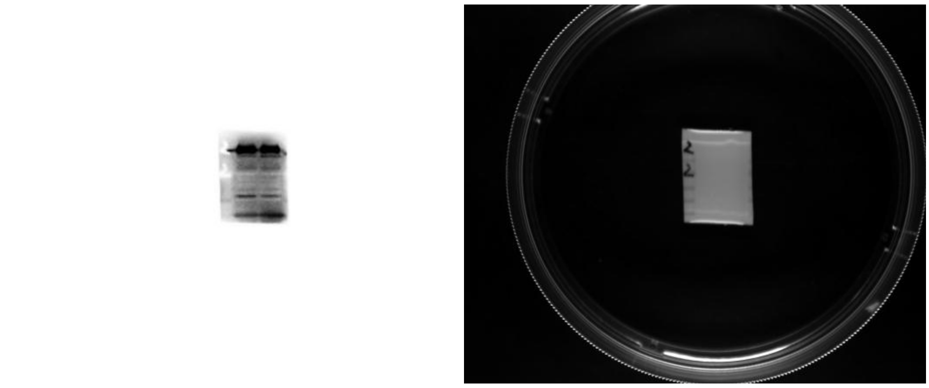

PRDX3 (monomer, SE, untreated, cytosol)

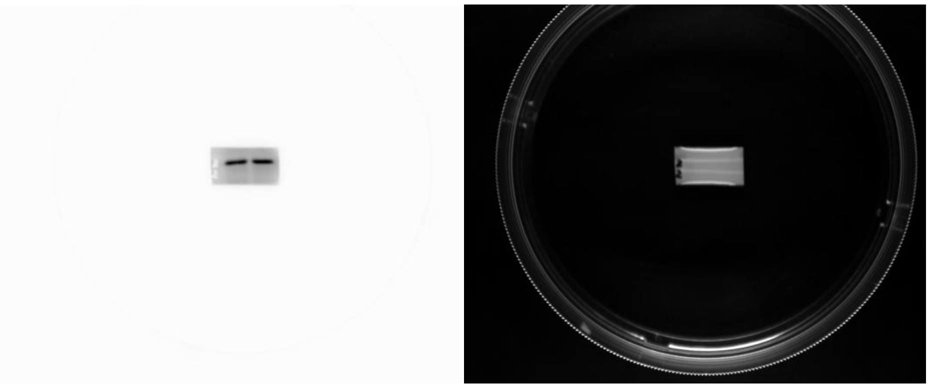

GAPDH (untreated, cytosol)

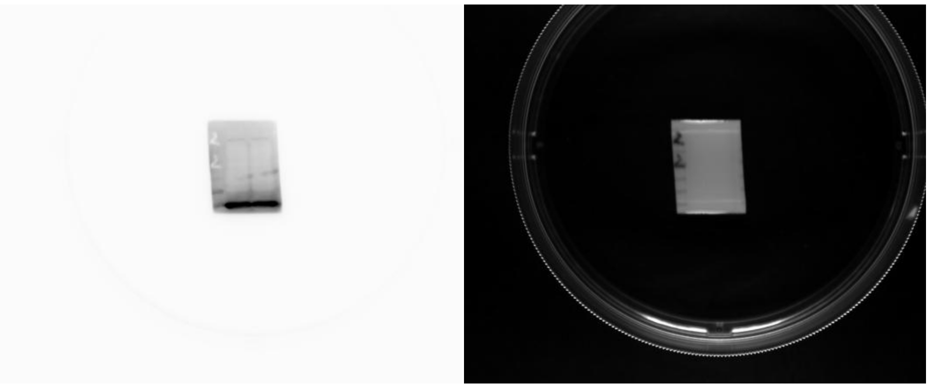

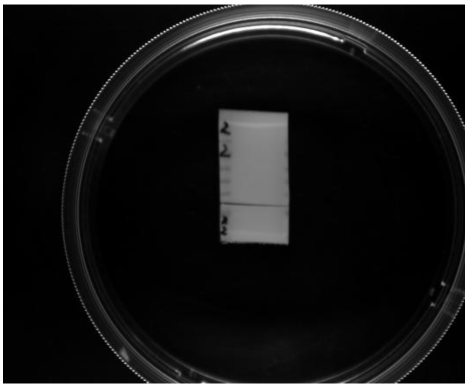

PRDX3 (SE, untreated, plasma membrane)

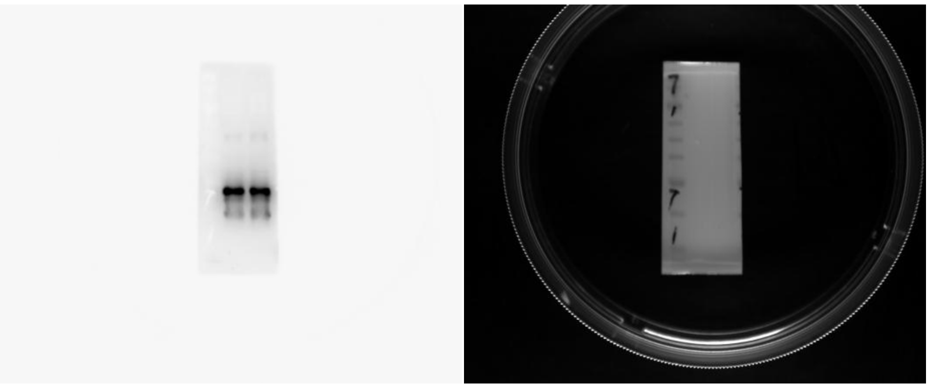

PRDX3 (dimer, LE, untreated, plasma membrane)

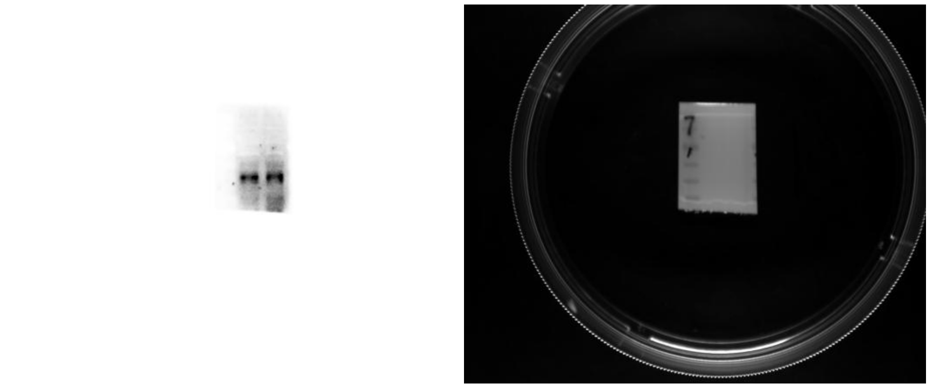

PRDX3 (monomer, SE, untreated, plasma membrane)

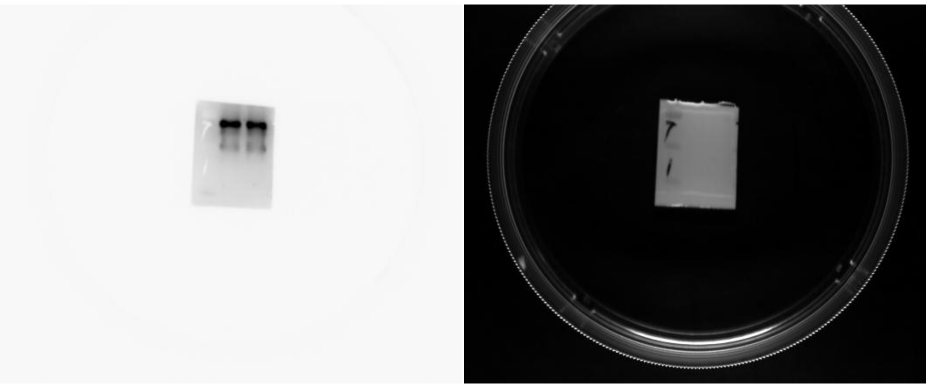

Na$^+$/K$^+$ ATPase a1 (untreated, plasma membrane)

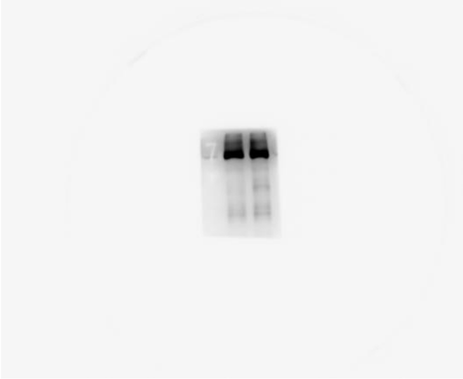

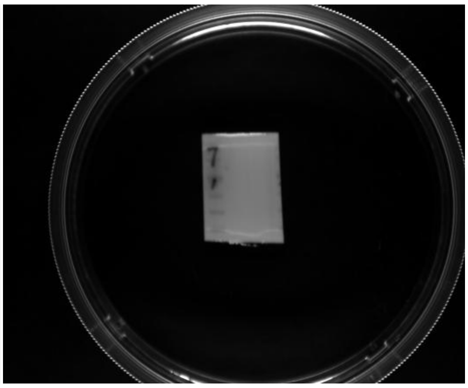

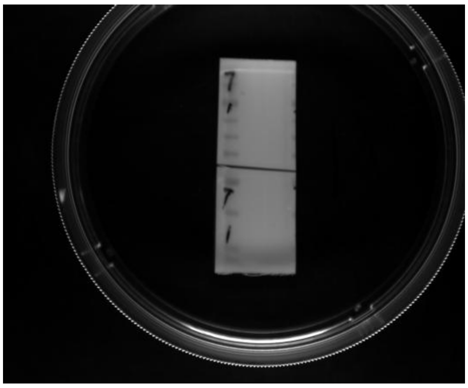

PRDX3 (SE, untreated, organelle)

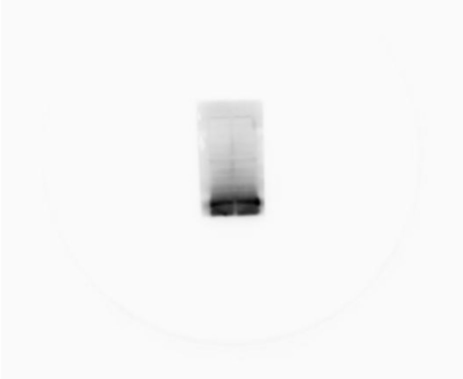

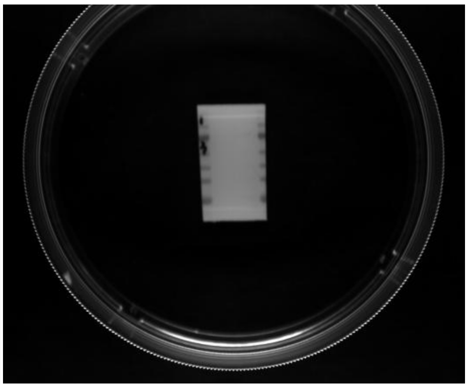

PRDX3 (dimer, LE, untreated, organelle)

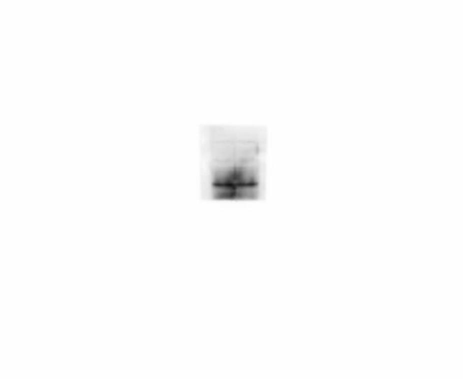

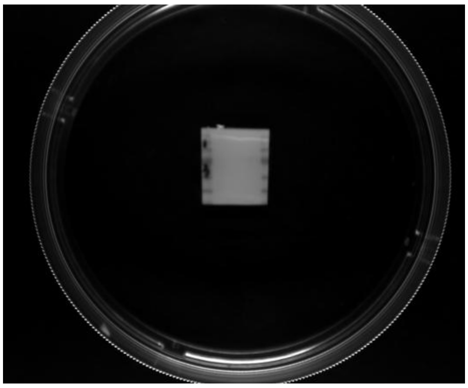

PRDX3 (monomer, SE, untreated, organelle)

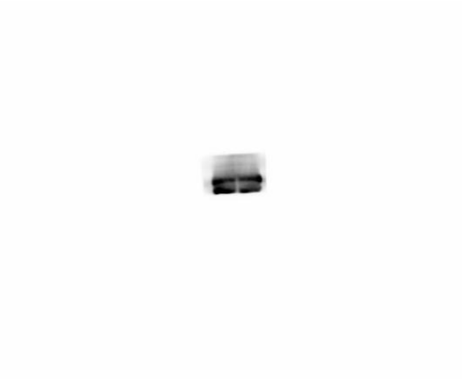

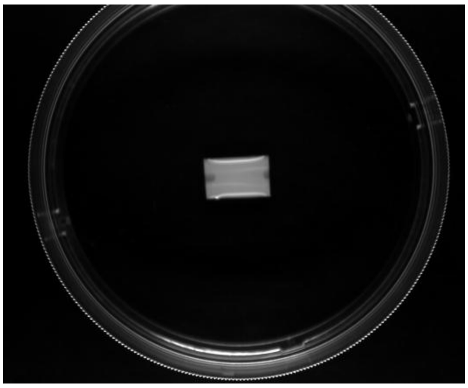

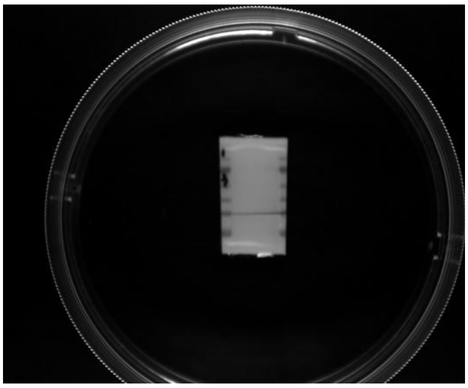

COX IV (untreated, organelle)

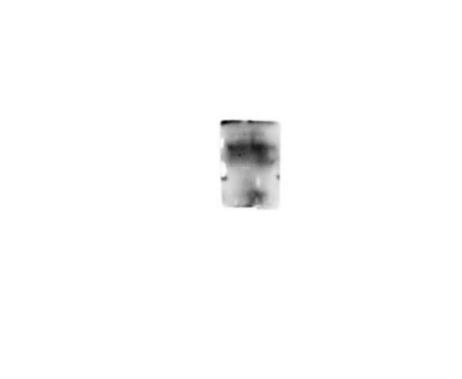

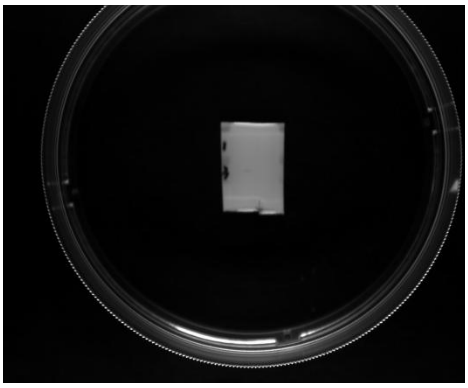

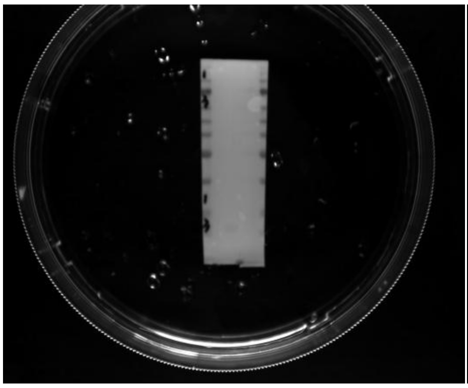

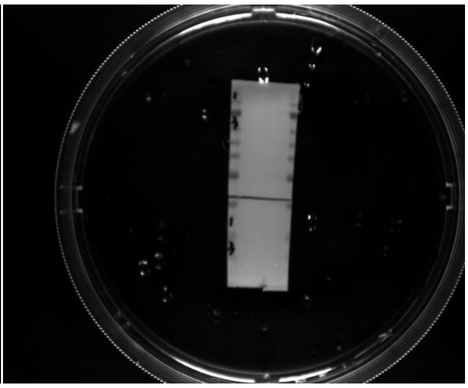

PRDX3 (SE, cisplatin, total)

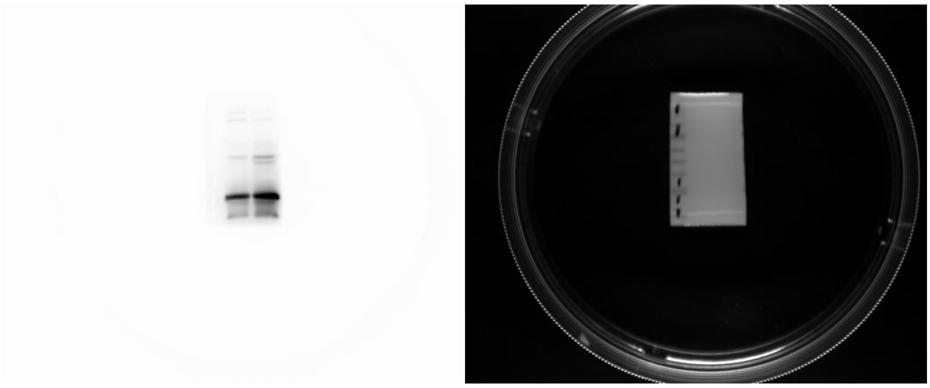

PRDX3 (dimer, LE, cisplatin, total)

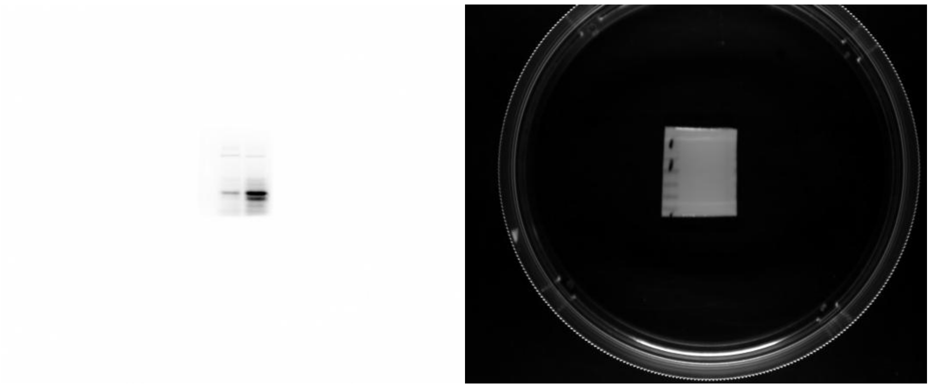

PRDX3 (monomer, SE, cisplatin, total)

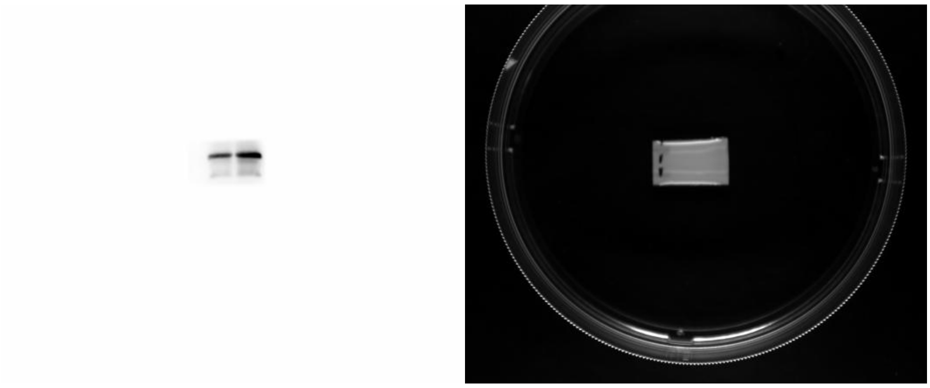

GAPDH (cisplatin, total)

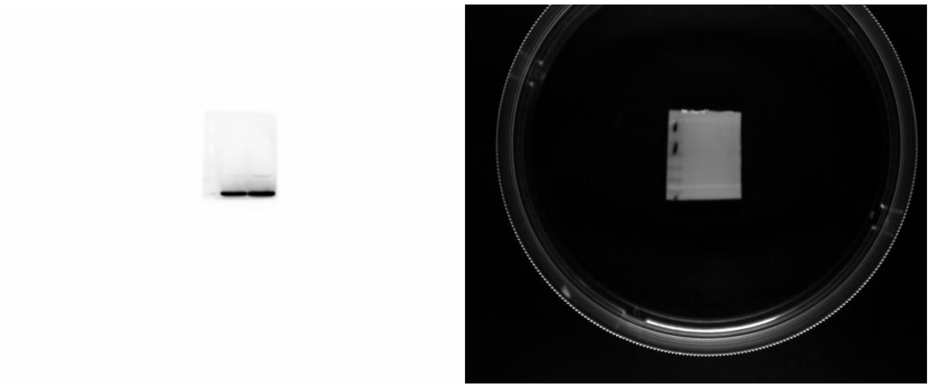

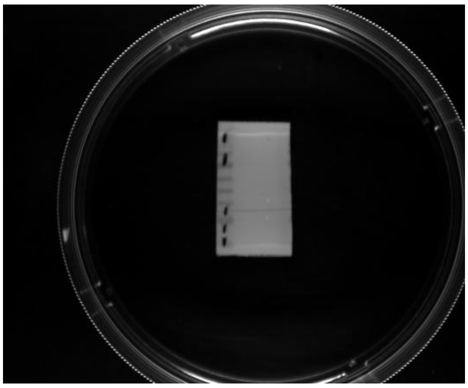

PRDX3 (SE, cisplatin, cytosol)

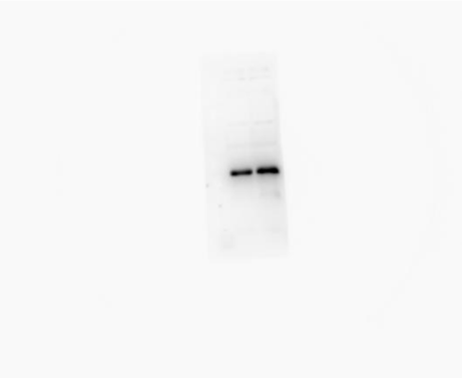

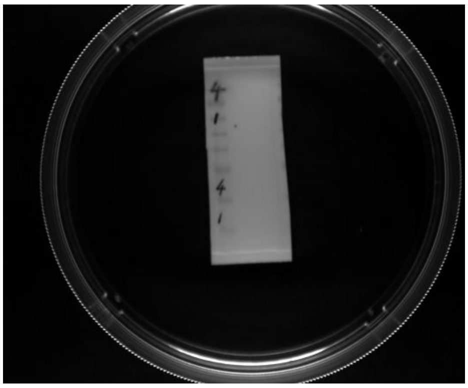

PRDX3 (dimer, LE, cisplatin, cytosol)

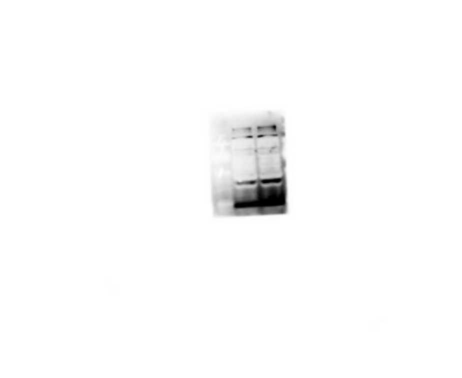

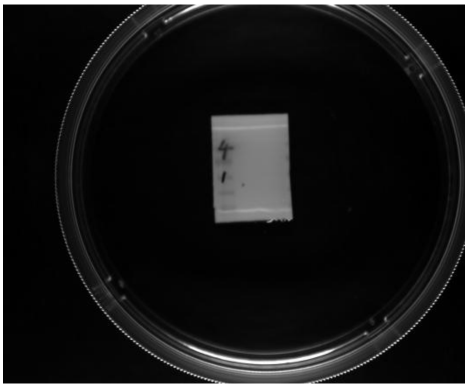

PRDX3 (monomer, SE, cisplatin, cytosol)

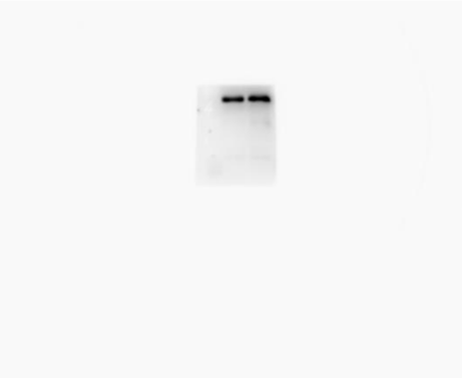

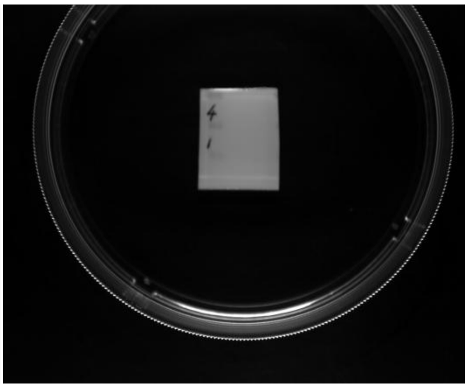

GAPDH (cisplatin, cytosol)

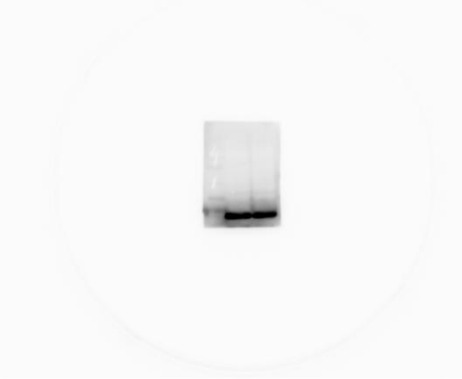
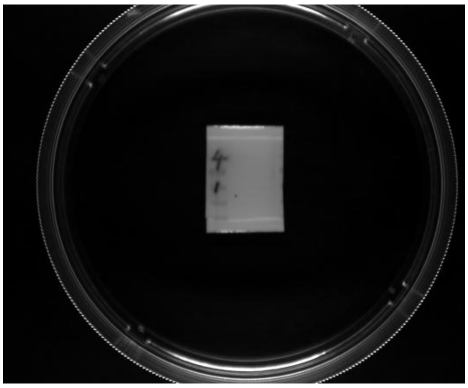

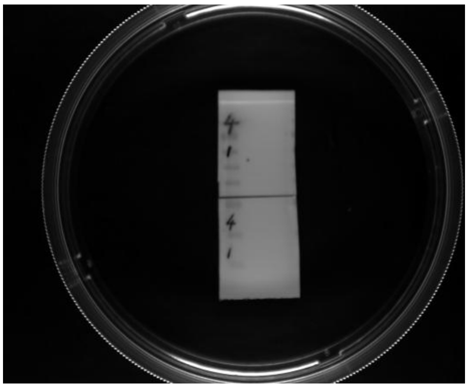

PRDX3 (SE, cisplatin, plasma membrane)

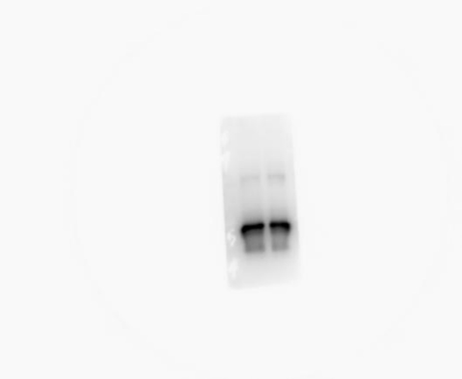
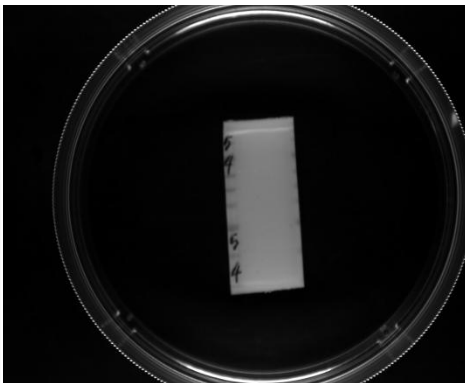

PRDX3 (dimer, LE, cisplatin, plasma membrane)

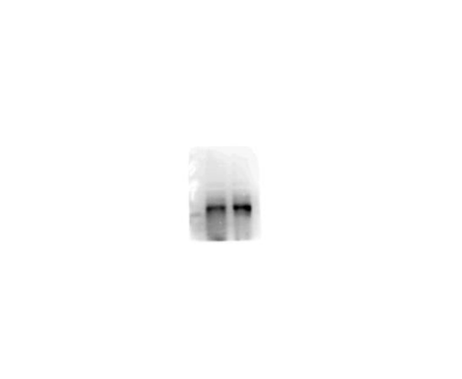
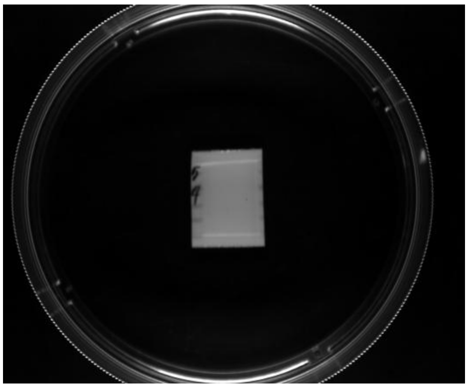

PRDX3 (monomer, SE, cisplatin, plasma membrane)

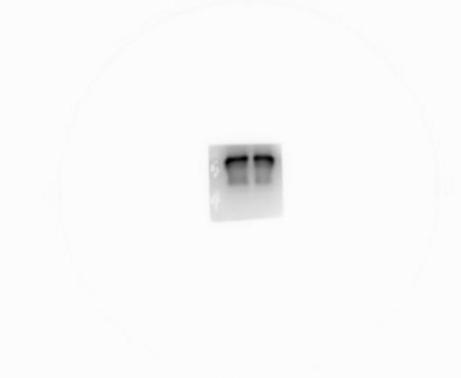 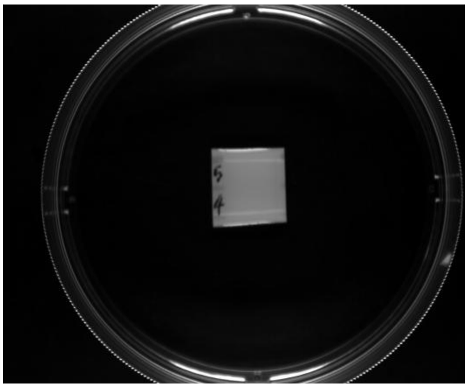

Na^+/K^+ ATPase a1 (cisplatin, plasma membrane)

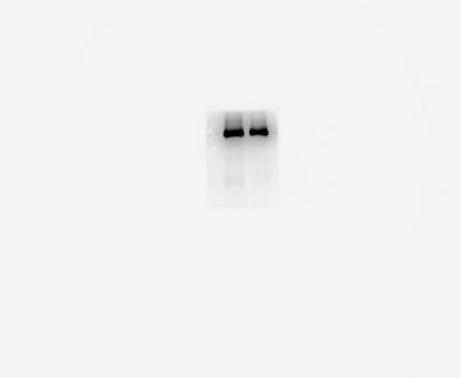 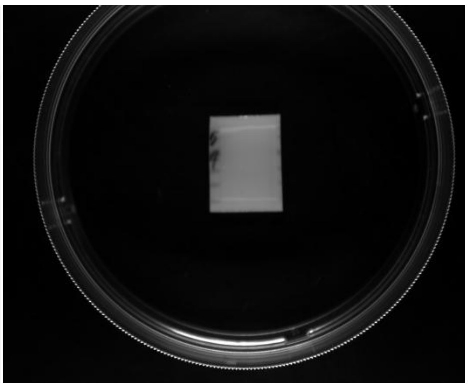

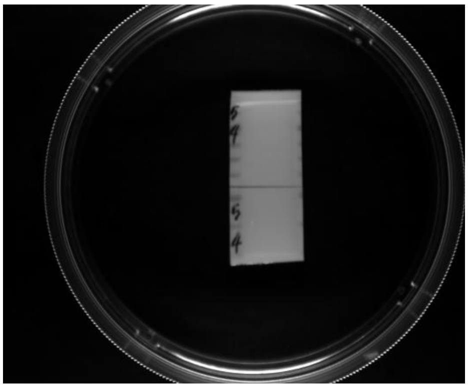

PRDX3 (SE, cisplatin, organelle)

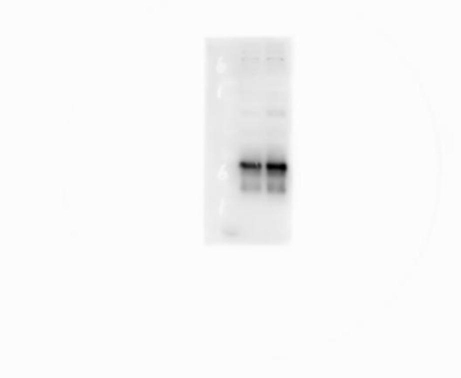 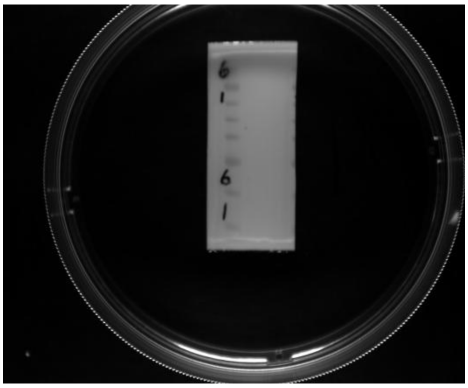

PRDX3 (dimer, LE, cisplatin, organelle)

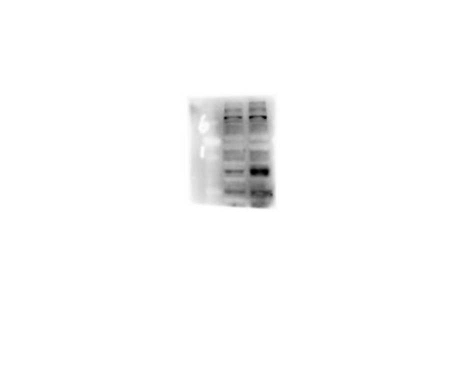

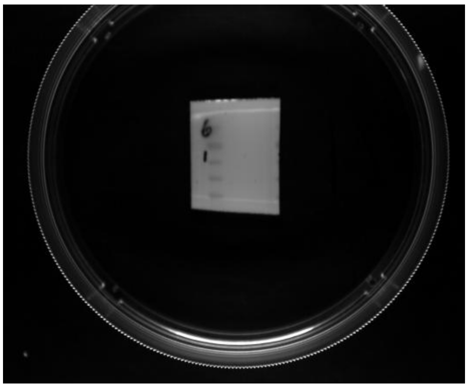

PRDX3 (monomer, SE, cisplatin, organelle)

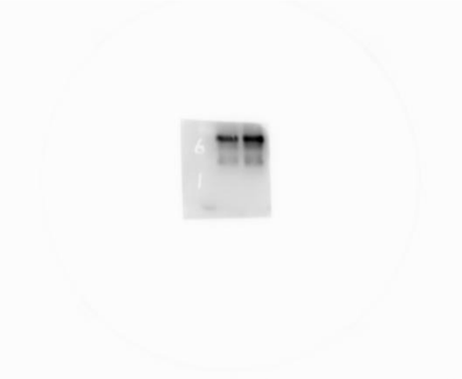

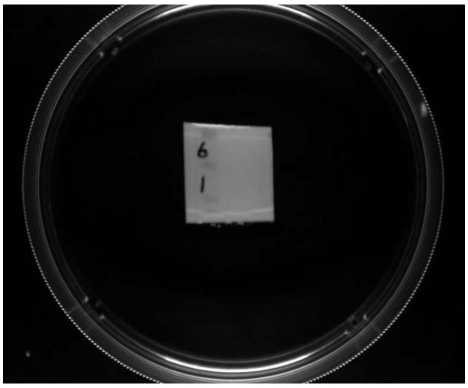

COX IV (cisplatin, organelle)

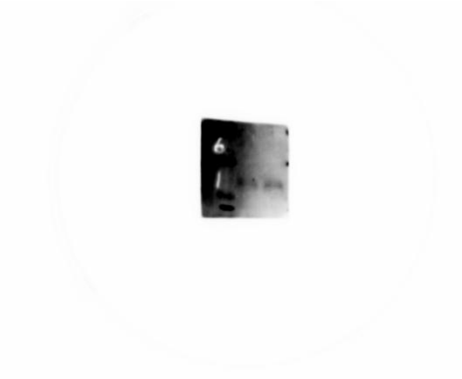

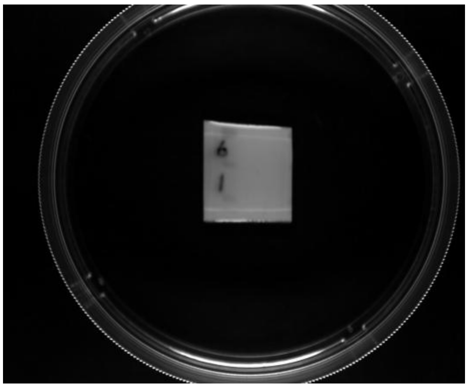

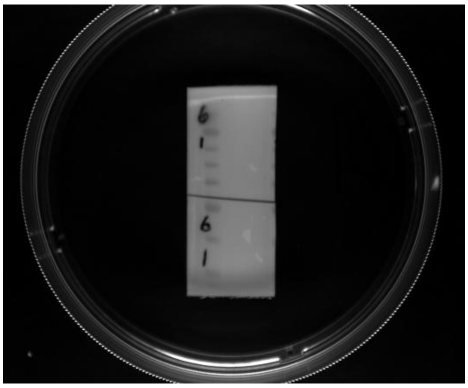

PRDX3 (SE, erastin, total)

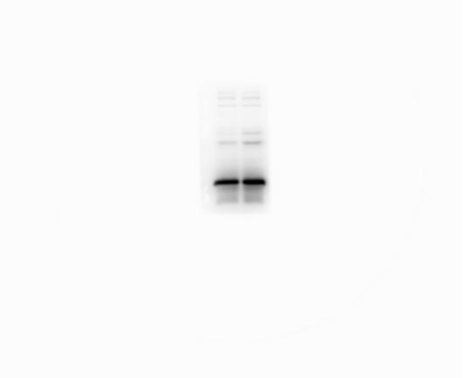

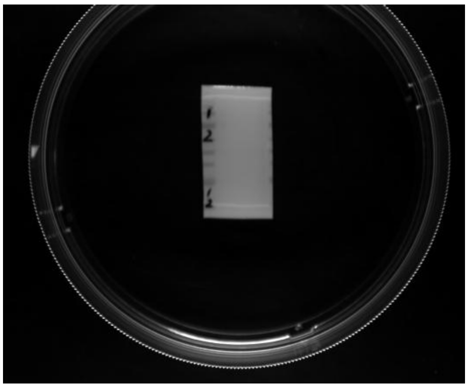

PRDX3 (dimer, LE, erastin, total)

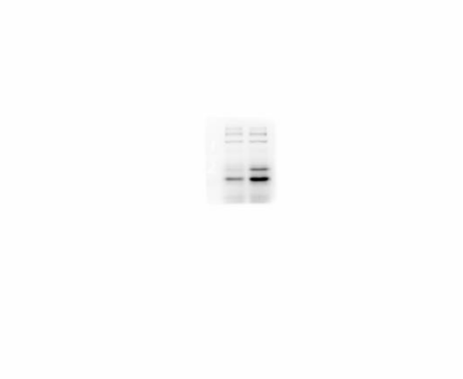

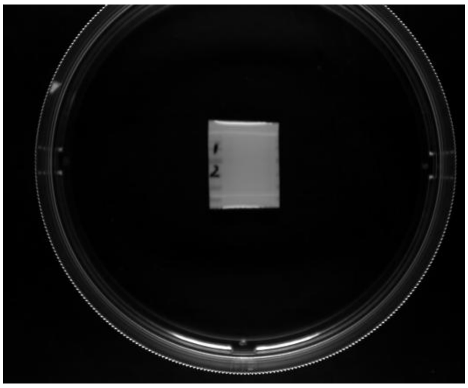

PRDX3 (monomer, SE, erastin, total)

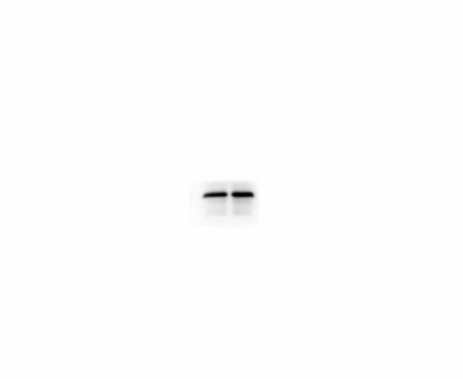

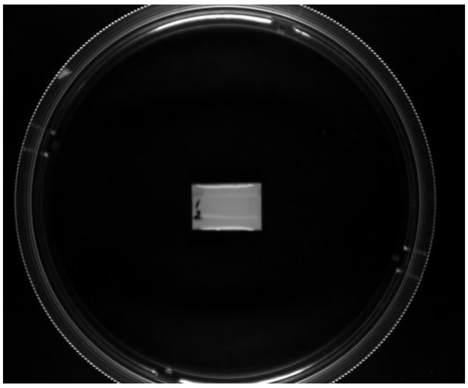

GAPDH (erastin, total)

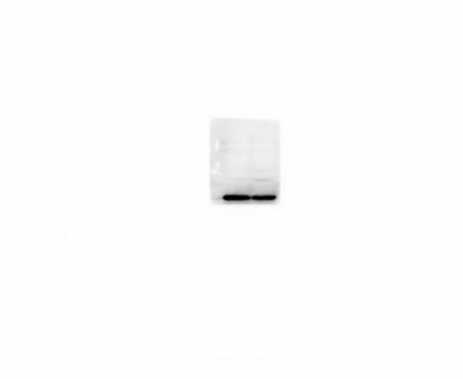

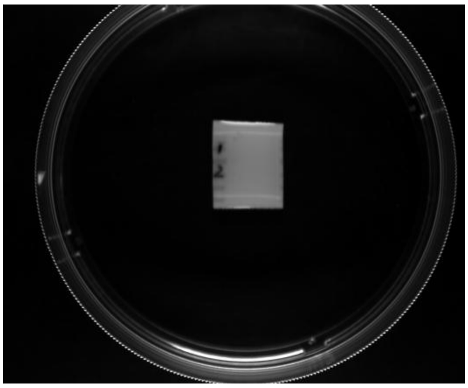

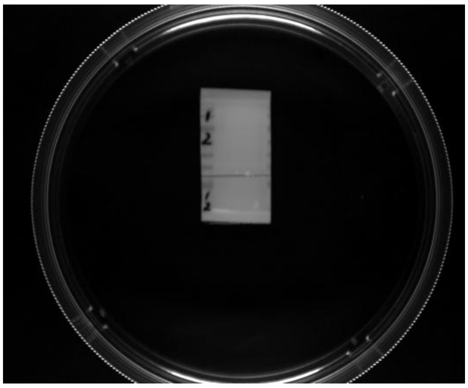

PRDX3 (SE, erastin, cytosol)

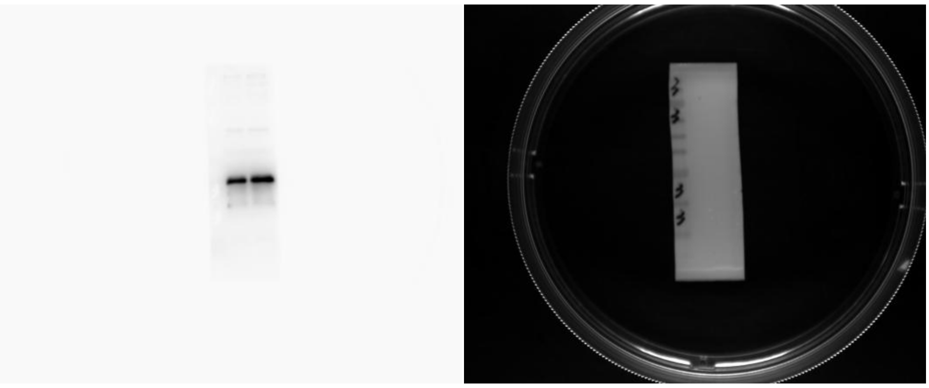

PRDX3 (dimer, LE, erastin, cytosol)

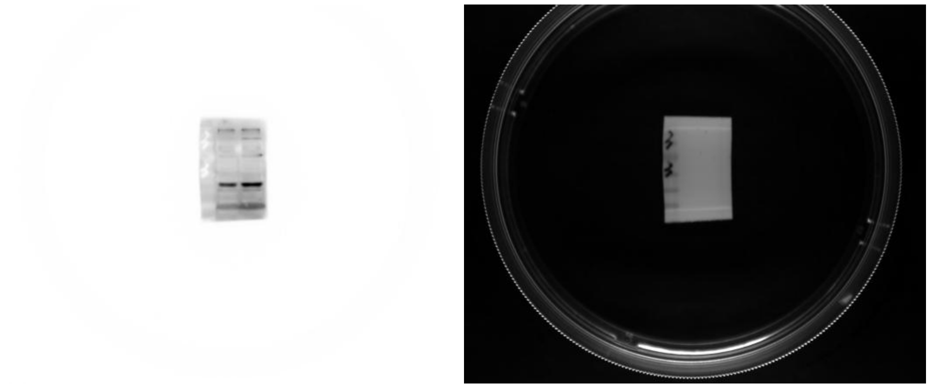

PRDX3 (monomer, SE, erastin, cytosol)

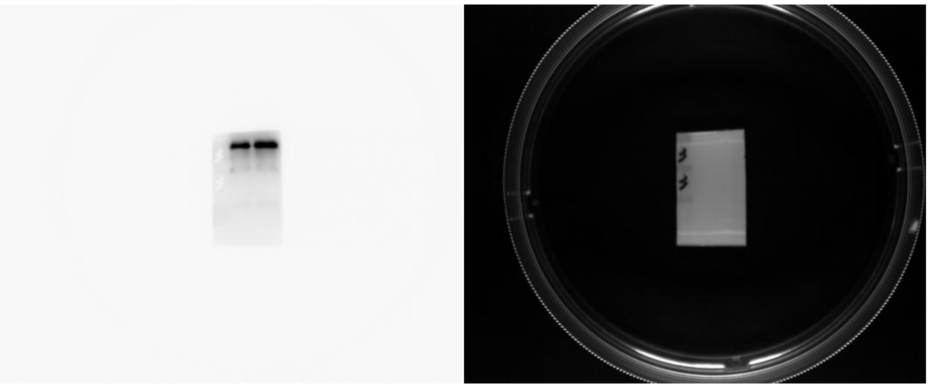

GAPDH (erastin, cytosol)

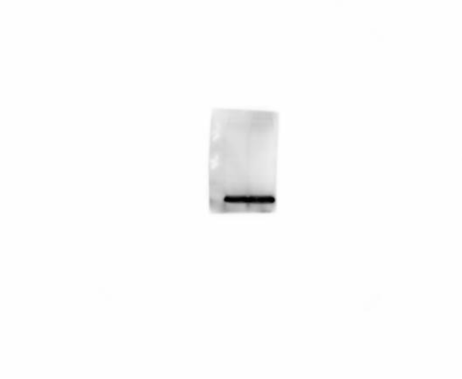

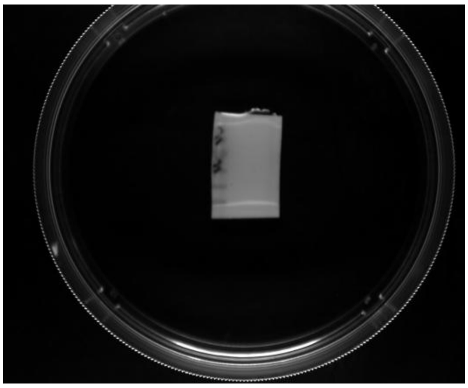

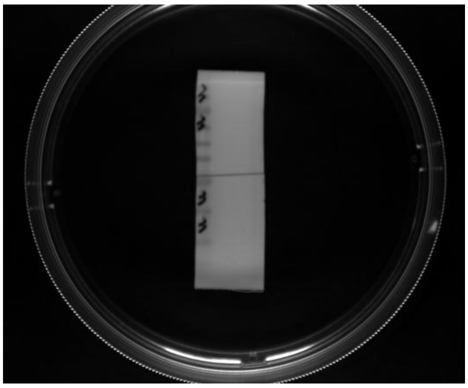

PRDX3 (SE, erastin, plasma membrane)

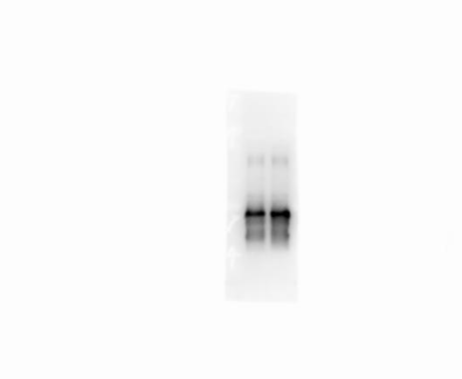

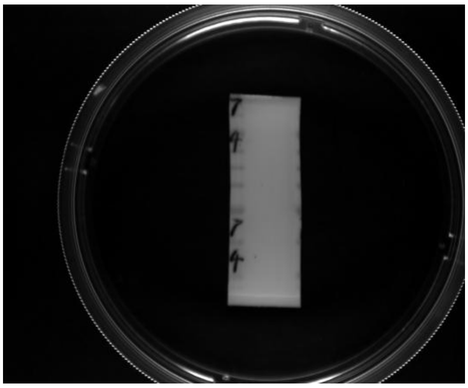

PRDX3 (dimer, LE, erastin, plasma membrane)

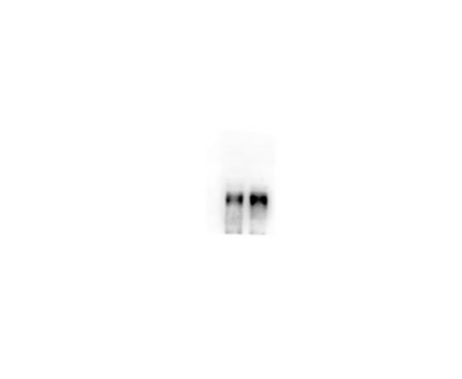

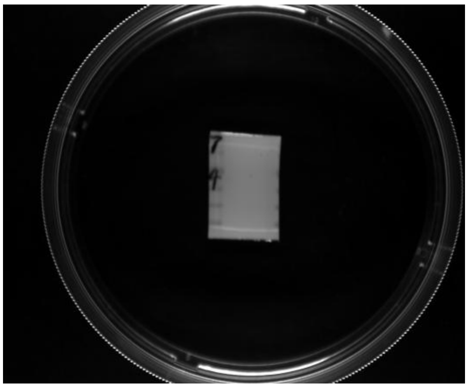

PRDX3 (monomer, SE, erastin, plasma membrane)

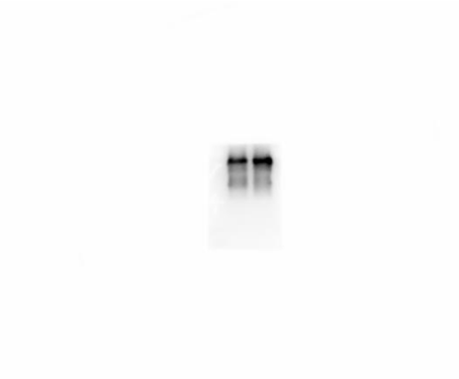

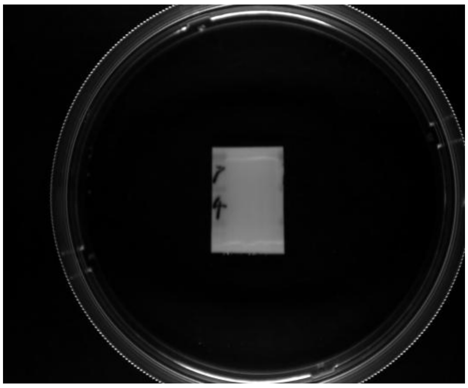

Na^{+}/K^{+} ATPase a1 (erastin, plasma membrane)

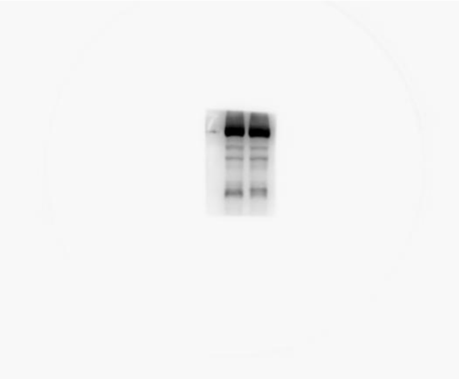

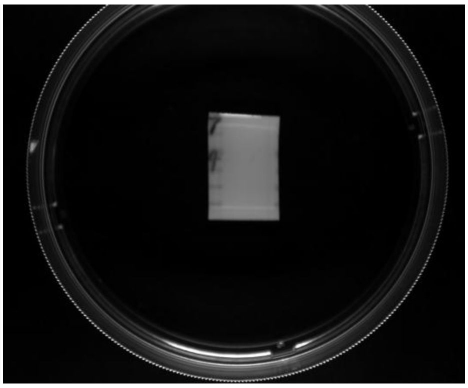

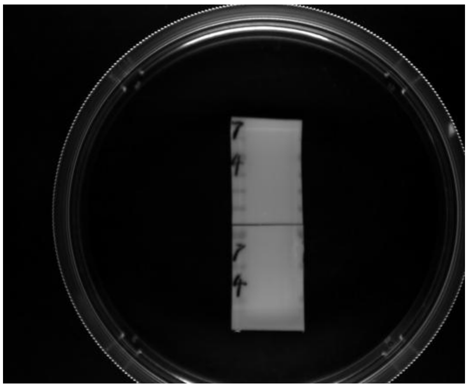

PRDX3 (SE, erastin, organelle)

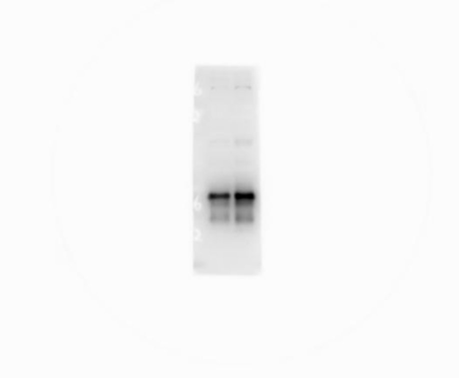

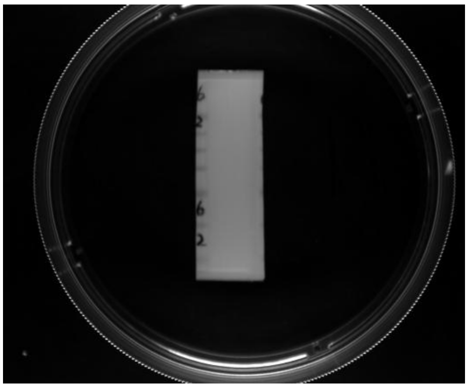

PRDX3 (dimer, LE, erastin, organelle)

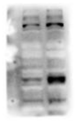

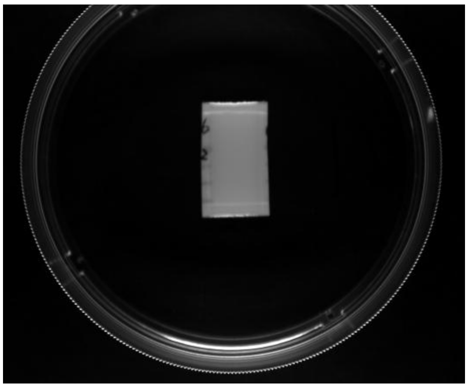

PRDX3 (monomer, SE, erastin, organelle)

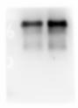

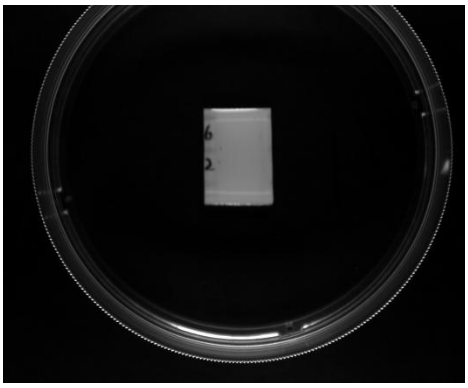

COX IV (erastin, organelle)

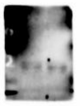

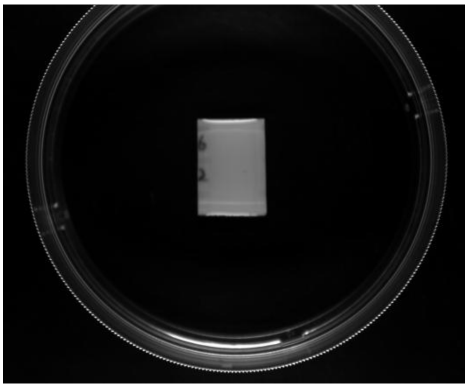

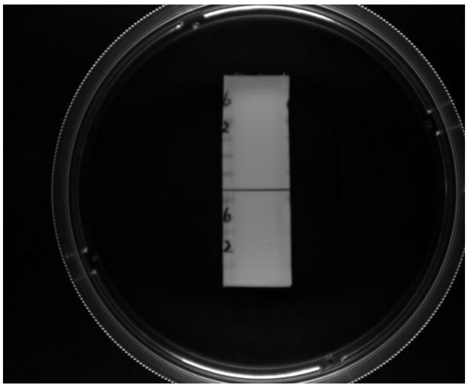

H446DDP

PRDX3 (SE, untreated, total)

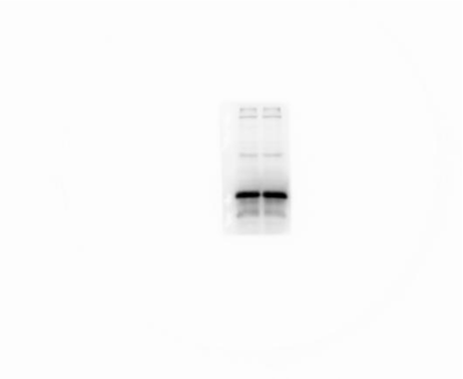
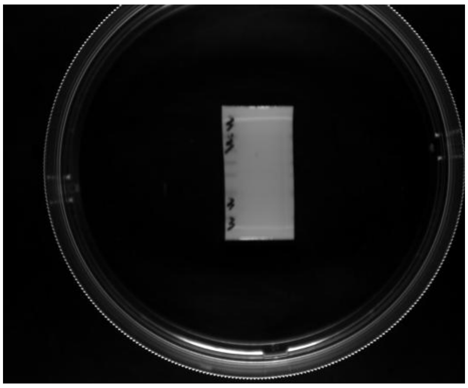

PRDX3 (dimer, LE, untreated, total)

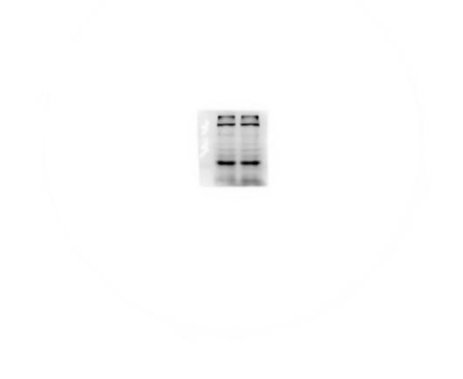
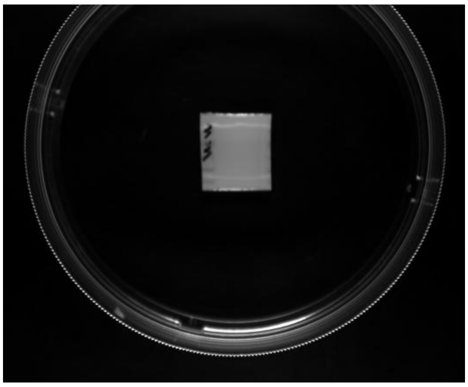

PRDX3 (monomer, SE, untreated, total)

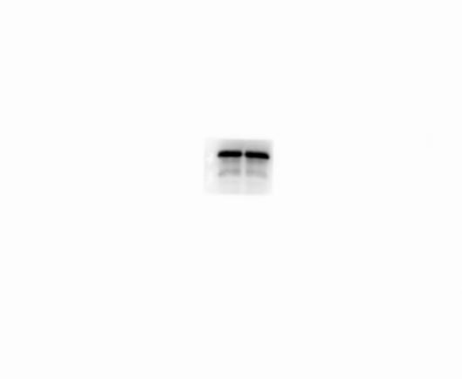
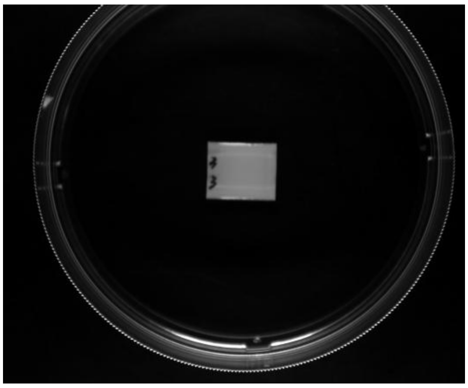

GAPDH (untreated, total)

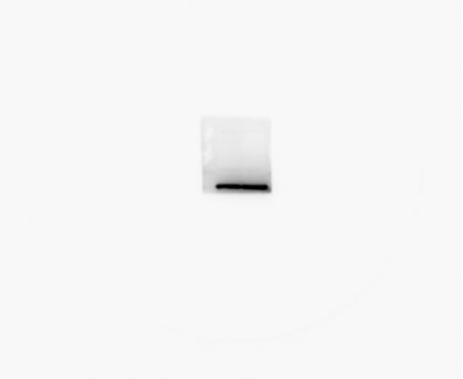
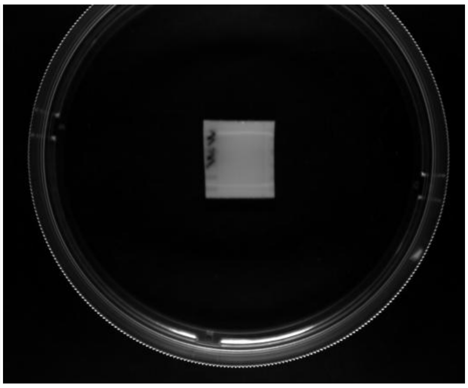

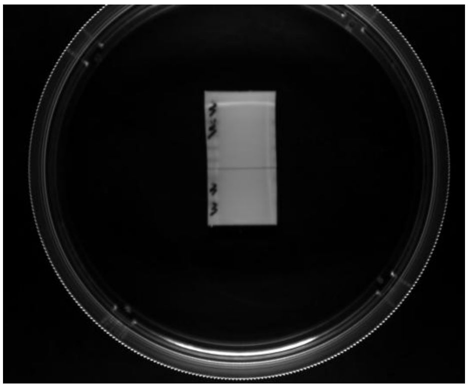

PRDX3 (SE, untreated, cytosol)

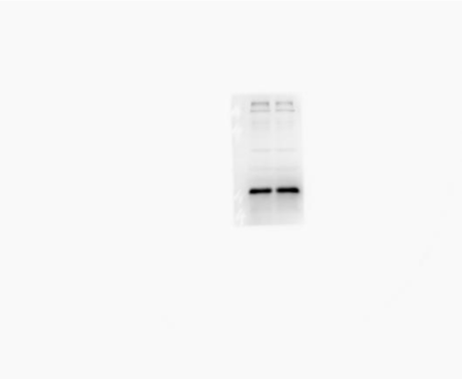

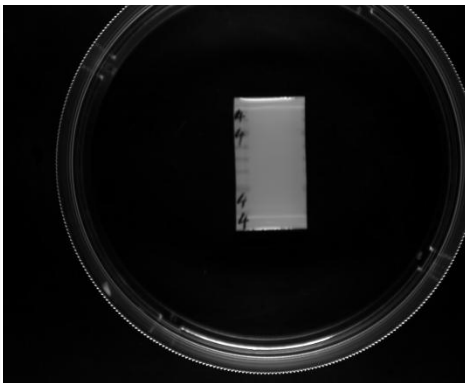

PRDX3 (dimer, LE, untreated, cytosol)

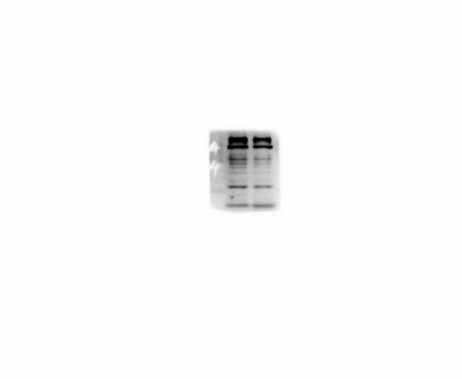

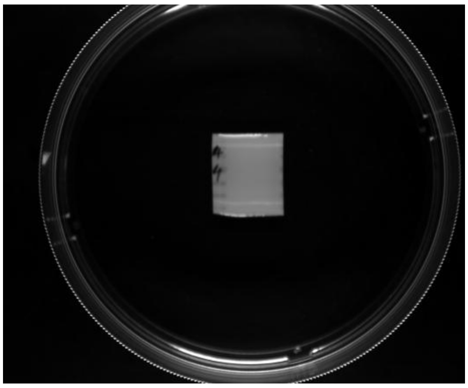

PRDX3 (monomer, SE, untreated, cytosol)

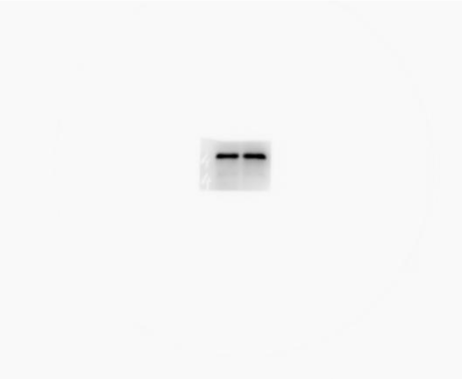

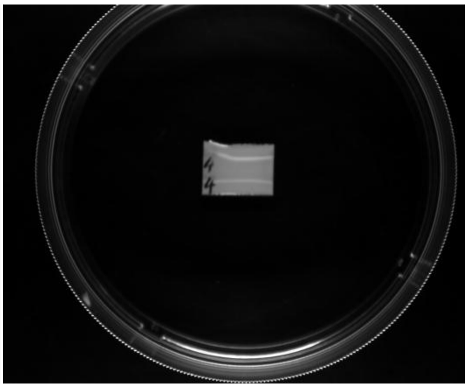

GAPDH (untreated, cytosol)

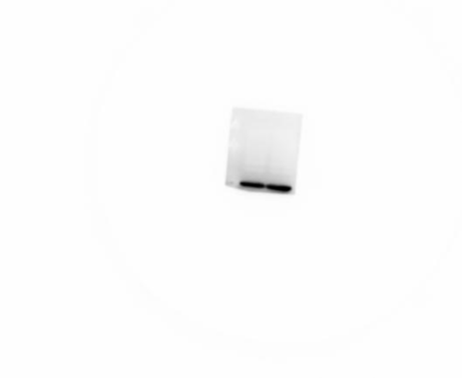

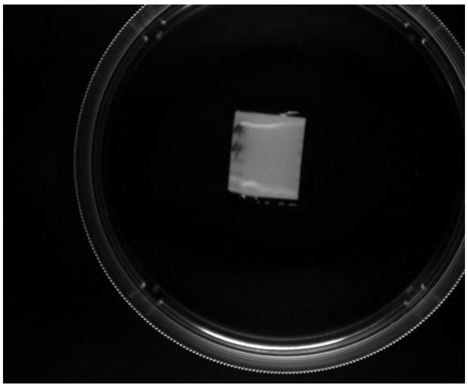

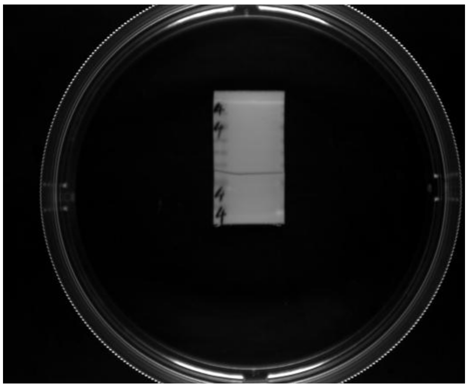

PRDX3 (SE, untreated, plasma membrane)

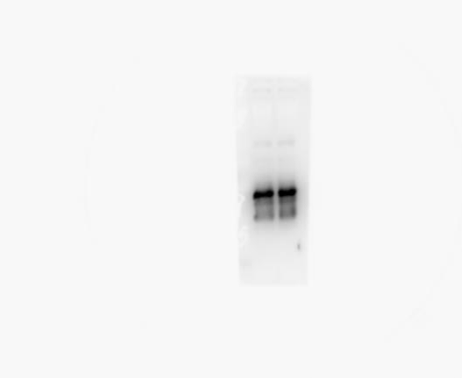

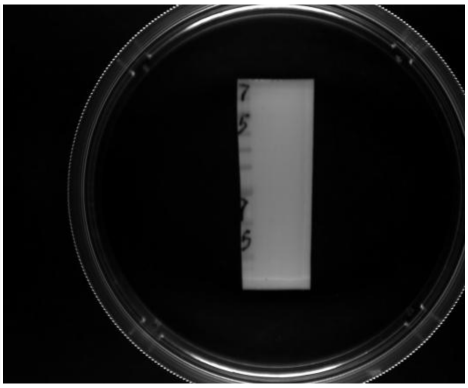

PRDX3 (dimer, LE, untreated, plasma membrane)

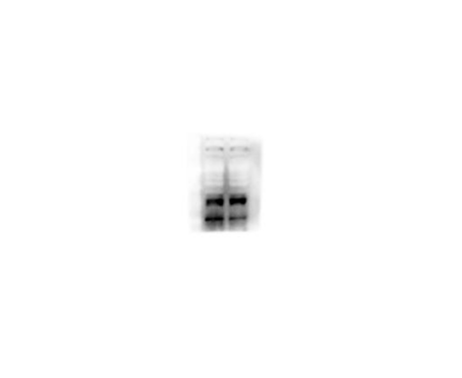

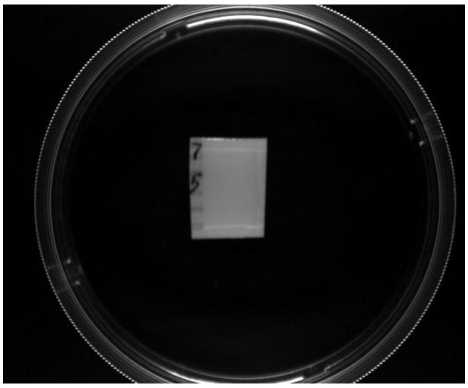

PRDX3 (monomer, SE, untreated, plasma membrane)

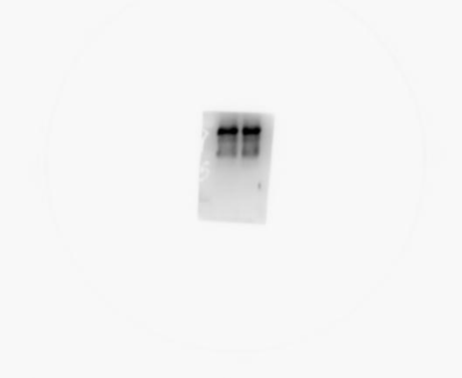

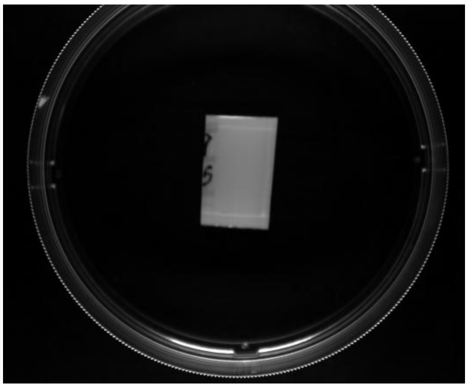

Na^+/K^+ ATPase a1 (untreated, plasma membrane)

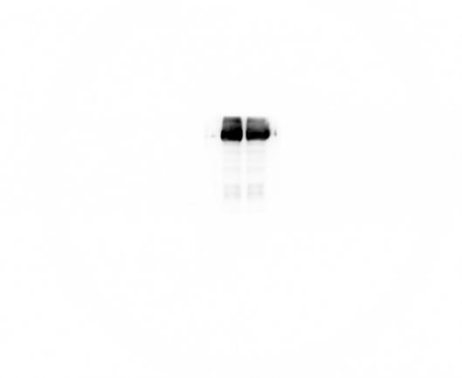

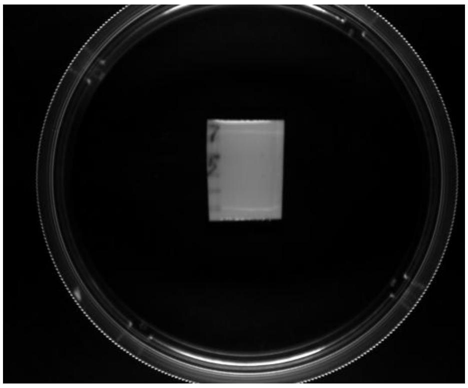

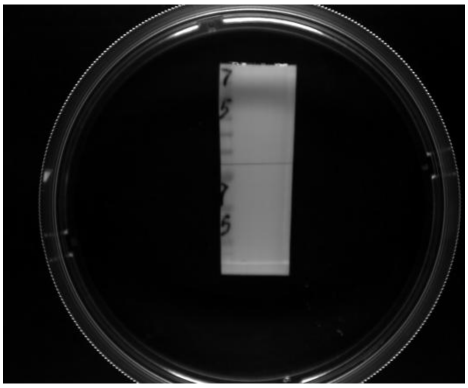

PRDX3 (SE, untreated, organelle)

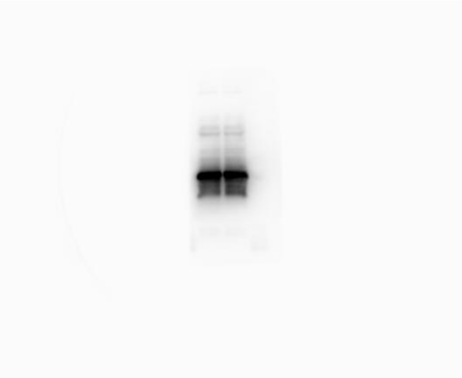

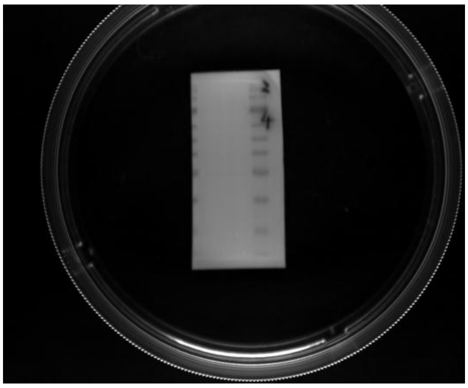

PRDX3 (dimer, LE, untreated, organelle)

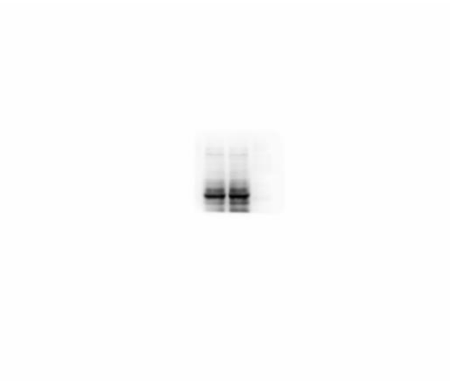

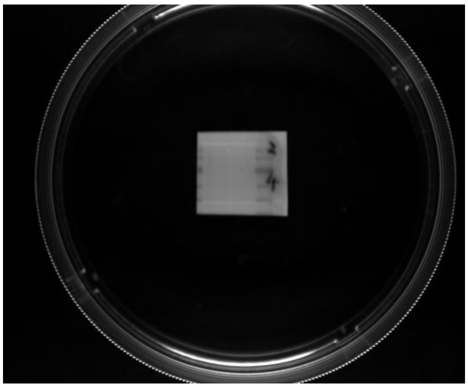

PRDX3 (monomer, SE, untreated, organelle)

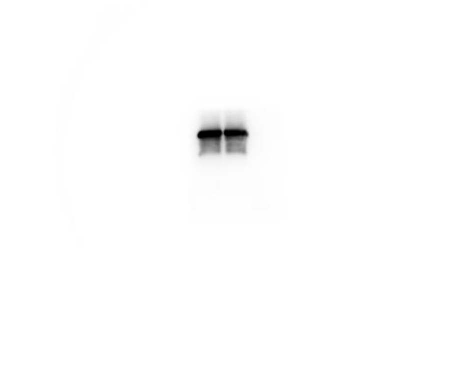

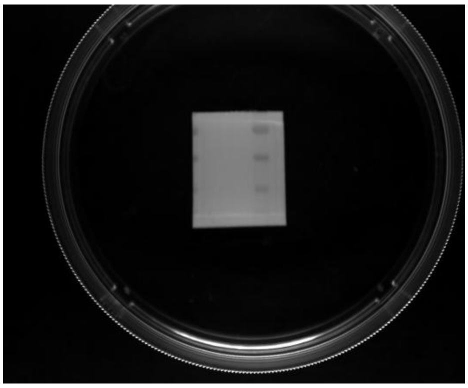

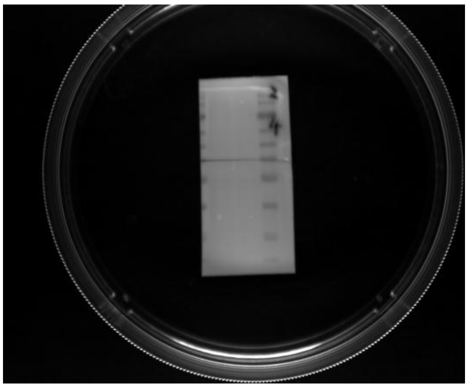

COX IV (untreated, organelle)

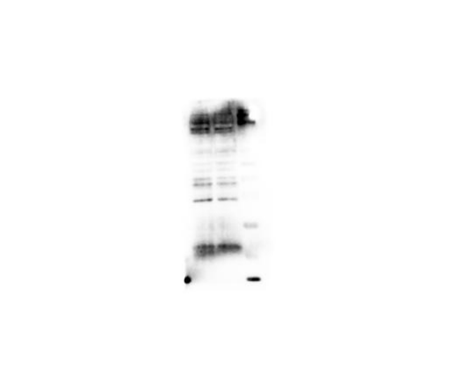

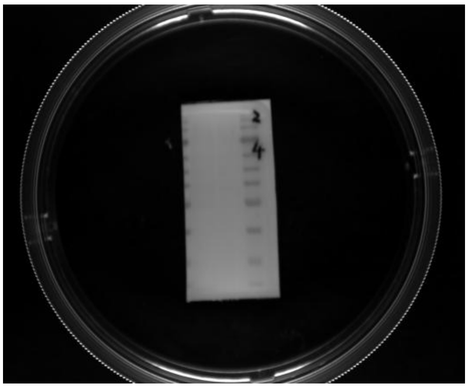

PRDX3 (SE, cisplatin, total)

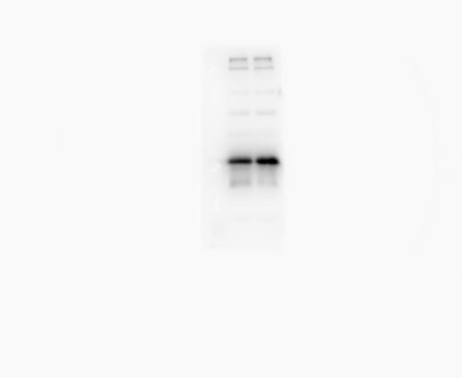

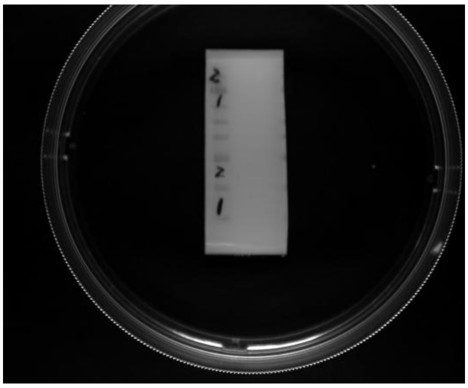

PRDX3 (dimer, LE, cisplatin, total)

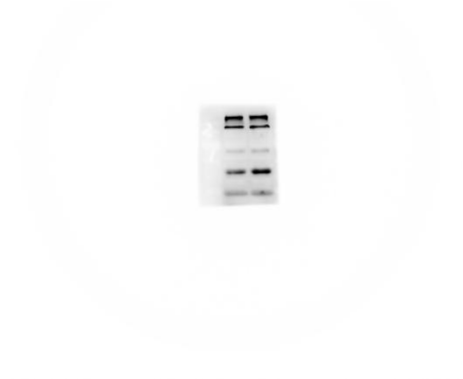

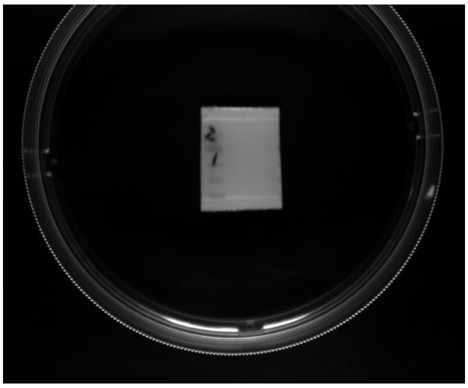

PRDX3 (monomer, SE, cisplatin, total)

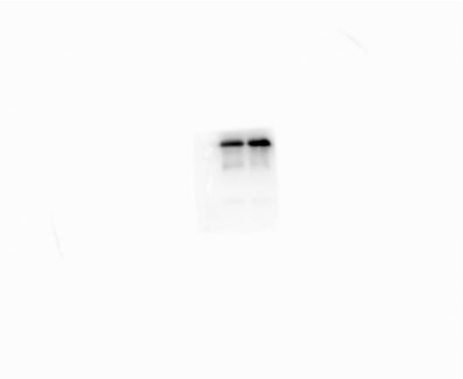

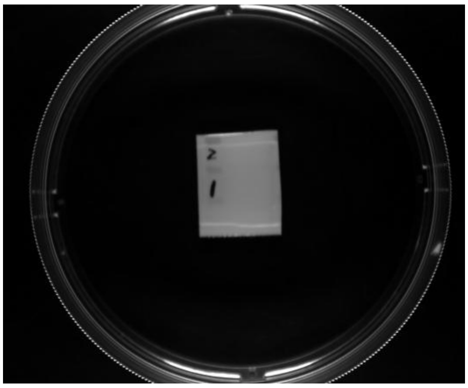

GAPDH (cisplatin, total)

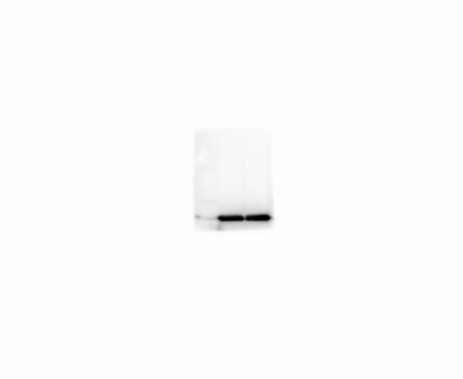

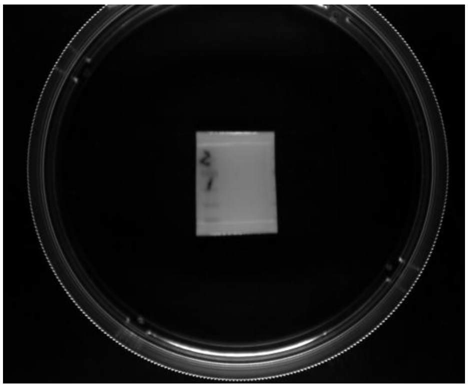

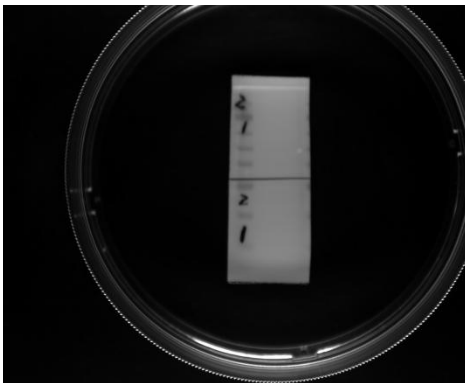

PRDX3 (SE, cisplatin, cytosol)

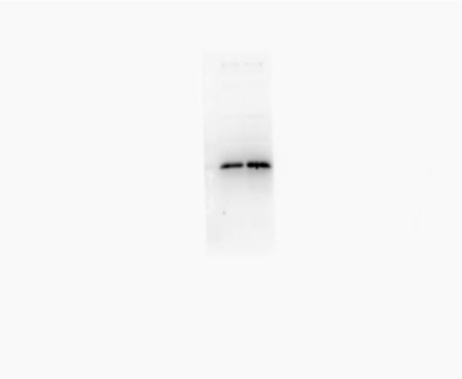

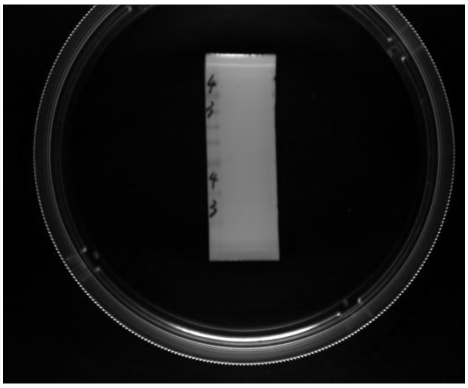

PRDX3 (dimer, LE, cisplatin, cytosol)

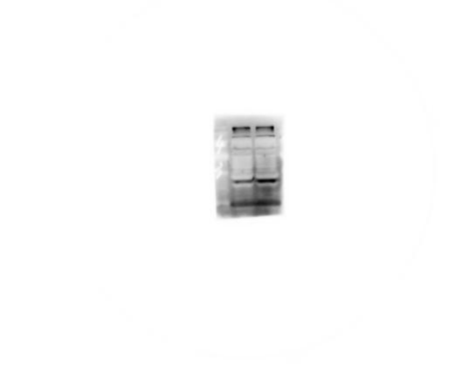

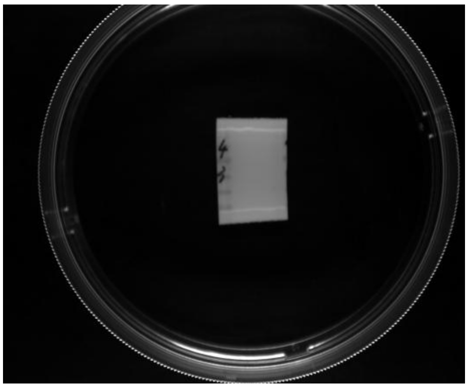

PRDX3 (monomer, SE, cisplatin, cytosol)

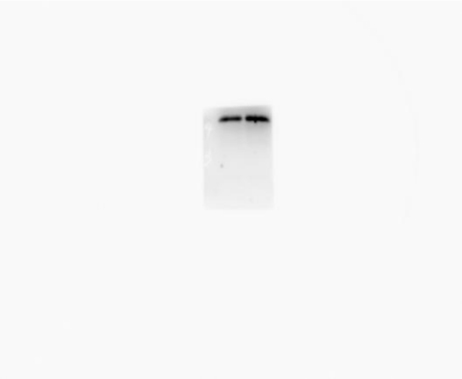

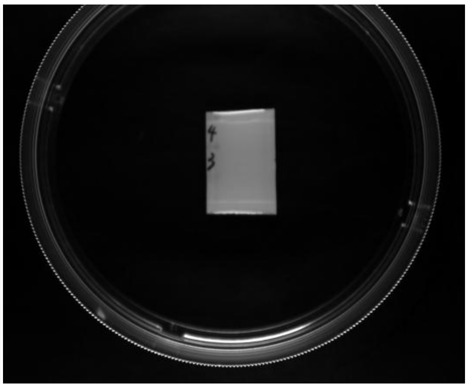

GAPDH (cisplatin, cytosol)

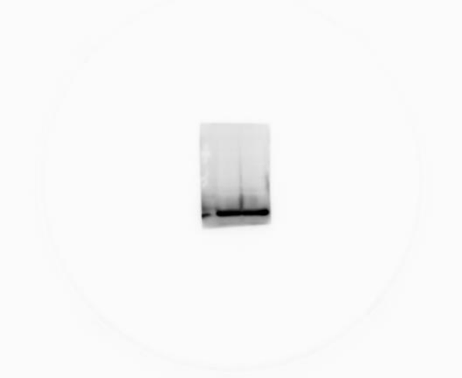

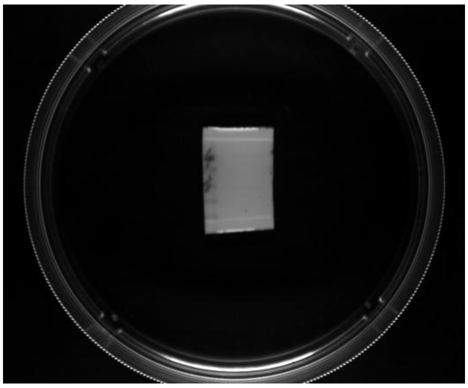

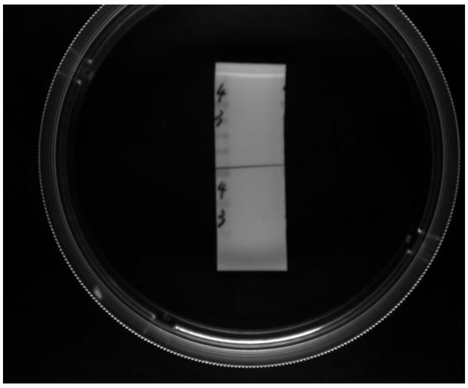

PRDX3 (SE, cisplatin, plasma membrane)

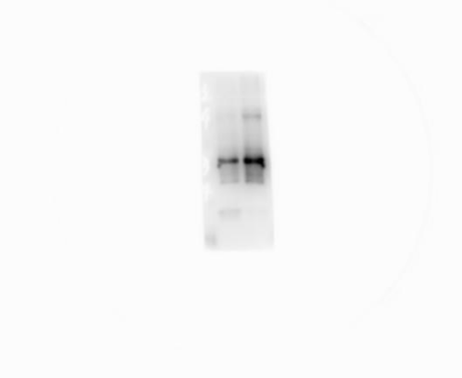

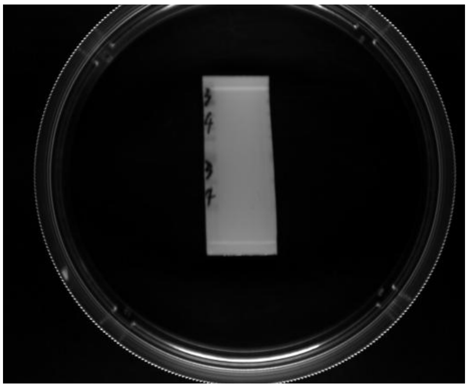

PRDX3 (dimer, LE, cisplatin, plasma membrane)

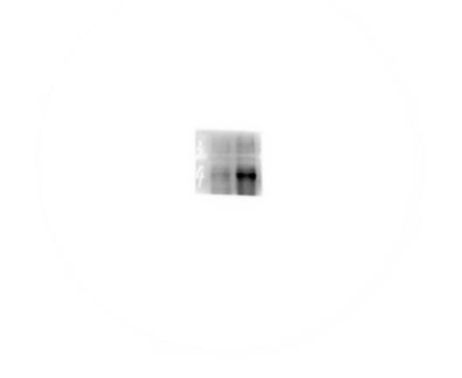

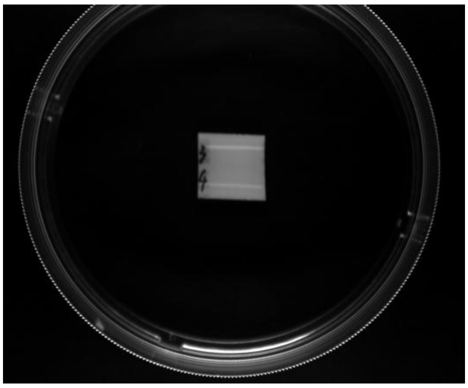

PRDX3 (monomer, SE, cisplatin, plasma membrane)

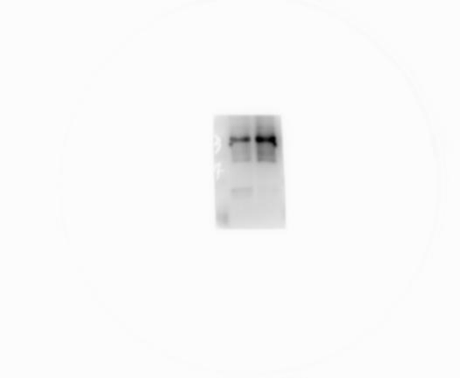
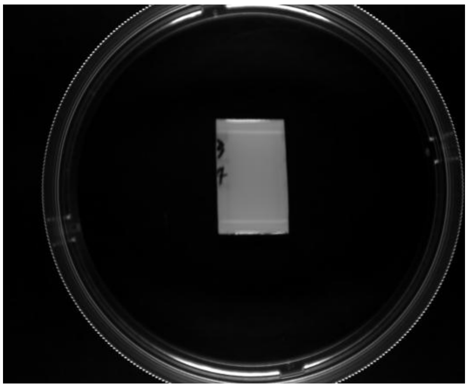

Na^+/K^+ ATPase a1 (cisplatin, plasma membrane)

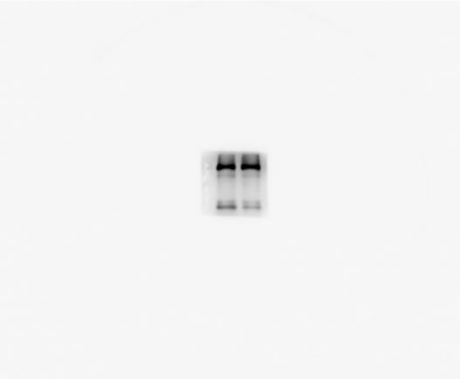
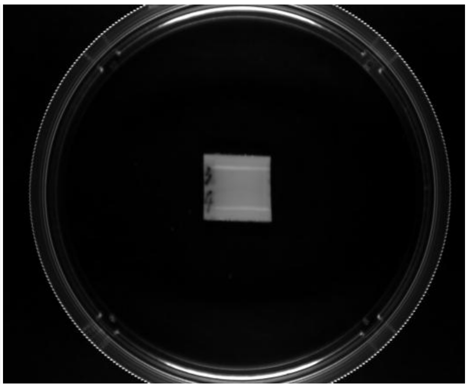

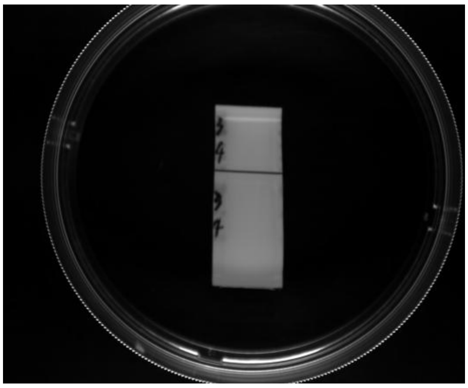

PRDX3 (SE, cisplatin, organelle)

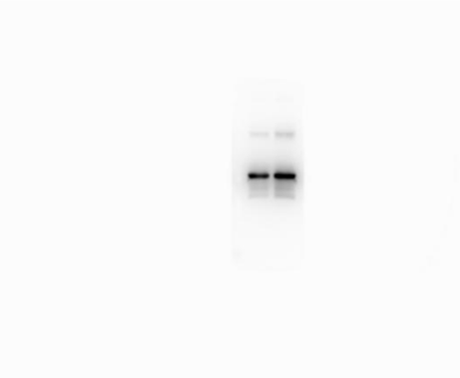
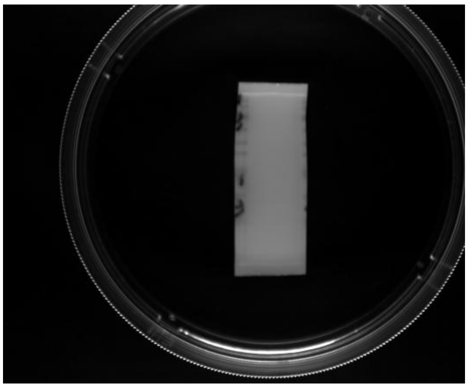

PRDX3 (dimer, LE, cisplatin, organelle)

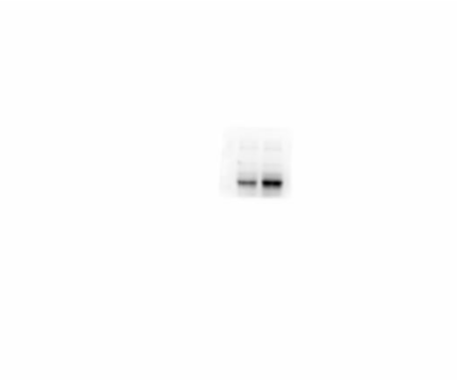

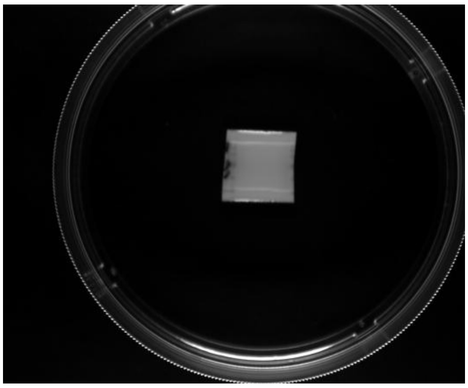

PRDX3 (monomer, SE, cisplatin, organelle)

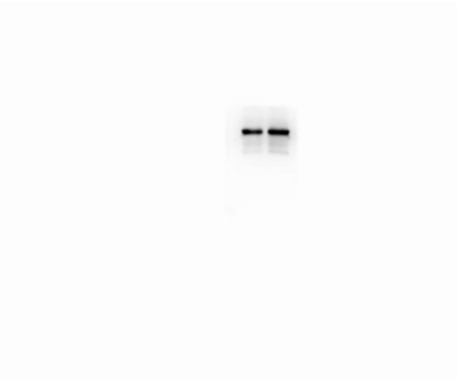

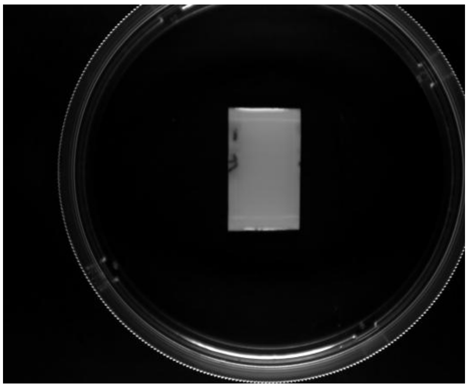

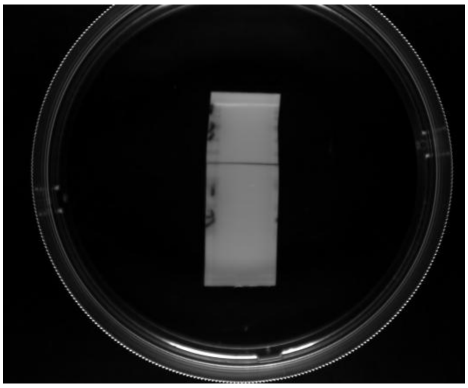

COX IV (cisplatin, organelle)

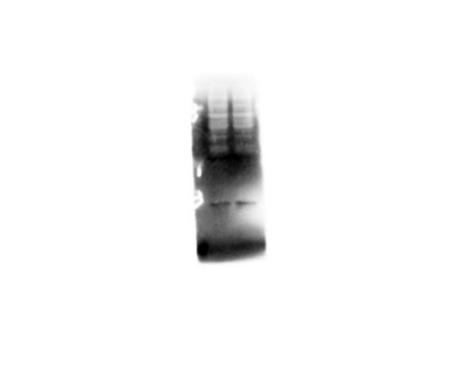

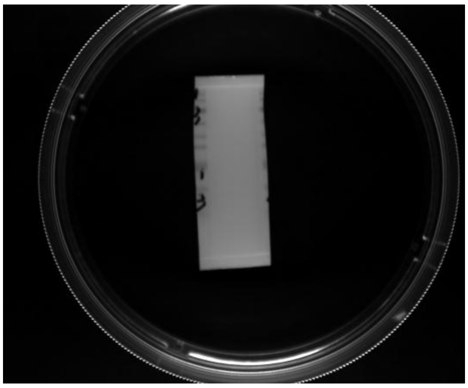

PRDX3 (SE, erastin, total)

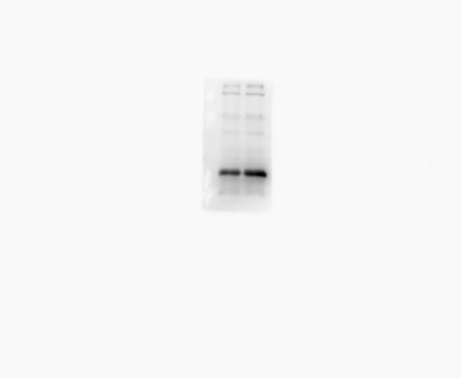
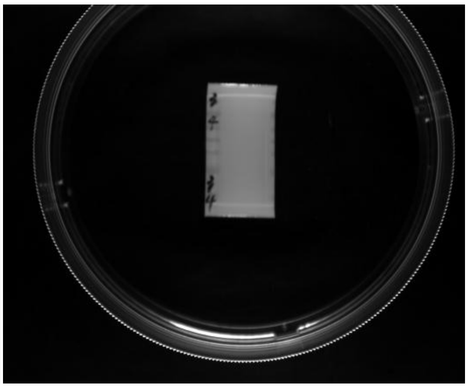

PRDX3 (dimer, LE, erastin, total)

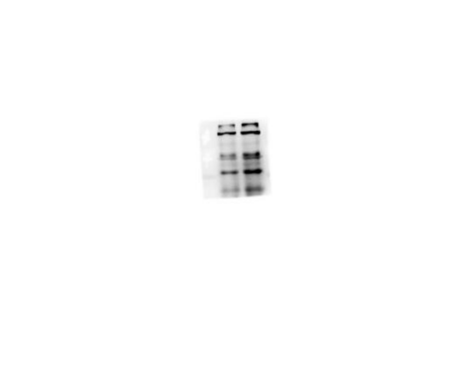
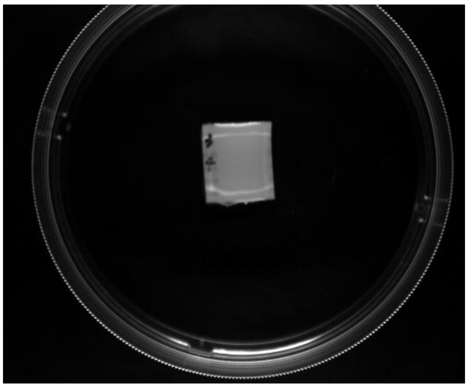

PRDX3 (monomer, SE, erastin, total)

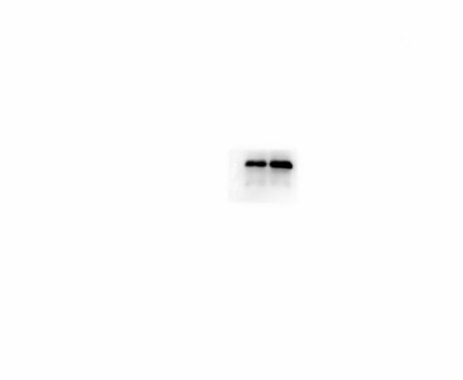
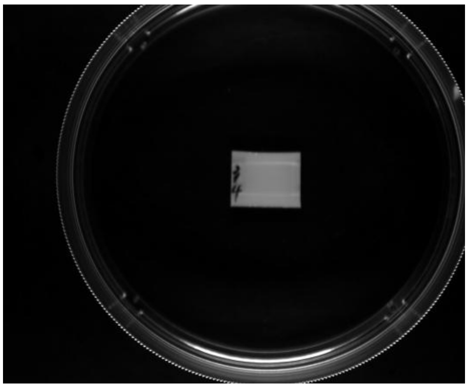

GAPDH (erastin, total)

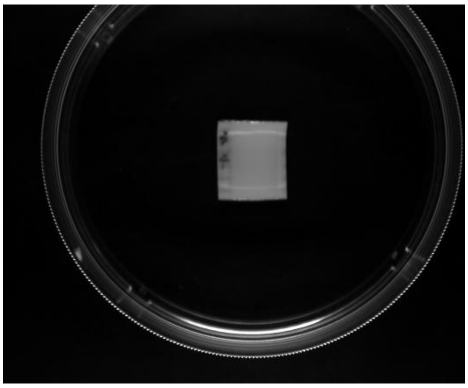

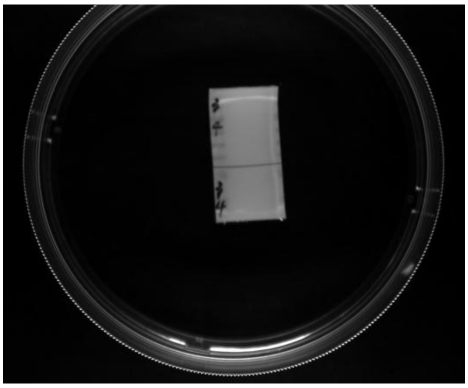

PRDX3 (SE, erastin, cytosol)

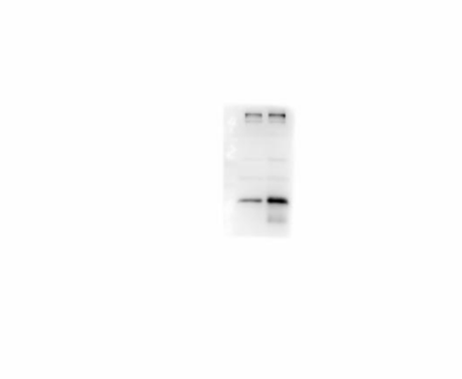

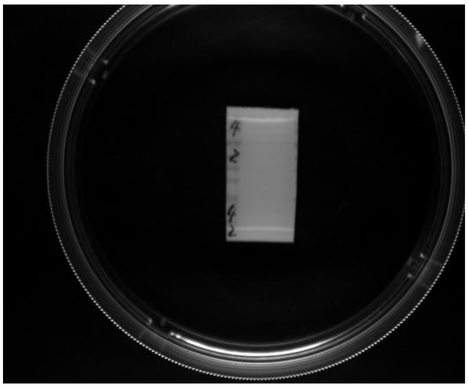

PRDX3 (dimer, LE, erastin, cytosol)

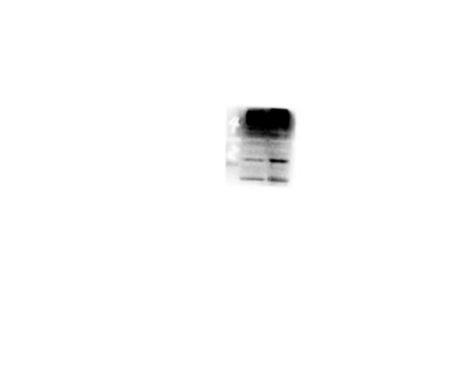

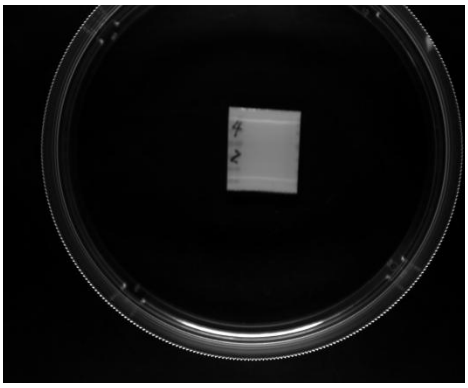

PRDX3 (monomer, SE, erastin, cytosol)

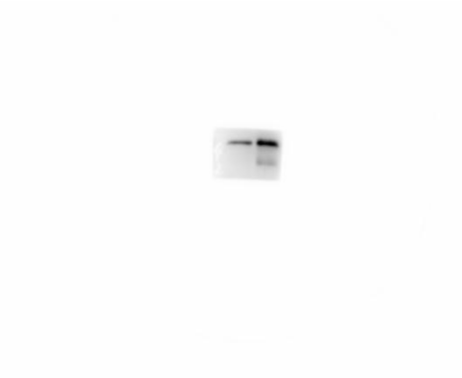

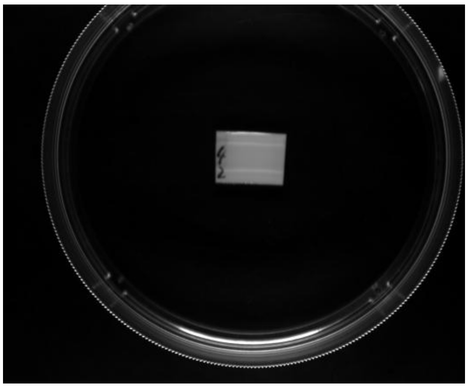

GAPDH (erastin, cytosol)

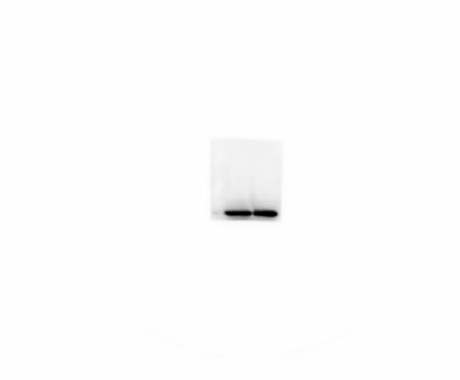

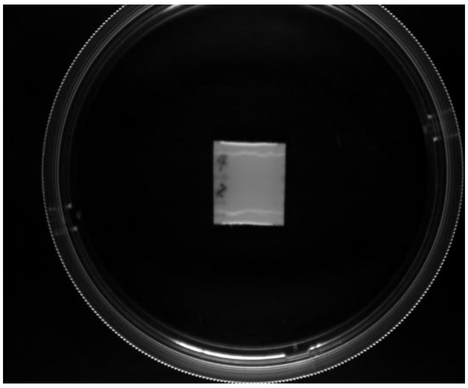

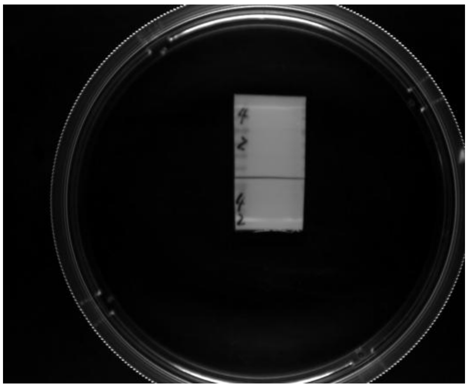

PRDX3 (SE, erastin, plasma membrane)

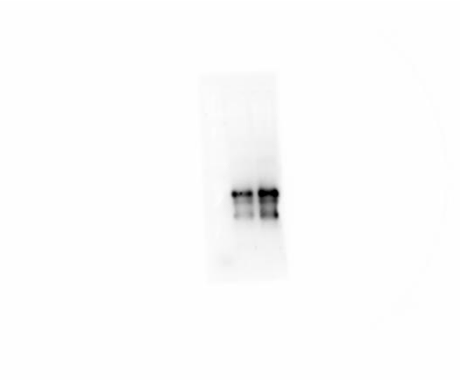

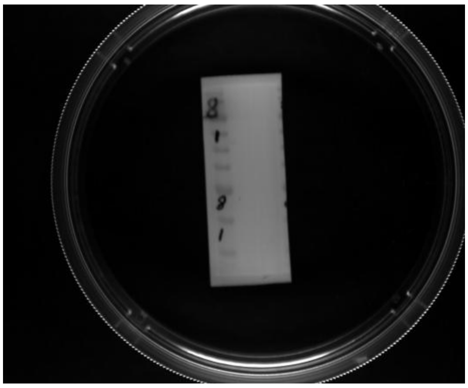

PRDX3 (dimer, LE, erastin, plasma membrane)

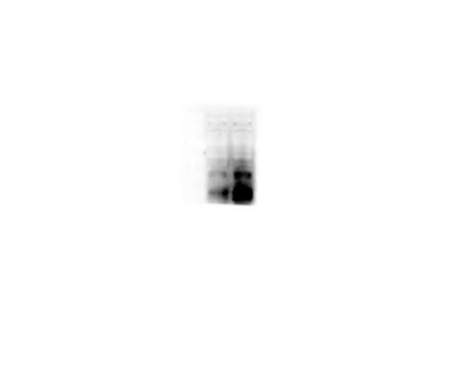

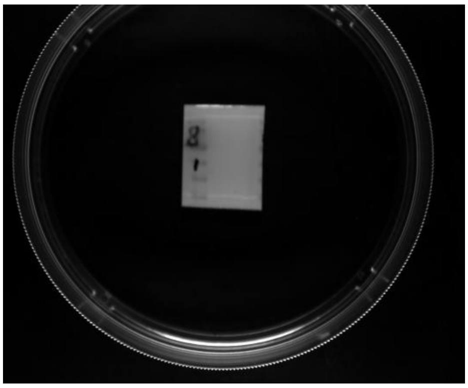

PRDX3 (monomer, SE, erastin, plasma membrane)

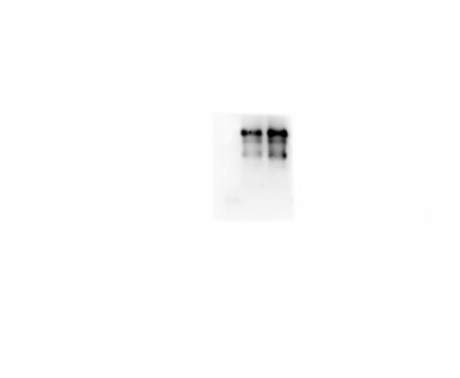

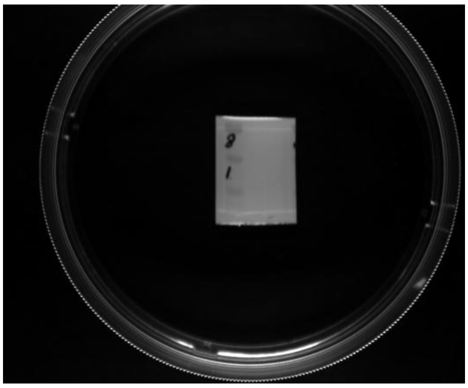

Na^+/K^+ ATPase a1 (erastin, plasma membrane)

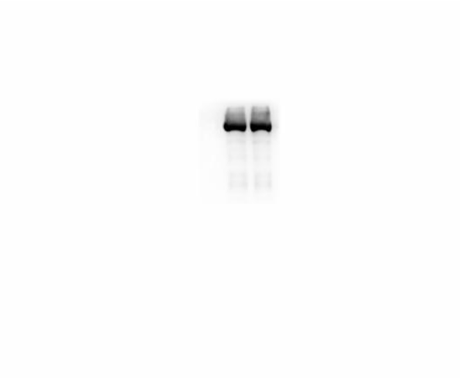

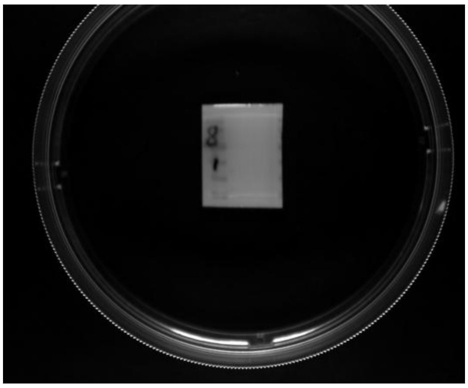

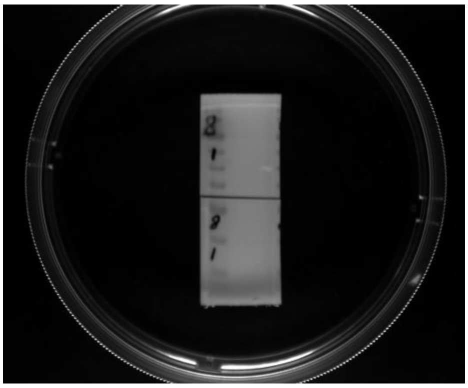

PRDX3 (SE, erastin, organelle)

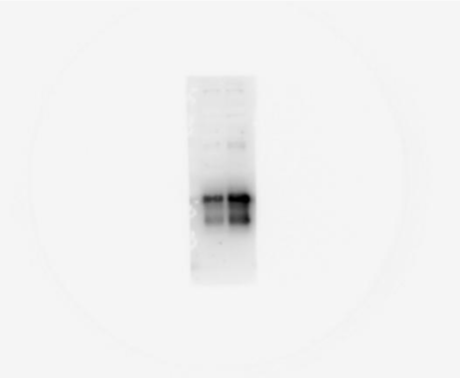

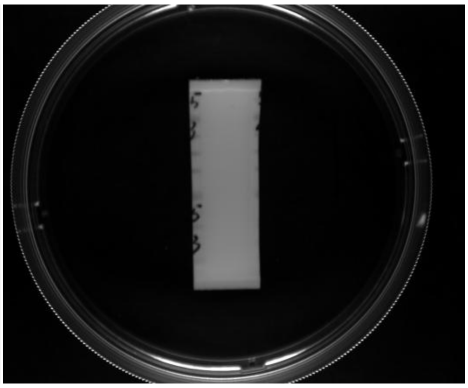

PRDX3 (dimer, LE, erastin, organelle)

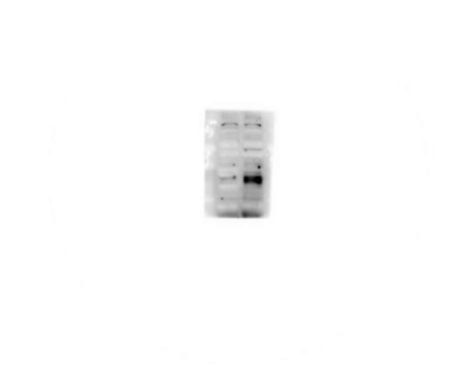

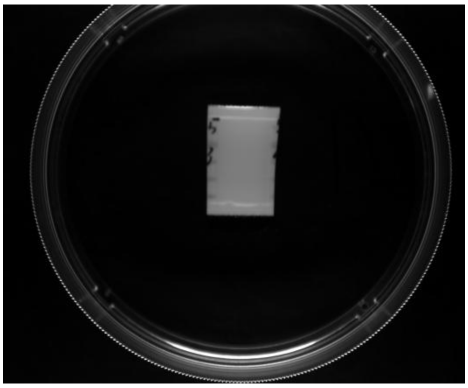

PRDX3 (monomer, SE, erastin, organelle)

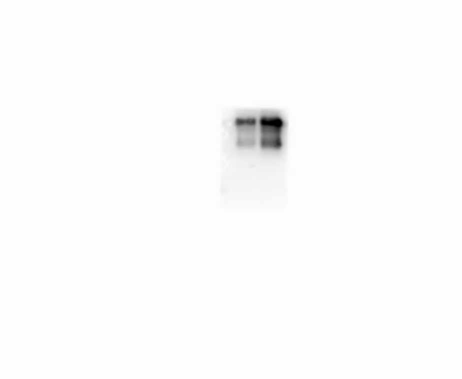

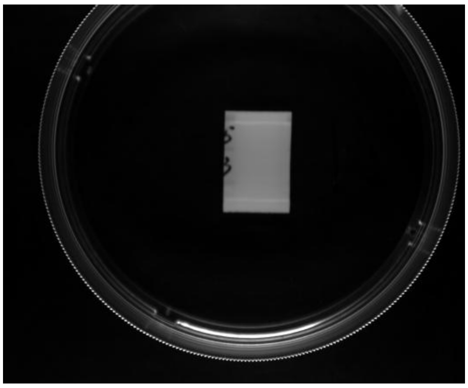

COX IV (erastin, organelle)

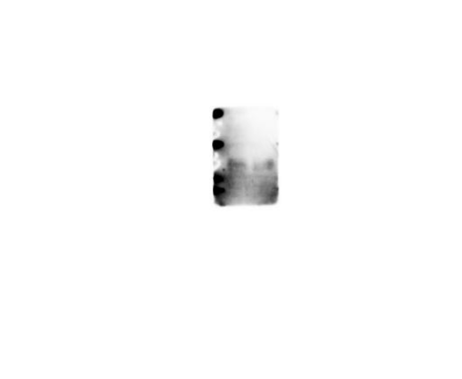

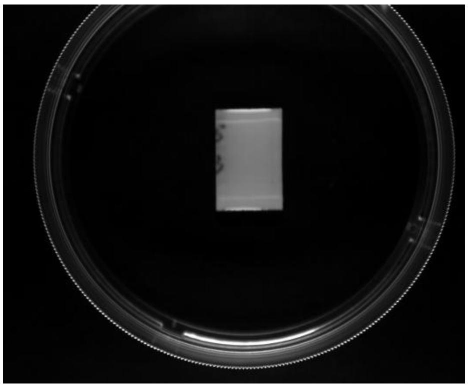

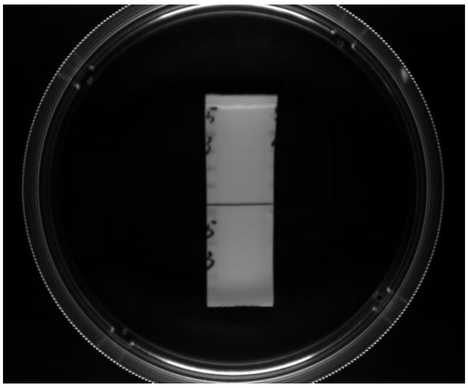

Fig. 7J Third Repetition

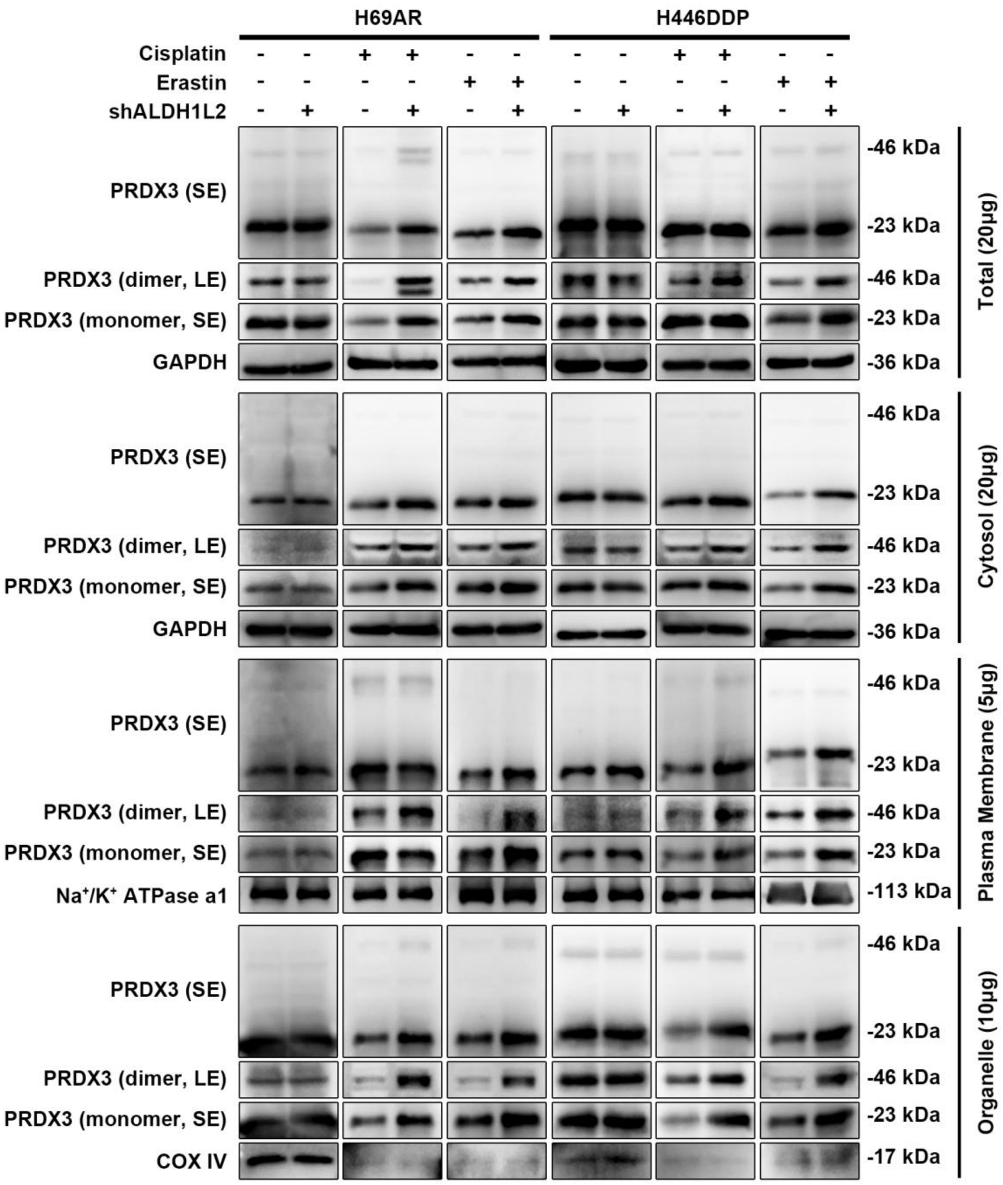

H69AR

PRDX3 (SE, untreated, total)

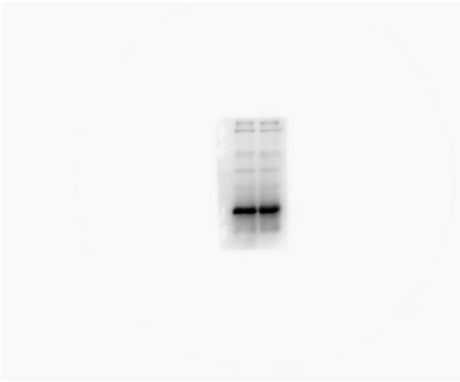

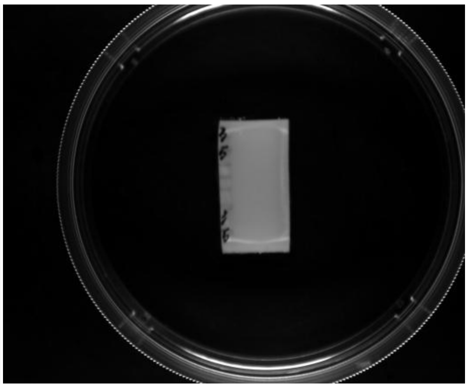

PRDX3 (dimer, LE, untreated, total)

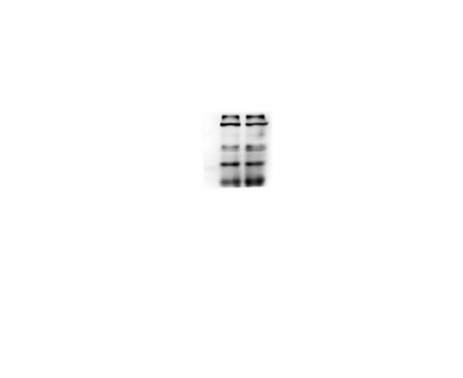
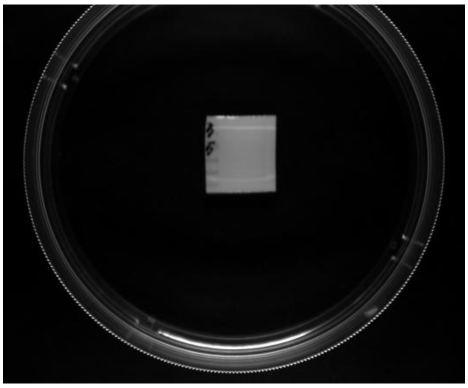

PRDX3 (monomer, SE, untreated, total)

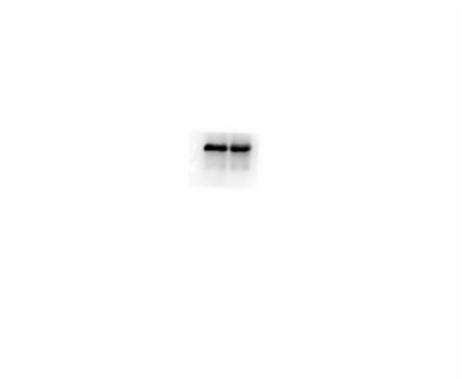
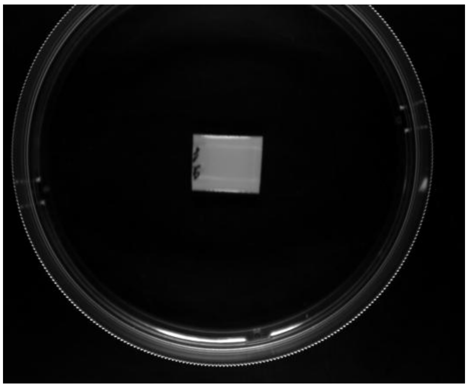

GAPDH (untreated, total)

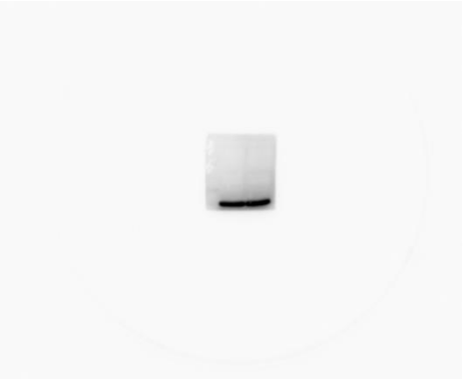
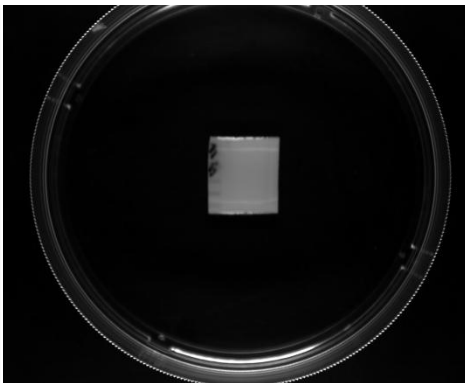

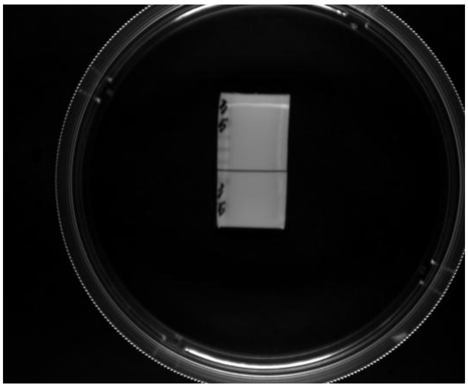

PRDX3 (SE, untreated, cytosol)

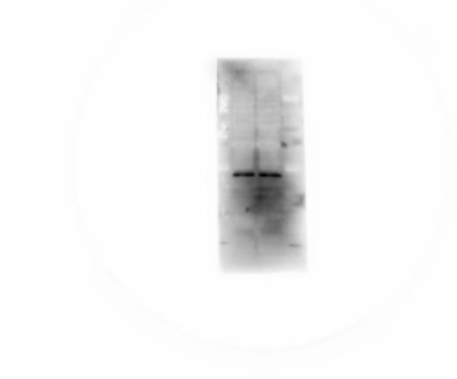

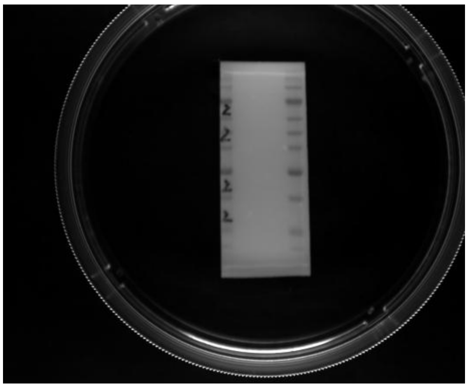

PRDX3 (dimer, LE, untreated, cytosol)

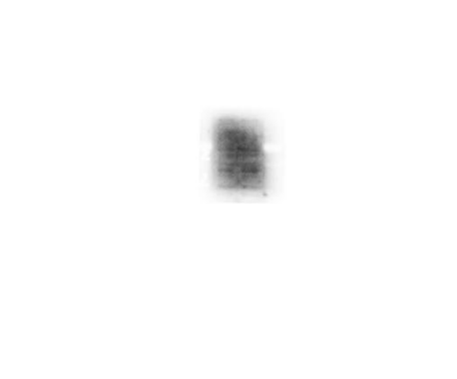

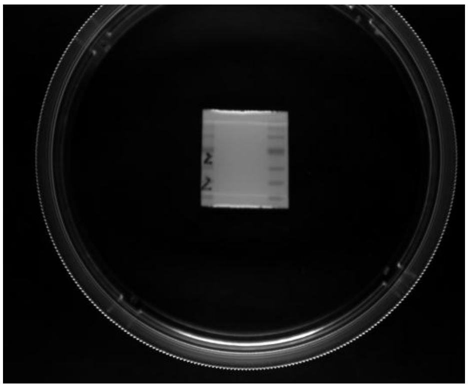

PRDX3 (monomer, SE, untreated, cytosol)

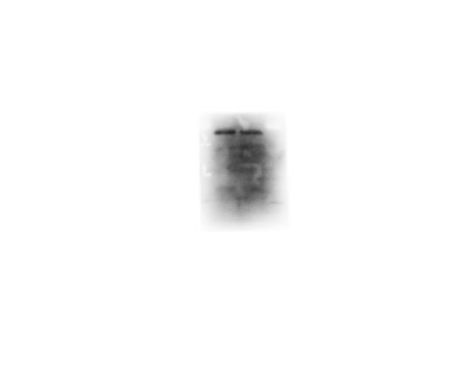

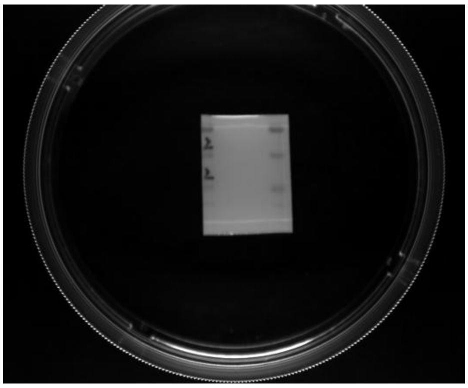

GAPDH (untreated, cytosol)

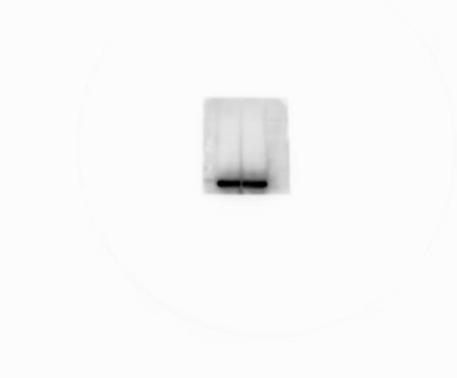

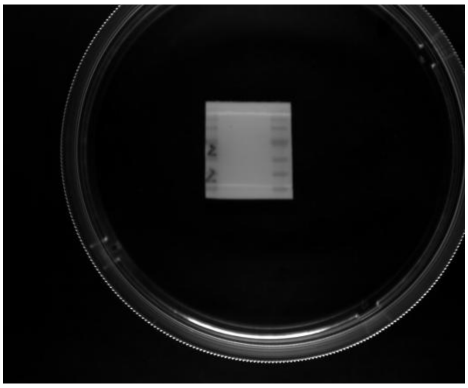

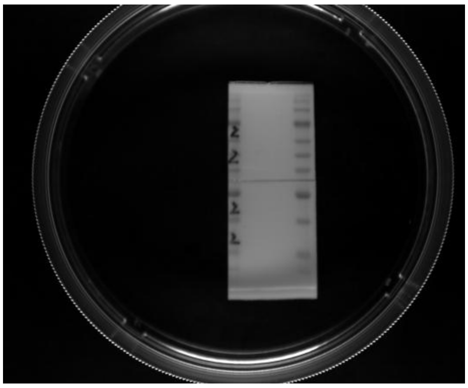

PRDX3 (SE, untreated, plasma membrane)

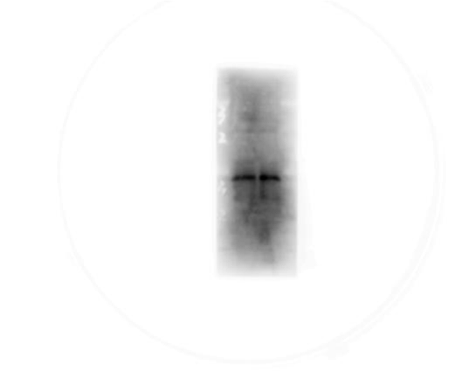

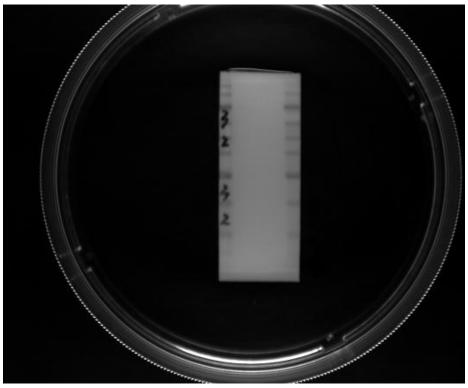

PRDX3 (dimer, LE, untreated, plasma membrane)

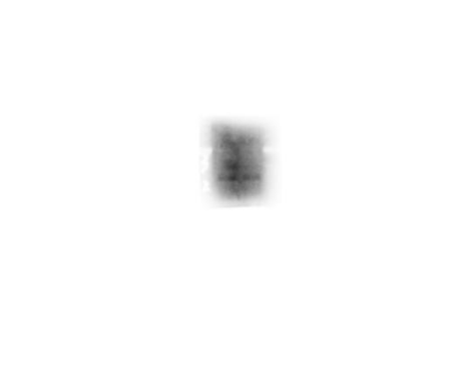

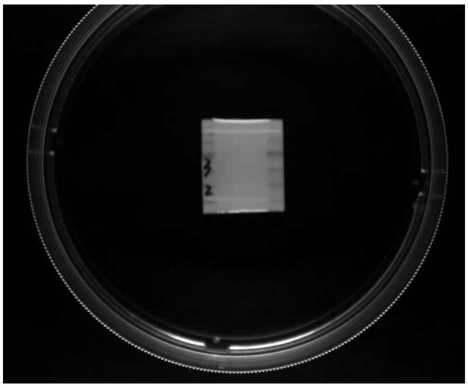

PRDX3 (monomer, SE, untreated, plasma membrane)

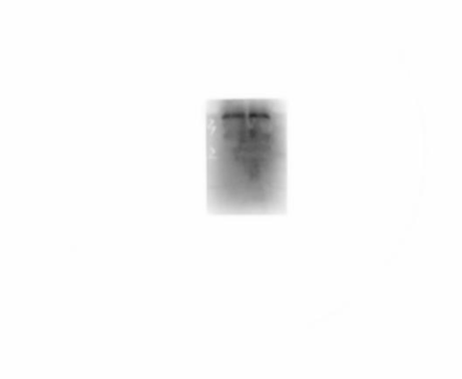

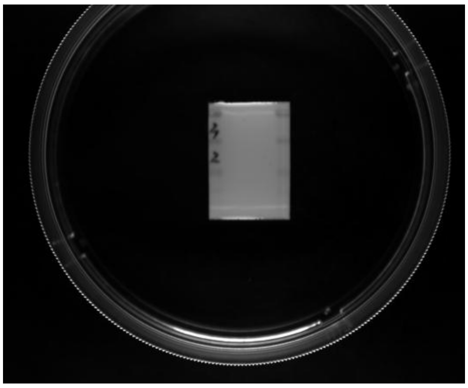

Na^{+}/K^{+} ATPase a1 (untreated, plasma membrane)

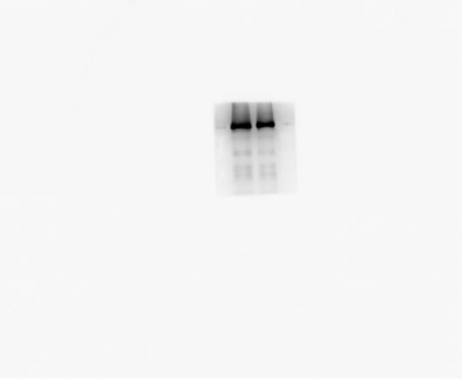
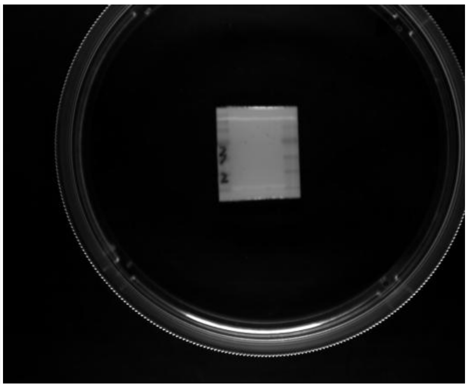

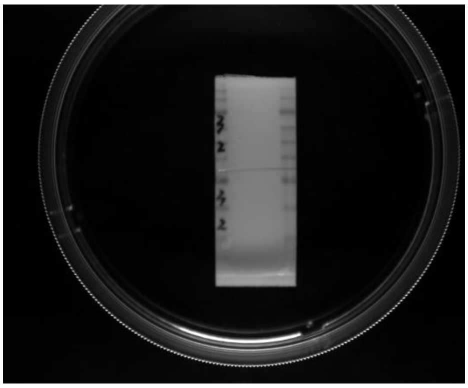

PRDX3 (SE, untreated, organelle)

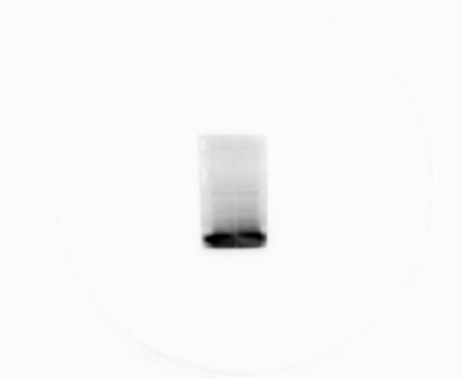
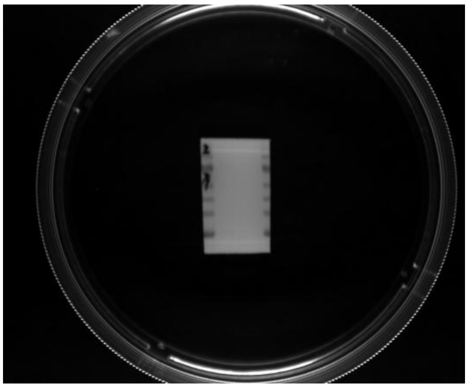

PRDX3 (dimer, LE, untreated, organelle)

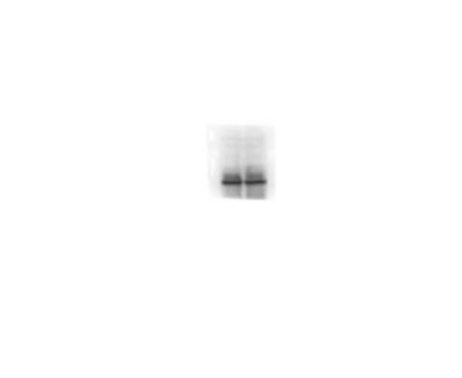
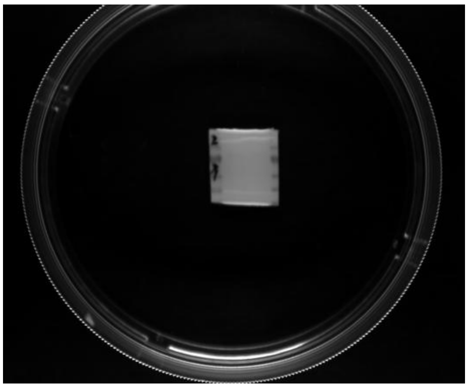

PRDX3 (monomer, SE, untreated, organelle)

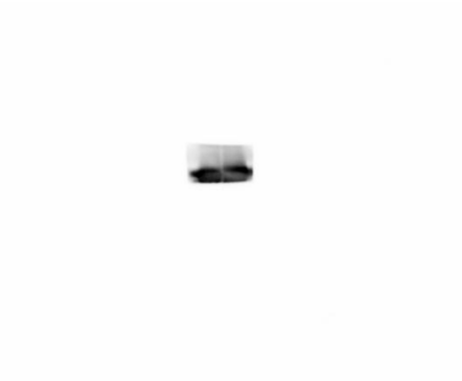

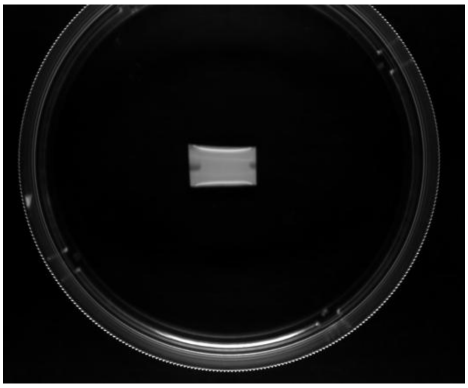

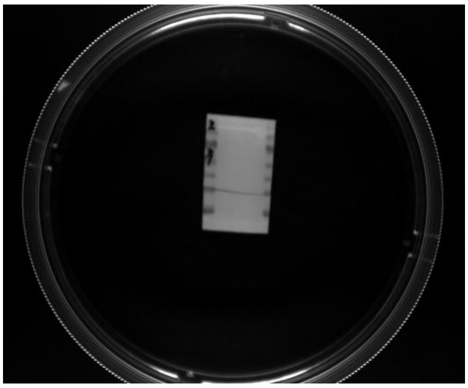

COX IV (untreated, organelle)

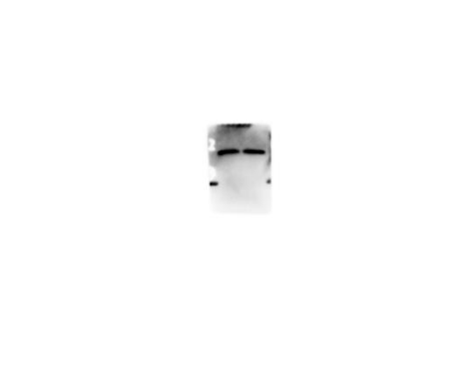

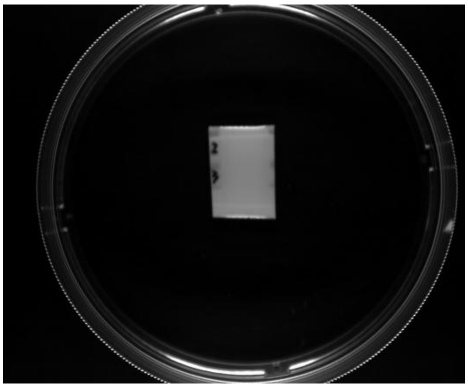

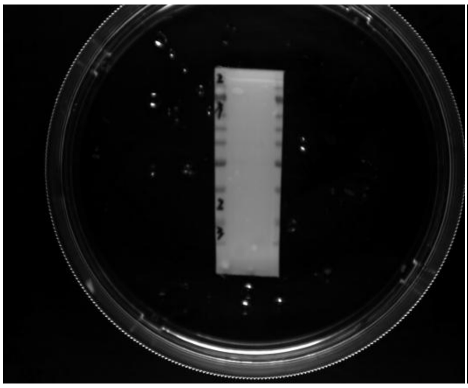

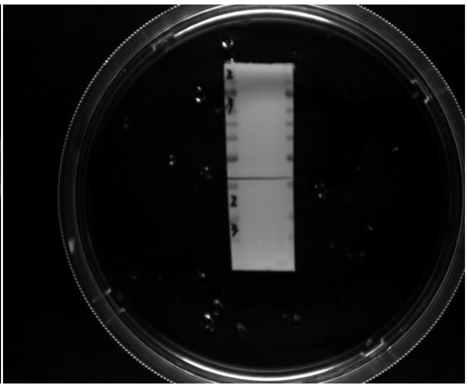

PRDX3 (SE, cisplatin, total)

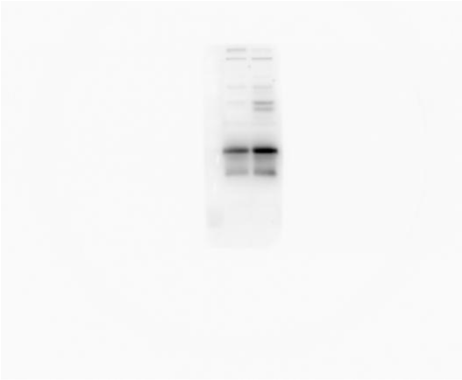

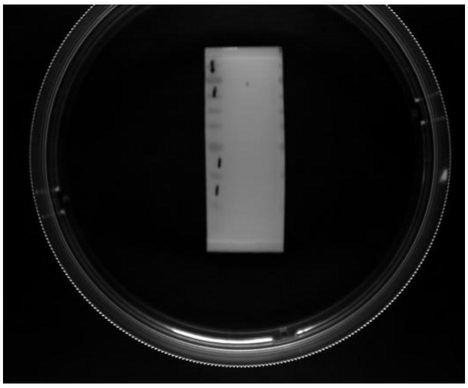

PRDX3 (dimer, LE, cisplatin, total)

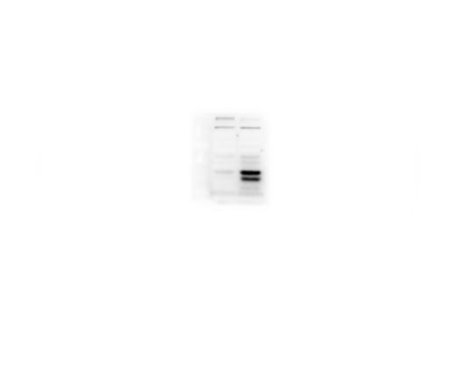

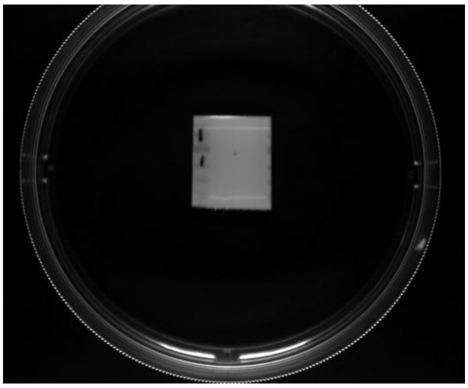

PRDX3 (monomer, SE, cisplatin, total)

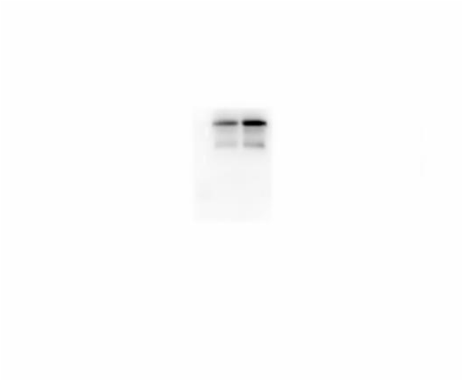

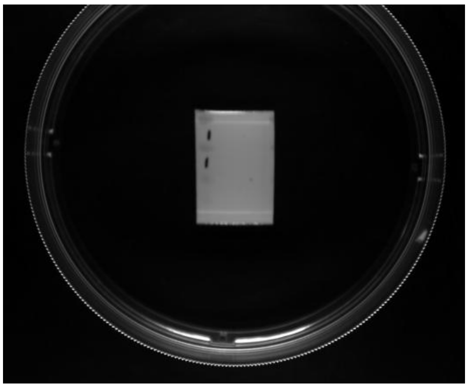

GAPDH (cisplatin, total)

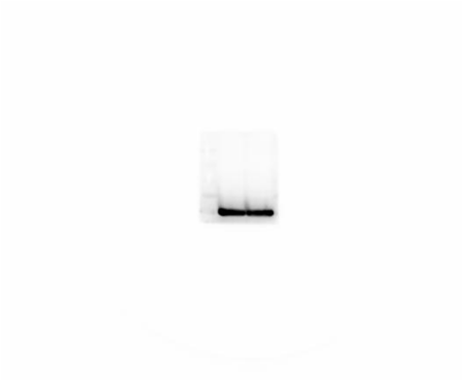

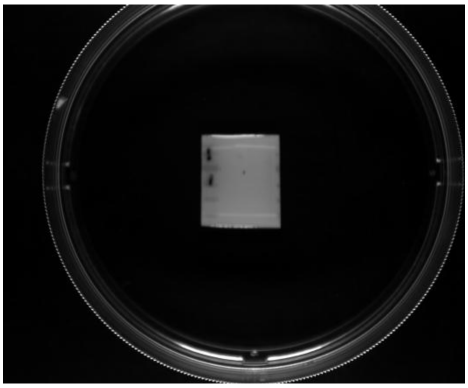

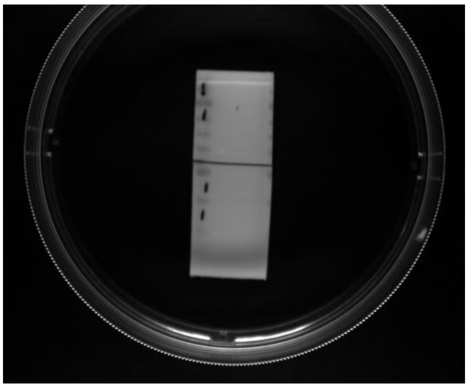

PRDX3 (SE, cisplatin, cytosol)

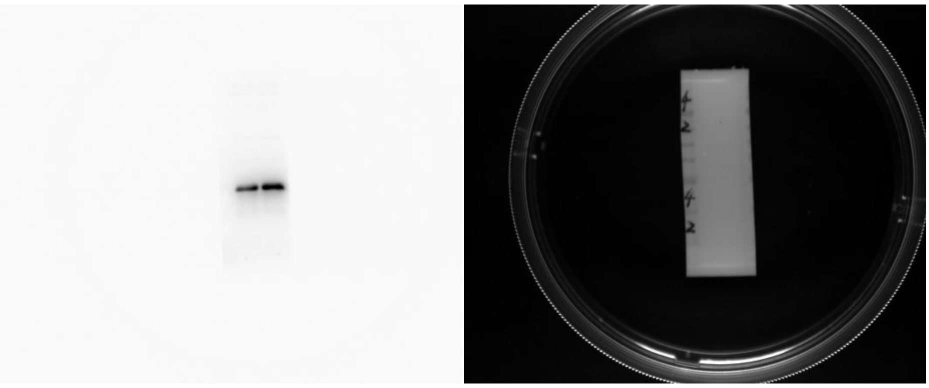

PRDX3 (dimer, LE, cisplatin, cytosol)

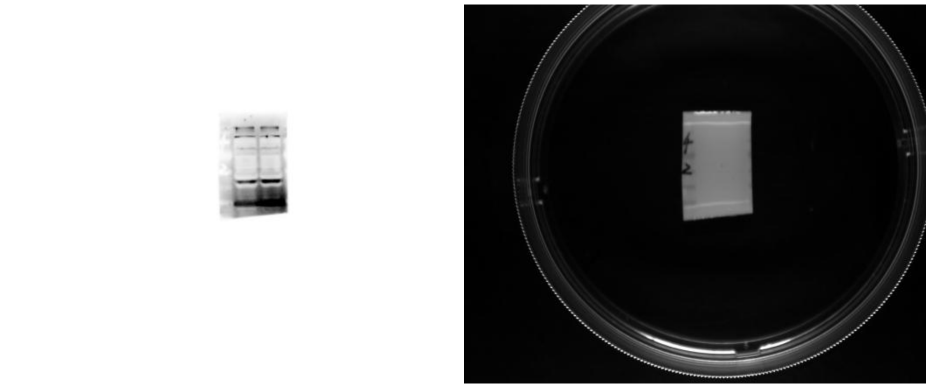

PRDX3 (monomer, SE, cisplatin, cytosol)

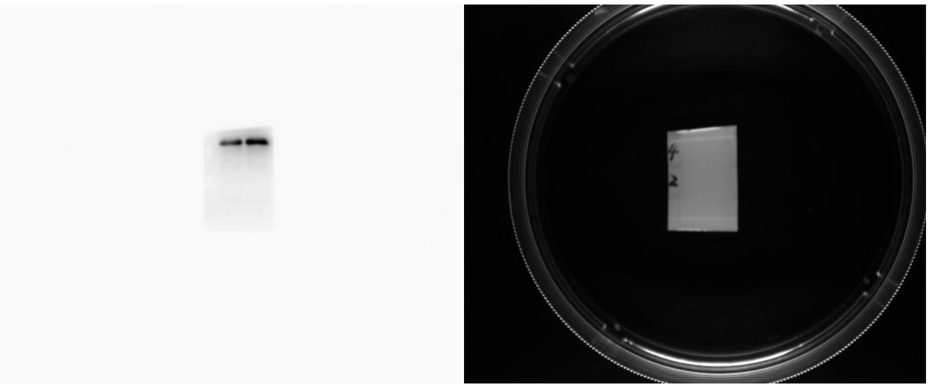

GAPDH (cisplatin, cytosol)

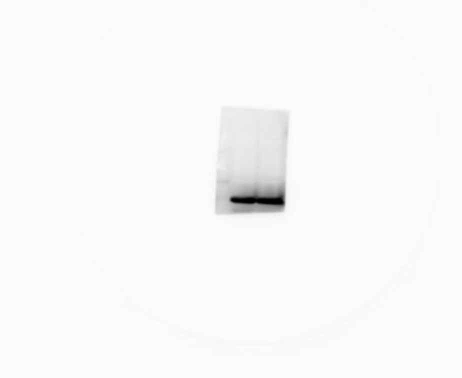

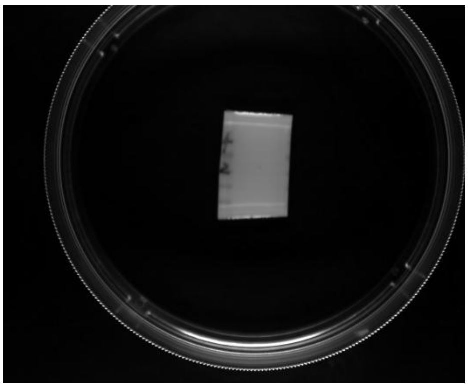

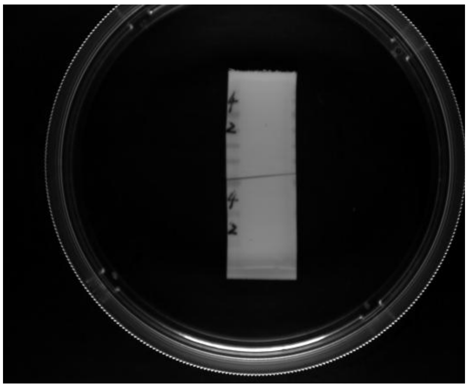

PRDX3 (SE, cisplatin, plasma membrane)

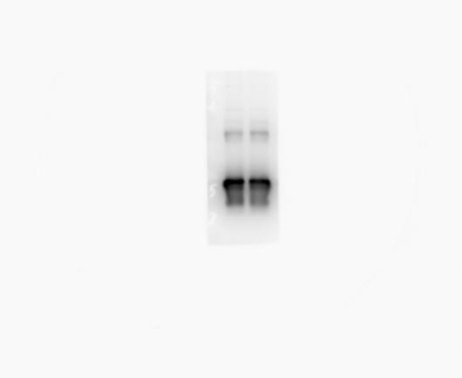

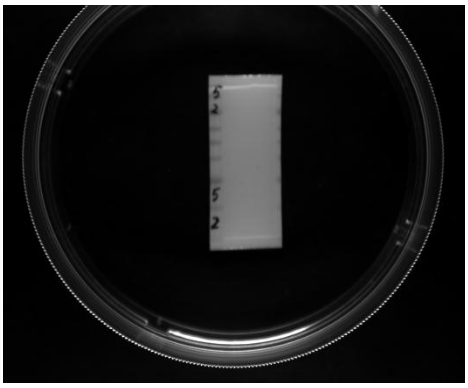

PRDX3 (dimer, LE, cisplatin, plasma membrane)

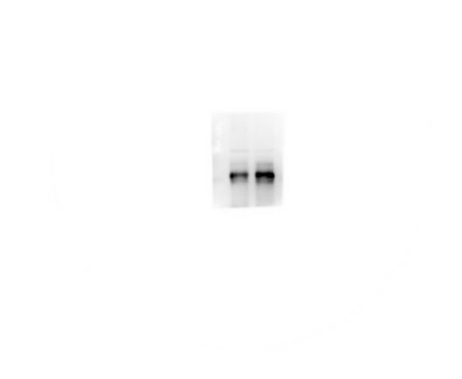

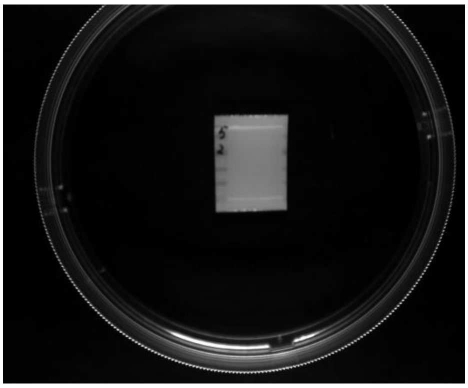

PRDX3 (monomer, SE, cisplatin, plasma membrane)

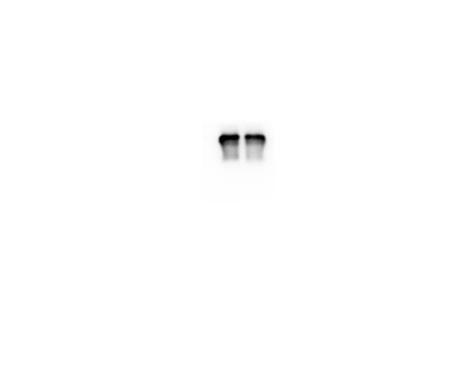

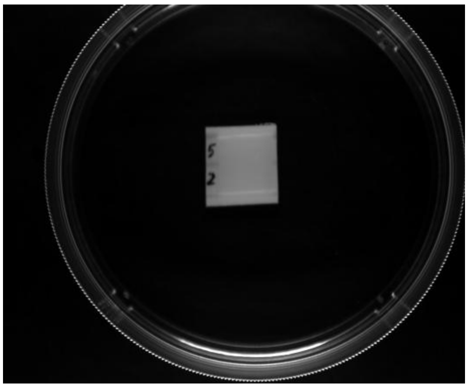

Na^+/K^+ ATPase a1 (cisplatin, plasma membrane)

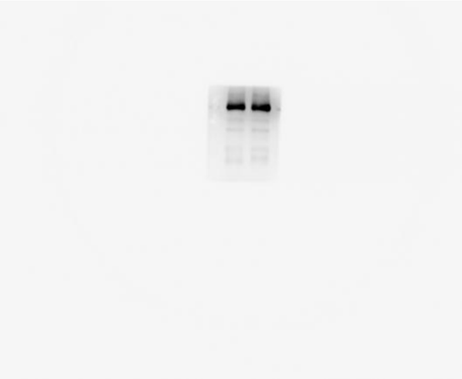

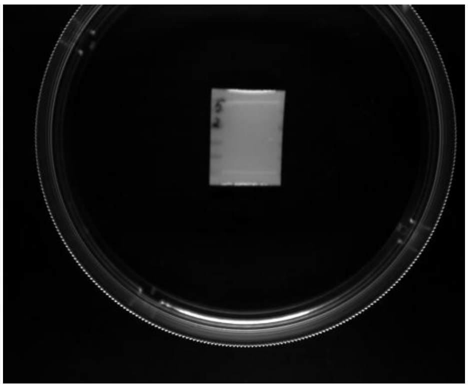

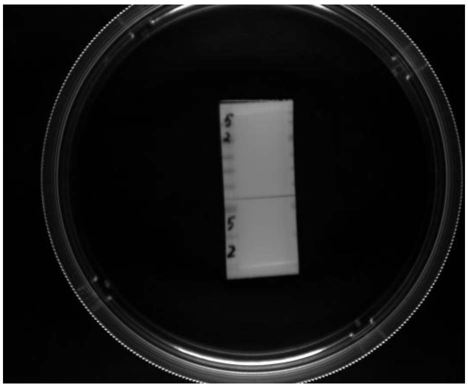

PRDX3 (SE, cisplatin, organelle)

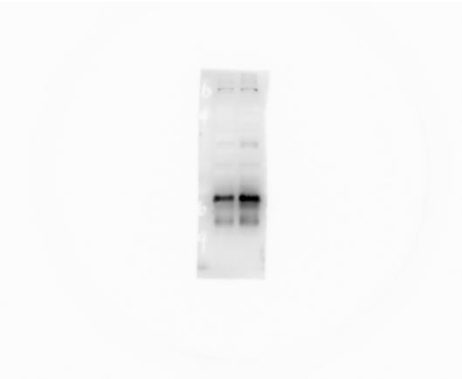

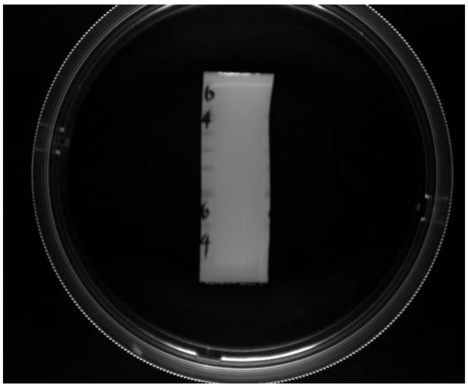

PRDX3 (dimer, LE, cisplatin, organelle)

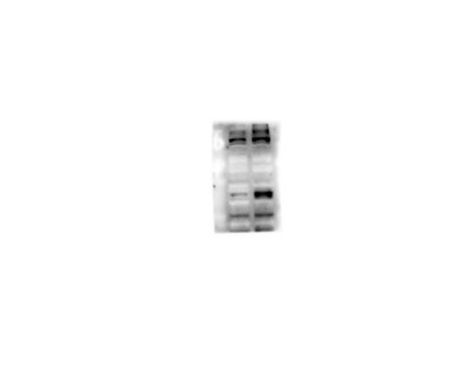

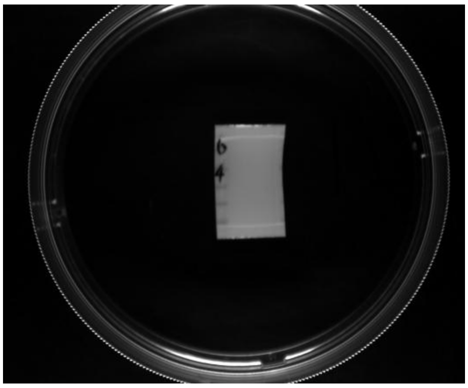

PRDX3 (monomer, SE, cisplatin, organelle)

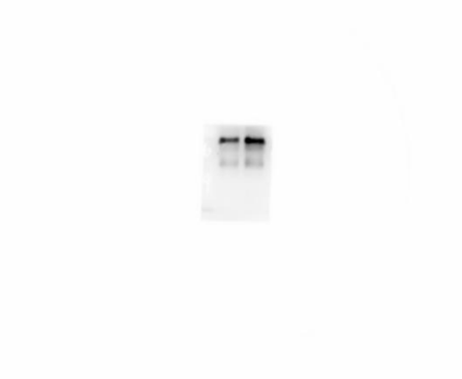

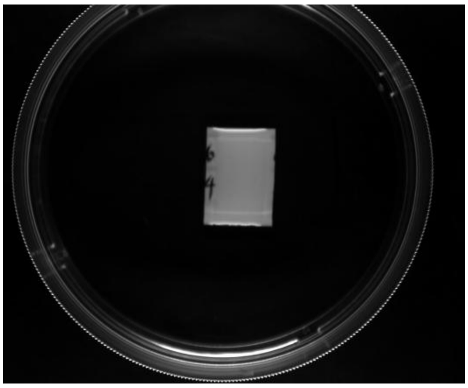

COX IV (cisplatin, organelle)

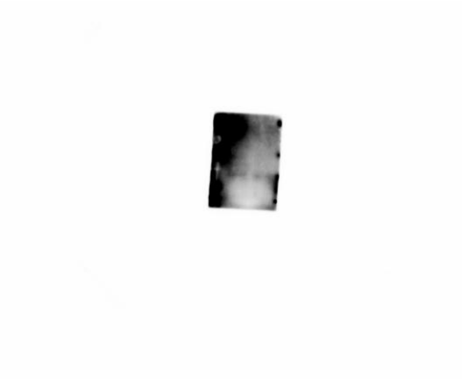

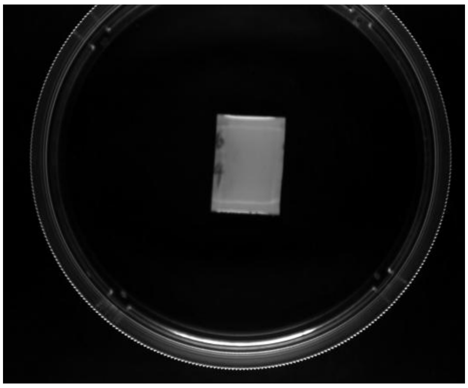

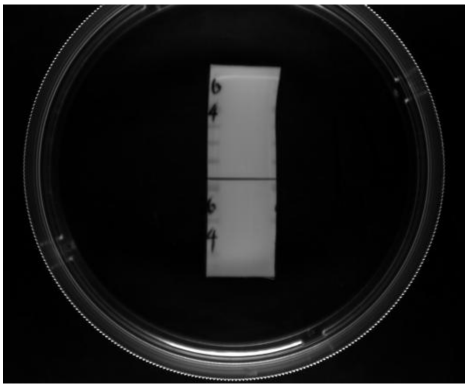

PRDX3 (SE, erastin, total)

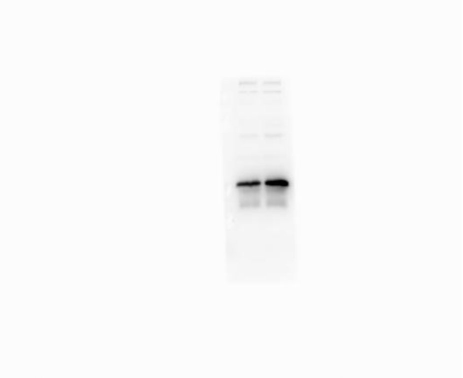

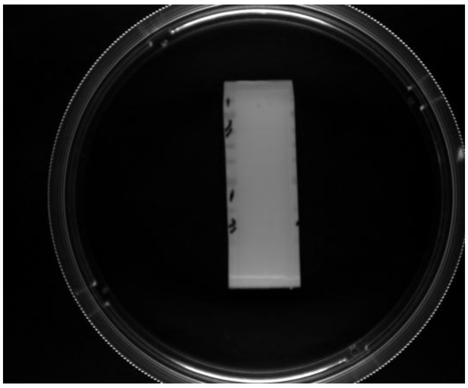

PRDX3 (dimer, LE, erastin, total)

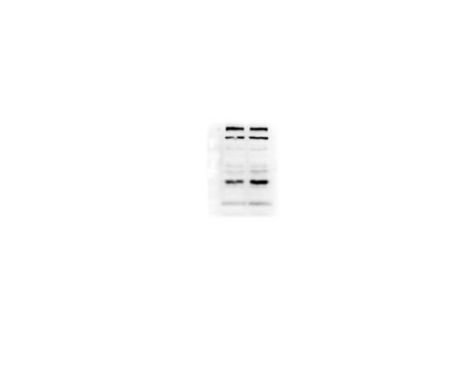

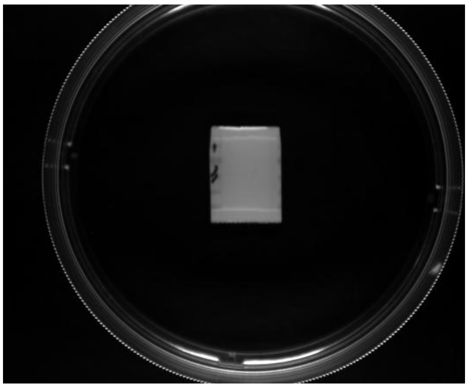

PRDX3 (monomer, SE, erastin, total)

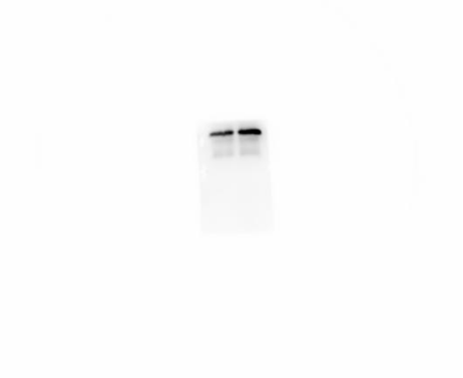

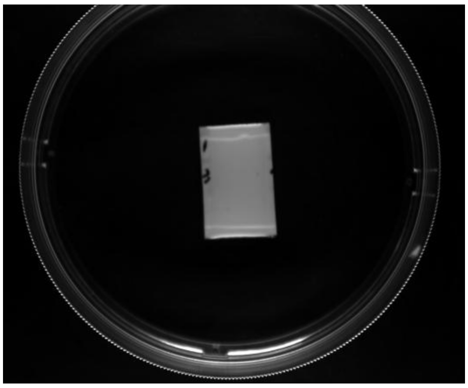

GAPDH (erastin, total)

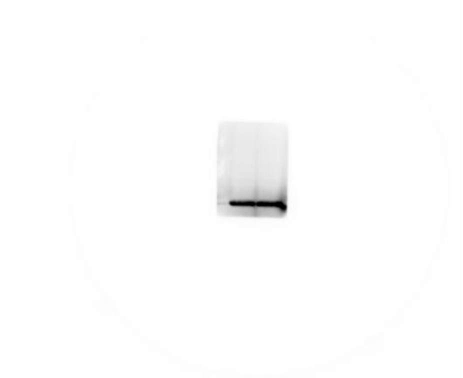

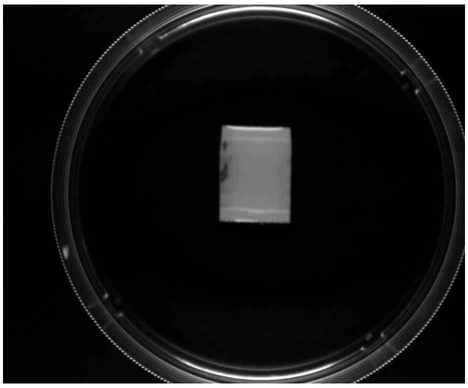

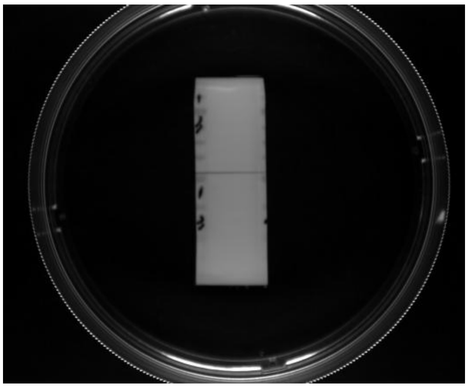

PRDX3 (SE, erastin, cytosol)

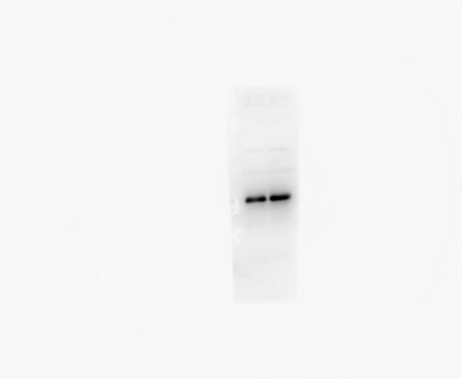

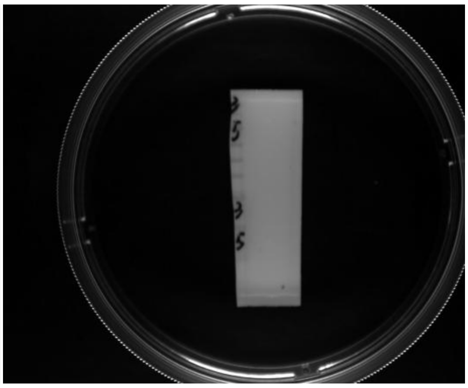

PRDX3 (dimer, LE, erastin, cytosol)

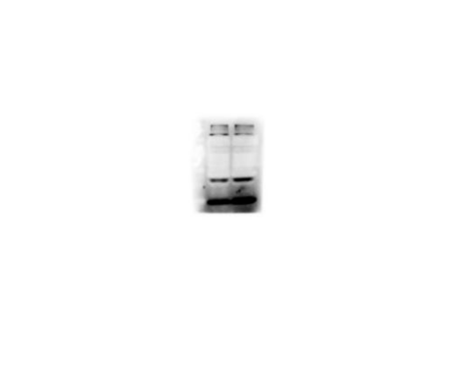

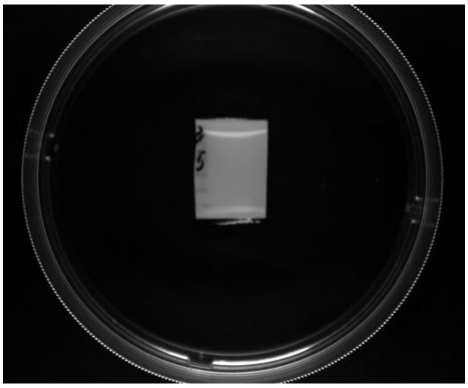

PRDX3 (monomer, SE, erastin, cytosol)

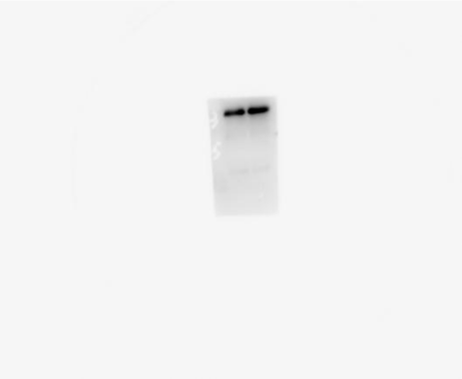

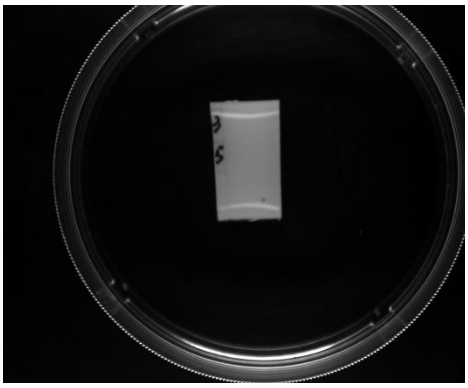

GAPDH (erastin, cytosol)

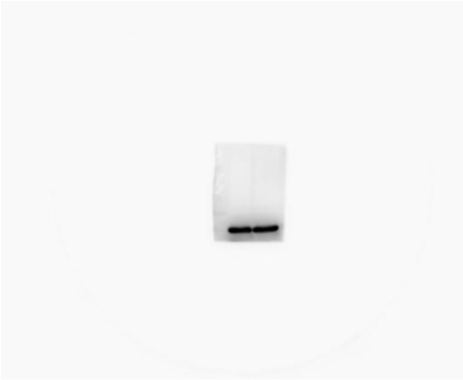

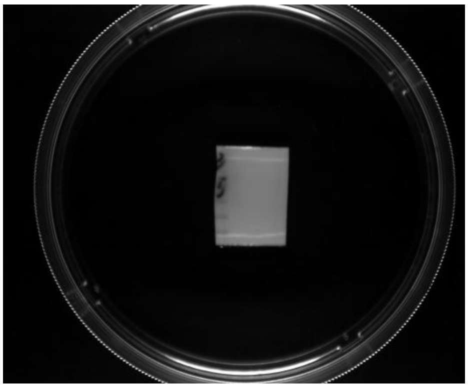

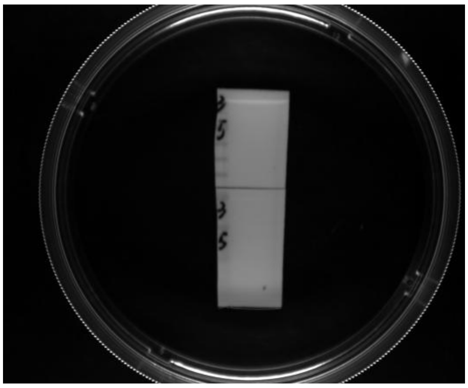

PRDX3 (SE, erastin, plasma membrane)

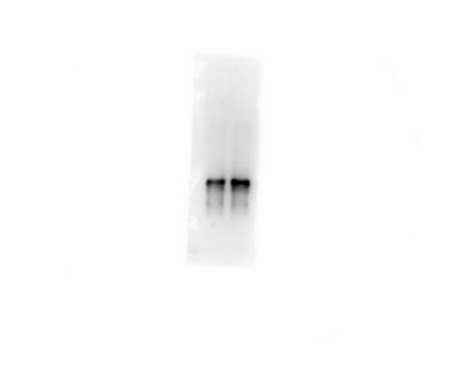

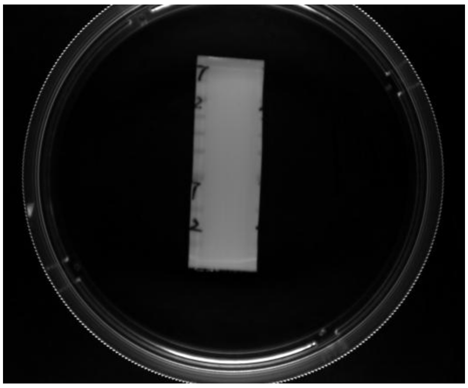

PRDX3 (dimer, LE, erastin, plasma membrane)

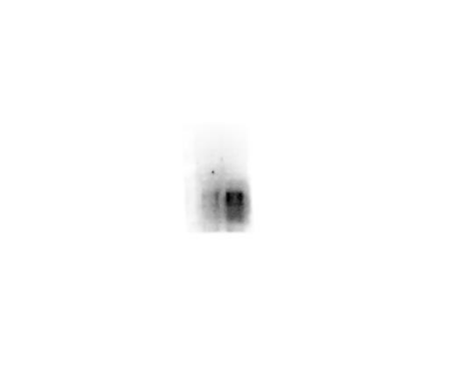

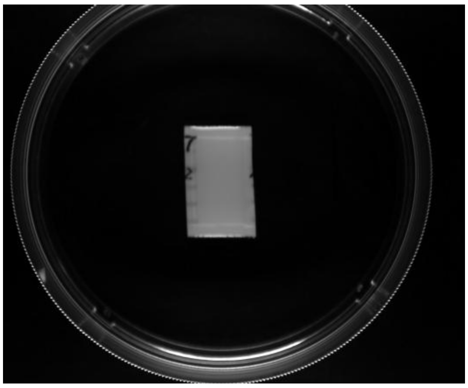

PRDX3 (monomer, SE, erastin, plasma membrane)

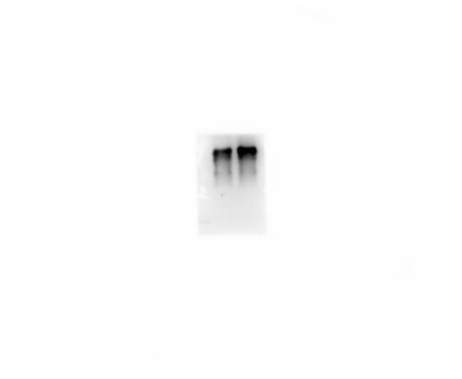

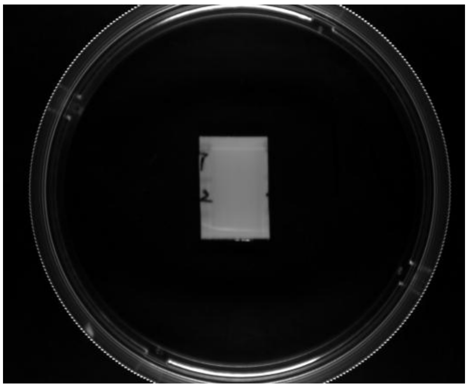

Na^{+}/K^{+} ATPase a1 (erastin, plasma membrane)

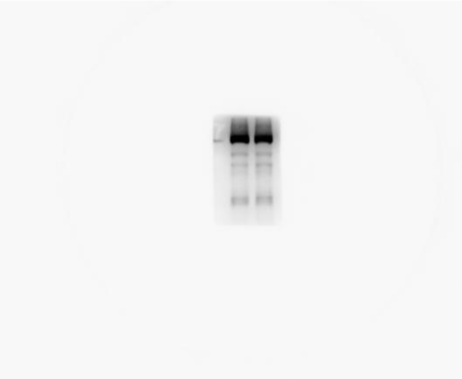

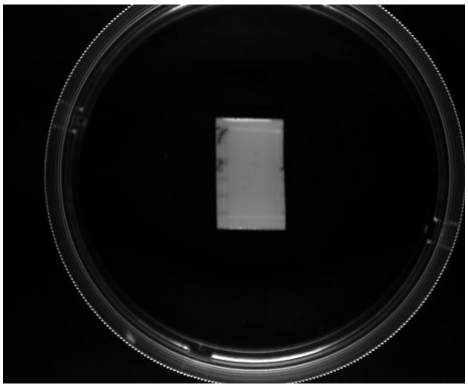

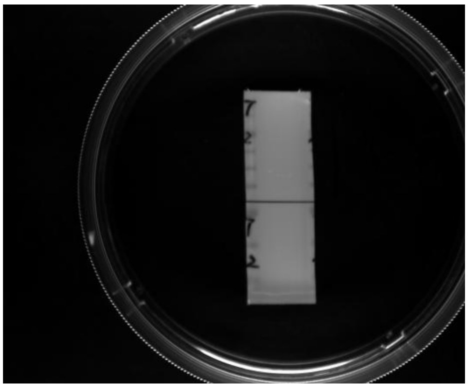

PRDX3 (SE, erastin, organelle)

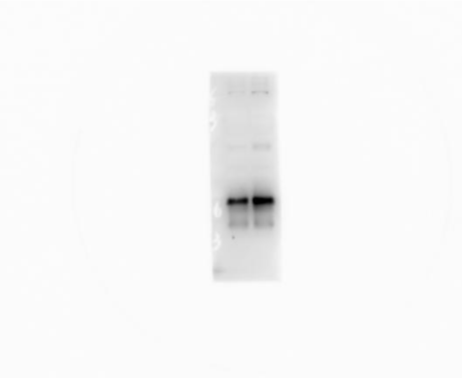

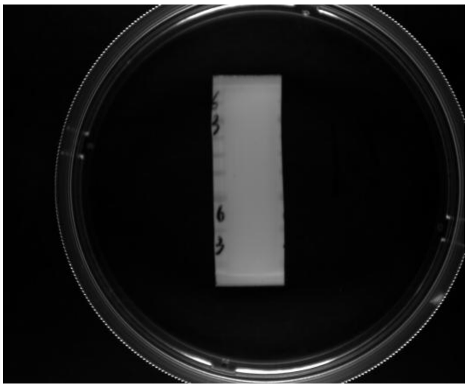

PRDX3 (dimer, LE, erastin, organelle)

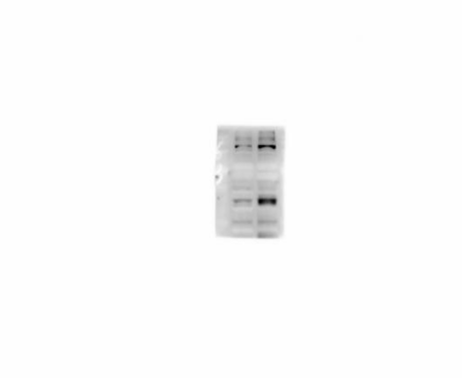

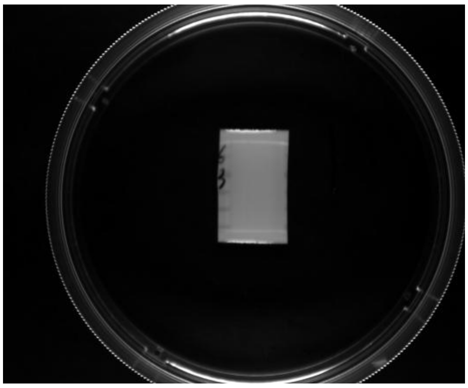

PRDX3 (monomer, SE, erastin, organelle)

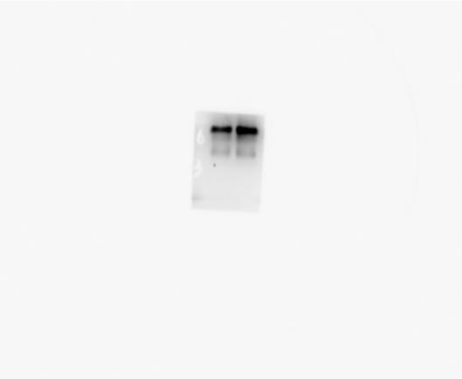

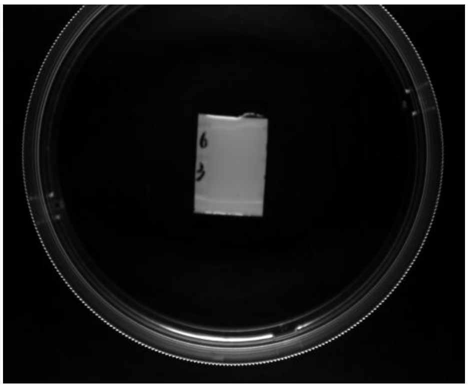

COX IV (erastin, organelle)

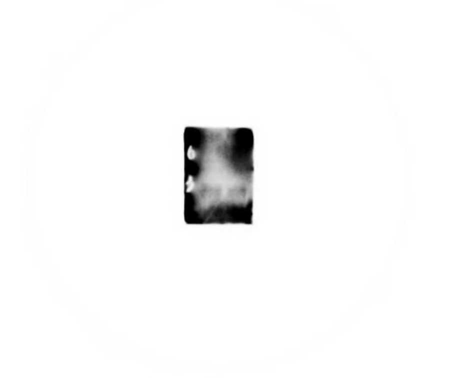

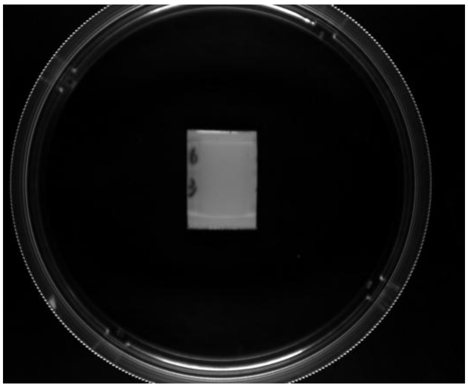

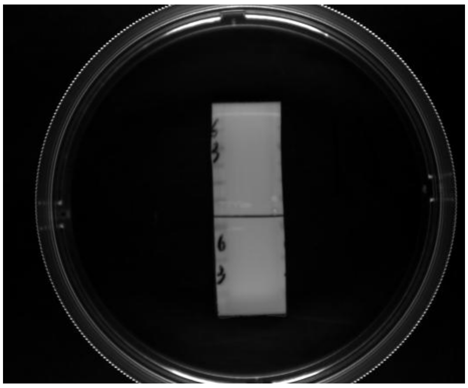

H446DDP

PRDX3 (SE, untreated, total)

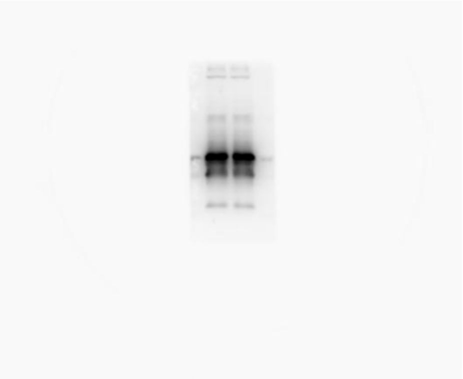
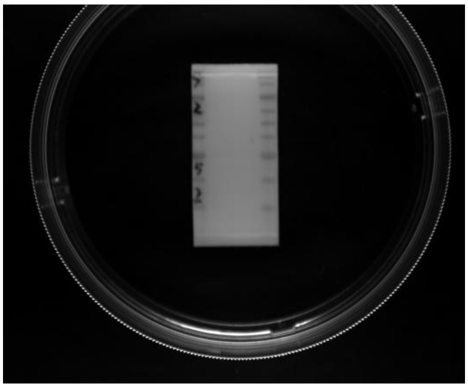

PRDX3 (dimer, LE, untreated, total)

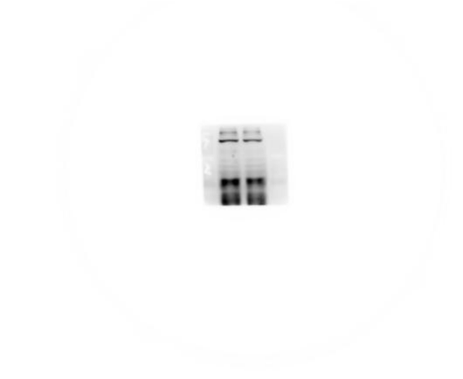
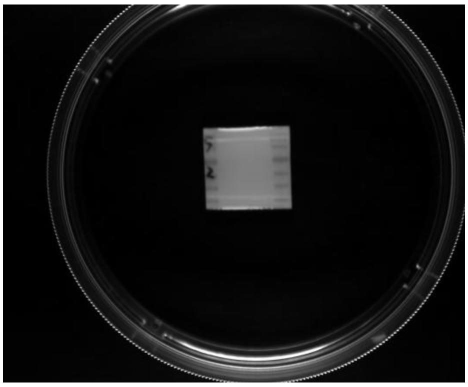

PRDX3 (monomer, SE, untreated, total)

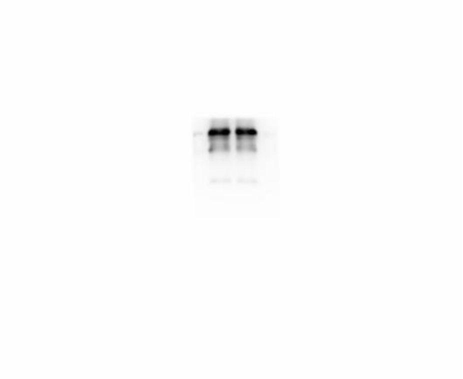
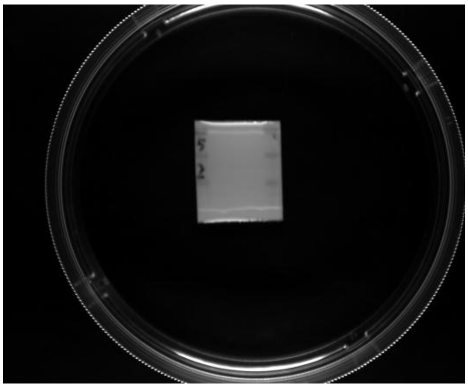

GAPDH (untreated, total)

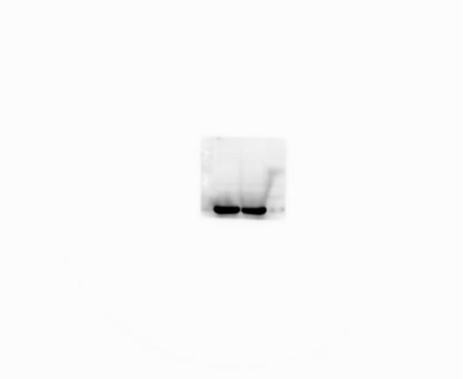
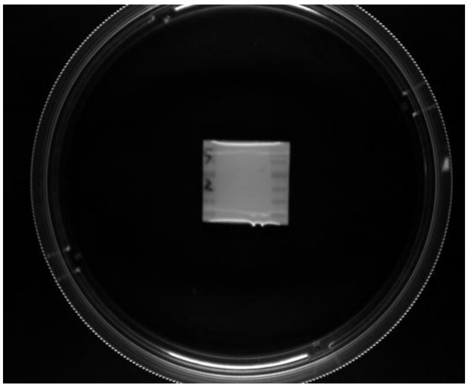

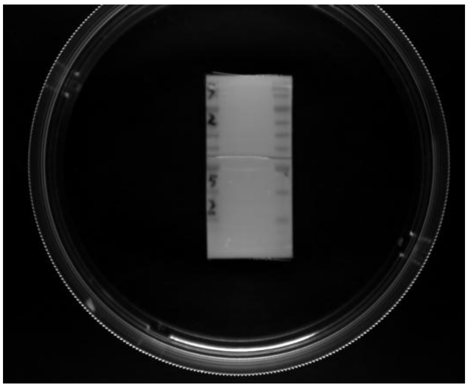

PRDX3 (SE, untreated, cytosol)

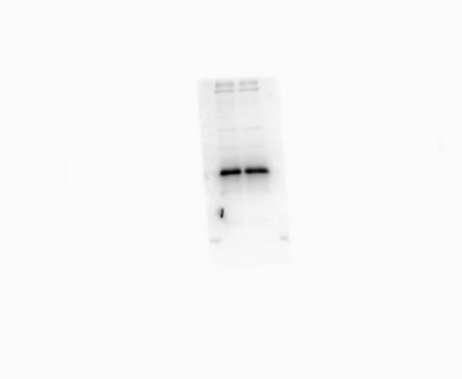 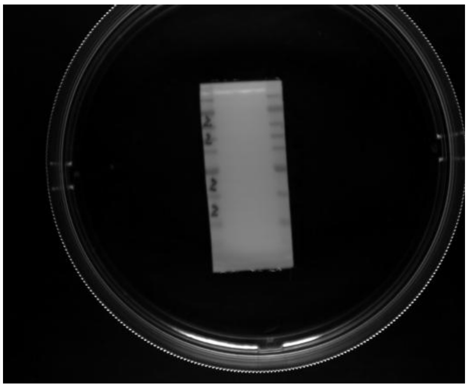

PRDX3 (dimer, LE, untreated, cytosol)

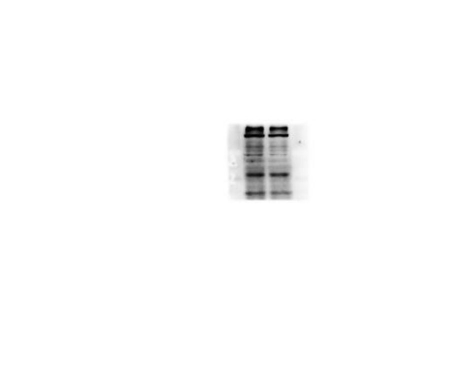 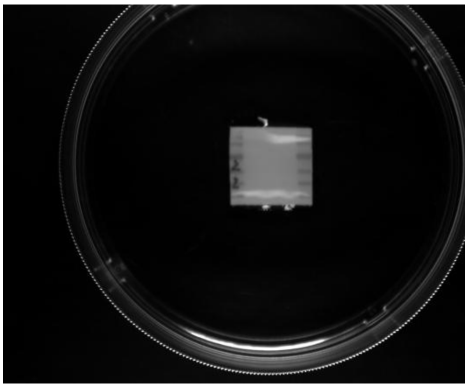

PRDX3 (monomer, SE, untreated, cytosol)

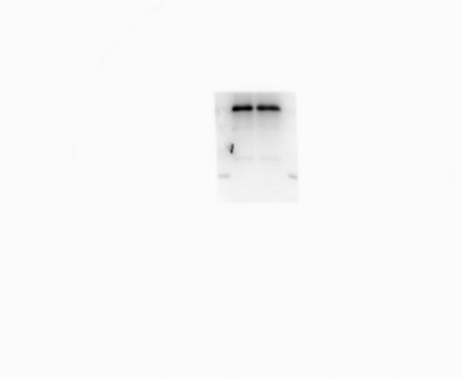 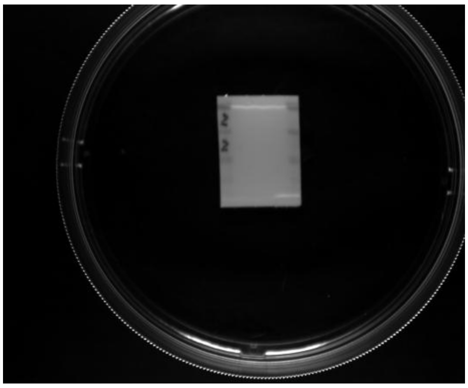

GAPDH (untreated, cytosol)

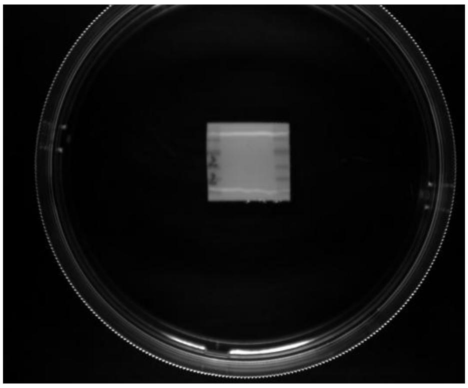

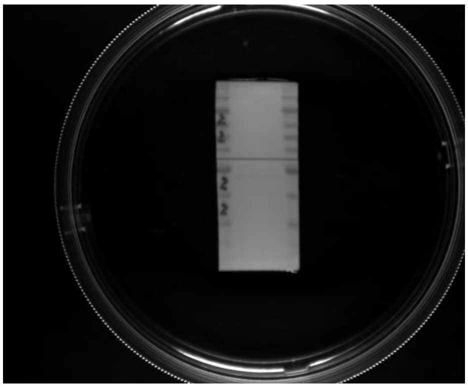

PRDX3 (SE, untreated, plasma membrane)

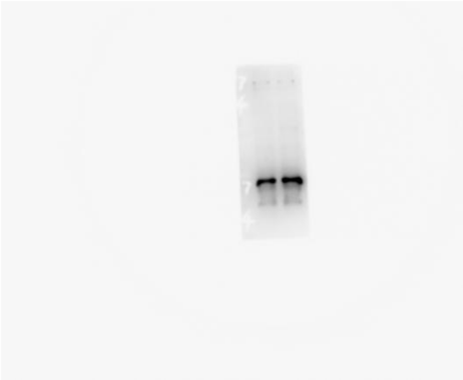

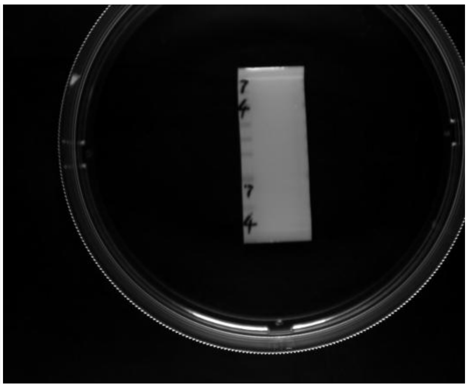

PRDX3 (dimer, LE, untreated, plasma membrane)

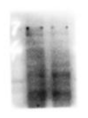

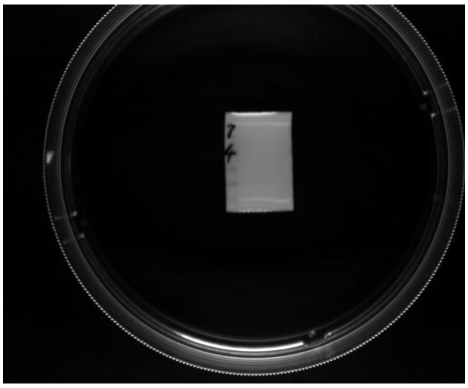

PRDX3 (monomer, SE, untreated, plasma membrane)

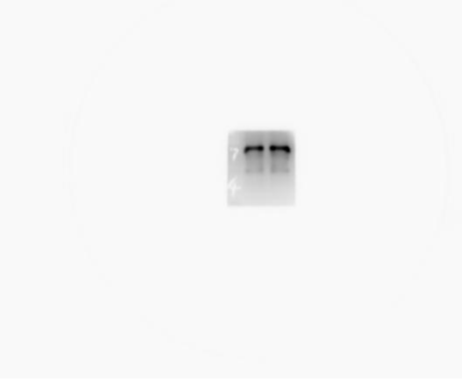
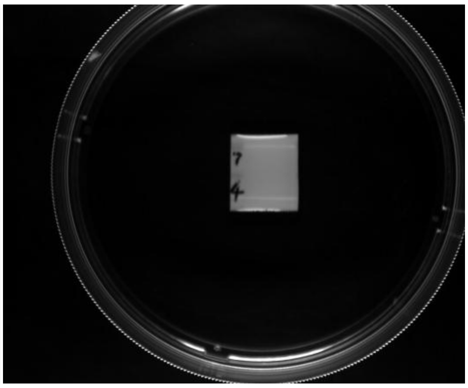

Na^+/K^+ ATPase a1 (untreated, plasma membrane)

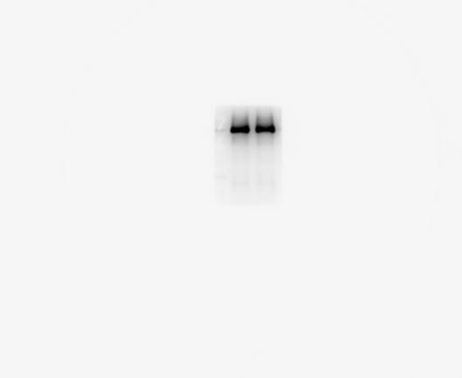
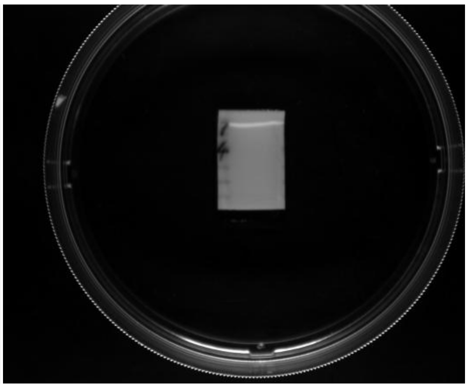

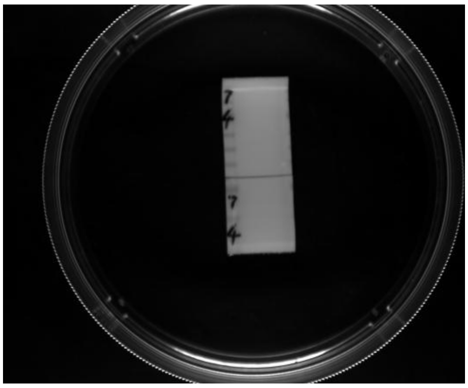

PRDX3 (SE, untreated, organelle)

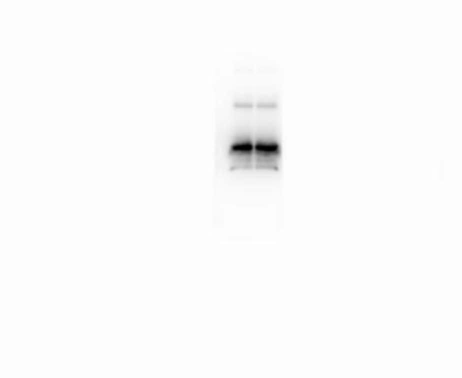
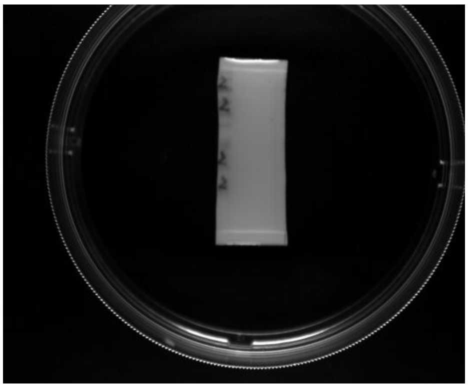

PRDX3 (dimer, LE, untreated, organelle)

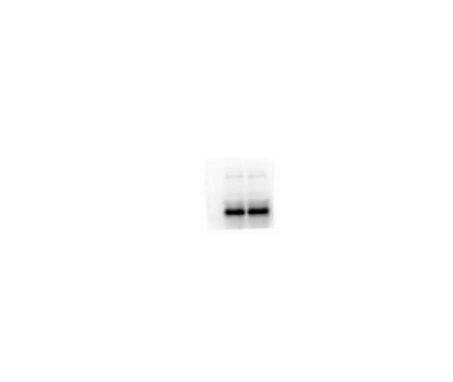

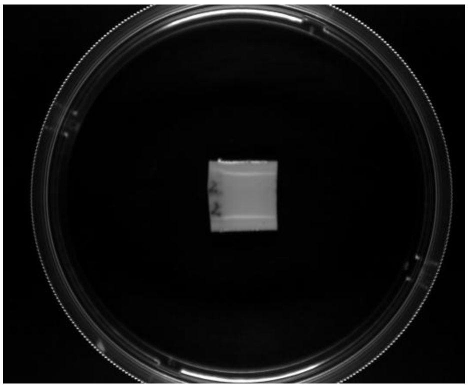

PRDX3 (monomer, SE, untreated, organelle)

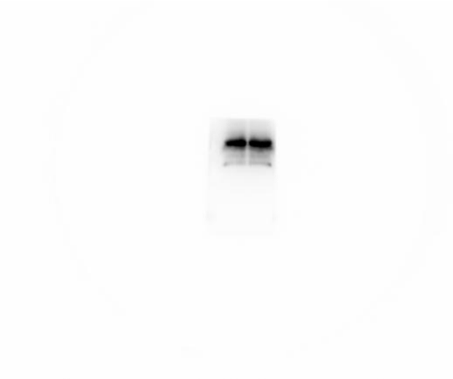

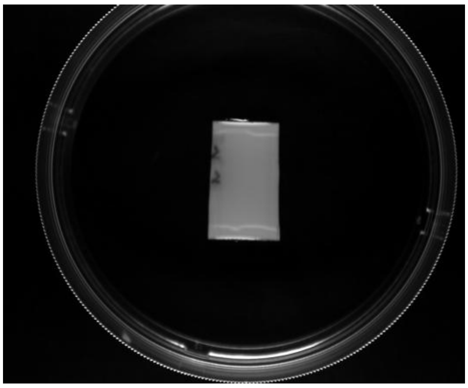

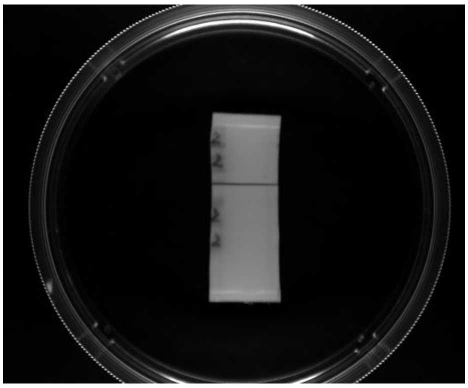

COX IV (untreated, organelle)

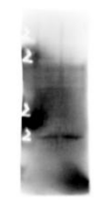

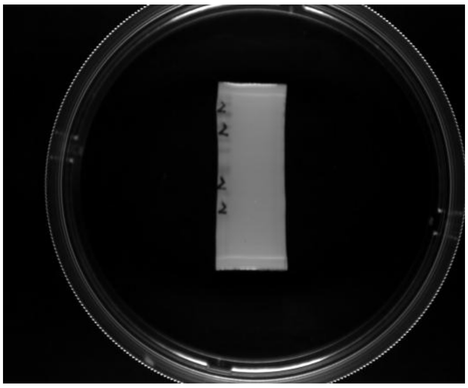

PRDX3 (SE, cisplatin, total)

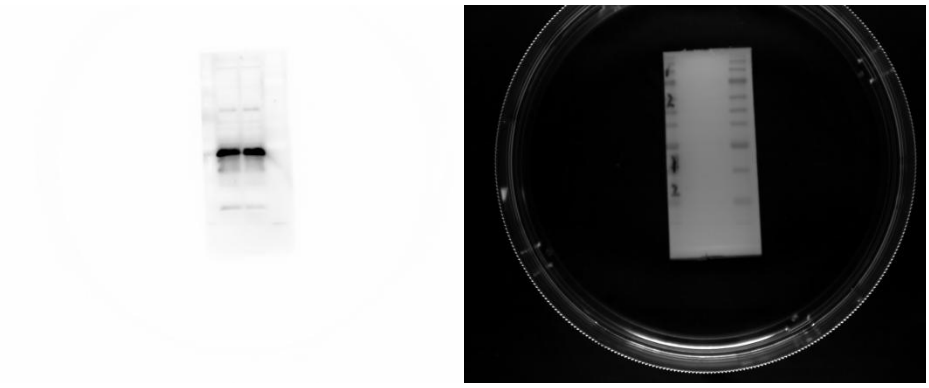

PRDX3 (dimer, LE, cisplatin, total)

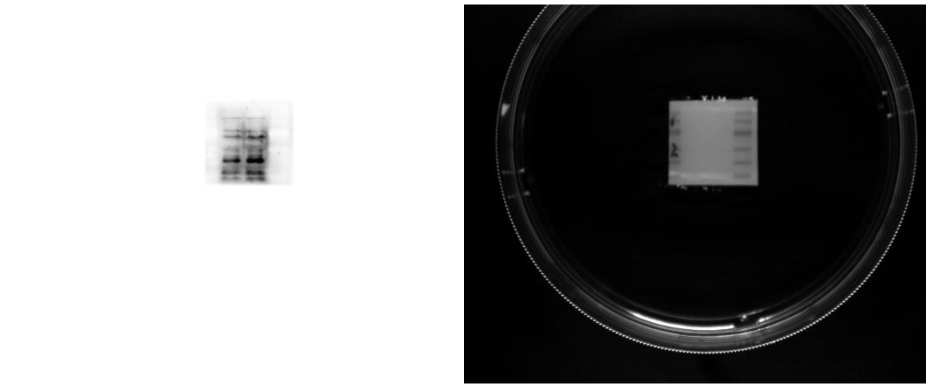

PRDX3 (monomer, SE, cisplatin, total)

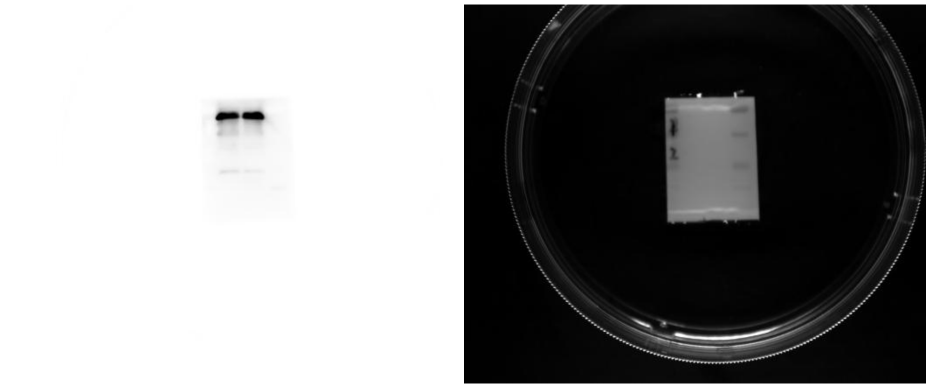

GAPDH (cisplatin, total)

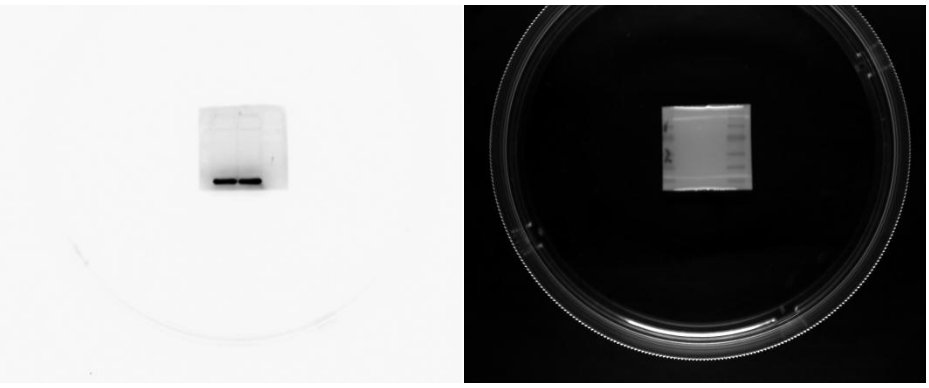

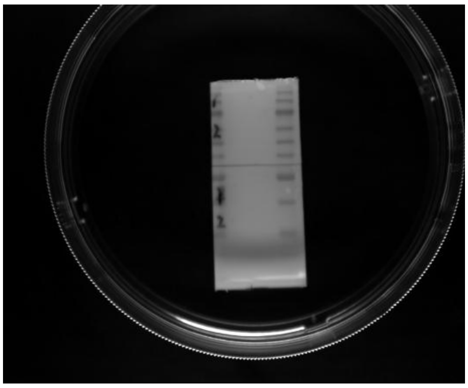

PRDX3 (SE, cisplatin, cytosol)

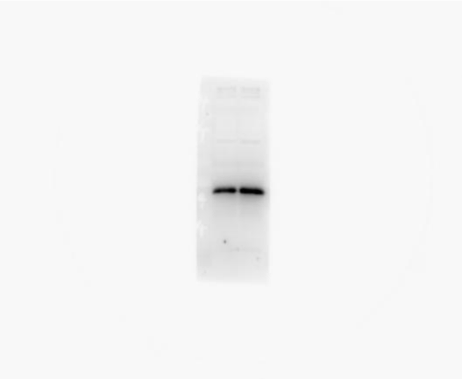

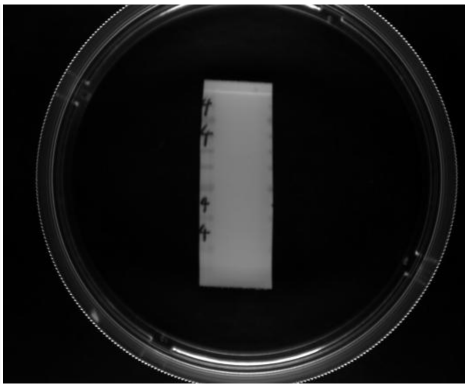

PRDX3 (dimer, LE, cisplatin, cytosol)

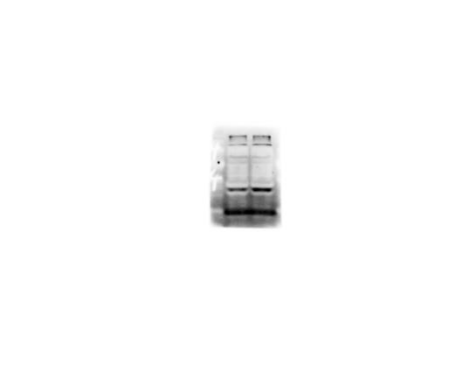

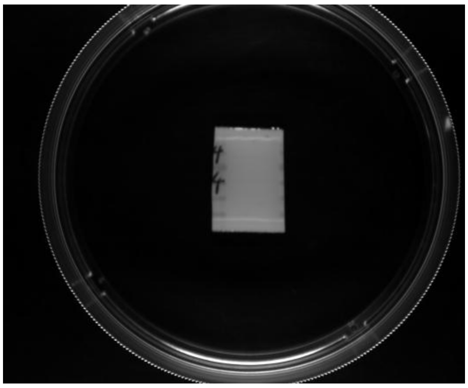

PRDX3 (monomer, SE, cisplatin, cytosol)

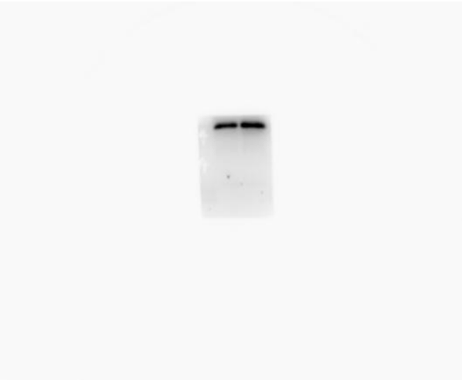

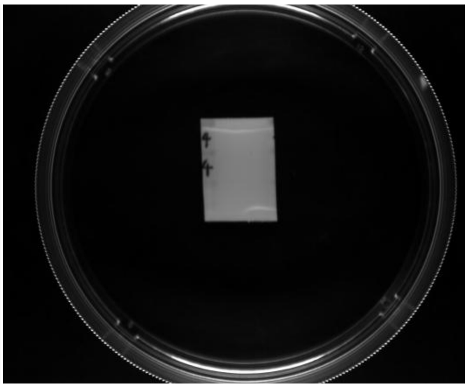

GAPDH (cisplatin, cytosol)

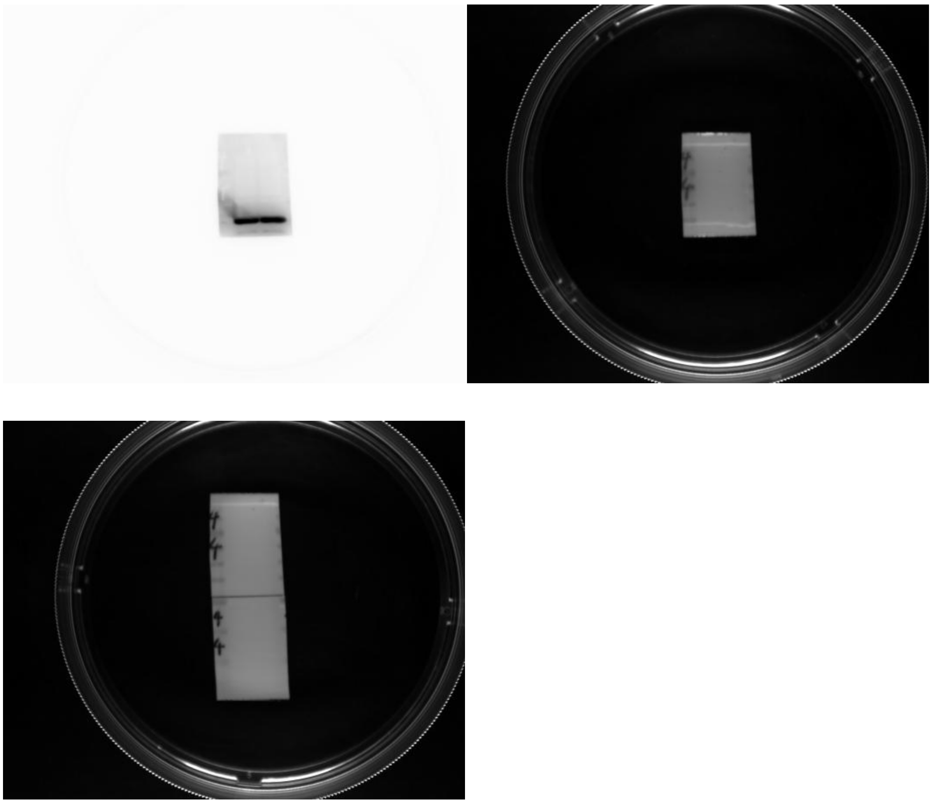

PRDX3 (SE, cisplatin, plasma membrane)

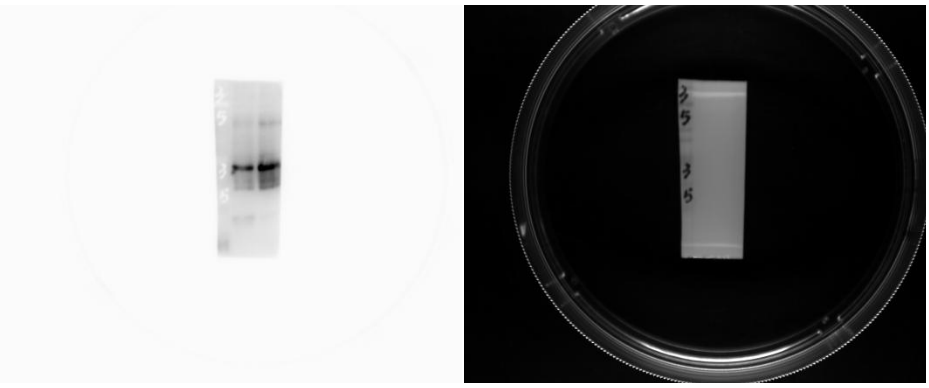

PRDX3 (dimer, LE, cisplatin, plasma membrane)

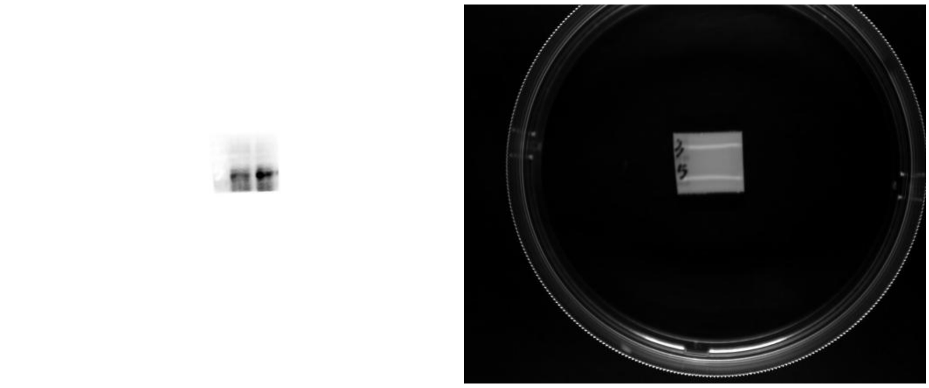

PRDX3 (monomer, SE, cisplatin, plasma membrane)

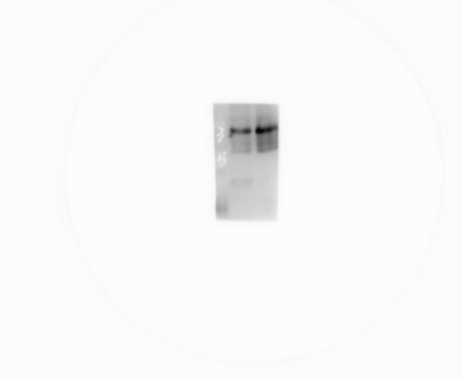

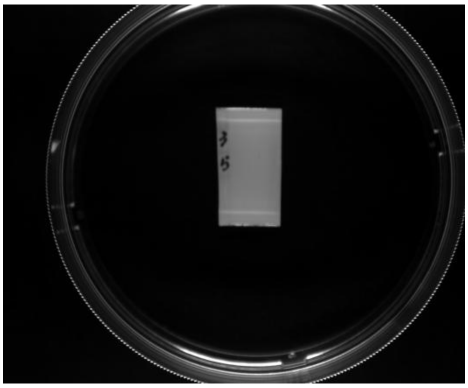

Na^+/K^+ ATPase a1 (cisplatin, plasma membrane)

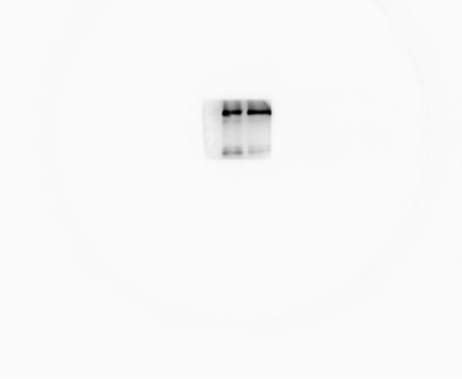

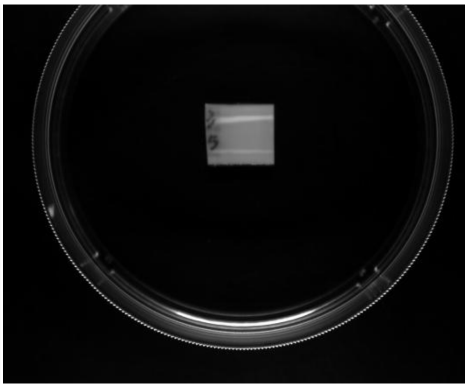

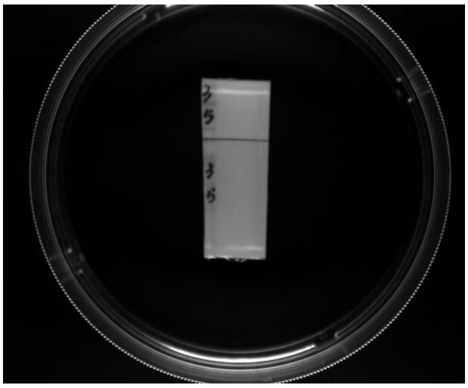

PRDX3 (SE, cisplatin, organelle)

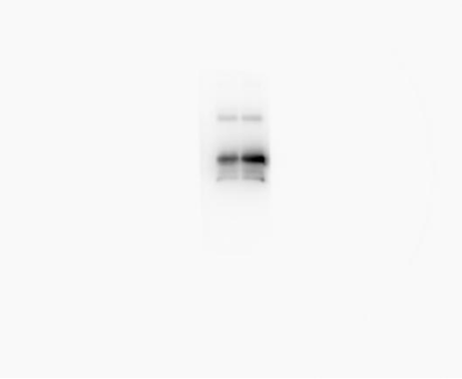

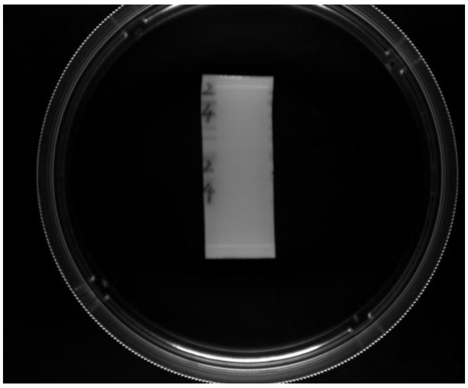

PRDX3 (dimer, LE, cisplatin, organelle)

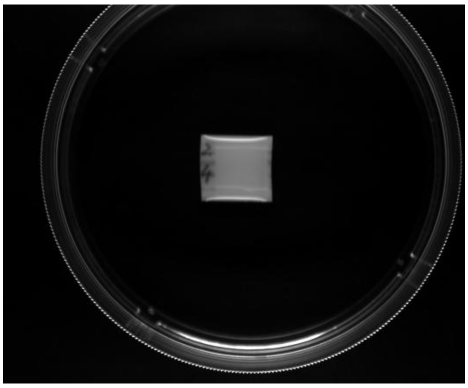

PRDX3 (monomer, SE, cisplatin, organelle)

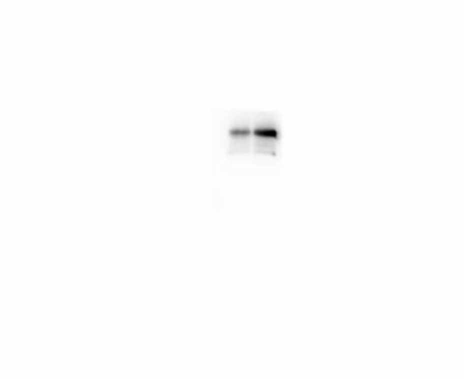

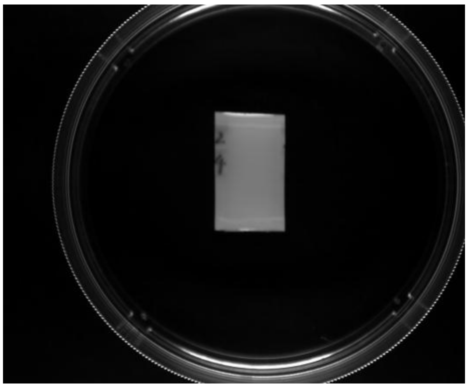

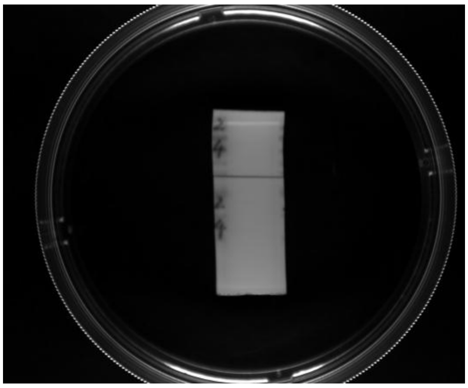

COX IV (cisplatin, organelle)

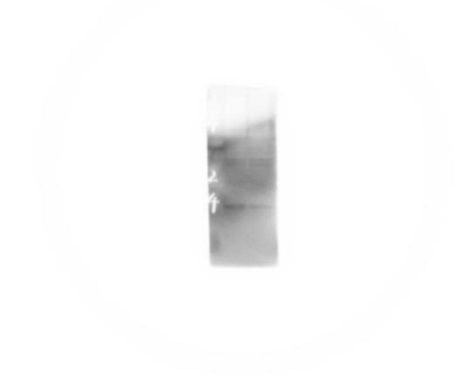

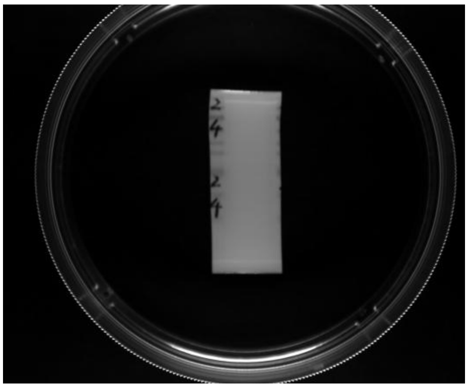

PRDX3 (SE, erastin, total)

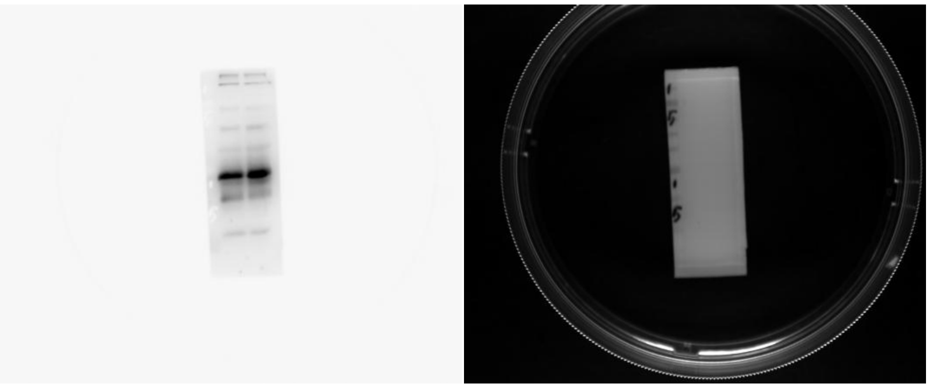

PRDX3 (dimer, LE, erastin, total)

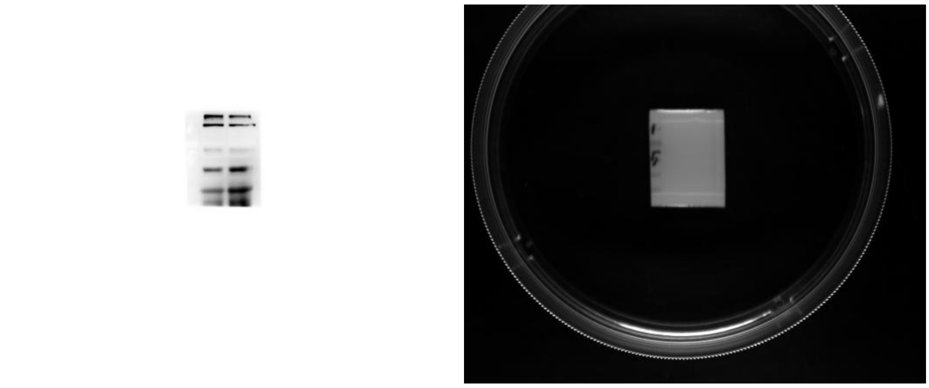

PRDX3 (monomer, SE, erastin, total)

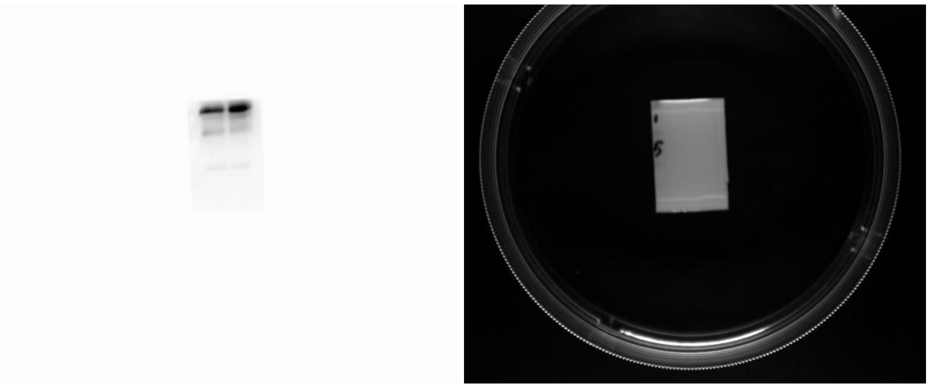

GAPDH (erastin, total)

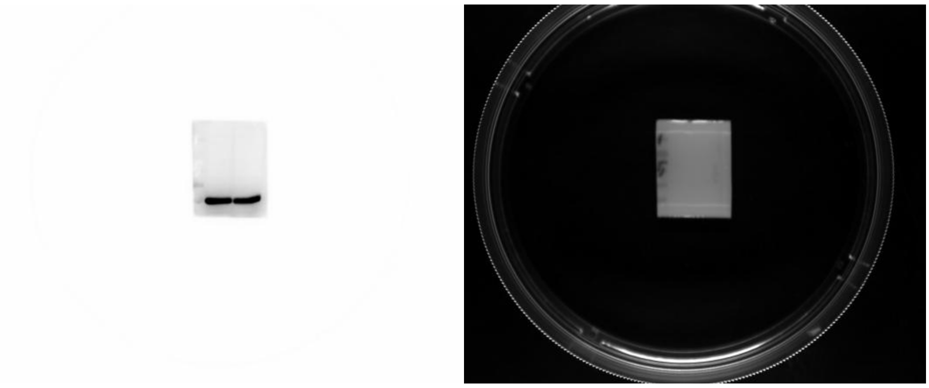

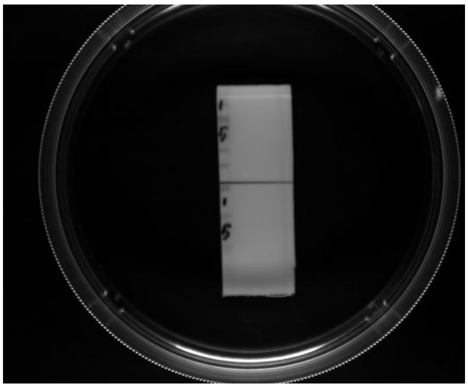

PRDX3 (SE, erastin, cytosol)

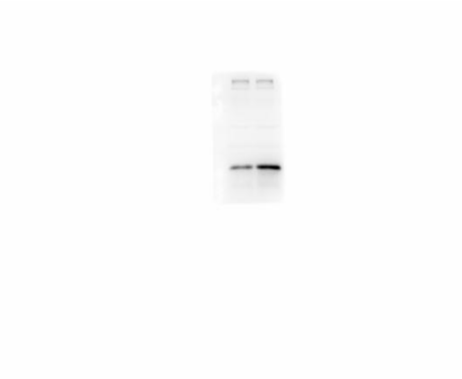

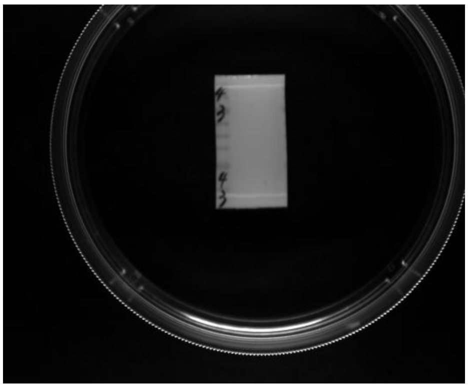

PRDX3 (dimer, LE, erastin, cytosol)

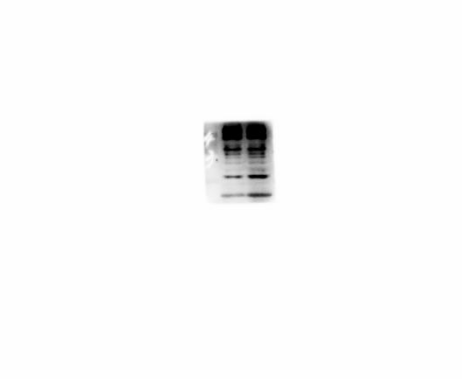

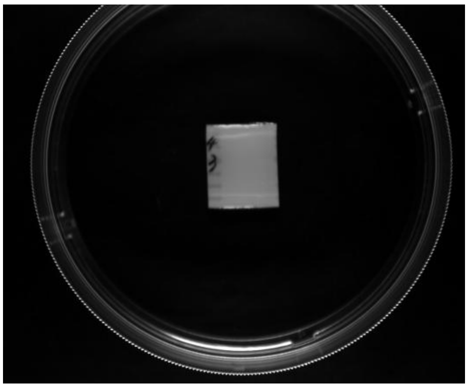

PRDX3 (monomer, SE, erastin, cytosol)

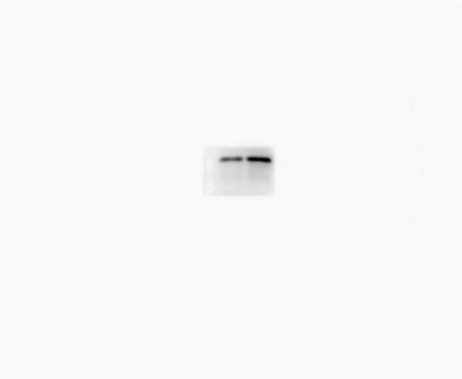

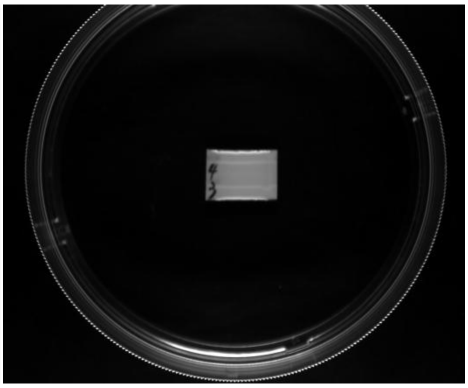

GAPDH (erastin, cytosol)

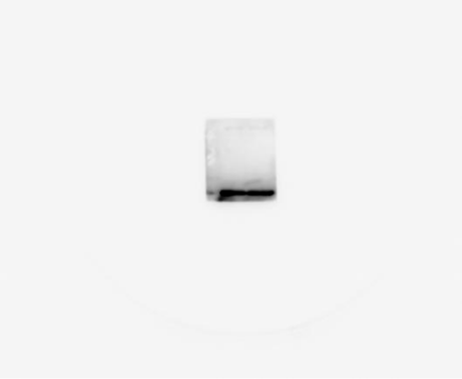

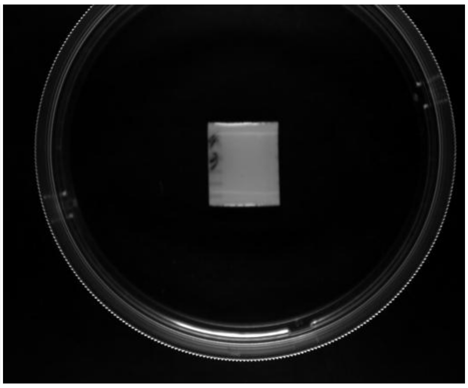

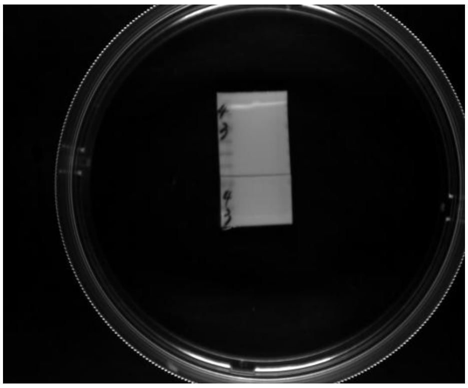

PRDX3 (SE, erastin, plasma membrane)

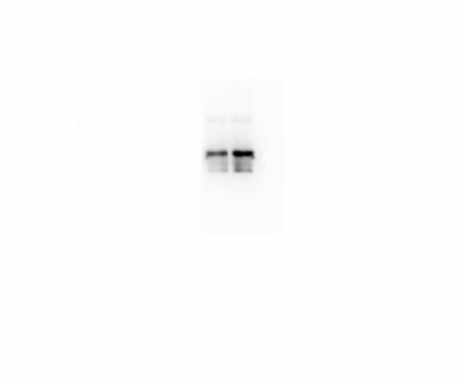

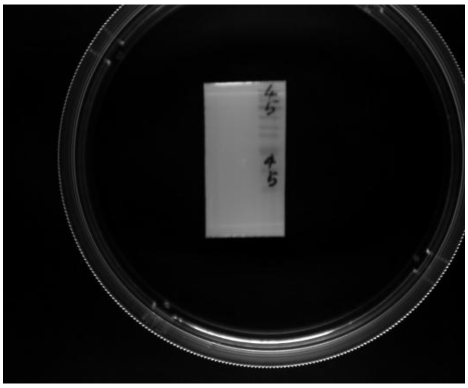

PRDX3 (dimer, LE, erastin, plasma membrane)

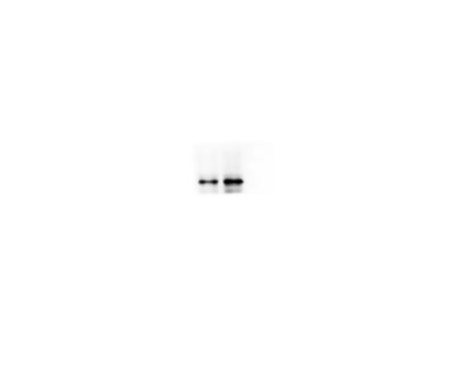

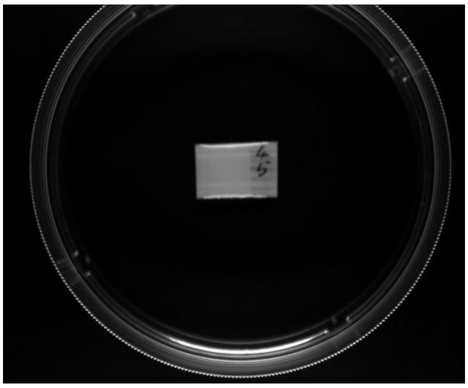

PRDX3 (monomer, SE, erastin, plasma membrane)

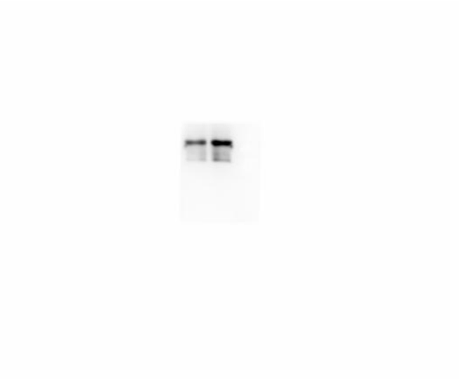

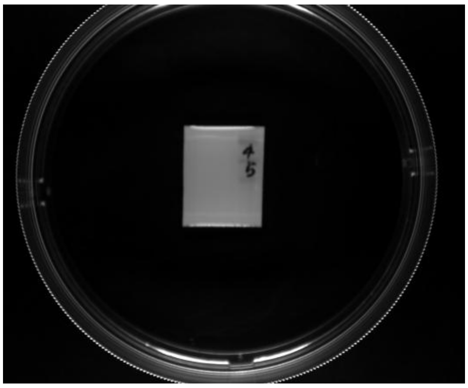

Na$^+$/K$^+$ ATPase a1 (erastin, plasma membrane)

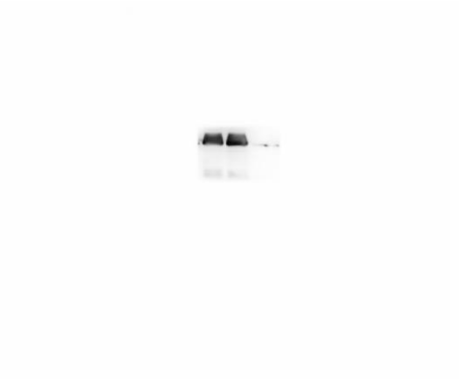

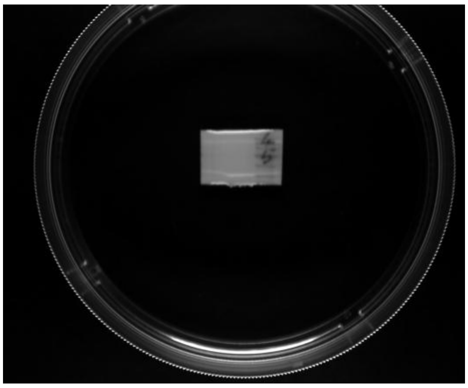

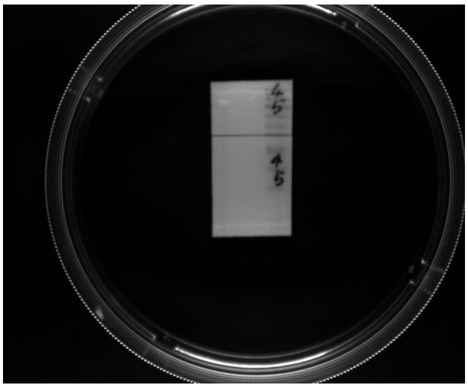

PRDX3 (SE, erastin, organelle)

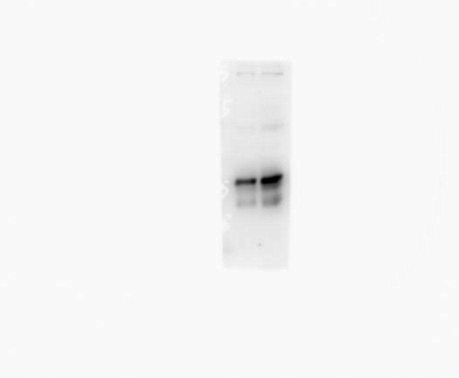

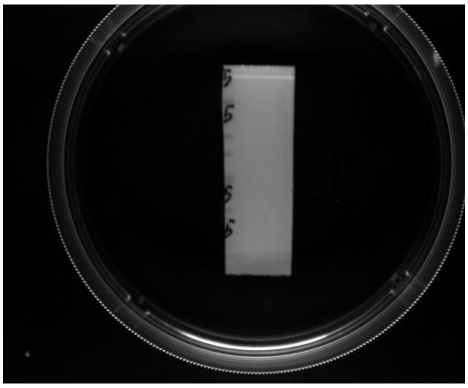

PRDX3 (dimer, LE, erastin, organelle)

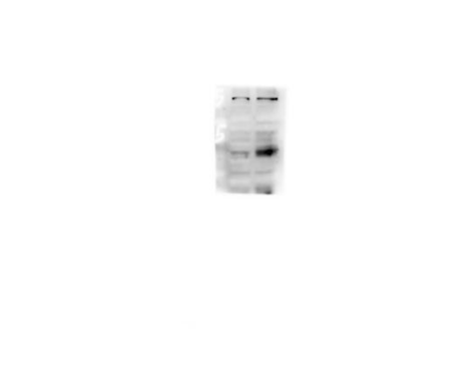

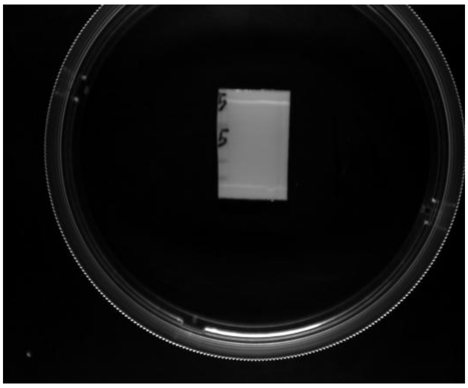

PRDX3 (monomer, SE, erastin, organelle)

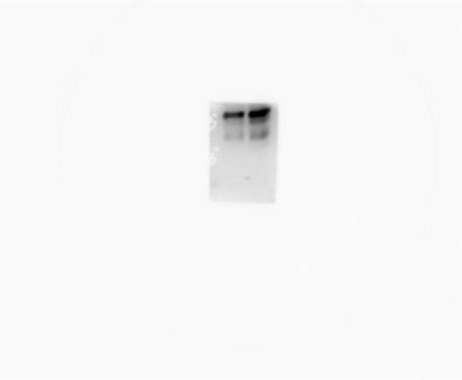

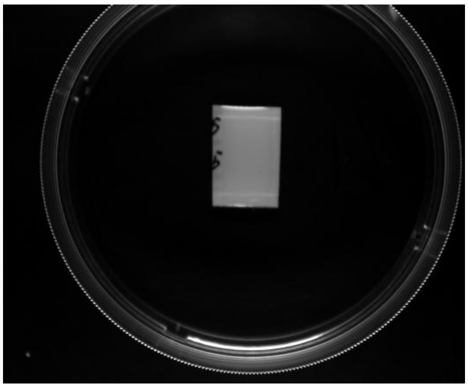

COX IV (erastin, organelle)

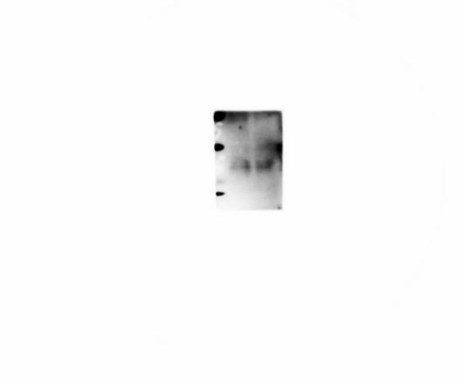

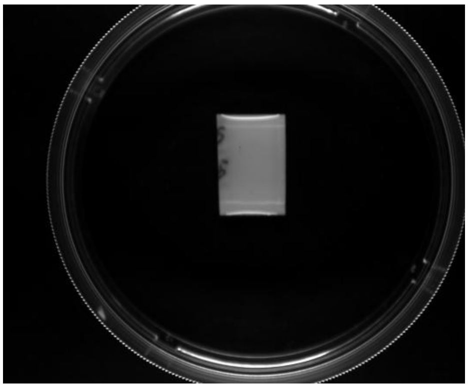

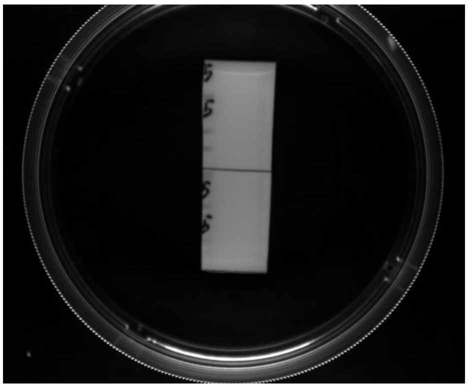

Fig. 8E First Repetition

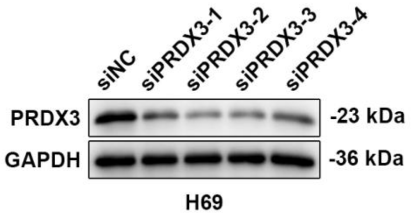

H69

PRDX3

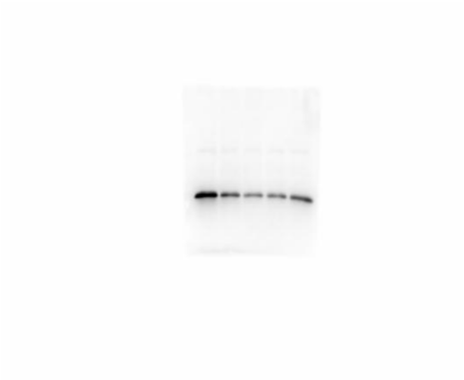

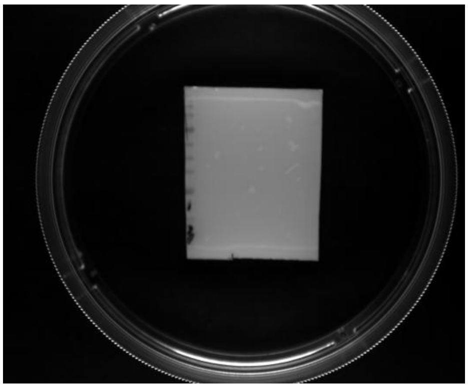

GAPDH

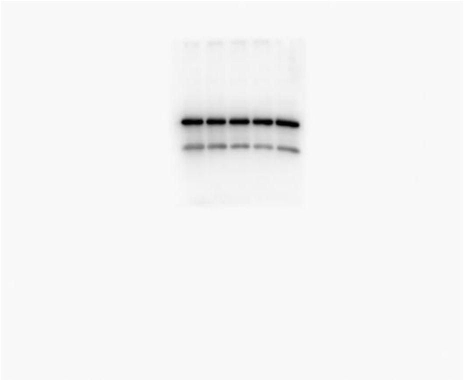

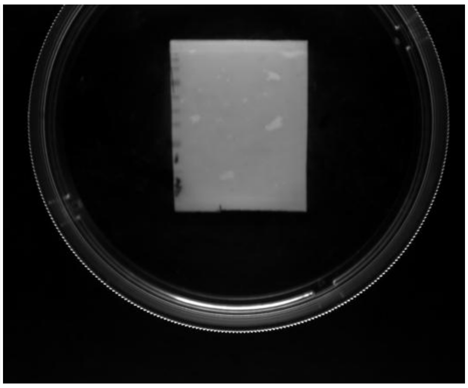

Fig. 8E Second Repetition

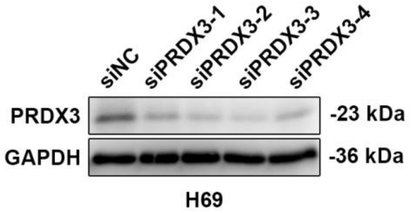

H69

PRDX3

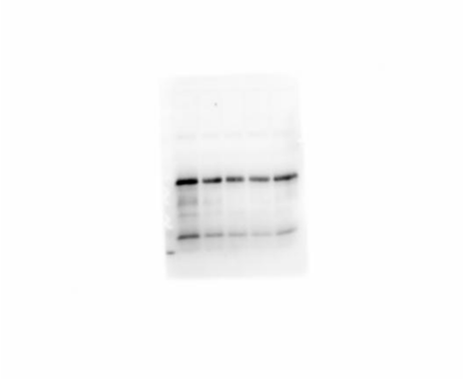

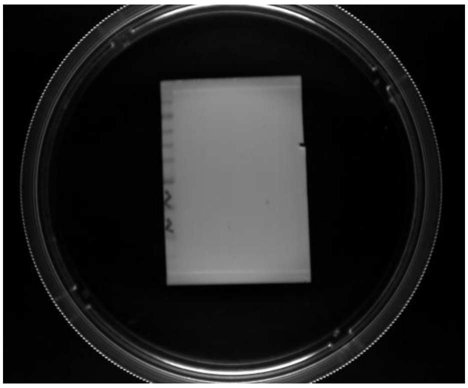

GAPDH

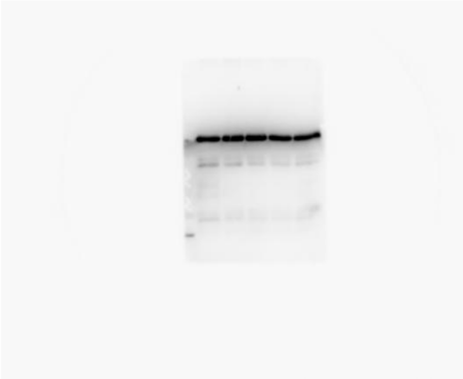

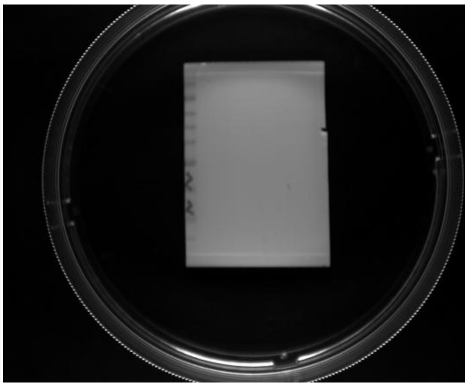

Fig. 8E Third Repetition

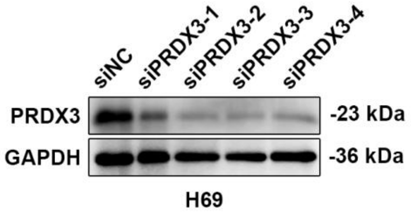

H69

PRDX3

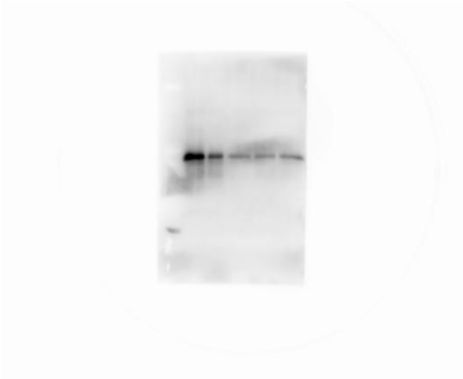

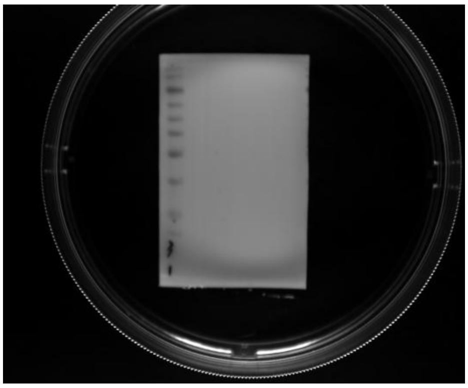

GAPDH

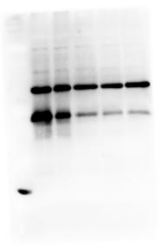

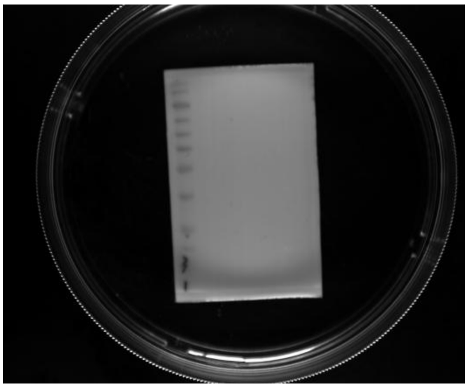

Fig. 8F First Repetition

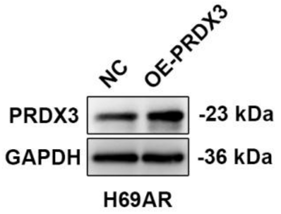

H69AR

PRDX3

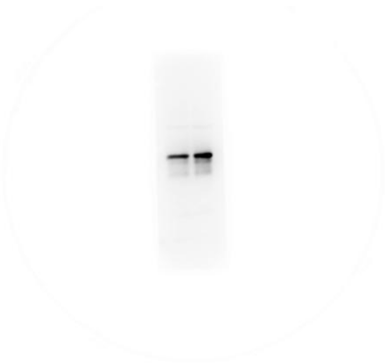

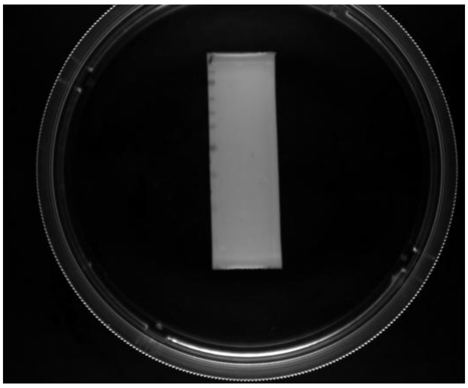

GAPDH

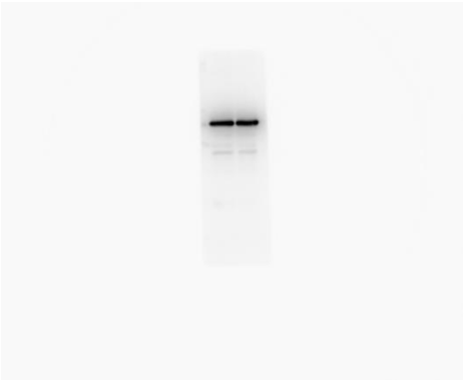

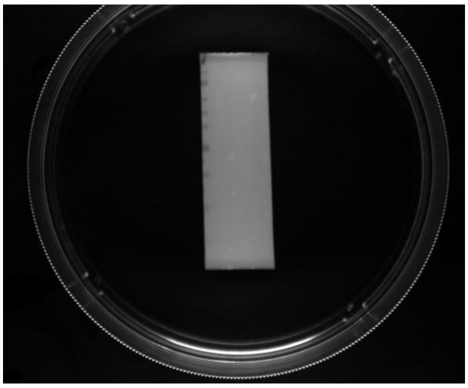

Fig. 8F Second Repetition

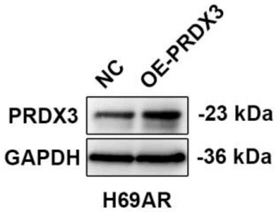

H69AR

PRDX3

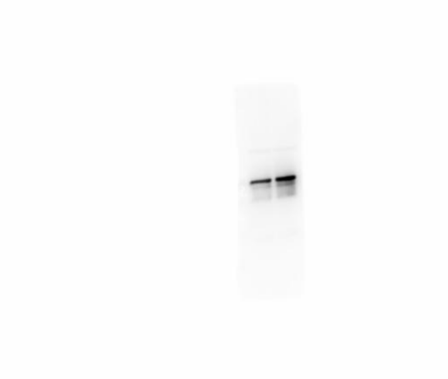

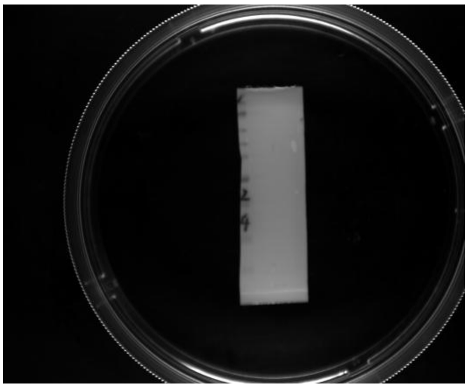

GAPDH

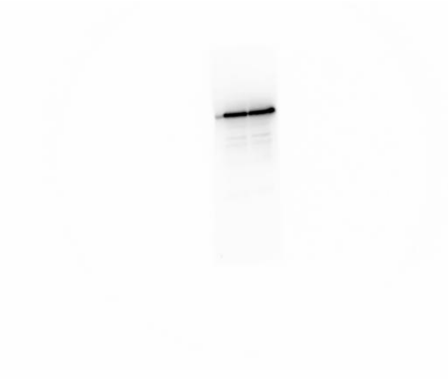

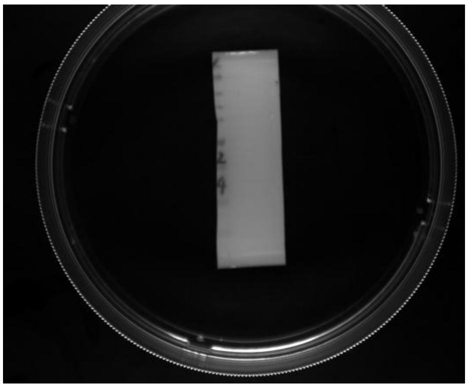

Fig. 8F Third Repetition

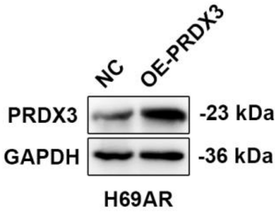

H69AR

PRDX3

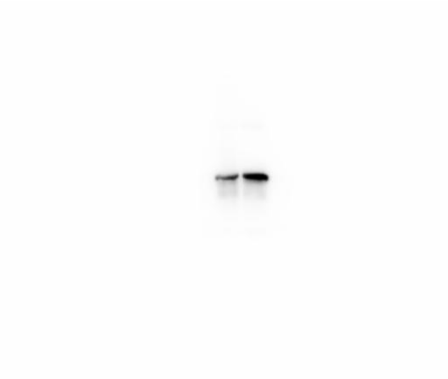

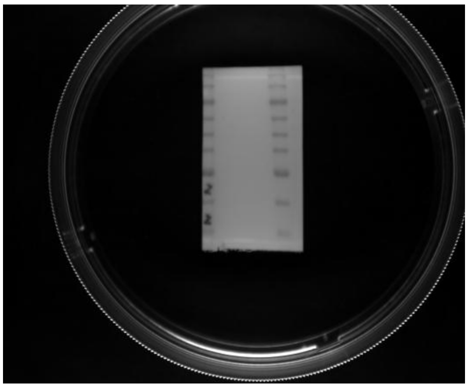

GAPDH

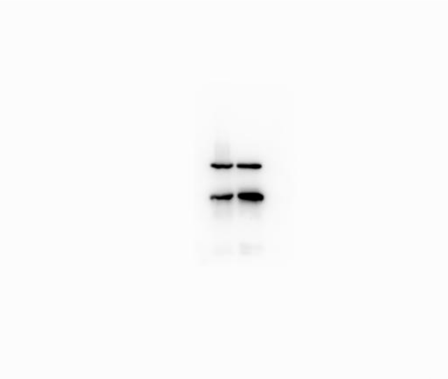

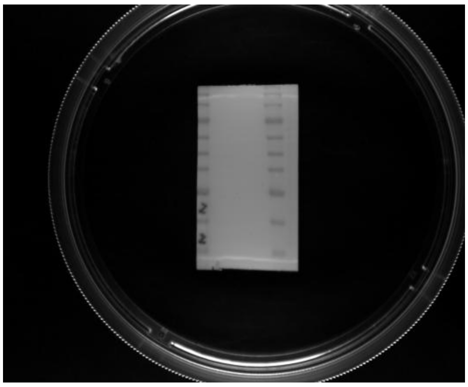

Fig. 8G First Repetition

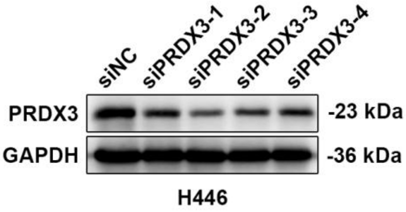

H446

PRDX3

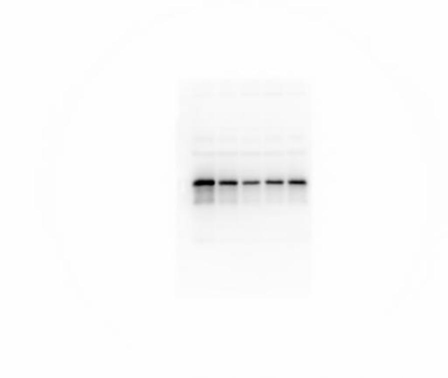

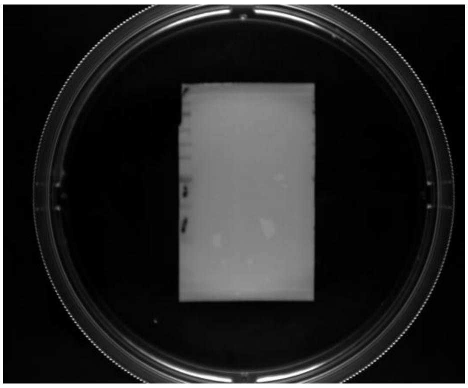

GAPDH

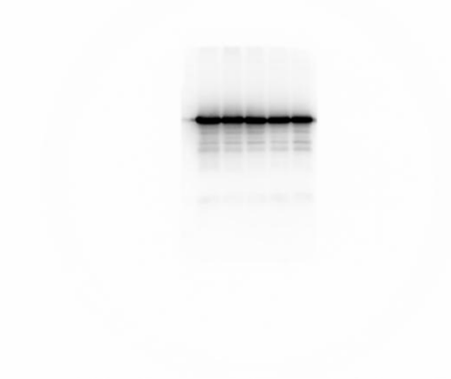

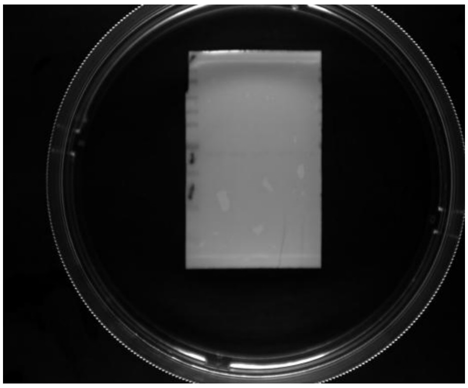

Fig. 8G Second Repetition

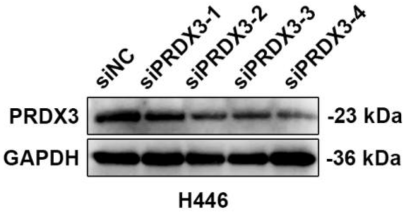

H446

PRDX3

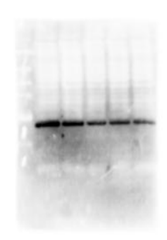

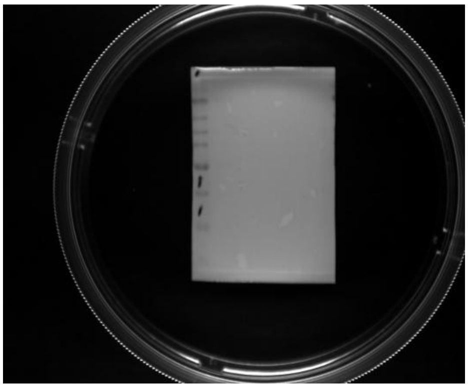

GAPDH

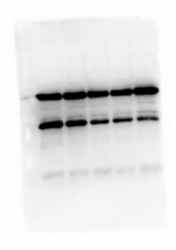

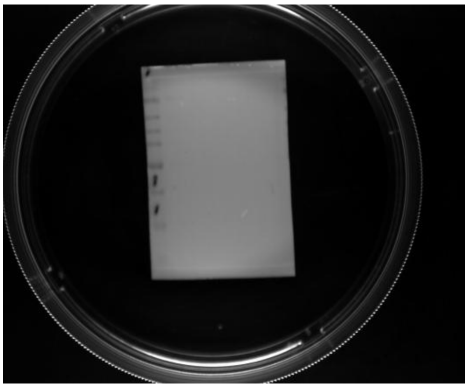

Fig. 8G Third Repetition

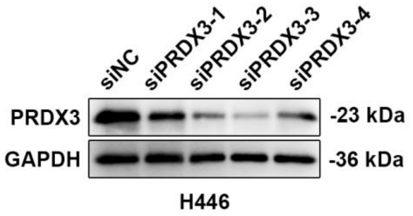

H446

PRDX3

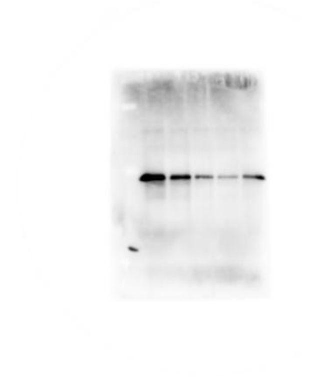

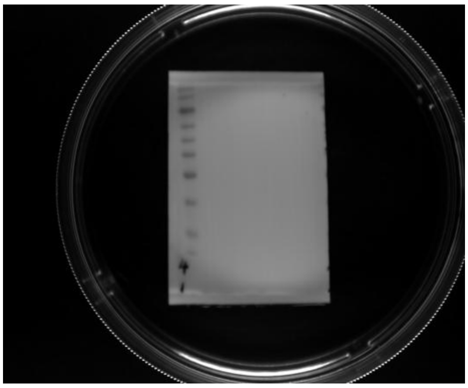

GAPDH

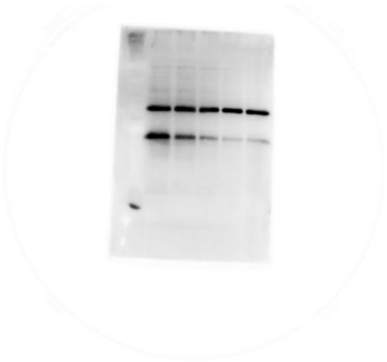

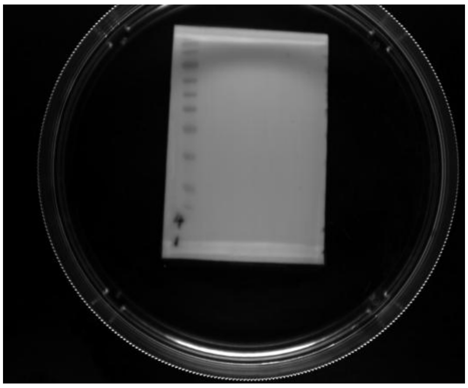

Fig. 8H First Repetition

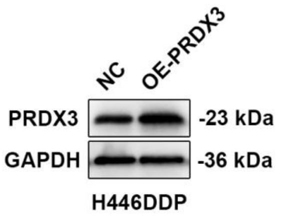

H446DDP

PRDX3

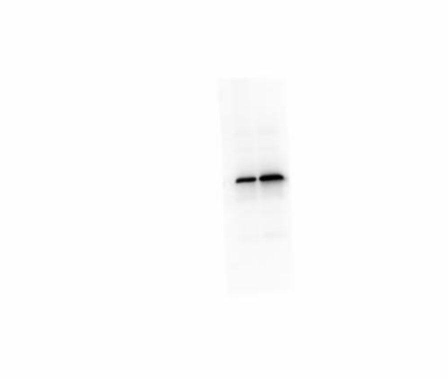

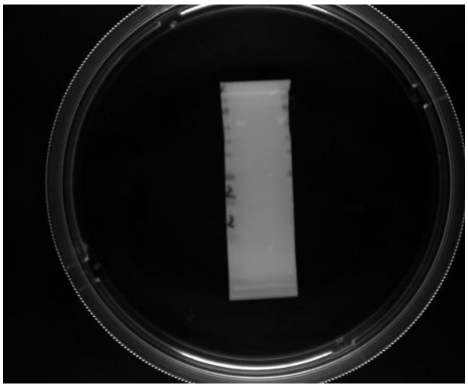

GAPDH

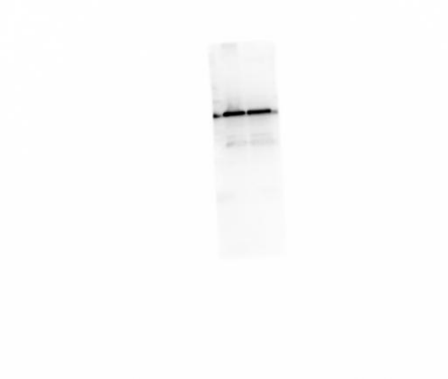

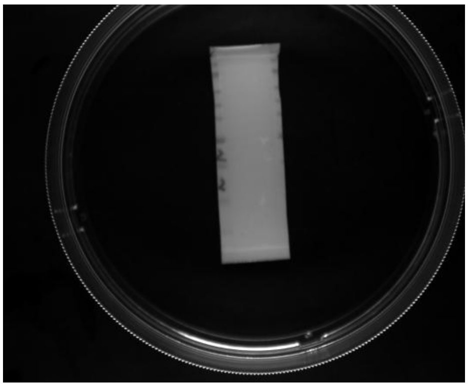

Fig. 8H Second Repetition

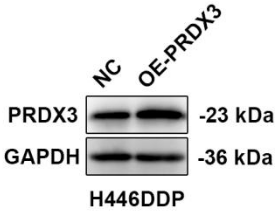

H446DDP

PRDX3

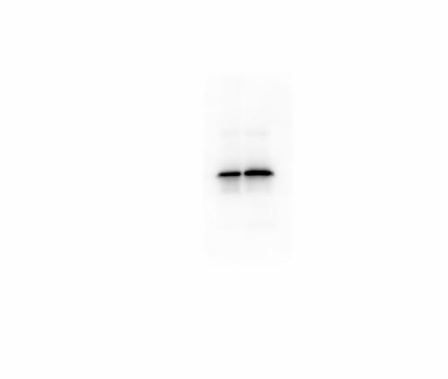

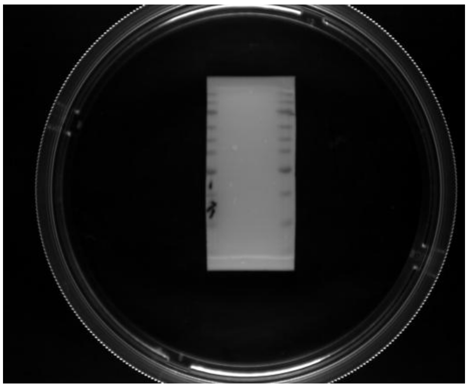

GAPDH

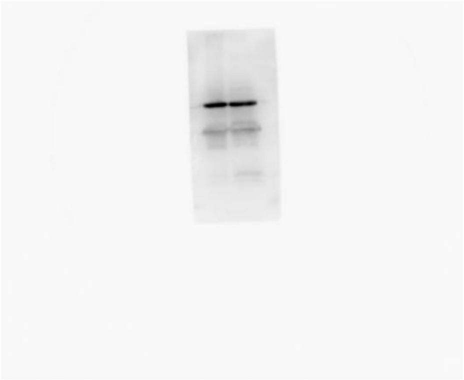
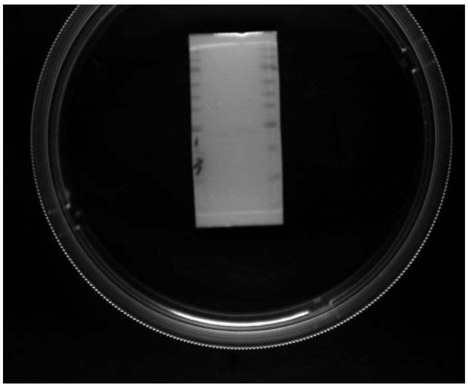

Fig. 8H Third Repetition

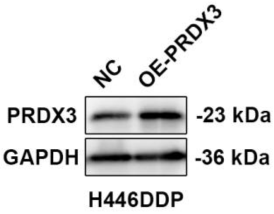

H446DDP

PRDX3

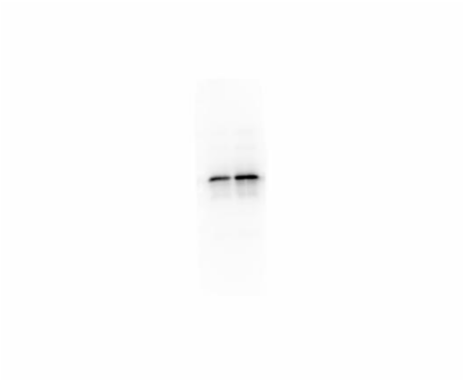
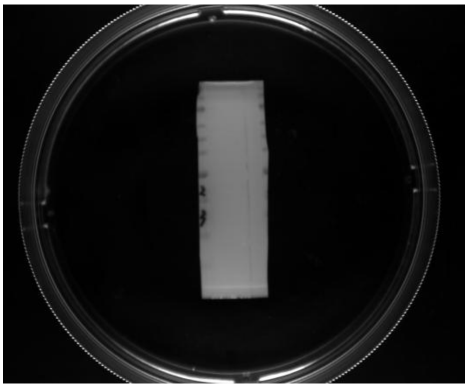

GAPDH

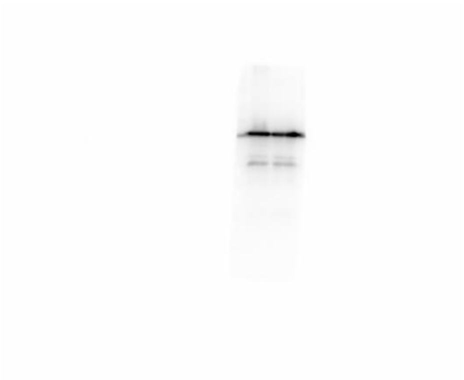
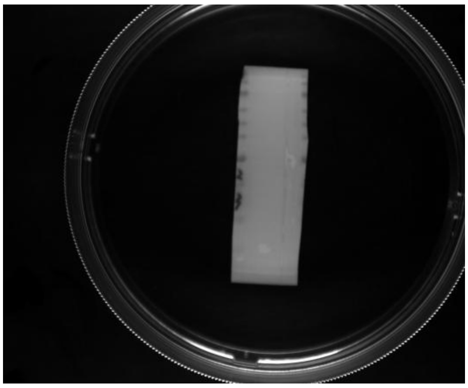

Fig. 9B First Repetition

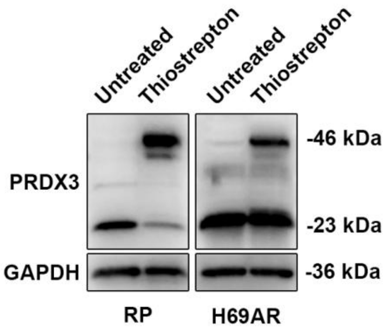

RP

PRDX3

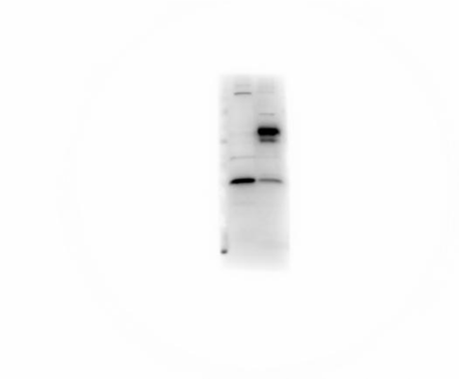

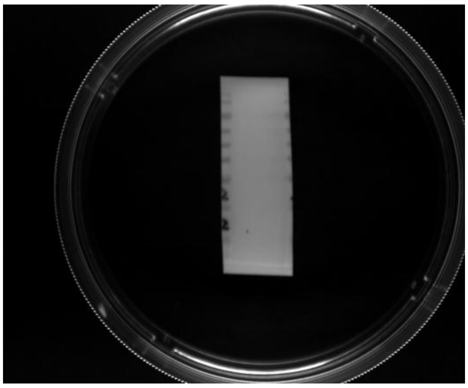

GAPDH

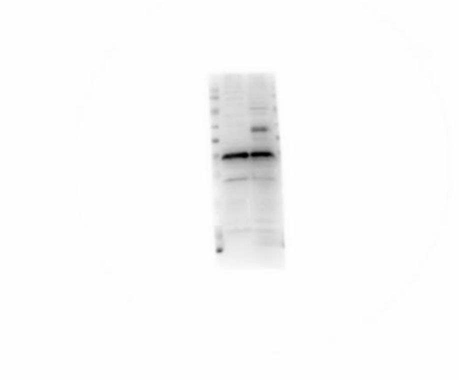

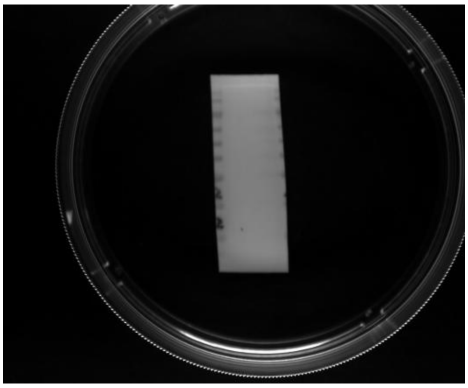

H69AR

PRDX3

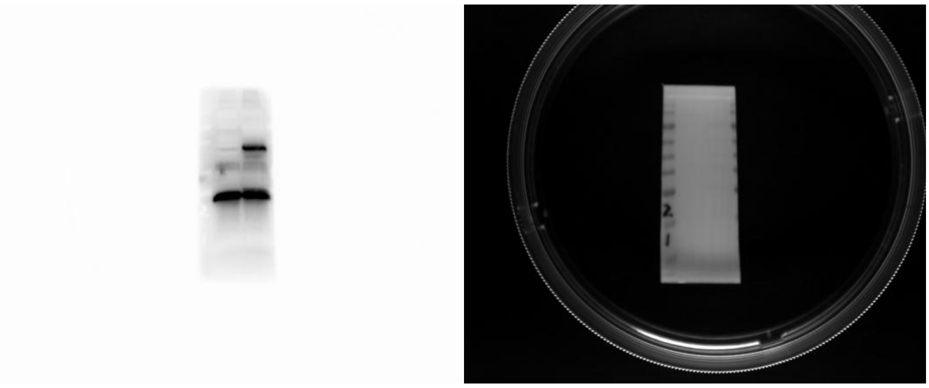

GAPDH

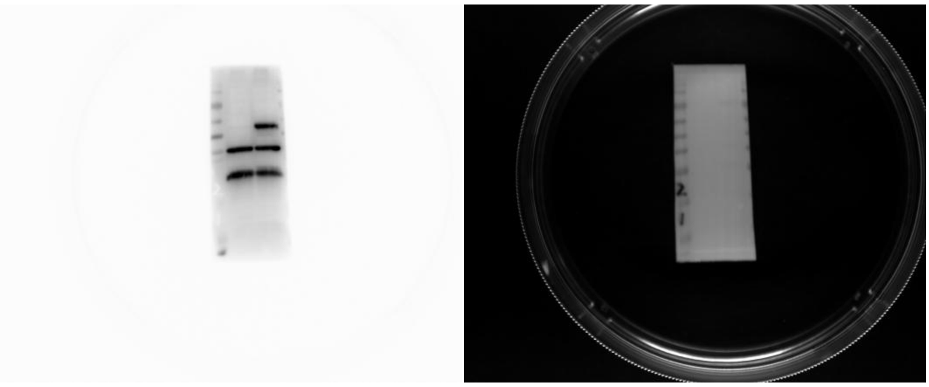

Fig. 9B Second Repetition

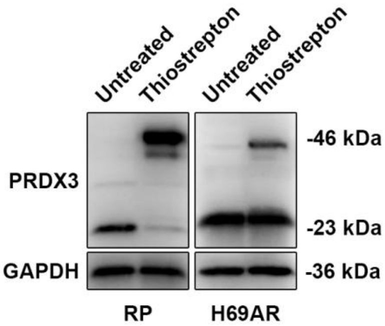

RP

PRDX3

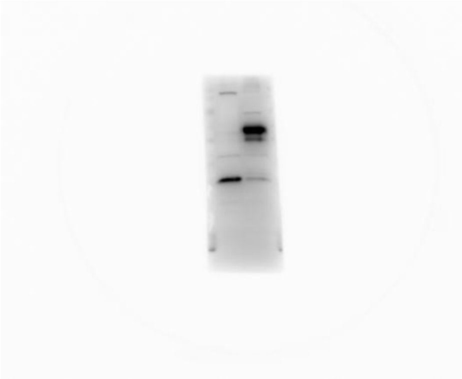
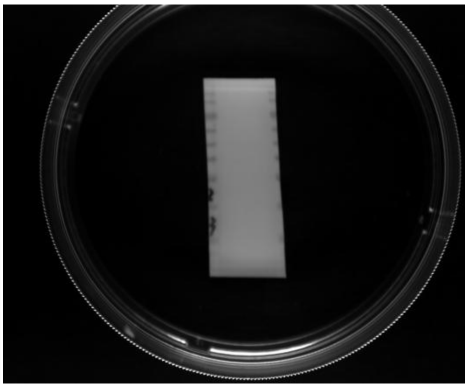

GAPDH

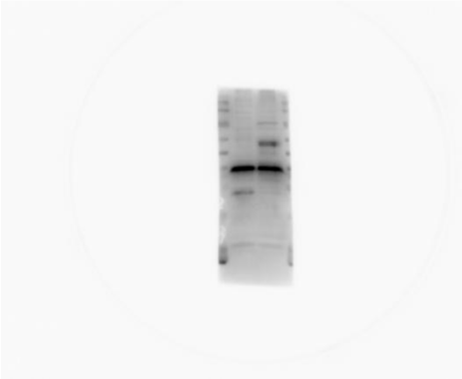
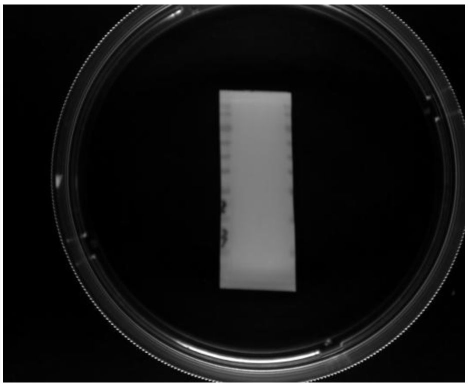

H69AR

PRDX3

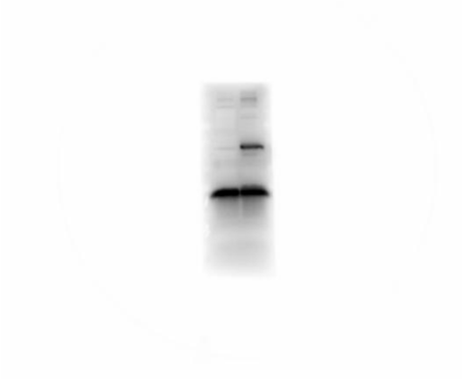
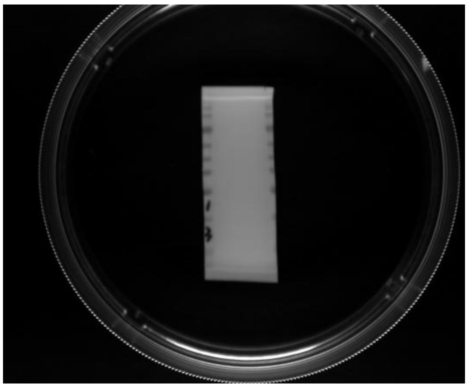

GAPDH

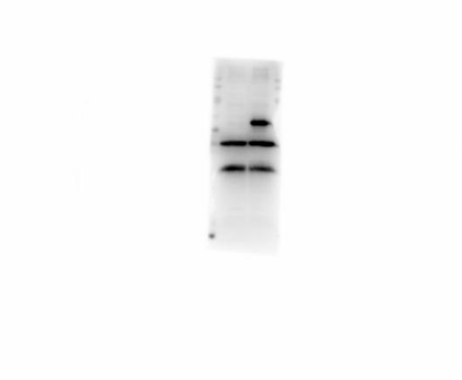

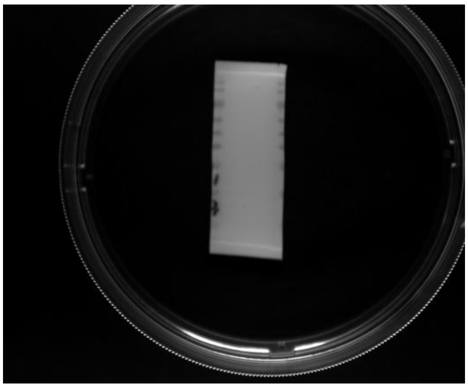

Fig. 9B Third Repetition

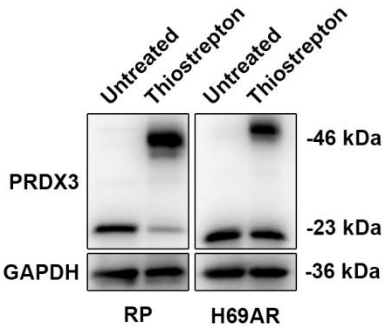

RP

PRDX3

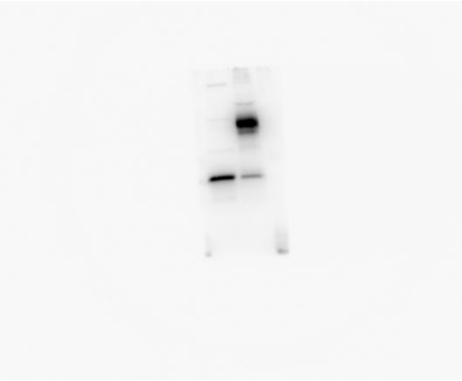

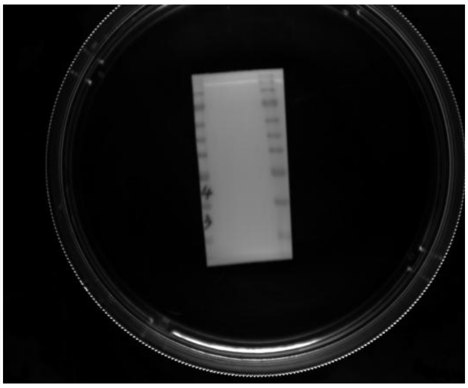

GAPDH

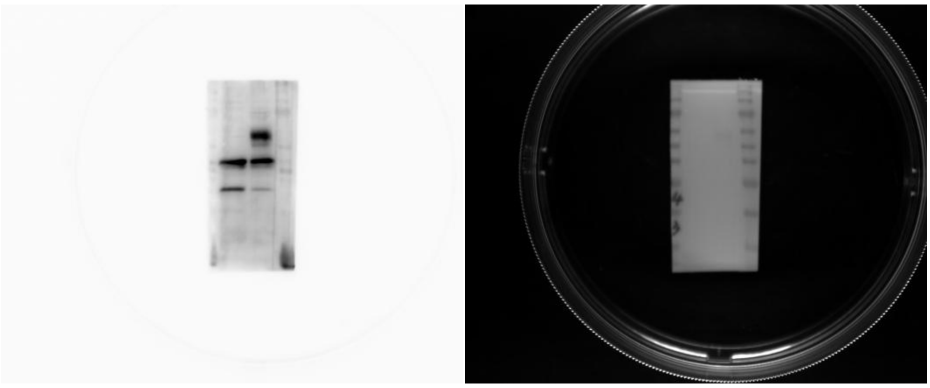

H69AR

PRDX3

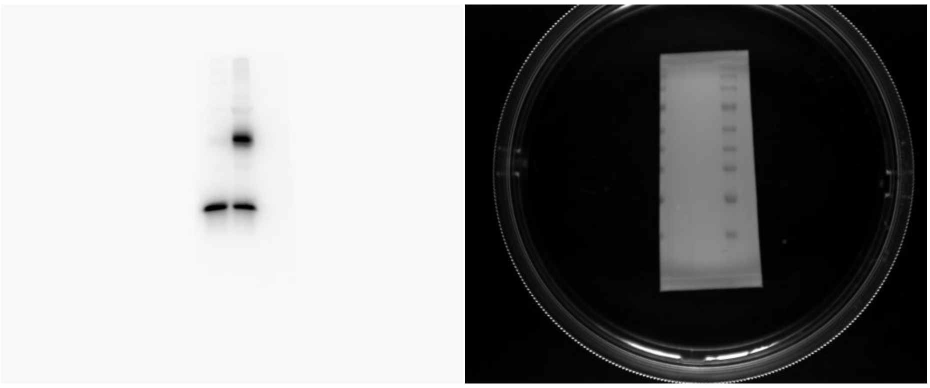

GAPDH

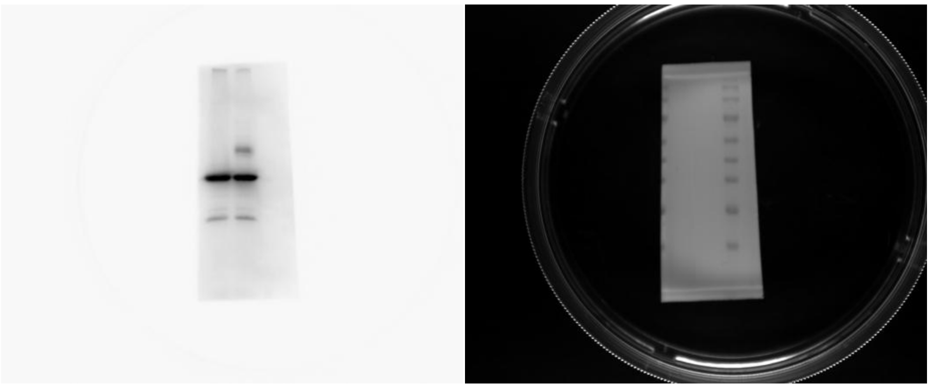

Fig. S3F First Repetition

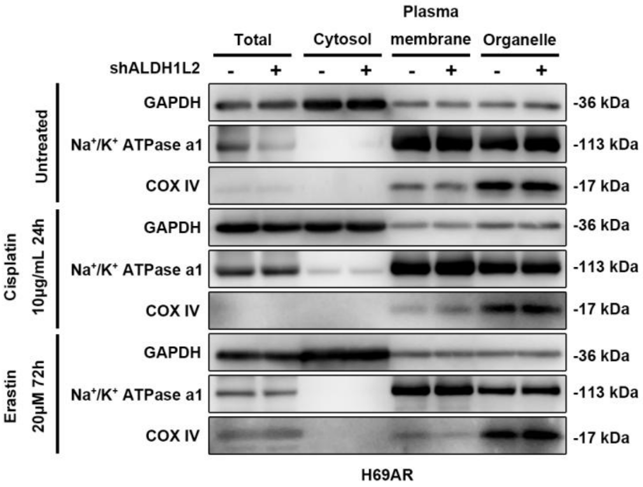

H69AR

GAPDH (untreated)

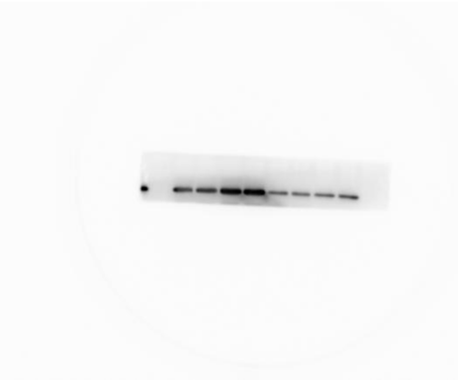

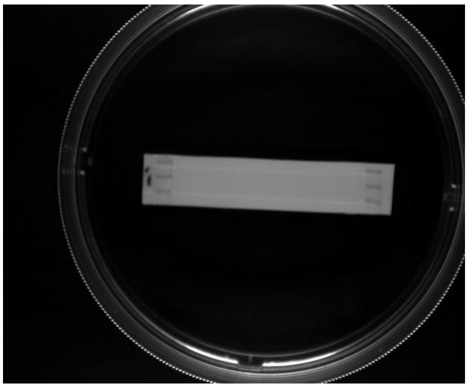

Na^+/K^+ ATPase a1 (untreated)

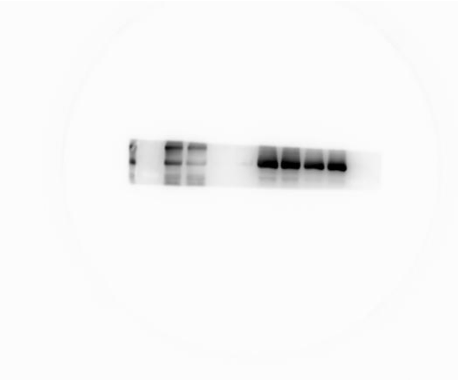

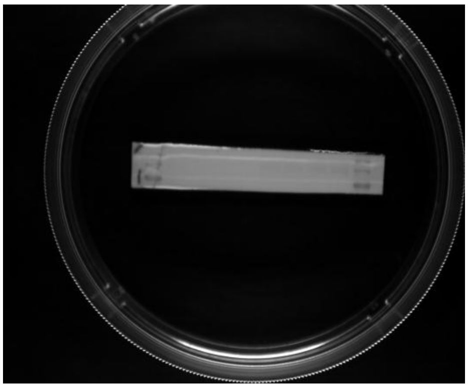

COX IV (untreated)

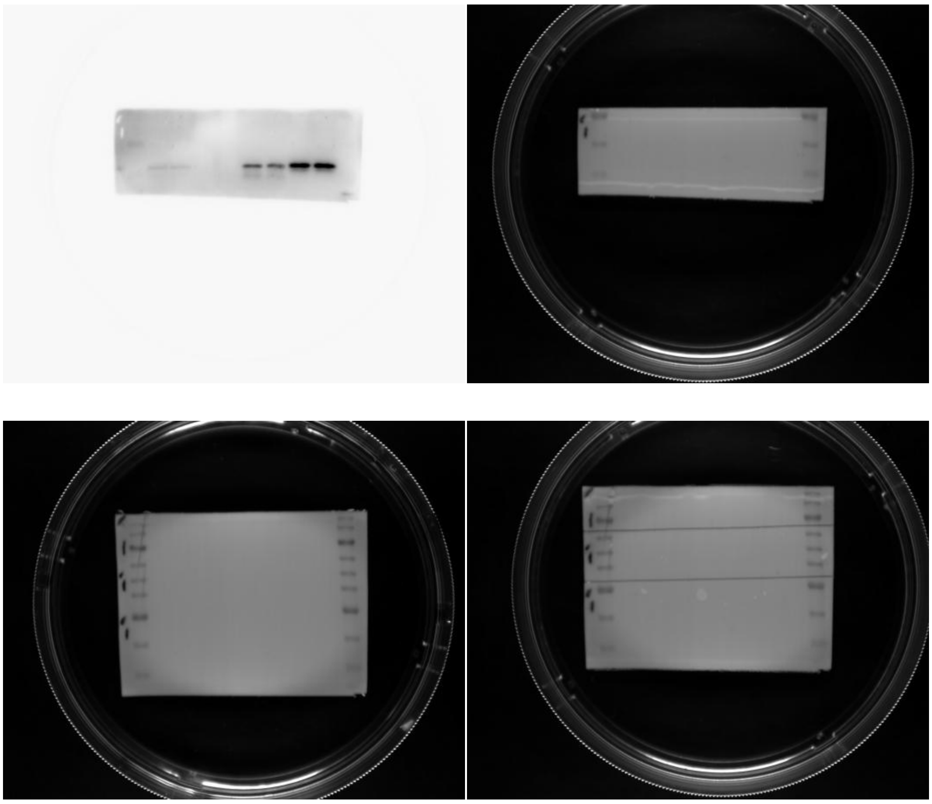

GAPDH (cisplatin)

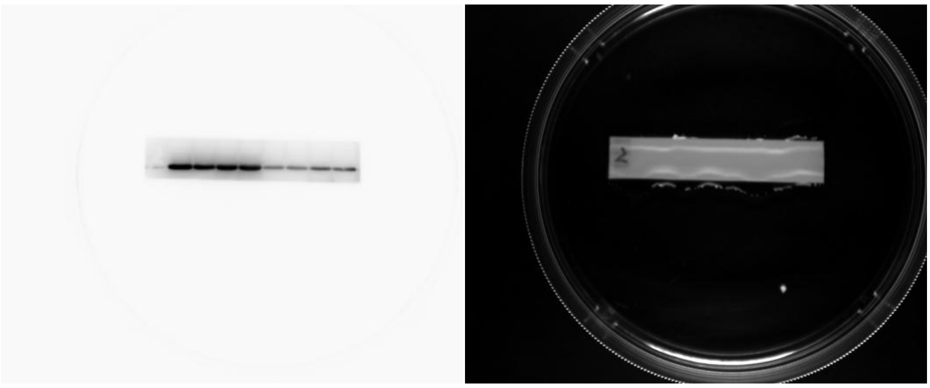

Na^+/K^+ ATPase a1 (cisplatin)

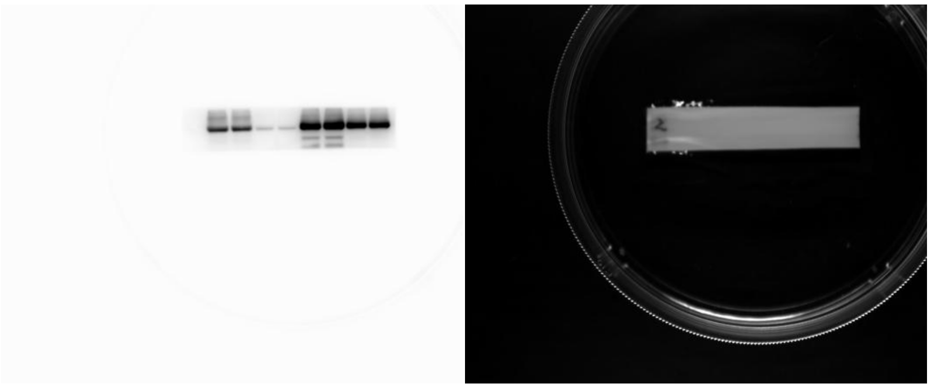

COX IV (cisplatin)

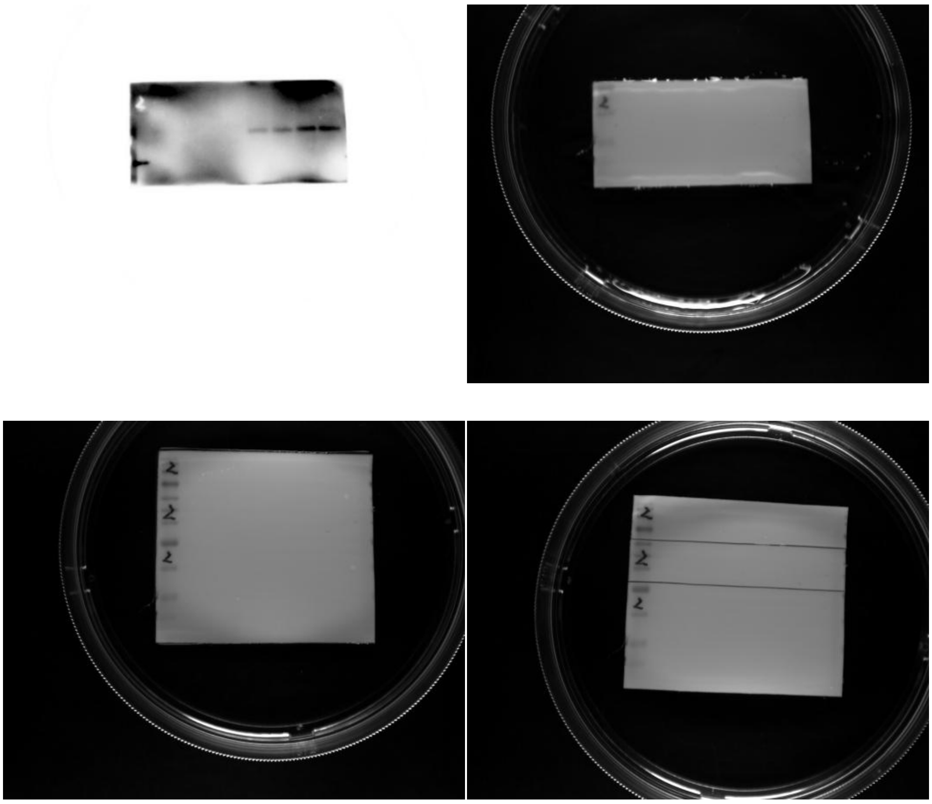

GAPDH (erastin)

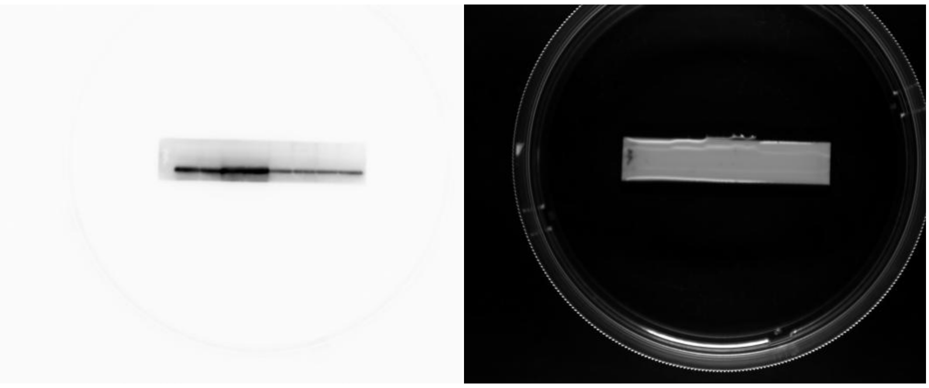

Na^+/K^+ ATPase a1 (erastin)

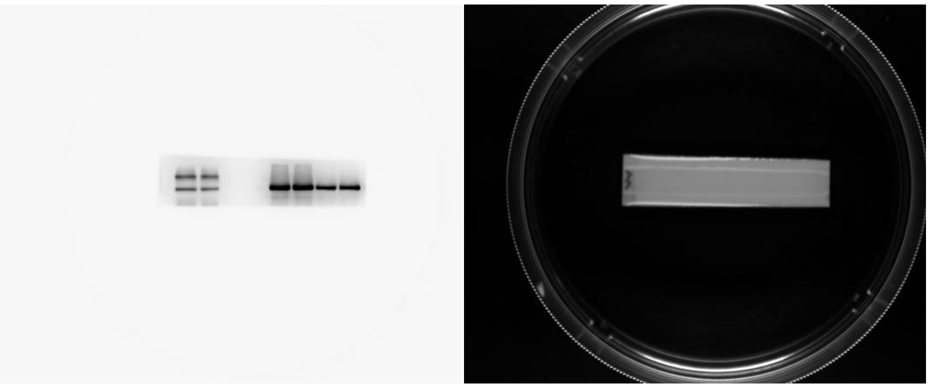

COX IV (erastin)

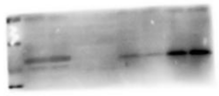

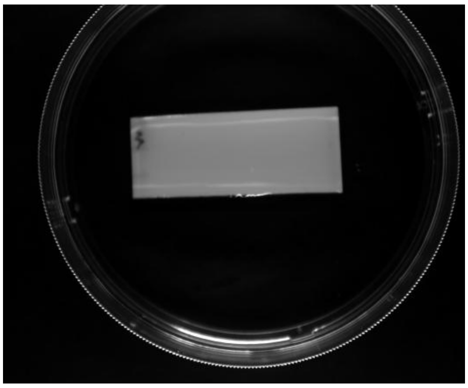

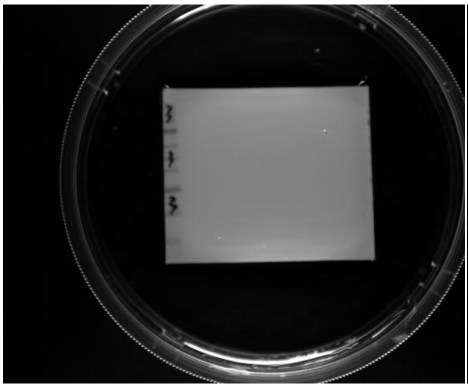

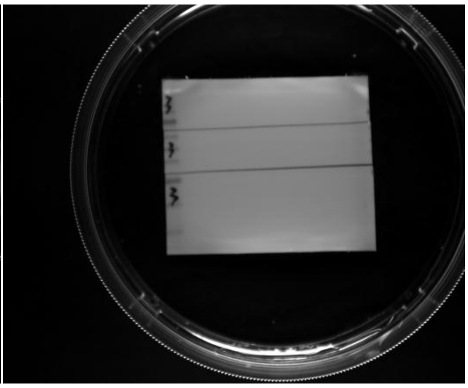

Fig. S3F Second Repetition

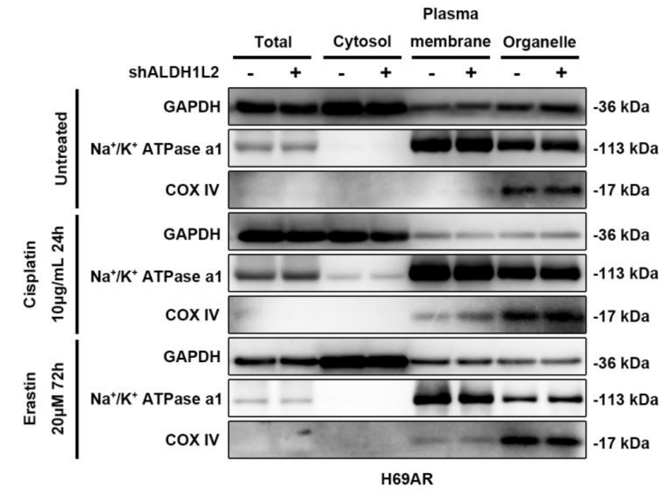

H69AR

GAPDH (untreated)

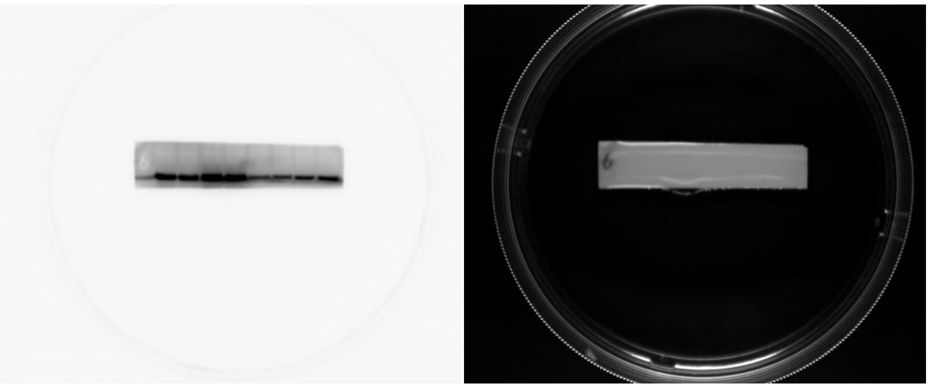

Na^+/K^+ ATPase a1 (untreated)

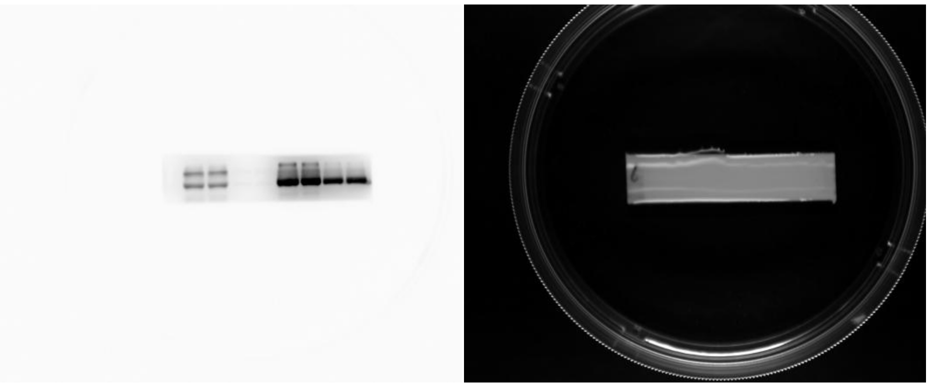

COX IV (untreated)

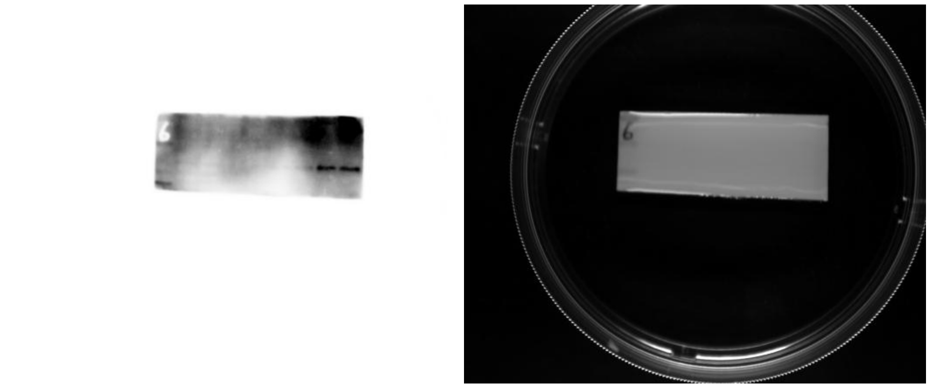

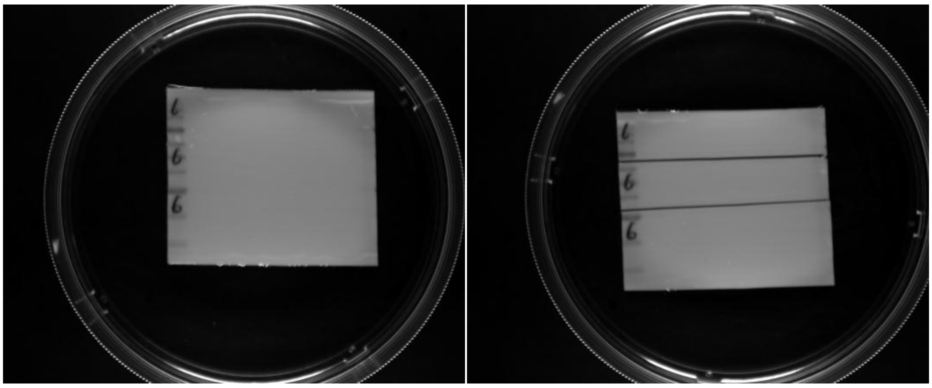

GAPDH (cisplatin)

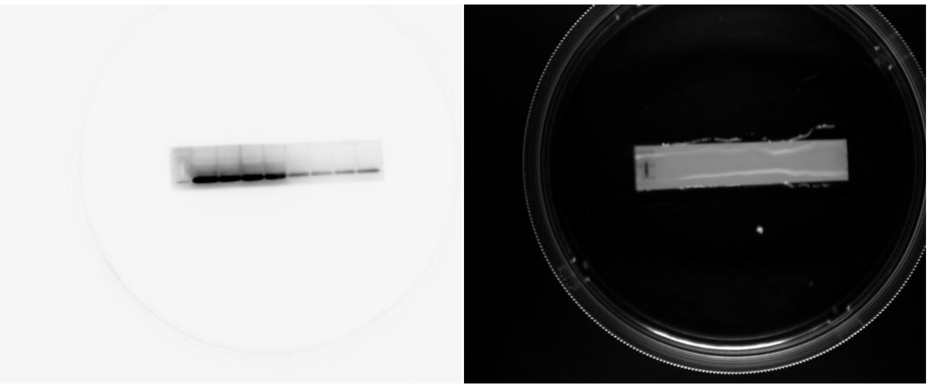

Na^+/K^+ ATPase a1 (cisplatin)

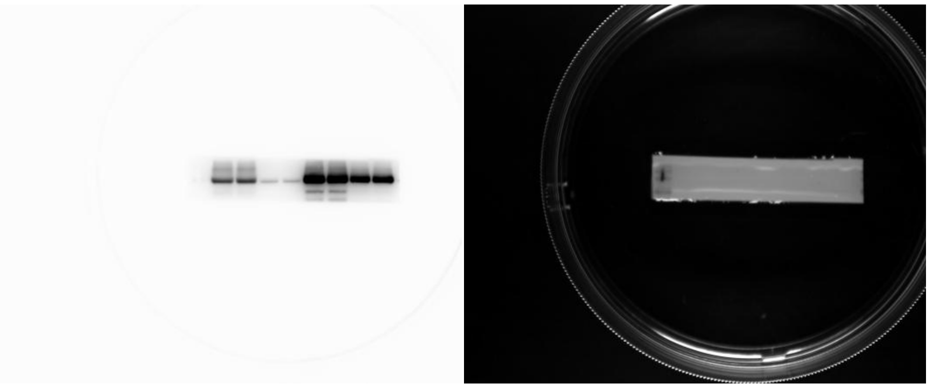

COX IV (cisplatin)

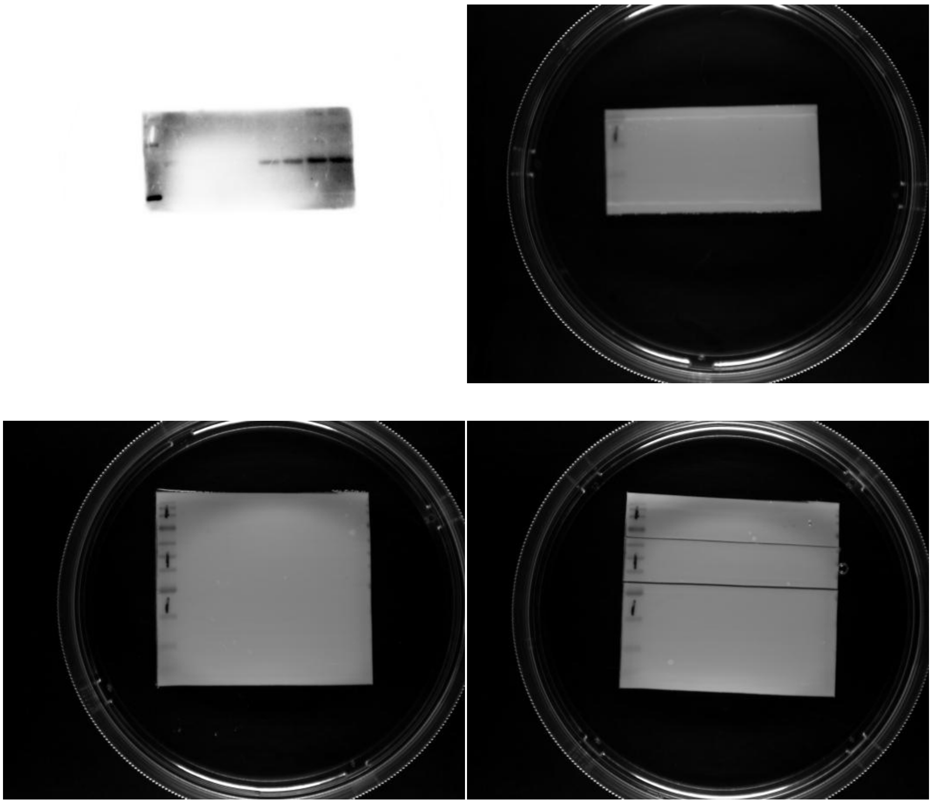

GAPDH (erastin)

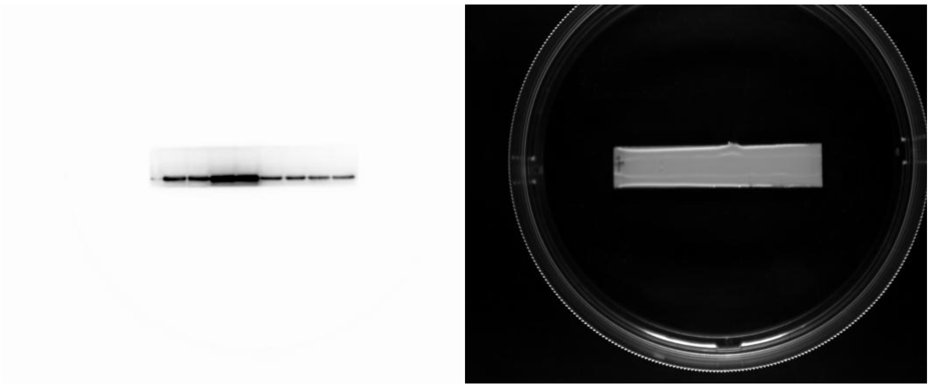

Na^+/K^+ ATPase a1 (erastin)

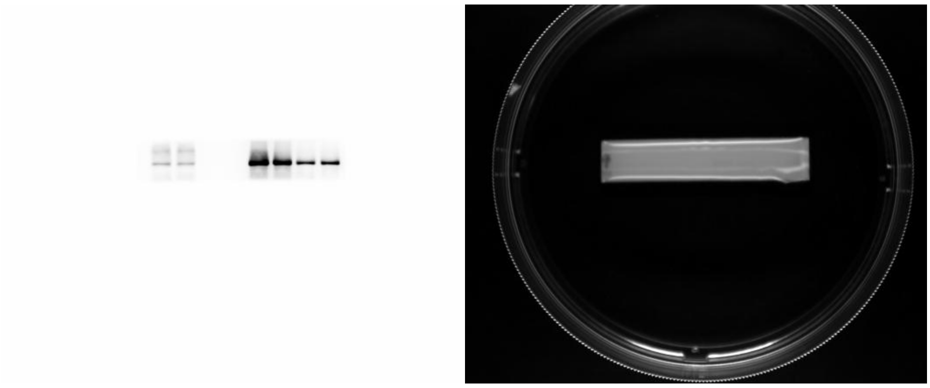

COX IV (erastin)

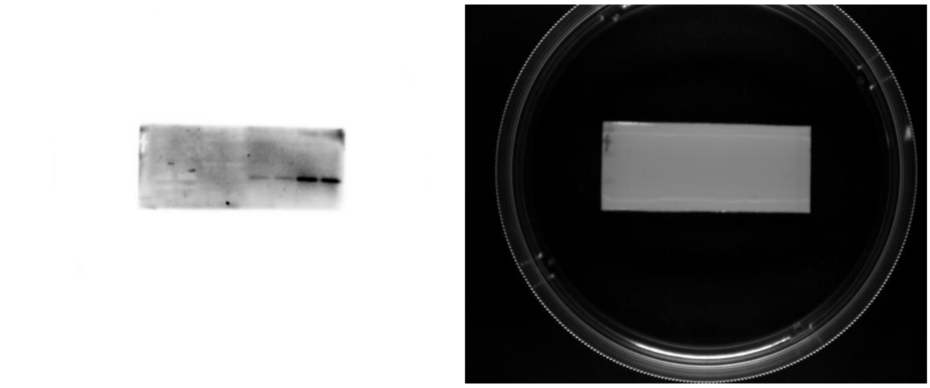

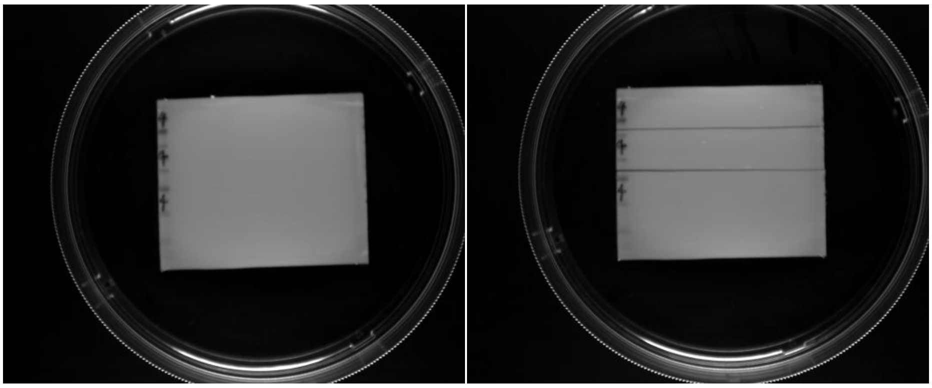

Fig. S3F Third Repetition

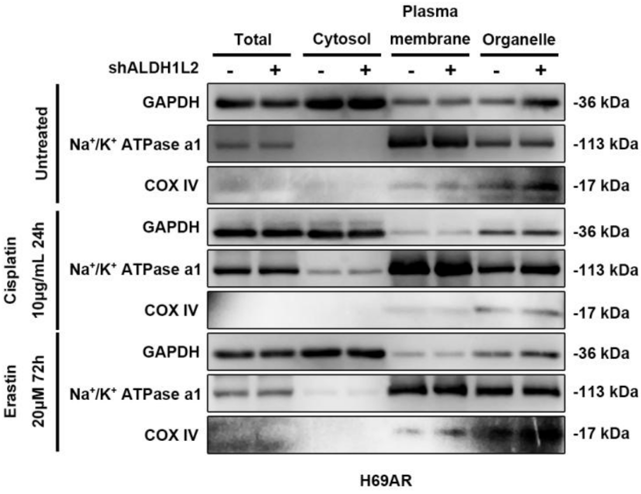

H69AR

GAPDH (untreated)

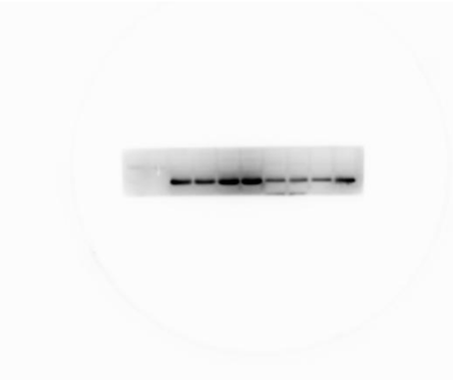

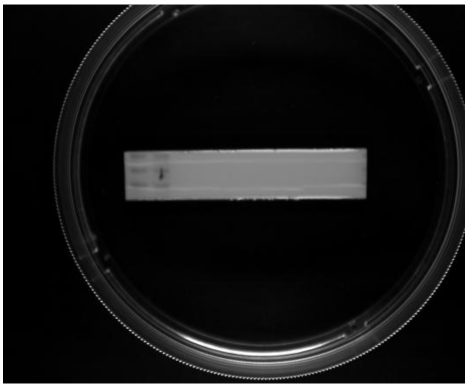

Na^{+}/K^{+} ATPase a1 (untreated)

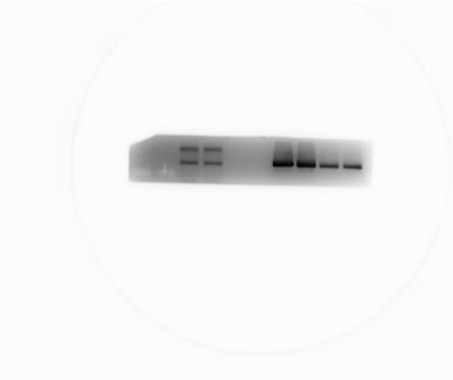

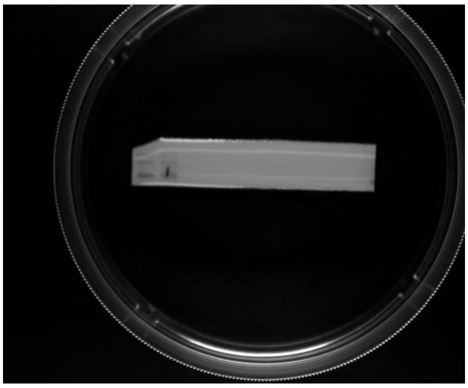

COX IV (untreated)

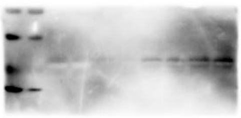

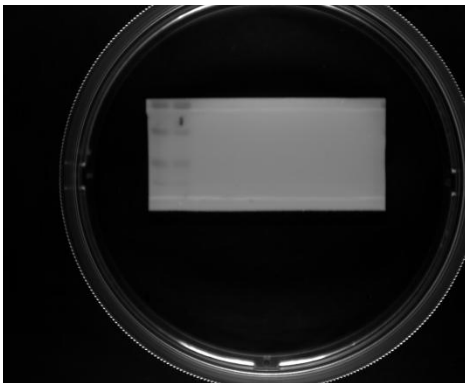

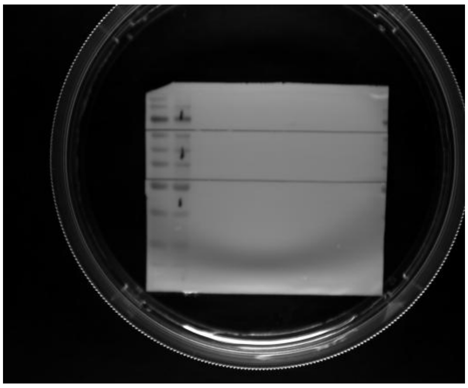

GAPDH (cisplatin)

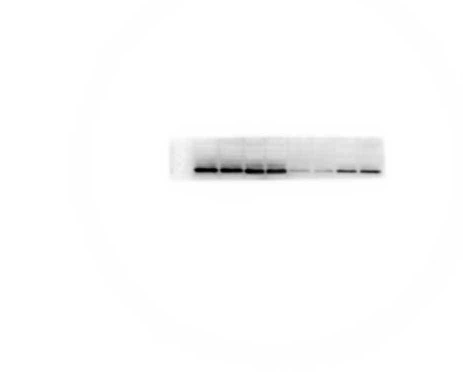

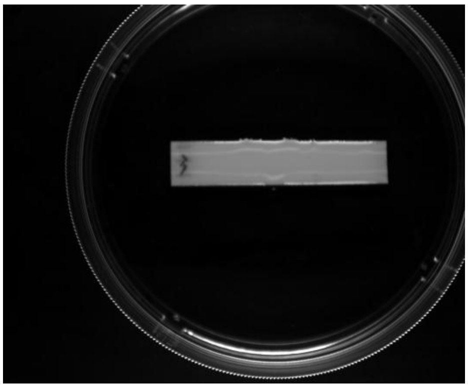

Na^{+}/K^{+} ATPase a1 (cisplatin)

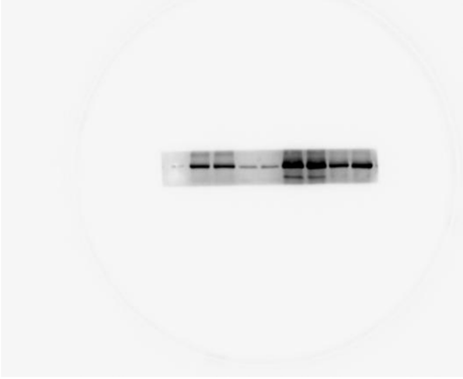

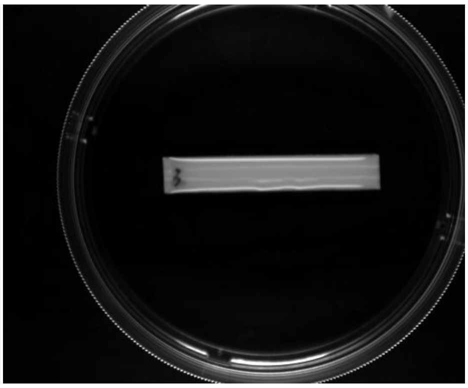

COX IV (cisplatin)

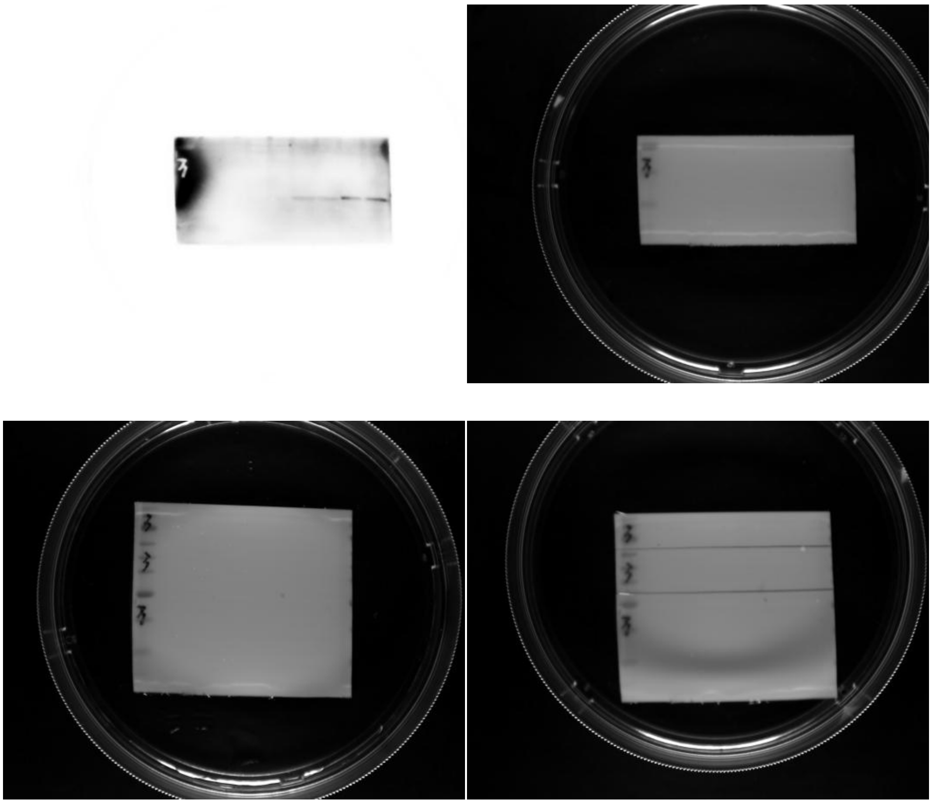

GAPDH (erastin)

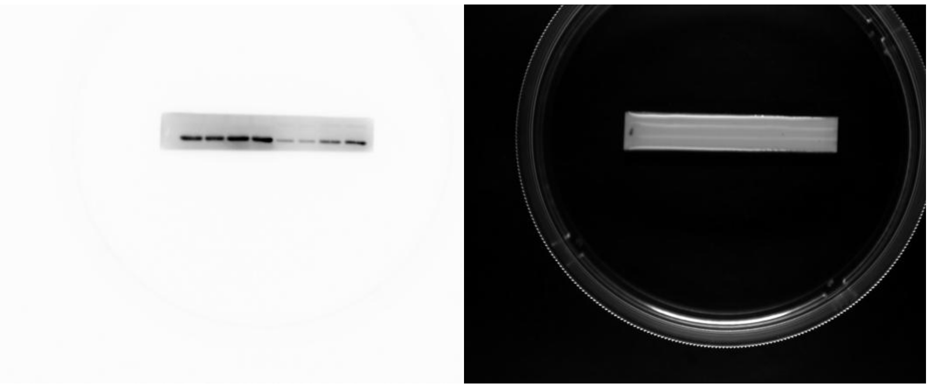

Na^{+}/K^{+} ATPase a1 (erastin)

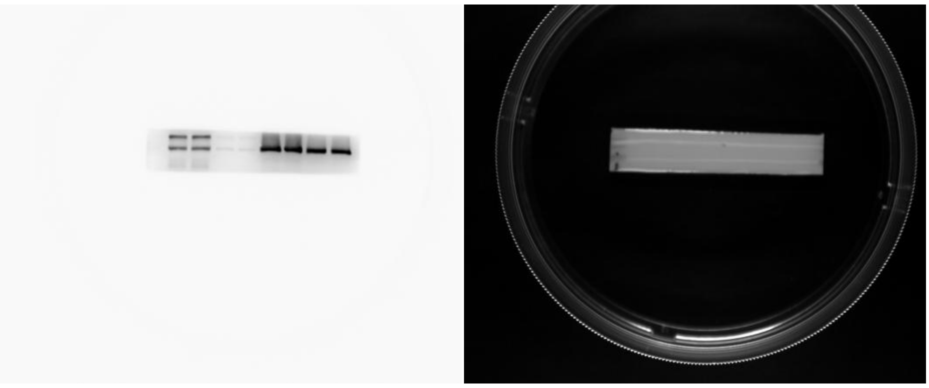

COX IV (erastin)

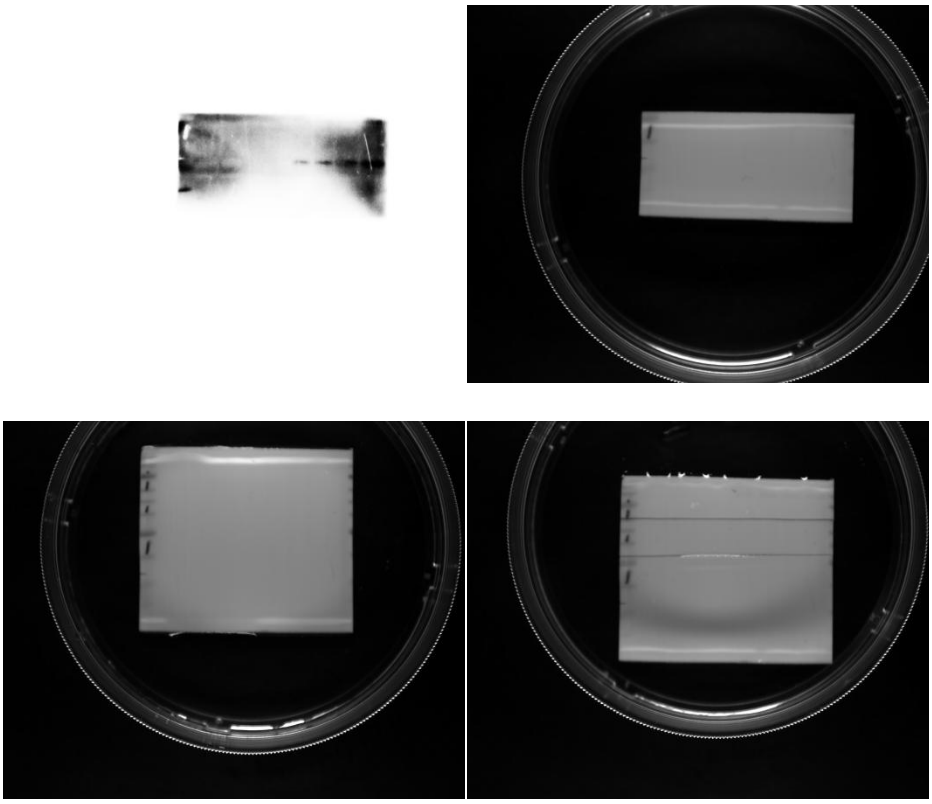

Fig. S3G First Repetition

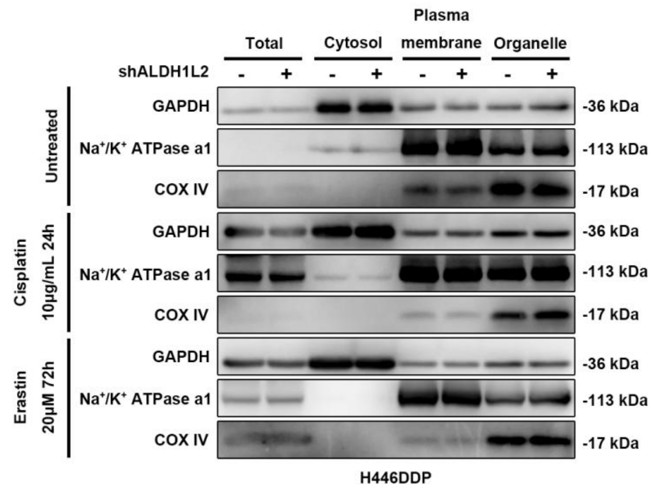

H446DDP

GAPDH (untreated)

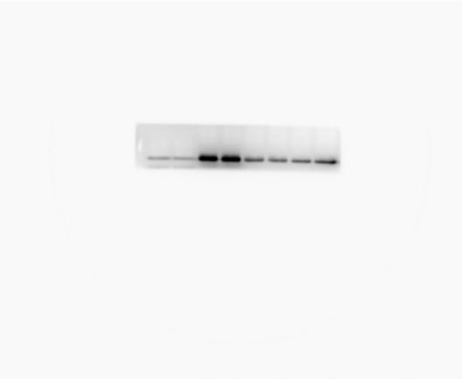 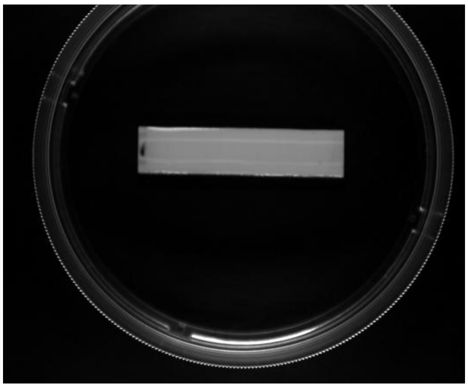

Na^+/K^+ ATPase a1 (untreated)

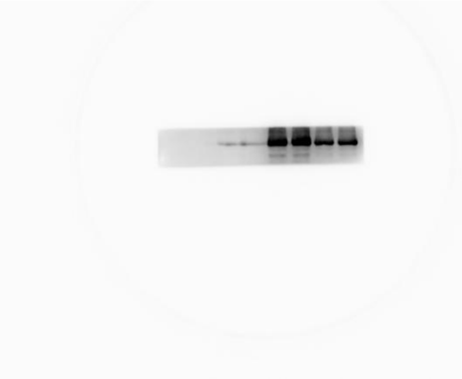 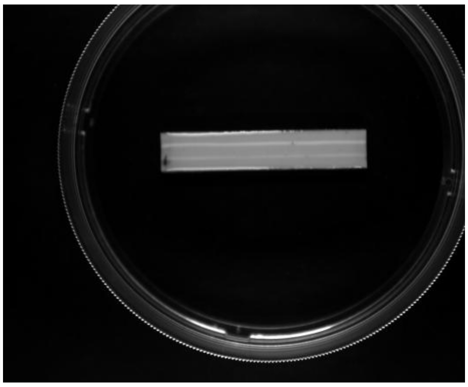

COX IV (untreated)

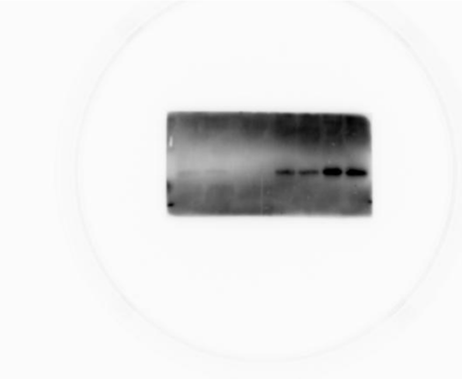 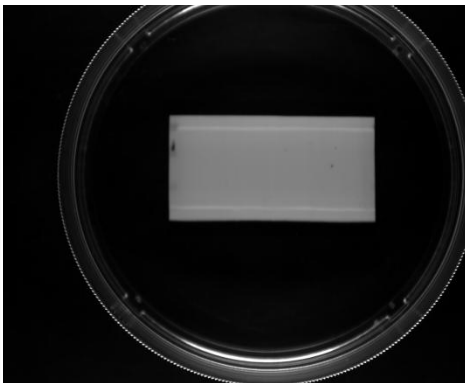

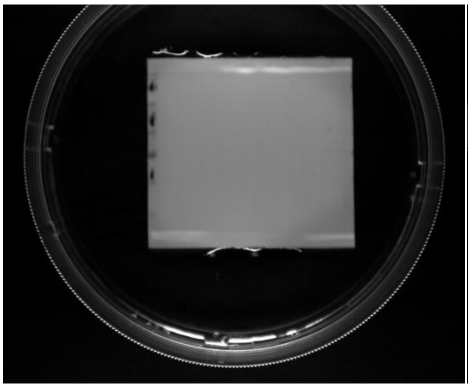 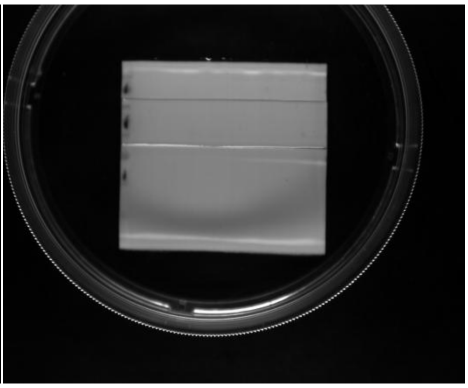

GAPDH (cisplatin)

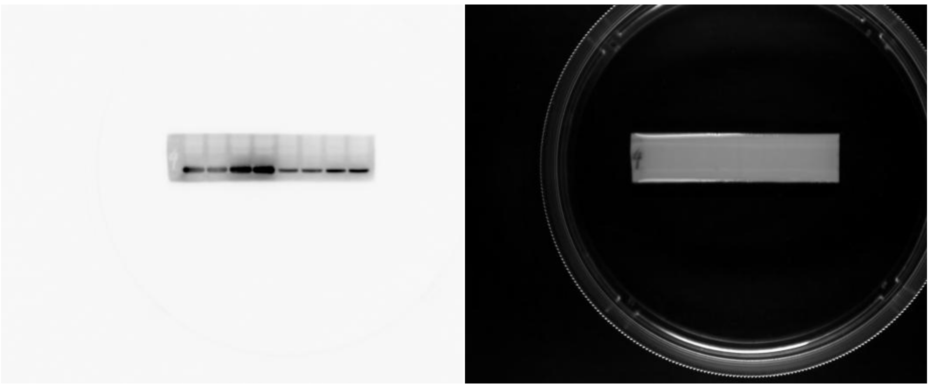

Na^{+}/K^{+} ATPase a1 (cisplatin)

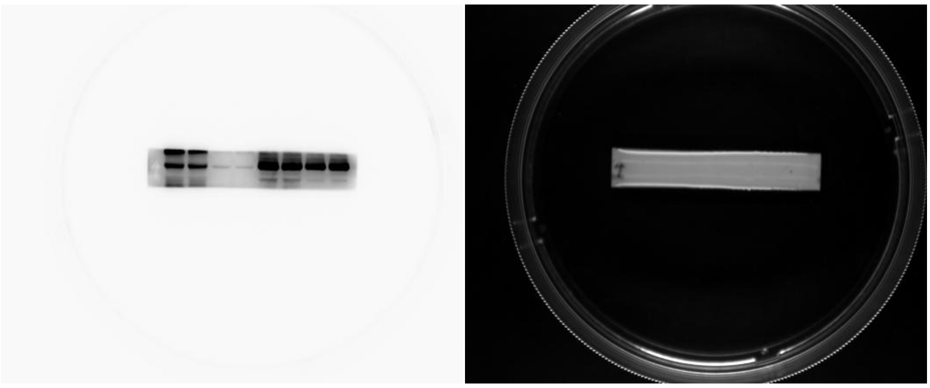

COX IV (cisplatin)

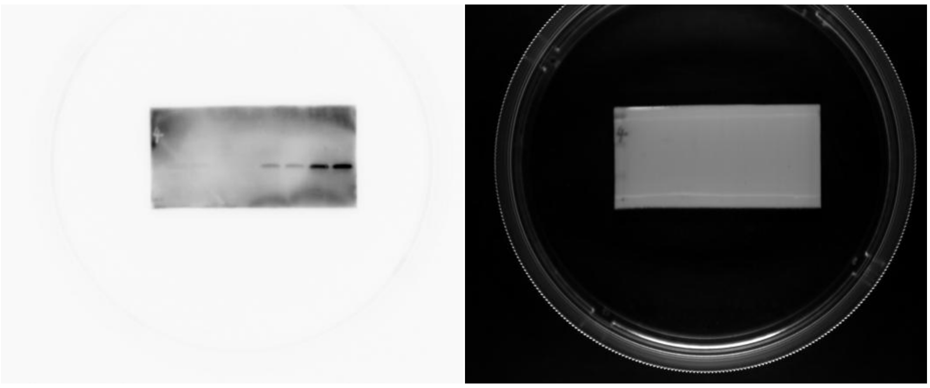

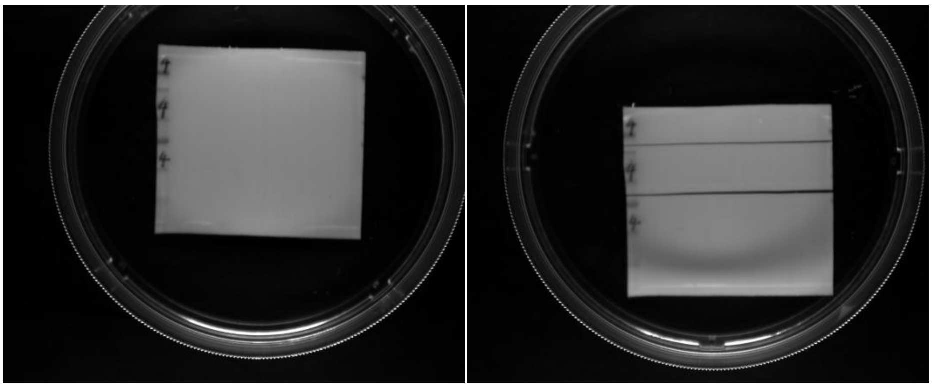

GAPDH (erastin)

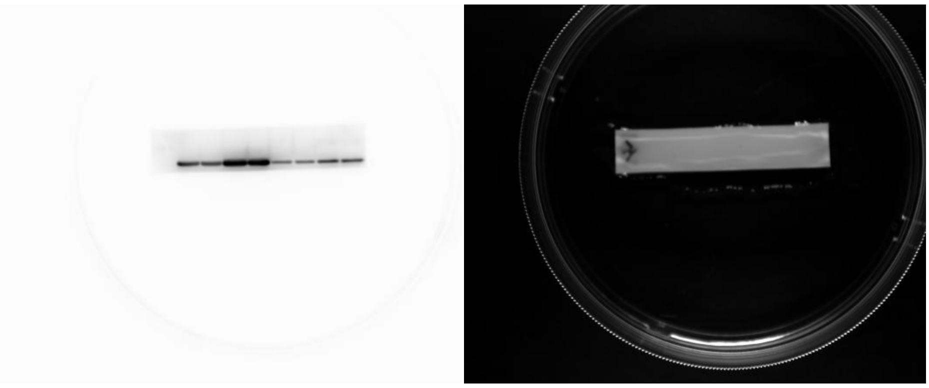

Na^{+}/K^{+} ATPase a1 (erastin)

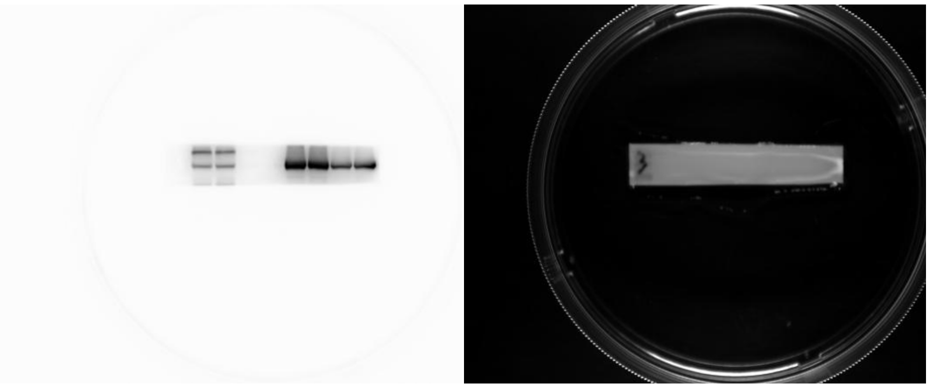

COX IV (erastin)

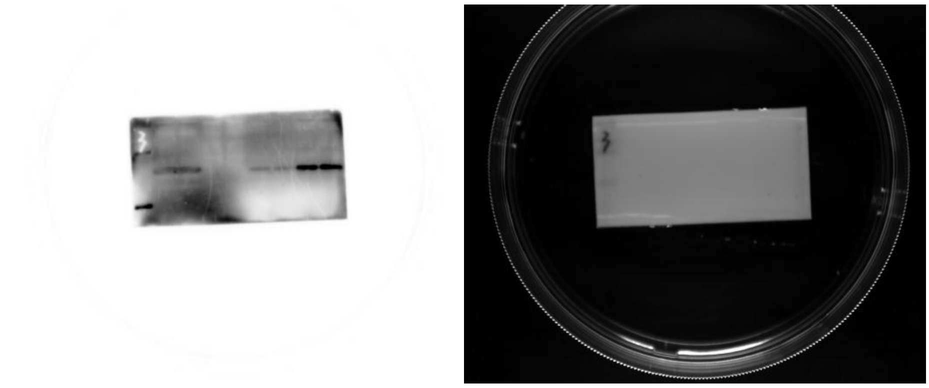

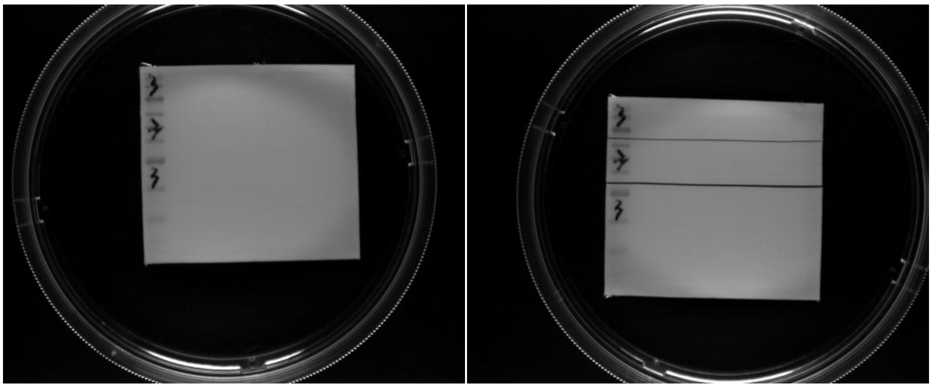

Fig. S3G Second Repetition

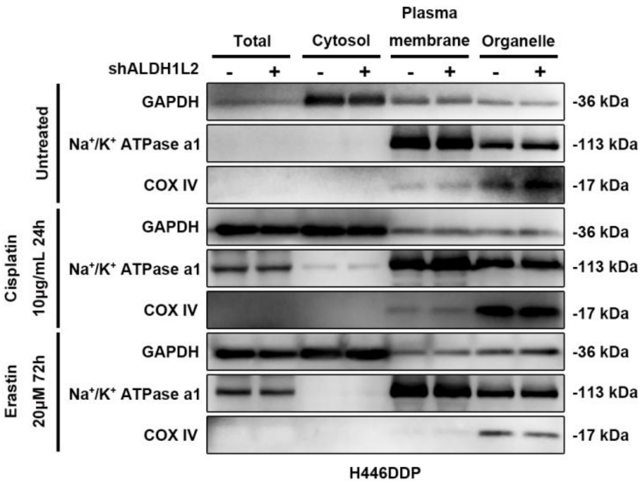

H446DDP

GAPDH (untreated)

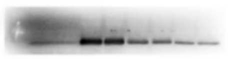

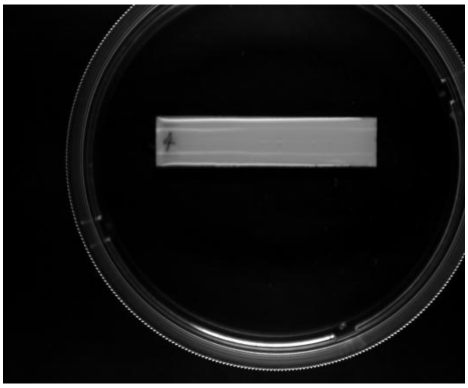

Na$^+$/K$^+$ ATPase a1 (untreated)

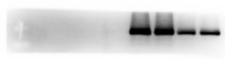

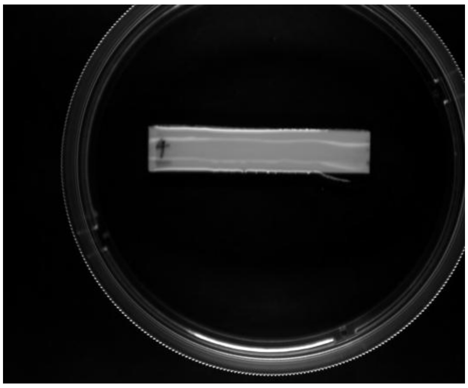

COX IV (untreated)

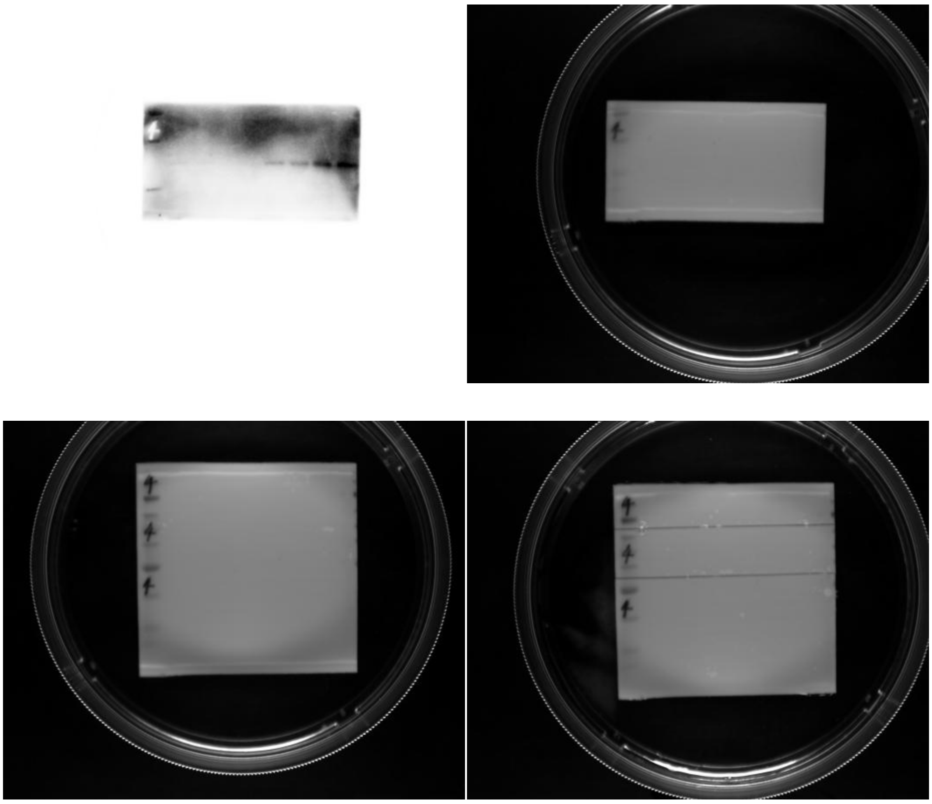

GAPDH (cisplatin)

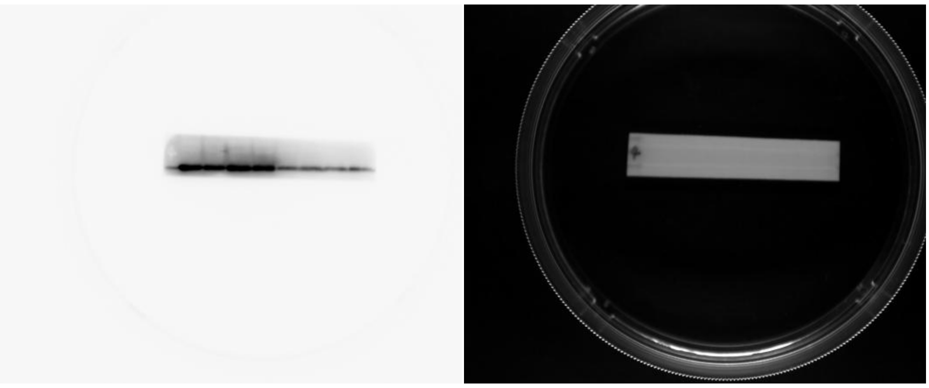

Na^+/K^+ ATPase a1 (cisplatin)

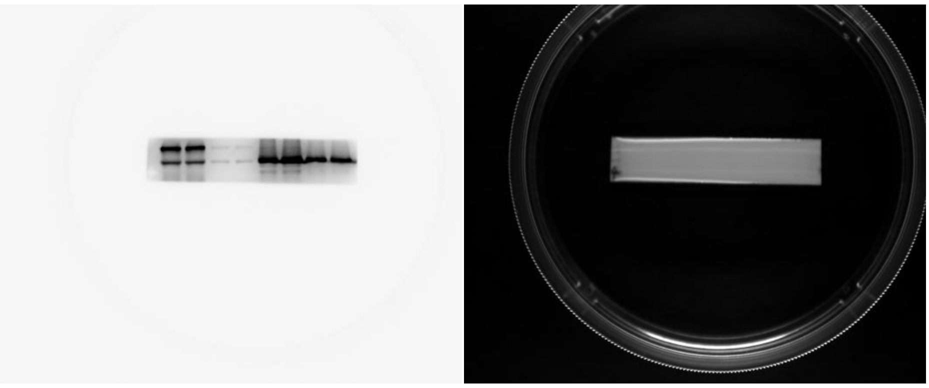

COX IV (cisplatin)

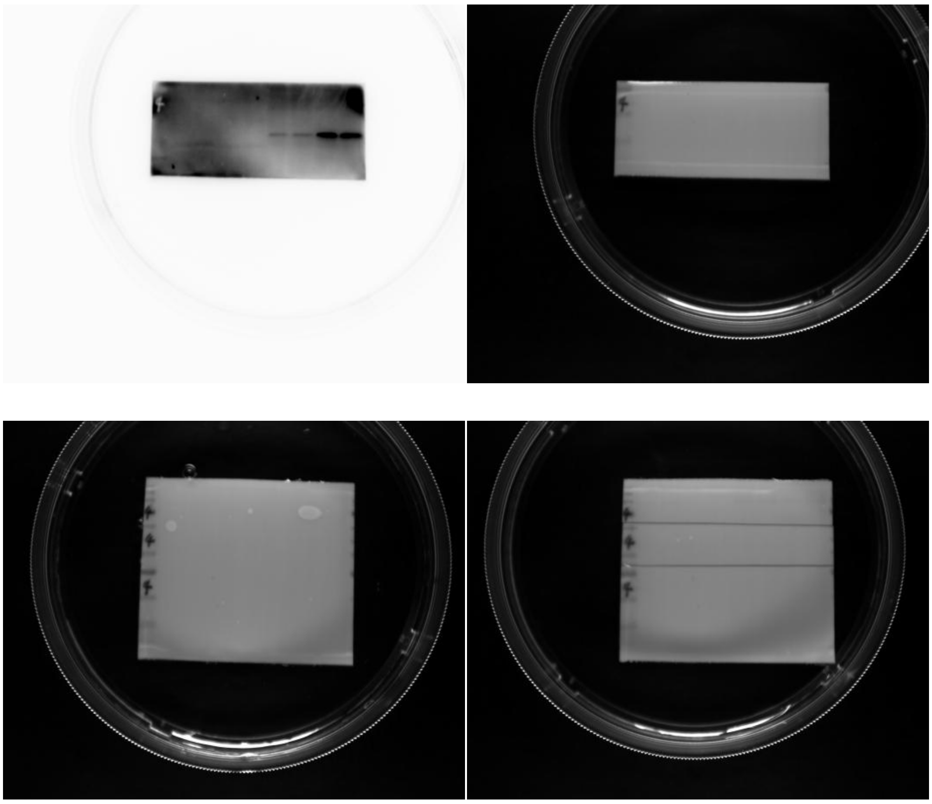

GAPDH (erastin)

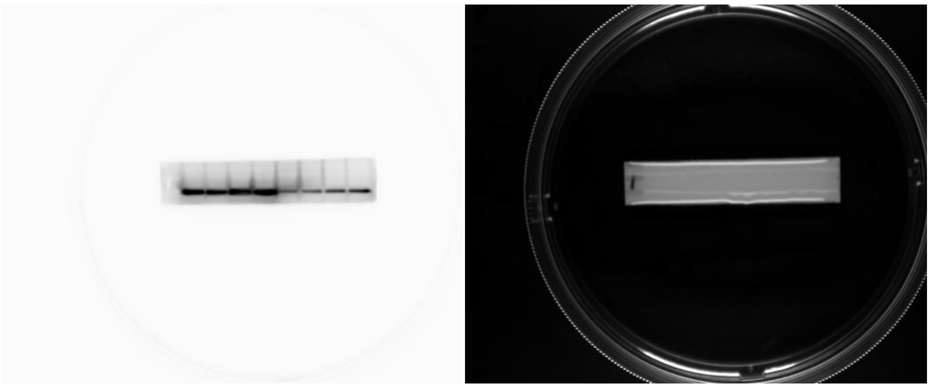

Na^+/K^+ ATPase a1 (erastin)

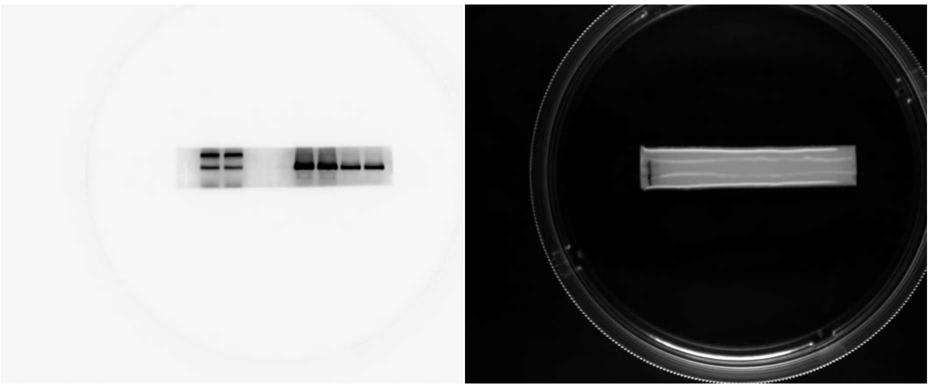

COX IV (erastin)

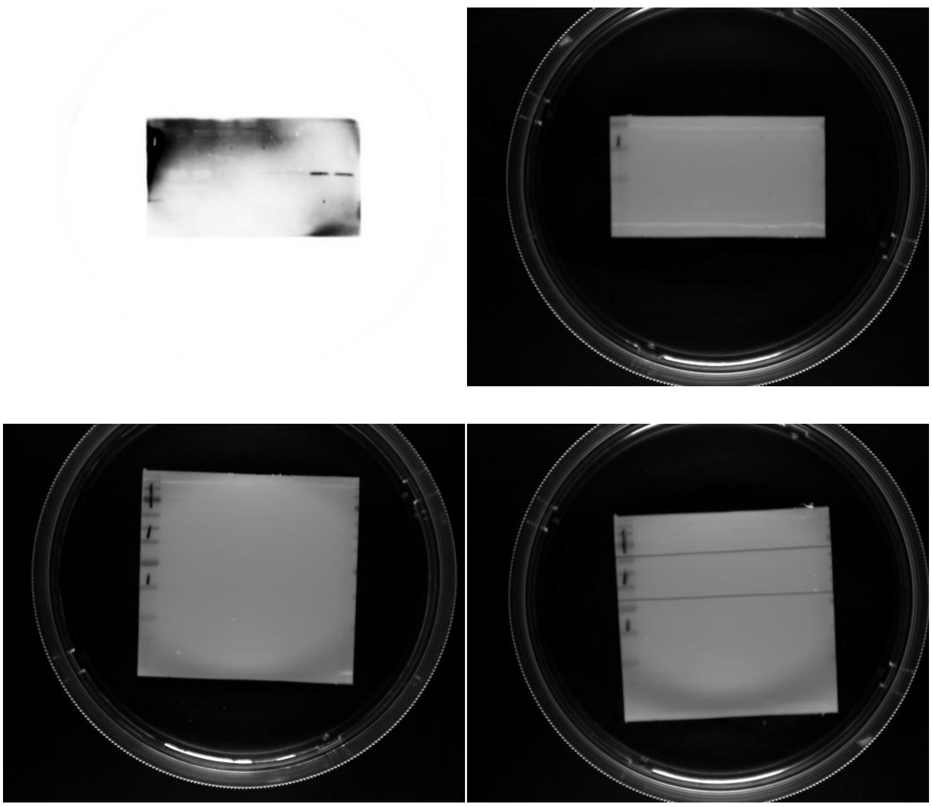

Fig. S3G Third Repetition

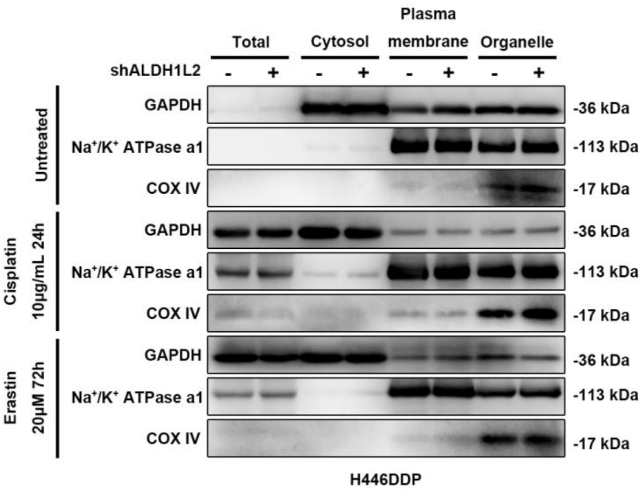

H446DDP

GAPDH (untreated)

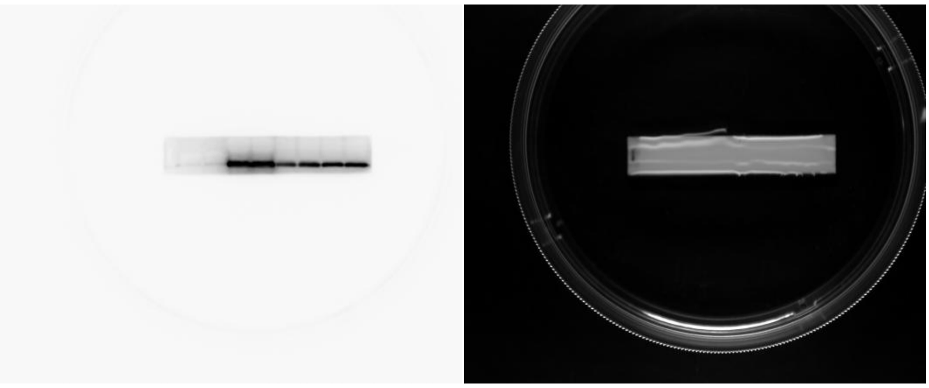

Na^{+}/K^{+} ATPase a1 (untreated)

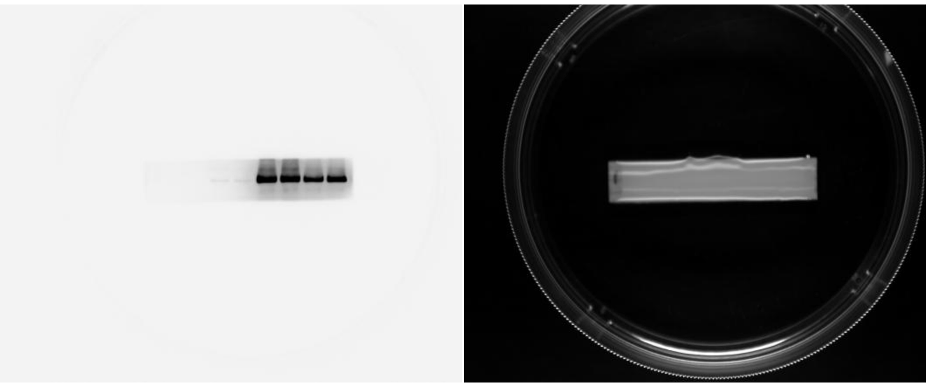

COX IV (untreated)

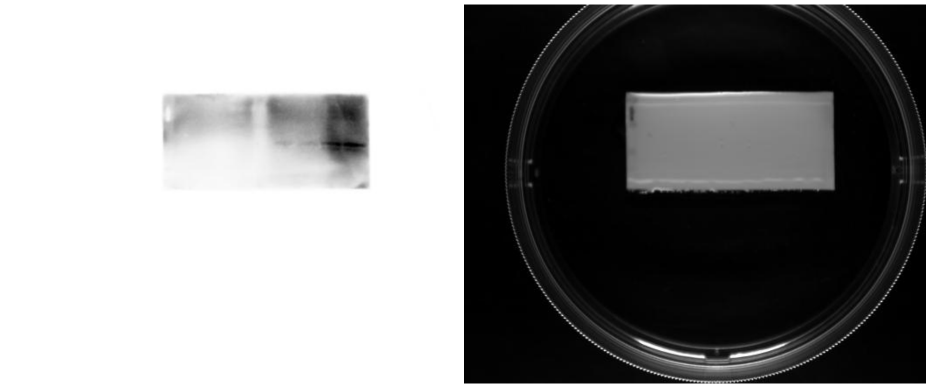

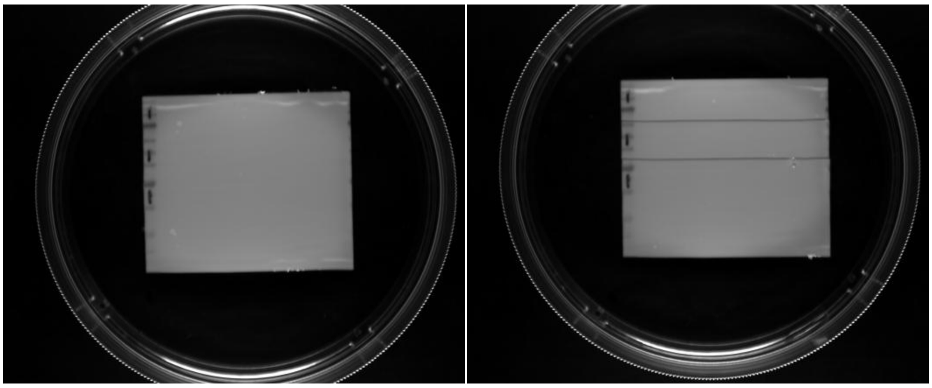

GAPDH (cisplatin)

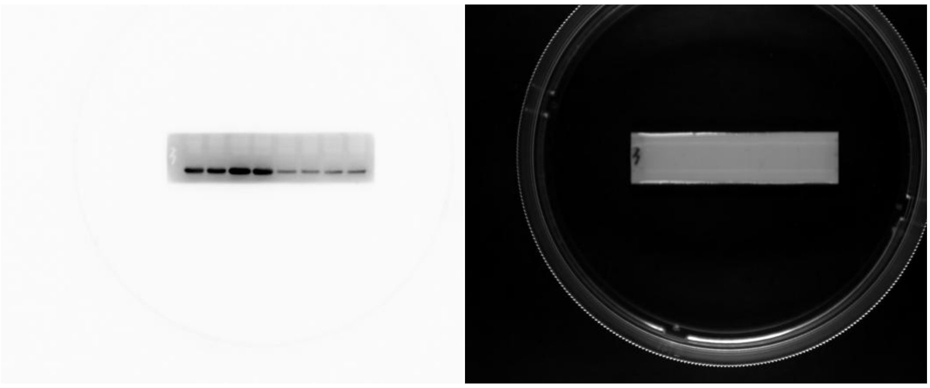

Na^+/K^+ ATPase a1 (cisplatin)

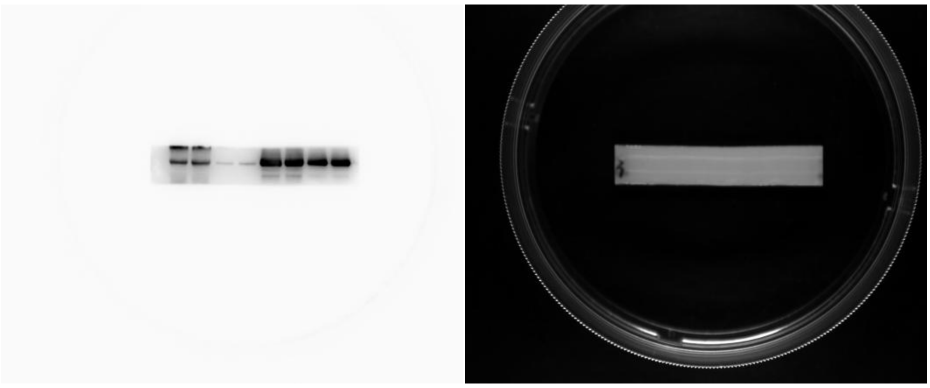

COX IV (cisplatin)

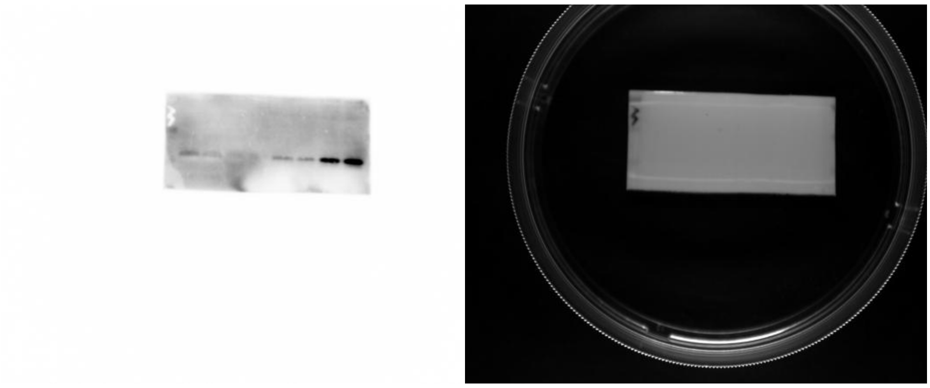

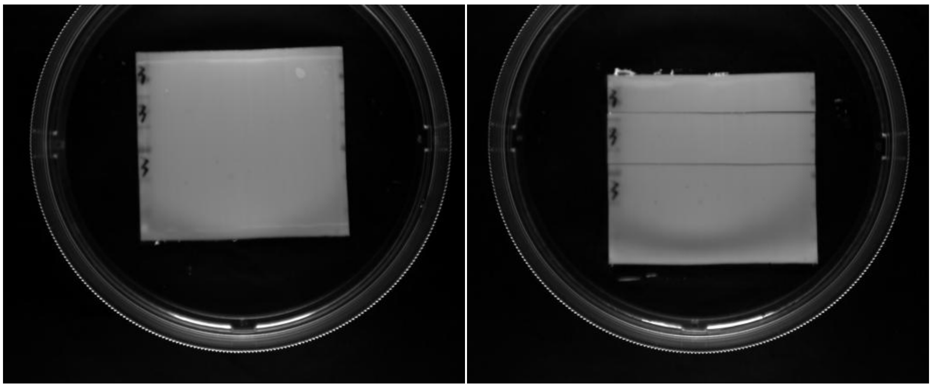

GAPDH (erastin)

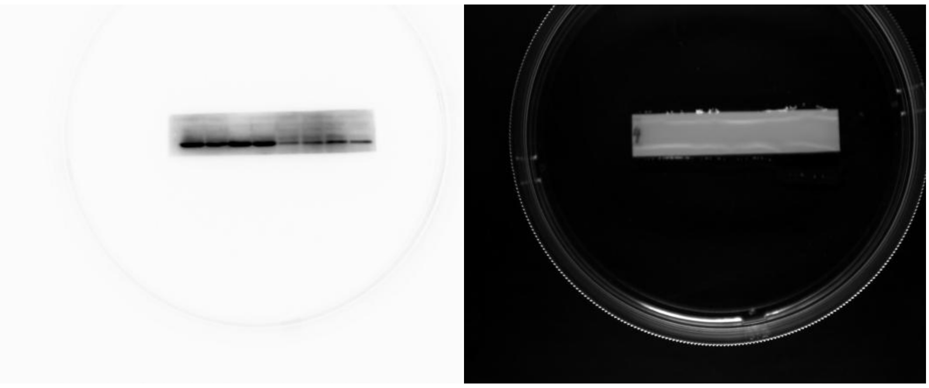

Na^+/K^+ ATPase a1 (erastin)

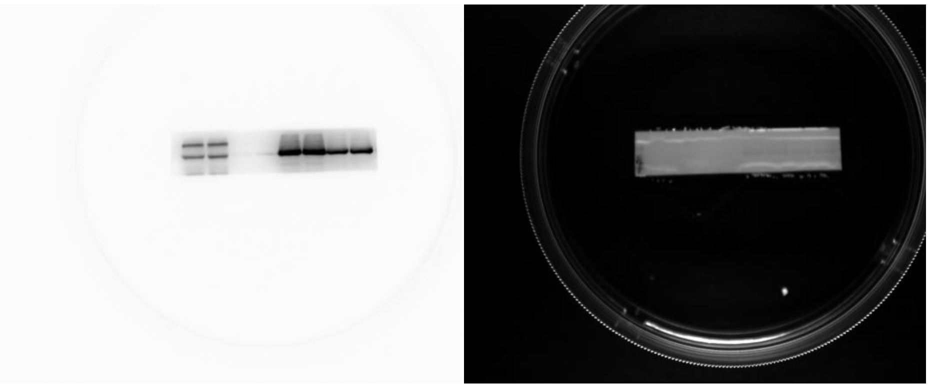

COX IV (erastin)

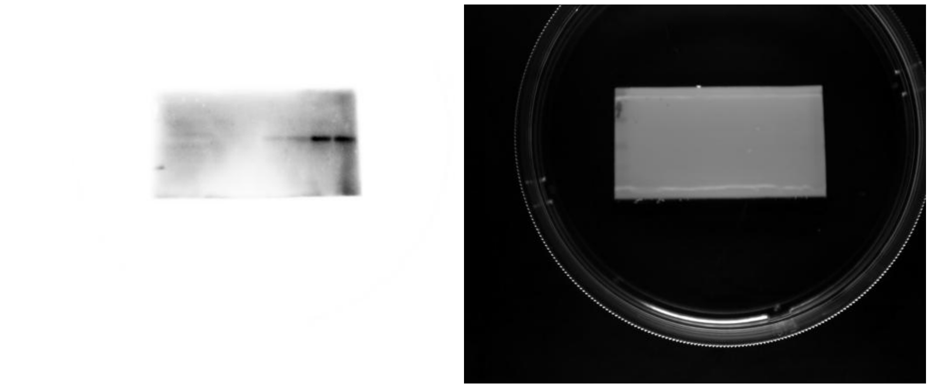

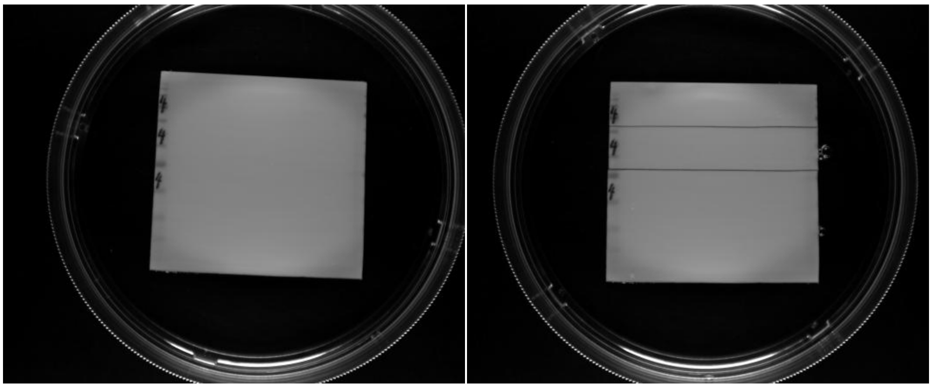

Supplement: Multimedia component 5 [file mmc5.pdf]
