# Supplementary material for: ALDH1L2 induces resistance to chemotherapy in small cell lung cancer by inhibiting ferroptosis
Source: Redox Biol. 2026 Feb 23;91:104098. doi: 10.1016/j.redox.2026.104098 (PMC12966749; doi:10.1016/j.redox.2026.104098)
Supplement: Fig. S3 — ALDH1L2 promotes SCLC chemoresistance by negatively regulating the hyperoxidized PRDX3 and PRDX3 dimer content in the plasma membrane, corresponding to Fig. 7. (A) The 3D structure prediction of the ALDH1L2 protein was obtained from the AlphaFold database. (B–C) Molecular docking was used to simulate the interaction of the ALDH1L2 protein with the PRDX3 protein (B) and the TRX2 protein (C). The blue color represents the ALDH1L2 protein, the green color represents the PRDX3 protein, and the gray color represents the TRX2 protein. (D-E) Immunofluorescence detection of the colocalization of PRDX3 and Na+/K+ ATPase a1 in H69AR (D) and H446DDP (E) cells. The cell nuclei are labeled with blue fluorescence, the Na+/K+ ATPase a1 protein is labeled with green fluorescence, and the PRDX3 protein is labeled with red fluorescence. Scale bar: 20 μm. (F-G) Total cell lysates of H69AR (F) and H446DDP (G) cells were separated into to cytosolic, plasma membrane and organelle fractions (containing mitochondria) using the protein isolation and cell fractionation kit (Invent Biotechnologies, catalog number: SM-005). Immunoblotting was used to verify successful cell fractionation. GAPDH was used as an internal reference protein for the total and cytosolic fractions, and Na+/K+ ATPase a1 was used as an internal reference protein for the membrane fraction. PRDX3 is a mitochondrial protein; therefore, COX IV was used as an internal reference protein for the organelle fraction. [file mmc3.pdf]

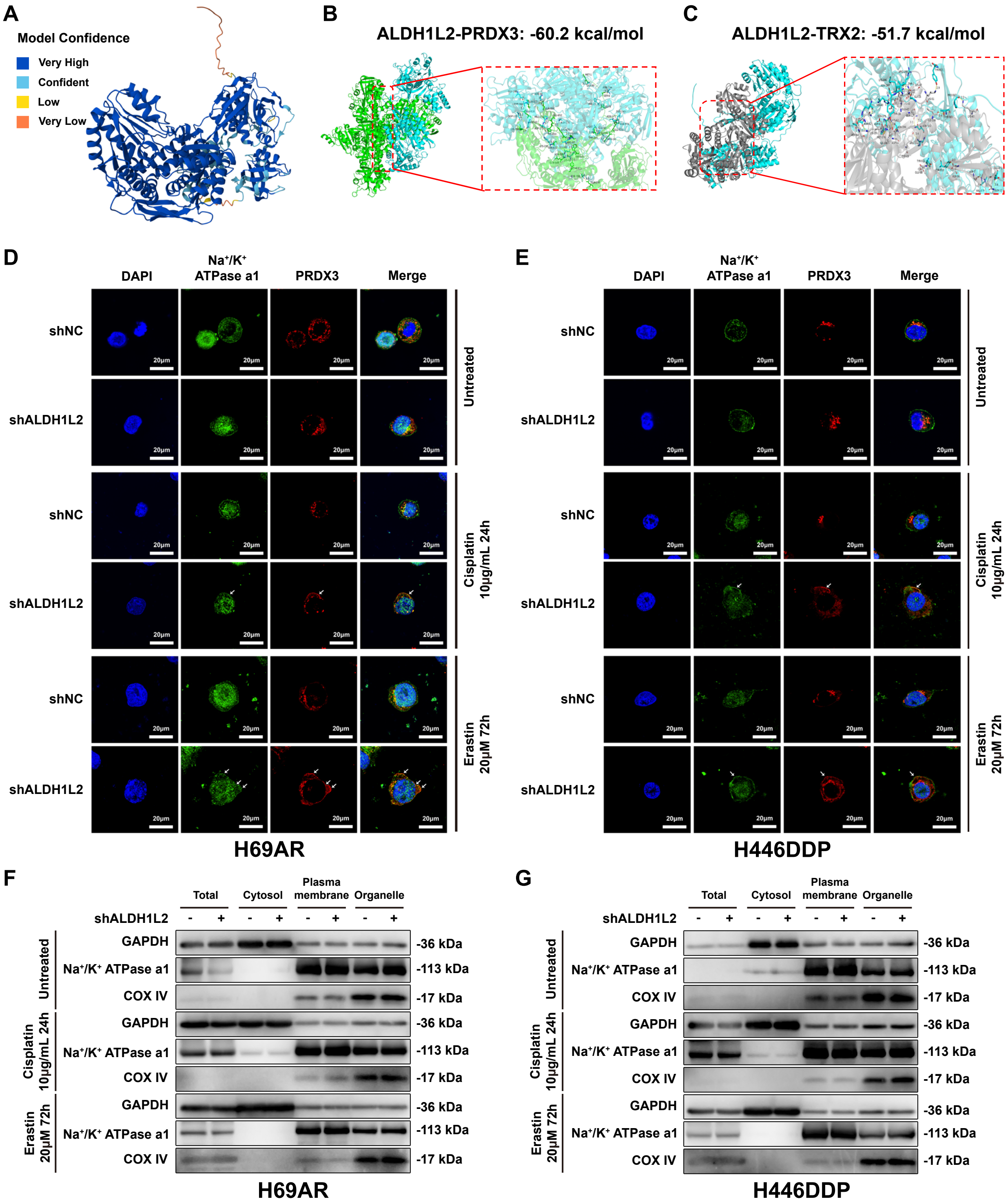
A
Model Confidence
Very High
Confident
Low
Very Low
B
ALDH1L2-PRDX3: -60.2 kcal/mol
C
ALDH1L2-TRX2: -51.7 kcal/mol
D
DAPI
Na⁺/K⁺ ATPase a1
PRDX3
Merge
shNC
shALDH1L2
Untreated
Cisplatin 10μg/mL 24h
Erastin 20μM 72h
20μm
H69AR
E
DAPI
Na⁺/K⁺ ATPase a1
PRDX3
Merge
shNC
shALDH1L2
Untreated
Cisplatin 10μg/mL 24h
Erastin 20μM 72h
20μm
H446DDP
F
Total
Cytosol
Plasma membrane
Organelle
shALDH1L2
- + - + - + - +
Untreated
Cisplatin 10μg/mL 24h
Erastin 20μM 72h
GAPDH
Na⁺/K⁺ ATPase a1
COX IV
-36 kDa
-113 kDa
-17 kDa
H69AR
G
Total
Cytosol
Plasma membrane
Organelle
shALDH1L2
- + - + - + - +
Untreated
Cisplatin 10μg/mL 24h
Erastin 20μM 72h
GAPDH
Na⁺/K⁺ ATPase a1
COX IV
-36 kDa
-113 kDa
-17 kDa
H446DDP
